# Author Correction: Bone morphogenetic protein 7 promotes resistance to immunotherapy

**DOI:** 10.1038/s41467-020-19083-3

**Published:** 2020-10-08

**Authors:** Maria Angelica Cortez, Fatemeh Masrorpour, Cristina Ivan, Jie Zhang, Ahmed I. Younes, Yue Lu, Marcos R. Estecio, Hampartsoum B. Barsoumian, Hari Menon, Mauricio da Silva Caetano, Rishab Ramapriyan, Jonathan E. Schoenhals, Xiaohong Wang, Ferdinandos Skoulidis, Mark D. Wasley, George Calin, Patrick Hwu, James W. Welsh

**Affiliations:** 1 Departments of Radiation Oncology, The University of Texas MD Anderson Cancer Center, Houston, TX USA; 2 Experimental Therapeutics, The University of Texas MD Anderson Cancer Center, Houston, TX USA; 3 Experimental Radiation Oncology, The University of Texas MD Anderson Cancer Center, Houston, TX USA; 4 Epigenetic and Molecular Carcinogenesis, The University of Texas MD Anderson Cancer Center, Houston, TX USA; 5 Thoracic/Head and Neck Medical Oncology, The University of Texas MD Anderson Cancer Center, Houston, TX USA; 6 Melanoma Medical Oncology, The University of Texas MD Anderson Cancer Center, Houston, TX USA

**Keywords:** Cancer therapeutic resistance, Cancer immunotherapy, Cancer therapeutic resistance

Correction to: *Nature Communications* 10.1038/s41467-020-18617-z, published online 24 September 2020.

The original version of this Article contained an error in the spelling of the author Ahmed I. Younes, which was incorrectly given as Ahmed Younes. This has now been corrected in both the PDF and HTML versions of the Article.

