# Supplementary material for: Bone morphogenetic protein 7 promotes resistance to immunotherapy
Source: Nat Commun. 2020 Sep 24;11:4840. doi: 10.1038/s41467-020-18617-z (PMC7519103; doi:10.1038/s41467-020-18617-z)
Supplement: Supplementary file 1 — Supplementary Information [file 41467_2020_18617_MOESM1_ESM.pdf]

Supplementary information

Bone morphogenetic protein 7 promotes resistance to immunotherapy

Cortez, MA *et al*

Supplementary Figure 1

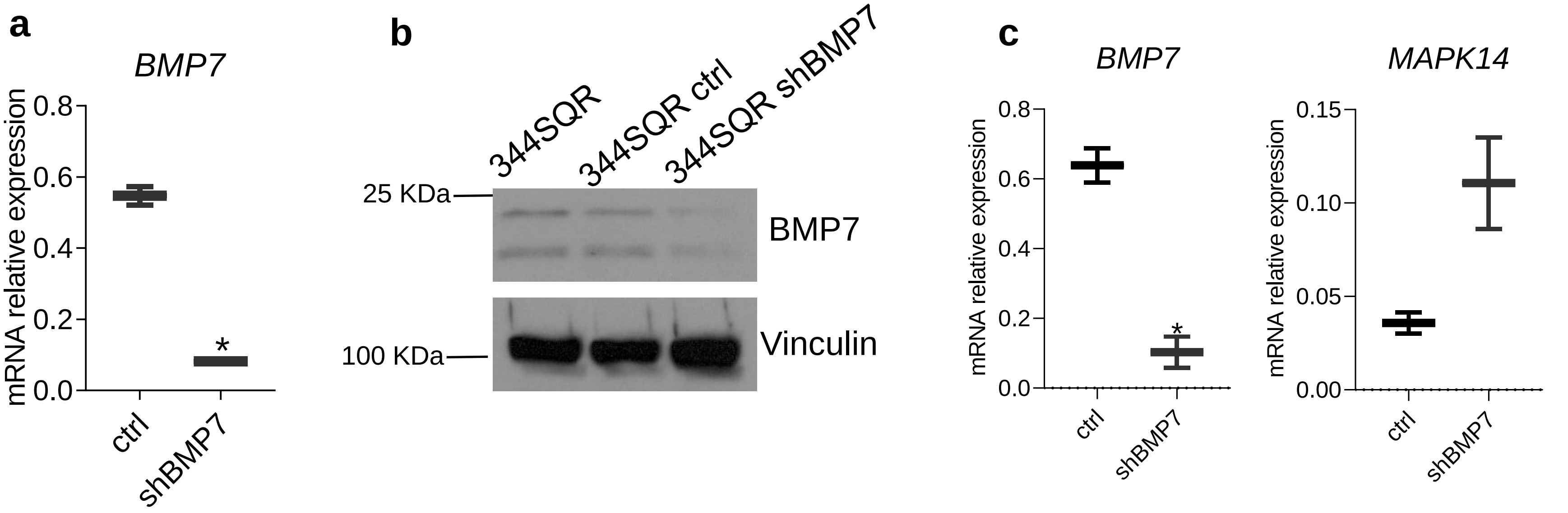

Supplementary Figure 1. **a,b** Establishment of 344SQR stable cell lines overexpressing shRNAs against *BMP7* (shBMP7) validated by quantitative PCR and western blotting. *ACTB* expression was used as a housekeeping gene for quantitative PCR. The comparative Ct method was used to calculate the relative abundance of mRNAs compared with *ACTB* expression. Box-and-whisker plots show the minimum and maximum from 2 biologically independent samples. BMP7, *p = 0.0296 (ctrl vs. shBMP7) unpaired, two-sided t tests. BMP7 mature form was detected by western blotting IN 344SQR, 344SQR control (non-targeting shRNA), and 344SQR shBMP7. Vinculin expression was used for normalization in western blotting. This experiment was repeated 2 times. **c**, *BMP7*-knockdown or –control cells (0.5×10^6) were injected into 129Sv/Ev mice and ctrl and shBMP7 tumors (n = 2 biologically independent samples for each cohort) were collected for *BMP7* and *MAPK14* expression analysis by quantitative PCR. *ACTB* expression was used as a housekeeping gene for quantitative PCR. The comparative Ct method was used to calculate the relative abundance of mRNAs compared with *ACTB* expression. Box-and-whisker plots show the minimum and maximum. *BMP7*, *p = 0.0296 (ctrl vs. shBMP7) unpaired, two-sided t tests.

Supplementary Figure 2

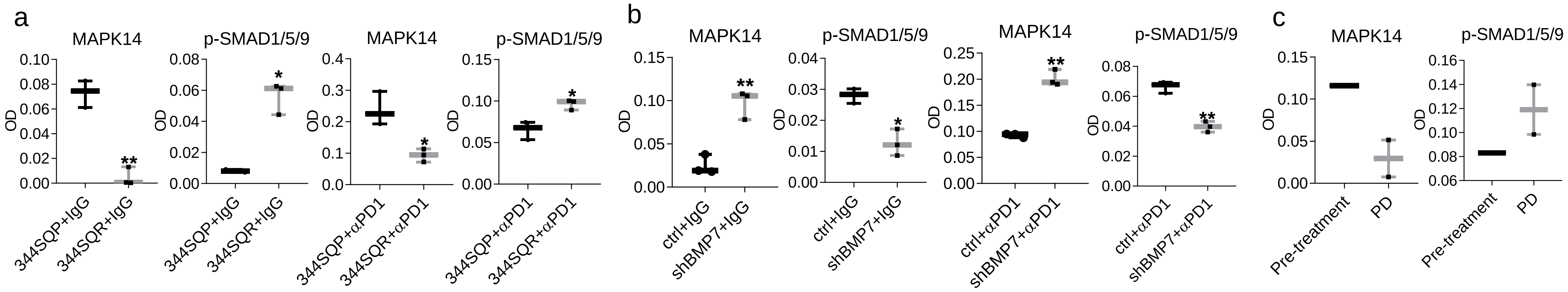

Supplementary Figure 2. Quantification of immunohistochemical stains for MAPK14, SMAD1/5/9 phosphorylation, and SMAD1 in formalin-fixed paraffin-embedded tissue sections using Fiji software. Optical Density (OD) numbers were obtained with the following formula: OD = log (max intensity/Mean intensity), where max intensity = 255 for 8-bit images. **a**, Quantification of immunohistochemical stains for MAPK14 and p-SMAD1/5/9 expression from 344SQP and 344SQR tumors treated with IgG or anti-PD1 (n = 3 biologically independent samples). MAPK14, $^{**}p$ = 0.0015, p-SMAD1/5/9, $^{*}p$ = 0.0140 (344SQP vs. 344SQR plus IgG) unpaired, two-sided t tests. MAPK14, $^{*}p$ = 0.0284, p-SMAD1/5/9, $^{*}p$ = 0.0203 (344SQP vs. 344SQR plus PD1) unpaired, two-sided t tests. Box-and-whisker plots show the minimum and maximum. Statistical significance was defined as *, $P<0.05$, **, $P<0.01$, ***$P<0.001$, and ****, $P<0.0001$. **b**, Quantification of immunohistochemical stains for MAPK14 and p-SMAD1/5/9 expression from 344SQR ctrl and 344SQR BMP7 knockdown tumors treated with IgG or anti-PD1 (n = 3 biologically independent samples). Box-and-whisker plots show the minimum and maximum. MAPK14, $^{**}p$ = 0.0051, p-SMAD1/5/9, $^{*}p$ = 0.0116 (ctrl vs. shBMP7 plus IgG) unpaired, two-sided t tests. MAPK14, $^{**}p$ = 0.0045, p-SMAD1/5/9, $^{**}p$ = 0.0010 (ctrl vs. shBMP7 plus PD1) unpaired, two-sided t tests. **c**, Quantification of immunohistochemical stains for MAPK14 and p-SMAD1/5/9 expression from patients that progressed (PD) on anti-PD1 or anti-CTLA-4 compared to pre-treatment biopsies (n = 2 biologically independent samples). Box-and-whisker plots show the minimum and maximum.

Supplementary Figure 3

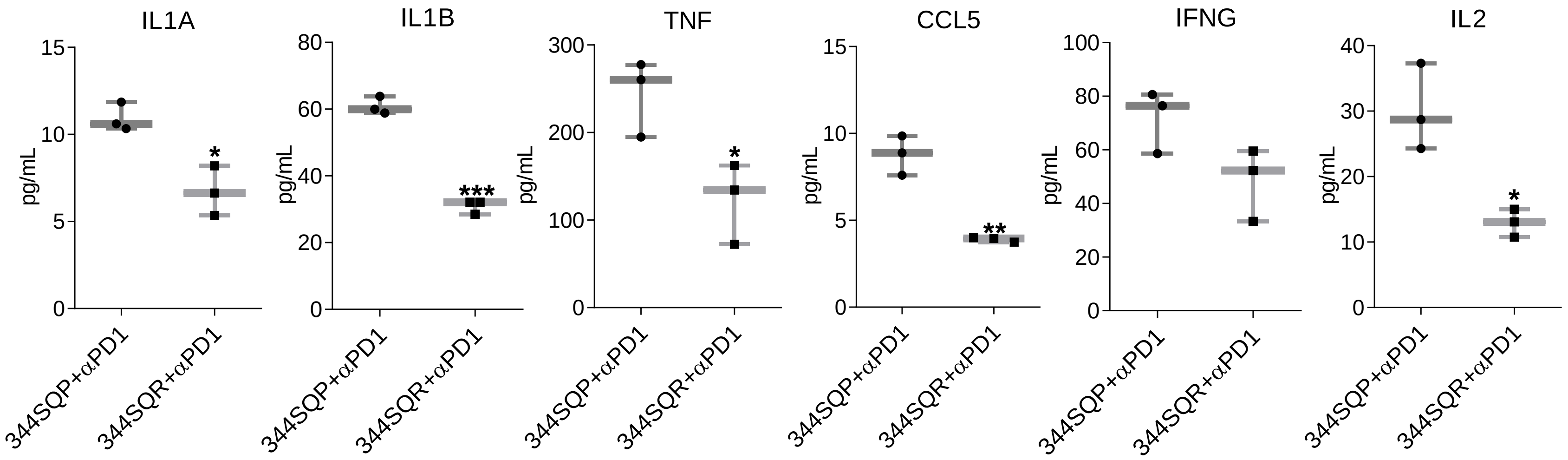

Supplementary Figure 3. Multiplex enzyme-linked immunosorbent assay for cytokines and chemokines in serum samples from mice bearing 344SQR (*n* = 3 biologically independent samples) or 344SQP tumors (*n* = 3 biologically independent samples) treated with anti-PD1 twice per week for a total of 4 doses. Box-and-whisker plots show the minimum and maximum. P values are from unpaired, two-sided t tests Statistical significance was defined as *, P<0.05, **, P<0.01, ***P<0.001, and ****, P<0.0001.

Supplementary Figure 4

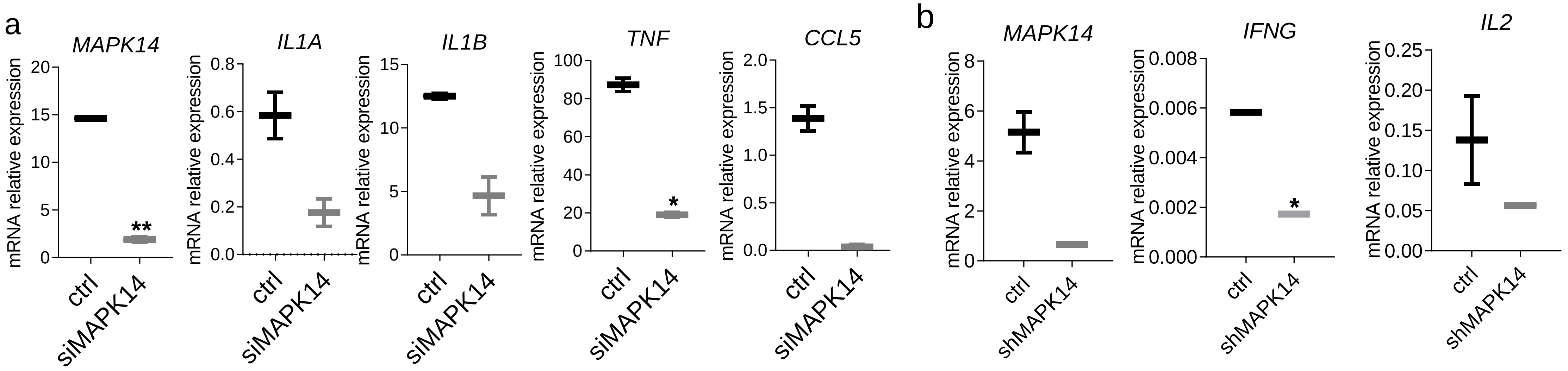

Supplementary Figure 4. **a**, Analysis of *MAPK14*, *IL1A*, *IL1B*, *TNF*, and *CCL5* expression in RAW 264.7 cells transfected with siRNAs ctrl and siMAPK14 (100 µM) targeting MAPK14 for 24 or 48 hours using quantitative PCR. Box-and-whisker plots show the minimum and maximum values from 2 independent experiments. **b**, Analysis of *MAPK14*, *IFNG* and *IL2* expression in EL4 cells stably overexpressing shRNAs targeting MAPK14 (EL4 shMAPK14) compared with control (EL4 ctrl) cells using quantitative PCR. CD45 expression was used as normalizer control. The comparative Ct method was used to calculate the relative abundance of mRNAs compared with CD45 expression. Box-and-whisker plots show the minimum and maximum values from 2 independent experiments. P values are from unpaired, two-sided t tests. Statistical significance was defined as *, P<0.05, **, P<0.01, ***P<0.001, and ****, P<0.0001.

Supplementary Figure 5

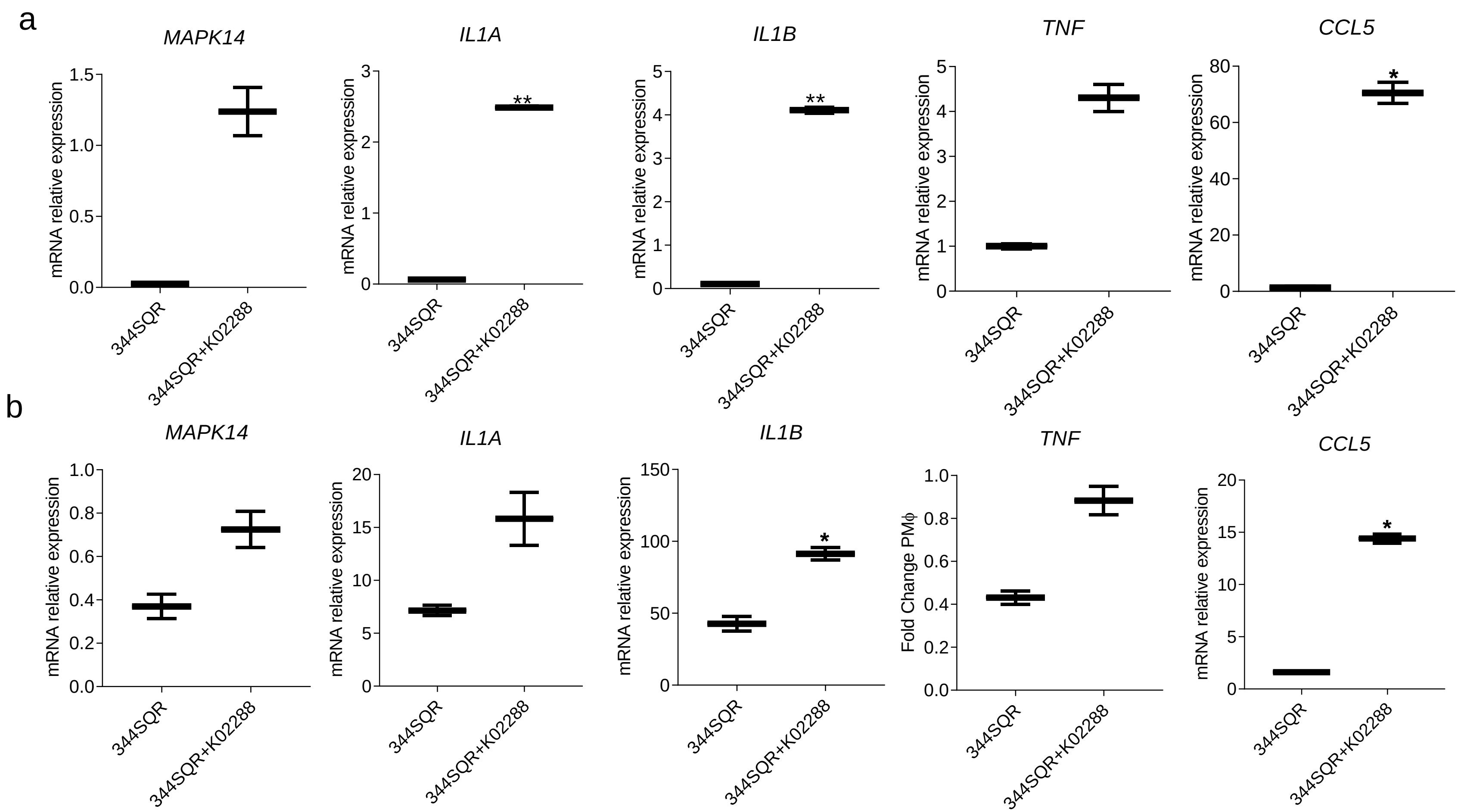

Supplementary Figure 5. a,b 344SQR cells were seeded at the top inserts, and RAW 264.7 cells (a) or peritoneal macrophages (b) were seeded at the bottom of the transwell system. Cells were then cultured in complete medium (RPMI-1640 supplemented with 100 units/mL penicillin, 100 µg/mL streptomycin, and 10% heat-inactivated fetal bovine serum) and incubated at 37°C in 5% CO_2 for 24 or 48 hours, after which cells were treated with K02288 (6.4 nM). *CD45* expression was used as a housekeeping gene for qPCR. The comparative Ct method was used to calculate the relative abundance of *MAPK14*, *IL1A*, *IL1B*, *TNF* and *CCL5* mRNAs compared with CD45 expression. Box-and-whisker plots show the minimum and maximum values from 2 independent experiments. P values are from unpaired, two-sided t tests. Statistical significance was defined as *, P<0.05, **, P<0.01, ***P<0.001, and ****, P<0.0001.

Supplementary Figure 6

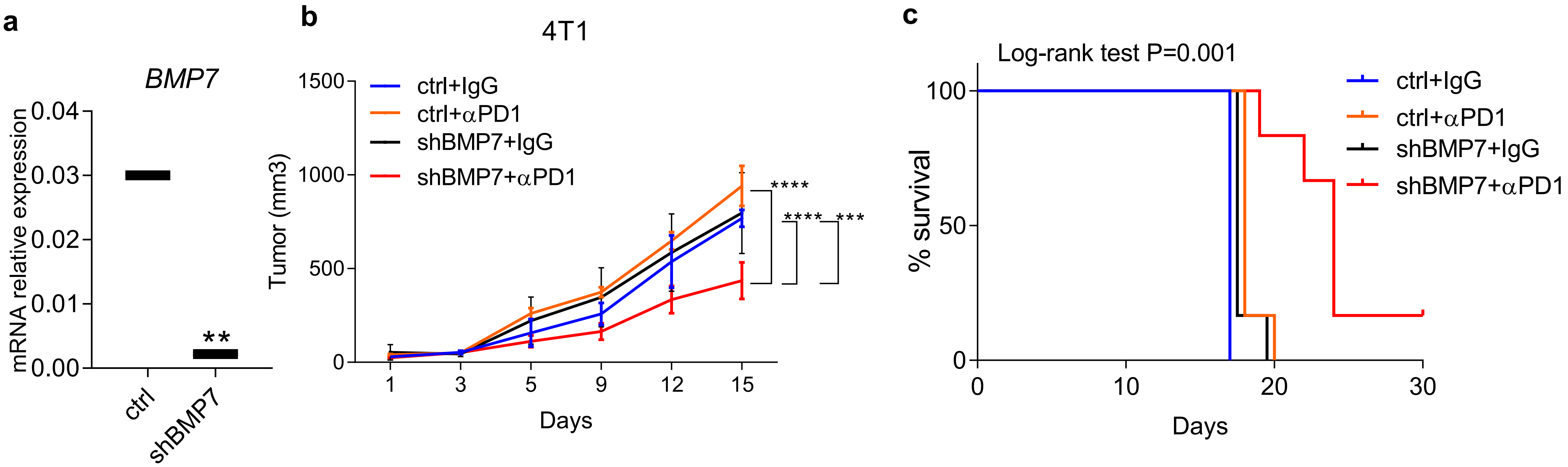

Supplementary Figure 6. **a**, Establishment of 4T1 stable cell lines overexpressing shRNAs against *BMP7* (shBMP7) validated by quantitative PCR. *ACTB* expression was used as a housekeeping gene for quantitative PCR. The comparative Ct method was used to calculate the relative abundance of mRNAs compared with *ACTB* expression. Box-and-whisker plots show the minimum and maximum values from 2 independent experiments. P values are from unpaired, two-sided t tests. **b,c**, Tumor growth and survival analysis of mice with 4T1 ctrl (n = 6 animals) or 4T1-shBMP7 (n=6 animals) tumors treated with IgG or anti-PD1 (10 mg/kg) twice a week for 3 weeks. ctrl+ IgG vs. shBMP7+αPD1, ****$p < 0.0001$, ctrl+ αPD1 vs. shBMP7+αPD1, ****$p < 0.0001$ shBMP7+IgG vs. shBMP7+αPD1, ***$p = 0.0002$, Two-way RM ANOVA. Mouse survival rates were analyzed by the Kaplan–Meier method and compared with log-rank test. Statistical significance was defined as *, P<0.05, **, P<0.01, ***P<0.001, and ****, P<0.0001.

Supplementary Figure 7

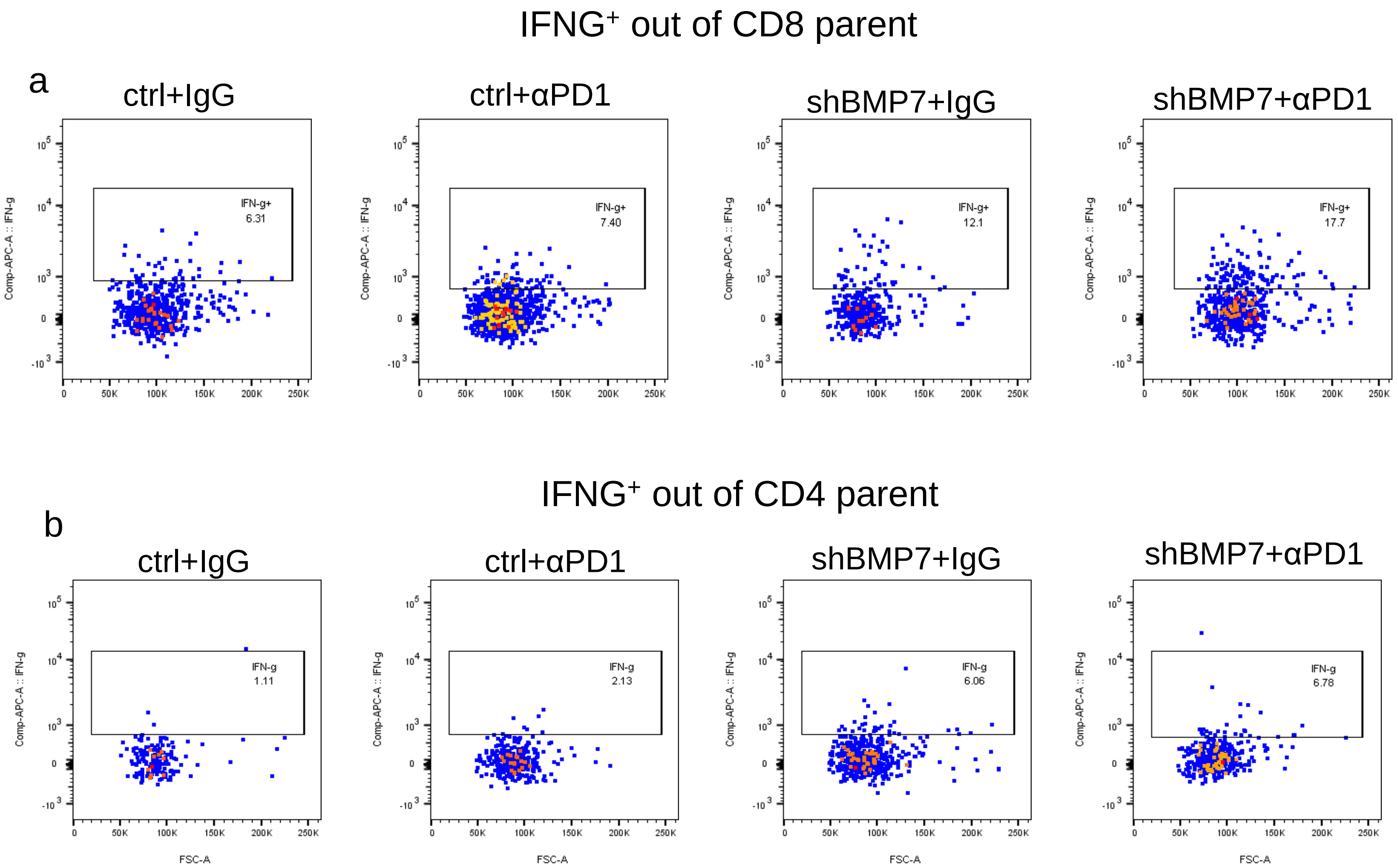

Supplementary Figure 7. a, Representative of Flow cytometry analysis of CD8$^+$IFNG$^+$ out of the CD8$^+$ T cells parent in tumor-infiltrating leucocytes (TILs) from 344SQR ctrl (*n* = 3 independent biological samples) and 344SQR-shBMP7 (*n* = 3 independent biological samples) tumors treated with IgG or anti-PD1 (10 mg/kg) twice a week for 2 weeks. Cells were first gated on lymphocytes, then CD45$^+$, then CD8$^+$ T cells, followed by IFNG$^+$ population. **b**, Representative of Flow cytometry analysis of CD4$^+$IFNG$^+$ out of the CD4$^+$ T cells parent in TILs from 344SQR ctrl (*n* = 3 independent biological samples) and 344SQR-shBMP7 (*n* = 3 independent biological samples) tumors treated with IgG or anti-PD1 (10 mg/kg) twice a week for 2 weeks. Cells were first gated on Lymphocytes, then CD45$^+$, then CD4$^+$ T cells, followed by IFNG$^+$ population.

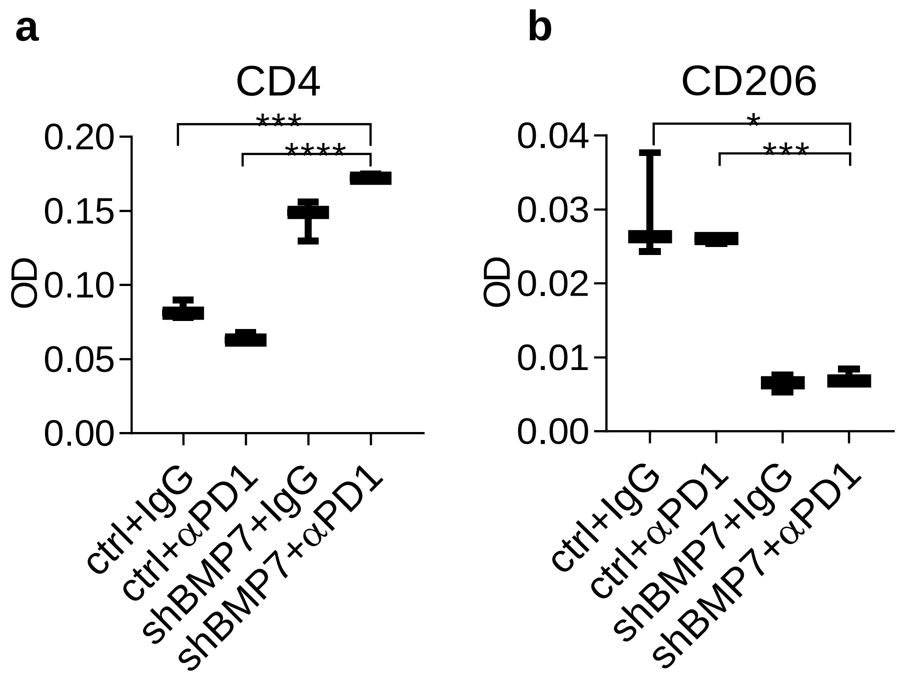

Supplementary Figure 8. Quantification of immunohistochemical stains for CD4 and CD206 in formalin-fixed paraffin-embedded tissue sections using Fiji software. Optical Density (OD) numbers were obtained with the following formula: OD = log (Max intensity/Mean intensity), where max intensity = 255 for 8-bit images. **a**, Quantification of immunohistochemical stains for CD4 T cells from 344SQR ctrl and 344SQR BMP7 knockdown tumors treated with IgG or anti-PD1 (n = 3 biologically independent samples). ***p = 0.0004 (ctrl + IgG vs. shBMP7+αPD1), ****p < 0.0001 (ctrl + αPD1 vs. shBMP7 + αPD1), unpaired, two-sided t tests. Box-and-whisker plots show the minimum and maximum. Statistical significance was defined as *, P<0.05, **, P<0.01, ***P<0.001, and ****, P<0.0001. **b**, Quantification of immunohistochemical stains for CD206 macrophages from 344SQR ctrl and 344SQR BMP7 knockdown tumors treated with IgG or anti-PD1 (n = 3 biologically independent samples). *p = 0.0319 (ctrl + IgG vs. shBMP7 + αPD1), ***p = 0.0001 (ctrl + αPD1 vs. shBMP7 + αPD1), unpaired, two-sided t tests. Box-and-whisker plots show the minimum and maximum. Statistical significance was defined as *, P<0.05, **, P<0.01, ***P<0.001, and ****, P<0.0001.

Supplementary Figure 9

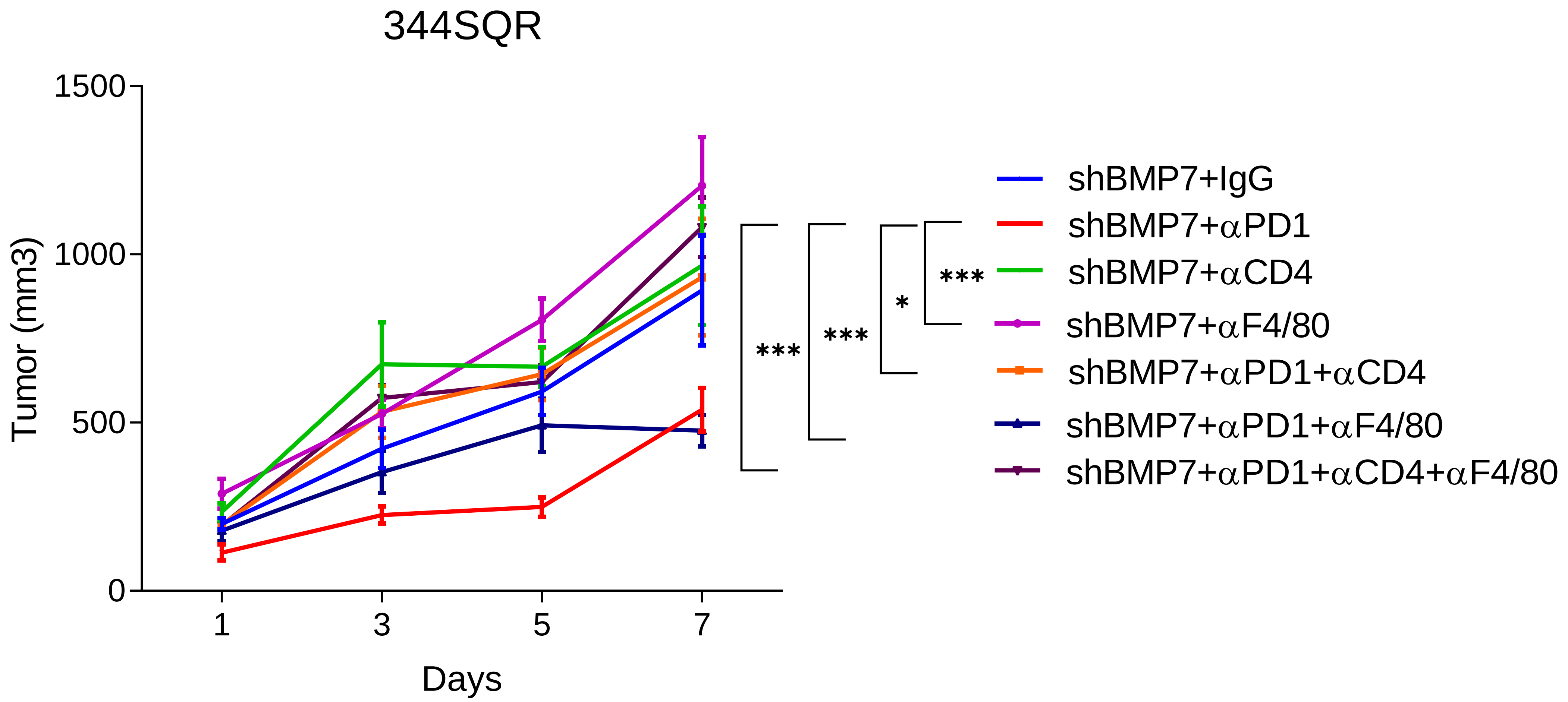

Supplementary Figure 9. Tumor growth and survival analysis of mice with 344SQR-shBMP7 (*n* = 4 animals or *n* = 5 animals) tumors treated with IgG, anti-PD1, anti-CD4 or anti-F4/80 (10 mg/kg) twice a week for 2 weeks. F4/80 (10 mg/kg) twice a week for 2 weeks. Tumor growth curves were compared Two-way RM ANOVA. Statistical significance was defined as *, P<0.05, **, P<0.01, ***P<0.001, and ****, P<0.0001. shBMP7+αPD1 vs. shBMP7+αF4/80, ****p* = 0.0003, shBMP7+αPD1 vs. shBMP7+αPD1+αCD4, **p* = 0.0282, shBMP7+αPD1 vs. shBMP7+αPD1+αF4/80, ****p* = 0.0002, shBMP7+αPD1 vs. shBMP7+αPD1+αCD4+αF4/80, ****p* = 0.0002.

Supplementary Figure 10

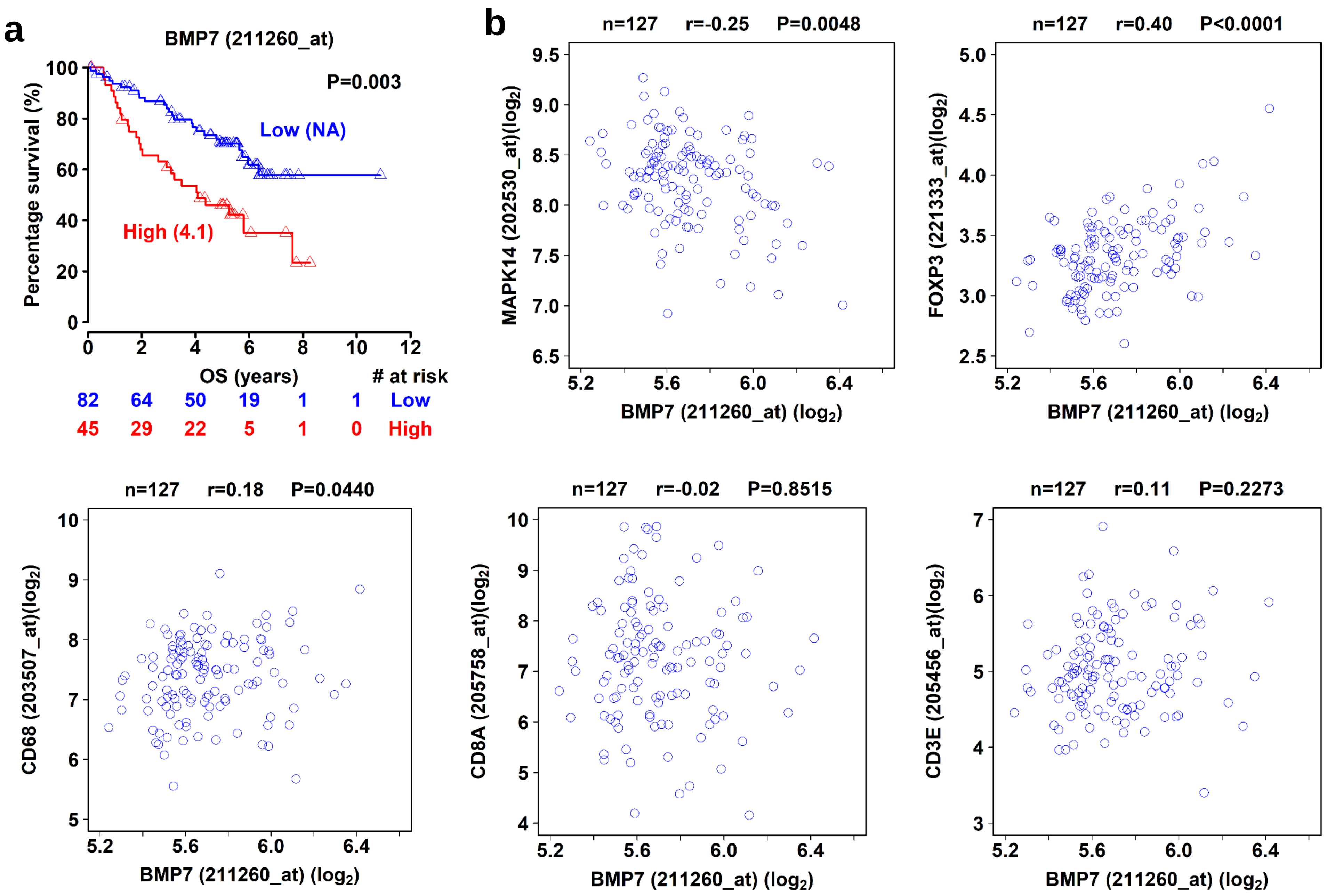

Supplementary Figure 10. **a**, *BMP7* is an independent marker of poor overall survival in NSCLC GSE9893 LUAD cohort. The Spearman's rank-order correlation test was applied to measure the strength of the association between different pairs of mRNA expression levels in tumor samples. P values were given by log-rank test, two-sided. **b**, Analysis of *MAPK14*, *FOXP3*, *CD68*, *CD8A*, *CD3E* correlation with *BMP7* expression in samples from GSE9893 LUAD cohort. Correlation analysis for mRNA data for TCGA Lung Adenocarcinoma cohort was performed in R (version 3.5.1; http:///www.r-project.org/).

Supplementary Figure 11

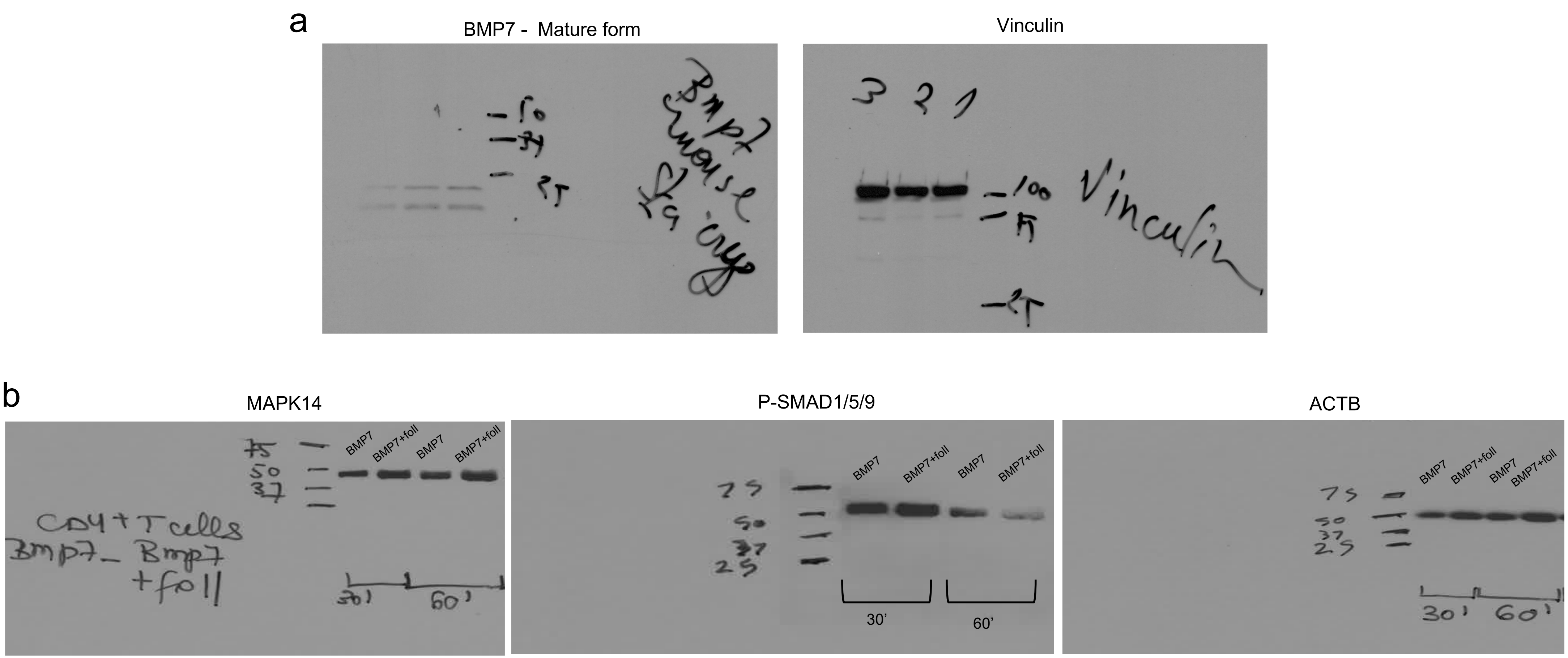

Supplementary Figure 11. Uncropped images of immunoblots from Supplementary Figure 1 (a) and Figure 4 (b).

Supplementary Figure 12

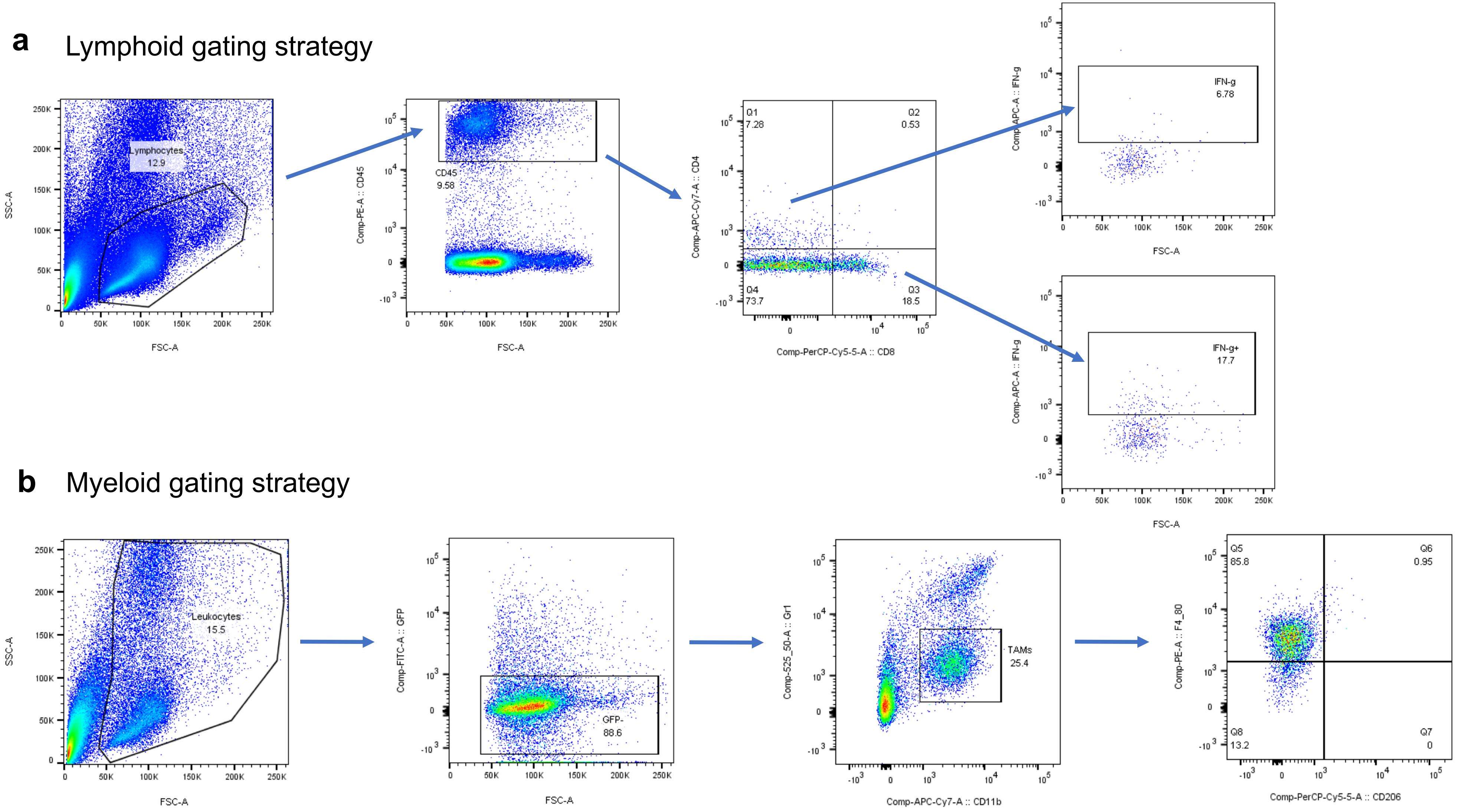

Supplementary Figure 12. **a**, Lymphoid population gating: Cells were gated on lymphocytes, then on CD45$^+$, then on CD4 and CD8 T cells. IFNG was then gated on either CD4 parent population or CD8 parent population, and percentages were recorded accordingly. **b**, Myeloid population gating: Cells were first gated on total leukocytes, then on GFP-negative cells (since tumor cells were GFP-positive). Tumor associated macrophages (TAMs) were then gated on Gr1high CD11b intermediate population, followed by F4/80$^+$ and CD206$^+$ gating for M2 subpopulation.

Supplementary Table 1. Microarray data for BMP7 expression in 344SQP versus 344SQR tumors treated with anti-PD1. The analysis was performed in R using functions in LIMMA library. A linear model was fitted to each gene and empirical Bayes methods were used to obtain the statistics. Data analysis is two-sided.

Gene.Symbol	Gene.Title	Representative.Public.ID	ENTREZ_GENE_ID	efSeq.Transcript.I	probe	Average 344SQR	Average 344SQP	P.Value	FDR	FCH
Bmp7	bone morphogenetic protein 7	NM_007557	12162	NM_007557	1418910_at	6.643333333	3.17	0.000000026643	0.000122277	11.1065076

Supplementary Table 2. Pyrosequencing methylation assay (PMA) primers and assay conditions

Primer Name	Primer Sequence	PCR Size	Sequence to Analyze
Bmp7/F1	ATTTGTAGGTTTGTAAGTTG	150bp	TTTGTYGTGY GTTTTGGYGA GTGYGGGTYG A
Bmp7/Rbio1	CCCTATCCTTTACTCAATT		
Bmp7/S1	GGAGTTGTTGTGTTAGT		

PCR Reaction	Volume (uL)
Bisulfite DNA	2
2x Zymo Reaction Buffer	7.5
dNTP Mix (25mM each)	0.15
F primer (10uM)	0.3
R primer (10uM)	0.3
Zymo Taq	0.12
DW	4.63

Cycling conditions

Step	Temp (oC)	Duration
1	95	10 m
2	95	30 s
3	55	30s
4	72	30s
5	Repeat (steps 2-4) 45 times	
6	72	7 m
7	12	infinite

**Supplementary Table 3. Reverse Phase Protein Array (RPPA) analysis in 344SQP versus 344SQR tumors treated with anti-PD1**						
Name	344SQR+anti-PD1	344SQP+anti-PD1	FCH_Treat	P.Value	FDR	
CDKN2A(p16INK4a)	0.518330427	1.483368476	-1.95212	1.32139E-06	0.000321	
STAT3(Stat3_pY705)	0.868652785	1.137474558	-1.20482	0.000548188	0.066605	
GZMB(Granzyme-B)	0.933295058	1.171671519	-1.17966	0.012649596	0.229168	
GAB2(Gab2)	0.875751417	1.109417935	-1.17582	0.014146152	0.229168	
AKT1(Akt_pS473)	0.948901202	1.171942821	-1.16719	0.017004134	0.229556	
GSK3A GSK3B(GSK-3a-b_pS21_S9-R-V)	0.87736123	1.080803392	-1.15144	0.048659823	0.369511	
PRKCB(PKC-b-II_pS660-R-V)	0.871850633	1.071833362	-1.14868	0.000907554	0.073512	
MAPK14(p38-MAPK-R-V)	0.905617679	1.101034918	-1.14506	0.01066066	0.229168	
ATG7(Atg7-R-V)	0.896793114	1.053614705	-1.11483	0.009409163	0.229168	
PTEN(PTEN-R-V)	0.896097906	1.029583482	-1.09694	0.020794784	0.240625	
PIK3R1(PI3K-p85-R-V)	0.928080999	1.058985795	-1.09498	0.01202774	0.229168	
YAP1(YAP_pS127-R-E)	0.902019999	1.023365166	-1.08775	0.025360697	0.28012	
BRAF(B-Raf_pS445-R-V)	0.950656284	1.070526522	-1.08664	0.036980722	0.295398	
RPS6K(p90RSK_pT573-R-C)	0.94457235	1.057634598	-1.08152	0.033410093	0.286374	
AKT1(Akt_pT308-R-V)	0.953960797	1.066955415	-1.08147	0.03344592	0.286374	
ATG3(Atg3-R-V)	0.941715848	1.034591556	-1.06649	0.033288299	0.286374	

Supplementary Table 3. Reverse Phase Protein Array (RPPA) analysis in 344SQP versus 344SQR tumors treated with anti-PD1

Name	344SQR+anti-PD1	344SQP+anti-PD1	FCH_Treat	P.Value	FDR
HIST3H3(Histone-H3-R-V)	1.436185685	0.823742326	1.528846	0.031329	0.286374
RB1(Rb_pS807_S811-R-V)	1.162809709	0.95184406	1.157463	0.011231	0.229168
EEF2(eEF2-R-C)	1.089460833	0.887669061	1.150126	0.006366	0.229168
PAICS(PAICS-R-C)	1.140026763	0.974919263	1.12125	0.008233	0.229168
IGF1R(IGFRb-R-C)	1.033271991	0.868931095	1.120654	0.016244	0.229556
EIF4EBP1(4E-BP1-R-V)	1.142311656	0.994165913	1.108144	0.034176	0.286374
CTNNB1(b-Catenin-R-V)	1.028320415	0.885804872	1.103828	0.033091	0.286374
XBP1(XBP-1-G-C)	1.113259085	0.978732559	1.097732	0.009922	0.229168
PARP1(PARP1-R-V)	1.059064439	0.934784201	1.089964	0.009685	0.229168
SOX2(Sox2-R-V)	1.090813985	0.971738257	1.086039	0.013587	0.229168
IRF1(IRF-1-R-C)	1.076926249	0.957971571	1.085948	0.018926	0.240625
INSRB(IR-b-R-C)	1.050014853	0.936809066	1.081629	0.009123	0.229168
INPP4B(INPP4b-R-V)	1.079594442	0.967300694	1.080945	0.020113	0.240625
YBX1(YB1_pS102-R-V)	1.040302935	0.934263403	1.07627	0.016507	0.229556
CDH1(E-Cadherin-R-V)	1.056240224	0.974273332	1.05846	0.037684	0.295398
XRCC1(XRCC1-R-C)	1.039682342	0.96386856	1.053955	0.029559	0.286374

Supplementary Table 3. Reverse Phase Protein Array (RPPA) analysis in 344SQP versus 344SQR tumors treated with anti-PD1

Name	344SQR+anti-PD1	344SQP+anti-PD1	FCH_Treat	P.Value	FDR
YWHAB(14-3-3-beta-R-V)	1.016002128	1.024421077	-1.00585	0.798488	0.927787
YWHAZ(14-3-3-zeta-R-V)	1.028655394	1.02228535	1.004425	0.88257	0.940925
EIF4EBP1(4E-BP1_pS65-R-V)	1.022493271	0.992447891	1.021044	0.402201	0.723962
TP53BP1(53BP1-R-V)	0.988359257	0.987077383	1.000889	0.971717	0.979781
ARAF(A-Raf-R-V)	0.987630892	1.054465821	-1.04742	0.266061	0.646652
ACACA(ACC_pS79-R-V)	0.96368948	0.985753065	-1.01541	0.814663	0.932783
ACACA(ACC1-R-C)	1.007859117	0.992369001	1.010795	0.816886	0.932783
AKT1(Akt-R-V)	0.952754134	1.020599935	-1.04815	0.389478	0.719895
PRKAA2(AMPK-a2_pS345-R-V)	0.979622939	0.987608868	-1.00555	0.796057	0.927787
PRKAA1(AMPKa-R-C)	0.992353405	1.020399884	-1.01963	0.671771	0.854059
PRKAA1(AMPKa_pT172-R-C)	0.880358921	1.017418917	-1.09966	0.096566	0.472879
AR(AR-R-V)	0.988904614	1.014568413	-1.01795	0.424174	0.735275
ARID1A(ARID1A-R-C)	0.988133794	0.847941263	1.102052	0.101829	0.472879
ATM(ATM-R-V)	1.025127921	1.052258819	-1.01898	0.434305	0.738015
ATM(ATM_pS1981-R-V)	1.067890375	0.998852552	1.049017	0.281373	0.646652
ATR(ATR_pS428-R-C)	0.988908409	1.02313449	-1.02401	0.417265	0.734749
ATRX(ATRX-R-C)	1.099145079	0.934138038	1.121172	0.078321	0.4396
AIM1(Aurora-B-R-V)	0.920699364	1.074181901	-1.11225	0.130334	0.510824
AXL(Axl-R-V)	0.982548877	1.029296439	-1.03293	0.550004	0.802202
ACTB(b-Actin-R-C)	1.170498301	1.053955963	1.084133	0.368311	0.710314
CTNNB1(b-Catenin_pT41_S45-R-V)	1.018773532	1.00180628	1.01183	0.681842	0.854059
BRAF(B-Raf-R-V)	0.99088914	0.987143422	1.0026	0.899062	0.942317
VTCN1(B7-H4-R-C)	0.974558176	1.003007247	-1.01992	0.499928	0.783759
BAD(Bad_pS112-R-V)	1.002770252	1.025186279	-1.01566	0.563692	0.806855
BAK1(Bak-R-C)	0.987960853	0.980415891	1.005243	0.835545	0.936284
BAX(Bax-R-V)	1.077295599	1.035916305	1.029097	0.412341	0.731378
BCL2L1(Bcl-xL-R-V)	1.072152394	1.00775315	1.045649	0.271078	0.646652
BCL2A1(Bcl2A1-R-V)	1.038627528	0.988396466	1.035431	0.174699	0.598242
BECN1(Beclin-G-C)	0.97121917	0.955451662	1.010989	0.688061	0.856598
BID(Bid-R-C)	1.069559088	1.018547454	1.035991	0.664801	0.850246
BCL2L11(Bim-R-V)	1.058804037	1.012297218	1.032761	0.304705	0.685587
BRD4(BRD4-R-V)	0.93863114	1.03635161	-1.07008	0.167561	0.590107
ABL1(c-Abl-R-V)	1.007277938	1.048021832	-1.02864	0.398465	0.722589
BIRC3(c-IAP2-R-C)	1.029015693	1.029176145	-1.00011	0.997353	0.997353
JUN(c-Jun_pS73-R-V)	1.021545965	0.948861806	1.051672	0.112377	0.472879
KIT(c-Kit-R-V)	0.957539432	0.975991617	-1.01287	0.654457	0.8475
MET(c-Met_pY1234_Y1235-R-V)	1.006780879	0.99358585	1.009188	0.717336	0.867228
MYC(c-Myc-R-C)	1.055993942	1.003949879	1.036733	0.231218	0.631598
RAF1(C-Raf-R-C)	1.029379365	1.035219457	-1.00406	0.908434	0.942317
RAF1(C-Raf_pS338-R-V)	0.966528605	1.00593716	-1.02769	0.391054	0.719895
CASP3(Caspase-3-R-C)	0.972927237	1.028239439	-1.03908	0.212572	0.622349
CASP7(Caspase-7-cleaved-R-C)	1.173166441	1.761179829	-1.50318	0.179433	0.598242
CAV1(Caveolin-1-R-V)	0.989501817	0.941955829	1.033505	0.395561	0.722589
DPP4(CD26-R-V)	1.040580098	1.002952997	1.026424	0.259332	0.646652
CDK1(Cdc2_pY15-R-C)	0.993601833	0.991874504	1.001198	0.9596	0.97566
CDC25C(cdc25C-R-V)	0.970252631	1.014104394	-1.03086	0.250269	0.646652
CDK1(CDK1-R-C)	1.052982997	0.961048817	1.065798	0.216639	0.626706
CHEK1(Chk1_pS296-R-V)	1.093197425	1.177885439	-1.06046	0.343878	0.698143
CHEK2(Chk2_pT68-R-C)	0.953794953	0.999437768	-1.03214	0.333506	0.695756
CLDN7(Claudin-7-R-V)	0.991265204	1.016330462	-1.01753	0.5151	0.786164
COG3(COG3-R-V)	0.98318969	1.016250248	-1.02318	0.331497	0.695756
COL6A1(Collagen-VI-R-V)	1.048253879	1.000094322	1.033945	0.261295	0.646652
CNST43(Connexin-43-R-C)	0.985765548	0.864853037	1.087422	0.209126	0.619726
PTGS3(Cox-IV-R-V)	1.025486305	1.088073707	-1.04434	0.630053	0.832081
CMC2(Cox2-R-C)	1.05297881	0.969169652	1.059813	0.185684	0.598242
CREB1(Creb-R-C)	1.03502034	0.998093815	1.025926	0.485198	0.771439
CCNB1(Cyclin-B1-R-V)	0.994138089	0.970321764	1.016645	0.542331	0.802202
CCND1(Cyclin-D1-R-V)	0.956202151	0.981525816	-1.01771	0.488895	0.771439
TUBA1A(D-a-Tubulin-R-V)	0.998141741	0.991239209	1.004796	0.854688	0.93977

PARK7(DJ1-R-V)	1.018744176	0.977535643	1.028975	0.466183	0.771439	
HISTH3(DM-Histone-H3-R-V)	1.039263553	1.004907517	1.0241	0.547003	0.802202	
H3K9ME2(DM-K9-Histone-H3-R-C)	1.091679941	0.937140852	1.113066	0.188984	0.598242	
DUSP4(DUSP4-R-V)	0.982154817	0.977472591	1.003251	0.906356	0.942317	
EEF2K(eEF2K-R-V)	0.977134486	1.007571412	-1.02132	0.564466	0.806855	
EGFR(EGFR-R-V)	1.100941907	1.04860111	1.036946	0.180583	0.598242	
EGFR(EGFR_pY1173-R-V)	1.044711467	1.014124731	1.021427	0.61508	0.829313	
EIF4E(eIF4E-R-V)	1.002017984	0.999816532	1.001527	0.965107	0.977171	
EIF4E(eIF4E_pS209-R-V)	0.996282173	1.030822262	-1.02423	0.267794	0.646652	
EIF4G1(eIF4G-R-C)	1.027001929	0.982611415	1.031247	0.348167	0.698143	
ELK1(Elk1_pS383-R-C)	1.038820927	0.992298898	1.032772	0.316575	0.693043	
ESR1(ER-a_pS118-R-V)	1.015067485	0.978698964	1.025529	0.255652	0.646652	
ESR1(ER-R-V)	0.969414109	0.983513274	-1.00982	0.659167	0.8475	
ERCC5(ERCC5-R-C)	1.026028375	0.963403842	1.044364	0.109643	0.472879	
ETS1(Ets-1-R-V)	1.047509155	1.09466607	-1.03323	0.604805	0.829313	
PTK2(FAK-R-C)	1.064566412	1.021269196	1.030466	0.458085	0.771439	
PTK2(FAK_pY397-R-V)	0.925984101	0.977020546	-1.03601	0.219258	0.626821	
FASN(FASN-R-V)	0.966554928	0.969596856	-1.00211	0.956326	0.97566	
FOXM1(FoxM1-R-V)	1.005548319	0.996730653	1.006131	0.870797	0.940925	
FOX3(FoxO3a-R-C)	0.975233993	1.010805884	-1.02496	0.261519	0.646652	
FOXO3(FoxO3a_pS318_S321-R-C)	0.994672797	1.024138302	-1.02063	0.329374	0.695756	
FOSL1(FRA-1-R-C)	1.031895917	0.983406773	1.034181	0.204203	0.612609	
G6PD(G6PD-R-V)	0.974359454	1.012843706	-1.02703	0.429232	0.735275	
GCLM(GCLM-R-C)	1.063532972	1.069173906	-1.00392	0.931415	0.959042	
KAT2A(GCN5L2-R-V)	1.024131272	0.945258984	1.056192	0.075911	0.4396	
GLUD(Glutamate-D1-2-R-C)	1.119274686	1.072088987	1.033247	0.334994	0.695756	
GLS(Glutaminase-R-C)	0.975839153	1.017313751	-1.02917	0.355714	0.698143	
GYS1(Gys-R-V)	0.884331636	1.170858898	-1.2197	0.053403	0.381675	
GYS1(Gys_pS641-R-V)	1.005470069	1.026934943	-1.01499	0.47455	0.771439	
ERBB2(HER2_pY1248-R-C)	0.98131649	0.987946724	-1.00461	0.824226	0.933549	
ERBB3(HER3-R-V)	1.003233239	0.89462598	1.078187	0.05951	0.403529	
ERBB3(HER3_pY1289-R-C)	1.000446493	0.989267759	1.007779	0.745926	0.88853	
NRG1(Heregulin-R-V)	1.013182652	1.0855781	-1.05146	0.706092	0.860908	
HES1(HES1-R-V)	1.110359457	0.971741092	1.10085	0.061443	0.403529	
HK2(Hexokinase-II-R-V)	1.073242286	1.013486951	1.042289	0.151747	0.551293	
HSBP1(HSP27_pS82-R-V)	1.024515394	0.927376355	1.06965	0.092762	0.472879	
HSPA1A(HSP70-R-C)	1.066025078	0.978595941	1.062475	0.241396	0.644607	
IGF1R(IGF1R_pY1135_Y1136-R-V)	0.998555854	1.013133689	-1.01016	0.744994	0.88853	
IGFBP2(IGFBP2-R-V)	1.01522399	0.972366127	1.030152	0.33131	0.695756	
IRS1(IRS1-R-V)	1.036407631	0.97485262	1.04359	0.081407	0.4396	
JAG1(Jagged1-R-V)	0.980675014	0.986606575	-1.00412	0.901371	0.942317	
JAK2(Jak2-R-V)	1.02118038	1.012038216	1.006357	0.847558	0.936284	
MAPK8(JNK_pT183_Y185-R-V)	1.014312759	1.03124293	-1.0118	0.739399	0.88853	
MAPK9(JNK2-R-C)	1.067353565	1.007243538	1.042545	0.275632	0.646652	
LC3AB(LC3A-B-R-C)	1.095161397	1.040187792	1.03884	0.625472	0.832081	
LCK(Lck-R-V)	1.064125342	1.145561706	-1.05807	0.515763	0.786164	
LDHA(LDHA-R-C)	1.028392572	1.007071033	1.014889	0.550534	0.802202	
LRP6(LRP6_pS1490-R-V)	0.98680145	0.98209026	1.003271	0.880752	0.940925	
MAPK3(MAPK_pT202_Y204-R-V)	0.983583957	1.011325783	-1.01942	0.466932	0.771439	
MCL1(Mcl-1-R-V)	1.028754449	1.073964699	-1.03183	0.281495	0.646652	
SLC16A4(MCT4-R-V)	0.934075922	1.047434817	-1.08174	0.11676	0.472879	
MDM2(MDM2_pS166-R-V)	1.041605992	0.924585576	1.084493	0.186244	0.598242	
MAP2K1(MEK1-R-V)	0.961608679	0.981609433	-1.01396	0.587009	0.825373	
MAP2K1(MEK1_pS217_S221-R-V)	1.002281658	1.021248407	-1.01323	0.580344	0.824699	
BABAM1(MERIT40_pS29-R-V)	1.047978547	1.038332008	1.006709	0.881191	0.940925	
NF2(Merlin-R-C)	1.027693402	1.008664078	1.013277	0.700516	0.859724	
MIF(MIF-R-C)	0.951905293	1.025376485	-1.05225	0.113547	0.472879	
MMP2(MMP2-R-V)	0.983355392	1.061915373	-1.05596	0.232098	0.631598	
MKNK1(Mnk1-R-V)	0.969287745	1.038565145	-1.04919	0.080138	0.4396	
MSH6(MSH6-R-C)	1.046354004	0.958478116	1.062804	0.07409	0.4396	
MSI2(MSI2-R-C)	0.928088801	1.029988346	-1.07319	0.097057	0.472879	

MTOR(mTOR-R-V)	0.940080999	1.02928876	-1.06379	0.228853	0.631598	
MTOR(mTOR_pS2448-R-C)	0.97228794	0.999780916	-1.01924	0.65721	0.8475	
MYH11(Myosin-11-R-V)	0.976519925	0.944339874	1.022556	0.838654	0.936284	
MYO2A(Myosin-IIa_pS1943-R-V)	0.999260675	1.044235308	-1.03167	0.503666	0.784556	
MYT1(Myt1-R-C)	0.964113035	1.025685177	-1.0436	0.107804	0.472879	
CDH2(N-Cadherin-R-V)	0.994718216	1.018444714	-1.01658	0.517639	0.786164	
NAPSA(NAPSIN-A-R-C)	0.993411284	1.005618842	-1.0085	0.694444	0.856598	
NDRG1(NDRG1_pT346-R-V)	0.993916987	1.16248877	-1.12395	0.101667	0.472879	
NFKB1(NF-kB-p65_pS536-R-C)	0.996240175	0.976491769	1.013783	0.532339	0.802202	
NOTCH1(Notch1-R-V)	0.974206752	0.926051509	1.033942	0.587612	0.825373	
NOTCH3(Notch3-R-C)	1.029483165	1.010140911	1.013497	0.680775	0.854059	
4-Oct(Oct-4-R-C)	0.990873588	0.960964203	1.020948	0.353496	0.698143	
CDH3(P-Cadherin-R-C)	1.015572464	0.994779638	1.014517	0.617719	0.829313	
CDKN1A(p21-R-V)	0.991062962	1.012186869	-1.01475	0.551308	0.802202	
CDKN1B(p27-Kip-1-R-V)	0.988768988	1.006850162	-1.01261	0.638686	0.838731	
CDKN1B(p27_pT198-R-V)	1.050516863	1.016055482	1.024174	0.389604	0.719895	
MAPK14(p38_pT180_Y182-R-V)	0.850377445	0.990610257	-1.10208	0.080331	0.4396	
MAPK3(p44-42-MAPK-R-V)	0.996986421	1.025638479	-1.02006	0.545931	0.802202	
TP53(p53-R-C)	1.027396405	1.000620326	1.018733	0.598117	0.829313	
RPS6KB1(p70-S6K_pT389-R-V)	0.954456502	1.03168225	-1.05499	0.059907	0.403529	
RPS6KB1(p70-S6K1-R-V)	0.975649312	0.998715327	-1.01612	0.629968	0.832081	
PAK1(PAK1-R-V)	1.00149347	0.966183973	1.024777	0.614633	0.829313	
PAK4(PAK4-R-V)	0.99633473	1.040664892	-1.0312	0.343484	0.698143	
PAR(PAR-R-C)	1.272138179	0.890327757	1.302976	0.118981	0.473972	
PAX8(PAX8-R-C)	0.95018932	0.962507532	-1.00857	0.692074	0.856598	
PXN(Paxillin-R-C)	0.994747861	0.9293991	1.046338	0.310368	0.685632	
CD274(PD-L1-R-C)	0.919877397	1.048868038	-1.09353	0.612986	0.829313	
PDCD1(Pdcd-1L1-G-C)	1.06479464	0.966124689	1.070786	0.052675	0.381675	
PDCD4(Pdcd4-R-C)	0.926746113	0.987879352	-1.04328	0.203407	0.612609	
PDGFR(PDGFR-b-R-V)	0.947288953	0.939073766	1.005711	0.905474	0.942317	
PDHK1(PDHK1-R-C)	0.9414977	1.00239073	-1.04311	0.255081	0.646652	
PDK1(PDK1-R-V)	0.843808468	1.046666381	-1.15098	0.279915	0.646652	
PDK1(PDK1_pS241-R-V)	1.070629004	0.986038636	1.060387	0.113261	0.472879	
PEA15(PEA-15-R-V)	1.036825395	1.0255523	1.007845	0.847665	0.936284	
PEA15(PEA-15_pS116-R-V)	1.02563928	0.995231297	1.021301	0.356254	0.698143	
PIK3C2A(PI3K-p110-a-R-C)	0.984473161	1.049790429	-1.04632	0.116521	0.472879	
PRKAR1A(PKA-a-R-V)	1.122131477	1.009957801	1.080856	0.195368	0.608647	
PRKCA(PKC-a_pS657-R-C)	1.059049635	1.003271013	1.03942	0.189566	0.598242	
PRKCD(PKC-delta_pS664-R-V)	0.981489808	0.998031928	-1.01153	0.601217	0.829313	
PKM2(PKM2-R-C)	1.087395488	1.032858778	1.038526	0.641991	0.838731	
PLCG2(PLC-gamma2_pY759-R-C)	0.976594447	1.032490358	-1.0395	0.138874	0.527288	
PLK1(PLK1-R-C)	0.99317841	0.981934177	1.007824	0.893536	0.942317	
PMS2(PMS2-R-V)	0.98405524	1.020545909	-1.02562	0.376126	0.714052	
PGR(PR-R-V)	0.987025266	0.99968235	-1.00881	0.76161	0.902787	
AKT1S1(PRAS40_pT246-R-V)	0.991404477	0.985284384	1.004251	0.878537	0.940925	
PREX1(PREX1-R-V)	0.950546276	0.990428938	-1.02803	0.307931	0.685632	
RAB11A(Rab11-R-E)	1.036203979	1.026543533	1.006719	0.817625	0.932783	
RAB25(Rab25-R-V)	0.902723693	0.973633734	-1.05038	0.152003	0.551293	
RAD51(Rad51-R-V)	1.090765431	1.009780413	1.05774	0.085843	0.453477	
RPTOR(Raptor-R-V)	1.041852701	1.038505786	1.002323	0.942064	0.965914	
RBM15(RBM15-R-V)	1.017893166	0.958177902	1.04226	0.13497	0.520599	
RICTOR(Rictor-R-C)	0.946877054	1.001667742	-1.03871	0.409577	0.731378	
RICTOR(Rictor_pT1135-R-V)	0.966053761	0.996562668	-1.02137	0.485696	0.771439	
RIP(RIP-R-C)	0.916735974	1.051365092	-1.09781	0.068194	0.424899	
ROCK1(Rock-1-R-C)	0.959803299	1.000668088	-1.02873	0.476526	0.771439	
RPA2(RPA32_pS4_S8-R-C)	0.967809735	0.94262963	1.017607	0.782012	0.922471	
RPS6KA1(RSK-R-C)	1.021922775	1.053067877	-1.02182	0.429667	0.735275	
RPS6(S6_pS235_S236-R-V)	1.002182231	1.056525977	-1.03839	0.611956	0.829313	
RPS6(S6_pS240_S244-R-V)	1.013970092	0.992494969	1.014997	0.863694	0.940925	
SDHA(SDHA-R-V)	1.091968416	0.979713443	1.080916	0.100919	0.472879	
SHC1(Shc_pY317-R-V)	0.999428732	0.987290965	1.008449	0.708566	0.860908	

PTPN11(SHP-2_pY542-R-C)	0.938300174	0.997824041	-1.04212	0.106862	0.472879	
SLC1A5(SLC1A5-R-C)	1.079686225	1.038819988	1.028731	0.792023	0.927787	
SMAD1(Smad1-R-V)	1.056832678	1.015607027	1.028988	0.282079	0.646652	
SMAD3(Smad3-R-V)	1.018871014	0.978153514	1.028625	0.380167	0.716129	
SRC(Src_pY416-R-V)	0.979432043	1.003462504	-1.0168	0.508035	0.786164	
SRC(Src_pY527-R-V)	0.959998657	1.011411868	-1.03628	0.299254	0.679614	
STAT(Stat_pY694-R-N)	0.988766812	1.055361261	-1.04724	0.15555	0.555862	
STAT3(Stat3-R-C)	0.8504577	1.017609605	-1.12284	0.186502	0.598242	
STAT5A(Stat5a-R-V)	1.042990888	0.951784543	1.065261	0.226569	0.631598	
STMN1(Stathmin-1-R-V)	0.953139659	1.018587868	-1.04641	0.199151	0.612577	
TAZ(TAZ-R-V)	0.974654671	1.040352395	-1.04659	0.066271	0.423789	
TFAM(TFAM-R-V)	0.955765735	0.96097697	-1.00362	0.911294	0.942317	
TFRC(TFRC-R-V)	0.972993844	1.047161538	-1.05275	0.329589	0.695756	
TIGAR(TIGAR-R-V)	0.939045107	1.01014504	-1.05052	0.233925	0.631598	
TRIM25(TRIM25-R-C)	1.027547954	1.16498164	-1.09995	0.352051	0.698143	
TSC1(TSC1-R-C)	0.988617373	1.027950045	-1.02764	0.48622	0.771439	
TTF1(TTF1-R-V)	1.030522562	1.008587881	1.01532	0.555767	0.803878	
TSC2(Tuberin-R-V)	1.00262641	1.009404662	-1.00471	0.882844	0.940925	
TSC2(Tuberin_pT1462-R-V)	1.006593003	1.029327527	-1.01588	0.471142	0.771439	
TUFM(TUFM-R-V)	1.016871798	1.047005518	-1.02111	0.428887	0.735275	
TYRO3(Tyro3-R-V)	1.021050207	1.021202551	-1.00011	0.996052	0.997353	
UBAC1(UBAC1-R-V)	1.017426194	0.965559574	1.036605	0.2786	0.646652	
ULK1(ULK1_pS757-R-C)	0.961140121	1.018301056	-1.04042	0.141722	0.529824	
VASP(VASP-R-V)	1.01464784	0.974167047	1.028457	0.372732	0.713179	
KDR(VEGFR-2-R-V)	1.010889486	0.998452343	1.008658	0.825979	0.933549	
WEE1(Wee1-R-C)	0.972246554	0.981382454	-1.00635	0.801791	0.927787	
WIPI1(WIPI1-R-C)	1.00276468	0.994394131	1.005819	0.84386	0.936284	
WIPI2(WIPI2-R-C)	0.981949502	1.00437769	-1.01567	0.464352	0.771439	
YAP1(YAP-R-E)	1.021591405	1.040565826	-1.01324	0.677597	0.854059	
ZAP70(ZAP-70-R-C)	1.021194615	1.134941437	-1.08203	0.365081	0.709717	

Supplementary Table 4. Nanostring analysis in tumor infiltrating lymphocytes from 344SQP versus 344SQR tumors treated with anti-PD1

Symbol	344SQR+anti-PD1_mean	344SQP+anti-PD1_mean	P.Value	FDR	FCH_ResIdrug/Pardrug
Slc7a11	3.768	7.629	0.000	0.042	-14.534
Ptgdr2	5.313	7.898	0.001	0.163	-6.000
Il1a	7.681	10.111	0.002	0.175	-5.389
Cd53	5.449	7.670	0.003	0.175	-4.659
Clec5a	7.229	9.467	0.004	0.175	-4.717
Irak3	6.096	8.444	0.004	0.175	-5.091
Fpr2	9.639	12.020	0.005	0.175	-5.210
Mapk14	3.722	6.376	0.005	0.175	-6.293
Atf1	4.694	6.973	0.005	0.175	-4.853
Ccr2	6.880	9.398	0.006	0.175	-5.726
Gbp5	7.629	9.971	0.006	0.175	-5.068
Clec7a	7.573	9.665	0.006	0.175	-4.263
Itga4	7.442	9.585	0.006	0.175	-4.416
Gbp2b	9.419	11.580	0.006	0.175	-4.471
Fas	6.241	8.256	0.006	0.175	-4.041
Ifih1	4.520	6.709	0.007	0.181	-4.557
Mefv	8.419	10.501	0.008	0.199	-4.232
Ccl17	8.465	10.402	0.010	0.219	-3.829
Tnf	9.514	11.570	0.010	0.219	-4.158
Ccl24	12.240	14.445	0.010	0.219	-4.611
Il1rl1	8.686	10.660	0.010	0.219	-3.929
Cd200r1	7.375	9.112	0.013	0.233	-3.333
Ccr3	9.148	10.910	0.014	0.233	-3.392
Herc6	6.126	8.136	0.015	0.233	-4.025
Ikzf1	7.603	9.365	0.015	0.233	-3.391
Dpp4	4.546	6.211	0.016	0.233	-3.171
Il1r2	9.821	11.435	0.016	0.233	-3.060
Creb5	5.877	7.568	0.017	0.233	-3.230
Nod2	7.637	9.322	0.018	0.233	-3.214
Il18	7.247	8.932	0.018	0.233	-3.216
Tlr6	7.382	9.291	0.018	0.233	-3.755
Rsad2	7.039	8.979	0.019	0.233	-3.835
Cd33	9.322	10.915	0.019	0.233	-3.016
Cxcl2	8.715	10.383	0.019	0.233	-3.178
Ifit2	4.421	6.153	0.019	0.233	-3.322
Cd274	10.977	12.855	0.020	0.233	-3.677
Sell	9.804	11.465	0.020	0.233	-3.162
Il15ra	4.474	6.066	0.021	0.233	-3.013
Ifi44l	7.032	8.906	0.022	0.233	-3.665
Il13ra1	8.807	10.426	0.022	0.233	-3.072
Nlrp3	10.833	12.615	0.022	0.233	-3.438
Il18rap	7.357	8.956	0.022	0.233	-3.028
H60a	6.423	8.037	0.022	0.233	-3.060
Slamf6	4.805	6.450	0.025	0.248	-3.127
Cd80	9.049	10.580	0.025	0.248	-2.891
Cxcr2	9.533	11.245	0.026	0.253	-3.276
Cd28	5.134	7.292	0.029	0.273	-4.463
Il1rn	13.593	15.220	0.030	0.275	-3.088
Il18r1	6.084	7.493	0.030	0.275	-2.656
Amica1	9.221	10.745	0.031	0.276	-2.876
Il1b	12.553	14.180	0.032	0.282	-3.088
Hif1a	11.203	12.700	0.033	0.283	-2.822
Bst1	9.098	10.605	0.034	0.283	-2.842
Cd86	9.154	10.680	0.035	0.283	-2.881
Cd84	9.425	10.905	0.036	0.283	-2.789
Chil3	12.077	13.860	0.036	0.283	-3.442
Irgm2	6.745	8.159	0.037	0.283	-2.663
Il7r	8.805	10.276	0.037	0.283	-2.771
Akt3	7.020	8.422	0.038	0.283	-2.642
Csf2rb	12.097	13.520	0.038	0.283	-2.682
Rel	8.366	9.823	0.038	0.283	-2.746
Cxcl10	9.043	10.670	0.039	0.285	-3.089
Il12rb2	6.133	7.479	0.043	0.302	-2.541
Lif	5.718	7.115	0.045	0.305	-2.634
Mertk	7.493	8.854	0.046	0.305	-2.568
Cxcl11	8.271	9.650	0.046	0.305	-2.599
Lyn	11.233	12.615	0.047	0.305	-2.606
Cybb	10.947	12.325	0.047	0.305	-2.600
Tlr1	6.618	8.170	0.048	0.305	-2.932

Ccl6	13.037	14.410	0.050	0.305	-2.591
Hck	8.719	10.039	0.050	0.305	-2.495

Supplementary Table 4. Nanostring analysis in tumor infiltrating lymphocytes from 344SQP versus 344SQR tumors treated with anti-PD1

Symbol	344SQR+anti-PD1_mean	344SQP+anti-PD1_mean	P.Value	FDR	FCH_ResIdrug/Pardrug
Muc1	8.594	5.466	0.0004211	0.1072	8.744
Erbb2	7.181	3.415	0.0021734	0.1752	13.604
Colec12	7.780	5.296	0.0022187	0.1752	5.593
Il22ra1	8.133	6.450	0.014645	0.2327	3.210
Ccl8	11.950	10.319	0.01499	0.2327	3.098
Ctsg	5.503	3.586	0.0156567	0.2327	3.777
Pdgfc	9.074	7.426	0.0262608	0.2528	3.135
Itgb4	10.107	8.741	0.0344695	0.2826	2.579
Il3ra	7.010	5.512	0.0418838	0.3003	2.825
Blnk	7.099	5.671	0.0432657	0.3017	2.692
Ccr8	6.077	4.757	0.0483025	0.305	2.496

Supplementary Table 5a. TCGA LUAD cohort Univariate analysis. The p-values are given by the Wald test. All statitical test were two-sided.

variable	HR	lower .95	upper .95	p-value	no pt.
Age (continuous)	0.999	0.98	1.01	0.88914	422
Smoking (Ever vs Never)	0.87	0.55	1.37	0.55186	419
Gender (Male vs Female)	0.95	0.69	1.31	0.77607	431
Stage (III-IV vs I-II)	2.64	1.88	3.69	0.00000002	423
BMP7 (FPKM)(continuous)	1.19	1.02	1.39	0.0247	431

Supplementary Table 5b. TCGA LUAD cohort Multivariate analysis Stage & BMP7 (n=423). The p-values are given by the Wald test. All statitical test were two-sided.

variable	HR	lower .95	upper .95	p-value
Stage (III-IV vs I-II)	2.57	1.83	3.6	0.00000005
BMP7 (FPKM)(continuous)	1.14	0.98	1.32	0.09778

Supplementary Table 5c. Der SD et al, LUAD cohort Univariate analysis (n=127).The p-values are given by the Wald test. All statitical test were two-sided.

variable	HR	lower .95	upper .95	p-value
Gender	1.41	0.81	2.46	0.22804
Stage (II vs I)	2.44	1.38	4.32	0.0021
Age (continuous)	1.02	0.99	1.05	0.19195
Smoking (Ever vs Never)	1.68	0.75	3.77	0.20721
BMP7 (209590_at) (continuous)	1.17	0.91	1.52	0.22187
BMP7 (209591_s_at) (continuous)	1.16	1	1.35	0.04274
BMP7 (211259_s_at) (continuous)	1.4	0.89	2.2	0.14535
BMP7 (211260_at) (continuous)	5.4	1.7	17.15	0.00426
BMP7 (233583_at) (continuous)	1.12	0.29	4.29	0.86452

Supplementary Table 5d. Der SD et al, LUAD cohort Multivariate analysis Stage & BMP7 (209591_s_at) (n=127).The p-values are given by the Wald test. All statitical test were two-sided.

variable	HR	lower .95	upper .95	p-value
Stage (II vs I)	2.4	1.36	4.24	0.00252
BMP7 (209591_s_at) (continuous)	1.15	0.998	1.32	0.05349

Supplementary Table 5e. Der SD et al, LUAD cohort Multivariate analysis Stage & BMP7 (211260_at) (n=127). The p-values are given by the Wald test. All statitical test were two-sided.

variable	HR	lower .95	upper .95	p-value
Stage (II vs I)	2.42	1.37	4.29	0.00234
BMP7 (211260_at) (continuous)	5.43	1.68	17.53	0.00463

Supplementary Table 6. List of Primers used in quatitative PCR (qPCR) analysis

BMP7 Fw	CAGCCAGAATCGCTCCAAGA
BMP7 Rv	GCAATGATCCAGTCCTGCCA
MAPK14 Fw	ATCATTCACGCCAAAAGGAC
MAPK14 Rv	AGCTTCTGGCACTTCACGAT
IL1A Fw	CGCTTGAGTCGGCAAAGAAAT
IL1A Rv	TGGCAGAACTGTAGTCTTCGT
IL1B Fw	GTGGCAGCTACCTGTGTCTT
IL1B Rv	AATGGGAACGTCACACACCA
TNF Fw	CGGGCAGGTCTACTTTGGAG
TNF Rv	AAGGATACAGACTGGGGGCT
CCL5_Fw	CTCACCATATGGCTCGGACA
CCL5_Rv	CGACTGCAAGATTGGAGCAC
IFNG_Fw	CGGCACAGTCATTGAAAGCC
IFNG_Rv	TGTCACCATCCTTTTGCCAGT
IL2_Fw	AGGAACCTGAAACTCCCCAG
IL2_Rv	CTTTCAATTCTGTGGCCTGCTT
ACTB_Fw	GTGACGTTGACATCCGTAAAGA
ACTB_Rv	GCCGGACTCATCGTACTCC
CD45_Fw	CTTTGCTTATGTGGCGTGTGT
CD45_Rv	TTATCCCCTTCTGATGCGCC

Supplementary Methods

Bio-Plex mouse cytokine 23-plex assay

Serum samples were collected from mice bearing 344SQP or 344SQR tumors treated with anti-PD1 twice per week for a total of 4 doses. At 24 hours after the last anti-PD1 treatment, whole blood samples were collected by cardiac puncture and centrifuged at 1,000×*g* for 10 min, and serum was collected and kept in –80°C until analysis. Serum was diluted 1:4 with diluent solutions from the BioPlex Multiplex assay (BioRad). Twenty-three cytokines, including IL1A, IL1B, TNF, CCL5, IFNG, and IL2 were measured by ELISA according to the manufacturer's protocol (Biorad, Catalog #m60009rdpd).

Transfection siRNA

siRNA targeting MAPK14 (Life Technologies, Cat #4390771) and its respective negative-control negative control (Life Technologies, Cat #4390843) were reverse-transfected into RAW 264.7 macrophages with Lipofectamine 2000 (Life Technologies) to a final concentration of 100 nm/L.

Co-culture experiments and treatments

Viable cells were counted with a hemocytometer (0.4% Trypan blue solution) and diluted to 40,000 cells per well in 24-wells plates. 344SQR cells were seeded at the top inserts (24-mm Transwell with 0.4-µm pore polycarbonate membrane insert, Sigma-Aldrich), and RAW 264.7, or peritoneal macrophages were seeded at the bottom of the transwell system. Cells were then cultured in complete medium (RPMI-1640 supplemented with 100 units/mL penicillin, 100 µg/mL streptomycin, and 10% heat-inactivated fetal bovine serum) and incubated at 37°C in 5% CO_2 for 24 or 48 hours, after which cells were treated with K02288 (6.4 nM) (catalog # S7359, Selleck

Chemicals) for 24 or 48 hours. RNA was then isolated from RAW 264.7 cells or peritoneal macrophages and analyzed for MAPK14, IL1A, IL1B, TNF and CCL5 expression with qPCR.

In vivo study in triple negative breast cancer model 4T1

All mouse studies were approved by the Institutional Animal Care and Use Committee (IACUC) of The University of Texas MD Anderson Cancer Center before their initiation; animal care was provided according to IACUC standards, and all mice had been bred and were maintained in our own specific pathogen-free mouse colony. For tumor growth and survival studies, primary tumors were established by subcutaneous injection of 4T1 cells (5×10^4 in 100 μL of sterile PBS) into the leg of syngeneic BALB/c mice (female, 12–16 weeks old). The mice were then given intraperitoneal injections of anti-PD-1 or control IgG antibodies (10 mg/kg) (Bio X cell), starting on day 4 after tumor cell inoculation and continuing twice per week for a total of 6 doses. Tumors were measured with calipers three times per week and recorded as tumor volume (in mm^3) = $width^2 \times length / 2$. A Two-way RM ANOVA analysis of variance was done to compare tumor growth curves between groups. Mouse survival rates were analyzed by using the Kaplan–Meier method and compared with log-rank tests.

Depletion in vivo studies

All mouse studies were approved by the Institutional Animal Care and Use Committee (IACUC) of The University of Texas MD Anderson Cancer Center before their initiation; animal care was provided according to IACUC standards, and all mice had been bred and were maintained in our own specific pathogen-free mouse colony. For tumor growth and survival studies, primary tumors were established by subcutaneous injection of 344SQR ctrl or 344SQR shBMP7 cells (0.25 × 106 in 100 μL of sterile PBS) into the leg of syngeneic 129Sv/Ev mice (female, 12–16 weeks old). The

mice were then given intraperitoneal injections of anti-PD-1 (10 mg/kg), anti-CD4 (15 mg/kg), anti-F4/80 (5 mg/kg) or control IgG antibodies (10 mg/kg) (Bio X cell), starting on day 4 after tumor cell inoculation and continuing twice per week for a total of 4 doses. Tumors were measured with calipers three times per week and recorded as tumor volume (in mm3) = width2 × length / 2. Tumor growth curves were compared with Two-way RM ANOVA.

TCGA data

Analysis of TCGA data was performed in R (version 3.5.3; http:///www.r-project.org/) and the statistical significance was defined as a $P < 0.05$. Clinical information for patients with lung adenocarcinoma was retrieved from the article "An Integrated TCGA Pan-Cancer Clinical Data Resource to Drive High-Quality Survival Outcome Analytics", Cell. Volume 173 (https://www.sciencedirect.com/science/article/pii/S0092867418302290?via%3Dihub), but smoking status. The information regarding smoking status of these was retrieved from cBioPortal for Cancer Genomics (http://www.cbioportal.org/)(Ref: Cerami E et al, The cBio Cancer Genomics Portal: An Open Platform for Exploring Multidimensional Cancer Genomics Data, Cancer Discov. 2012 May;2(5):401-4. doi: 10.1158/2159-8290.CD-12-0095). Gene expression for BMP7 was downloaded as fragments per kilobase millions (FPKM) quantification mRNA-seq data from the Genomic Data Commons Data Portal (https://portal.gdc.cancer.gov/). The relationship between overall survival and covariates (mRNA expression levels and various patient data information) was examined using a Cox proportional hazard model (univariate followed by multivariate for the resulting significant variables). Results are presented in the tables bellow.

Der SD et al, LUAD cohort

Survival analyses were performed in R (version 3.5.3; http:///www.r-project.org) and the statistical significance was defined as a $P < 0.05$. We retrieved from GEO repository microarray expression

(normalized log2) data BMP7 and clinical information for 181 Stage I and II NSCLC cases (GSE9893, ref: Der SD et al, Validation of a histology-independent prognostic gene signature for early-stage, non-small-cell lung cancer including stage IA patients, J Thorac Oncol. 2014 Jan;9(1):59-64. doi: 10.1097/JTO.0000000000000042, PMID: 24305008). Univariate Cox proportional hazards model was fitted to evaluate the association between overall survival and covariates including BMP7 expression and available clinical variables. The variables significant in the univariate analysis were further examined in a multivariate analysis. We found BMP7 (21160_at) to be an independent marker of poor overall survival. To visualize the result, we used the log-rank test to find the point (cut-off) with the most significant (lowest p-value) split in high vs low mRNA level groups. The cut-off is 0.65. Patients at risk in low and high mRNA groups at different time points are presented at the bottom of the graph, and the medium survival in each group are presented in brackets.

Correlation Analysis

The Spearman's rank-order correlation test was applied to measure the strength of association between gene expression levels in tumor samples.
