# Supplementary material for: Bone morphogenetic protein 7 promotes resistance to immunotherapy
Source: Nat Commun. 2020 Sep 24;11:4840. doi: 10.1038/s41467-020-18617-z (PMC7519103; doi:10.1038/s41467-020-18617-z)
Supplement: Supplementary file 3 — Description of Additional Supplementary Files [file 41467_2020_18617_MOESM3_ESM.pdf]

Description of Additional Supplementary Files

File Name: Supplementary Data 1
Description: Reduced-representation bisulfite sequencing (RRBS) in 344SQP versus 344SQR tumors treated with anti-PD1

File Name: Supplementary Data 2
Description: Raw data for Figure 3
