# Supplementary material for: Bone morphogenetic protein 7 promotes resistance to immunotherapy
Source: Nat Commun. 2020 Sep 24;11:4840. doi: 10.1038/s41467-020-18617-z (PMC7519103; doi:10.1038/s41467-020-18617-z)
Supplement: Supplementary file 4 — Supplementary Data 1 [file 41467_2020_18617_MOESM4_ESM.pdf]

Supplementary data 1. Reduced-representation bisulfite sequencing in 344SQP versus 344SQR tumors treated with anti-PD1. For Methylation Calling analysis, the methylation percentages for CpG sites were calculated by the bismark_methylation_extractor script from Bismark and an in-house Perl script. The differential methylation on CpG sites was statistically assessed by R/Bioconductor package methylKit (version 0.9.5). The CpG sites with read coverage ≥ 20 in all the samples were qualified for the test. The significance of differential methylation on gene level was calculated using Stouffer's zscore method by combining all the qualified CpG sites inside each gene's promoter region (defined as -1000bp to +500 of TSS), and was corrected to FDR by Benjamini & Hochberg (BH) method.

gene	num_sites_in_promoter	meth_control	meth_treatment	meth_diff	pvalue	FDR
Ppm1h	81	10.10873295	2.179408451	-7.9293245	0	0
Cacna2d1	100	39.76517923	20.45530044	-19.30987878	0	0
Gm23450	85	21.15775033	7.867240004	-13.29051032	0	0
Kif21a	67	23.34758317	2.213543368	-21.1340398	0	0
Adcy3	66	29.1967836	12.15585854	-17.04092506	0	0
Lmo1	65	32.23789966	15.70531019	-16.53258947	0	0
Ncam2	81	12.20275002	3.563276109	-8.639473907	0	0
Slc2a13	57	28.75605774	5.712374872	-23.04368286	0	0
Irx2	79	53.80527918	34.96523774	-18.84004144	0	0
Ptprm	111	7.163029664	1.43335333	-5.729676334	0	0
Atp9a	118	11.22947198	2.029095799	-9.200376185	0	0
Mir8104	111	9.612229918	2.380949527	-7.231280391	0	0
Fam196a	72	37.94969671	14.79962411	-23.1500726	0	0
Gm3230	82	10.12961789	1.119227518	-9.010390373	0	0
Kazn	96	24.34131967	8.935775418	-15.40554425	0	0
Tmem200b	195	20.12096797	42.21797333	22.09700536	0	0
Raver2	85	16.73783178	37.32816307	20.59033128	0	0
Igf2bp1	80	19.40391987	5.67095636	-13.73296351	0	0
330182L06R	64	23.19194654	6.763023393	-16.42892315	0	0
Bmp7	61	30.088685	6.517012078	-23.57167292	0	0
Shank3	135	9.549899343	2.603902499	-6.945996844	1.25E-301	8.75E-299
Med12l	155	25.24997482	36.11538724	10.86541243	3.74E-300	2.50E-297
Fam69c	74	20.71306285	9.275775339	-11.43728751	5.66E-300	3.61E-297
Mlf1	36	21.86053942	5.877691088	-15.98284833	4.08E-299	2.50E-296
Plcl2	123	7.013507747	1.330238083	-5.683269664	1.23E-296	7.23E-294
Fam65b	62	17.84912364	5.579842708	-12.26928093	5.99E-288	3.38E-285
Adcy1	73	28.15648465	12.78769409	-15.36879056	3.62E-286	1.97E-283

Mir6973b	65	16.22101059	5.730171763	-10.49083883	1.05E-285	5.52E-283
Igf2	109	17.98229053	8.647108387	-9.335182141	3.45E-285	1.75E-282
Slc35f1	52	36.96327435	17.74789464	-19.21537971	3.31E-280	1.62E-277
Ppp1r9a	82	29.94690503	14.76951991	-15.17738512	1.28E-272	6.05E-270
Rims2	166	11.41744174	5.475814002	-5.941627737	5.07E-263	2.33E-260
Rbm20	93	26.90174192	41.63870623	14.73696431	1.59E-261	7.08E-259
Limch1	51	24.05636212	9.003431048	-15.05293108	1.15E-259	4.96E-257
Igf2os	87	15.7863015	6.203250158	-9.583051341	1.21E-258	5.05E-256
Nim1k	37	19.81855412	2.973043956	-16.84551017	1.61E-257	6.57E-255
Zfp518b	77	31.03618277	47.4329252	16.39674243	1.71E-256	6.78E-254
Abr	58	38.8192606	21.47154068	-17.34771992	2.65E-253	1.02E-250
Aff3	157	22.80399439	15.58865001	-7.215344386	2.29E-252	8.63E-250
Aldh1a2	128	27.08543708	40.199403	13.11396592	2.18E-250	8.01E-248
Kcnn2	90	39.3422319	25.69279168	-13.64944022	3.45E-245	1.23E-242
P2rx5	40	13.570189	2.172622642	-11.39756636	2.83E-239	9.89E-237
Pmaip1	46	14.50173894	2.231462054	-12.27027689	2.72E-231	9.29E-229
B4galt4	62	14.75679194	3.739111454	-11.01768049	1.04E-227	3.46E-225
Satb1	150	27.92422694	19.53431857	-8.389908371	2.05E-223	6.68E-221
Fbxl7	105	48.90128127	37.82982639	-11.07145489	1.45E-222	4.64E-220
Grb10	114	48.60047164	63.90311495	15.30264331	6.97E-214	2.18E-211
Vegfc	54	31.27397723	45.48578298	14.21180574	1.77E-213	5.40E-211
Npas2	90	14.63681798	6.76421778	-7.8726002	6.68E-210	2.00E-207
B4galnt4	85	12.42873424	4.393135777	-8.035598459	5.56E-209	1.63E-206
Fam49a	49	10.19086194	1.455984581	-8.73487736	6.17E-207	1.77E-204
AW046200	45	15.94725266	4.350976195	-11.59627646	4.64E-206	1.31E-203
Dmrta1	37	20.47618842	44.25871918	23.78253076	2.08E-204	5.76E-202
Dact1	89	14.87951013	7.857914744	-7.02159539	1.62E-202	4.39E-200
Ptprg	88	7.441147208	1.504240387	-5.936906821	5.46E-202	1.46E-199
Kcnab2	56	38.79783188	23.05141953	-15.74641235	5.12E-201	1.34E-198
Map9	60	8.458541979	20.31083104	11.85228906	1.22E-195	3.15E-193
Nova1	124	20.86261419	14.37498032	-6.487633872	1.05E-194	2.67E-192
Trim47	73	8.182708103	1.77937163	-6.403336472	1.50E-194	3.74E-192
Kctd1	89	10.73363316	3.618905754	-7.114727401	2.80E-194	6.84E-192
Sema4f	43	15.94934672	5.11070337	-10.83864335	2.97E-194	7.14E-192

Krba1	35	12.60174732	2.352763736	-10.24898358	1.59E-193	3.77E-191
Xylt1	87	34.34579734	23.53621831	-10.80957903	1.87E-188	4.36E-186
330050F15F	69	28.10644738	18.97961639	-9.126830995	9.68E-188	2.22E-185
Mapk8ip1	92	53.91094751	66.27500363	12.36405612	1.00E-186	2.27E-184
Gsx1	74	21.43308943	11.83297616	-9.600113268	8.63E-185	1.92E-182
Dock3	83	11.21935228	22.62092613	11.40157385	1.20E-183	2.64E-181
Slitrk5	47	29.89813925	47.05981622	17.16167696	7.66E-183	1.65E-180
330082K12F	21	13.95503669	1.394569669	-12.56046702	2.52E-182	5.35E-180
Kcnk2	56	39.0416805	24.07784416	-14.96383634	8.31E-176	1.74E-173
Sfi1	30	84.71670077	80.21566903	-4.501031748	6.25E-175	1.29E-172
Rax	32	30.3689488	54.78914016	24.42019137	5.93E-174	1.21E-171
Rprm	42	30.64532117	13.92850257	-16.7168186	6.74E-174	1.36E-171
Galnt9	73	18.6197997	33.63952003	15.01972033	1.74E-172	3.44E-170
St8sia5	81	14.78098243	28.03493637	13.25395394	6.44E-172	1.26E-169
Nlrx1	56	9.573064821	2.263168243	-7.309896578	4.00E-171	7.73E-169
930444P10F	45	18.91796846	7.788278499	-11.12968997	6.37E-168	1.21E-165
Lppr5	58	47.2486515	31.41783384	-15.83081766	2.82E-167	5.31E-165
Ttyh3	68	30.41184718	43.99018122	13.57833404	7.64E-167	1.42E-164
A4galt	68	45.17666974	30.96999658	-14.20667316	9.15E-167	1.68E-164
Gas6	36	12.90879114	4.005994758	-8.902796377	1.48E-164	2.67E-162
Zfp641	36	24.67287298	11.75666373	-12.91620925	3.02E-160	5.41E-158
H2-Q6	13	21.80383229	6.984766812	-14.81906548	1.03E-159	1.82E-157
Dnajc6	93	32.62515894	46.46469177	13.83953282	4.83E-159	8.44E-157
Syde2	161	7.112759622	13.11922778	6.006468157	1.06E-157	1.83E-155
Rap1gap	62	18.34414196	9.25812301	-9.086018952	4.28E-157	7.30E-155
Mmp2	41	37.37962315	23.1472109	-14.23241225	7.44E-153	1.26E-150
Lifr	80	5.181614328	1.143972243	-4.037642085	1.20E-152	2.01E-150
Unc5a	116	21.12656396	15.20872779	-5.917836168	2.62E-151	4.31E-149
Bmp8b	61	52.85390313	67.53208637	14.67818324	1.02E-150	1.66E-148
Kcnk4	25	44.9366223	68.11734957	23.18072727	1.47E-150	2.37E-148
Foxe1	58	53.65274906	68.94672712	15.29397806	1.32E-149	2.10E-147
Prkd1	70	9.880890046	3.038855559	-6.842034487	1.97E-149	3.10E-147
Cpeb1	123	14.49105914	22.13561828	7.644559143	2.14E-149	3.35E-147
Nlgn3	15	46.13601234	17.40864037	-28.72737197	7.43E-148	1.15E-145

Lin7a	21	27.5375621	10.35673952	-17.18082257	9.98E-148	1.53E-145
Otud1	118	23.82431804	19.28314521	-4.541172833	2.29E-146	3.46E-144
Pclo	76	25.26600892	15.929757	-9.336251925	1.70E-145	2.54E-143
Jazf1	137	11.99586659	7.388296729	-4.607569858	8.03E-145	1.19E-142
Id4	135	49.95326124	59.85735372	9.904092477	8.94E-143	1.31E-140
Sdk1	182	46.10134621	54.2868068	8.185460586	3.01E-142	4.38E-140
Ror2	88	35.64283839	46.88991892	11.24708053	1.61E-141	2.32E-139
Slc34a2	27	17.86186925	5.856039374	-12.00582988	5.01E-140	7.14E-138
Zfp704	73	6.51897495	2.446674465	-4.072300485	7.49E-140	1.06E-137
Zbtb10	114	10.18212934	5.561998972	-4.620130372	2.03E-139	2.84E-137
Tub	79	19.32827542	11.24755832	-8.080717101	2.09E-139	2.89E-137
Palm3	29	46.29095632	68.71935749	22.42840117	2.55E-139	3.50E-137
Dclk1	17	23.5221403	5.524629997	-17.9975103	5.63E-139	7.65E-137
Npr3	92	19.9296407	9.530779568	-10.39886113	1.08E-138	1.45E-136
Prkg1	67	30.65880134	20.90998523	-9.748816108	1.41E-138	1.88E-136
Tlr2	20	21.49320065	5.409083786	-16.08411687	2.10E-138	2.77E-136
Galnt7	68	9.010526554	3.975914327	-5.034612226	1.19E-137	1.56E-135
Dsc2	25	28.04893222	9.528433801	-18.52049842	1.60E-137	2.08E-135
Zyg11a	62	34.47752949	50.43059391	15.95306442	2.17E-136	2.79E-134
Pou3f1	122	53.62082717	64.19914107	10.5783139	7.73E-136	9.86E-134
Prdm11	120	7.039825027	1.182479086	-5.857345941	1.55E-135	1.96E-133
Osbpl1a	79	21.88466151	17.28701313	-4.597648389	4.36E-135	5.47E-133
Nphp4	48	39.49030772	25.40335656	-14.08695116	1.95E-134	2.43E-132
Slc5a5	35	48.52985073	65.73629133	17.20644059	5.18E-133	6.39E-131
Zic3	43	36.86236006	18.22328267	-18.63907739	2.59E-132	3.17E-130
Scn8a	142	26.43892893	33.52047526	7.081546334	1.83E-131	2.22E-129
Nptx2	109	51.23053992	62.05743121	10.82689129	1.97E-131	2.36E-129
Dlx3	25	17.49819777	2.055967329	-15.44223045	2.39E-131	2.85E-129
Ugt8a	101	49.00752509	57.57495415	8.56742906	1.05E-130	1.24E-128
Hotairm1	25	18.69559045	6.806861757	-11.88872869	1.70E-130	1.99E-128
Fam189a1	145	42.14248744	34.22924498	-7.913242462	2.70E-130	3.15E-128
Grip1	108	13.70051323	8.376476565	-5.324036668	4.03E-129	4.66E-127
Cecr6	91	8.030178969	3.266279006	-4.763899963	2.89E-128	3.31E-126
Maneal	113	25.03953794	34.41476755	9.37522961	5.34E-128	6.07E-126

Mtmr7	51	31.82012809	45.97845543	14.15832734	7.34E-128	8.29E-126
Reln	134	57.69096534	66.44463439	8.75366905	2.59E-126	2.90E-124
Rab42	17	3.617393581	26.06181841	22.44442483	1.33E-125	1.48E-123
Cacnb4	47	20.00760004	32.40712151	12.39952146	2.47E-125	2.73E-123
Ccser1	71	30.82871045	20.86351554	-9.965194908	7.38E-124	8.08E-122
'30090H04F	26	15.89424972	3.486271907	-12.40797781	2.13E-123	2.31E-121
Mgll	79	19.87840928	32.01972369	12.14131442	2.57E-123	2.78E-121
H2-Q9	15	20.22721489	4.050417837	-16.17679706	3.73E-123	4.00E-121
Cpne8	39	7.050915815	2.107292846	-4.943622969	1.81E-122	1.93E-120
Msx3	36	19.57441732	7.458063796	-12.11635353	2.08E-122	2.20E-120
Chrm3	38	23.70237	12.7208403	-10.98152969	1.30E-120	1.37E-118
Sall3	175	42.60436309	51.64630684	9.041943751	5.39E-120	5.61E-118
Mir1247	87	22.87108007	35.49581957	12.6247395	1.02E-119	1.06E-117
Nkain3	45	24.9102855	15.44920712	-9.461078379	1.07E-118	1.10E-116
Foxe3	68	25.32949975	15.61533844	-9.714161312	1.41E-117	1.43E-115
B3gat2	57	23.3143972	15.08879954	-8.225597659	2.39E-116	2.42E-114
Ttc22	27	33.11685263	14.66938383	-18.4474688	7.60E-116	7.64E-114
Gkap1	172	3.96045441	1.08442586	-2.87602855	1.11E-114	1.11E-112
Adra2a	62	7.173551647	14.47582214	7.30227049	2.97E-114	2.95E-112
Sfrp1	64	32.22959051	45.62765077	13.39806026	6.13E-114	6.04E-112
Ngef	109	6.931408637	3.075357967	-3.85605067	1.46E-113	1.43E-111
Arhgef26	51	6.619321398	1.669774113	-4.949547285	2.02E-113	1.96E-111
Mall	15	18.48878145	2.048136078	-16.44064537	2.06E-113	1.98E-111
Tnfrsf19	28	19.1669753	7.492268297	-11.674707	3.57E-113	3.42E-111
Meis2	52	7.244895878	1.293899443	-5.950996434	6.11E-113	5.82E-111
Camk1d	92	11.21382646	5.933563101	-5.280263356	8.22E-113	7.78E-111
Cacna2d3	113	18.10813686	11.59614438	-6.511992479	7.92E-112	7.45E-110
Ccna1	30	23.74727892	9.851048183	-13.89623074	8.31E-112	7.77E-110
Rrad	12	29.76901148	10.37618423	-19.39282726	2.59E-111	2.41E-109
Bean1	59	6.440664284	1.531258383	-4.909405901	2.67E-111	2.46E-109
Ampd3	18	45.34576372	25.2321049	-20.11365881	4.31E-111	3.95E-109
Fgf2	66	8.546977763	4.136540904	-4.410436859	7.69E-111	7.01E-109
Eya2	80	11.779166	19.58757628	7.808410284	6.70E-108	6.07E-106
Htr1d	89	9.184359306	4.977515403	-4.206843904	2.29E-107	2.06E-105

Lypd6b	48	17.48424352	8.242635956	-9.241607563	3.07E-107	2.74E-105
Rac3	89	11.53279052	4.619888542	-6.912901978	3.80E-107	3.38E-105
Fam13c	26	19.19579942	7.437005352	-11.75879407	1.37E-106	1.21E-104
Igsf9b	26	17.26017387	4.55119745	-12.70897642	3.53E-106	3.10E-104
Nav1	16	20.85673495	47.31106702	26.45433206	3.76E-106	3.29E-104
Hoxa4	92	46.51864611	56.63729892	10.11865281	6.31E-106	5.47E-104
Abca14	13	27.08541768	8.909865564	-18.17555211	1.05E-105	9.08E-104
Afap1l2	47	8.841743588	2.715645753	-6.126097835	1.54E-105	1.32E-103
Lmx1b	121	38.41060105	46.93028706	8.519686012	4.48E-105	3.83E-103
Adcy5	162	17.39503058	24.48189046	7.086859874	7.71E-105	6.54E-103
Dab1	68	48.67028042	60.7161277	12.04584729	1.87E-104	1.57E-102
Fam83f	38	52.96191578	38.13619373	-14.82572205	1.55E-103	1.30E-101
Mark1	174	13.30272603	10.4600144	-2.842711631	1.62E-103	1.35E-101
Celsr3	59	22.61273472	33.97081679	11.35808206	2.07E-103	1.71E-101
Trhde	113	52.77299474	61.23384077	8.460846035	3.57E-103	2.94E-101
Tbc1d30	54	15.23308115	8.283285678	-6.949795467	4.57E-103	3.75E-101
Gsc	52	32.07095484	22.14572346	-9.925231377	2.35E-102	1.92E-100
Gas1	60	48.73513047	59.69066402	10.95553355	4.39E-102	3.56E-100
Ctnnd2	127	25.78583522	19.8666499	-5.919185326	6.67E-101	5.38E-99
Pde8b	86	18.59876014	12.13366589	-6.465094248	1.27E-100	1.02E-98
Nr2f1	70	5.586469117	1.56613982	-4.020329297	1.33E-100	1.06E-98
Kcnj8	23	22.10290683	8.383595298	-13.71931153	5.38E-100	4.26E-98
700112E06R	28	11.41196148	2.819573584	-8.592387892	8.06E-100	6.36E-98
Ascl5	32	11.73421705	3.463333222	-8.270883831	1.36E-99	1.06E-97
Lbx1	94	36.85797116	45.56032159	8.702350437	1.99E-99	1.55E-97
Slc18a2	25	28.44613575	15.41769147	-13.02844428	7.72E-98	6.00E-96
700086L19R	28	16.34593855	9.46576691	-6.880171642	2.61E-97	2.02E-95
Fam109b	23	18.85118139	6.822573423	-12.02860797	3.31E-97	2.54E-95
Obsl1	53	14.9397333	25.50195557	10.56222227	1.15E-96	8.81E-95
Zdbf2	127	56.28908782	64.54665414	8.257566316	1.40E-96	1.07E-94
Ptprz1	33	26.06105914	14.7330573	-11.32800185	1.70E-96	1.28E-94
Dio3	80	24.92920057	37.80756638	12.87836581	1.01E-95	7.63E-94
Hs3st2	80	56.97115364	66.2031502	9.231996567	1.04E-95	7.77E-94
Kctd17	29	10.07436857	2.469038292	-7.605330278	1.81E-95	1.34E-93

Hoxa1	12	15.3309757	3.347437119	-11.98353858	2.05E-95	1.52E-93
Ust	37	10.78574037	3.677529535	-7.108210836	8.74E-95	6.44E-93
Plekhg1	26	18.12151049	3.263651582	-14.8578589	1.04E-94	7.65E-93
Hs6st3	159	51.90498547	58.46473645	6.559750984	2.67E-94	1.95E-92
Fgf8	61	26.87046166	37.20179732	10.33133566	2.88E-94	2.09E-92
Ptprb	17	22.80764632	7.650695434	-15.15695089	7.72E-94	5.58E-92
Syngr3	28	11.29741201	3.645925691	-7.651486317	1.37E-93	9.83E-92
130021I20R	108	39.22535002	47.84387218	8.61852216	1.98E-93	1.42E-91
Elavl2	132	37.45201212	46.5346414	9.082629283	2.34E-93	1.67E-91
Barhl2	116	52.68640486	60.64977629	7.963371428	2.45E-93	1.73E-91
Lmx1a	63	26.73606235	19.49072117	-7.245341181	1.35E-92	9.56E-91
Hey2	40	7.215796439	20.13520921	12.91941277	4.68E-92	3.29E-90
Fam19a5	363	64.80597935	69.11164366	4.305664302	5.57E-91	3.89E-89
Gstp1	13	11.42801278	1.984601121	-9.443411663	1.47E-90	1.02E-88
Hoxd8	144	43.35788807	50.75130958	7.393421507	3.00E-90	2.07E-88
Dscam	104	18.85778189	12.47877321	-6.379008684	7.27E-90	5.01E-88
Smoc2	53	19.22931594	11.01557075	-8.213745191	1.48E-89	1.02E-87
Syn2	69	20.47178756	30.61771643	10.14592888	1.53E-89	1.05E-87
Gm11837	99	19.16133345	14.24859108	-4.912742377	1.90E-89	1.29E-87
Agtr1b	8	45.01523817	20.18630548	-24.82893269	2.01E-89	1.36E-87
Runx1t1	126	26.63027159	21.02274958	-5.607522011	3.91E-89	2.63E-87
Gm11549	40	25.90662972	36.70198989	10.79536017	4.34E-89	2.91E-87
730020E08R	91	28.67703339	21.23105554	-7.445977849	6.86E-89	4.58E-87
Foxi2	31	38.97071471	26.32659619	-12.64411852	2.28E-88	1.52E-86
Mal2	35	8.405938852	2.281553358	-6.124385494	4.12E-88	2.72E-86
Irf5	117	7.231350988	3.695816358	-3.53553463	9.97E-88	6.56E-86
B3glct	46	1.225196717	4.527199726	3.302003009	1.91E-87	1.25E-85
Ntf3	21	29.7220543	14.72435295	-14.99770136	3.58E-87	2.33E-85
Pik3r5	22	12.2319647	2.023385072	-10.20857963	3.70E-87	2.41E-85
Elmod1	26	22.65728953	40.23650996	17.57922043	7.08E-86	4.57E-84
Chrnb2	26	22.70734995	12.08637585	-10.6209741	3.39E-85	2.18E-83
AI839979	10	17.24209257	3.454738593	-13.78735398	7.31E-85	4.69E-83
Dio3os	23	22.27115758	38.41309134	16.14193376	8.73E-85	5.57E-83
Rasl11a	55	7.909498517	2.422331468	-5.487167049	2.34E-84	1.49E-82

Hoxc8	35	43.19239511	28.941502	-14.25089311	6.30E-84	3.99E-82
Ppp1r36	30	34.27441427	48.57838954	14.30397527	6.66E-84	4.19E-82
Ttpa	45	21.71499666	13.82530111	-7.889695546	2.90E-83	1.82E-81
Prkg2	71	11.15131855	4.636920796	-6.514397751	6.81E-83	4.25E-81
Gabbr2	124	27.2367571	21.94579384	-5.290963265	1.57E-82	9.76E-81
30059O14	70	18.80239289	11.81699636	-6.985396536	9.37E-82	5.80E-80
Gli3	128	8.126366285	4.670284307	-3.456081978	1.23E-81	7.60E-80
Ptpn20	16	80.56214658	60.76905295	-19.79309363	1.73E-81	1.06E-79
Ttc34	74	26.27280554	36.56951627	10.29671073	2.38E-81	1.46E-79
Prok2	33	24.53992425	14.58448273	-9.955441521	1.28E-80	7.77E-79
Ncmap	30	14.9716586	7.246494264	-7.725164337	1.49E-80	9.03E-79
Neil2	14	21.15631975	41.54613165	20.3898119	1.61E-80	9.72E-79
Zic5	13	11.50078596	1.170029856	-10.3307561	1.67E-80	1.00E-78
Chpt1	63	6.480319196	13.14809963	6.667780432	2.45E-80	1.47E-78
Cecr2	172	4.31885143	1.89520975	-2.42364168	4.68E-80	2.79E-78
Cnnm1	115	22.91515993	29.81892057	6.903760645	6.07E-80	3.61E-78
Evc	54	37.30245675	27.84004098	-9.462415777	1.10E-79	6.48E-78
Stk39	92	9.431879258	5.02894567	-4.402933588	1.87E-79	1.10E-77
Klhl1	8	46.32792414	20.68558555	-25.64233859	5.61E-79	3.29E-77
Irx1	80	29.74003571	21.92766854	-7.812367175	1.83E-78	1.07E-76
Gm16793	48	37.98714282	28.7968447	-9.190298124	2.26E-78	1.32E-76
En2	89	16.17775607	23.95494663	7.77719056	2.46E-78	1.43E-76
Stox2	30	37.75377733	23.85528708	-13.89849025	2.53E-78	1.46E-76
Chst10	35	23.04324796	35.72687072	12.68362276	2.61E-78	1.50E-76
Bspry	30	21.67463643	10.41236785	-11.26226858	2.64E-78	1.51E-76
Calcr	27	23.40299224	11.79948156	-11.60351068	3.03E-78	1.73E-76
Lrrc3	33	21.31942348	36.10261685	14.78319337	3.07E-78	1.75E-76
Htr1b	32	2.808679148	12.18324891	9.374569759	3.32E-78	1.88E-76
Rprml	52	15.02994678	7.594041486	-7.435905292	3.07E-77	1.73E-75
Zfp37	36	7.086637814	1.695366221	-5.391271594	4.02E-77	2.26E-75
Pard3b	114	4.135335969	1.702006459	-2.433329509	1.16E-76	6.52E-75
Galnt13	103	43.48195728	50.93725822	7.455300944	1.24E-76	6.90E-75
Tgfbr3	27	7.665819144	1.381211641	-6.284607502	2.17E-76	1.20E-74
Col15a1	43	16.01367677	7.609070563	-8.404606212	2.23E-76	1.23E-74

Pus3	38	14.43740931	6.200255314	-8.237153992	2.94E-76	1.62E-74
Hpca	66	17.11719463	10.77541201	-6.341782618	4.48E-76	2.46E-74
Msi1	98	6.729321704	3.129576321	-3.599745383	9.47E-75	5.19E-73
Mir8092	17	12.8495841	3.402658244	-9.446925855	1.01E-74	5.51E-73
Pamr1	7	29.04202485	6.395130661	-22.64689419	2.46E-74	1.33E-72
Lpar3	73	26.01617188	33.1794261	7.163254224	7.04E-74	3.81E-72
Grik3	133	54.79416145	60.74277141	5.948609963	1.00E-73	5.41E-72
Lgi2	49	7.61517455	2.337352814	-5.277821735	1.60E-73	8.62E-72
Penk	50	12.32957315	5.123523957	-7.206049195	2.62E-73	1.40E-71
Snord37	6	70.05742106	35.26058863	-34.79683243	2.93E-73	1.56E-71
Ddx43	56	37.88720157	48.61894402	10.73174244	2.97E-73	1.58E-71
Clic6	8	15.59110758	52.281968	36.69086042	3.24E-73	1.71E-71
Zdhhc25	22	83.87993789	69.91403753	-13.96590035	3.75E-73	1.98E-71
Kcnk9	71	58.8479419	67.23713438	8.389192471	4.82E-73	2.53E-71
Nlrp5-ps	112	58.38283232	65.21176617	6.828933856	1.67E-72	8.76E-71
)30019B13F	10	23.05789646	56.24546643	33.18756997	2.37E-72	1.24E-70
Espnl	10	29.29152979	13.62613095	-15.66539884	2.82E-72	1.47E-70
Gldc	31	25.79891127	37.64606931	11.84715804	3.52E-72	1.82E-70
Fzd10	71	60.16917779	68.71353573	8.544357938	6.69E-72	3.46E-70
Kcnh1	66	8.326459215	3.677399182	-4.649060033	9.56E-72	4.92E-70
Trank1	94	43.78584207	53.36705432	9.581212242	1.25E-71	6.44E-70
Tspyl5	43	20.80626933	10.70231689	-10.10395244	1.50E-71	7.65E-70
Fam169a	148	4.75165452	2.012993455	-2.738661065	1.64E-71	8.37E-70
Ntng1	9	59.30074154	20.04664708	-39.25409446	1.95E-71	9.89E-70
Scrn1	41	4.835150107	1.388263217	-3.44688689	2.17E-71	1.10E-69
Rpp25	62	10.64958799	7.918344126	-2.731243866	2.56E-71	1.29E-69
Mir6537	31	79.94237953	63.85256976	-16.08980978	2.96E-71	1.49E-69
Vstm2b	72	48.22285423	56.11167044	7.888816208	3.66E-71	1.83E-69
Slc27a3	54	53.46883096	62.16780827	8.698977307	4.60E-71	2.30E-69
Dapk1	82	39.03497141	31.40580567	-7.629165742	7.40E-71	3.68E-69
Mir193b	18	11.40911748	2.02697954	-9.38213794	2.40E-70	1.19E-68
Shisa3	57	29.58046613	20.05861788	-9.521848249	2.66E-70	1.31E-68
Npas1	27	22.08906787	39.26425874	17.17519087	4.87E-70	2.40E-68
Lhfpl4	57	21.973942	14.56378928	-7.410152715	6.28E-70	3.08E-68

Acan	36	8.478941059	1.992819389	-6.48612167	6.90E-70	3.38E-68
Gmnc	8	24.05590469	7.437472547	-16.61843214	1.05E-69	5.10E-68
Tlx3	101	53.26446571	61.36560368	8.101137967	1.32E-69	6.40E-68
AI854517	112	35.63037386	42.6487633	7.018389449	1.47E-69	7.10E-68
Sv2c	57	16.27667218	9.19562753	-7.081044654	1.84E-69	8.90E-68
Homer2	95	4.254647508	1.776668202	-2.477979306	3.15E-69	1.52E-67
Osr1	5	42.89682371	4.00103213	-38.89579158	3.19E-69	1.53E-67
Gpc5	28	21.26354165	33.30594171	12.04240006	4.37E-69	2.09E-67
Radil	21	7.14869711	21.30824332	14.1595462	5.94E-69	2.83E-67
Ryr2	41	15.38833179	8.568668344	-6.819663448	6.41E-69	3.04E-67
Gm5089	25	13.08736878	2.576209921	-10.51115886	7.42E-69	3.51E-67
Nkx6-2	69	56.44787162	66.49047213	10.04260051	1.24E-68	5.86E-67
Dlx6	30	24.03328942	13.62154938	-10.41174004	1.36E-68	6.38E-67
Fbll1	30	43.2342451	58.54136771	15.30712261	2.01E-68	9.44E-67
Slc35f2	10	11.83176631	0.994353505	-10.8374128	3.46E-68	1.62E-66
Kcnd3	98	38.2463561	33.48953195	-4.756824147	3.91E-68	1.82E-66
Rasef	45	6.14245496	2.416701648	-3.725753312	4.24E-68	1.96E-66
Gm11240	45	6.14245496	2.416701648	-3.725753312	4.24E-68	1.96E-66
Mir7656	6	85.14294056	58.09969197	-27.04324859	2.12E-67	9.80E-66
Dyrk1b	64	2.563296939	6.263952813	3.700655873	2.26E-67	1.04E-65
Barx2	65	28.92182585	23.43564184	-5.486184015	8.38E-67	3.84E-65
Ddah2	13	37.05211723	15.98327153	-21.0688457	1.17E-66	5.36E-65
Kndc1	25	40.47240126	27.47072186	-13.0016794	1.23E-66	5.60E-65
Trim9	24	14.30342493	5.687529339	-8.615895589	1.32E-66	5.98E-65
Lepr	14	22.54492796	5.004010186	-17.54091777	1.50E-66	6.81E-65
)30025P21F	57	18.40714534	27.20770091	8.800555571	1.98E-66	8.95E-65
Chdh	40	15.79903007	8.541046304	-7.257983769	2.18E-66	9.83E-65
Foxd3	45	54.58637874	66.44492991	11.85855117	4.69E-66	2.11E-64
Cercam	27	14.89730294	30.93584311	16.03854017	4.86E-66	2.18E-64
Ephx2	18	11.18591081	3.279033095	-7.906877711	6.66E-66	2.97E-64
Cacna1h	91	3.214999385	7.241438899	4.026439514	7.25E-66	3.22E-64
Prr16	52	34.27593987	23.92964858	-10.3462913	8.17E-66	3.62E-64
Pcsk2	51	27.3805493	19.65691064	-7.723638662	2.36E-65	1.04E-63
Kbtbd13	16	18.69656073	35.92606263	17.22950189	2.73E-65	1.20E-63

Pld5	39	46.45871632	59.38684931	12.92813299	3.53E-65	1.55E-63
Marveld2	69	6.119263114	2.709237332	-3.410025782	4.54E-65	1.99E-63
Nell1	47	36.9703136	27.51917153	-9.451142071	8.68E-65	3.79E-63
Lhfpl2	68	4.696226283	1.945357095	-2.750869187	8.92E-65	3.89E-63
Phox2a	37	38.62378973	50.47690378	11.85311405	1.44E-64	6.26E-63
Lrrtm3	8	29.91033675	8.194564567	-21.71577218	1.56E-64	6.74E-63
Myrip	91	57.09861795	64.4626143	7.363996345	2.20E-64	9.47E-63
Zpbp	18	43.88248169	63.59510465	19.71262296	2.28E-64	9.82E-63
Lhb	29	66.79474951	50.87948023	-15.91526928	2.30E-64	9.86E-63
Dnaja4	79	4.039316449	1.274264426	-2.765052022	2.42E-64	1.03E-62
Tmem184a	25	60.61039972	47.55370949	-13.05669023	3.97E-64	1.69E-62
Gm3458	74	21.62675579	29.78576233	8.159006537	7.27E-64	3.09E-62
Cacna1c	111	8.85770302	5.698756934	-3.158946087	7.37E-64	3.13E-62
Wtip	35	2.234430728	9.13996436	6.905533632	1.85E-63	7.81E-62
Fam217b	38	60.10876449	72.33739932	12.22863483	2.38E-63	1.00E-61
Lrig3	95	43.9006347	51.5873651	7.686730398	3.10E-63	1.31E-61
Repin1	20	29.30861419	14.52018393	-14.78843026	4.19E-63	1.76E-61
Sec31b	28	23.20030429	14.31064708	-8.889657204	6.40E-63	2.67E-61
Cbln2	14	35.55773635	15.85741352	-19.70032284	9.12E-63	3.80E-61
Sohlh2	38	93.23491191	84.42818361	-8.8067283	9.51E-63	3.95E-61
Dlc1	74	25.78529157	19.08802092	-6.697270648	9.73E-63	4.03E-61
Gnmt	27	44.68042458	30.13989445	-14.54053013	9.96E-63	4.11E-61
Tmem200a	33	9.060734735	2.402485274	-6.65824946	1.07E-62	4.40E-61
T	55	30.09043355	23.05784389	-7.032589655	1.76E-62	7.23E-61
Pdpn	17	32.07614745	17.58236112	-14.49378633	2.15E-62	8.83E-61
Sorl1	48	5.866059723	14.52931201	8.663252291	2.55E-62	1.04E-60
Foxc2	77	52.22525922	60.28362485	8.058365628	2.83E-62	1.15E-60
Nkx3-2	68	37.22346293	30.54104524	-6.682417694	3.44E-62	1.40E-60
Cldn3	33	15.81428113	29.95194142	14.13766029	4.99E-62	2.02E-60
Nmb	24	16.79346284	28.81349805	12.02003522	6.35E-62	2.57E-60
Tkfc	5	64.33929458	30.20779153	-34.13150305	8.89E-62	3.58E-60
Tubg2	22	15.04412529	6.252523216	-8.791602074	1.12E-61	4.48E-60
Adam11	51	7.712355533	3.206653429	-4.505702103	3.00E-61	1.20E-59
Lrfn5	30	33.18218633	20.09544386	-13.08674247	3.68E-61	1.47E-59

Dlk1	61	52.01302622	45.29054521	-6.722481003	3.82E-61	1.52E-59
Arhgap8	17	11.81891721	2.387139732	-9.431777483	3.98E-61	1.58E-59
Pik3cd	44	17.68314827	10.0285873	-7.654560971	4.98E-61	1.98E-59
Mir6943	5	67.72234092	29.52732039	-38.19502052	6.47E-61	2.56E-59
She	143	33.86194628	39.94511944	6.083173163	6.51E-61	2.57E-59
Ephb2	116	6.001273932	3.521510631	-2.479763302	6.63E-61	2.61E-59
Gdf7	50	26.31039838	36.44011336	10.12971498	8.85E-61	3.47E-59
Gm829	9	66.29510332	40.76057115	-25.53453217	2.39E-60	9.35E-59
Adra2c	28	27.65836873	39.93112135	12.27275262	2.60E-60	1.01E-58
Ltk	112	8.04014965	4.689171967	-3.350977683	3.69E-60	1.44E-58
Cabp7	171	19.17961845	24.89294541	5.713326957	3.75E-60	1.45E-58
Trpv4	24	17.4361334	9.310263278	-8.125870127	5.75E-60	2.23E-58
Gm6194	26	89.86493994	76.85212634	-13.0128136	1.14E-59	4.41E-58
Tox	57	10.77046692	6.308054819	-4.462412103	1.30E-59	4.99E-58
Rai2	50	29.92987898	16.89787289	-13.03200609	2.33E-59	8.94E-58
Rtn1	65	37.05251244	30.50043607	-6.55207636	3.38E-59	1.30E-57
Hoxd11	100	52.75597881	61.4629748	8.70699598	3.61E-59	1.38E-57
Ccdc158	44	20.303295	30.31806069	10.01476568	6.42E-59	2.45E-57
Ptchd1	59	24.10548923	15.15283325	-8.952655988	6.88E-59	2.62E-57
Asxl3	85	26.52104973	33.52286026	7.00181053	7.97E-59	3.02E-57
H2-Q10	35	19.33247267	30.5287515	11.19627883	8.22E-59	3.11E-57
Gm16617	33	90.93485852	80.79421541	-10.14064311	8.45E-59	3.19E-57
Abcg1	32	12.3516865	5.461080084	-6.890606413	9.42E-59	3.55E-57
Ripply3	10	22.56504731	8.947574097	-13.61747321	2.65E-58	9.95E-57
Notum	123	7.51979075	5.775528665	-1.744262085	2.71E-58	1.01E-56
Necab3	21	19.26779217	33.35443207	14.0866399	3.07E-58	1.15E-56
Fzd9	94	26.91399245	21.43610486	-5.477887595	3.29E-58	1.23E-56
Kcnj12	124	8.930553532	6.063667199	-2.866886332	4.07E-58	1.51E-56
Chst13	15	15.49520395	28.23519257	12.73998862	6.75E-58	2.50E-56
Tuba4a	72	13.2180774	7.3417438	-5.876333596	1.20E-57	4.44E-56
Sstr4	55	52.9587841	62.92717696	9.968392865	2.22E-57	8.19E-56
Jph1	87	25.53335928	33.36466517	7.831305886	2.71E-57	9.97E-56
Gm20556	15	13.8725555	3.134066253	-10.73848925	6.48E-57	2.38E-55
Magohb	23	5.180896134	1.17919206	-4.001704074	7.04E-57	2.57E-55

Lrp2	68	13.97492125	9.555079287	-4.419841958	1.32E-56	4.82E-55
Scx	48	15.92433243	10.37228242	-5.552050015	1.38E-56	5.03E-55
Isl1	57	53.34757471	63.95433254	10.60675782	1.78E-56	6.46E-55
Gbx2	87	38.93098647	47.08488026	8.153893783	3.28E-56	1.19E-54
Atoh7	63	16.33920213	11.31016238	-5.029039747	3.88E-56	1.40E-54
Prima1	65	29.13171552	23.78402611	-5.34768941	4.12E-56	1.49E-54
Stc2	26	11.95726938	5.776989026	-6.180280353	4.28E-56	1.54E-54
Pla2r1	6	40.23674991	10.90257753	-29.33417238	4.60E-56	1.65E-54
Sema3b	7	76.38849866	40.46285251	-35.92564615	6.05E-56	2.16E-54
Cwh43	25	37.24539757	25.49277008	-11.75262749	9.84E-56	3.51E-54
Sntb1	37	18.65493899	13.8674757	-4.787463293	1.15E-55	4.09E-54
Mir6990	4	78.80774256	50.47933862	-28.32840395	1.27E-55	4.52E-54
Hdgfl1	9	91.76643637	76.11667407	-15.6497623	1.36E-55	4.80E-54
Sulf2	122	4.378953733	2.076432641	-2.302521092	1.61E-55	5.68E-54
Grm2	31	20.58781167	8.702505331	-11.88530634	3.89E-55	1.37E-53
BC021891	39	23.77133487	16.63042257	-7.140912302	8.14E-55	2.86E-53
Mesp1	28	24.39835392	37.82887151	13.43051759	1.16E-54	4.08E-53
Medag	8	32.41357777	8.129767982	-24.28380979	1.64E-54	5.74E-53
Ntrk3	61	32.48331986	24.40407756	-8.079242293	1.73E-54	6.05E-53
Pdx1	31	28.09869423	17.80203031	-10.29666392	2.30E-54	8.00E-53
Mir3544	9	83.95903574	61.02366216	-22.93537357	2.81E-54	9.74E-53
Mir540	9	83.95903574	61.02366216	-22.93537357	2.81E-54	9.74E-53
Tcfl5	96	67.87271766	74.65384802	6.78113036	3.88E-54	1.34E-52
Oxtr	17	23.22315492	8.905396947	-14.31775798	4.51E-54	1.56E-52
Cap2	31	21.94297103	14.15794908	-7.785021943	4.53E-54	1.56E-52
Lgi3	33	7.612231858	2.94420514	-4.668026719	4.90E-54	1.68E-52
Ube2q2	91	3.366211807	1.270856046	-2.095355761	5.50E-54	1.88E-52
Slc24a2	31	34.23073515	21.09233316	-13.13840199	8.32E-54	2.84E-52
Prr18	79	12.70224383	19.31432535	6.612081518	9.68E-54	3.30E-52
Pdlim4	52	14.26757046	21.39826539	7.130694923	1.09E-53	3.72E-52
Sim2	54	11.33320818	19.34107415	8.00786597	1.47E-53	5.00E-52
Eid2	74	4.935482839	2.083101553	-2.852381286	1.96E-53	6.64E-52
Unc5d	58	47.48703338	56.82567371	9.338640334	1.99E-53	6.73E-52
Soga3	16	28.39946893	15.63501914	-12.76444979	2.00E-53	6.76E-52

Adamts2	86	22.63930478	18.0027492	-4.636555578	2.75E-53	9.25E-52
P3h3	102	4.682605048	3.218394439	-1.464210609	3.87E-53	1.30E-51
Gyg	34	3.602482061	1.139225601	-2.46325646	4.91E-53	1.65E-51
Mei4	37	11.69368232	22.38298568	10.68930337	5.88E-53	1.97E-51
Klhl23	40	7.915076541	4.605359576	-3.309716966	9.63E-53	3.21E-51
Hoxd9	48	47.57012847	58.42654535	10.85641689	1.11E-52	3.71E-51
Foxd1	40	6.683355482	2.052907788	-4.630447694	1.27E-52	4.20E-51
Susd1	45	6.553644148	11.67477526	5.121131117	1.92E-52	6.37E-51
Fgf13	59	24.03075463	14.59422765	-9.436526985	2.95E-52	9.75E-51
Heyl	35	8.90065879	3.847257329	-5.053401461	4.26E-52	1.41E-50
Cited4	33	7.962085272	2.53438404	-5.427701232	4.81E-52	1.58E-50
Cyct	8	76.67608486	46.92551601	-29.75056885	8.08E-52	2.65E-50
Adcy7	47	15.57283675	23.93310587	8.360269118	1.57E-51	5.13E-50
Casd1	76	3.27451161	1.595080201	-1.679431409	1.74E-51	5.68E-50
Mag	8	69.62465806	39.82329803	-29.80136003	1.85E-51	6.04E-50
Tcf24	114	41.76762529	48.9436271	7.176001814	2.01E-51	6.54E-50
Spock2	39	14.3903801	24.1628125	9.772432397	2.39E-51	7.75E-50
Efcab6	10	60.5014111	31.00680879	-29.49460231	2.47E-51	7.99E-50
Nfe2	22	17.71813264	9.024699301	-8.693433336	3.52E-51	1.14E-49
Anks6	109	4.904347287	2.702595379	-2.201751909	3.65E-51	1.18E-49
Tc2n	104	11.80104102	8.056581255	-3.744459767	4.76E-51	1.53E-49
Tmem233	21	47.62267511	34.95778575	-12.66488936	4.93E-51	1.58E-49
Ebf3	99	14.70785209	11.57040625	-3.137445839	5.66E-51	1.81E-49
Frmd4b	46	39.46711561	30.23857554	-9.228540071	6.10E-51	1.95E-49
700039E15R	10	73.73043539	49.60839802	-24.12203737	6.76E-51	2.16E-49
Ankrd6	60	26.63559143	19.63731444	-6.99827699	7.14E-51	2.27E-49
Slc26a4	28	7.879771482	19.08325325	11.20348177	7.78E-51	2.47E-49
Pank1	88	2.57423673	0.973247504	-1.600989226	7.91E-51	2.51E-49
Cplx2	18	18.58376836	8.54626405	-10.03750431	8.80E-51	2.78E-49
Tmem178	109	18.08416706	14.67900747	-3.405159593	9.02E-51	2.85E-49
Nol4l	201	5.665270339	3.459149943	-2.206120396	9.63E-51	3.03E-49
AF529169	27	38.46437943	27.42342819	-11.04095124	1.11E-50	3.50E-49
Gm20554	30	46.28818406	33.42523046	-12.8629536	1.57E-50	4.93E-49
Ccdc64b	25	24.30071454	14.79383158	-9.506882964	1.85E-50	5.78E-49

Catsper1	4	84.61201919	45.41657347	-39.19544572	2.24E-50	7.00E-49
Gab1	62	5.975463327	2.959650774	-3.015812552	2.35E-50	7.31E-49
Ogfod2	17	26.97605541	47.16560517	20.18954976	3.07E-50	9.55E-49
Hs3st3b1	94	5.87912083	10.08133481	4.202213984	4.16E-50	1.29E-48
Ptk6	6	65.31079309	34.27781194	-31.03298115	4.35E-50	1.35E-48
Kcnq3	73	30.50302156	35.10185514	4.598833583	6.19E-50	1.91E-48
H2-Q8	8	10.81429012	2.518459506	-8.29583061	6.55E-50	2.02E-48
Rnls	18	4.824268045	0.657999241	-4.166268804	6.67E-50	2.05E-48
Eef1a2	22	34.43780724	48.816665	14.37885776	6.90E-50	2.12E-48
Cdh6	29	16.39838053	8.626077253	-7.772303279	7.46E-50	2.28E-48
'00125G02F	53	13.02887987	8.775581767	-4.253298099	8.92E-50	2.73E-48
Syt14	39	36.54691392	48.47931193	11.93239801	1.16E-49	3.53E-48
Mapk12	43	5.122858741	2.0577989	-3.065059842	1.44E-49	4.40E-48
i10027K06F	27	13.07550459	6.339577171	-6.735927421	1.69E-49	5.14E-48
Bfsp1	69	41.82838313	48.93218055	7.103797415	1.72E-49	5.21E-48
St8sia1	62	25.98693271	19.84409776	-6.142834953	2.02E-49	6.12E-48
Csmd2	49	58.13124398	69.96304709	11.83180311	2.51E-49	7.57E-48
Bves	67	42.36598626	50.83838931	8.472403042	2.63E-49	7.93E-48
Gpx7	38	31.66310194	42.46819291	10.80509097	2.73E-49	8.21E-48
Mdga2	44	48.17498915	57.82511249	9.650123338	2.84E-49	8.52E-48
Fam163a	41	24.0977804	32.90640544	8.808625041	2.95E-49	8.83E-48
Adrbk2	17	26.50465116	14.04919925	-12.45545191	3.10E-49	9.26E-48
Scd1	29	6.052882934	2.572032475	-3.480850459	3.40E-49	1.01E-47
Actn2	36	38.68167069	48.97775879	10.2960881	4.34E-49	1.29E-47
Ttc39c	59	3.475444885	1.281969394	-2.193475491	5.04E-49	1.50E-47
Drd2	58	50.87006713	60.32804708	9.457979951	5.59E-49	1.66E-47
Faxc	82	4.238008488	2.014008079	-2.224000409	6.23E-49	1.84E-47
330409D20F	31	38.11498438	48.06631848	9.951334108	8.68E-49	2.56E-47
Grem1	26	23.06186395	32.97942009	9.917556143	8.78E-49	2.59E-47
BC055111	19	10.94909606	3.360326584	-7.588769475	1.19E-48	3.49E-47
Neurl1a	99	6.442899193	3.473325609	-2.969573584	1.44E-48	4.21E-47
Pmp22	59	11.0435931	6.202508711	-4.841084392	1.47E-48	4.30E-47
Dao	6	90.45864471	63.9741685	-26.48447621	1.48E-48	4.34E-47
Sorbs3	66	8.860310426	5.415873436	-3.44443699	1.54E-48	4.50E-47

Kcnc3	60	4.439379183	8.917548664	4.478169481	2.94E-48	8.57E-47
Hdgfrp3	77	47.99173029	56.3631958	8.371465504	3.98E-48	1.16E-46
Fam19a2	4	12.47746156	36.92280754	24.44534597	5.01E-48	1.45E-46
Gm21949	7	57.3979544	36.02188773	-21.37606667	5.46E-48	1.58E-46
Iqcj	7	57.3979544	36.02188773	-21.37606667	5.46E-48	1.58E-46
700016H13F	25	6.351349752	2.24321977	-4.108129982	5.68E-48	1.64E-46
630019K06F	32	28.47121029	17.38052269	-11.09068761	6.76E-48	1.94E-46
Kcnj6	16	46.58151384	26.72983548	-19.85167836	8.17E-48	2.35E-46
Popdc3	38	17.21600518	9.932231788	-7.283773388	1.02E-47	2.92E-46
Arsb	57	5.045318697	1.378534941	-3.666783756	1.06E-47	3.02E-46
Cxcl12	38	57.82471167	69.13382521	11.30911355	1.15E-47	3.29E-46
Kcnj3	15	33.82483417	21.09068441	-12.73414976	1.55E-47	4.42E-46
Prob1	17	18.95173876	33.47313721	14.52139844	1.92E-47	5.46E-46
Dapk2	57	5.209060781	2.37619384	-2.832866941	2.46E-47	6.98E-46
Gldn	17	42.25799943	30.98348154	-11.2745179	2.94E-47	8.34E-46
Chrna1	6	58.89837823	30.93956561	-27.95881262	3.01E-47	8.50E-46
Nefm	48	44.45314472	57.09489258	12.64174786	3.05E-47	8.62E-46
Qrfpr	10	27.06631319	14.76009037	-12.30622282	4.07E-47	1.15E-45
Fgf20	33	16.59068163	26.64805982	10.05737818	4.15E-47	1.17E-45
Krt72	11	93.35654108	73.1767274	-20.17981368	4.18E-47	1.17E-45
Gdf6	74	14.80387224	11.21423538	-3.589636859	4.77E-47	1.33E-45
630025P09F	38	5.434170024	1.15752367	-4.276646354	6.11E-47	1.71E-45
Bfsp2	5	74.37315465	50.33548985	-24.0376648	6.23E-47	1.74E-45
931419H13F	58	18.7744207	13.48255304	-5.291867657	7.46E-47	2.08E-45
Dcaf12l1	26	47.22297437	61.61891688	14.39594251	9.74E-47	2.71E-45
Cntln	59	31.59035503	40.7798419	9.189486875	1.00E-46	2.79E-45
Syne1	18	8.334645562	20.33425257	11.99960701	1.01E-46	2.80E-45
Zfp422	65	3.9609896	1.721049513	-2.239940087	1.01E-46	2.80E-45
Tdrd5	63	25.22139627	32.86865165	7.647255387	1.16E-46	3.20E-45
Gm5607	62	32.73765877	40.75745907	8.019800302	1.35E-46	3.72E-45
Grin1os	34	6.954117499	2.680831818	-4.27328568	2.22E-46	6.10E-45
Mybpc3	4	91.64011232	58.09690174	-33.54321059	3.25E-46	8.92E-45
Mroh5	65	26.0549105	20.99688658	-5.058023912	3.38E-46	9.25E-45
Dlgap3	102	7.235404509	4.423227159	-2.81217735	4.51E-46	1.23E-44

Gjb6	33	16.84936725	8.290171313	-8.559195942	4.91E-46	1.34E-44
Nkd2	28	12.58280198	3.897378619	-8.685423363	5.21E-46	1.42E-44
Sox17	25	46.68351143	59.00840158	12.32489015	5.48E-46	1.49E-44
Krtdap	11	69.66865929	51.52405338	-18.14460592	6.46E-46	1.75E-44
)30412G12F	64	50.64930799	58.50570047	7.856392485	7.57E-46	2.05E-44
Slc30a2	52	12.22564029	18.64024684	6.414606549	7.64E-46	2.06E-44
Slc12a8	14	17.19026209	7.545816558	-9.644445534	8.57E-46	2.31E-44
Cspg4	12	52.19713701	74.56321089	22.36607389	8.63E-46	2.32E-44
Cmya5	16	29.70124315	18.35655387	-11.34468928	9.32E-46	2.50E-44
Olfml1	7	72.90697192	49.72910069	-23.17787124	1.02E-45	2.75E-44
Ppic	37	3.44830805	7.614006517	4.165698467	1.46E-45	3.90E-44
)33406I18R	50	4.642623065	1.928327851	-2.714295214	1.71E-45	4.58E-44
Tmem163	65	4.094313168	1.63928695	-2.455026218	2.73E-45	7.29E-44
Amn	37	36.04241725	46.46204643	10.41962918	2.92E-45	7.79E-44
Chd5	118	8.335823591	12.2549495	3.919125908	3.24E-45	8.61E-44
Gfra1	41	8.971303348	4.671473044	-4.299830304	3.72E-45	9.88E-44
Fam78a	125	7.064520914	10.52988183	3.465360913	3.99E-45	1.06E-43
Gdf10	78	31.01019227	26.95516353	-4.05502874	4.02E-45	1.06E-43
Mixl1	45	19.22920751	27.9251383	8.695930787	4.32E-45	1.14E-43
Efhd1	43	7.422201882	14.44078069	7.018578806	5.63E-45	1.48E-43
Pax6	92	32.31687713	39.75031036	7.433433226	6.35E-45	1.67E-43
Ankrd29	31	5.655667722	1.7772771	-3.878390622	6.63E-45	1.74E-43
Rhbdl3	71	7.409858488	4.602731367	-2.807127121	7.06E-45	1.85E-43
Aqp3	25	19.8324145	12.23649797	-7.595916531	7.30E-45	1.91E-43
Rims1	64	17.00279371	12.06909317	-4.933700541	7.36E-45	1.92E-43
Ahrr	19	12.13580359	3.20831348	-8.927490115	1.53E-44	3.98E-43
Lhcgr	26	71.04537018	57.57317496	-13.47219522	1.93E-44	5.03E-43
Zfp518a	25	8.330005001	2.663236841	-5.66676816	2.08E-44	5.41E-43
Tox3	115	63.88509296	69.26852509	5.383432132	2.16E-44	5.59E-43
Colgalt2	27	11.84049032	25.62475104	13.78426072	2.47E-44	6.38E-43
Rnf186	16	73.52770345	56.93871842	-16.58898502	2.63E-44	6.80E-43
Pde11a	12	53.32696797	37.89692343	-15.43004454	3.25E-44	8.39E-43
Mir6385	15	89.17293665	76.3844396	-12.78849705	3.35E-44	8.64E-43
Arhgef3	19	26.95324032	21.01231835	-5.94092197	3.40E-44	8.74E-43

Fam110b	42	21.5340564	13.9852456	-7.548810798	5.94E-44	1.52E-42
Mir6366	8	89.29490788	70.1894935	-19.10541438	6.49E-44	1.66E-42
Ccdc185	22	92.83690392	81.82662634	-11.01027759	7.27E-44	1.86E-42
Kcnh2	83	9.329907637	6.884750721	-2.445156915	8.04E-44	2.05E-42
Lhpp	21	3.022477703	12.62446277	9.601985063	8.08E-44	2.06E-42
Erbb4	55	37.79190219	47.48980968	9.697907486	1.63E-43	4.15E-42
Timp3	32	13.09897741	6.88509251	-6.213884901	1.95E-43	4.96E-42
Thrb	81	5.069214518	9.845217021	4.776002504	2.36E-43	5.98E-42
Six3os1	38	18.76277803	13.82271593	-4.940062098	2.72E-43	6.89E-42
Cabyr	23	9.4335945	3.468941483	-5.964653017	3.90E-43	9.83E-42
9-Sep	110	27.74210528	22.96702281	-4.77508247	3.90E-43	9.83E-42
Otud7a	84	53.87956636	59.89574581	6.016179449	4.81E-43	1.21E-41
Gabrb3	93	52.46238301	58.6286934	6.166310391	5.54E-43	1.39E-41
Clgn	31	14.50218725	21.55342913	7.051241875	1.35E-42	3.38E-41
Pcdh19	31	29.99094885	18.71966644	-11.2712824	2.22E-42	5.55E-41
Erc2	49	5.18199256	2.29792434	-2.88406822	3.12E-42	7.81E-41
Ttll3	51	58.51888841	51.66832468	-6.850563729	3.40E-42	8.48E-41
Cebpe	17	60.57820471	44.24342717	-16.33477753	4.01E-42	9.98E-41
Nfatc2	82	12.4989028	18.93725016	6.438347352	4.37E-42	1.09E-40
Nrip3	60	11.122711	6.499799684	-4.622911318	4.46E-42	1.11E-40
Bmp3	30	15.23658864	6.815645025	-8.420943613	4.76E-42	1.18E-40
Rnf208	20	30.75897665	21.52310082	-9.235875836	4.78E-42	1.18E-40
Ebf1	41	26.48981047	20.28354952	-6.206260955	5.12E-42	1.27E-40
Gm996	18	33.32171188	45.60614553	12.28443365	5.20E-42	1.28E-40
Dennd2a	99	8.882400509	6.975052699	-1.90734781	6.03E-42	1.48E-40
Shd	29	13.11319373	21.07673498	7.963541252	7.00E-42	1.72E-40
Fgf6	9	79.88646605	61.40294962	-18.48351643	8.78E-42	2.15E-40
H2-Q5	11	17.58228844	4.938969411	-12.64331903	8.79E-42	2.15E-40
Gnao1	108	7.007739873	3.773702288	-3.234037585	1.06E-41	2.59E-40
Aebp1	31	12.65640406	5.426813357	-7.229590707	1.07E-41	2.62E-40
Tmem88	22	60.27052225	50.25723079	-10.01329146	1.12E-41	2.73E-40
Fes	11	16.16319084	4.853504328	-11.30968651	1.18E-41	2.88E-40
30006D01F	14	58.22335308	43.13415822	-15.08919486	1.91E-41	4.63E-40
Ajap1	157	62.97213502	67.64789171	4.675756695	2.35E-41	5.69E-40

Syndig1l	74	10.19891449	15.10207118	4.903156687	2.51E-41	6.07E-40
Mir128-2	13	62.34112493	46.8872194	-15.45390554	2.56E-41	6.19E-40
Ccnjl	77	7.731932969	4.201412061	-3.530520908	2.56E-41	6.19E-40
Slfn10-ps	11	49.72023791	30.59285181	-19.1273861	3.01E-41	7.25E-40
Tshz3	100	48.92746977	54.28021026	5.35274049	3.62E-41	8.71E-40
Matk	50	20.76825701	16.40407938	-4.36417763	5.13E-41	1.23E-39
Vgll2	34	17.5031718	27.11266875	9.609496947	5.37E-41	1.29E-39
Negr1	54	51.07495317	58.93867074	7.863717568	5.46E-41	1.31E-39
Rab11fip5	70	4.49951761	1.688917149	-2.810600462	5.69E-41	1.36E-39
Fbxo17	13	28.06180802	15.01167777	-13.05013025	6.12E-41	1.46E-39
Fzd6	44	4.081734507	1.569385468	-2.512349039	1.23E-40	2.93E-39
Lrrc75b	84	6.086394537	3.061698946	-3.024695591	1.32E-40	3.15E-39
Plcb1	76	5.528038625	2.808751913	-2.719286712	1.81E-40	4.29E-39
Mir1901	64	7.901721895	3.561633473	-4.340088422	1.90E-40	4.50E-39
Efcc1	51	6.480122543	2.865364659	-3.614757884	2.59E-40	6.14E-39
Tcte1	3	84.55288091	51.66022743	-32.89265348	3.08E-40	7.28E-39
Pde3a	26	48.15968568	58.70929106	10.54960538	3.67E-40	8.66E-39
Zfp882	19	8.85469139	2.075625764	-6.779065626	5.91E-40	1.39E-38
Slc2a4	15	6.991678222	18.56877689	11.57709867	5.97E-40	1.40E-38
Slain1	61	6.184398634	3.366844365	-2.817554268	6.48E-40	1.52E-38
Pkdrej	39	10.82688789	6.785565841	-4.041322051	7.40E-40	1.73E-38
Cited1	66	25.28109138	16.94537251	-8.335718874	7.51E-40	1.76E-38
Dlx4	43	7.65158779	2.569358242	-5.082229548	1.03E-39	2.41E-38
Spata6	45	25.31783057	36.25729445	10.93946388	1.04E-39	2.43E-38
Arhgap44	50	7.046515048	3.528604175	-3.517910873	1.27E-39	2.96E-38
Pgbd5	176	4.927817509	2.926396561	-2.001420948	2.04E-39	4.75E-38
700017J07R	10	31.39897501	11.63492087	-19.76405414	2.08E-39	4.82E-38
Slc47a1	26	15.98971194	6.822453121	-9.167258816	2.20E-39	5.10E-38
Mkx	32	7.226541926	2.668011087	-4.558530839	2.64E-39	6.12E-38
Fktn	39	34.03081065	44.71037534	10.67956469	2.91E-39	6.73E-38
Peg10	12	55.09404527	37.19635579	-17.89768948	4.14E-39	9.55E-38
Rbm24	17	31.97084774	21.89546064	-10.0753871	4.23E-39	9.75E-38
333428L15R	46	36.09594973	27.29532575	-8.800623982	4.25E-39	9.78E-38
Cacna1i	95	34.44946464	40.0711414	5.621676768	4.96E-39	1.14E-37

Svep1	32	33.17620502	42.25824566	9.082040645	7.86E-39	1.80E-37
Mir6923	13	75.66738681	58.73225393	-16.93513288	8.02E-39	1.84E-37
Pde3b	57	3.350341627	1.143314326	-2.207027301	9.11E-39	2.08E-37
Amigo3	7	55.62417561	36.34614537	-19.27803024	1.09E-38	2.48E-37
Mir874	4	73.81441552	50.70404874	-23.11036678	1.31E-38	2.98E-37
Mir6913	20	83.4906711	71.36632019	-12.12435091	1.68E-38	3.82E-37
Tctn2	16	21.65913359	9.770258712	-11.88887488	1.74E-38	3.96E-37
Mfap3l	69	5.562446799	2.623714317	-2.938732482	1.81E-38	4.10E-37
Lyl1	28	44.21732846	55.90212767	11.68479921	3.64E-38	8.25E-37
Begain	83	4.95641457	9.476520782	4.520106213	4.72E-38	1.07E-36
Tmem132d	74	60.21931355	66.45301546	6.233701914	5.14E-38	1.16E-36
Arhgef7	226	6.481624032	4.708170341	-1.773453691	5.22E-38	1.18E-36
Fry	15	36.76240482	21.71213746	-15.05026736	7.40E-38	1.67E-36
Il13	6	81.80555438	60.91696484	-20.88858954	9.13E-38	2.05E-36
Gpr150	12	18.49597743	7.564774697	-10.93120273	1.09E-37	2.44E-36
Ddx25	61	11.83690186	8.674437173	-3.162464689	1.17E-37	2.62E-36
Kcnj2	26	44.52595084	55.59143948	11.06548865	1.55E-37	3.46E-36
Wt1	28	47.72883181	58.06570453	10.33687271	1.58E-37	3.53E-36
Mnx1	66	24.04254771	20.61838537	-3.424162345	1.58E-37	3.53E-36
Nkx6-1	101	45.692665	51.06533773	5.372672729	1.73E-37	3.85E-36
Gdnf	84	33.47539039	28.88758017	-4.587810219	1.78E-37	3.96E-36
Cxcr4	11	22.03784982	7.725060836	-14.31278898	1.95E-37	4.33E-36
Tmem72	9	84.81850516	67.45174528	-17.36675988	2.12E-37	4.69E-36
Fbxl16	81	4.285649218	2.219951762	-2.065697455	2.22E-37	4.91E-36
'30457N03F	21	53.2760318	42.65409326	-10.62193854	2.38E-37	5.25E-36
Hoxb5	16	36.65057267	50.25782439	13.60725173	2.47E-37	5.46E-36
Shisa2	62	8.919629907	5.507109355	-3.412520552	2.64E-37	5.80E-36
Layn	18	40.11182873	29.49935735	-10.61247138	2.64E-37	5.80E-36
Fabp3	22	13.3757785	5.602915552	-7.772862944	2.64E-37	5.80E-36
Arrdc4	17	11.33445766	2.762182858	-8.572274807	2.97E-37	6.52E-36
Fbxo39	16	23.41115563	11.08987574	-12.32127989	3.13E-37	6.84E-36
Ttbk1	64	6.085721077	10.6539673	4.568246223	3.69E-37	8.08E-36
Gm8096	4	85.19046077	59.07060921	-26.11985156	3.94E-37	8.60E-36
Colec12	63	33.81107049	27.93037882	-5.880691673	4.27E-37	9.32E-36

Gm9899	72	14.71680577	21.84901642	7.132210645	4.35E-37	9.47E-36
Amigo2	109	3.342859667	2.03153561	-1.311324057	5.37E-37	1.17E-35
1-Mar	30	49.79710989	61.02147837	11.22436847	5.43E-37	1.18E-35
Agbl2	20	41.67405839	55.11721566	13.44315727	5.89E-37	1.28E-35
Bmpr1b	43	42.8515887	35.45434193	-7.397246776	6.32E-37	1.37E-35
Stac	60	47.59478079	55.2746789	7.67989811	6.52E-37	1.41E-35
Gata5	55	11.09798697	6.795138136	-4.302848838	7.16E-37	1.55E-35
Myb	56	17.47582155	15.13021844	-2.345603114	7.29E-37	1.57E-35
Cd200r2	5	73.22008091	47.50313246	-25.71694846	8.28E-37	1.78E-35
Cxcl2	5	42.88118409	16.49538559	-26.3857985	1.02E-36	2.20E-35
Cyp26a1	32	25.09676866	34.5298178	9.433049142	1.15E-36	2.48E-35
Mbnl3	28	24.68700742	15.65206568	-9.034941737	1.47E-36	3.15E-35
Slc5a4a	6	79.40230665	58.13941031	-21.26289634	1.67E-36	3.57E-35
Kcnh5	40	39.46430059	31.3294736	-8.13482699	1.78E-36	3.81E-35
Dnah7b	21	9.34971782	3.603373914	-5.746343905	1.86E-36	3.97E-35
Rnf144b	35	9.797134074	5.289785177	-4.507348897	1.92E-36	4.08E-35
Mks1	14	8.011799436	16.92719507	8.915395637	1.97E-36	4.19E-35
Arhgap20	99	11.08032051	9.10635714	-1.973963365	1.97E-36	4.19E-35
Slc22a17	60	4.772859411	1.834461618	-2.938397793	2.43E-36	5.16E-35
Rgs6	51	39.35606647	30.78405462	-8.572011853	2.91E-36	6.17E-35
Wscd2	3	74.45527097	44.87347203	-29.58179894	3.49E-36	7.37E-35
Mir8117	9	85.08817691	67.1349815	-17.95319541	3.83E-36	8.08E-35
Erich5	3	31.96662619	9.969803234	-21.99682296	4.13E-36	8.72E-35
Gm4598	10	40.65212428	25.56734637	-15.08477791	4.61E-36	9.71E-35
Six2	140	28.84768154	25.95163864	-2.896042908	4.91E-36	1.03E-34
Cd248	58	7.417296451	4.901960972	-2.51533548	7.92E-36	1.66E-34
Tpte	3	64.74782657	28.1838703	-36.56395627	1.07E-35	2.24E-34
Crhbp	8	67.01298672	47.52860405	-19.48438266	1.11E-35	2.32E-34
930014A18R	7	47.08506855	21.69122152	-25.39384703	1.19E-35	2.49E-34
Mcc	53	5.182907925	2.222692626	-2.960215298	1.77E-35	3.69E-34
Cabp2	11	91.54830424	76.86992997	-14.67837426	1.88E-35	3.92E-34
Mir7231	3	76.53774492	54.2289413	-22.30880362	2.00E-35	4.16E-34
Klf14	105	58.58862359	63.20426119	4.615637604	2.69E-35	5.59E-34
Man1c1	104	3.354239842	6.080529995	2.726290153	4.13E-35	8.57E-34

Nkd1	105	30.5313572	26.02759484	-4.50376236	4.16E-35	8.62E-34
Mctp1	80	7.470517592	6.24487403	-1.225643562	4.30E-35	8.89E-34
Twist1	86	53.80660824	60.59607905	6.789470807	5.03E-35	1.04E-33
Gpr35	3	84.8590959	50.2635104	-34.5955855	5.08E-35	1.05E-33
Kcns3	54	34.66849974	28.77254582	-5.895953924	5.10E-35	1.05E-33
Pak7	11	48.54257513	32.07054994	-16.47202519	7.16E-35	1.47E-33
Mcts2	18	74.44983187	62.50744267	-11.9423892	7.42E-35	1.53E-33
Prkar1b	53	19.20609527	13.19013263	-6.015962636	9.10E-35	1.87E-33
Glp1r	30	13.37541516	9.351879588	-4.023535576	1.24E-34	2.53E-33
Adgrg2	33	28.01243264	17.81879947	-10.19363317	1.25E-34	2.56E-33
Brinp2	21	32.4761124	42.54007826	10.06396586	1.37E-34	2.81E-33
Tacc1	82	3.730030291	1.723845753	-2.006184538	1.45E-34	2.95E-33
Ptgs2os	17	6.62214383	2.396129427	-4.226014403	1.53E-34	3.12E-33
Fam105a	53	5.491162583	10.82110401	5.32994143	1.56E-34	3.17E-33
St6galnac2	78	7.435291095	4.390547273	-3.044743821	1.86E-34	3.78E-33
Barx1	113	57.68470067	62.73538836	5.050687686	1.98E-34	4.01E-33
Kdr	23	10.77537766	19.07052794	8.295150282	2.39E-34	4.84E-33
Amph	23	18.4559215	13.11154492	-5.344376582	2.66E-34	5.38E-33
Pcdh18	23	7.115116016	2.496891623	-4.618224394	4.48E-34	9.05E-33
Ankrd33b	53	21.30320391	16.61599125	-4.687212667	4.59E-34	9.26E-33
Ncoa1	88	3.014374883	0.99974221	-2.014632673	4.66E-34	9.40E-33
Wnt2b	79	9.50283341	7.900283815	-1.602549594	4.95E-34	9.97E-33
Sod3	5	59.09509771	35.83319482	-23.26190289	4.96E-34	9.97E-33
Agxt	11	92.1771279	80.94140084	-11.23572706	6.55E-34	1.31E-32
Hecw2	59	13.05877701	20.68486328	7.626086264	7.03E-34	1.41E-32
Pnliprp2	8	65.15490077	49.18882635	-15.96607443	8.00E-34	1.60E-32
Frmpd1os	3	71.23695976	27.3268827	-43.91007706	8.13E-34	1.63E-32
Mir7023	12	87.88861885	72.80900603	-15.07961282	9.72E-34	1.94E-32
Prickle2	52	25.72754987	20.76092643	-4.966623441	1.16E-33	2.32E-32
Atp2b2	78	34.93075438	29.21107961	-5.719674764	1.27E-33	2.53E-32
Npas4	28	48.57611239	43.26772439	-5.308388008	1.36E-33	2.71E-32
Rgs20	39	6.956929159	2.361139066	-4.595790094	1.40E-33	2.77E-32
Padi2	3	20.71522732	3.799316337	-16.91591099	1.56E-33	3.09E-32
Kcnk18	15	82.00334671	68.85385959	-13.14948711	1.85E-33	3.66E-32

Neurog1	28	32.47499953	22.94757739	-9.527422135	1.87E-33	3.70E-32
Otogl	3	75.65242225	42.73289527	-32.91952698	1.99E-33	3.93E-32
Adamts8	72	10.04233853	15.57181601	5.529477474	2.57E-33	5.07E-32
Zcchc3	10	33.70544276	57.11890221	23.41345945	2.76E-33	5.44E-32
Spsb4	89	4.137211014	2.653822094	-1.483388919	3.12E-33	6.14E-32
Atp12a	29	13.78341893	8.503698641	-5.279720292	3.14E-33	6.17E-32
Fyn	87	2.919665083	1.350370745	-1.569294337	3.35E-33	6.56E-32
Tmprss6	5	89.83071687	62.29196736	-27.53874951	3.60E-33	7.06E-32
Ptprd	62	5.708656921	2.867961249	-2.840695673	4.32E-33	8.46E-32
Gm2694	45	55.61049053	64.28985558	8.679365041	4.46E-33	8.72E-32
Ccdc27	10	53.88649751	35.11087414	-18.77562337	6.38E-33	1.24E-31
Fzd3	103	3.527382169	1.92383733	-1.603544839	7.19E-33	1.40E-31
Pou3f3	127	46.94340718	51.63637095	4.692963767	7.65E-33	1.49E-31
Gimap1	6	36.6034669	12.03087202	-24.57259487	8.23E-33	1.60E-31
Satb2	3	36.41231593	8.134371159	-28.27794477	8.57E-33	1.66E-31
Klf15	60	14.98927963	10.55496005	-4.434319573	9.94E-33	1.93E-31
Msx1	47	33.53618087	27.74751629	-5.788664573	1.05E-32	2.03E-31
Apoe	19	17.75153076	7.474660622	-10.27687014	1.08E-32	2.10E-31
Slc25a21	23	55.83868083	66.03433507	10.19565423	1.11E-32	2.14E-31
Edn2	16	15.60420997	5.441767849	-10.16244212	1.16E-32	2.23E-31
Mga	18	3.386849799	11.91879528	8.531945477	1.25E-32	2.40E-31
Shc3	58	51.92720102	61.48062618	9.553425154	1.28E-32	2.46E-31
Nkain4	12	28.24128421	15.87970107	-12.36158313	1.31E-32	2.51E-31
Kcnn3	8	49.97051867	64.44285144	14.47233277	1.35E-32	2.59E-31
Ldhd	6	28.9951442	11.79097043	-17.20417377	1.39E-32	2.66E-31
Aqp5	90	19.57488936	24.14476239	4.569873026	1.81E-32	3.46E-31
Sox30	110	51.52263349	56.17901551	4.656382021	2.12E-32	4.05E-31
Fam81a	82	3.370698149	1.510645921	-1.860052228	2.27E-32	4.34E-31
Clec2l	79	6.107312751	4.028382245	-2.078930507	2.39E-32	4.55E-31
Hcn1	50	50.07485273	58.51619647	8.44134374	2.52E-32	4.80E-31
Frem2	26	44.58126769	55.87266329	11.2913956	2.75E-32	5.22E-31
Nkx2-2os	44	39.29924789	47.65249181	8.35324392	2.92E-32	5.54E-31
30015N05R	21	27.9206582	18.04588164	-9.87477657	3.59E-32	6.80E-31
Sowahb	72	5.525174278	3.291318109	-2.233856169	3.73E-32	7.07E-31

Tomm20	48	13.97345391	10.04348665	-3.929967261	5.36E-32	1.01E-30
Pak1	5	7.745002206	29.15800691	21.41300471	5.56E-32	1.05E-30
Syk	62	9.672491072	5.631330395	-4.041160677	5.67E-32	1.07E-30
Irf6	28	13.84707625	8.038683603	-5.808392652	6.27E-32	1.18E-30
Gm16532	3	30.5542737	7.39078746	-23.16348624	6.49E-32	1.22E-30
Cmtm1	6	89.35185185	68.67326499	-20.67858686	6.52E-32	1.23E-30
Fgf5	47	17.29987022	23.29888476	5.999014542	7.64E-32	1.43E-30
230112D13F	12	76.31369362	65.04888464	-11.26480898	8.13E-32	1.52E-30
Rbfox1	12	48.18927636	36.2146564	-11.97461996	8.60E-32	1.61E-30
Sdk2	95	52.60746491	57.49193728	4.884472365	8.85E-32	1.65E-30
Tmem223	20	48.16778045	57.38551496	9.217734512	9.01E-32	1.68E-30
Bicc1	108	2.27004103	4.157169929	1.8871289	1.19E-31	2.22E-30
Gm11733	12	62.73590618	48.17572095	-14.56018523	1.39E-31	2.59E-30
Gm10863	11	75.29424362	60.18524217	-15.10900146	1.40E-31	2.60E-30
Phc2	155	10.71530434	14.41014633	3.694841994	1.40E-31	2.60E-30
Glipr2	38	7.301620096	14.02083179	6.719211691	1.57E-31	2.92E-30
Hcn4	170	8.928120304	7.514420218	-1.413700086	1.61E-31	2.99E-30
Tspan33	64	4.959265712	2.326561333	-2.632704379	1.63E-31	3.01E-30
Mir1191b	8	24.07999555	8.563070765	-15.51692479	1.91E-31	3.53E-30
AI854703	22	17.08818881	10.8452985	-6.242890313	2.01E-31	3.72E-30
Ckm	9	65.826334	48.08970792	-17.73662608	2.03E-31	3.75E-30
Snph	46	6.22659042	12.26555261	6.038962186	2.05E-31	3.78E-30
Kcnq1	48	7.297391697	4.035974683	-3.261417014	2.24E-31	4.12E-30
Sowaha	9	45.83616641	60.54737177	14.71120536	2.30E-31	4.22E-30
Slc9a2	126	3.229812857	1.327617201	-1.902195656	2.37E-31	4.36E-30
Map7d2	69	22.28413542	16.59678478	-5.687350635	2.45E-31	4.49E-30
Mb21d1	25	27.0643281	20.62114628	-6.443181824	2.47E-31	4.52E-30
Oprd1	79	17.05772628	12.17389424	-4.883832047	2.48E-31	4.53E-30
H2-Q4	35	5.520788403	2.255250772	-3.265537631	2.81E-31	5.14E-30
Gpr4	15	11.18136975	5.870820338	-5.310549409	2.85E-31	5.19E-30
Gng4	71	8.572397785	5.623040457	-2.949357329	2.91E-31	5.29E-30
Mir5120	2	76.41012337	40.19416742	-36.21595596	2.98E-31	5.41E-30
Atg9b	2	79.5023511	29.65528222	-49.84706887	2.98E-31	5.41E-30
930554H23F	19	54.33878133	62.85970772	8.520926393	3.00E-31	5.44E-30

Dgkh	58	28.3469439	23.10218129	-5.244762613	3.16E-31	5.72E-30
Peg13	13	40.9710478	59.7367686	18.76572081	3.68E-31	6.66E-30
Tekt5	28	24.30775919	14.90380884	-9.403950353	3.70E-31	6.69E-30
Scin	18	9.321419229	3.55723518	-5.764184048	3.73E-31	6.73E-30
Tnfsf11	36	26.79640516	33.81728924	7.020884073	3.88E-31	6.99E-30
Rnf165	72	42.38492085	48.56559006	6.180669205	4.29E-31	7.72E-30
Fgd5	9	76.74089904	60.06653457	-16.67436447	4.54E-31	8.17E-30
730409E04R	34	6.964331435	4.554659857	-2.409671577	4.92E-31	8.84E-30
Clstn2	75	21.65743741	27.05912939	5.401691983	5.00E-31	8.97E-30
Atcayos	8	18.62455724	8.455703084	-10.16885415	5.43E-31	9.73E-30
Hoxa13	45	22.68224012	17.96939084	-4.712849282	5.79E-31	1.04E-29
Lrp3	119	6.467060655	9.661099327	3.194038672	5.88E-31	1.05E-29
Pde1c	4	19.95916566	4.692970223	-15.26619543	6.58E-31	1.17E-29
.30019P16R	19	6.380147957	2.403441485	-3.976706472	6.67E-31	1.19E-29
Zfp275	39	21.79498147	13.28598853	-8.508992943	7.82E-31	1.39E-29
Gipc2	44	4.409755667	1.758657869	-2.651097798	7.96E-31	1.42E-29
Tsks	6	73.53279632	49.24287515	-24.28992117	9.03E-31	1.60E-29
Prmt8	78	30.23510423	26.24685006	-3.988254175	1.01E-30	1.78E-29
Mir148a	70	26.48195557	23.33933476	-3.14262081	1.08E-30	1.91E-29
Plec	82	13.5390366	8.505303643	-5.033732957	1.13E-30	1.99E-29
Prph2	16	87.49816762	76.96685433	-10.53131329	1.23E-30	2.17E-29
Hrh1	50	14.67805208	19.32480521	4.646753131	1.29E-30	2.27E-29
Tacr3	21	44.63330511	36.67504642	-7.958258694	1.60E-30	2.82E-29
Ica1l	121	2.617309926	1.437626639	-1.179683286	1.62E-30	2.85E-29
Tunar	33	51.49846158	60.03773762	8.539276045	1.82E-30	3.21E-29
Arhgdig	74	5.258964565	2.502516562	-2.756448003	1.86E-30	3.28E-29
Amer3	47	18.46198597	14.14321912	-4.318766852	2.00E-30	3.51E-29
Asz1	17	89.11353131	79.66913962	-9.44439169	2.30E-30	4.03E-29
Igdcc4	31	4.587912113	2.034389744	-2.55352237	2.39E-30	4.19E-29
Lrrc6	10	17.55345432	9.184403179	-8.369051145	2.60E-30	4.55E-29
'00028K03R	6	73.32851915	85.99842871	12.66990956	2.77E-30	4.84E-29
Vax1	61	27.24737547	23.80879733	-3.438578135	3.49E-30	6.09E-29
Zfp947	6	21.38964093	7.898252124	-13.49138881	3.83E-30	6.67E-29
Gbx1	176	12.17067983	10.31387658	-1.856803255	3.91E-30	6.80E-29

Pcsk9	12	15.39200464	7.579067725	-7.812936916	4.32E-30	7.51E-29
Olfr279	3	67.56901766	33.35467606	-34.2143416	4.57E-30	7.93E-29
Prdm13	18	25.21379173	42.90696435	17.69317262	4.58E-30	7.94E-29
Crlf2	30	9.436852739	15.1910881	5.754235365	5.83E-30	1.01E-28
Hoxb13	25	20.15717521	11.82043415	-8.336741059	5.91E-30	1.02E-28
Map10	50	61.80939388	68.2563163	6.446922421	6.20E-30	1.07E-28
Mmp11	30	11.37965631	7.422650102	-3.95700621	6.35E-30	1.10E-28
Jakmip3	18	17.43364916	7.674054035	-9.759595124	1.03E-29	1.78E-28
Prrg1	20	23.66834016	13.65768782	-10.01065235	1.09E-29	1.88E-28
Col6a6	6	91.04038492	74.83438839	-16.20599653	1.13E-29	1.95E-28
Kank4	70	12.0356572	9.055158352	-2.980498852	1.21E-29	2.08E-28
4-Mar	17	11.74902737	3.648365713	-8.100661652	1.22E-29	2.09E-28
Gabra2	8	36.78135629	24.47183773	-12.30951856	1.28E-29	2.20E-28
Crmp1	144	54.44318574	58.75108747	4.307901733	1.44E-29	2.46E-28
Nos1	10	85.01572275	70.85109948	-14.16462328	1.51E-29	2.59E-28
Gprin2	48	20.98641311	29.08303768	8.096624568	1.64E-29	2.80E-28
Prss21	2	79.19254658	36.41328228	-42.7792643	2.02E-29	3.45E-28
Slc6a14	10	60.9377904	41.56448173	-19.37330867	2.13E-29	3.64E-28
Neto1	13	34.30464334	19.69427008	-14.61037326	2.51E-29	4.27E-28
Wscd1	33	41.56359467	52.54706902	10.98347435	2.55E-29	4.33E-28
Gm12992	78	65.08560729	70.24680855	5.161201253	2.63E-29	4.46E-28
Col23a1	82	6.709687211	5.268375111	-1.4413121	2.65E-29	4.50E-28
Sertad4	66	2.907915708	1.035974166	-1.871941543	3.13E-29	5.30E-28
Rph3a	15	19.98399519	11.2395908	-8.744404387	3.29E-29	5.58E-28
Prrt2	19	11.07484337	4.851884079	-6.222959292	4.02E-29	6.79E-28
Nkx1-1	86	44.02267539	39.64204127	-4.380634119	4.72E-29	7.97E-28
Pde4a	56	6.37663397	2.981187584	-3.395446385	4.76E-29	8.02E-28
Mir203	99	8.748211195	6.277635912	-2.470575282	5.05E-29	8.51E-28
St14	73	5.841646171	3.946666593	-1.894979579	5.57E-29	9.36E-28
Ovol1	108	12.42009375	10.78197435	-1.638119401	5.88E-29	9.88E-28
Snhg18	6	43.13130972	64.35714774	21.22583802	6.01E-29	1.01E-27
Icam1	8	19.72396581	7.258158953	-12.46580686	7.40E-29	1.24E-27
933432K03R	4	76.44101147	48.9981408	-27.44287067	8.28E-29	1.39E-27
Nkx2-5	12	23.76964808	10.30830595	-13.46134213	8.55E-29	1.43E-27

Gspt2	24	28.37544525	16.94850102	-11.42694423	1.00E-28	1.67E-27
Gprin1	130	4.250435506	3.000819458	-1.249616048	1.14E-28	1.90E-27
Gm15881	3	85.4016377	54.78156243	-30.62007526	1.23E-28	2.04E-27
Ano1	19	57.08206057	67.15372506	10.07166449	1.33E-28	2.22E-27
Gde1	51	6.064752814	4.206423353	-1.858329461	1.34E-28	2.24E-27
Tcf7l2	61	7.45115879	2.458351704	-4.992807086	1.42E-28	2.37E-27
Frmd5	55	7.601342031	4.159849626	-3.441492405	1.48E-28	2.46E-27
Barhl1	29	21.79734176	31.82249967	10.02515791	1.54E-28	2.56E-27
Slc30a3	57	9.822153791	13.3243723	3.502218509	1.56E-28	2.59E-27
Tagln3	19	46.1031336	57.91682658	11.81369298	1.62E-28	2.68E-27
Gm6307	11	30.54774924	20.01529076	-10.53245847	1.70E-28	2.81E-27
Rnf138rt1	16	42.64363409	56.11111807	13.46748397	1.71E-28	2.82E-27
Msrb3	70	4.677949875	2.290440312	-2.387509564	1.72E-28	2.84E-27
Gipc3	34	3.53110677	7.573067957	4.041961187	1.80E-28	2.96E-27
Enpp7	5	84.6425197	66.04232808	-18.60019162	1.92E-28	3.15E-27
Gja3	70	6.187545152	9.843103903	3.655558751	1.95E-28	3.20E-27
Ptpdc1	99	3.205671639	1.952346232	-1.253325407	2.00E-28	3.28E-27
Sdc2	8	11.13852428	4.530597761	-6.607926523	2.06E-28	3.38E-27
Slc2a12	24	25.29453833	18.32893051	-6.965607822	2.11E-28	3.46E-27
Pcsk5	46	31.68704934	27.35807945	-4.328969898	2.13E-28	3.49E-27
Tm6sf1	47	23.37650887	19.40566301	-3.970845861	2.34E-28	3.83E-27
)30419C18F	153	4.100171209	2.898987929	-1.20118328	2.41E-28	3.93E-27
Npy5r	17	53.10391377	63.8347718	10.73085803	2.51E-28	4.09E-27
Cdh4	53	55.30026259	60.6388893	5.338626707	2.84E-28	4.63E-27
Sh3rf3	85	16.78481771	13.71369861	-3.071119102	2.90E-28	4.71E-27
Trpc4	11	33.1660613	22.2802883	-10.885773	3.22E-28	5.24E-27
Mir7678	6	88.46525628	74.7765611	-13.68869518	3.31E-28	5.37E-27
Nlgn1	17	40.87374568	30.04300722	-10.83073846	3.41E-28	5.54E-27
430060I03R	2	89.93899937	59.14427899	-30.79472038	3.45E-28	5.60E-27
Ptger3	31	30.63495829	37.26204722	6.627088926	4.07E-28	6.58E-27
Nrn1	38	18.53350317	25.15215275	6.618649578	4.45E-28	7.19E-27
AI646519	12	16.71434328	30.42779746	13.71345418	4.46E-28	7.20E-27
Cplx1	47	22.67522523	30.63345848	7.95823325	4.48E-28	7.23E-27
Twist2	73	25.67295452	30.6192911	4.946336579	4.60E-28	7.41E-27

Cyp2r1	48	49.83813586	57.71295823	7.874822367	4.74E-28	7.63E-27
Galr1	8	28.38803777	12.09267787	-16.2953599	4.82E-28	7.74E-27
Mir375	26	16.81121498	26.75830048	9.947085501	5.75E-28	9.23E-27
Col16a1	7	19.98151567	7.655506652	-12.32600902	5.88E-28	9.43E-27
Gm715	45	31.69634149	24.59998138	-7.096360103	6.04E-28	9.67E-27
3-Sep	82	4.467062591	7.373756189	2.906693599	6.32E-28	1.01E-26
Stpg2	13	41.63454212	57.04652645	15.41198432	6.65E-28	1.06E-26
Cpxm2	33	35.09737661	30.26270947	-4.834667144	6.68E-28	1.07E-26
Adamts7	37	4.692133485	2.493907345	-2.19822614	7.09E-28	1.13E-26
Dysf	20	13.68864225	23.19096869	9.502326438	7.10E-28	1.13E-26
Adra1b	68	30.46158585	26.06276263	-4.398823223	7.16E-28	1.14E-26
Slco3a1	52	20.50968979	26.42905976	5.919369973	7.51E-28	1.19E-26
Gareml	57	17.51227625	13.11207696	-4.400199286	7.55E-28	1.20E-26
Mir5099	8	93.72833438	79.74221583	-13.98611856	8.28E-28	1.31E-26
i00014C23F	43	31.47660684	27.04358022	-4.433026618	8.74E-28	1.39E-26
Myo18b	8	90.32343078	77.22038035	-13.10305043	8.77E-28	1.39E-26
Calcrl	6	11.42448983	3.518443767	-7.906046065	9.26E-28	1.46E-26
Ntsr2	23	20.48206502	12.86254901	-7.619516007	9.75E-28	1.54E-26
Zfx	93	20.80715364	16.04723546	-4.759918175	1.08E-27	1.70E-26
Vsig8	2	95.29645191	62.27608008	-33.02037183	1.10E-27	1.73E-26
Asb18	3	76.75884924	53.22530069	-23.53354855	1.22E-27	1.91E-26
Itga9	84	5.450339952	3.332514661	-2.117825291	1.38E-27	2.17E-26
Zbtb16	55	21.42701631	16.51771573	-4.909300584	1.48E-27	2.32E-26
Cyp46a1	41	5.236460443	2.102978673	-3.13348177	1.49E-27	2.33E-26
Plcd1	54	31.81731949	36.97048404	5.15316455	1.48E-27	2.33E-26
Mrpl19	19	23.42065362	17.13868387	-6.281969756	1.82E-27	2.86E-26
'00072O05F	6	83.13814522	63.60312603	-19.53501918	1.89E-27	2.96E-26
Matn3	42	5.627623597	3.218017586	-2.409606012	2.53E-27	3.96E-26
Alox5	9	33.93215874	52.23349798	18.30133925	2.60E-27	4.05E-26
Serpine2	94	4.944582521	2.948317214	-1.996265307	2.60E-27	4.06E-26
Lrrn1	39	31.17968389	23.97048956	-7.209194332	2.82E-27	4.39E-26
Col6a5	11	28.03101701	17.28947335	-10.74154367	2.91E-27	4.52E-26
Plcl1	56	5.899092959	3.41098147	-2.488111489	3.06E-27	4.76E-26
Hspb9	22	31.87863375	41.86786541	9.989231655	3.48E-27	5.40E-26

Traf1	7	37.04816093	58.39108715	21.34292622	3.82E-27	5.93E-26
Ptprn2	56	17.09394603	22.96334188	5.869395853	4.42E-27	6.84E-26
St8sia2	54	42.38834244	48.6756706	6.287328166	4.81E-27	7.44E-26
Col7a1	12	33.65470308	11.87902164	-21.77568144	5.06E-27	7.83E-26
Klk8	11	18.82224711	4.25838937	-14.56385775	5.26E-27	8.13E-26
Wnt3a	72	9.825891825	7.161856391	-2.664035434	5.40E-27	8.34E-26
110032F04F	81	18.50422884	22.15655659	3.65232775	6.64E-27	1.02E-25
Pid1	65	25.70826323	21.36136737	-4.346895863	6.78E-27	1.04E-25
Grin3a	30	47.92061537	55.85219483	7.931579467	7.32E-27	1.13E-25
Ppp1r26	77	5.313052013	2.745208241	-2.567843772	7.70E-27	1.18E-25
Zfp783	60	52.48534211	58.07173947	5.586397355	7.74E-27	1.19E-25
Steap4	15	15.31605546	7.239154975	-8.076900482	8.02E-27	1.23E-25
Mcf2	20	34.69585315	21.05591618	-13.63993697	8.14E-27	1.25E-25
Hexa	45	3.222018217	1.467456986	-1.754561231	8.59E-27	1.31E-25
Acot12	33	16.38521619	11.10925369	-5.275962495	8.75E-27	1.34E-25
Asic2	122	52.2783573	56.66124182	4.38288452	9.08E-27	1.39E-25
Ctsz	21	4.677789778	1.462738998	-3.215050781	9.14E-27	1.39E-25
Daam2	9	19.91221734	31.33518193	11.42296459	1.15E-26	1.75E-25
Mlxipl	25	9.089280975	15.03608439	5.94680341	1.21E-26	1.84E-25
Sdsl	4	70.27411015	47.26677378	-23.00733637	1.31E-26	1.99E-25
Gm17762	24	6.989940917	1.139247105	-5.850693811	1.39E-26	2.11E-25
Tiam1	11	14.1934695	5.12220029	-9.071269212	1.39E-26	2.12E-25
Slc1a6	19	13.79171748	7.655161989	-6.136555493	1.51E-26	2.29E-25
'00018B24F	12	89.41780657	80.73422667	-8.683579897	1.54E-26	2.34E-25
Hpgd	9	38.8264525	26.0096864	-12.81676609	1.59E-26	2.41E-25
Sirpa	19	16.37981642	10.88565834	-5.494158081	1.61E-26	2.44E-25
Ago4	144	3.117605113	1.966798327	-1.150806787	1.80E-26	2.71E-25
'00019D03F	41	12.58770108	8.421148365	-4.166552711	1.84E-26	2.77E-25
Pacsin1	64	12.33544418	17.62814139	5.292697214	1.88E-26	2.84E-25
Speg	78	20.76968361	24.14058671	3.370903104	2.18E-26	3.28E-25
Unc80	68	24.50634903	19.8030149	-4.703334135	2.20E-26	3.30E-25
Bhlhe23	48	24.29832497	32.14938843	7.851063464	2.39E-26	3.59E-25
Rnf157	142	3.593105285	2.35504208	-1.238063205	2.48E-26	3.72E-25
Sema6a	18	9.944820362	3.082530793	-6.862289569	2.68E-26	4.01E-25

Gm11186	4	88.85017435	69.03420644	-19.81596791	3.34E-26	5.01E-25
Spint1	62	6.817482204	3.491823513	-3.32565869	3.45E-26	5.15E-25
Paqr6	10	66.8059701	55.89783913	-10.90813097	3.58E-26	5.34E-25
Slc6a5	27	37.35751331	46.30104421	8.943530901	3.64E-26	5.44E-25
Cd300a	6	52.35350875	34.771441	-17.58206775	3.66E-26	5.46E-25
Map7	168	5.682479585	3.628031284	-2.054448301	3.76E-26	5.59E-25
Galr2	7	28.14996932	47.36428856	19.21431924	4.14E-26	6.17E-25
Mamstr	13	72.54716463	53.98488031	-18.56228432	5.58E-26	8.30E-25
Dyx1c1	22	7.681421307	2.278434147	-5.402987159	6.15E-26	9.13E-25
Adgrg1	9	23.33179344	9.778132306	-13.55366113	6.26E-26	9.28E-25
Olfr522	4	75.51861489	49.79832953	-25.72028536	6.41E-26	9.50E-25
Arhgap25	5	63.7562724	31.23275691	-32.52351549	6.71E-26	9.94E-25
Kcp	30	7.272289779	3.548446448	-3.723843331	7.10E-26	1.05E-24
Tfap2c	94	45.60075916	51.95181716	6.351057998	7.26E-26	1.07E-24
Acss1	37	7.166837698	12.21407254	5.047234842	7.42E-26	1.10E-24
Topaz1	10	94.51935046	80.38167357	-14.13767689	7.56E-26	1.12E-24
Gm5712	3	81.64736763	54.17348751	-27.47388012	7.60E-26	1.12E-24
Cadps	41	5.354390634	2.438685607	-2.915705027	7.73E-26	1.14E-24
Ikzf3	45	31.27065368	40.12170313	8.85104945	8.23E-26	1.21E-24
Grm8	33	26.11939424	33.70714636	7.587752118	8.41E-26	1.24E-24
H2-Q2	21	13.98477222	10.21397805	-3.770794175	9.73E-26	1.43E-24
Synpo2l	6	91.76308126	77.47631362	-14.28676764	9.83E-26	1.44E-24
Hpn	33	51.53402719	44.56604261	-6.967984579	9.82E-26	1.44E-24
Olfr284	7	91.78605337	74.20501524	-17.58103813	9.86E-26	1.44E-24
Svip	33	6.053624848	3.453292013	-2.600332834	1.03E-25	1.50E-24
Mfsd6l	30	39.07483259	30.57111092	-8.503721671	1.05E-25	1.54E-24
Eif5a2	67	5.940517166	3.069033424	-2.871483742	1.05E-25	1.54E-24
Adap2	17	10.54924919	19.17992292	8.630673737	1.06E-25	1.55E-24
Pde8a	127	49.10448027	53.72926146	4.624781181	1.08E-25	1.58E-24
'00012B09F	12	55.09013039	38.09137369	-16.9987567	1.12E-25	1.63E-24
Calb2	26	55.05200674	62.63740876	7.585402014	1.16E-25	1.69E-24
Cnksr2	133	19.40037888	15.21417909	-4.186199788	1.19E-25	1.73E-24
)30020B18F	58	3.880054543	1.919764381	-1.960290162	1.21E-25	1.75E-24
Dnah9	32	22.24762696	14.49540227	-7.752224684	1.21E-25	1.76E-24

Dscaml1	32	7.414395964	13.05735206	5.642956099	1.24E-25	1.79E-24
333422C13F	23	14.50587699	8.656075323	-5.849801663	1.37E-25	1.98E-24
Umodl1	3	91.76315413	65.51616852	-26.24698561	1.52E-25	2.19E-24
Them7	17	28.78496873	19.33488392	-9.450084812	1.59E-25	2.29E-24
Adora1	21	7.528289886	3.379607729	-4.148682158	1.62E-25	2.34E-24
Gdf15	22	37.5580247	30.24880477	-7.309219929	1.71E-25	2.46E-24
Ccdc184	22	18.05600191	13.33394827	-4.722053636	1.76E-25	2.53E-24
Pygo1	108	10.38172742	7.931785612	-2.44994181	1.77E-25	2.54E-24
500029I14R	3	58.20896574	81.59515952	23.38619378	1.85E-25	2.65E-24
Gnal	96	8.366905101	12.33981181	3.972906707	1.87E-25	2.68E-24
)30423F21F	19	5.129075411	1.60078252	-3.528292891	1.99E-25	2.85E-24
Cyp2w1	9	79.54277672	65.31227063	-14.23050609	2.00E-25	2.86E-24
Tacc2	28	51.39370483	60.15572026	8.762015431	2.04E-25	2.91E-24
Def6	15	14.83631107	7.971694505	-6.864616562	2.45E-25	3.51E-24
Raet1a	18	7.893948623	3.787964741	-4.105983881	2.71E-25	3.87E-24
Meis1	46	15.85133147	11.94251583	-3.90881564	3.05E-25	4.34E-24
C1qtnf4	29	15.06831564	12.08118686	-2.987128787	3.05E-25	4.35E-24
Fign	92	3.369052557	1.721590162	-1.647462395	3.29E-25	4.68E-24
900005J15R	18	19.56878305	11.34996494	-8.218818118	3.66E-25	5.20E-24
Sox12	128	3.892239968	6.877594278	2.985354309	3.83E-25	5.44E-24
Ppm1e	72	3.578766808	1.702776773	-1.875990035	3.91E-25	5.55E-24
700015E13F	5	91.35574274	63.45966034	-27.8960824	4.19E-25	5.94E-24
532428N05F	22	23.67853178	32.73757549	9.059043717	4.28E-25	6.06E-24
Rundc3b	42	12.30480583	8.158451897	-4.146353936	4.29E-25	6.08E-24
Gm5144	17	5.906861502	11.61045966	5.703598159	4.65E-25	6.57E-24
Dmkn	5	70.42903107	44.65462468	-25.77440639	4.67E-25	6.59E-24
Siah3	7	19.7827125	43.33877624	23.55606374	5.28E-25	7.45E-24
Arhgef10l	51	14.06665917	9.289956445	-4.776702724	5.45E-25	7.69E-24
Actl6b	28	14.22095542	22.73255614	8.511600712	5.77E-25	8.12E-24
Sppl2c	9	78.11767181	65.73245249	-12.38521932	6.29E-25	8.85E-24
Wdr72	7	41.13914552	21.61495247	-19.52419305	6.69E-25	9.41E-24
Kif19a	26	12.651656	19.54527687	6.893620869	7.24E-25	1.02E-23
Rnf150	19	5.121239026	1.941746444	-3.179492583	7.34E-25	1.03E-23
Papolb	55	78.76592414	73.29040364	-5.4755205	7.53E-25	1.06E-23

Zfp365	53	11.15669994	7.63513099	-3.521568953	7.55E-25	1.06E-23
Gm9895	6	32.78487888	56.24636455	23.46148566	7.69E-25	1.08E-23
)C10086161	7	37.32253785	23.89258106	-13.42995679	8.12E-25	1.13E-23
Gpm6b	30	24.75076279	15.38966859	-9.361094207	8.21E-25	1.15E-23
Ska1	9	7.342929126	2.155811042	-5.187118084	8.27E-25	1.15E-23
Nell2	100	61.41715467	64.97727813	3.560123452	9.07E-25	1.26E-23
Mical1	31	20.13240053	15.5198647	-4.612535831	9.67E-25	1.35E-23
Stk32b	20	34.30893216	25.58327876	-8.725653393	9.83E-25	1.37E-23
Ascl4	42	3.843751935	1.76168302	-2.082068915	1.19E-24	1.65E-23
;10307P16F	4	30.61968569	13.69276094	-16.92692475	1.19E-24	1.65E-23
210019I11R	25	30.23414403	22.62502837	-7.609115664	1.34E-24	1.86E-23
Chst1	48	5.311752894	2.552884266	-2.758868628	1.34E-24	1.86E-23
Lhx1os	30	45.68804494	55.85772832	10.16968338	1.41E-24	1.95E-23
430019H16F	11	45.61723945	33.6958724	-11.92136705	1.41E-24	1.95E-23
Prr7	72	4.488630409	2.49904305	-1.989587359	1.52E-24	2.10E-23
Tnfsfm13	19	5.904714145	2.016376323	-3.888337823	1.58E-24	2.18E-23
Tnfsf12	19	5.904714145	2.016376323	-3.888337823	1.58E-24	2.18E-23
Rspo4	46	17.5931637	14.60403924	-2.989124458	1.73E-24	2.39E-23
Adamtsl3	51	42.43405254	35.7239049	-6.710147642	2.02E-24	2.78E-23
Rwdd2a	18	16.9930937	7.062786466	-9.930307238	2.07E-24	2.85E-23
Atp2c2	18	60.15208439	69.8796068	9.727522411	2.22E-24	3.05E-23
Cpq	15	9.090232319	3.660400231	-5.429832088	2.70E-24	3.71E-23
Antxr1	36	3.660500862	1.867505747	-1.792995115	3.06E-24	4.19E-23
Wnt5b	121	8.855742004	10.87923781	2.02349581	3.11E-24	4.26E-23
Hoxa11	40	38.50247369	35.23025337	-3.272220317	3.51E-24	4.81E-23
Ptchd2	19	50.77402114	39.61453638	-11.15948476	3.92E-24	5.36E-23
Zswim5	146	3.654720042	2.40195292	-1.252767122	5.21E-24	7.12E-23
;30058N18F	36	46.22753886	53.41029755	7.182758683	5.48E-24	7.48E-23
Pcdhac2	32	49.92027173	58.79099071	8.870718981	5.80E-24	7.92E-23
Ret	15	4.721635795	1.568079502	-3.153556292	6.12E-24	8.33E-23
Egr3	28	17.25286427	12.31974564	-4.93311863	6.49E-24	8.84E-23
Efs	43	11.42064707	9.128529852	-2.292117222	6.74E-24	9.16E-23
Elovl2	85	36.95640425	42.25775904	5.301354789	7.28E-24	9.89E-23
Foxp2	34	14.76826368	10.16950318	-4.598760497	7.72E-24	1.05E-22

Ggt7	26	8.23256881	15.94009721	7.707528405	8.01E-24	1.09E-22
Atp2b3	22	26.73031654	18.21734566	-8.51297088	8.28E-24	1.12E-22
Crb2	22	48.49279829	57.27750739	8.784709103	8.42E-24	1.14E-22
Gm19557	9	86.72277484	72.48087863	-14.24189621	8.73E-24	1.18E-22
Pax1	14	32.6790894	47.29921033	14.62012093	8.77E-24	1.19E-22
Il12rb2	10	20.20178831	12.42824287	-7.773545437	9.00E-24	1.22E-22
[illegible]30417C22[illegible]	11	12.2244469	24.31196141	12.08751451	9.14E-24	1.23E-22
Gm9767	71	14.18554268	12.50438176	-1.68116092	9.57E-24	1.29E-22
Gem	20	5.948522566	1.495985686	-4.45253688	9.73E-24	1.31E-22
Inhbe	5	76.37726824	59.33914103	-17.03812721	9.90E-24	1.33E-22
Aldh1l1	15	53.61389993	38.05860118	-15.55529875	1.05E-23	1.41E-22
Zfp2	19	7.477744104	3.173368963	-4.304375141	1.05E-23	1.41E-22
Fstl3	34	6.125633885	2.911633302	-3.214000583	1.07E-23	1.44E-22
Hs3st4	123	56.50489334	60.22790028	3.723006935	1.37E-23	1.84E-22
Myo18a	11	52.83008616	43.62233882	-9.207747342	1.47E-23	1.96E-22
Speer2	2	78.125	40.61085973	-37.51414027	1.48E-23	1.97E-22
Cybrd1	41	8.585831454	5.531656389	-3.054175065	1.97E-23	2.63E-22
Serpinb9c	5	70.81639541	52.50256021	-18.3138352	2.00E-23	2.67E-22
Sncg	8	44.36590913	30.87107262	-13.49483651	2.00E-23	2.67E-22
Itga11	4	22.30207919	6.548081548	-15.75399764	2.10E-23	2.80E-22
Fgd1	49	23.74233438	16.98820532	-6.754129061	2.17E-23	2.88E-22
Znrf1	100	4.985072763	2.760848934	-2.224223829	2.28E-23	3.03E-22
Haus7	2	67.54844961	29.08653846	-38.46191115	2.29E-23	3.05E-22
F7	6	58.27423614	43.04779582	-15.22644032	2.55E-23	3.39E-22
Adam12	44	4.2829388	1.87452942	-2.408409379	2.89E-23	3.83E-22
Cox6a2	6	80.71207528	66.11017417	-14.60190111	2.89E-23	3.83E-22
Slc6a1	27	32.99461816	41.36297386	8.368355695	2.90E-23	3.84E-22
Mir196a-2	16	20.94858505	10.49546911	-10.45311594	2.95E-23	3.91E-22
Elavl3	54	21.57046268	26.48781397	4.91735129	2.99E-23	3.95E-22
Pou4f2	72	25.36368277	30.60044758	5.236764809	3.10E-23	4.09E-22
Arx	15	53.77900097	67.89877494	14.11977396	3.26E-23	4.30E-22
Lrrc10b	53	3.700927792	1.832243671	-1.868684121	3.29E-23	4.34E-22
Sfrp2	59	22.38412398	19.97396604	-2.410157941	3.57E-23	4.70E-22
Atp1b2	6	11.13184791	2.656977772	-8.474870134	3.66E-23	4.82E-22

Trp53i11	61	15.32029449	12.83213286	-2.488161632	3.85E-23	5.06E-22
210039B01F	10	51.4120288	37.62252098	-13.78950782	4.51E-23	5.93E-22
Sgcd	3	32.00784078	7.938285828	-24.06955495	4.64E-23	6.09E-22
Dtx1	46	34.6592098	42.11044481	7.451235011	4.97E-23	6.52E-22
Fgf22	19	27.14254864	35.09408256	7.951533919	5.10E-23	6.68E-22
Mir3962	11	61.68479049	49.18958865	-12.49520184	5.49E-23	7.19E-22
Mir6954	3	91.03420201	64.56010396	-26.47409806	5.51E-23	7.20E-22
Rimklb	97	31.78193421	35.78950335	4.007569141	5.65E-23	7.38E-22
Bdh1	39	6.738114182	11.48873137	4.750617183	5.72E-23	7.47E-22
230077H06F	58	45.34354982	50.63121391	5.287664083	5.85E-23	7.63E-22
Mpped1	168	57.29281771	53.96398545	-3.328832266	6.33E-23	8.25E-22
Rhcg	12	19.95935326	27.88415081	7.924797547	6.54E-23	8.52E-22
Arid3c	35	5.591851096	2.529261588	-3.062589508	7.33E-23	9.54E-22
Taf9b	43	21.08662992	15.11090884	-5.975721086	7.87E-23	1.02E-21
Eomes	15	10.90136838	5.218992466	-5.682375918	9.31E-23	1.21E-21
230050P20F	27	16.45203953	12.58138939	-3.870650132	1.02E-22	1.32E-21
Aim1l	5	82.98925536	67.22706866	-15.7621867	1.03E-22	1.34E-21
Adcyap1r1	24	23.85521035	19.90668477	-3.94852558	1.05E-22	1.35E-21
Zfp583	14	46.29766974	58.38892245	12.09125271	1.30E-22	1.68E-21
Foxi1	7	88.74121203	75.43261801	-13.30859401	1.41E-22	1.82E-21
Ankrd63	33	7.306451897	4.082831265	-3.223620632	1.41E-22	1.82E-21
Kcnip4	8	23.50848322	11.11965276	-12.38883046	1.66E-22	2.14E-21
Vgll3	67	16.8151459	14.15393065	-2.661215254	1.67E-22	2.15E-21
Lppr3	42	3.219422461	6.217962738	2.998540277	1.71E-22	2.21E-21
Zcchc12	5	53.21548822	29.08747173	-24.12801648	1.89E-22	2.43E-21
Isl2	19	10.0796983	16.36390378	6.284205477	1.90E-22	2.45E-21
530403H02F	3	66.45651511	44.47256452	-21.98395059	1.91E-22	2.45E-21
Acp5	25	32.36563835	25.81391468	-6.551723668	1.96E-22	2.51E-21
Wnt9a	83	2.414300131	0.849102954	-1.565197176	2.17E-22	2.79E-21
Dmrta2	66	36.71128536	31.90338504	-4.807900327	2.18E-22	2.80E-21
Dnmt3b	73	2.216057647	0.967662123	-1.248395524	2.22E-22	2.84E-21
Prss50	8	37.78690613	26.50377647	-11.28312966	2.42E-22	3.10E-21
Lrrc26	18	5.781772896	1.828178489	-3.953594407	3.15E-22	4.03E-21
Gprc5c	74	4.241999118	2.261933853	-1.980065265	3.29E-22	4.20E-21

Mmd2	16	12.44281771	21.16984772	8.727030011	3.36E-22	4.29E-21
Reck	84	4.469635256	2.804344979	-1.665290277	3.42E-22	4.35E-21
Slc37a1	65	4.238330886	1.893807928	-2.344522958	3.54E-22	4.51E-21
Chga	26	32.4437768	39.44120657	6.99742977	3.58E-22	4.56E-21
C77370	75	31.42047027	26.53242613	-4.888044135	3.60E-22	4.58E-21
Mir9-3	69	43.84048634	49.35057684	5.510090503	4.16E-22	5.29E-21
Tmem221	50	11.45926963	8.21112582	-3.248143807	4.47E-22	5.68E-21
Gsc2	51	22.84770864	18.02514562	-4.822563024	4.58E-22	5.81E-21
Hoxc13	58	30.13216739	26.1430557	-3.989111695	4.71E-22	5.97E-21
Tll1	71	10.21272853	7.868439356	-2.344289171	4.83E-22	6.12E-21
Fut9	28	43.01172337	35.51375649	-7.497966881	4.92E-22	6.22E-21
Gpr153	87	6.158947289	4.702895735	-1.456051554	6.15E-22	7.78E-21
Lhfp	33	7.121968894	3.149378203	-3.972590691	7.19E-22	9.07E-21
Vma21	16	27.312943	15.01529923	-12.29764377	7.72E-22	9.74E-21
Gm17359	8	71.18458753	53.51946475	-17.66512278	8.73E-22	1.10E-20
Bglap3	5	80.19309908	63.9385842	-16.25451488	8.90E-22	1.12E-20
Adam19	26	17.15072512	23.2031784	6.052453285	9.12E-22	1.15E-20
Sst	9	13.72356956	6.14759354	-7.575976021	9.33E-22	1.17E-20
Gm38402	3	81.655585	60.38643385	-21.26915115	9.36E-22	1.18E-20
Phf24	86	8.728319913	7.975944627	-0.752375285	9.45E-22	1.19E-20
Chst7	53	27.80325101	23.08574008	-4.717510927	9.55E-22	1.20E-20
Tanc2	96	5.049135816	3.861561954	-1.187573863	1.22E-21	1.53E-20
Pantr1	14	46.4295811	62.94042822	16.51084712	1.23E-21	1.54E-20
Ppp1r14c	13	28.36317353	13.92975461	-14.43341892	1.29E-21	1.61E-20
Zfp105	24	39.86463309	34.07894374	-5.785689354	1.30E-21	1.63E-20
Mir6396	5	89.22945285	73.66769277	-15.56176008	1.31E-21	1.63E-20
Chrna6	4	48.49204259	29.57071868	-18.92132391	1.31E-21	1.63E-20
Cd22	2	83.71021399	56.34001637	-27.37019762	1.31E-21	1.64E-20
Frmd7	5	73.17279025	55.68909681	-17.48369344	1.33E-21	1.66E-20
Skor1	107	11.45247051	10.51495019	-0.937520322	1.39E-21	1.73E-20
Fgfr1	54	10.02861889	14.83221396	4.803595071	1.40E-21	1.74E-20
Scube1	93	21.42892313	25.15948467	3.730561539	1.43E-21	1.78E-20
Kcnk1	29	7.06137825	4.286087323	-2.775290927	1.43E-21	1.78E-20
Nfam1	10	51.31958035	39.81065496	-11.50892539	1.55E-21	1.92E-20

Insm1	126	8.269286718	5.595971491	-2.673315227	1.60E-21	1.98E-20
Cacna1d	136	20.27114238	18.79438008	-1.4767623	1.70E-21	2.10E-20
Hhipl1	52	5.836587695	3.717853457	-2.118734238	1.76E-21	2.18E-20
Tmcc2	66	2.374481966	1.256083277	-1.118398688	1.96E-21	2.42E-20
Synpo	38	7.512191656	3.4495782	-4.062613456	2.30E-21	2.84E-20
Epha5	31	21.49109032	17.06459539	-4.426494928	2.34E-21	2.89E-20
Shc2	94	3.057280149	1.669853484	-1.387426665	2.49E-21	3.07E-20
Hhla1	3	59.04104763	34.55800748	-24.48304015	2.69E-21	3.31E-20
Bdkrb2	12	13.12820713	4.748605146	-8.379601979	2.75E-21	3.39E-20
230112J18R	5	50.44054825	29.74034815	-20.7002001	3.02E-21	3.71E-20
Gm14139	5	83.74069638	67.65269306	-16.08800332	3.11E-21	3.83E-20
Bcl2l10	17	94.85346155	87.97004916	-6.883412389	3.50E-21	4.30E-20
Cds1	119	3.354164399	2.384367589	-0.96979681	3.75E-21	4.60E-20
Tab3	68	25.15788405	19.99739268	-5.160491364	3.84E-21	4.70E-20
Gabra5	84	54.63034831	58.54388197	3.91353366	4.06E-21	4.97E-20
Hopx	66	14.13677452	13.30366943	-0.833105081	4.09E-21	5.00E-20
Gng7	7	86.46880005	70.42481981	-16.04398024	4.28E-21	5.24E-20
Coro2b	59	6.862532007	10.21114313	3.348611119	4.30E-21	5.25E-20
Gstm4	17	6.445926532	2.282645922	-4.16328061	4.72E-21	5.76E-20
Fancd2os	12	72.67410517	79.48817745	6.814072273	5.18E-21	6.32E-20
Slc22a3	33	30.40530288	25.95678528	-4.448517607	5.74E-21	7.00E-20
Trim58	18	31.73069609	22.64951859	-9.081177494	5.80E-21	7.06E-20
Lrrc9	5	29.97490321	16.9288196	-13.04608361	6.50E-21	7.90E-20
Bcl11b	69	9.762975468	12.90291484	3.139939367	6.78E-21	8.24E-20
Gm16596	58	2.101810468	1.032493192	-1.069317276	7.33E-21	8.90E-20
Lhfpl5	25	11.11812806	17.2255308	6.107402741	7.42E-21	9.00E-20
Ammecr1	77	21.22995501	16.24487692	-4.985078089	7.62E-21	9.24E-20
Creg2	35	11.10375185	16.35311289	5.249361037	7.64E-21	9.25E-20
Gprin3	8	10.40139072	5.245993593	-5.15539713	7.64E-21	9.25E-20
Foxg1	41	4.242156193	2.154826694	-2.087329499	8.49E-21	1.03E-19
B3gnt3	14	11.68950331	6.055669874	-5.633833434	8.93E-21	1.08E-19
Auts2	31	54.37283843	62.63343764	8.260599207	8.98E-21	1.08E-19
Zfp819	21	12.13215217	7.165795486	-4.966356687	1.08E-20	1.31E-19
Hcar1	9	46.26897193	33.93219526	-12.33677667	1.14E-20	1.37E-19

310429I04R	13	20.07740194	12.01310056	-8.064301374	1.18E-20	1.42E-19
Cand2	15	21.34170722	16.4549455	-4.886761722	1.20E-20	1.44E-19
Tmem164	66	15.24045862	10.89656839	-4.343890229	1.25E-20	1.50E-19
Mmrn2	9	49.21897237	37.36544104	-11.85353133	1.29E-20	1.55E-19
Rasl2-9	35	38.60087757	31.95323537	-6.647642199	1.32E-20	1.58E-19
Akap5	42	28.8906149	34.32418536	5.433570462	1.43E-20	1.71E-19
Slc16a12	41	15.33335697	11.51791739	-3.815439576	1.50E-20	1.80E-19
Ppp1r16b	34	17.09456846	13.6518028	-3.442765664	1.60E-20	1.92E-19
Kcna2	76	23.31974081	27.19533464	3.87559383	1.69E-20	2.03E-19
Gyltl1b	31	7.132665492	11.25310085	4.120435353	1.70E-20	2.04E-19
Prickle3	22	29.42072836	19.47698594	-9.943742427	1.77E-20	2.12E-19
Ppp1r3f	43	21.19069603	14.76208194	-6.428614089	1.93E-20	2.30E-19
Mum1l1	10	31.14244374	15.59483934	-15.5476044	2.04E-20	2.43E-19
Mir6392	40	6.392464764	2.93009729	-3.462367473	2.04E-20	2.43E-19
Kcnt1	46	17.73142074	13.5680011	-4.163419644	2.09E-20	2.49E-19
Padi1	5	52.31481481	24.97915162	-27.33566319	2.09E-20	2.49E-19
30021M05I	23	36.82660918	45.33605318	8.509443995	2.10E-20	2.49E-19
Htr3b	4	39.64667688	22.1951795	-17.45149738	2.10E-20	2.49E-19
Gm7271	4	75.70332343	58.57278975	-17.13053369	2.21E-20	2.62E-19
Scnn1b	10	21.92606538	13.24462164	-8.681443747	2.50E-20	2.96E-19
Avpr1a	26	21.34555537	31.44177182	10.09621645	2.54E-20	3.01E-19
230079O03F	6	86.47735899	74.36332135	-12.11403763	2.58E-20	3.05E-19
Vmn2r29	23	70.47568137	63.10457493	-7.371106437	2.71E-20	3.21E-19
Bst2	20	5.811733957	1.437971401	-4.373762557	2.73E-20	3.22E-19
Vstm2l	81	40.48811565	45.00206209	4.513946442	3.13E-20	3.70E-19
Eif4e3	64	2.943590019	1.382938484	-1.560651534	3.28E-20	3.88E-19
Plbd1	28	20.26170643	14.60275544	-5.658950989	3.34E-20	3.94E-19
Mospd4	35	19.92807011	27.58278328	7.654713169	3.66E-20	4.32E-19
Ms4a7	2	74.1300813	38.12947242	-36.00060888	4.24E-20	4.99E-19
Gm13752	4	79.64038945	55.9133763	-23.72701315	4.34E-20	5.11E-19
Tgfb2	18	11.35592964	6.410932277	-4.944997365	4.80E-20	5.64E-19
Gpr6	11	40.65963635	27.17214797	-13.48748838	5.15E-20	6.05E-19
732456N10F	5	94.02513818	83.02760865	-10.99752954	5.20E-20	6.10E-19
Mir5126	46	32.72195487	28.41945029	-4.30250458	5.47E-20	6.42E-19

Abca13	5	75.51381424	57.93869103	-17.57512321	5.56E-20	6.51E-19
BC051628	7	8.389795592	1.09966238	-7.290133213	5.92E-20	6.94E-19
Cdh22	100	6.539906464	5.781966261	-0.757940203	6.74E-20	7.89E-19
Necab2	93	16.38665064	19.65136006	3.264709419	6.79E-20	7.94E-19
Cstad	17	26.99324638	21.63939671	-5.353849667	7.27E-20	8.49E-19
Mettl24	42	11.96003799	8.684923364	-3.275114625	7.29E-20	8.51E-19
510035F20R	57	4.068389573	2.156905935	-1.911483639	8.89E-20	1.04E-18
Spag16	24	61.41976169	68.59094816	7.171186464	8.91E-20	1.04E-18
Tbx15	72	2.739017355	1.714812466	-1.024204889	9.05E-20	1.05E-18
Rps6	60	3.936748213	6.740896478	2.804148265	1.13E-19	1.31E-18
Tbx1	70	27.22185208	24.17985279	-3.04199929	1.30E-19	1.51E-18
Kcnk12	77	32.20065731	37.54234145	5.341684148	1.33E-19	1.55E-18
Podxl2	41	11.32697315	8.751809337	-2.575163814	1.47E-19	1.71E-18
Slc17a7	35	12.45254701	10.30105494	-2.151492072	1.54E-19	1.78E-18
Kcnmb1	9	55.04550854	38.44009815	-16.60541039	1.57E-19	1.82E-18
Dtna	52	13.32295282	17.15279557	3.829842744	1.63E-19	1.89E-18
Nrcam	77	14.38809119	17.72300802	3.334916833	1.78E-19	2.06E-18
Megf6	20	4.266807125	1.628491731	-2.638315394	1.78E-19	2.06E-18
930533P14R	6	87.6803301	68.1425383	-19.5377918	1.85E-19	2.14E-18
Cntnap1	15	15.13713354	5.576933071	-9.560200468	1.88E-19	2.17E-18
Dnah1	15	7.630591892	2.851944012	-4.77864788	1.90E-19	2.19E-18
Cgref1	19	14.23776359	9.477595069	-4.760168519	1.94E-19	2.24E-18
Syt7	97	2.075423185	1.292634072	-0.782789113	1.97E-19	2.26E-18
Timm8a2	4	97.63120393	86.7854746	-10.84572932	2.17E-19	2.49E-18
Cnbd2	89	3.325666877	1.608702558	-1.716964319	2.17E-19	2.50E-18
Sntg1	23	46.39232333	54.16444518	7.772121857	2.19E-19	2.51E-18
Pou4f3	10	21.21956111	12.62441422	-8.595146883	2.25E-19	2.58E-18
Fam131c	40	3.592368845	1.772630045	-1.819738799	2.27E-19	2.60E-18
Gm3558	3	25.09121061	46.64464309	21.55343247	2.44E-19	2.80E-18
Apba3	13	7.153065469	2.978268196	-4.174797272	2.49E-19	2.85E-18
Sdr16c5	4	72.7422322	54.94052614	-17.80170606	2.55E-19	2.92E-18
Robo1	30	29.59859547	24.36164014	-5.236955333	2.56E-19	2.92E-18
Gstp2	5	80.96345966	63.39720491	-17.56625475	2.70E-19	3.08E-18
Foxn4	48	16.65794753	21.7151685	5.057220977	2.87E-19	3.28E-18

Tead2	25	7.354644488	3.705383696	-3.649260793	2.88E-19	3.29E-18
200002D01F	25	25.89031926	19.34596345	-6.544355814	2.91E-19	3.32E-18
Pnpla3	38	7.247959002	3.656097026	-3.591861976	3.17E-19	3.61E-18
Dmrtb1	25	88.00769103	82.55654119	-5.451149838	3.32E-19	3.78E-18
Fgf11	23	19.45430698	14.20299414	-5.251312831	3.54E-19	4.03E-18
Onecut1	37	15.83258272	11.66372646	-4.168856255	3.71E-19	4.22E-18
Gata2	51	11.16010265	7.084441937	-4.075660717	3.79E-19	4.30E-18
Adgra1	18	6.468869737	2.68004828	-3.788821457	4.00E-19	4.54E-18
Slc7a2	11	10.33241007	4.762268703	-5.570141371	4.04E-19	4.58E-18
Fli1	30	8.972896478	15.2067698	6.23387332	4.17E-19	4.73E-18
Slc12a5	48	22.00848092	28.09604788	6.087566958	4.35E-19	4.92E-18
Smagp	68	5.177305392	3.800166208	-1.377139184	4.34E-19	4.92E-18
Bik	50	26.89595405	31.55849679	4.662542738	4.84E-19	5.47E-18
P4htm	39	8.469674107	5.571817899	-2.897856208	5.21E-19	5.89E-18
Mir7240	24	18.10073476	10.35876918	-7.741965579	5.50E-19	6.20E-18
Tuba3a	15	93.77073516	82.44868762	-11.32204754	5.53E-19	6.23E-18
Pnma2	80	36.42008039	33.29224699	-3.127833406	5.57E-19	6.27E-18
Plk5	68	10.7537281	8.649354774	-2.104373328	6.21E-19	6.99E-18
Cst6	12	5.631312636	1.011804799	-4.619507837	6.46E-19	7.27E-18
Rprl2	2	89.99603489	67.59836698	-22.39766791	6.73E-19	7.57E-18
Gna14	11	9.893360948	3.362744472	-6.530616476	6.81E-19	7.66E-18
Fam198b	4	28.23518755	14.40849373	-13.82669383	7.14E-19	8.02E-18
930520O04F	45	7.125188449	3.979545918	-3.145642531	7.28E-19	8.17E-18
Clcn5	41	18.3992848	13.05259077	-5.34669403	7.34E-19	8.23E-18
Gm960	13	4.770349913	0.91333577	-3.857014143	7.40E-19	8.29E-18
Shisa6	156	24.53194896	27.75340673	3.221457775	7.75E-19	8.67E-18
330158H04F	14	42.87907986	35.82987157	-7.049208287	7.80E-19	8.72E-18
Cnih3	43	6.035133288	2.481429578	-3.55370371	7.85E-19	8.76E-18
Cyb5r2	11	11.71061928	5.368542158	-6.342077124	7.85E-19	8.76E-18
Slc43a3	16	38.0581557	32.01074779	-6.047407906	7.97E-19	8.89E-18
Abcg4	47	6.173353121	4.099299584	-2.074053536	8.04E-19	8.96E-18
Tcl1	4	91.08743077	72.49760399	-18.58982678	8.19E-19	9.12E-18
Palm2	25	13.38775857	19.18963731	5.801878745	8.23E-19	9.16E-18
Pakap	25	13.38775857	19.18963731	5.801878745	8.23E-19	9.16E-18

Cd200r3	2	92.48177447	71.96947979	-20.51229468	8.40E-19	9.34E-18
Hcrtr1	46	9.371507404	14.2191918	4.847684396	8.49E-19	9.43E-18
Asb10	2	91.59820031	43.22836662	-48.3698337	8.63E-19	9.57E-18
Lypd3	10	59.33496731	47.28519292	-12.04977439	9.28E-19	1.03E-17
Cthrc1	4	24.92869556	43.91921476	18.9905192	9.61E-19	1.06E-17
Mir330	8	8.583029905	1.996338841	-6.586691064	1.07E-18	1.19E-17
AU022751	29	88.93555914	83.25808659	-5.67747255	1.11E-18	1.23E-17
Chst3	64	10.77136958	7.898807228	-2.872562355	1.13E-18	1.25E-17
Nbl1	99	10.68065576	8.367033779	-2.313621984	1.15E-18	1.27E-17
Mmp25	28	14.5569376	21.61927591	7.06233831	1.17E-18	1.29E-17
Tnfrsf8	12	25.95598351	15.96968957	-9.986293933	1.22E-18	1.35E-17
Madcam1	12	66.55674953	54.7048384	-11.85191113	1.25E-18	1.38E-17
H1fnt	8	72.9764633	57.89587189	-15.08059141	1.33E-18	1.46E-17
Ptgis	33	23.3248839	29.40696533	6.082081423	1.60E-18	1.76E-17
Armc12	6	81.72505315	66.41161758	-15.31343557	1.69E-18	1.86E-17
Ccdc3	35	5.84659226	3.56240527	-2.28418699	1.73E-18	1.90E-17
Suv39h1	89	19.84401913	15.21117328	-4.632845851	1.79E-18	1.97E-17
Lrfn2	6	22.80904691	13.77781478	-9.031232135	1.82E-18	2.00E-17
Prr15	5	25.86312568	12.91919393	-12.94393175	1.91E-18	2.09E-17
Kcnip3	40	20.63069046	16.73986516	-3.890825304	1.97E-18	2.16E-17
Mxra8	7	46.15339127	30.64012445	-15.51326682	2.04E-18	2.24E-17
Nrgn	6	3.851218157	20.27940463	16.42818647	2.08E-18	2.28E-17
.30074G19F	68	2.640635221	1.539767739	-1.100867481	2.11E-18	2.30E-17
Mpl	2	51.32985941	21.42071604	-29.90914337	2.47E-18	2.70E-17
Cntn2	23	23.11350498	15.53824626	-7.575258715	2.53E-18	2.76E-17
Slc2a10	6	22.33632402	13.01855538	-9.317768639	2.64E-18	2.88E-17
Sobp	87	4.824571708	3.105234987	-1.719336721	2.91E-18	3.17E-17
Fhl1	43	29.48599488	23.21735283	-6.268642053	3.16E-18	3.44E-17
Efnb3	47	54.22278712	59.40174688	5.178959765	3.18E-18	3.46E-17
Arhgef18	22	26.29305993	19.98289163	-6.310168299	3.50E-18	3.81E-17
Krt4	6	93.4123801	79.08868302	-14.32369709	3.52E-18	3.83E-17
Stag2	74	24.53790761	18.98364179	-5.554265822	3.55E-18	3.86E-17
Dgkg	35	22.61139559	31.03255765	8.421162054	3.98E-18	4.32E-17
Xkr5	7	4.963003254	13.91897695	8.955973696	4.14E-18	4.49E-17

Lemd1	68	8.446764761	6.961486176	-1.485278585	4.19E-18	4.54E-17
Pex5l	116	61.2394059	64.02706962	2.78766372	4.27E-18	4.63E-17
Tiam2	36	4.862616486	2.018461977	-2.844154509	4.56E-18	4.93E-17
930451I11R	7	27.0345668	14.31262127	-12.72194553	4.89E-18	5.28E-17
Fkbp6	14	90.23669473	78.90314579	-11.33354894	5.01E-18	5.42E-17
Cacng2	64	29.80092098	34.382833	4.581912017	5.16E-18	5.57E-17
Thbs4	31	19.65933242	26.72426835	7.064935931	5.46E-18	5.89E-17
Slc15a1	18	21.40311719	13.80567973	-7.597437463	5.73E-18	6.18E-17
Cdx1	69	69.80798691	74.20949638	4.40150947	5.90E-18	6.36E-17
Lhfpl3	54	23.49656518	19.15640708	-4.340158103	6.28E-18	6.76E-17
Dyrk4	4	22.27038872	10.69715565	-11.57323307	6.83E-18	7.35E-17
Mir181d	3	93.12523911	78.00396908	-15.12127003	7.21E-18	7.75E-17
H60c	5	77.02990333	62.03424849	-14.99565484	7.35E-18	7.89E-17
BC051537	6	72.68645772	58.82906747	-13.85739025	7.90E-18	8.48E-17
Slit1	37	29.70951306	36.17567543	6.466162371	8.07E-18	8.66E-17
Pnma1	54	46.46166111	51.90143102	5.439769913	8.18E-18	8.77E-17
Tmem200c	30	44.04821956	54.65940815	10.61118858	8.84E-18	9.47E-17
Ppp1r3d	60	55.20635471	60.14021557	4.933860859	9.65E-18	1.03E-16
Ephx1	2	6.265011741	21.06227106	14.79725932	1.00E-17	1.07E-16
Ehd3	30	32.67762351	38.82500985	6.147386342	1.01E-17	1.08E-16
Gm684	28	13.83962144	8.464311326	-5.375310112	1.09E-17	1.17E-16
Wnt9b	42	14.81131307	12.0051799	-2.806133165	1.11E-17	1.18E-16
Tceal3	10	39.92462418	23.52717862	-16.39744556	1.15E-17	1.22E-16
Il3	5	80.19278976	64.47461036	-15.7181794	1.15E-17	1.22E-16
Krt85	11	92.52118863	80.39060304	-12.13058559	1.18E-17	1.26E-16
Mchr1	18	15.82272735	10.37196005	-5.450767302	1.48E-17	1.58E-16
Gpr45	31	15.54288847	9.923658147	-5.619230324	1.51E-17	1.61E-16
530402F18F	69	7.219869682	5.903190796	-1.316678886	1.52E-17	1.62E-16
Podn	67	9.603929017	7.812822525	-1.791106492	1.54E-17	1.64E-16
Inhbb	38	6.428962477	3.462072394	-2.966890083	1.59E-17	1.68E-16
Ikzf4	8	13.14028513	25.91160221	12.77131708	1.67E-17	1.77E-16
Gm14812	7	70.59745279	51.05920997	-19.53824283	1.81E-17	1.92E-16
Pyroxd2	12	12.67857803	7.073922719	-5.604655308	2.02E-17	2.14E-16
Slc44a3	32	19.20213363	16.64547063	-2.556663001	2.08E-17	2.20E-16

Mir7086	3	66.34825328	48.48811752	-17.86013576	2.33E-17	2.47E-16
Gad2	2	17.95938929	1.275463146	-16.68392614	2.55E-17	2.69E-16
Kif1a	29	32.91794212	41.2508821	8.332939978	2.67E-17	2.82E-16
Porcn	36	17.45049565	12.2543724	-5.196123254	2.75E-17	2.90E-16
Mgp	8	78.90366314	70.05602947	-8.847633671	2.76E-17	2.91E-16
Vpreb2	5	90.96202375	78.73161028	-12.23041347	2.87E-17	3.02E-16
Ppp1r2-ps7	8	83.85503197	70.34864728	-13.50638469	2.95E-17	3.10E-16
Grin2c	60	10.24413688	15.8724758	5.628338922	3.02E-17	3.17E-16
10459M11I	78	4.890138789	3.816073142	-1.074065647	3.03E-17	3.18E-16
32451O06I	8	21.64134356	12.9403234	-8.701020167	3.06E-17	3.21E-16
Nudt16	42	2.605840371	1.161381559	-1.444458812	3.05E-17	3.21E-16
Ccdc60	11	56.55095956	46.44975971	-10.10119985	3.67E-17	3.85E-16
Smpdl3a	22	6.210727101	2.972616094	-3.238111007	3.71E-17	3.89E-16
Mir3547	2	70.99556565	48.56722057	-22.42834508	4.03E-17	4.22E-16
Slc6a3	32	14.91981038	23.85978205	8.939971672	4.06E-17	4.25E-16
C10263651	5	71.42857143	46.04576772	-25.38280371	4.07E-17	4.26E-16
Foxl1	8	49.24170924	26.01972351	-23.22198573	4.12E-17	4.30E-16
Mcemp1	4	74.13640168	55.96012811	-18.17627357	4.36E-17	4.55E-16
Adrb1	47	15.44809072	19.21519149	3.767100764	4.37E-17	4.56E-16
Pla2g2f	5	88.73789216	68.51948052	-20.21841164	4.47E-17	4.66E-16
Pabpc6	18	90.16513779	83.40861639	-6.756521395	4.49E-17	4.68E-16
Slc35d3	43	14.23615834	18.66783801	4.431679668	4.63E-17	4.82E-16
Tfap2e	23	14.56888688	25.41368038	10.8447935	4.70E-17	4.89E-16
Trim63	3	70.25131283	42.24256126	-28.00875156	4.86E-17	5.06E-16
B4galnt2	37	14.3875145	19.50209853	5.114584028	5.10E-17	5.30E-16
Galr3	3	92.49321364	74.8049317	-17.68828194	5.23E-17	5.43E-16
Rgs7	89	23.42785496	26.61209382	3.184238861	5.30E-17	5.50E-16
Galnt18	44	8.400910027	6.046944752	-2.353965275	5.97E-17	6.19E-16
Pros1	26	3.020113601	1.199870264	-1.820243336	6.13E-17	6.35E-16
Fam174b	25	33.69836409	28.79650735	-4.901856739	6.43E-17	6.66E-16
Ccdc160	18	30.44860964	22.83519025	-7.613419393	6.51E-17	6.74E-16
Zfp629	46	4.440628876	3.07370507	-1.366923805	6.81E-17	7.05E-16
Pced1b	43	11.02401985	8.555096819	-2.46892303	6.91E-17	7.14E-16
Galnt16	110	17.86522156	15.82591544	-2.039306125	7.34E-17	7.58E-16

430419L09F	56	2.690678727	1.527018897	-1.163659829	7.34E-17	7.58E-16
Slc38a3	41	20.32166566	16.87753438	-3.444131278	7.56E-17	7.79E-16
Ankrd13d	8	5.128122457	1.701740129	-3.426382328	7.56E-17	7.79E-16
700013F07F	9	5.175538849	15.27665001	10.10111116	8.26E-17	8.50E-16
Fcgbp	9	81.39590964	66.53666581	-14.85924382	8.69E-17	8.94E-16
Hoxd12	33	40.9340708	34.9251661	-6.008904703	8.72E-17	8.97E-16
Proz	3	48.5717523	19.38993464	-29.18181766	8.85E-17	9.10E-16
Lrch2	29	31.77828432	40.87444732	9.096162999	9.13E-17	9.38E-16
Abat	28	8.583298956	5.313929922	-3.269369034	9.43E-17	9.67E-16
Gcgr	24	28.63361705	33.15234935	4.518732297	9.46E-17	9.70E-16
Ccdc183	2	83.97181757	58.06784027	-25.90397729	9.58E-17	9.81E-16
Col11a2	24	38.87876895	31.03595871	-7.842810233	9.84E-17	1.01E-15
Evl	103	2.752664203	1.73092866	-1.021735543	1.03E-16	1.06E-15
Sv2a	18	4.440663472	2.321593671	-2.119069801	1.04E-16	1.06E-15
Cox4i2	8	54.85303383	68.5769034	13.72386958	1.04E-16	1.06E-15
Mir433	5	94.9061059	79.24015695	-15.66594895	1.05E-16	1.07E-15
Slc4a3	26	3.51164543	1.362858968	-2.148786461	1.15E-16	1.17E-15
Hormad1	28	92.0780049	87.85942043	-4.218584466	1.25E-16	1.27E-15
Dmxl2	114	1.972184622	0.948157159	-1.024027463	1.29E-16	1.31E-15
Rln1	9	23.77104521	16.34736135	-7.423683864	1.30E-16	1.32E-15
Slc30a10	37	23.92958001	30.37645783	6.44687782	1.44E-16	1.46E-15
Gm6484	4	23.25998185	9.368241246	-13.89174061	1.49E-16	1.52E-15
Mir7019	4	94.63177592	79.56284069	-15.06893524	1.53E-16	1.56E-15
Spi1	6	54.93339275	40.71364199	-14.21975076	1.63E-16	1.65E-15
Htra4	41	9.176003144	6.725193456	-2.450809688	1.83E-16	1.86E-15
Fgfr3	106	3.831878158	2.366594236	-1.465283922	1.96E-16	1.99E-15
Ctcflos	1	70.04132231	34.94505495	-35.09626737	2.00E-16	2.02E-15
Fhitos	1	91.46757679	53.40501792	-38.06255887	2.00E-16	2.02E-15
Lrrc55	1	100	75.77319588	-24.22680412	2.00E-16	2.02E-15
Slc25a45	1	49.14285714	21.71189979	-27.43095735	2.00E-16	2.02E-15
Zdhhc22	1	67.76859504	34.28571429	-33.48288076	2.00E-16	2.02E-15
Psd4	1	61.80257511	25.81818182	-35.98439329	2.00E-16	2.02E-15
Hk3	1	66.43835616	16.77018634	-49.66816983	2.00E-16	2.02E-15
Mir3080	1	86.24161074	54.06504065	-32.17657009	2.00E-16	2.02E-15

Olfr773	2	51.85391104	23.04096639	-28.81294466	2.11E-16	2.13E-15
Chchd10	20	1.922437708	5.047337525	3.124899818	2.12E-16	2.13E-15
Gm14327	3	37.30684327	15.7798108	-21.52703246	2.14E-16	2.15E-15
Kcnc1	103	12.81116697	16.52941918	3.718252219	2.17E-16	2.19E-15
Gnat1	1	65.42553191	24.75728155	-40.66825036	2.22E-16	2.23E-15
)30013C21F	7	53.39479702	69.43331506	16.03851804	2.28E-16	2.29E-15
Cbln4	27	54.36271856	60.52421969	6.161501133	2.31E-16	2.32E-15
Fam159a	22	25.04929024	34.01706141	8.967771166	2.43E-16	2.44E-15
Syndig1	79	47.88437533	52.41736697	4.532991641	2.49E-16	2.50E-15
Serinc2	21	8.427484277	4.421177319	-4.006306958	2.52E-16	2.53E-15
Kremen2	35	11.62657738	7.919308345	-3.70726903	2.73E-16	2.73E-15
Plekhb1	16	35.1541804	26.6214	-8.532780403	2.78E-16	2.78E-15
Cadps2	106	27.10144982	24.83575774	-2.265692079	2.80E-16	2.79E-15
Kcnn1	18	9.831559602	5.171038804	-4.660520799	2.81E-16	2.80E-15
Hoxc10	19	50.62926187	58.64877242	8.019510555	2.84E-16	2.84E-15
330017H08F	35	8.53071835	5.837538571	-2.693179779	2.85E-16	2.84E-15
Gm833	12	67.42376112	53.98078475	-13.44297636	2.94E-16	2.94E-15
Nrk	45	20.70979826	16.25024373	-4.459554531	3.00E-16	2.99E-15
Fgf3	47	11.43040639	16.99931204	5.568905643	3.01E-16	3.00E-15
Drd1	15	35.58785245	26.3607265	-9.227125943	3.31E-16	3.29E-15
Adgre1	1	73.45132743	20.53571429	-52.91561315	3.33E-16	3.31E-15
Chrnb3	2	57.91036236	32.32865978	-25.58170258	3.46E-16	3.43E-15
Marveld3	43	9.019820595	5.912070979	-3.107749616	3.61E-16	3.58E-15
30215H24F	12	9.480982358	18.3503826	8.869400243	3.71E-16	3.68E-15
Hspb1	11	8.229607457	1.488296442	-6.741311014	3.75E-16	3.72E-15
Serpinh1	9	7.901953646	3.17227773	-4.729675916	3.85E-16	3.82E-15
Olfm2	42	4.052393168	2.726067425	-1.326325743	4.02E-16	3.98E-15
430036J16F	4	76.02534707	59.39584065	-16.62950643	4.07E-16	4.03E-15
'00081O15F	127	3.906251378	2.927472613	-0.978778766	4.07E-16	4.03E-15
Fbxo2	41	9.420285247	6.946768129	-2.473517118	4.10E-16	4.06E-15
Ptprt	62	41.48628596	46.45944279	4.973156822	4.23E-16	4.18E-15
Gal	30	18.2375746	26.13664716	7.899072563	4.39E-16	4.34E-15
Galnt14	57	36.05252594	32.43854226	-3.613983683	4.56E-16	4.50E-15
Cidea	16	5.137345879	1.980180665	-3.157165214	4.63E-16	4.55E-15

430212C06F	16	5.137345879	1.980180665	-3.157165214	4.63E-16	4.55E-15
Fcgrt	7	91.61999563	79.22187558	-12.39812005	4.65E-16	4.58E-15
D2hgdh	9	15.21284448	4.453781621	-10.75906286	5.93E-16	5.83E-15
Gm38436	38	15.11554346	19.43937554	4.323832085	6.09E-16	5.99E-15
Ffar4	21	4.367597459	1.684722736	-2.682874723	6.28E-16	6.17E-15
Lppr1	32	24.92918756	20.37321979	-4.555967775	7.06E-16	6.93E-15
Nags	48	22.72307476	27.333401	4.61032624	7.26E-16	7.12E-15
Igfbpl1	39	32.01646113	37.81975787	5.803296737	7.28E-16	7.13E-15
Ccdc13	47	37.40075291	41.91366826	4.512915353	7.57E-16	7.42E-15
Gm648	5	89.36247487	67.70760835	-21.65486652	7.78E-16	7.62E-15
Iglon5	37	10.40921171	6.358879211	-4.050332496	7.85E-16	7.68E-15
Siglecf	2	74.41077441	43.31513595	-31.09563847	7.87E-16	7.69E-15
Zc3h12b	11	31.53132238	19.83018247	-11.70113991	7.89E-16	7.71E-15
Tpm2	48	30.31458484	25.16184859	-5.152736243	7.91E-16	7.72E-15
Gas2l2	17	61.72756481	53.12759744	-8.59996737	7.92E-16	7.73E-15
Phlda3	66	2.885786155	1.331857869	-1.553928286	7.93E-16	7.73E-15
Rcor2	34	3.640964913	2.089314936	-1.551649977	8.45E-16	8.23E-15
Pigz	25	10.60034538	5.610429412	-4.989915966	9.29E-16	9.05E-15
Hebp2	24	10.5121984	17.05352681	6.541328405	9.50E-16	9.24E-15
Cyp4v3	14	5.337815979	2.092816696	-3.244999283	9.90E-16	9.63E-15
Dpf1	37	4.731237254	2.298349855	-2.432887399	1.00E-15	9.72E-15
Tal2	5	13.34204962	4.909060539	-8.432989082	1.02E-15	9.90E-15
Rasl10a	70	6.467107766	5.342824479	-1.124283287	1.02E-15	9.90E-15
Lrcol1	6	41.77232694	30.65674437	-11.11558257	1.02E-15	9.93E-15
Thpo	18	31.20728442	41.26870921	10.06142479	1.04E-15	1.01E-14
Hoxa11os	50	39.2571663	34.71661631	-4.540549995	1.16E-15	1.13E-14
Galnt6	40	50.05064777	44.80461491	-5.246032859	1.19E-15	1.15E-14
Car10	39	45.30571093	50.78853469	5.482823756	1.26E-15	1.22E-14
Egfem1	62	57.27768876	53.62469858	-3.652990183	1.27E-15	1.22E-14
Dkk3	8	58.56336602	72.10195725	13.53859124	1.31E-15	1.26E-14
Cldn5	18	54.05957018	61.49421077	7.43464059	1.31E-15	1.26E-14
Mak	35	34.65407771	40.09750244	5.443424724	1.38E-15	1.34E-14
Pstpip1	20	41.44078096	31.79105967	-9.649721294	1.41E-15	1.36E-14
Magel2	23	61.04373505	54.83400985	-6.209725193	1.41E-15	1.36E-14

Crhr2	33	20.9859905	18.56102851	-2.424961986	1.41E-15	1.36E-14
Fgfr4	43	10.06167138	6.927733056	-3.133938321	1.42E-15	1.37E-14
Insrr	10	4.2095895	10.4029169	6.193327402	1.43E-15	1.37E-14
Xkr6	75	38.71179884	43.21902851	4.507229673	1.50E-15	1.44E-14
Adcy2	92	55.58239511	52.11792101	-3.4644741	1.53E-15	1.47E-14
Arpp21	50	43.74215531	39.63083985	-4.111315462	1.57E-15	1.51E-14
Atf3	26	2.101821447	4.729253383	2.627431936	1.59E-15	1.53E-14
C1ql3	29	9.944404412	7.26593759	-2.678466822	1.73E-15	1.66E-14
Mmel1	6	86.72223798	69.82085533	-16.90138266	1.79E-15	1.72E-14
Asic1	90	3.597062371	2.64149445	-0.955567921	1.81E-15	1.73E-14
)30624G23F	9	68.69158879	78.4426322	9.751043418	2.04E-15	1.95E-14
Wdr63	4	92.83426853	79.47951657	-13.35475195	2.16E-15	2.07E-14
Slco5a1	24	47.74697532	54.79927481	7.052299492	2.25E-15	2.15E-14
Vwa2	45	7.207601736	5.07995194	-2.127649796	2.51E-15	2.40E-14
Prdm12	32	27.38552469	23.60062925	-3.784895442	2.71E-15	2.59E-14
Rgs16	25	15.51668779	10.94707466	-4.569613122	2.72E-15	2.59E-14
Stk26	32	36.40464853	27.87633686	-8.528311665	2.81E-15	2.68E-14
Fam222a	127	3.645589254	6.327174255	2.681585001	2.82E-15	2.68E-14
Fam131b	57	5.589839681	4.799975988	-0.789863693	2.93E-15	2.79E-14
Mcam	42	5.664616706	2.495613146	-3.169003561	2.97E-15	2.83E-14
Fbxo44	54	7.127815014	5.338486594	-1.78932842	3.02E-15	2.87E-14
Rbfox3	40	27.96218496	23.88372572	-4.078459239	3.17E-15	3.01E-14
Rnf113a1	44	22.60961136	16.43769534	-6.171916028	3.20E-15	3.04E-14
Prr30	4	88.09345479	65.6301386	-22.4633162	3.20E-15	3.04E-14
Tppp3	10	6.519892088	15.22462427	8.704732178	3.24E-15	3.07E-14
Ckb	55	11.60039324	9.360643821	-2.239749421	3.30E-15	3.12E-14
'00001C02F	20	19.77100313	14.85031316	-4.920689971	3.58E-15	3.39E-14
Tacr1	30	25.70701428	20.21922012	-5.48779416	3.59E-15	3.40E-14
Reps2	31	24.40344298	15.90451555	-8.498927431	3.89E-15	3.67E-14
Mafa	124	12.56559929	15.23544061	2.669841313	4.16E-15	3.93E-14
Gmpr	21	28.545366	23.0176575	-5.527708495	4.18E-15	3.95E-14
)30412C18F	11	5.551660587	14.30032874	8.748668149	4.35E-15	4.11E-14
Zfp939	15	6.664663132	2.572742306	-4.091920825	4.46E-15	4.21E-14
Adamts5	30	25.74292439	20.26929308	-5.473631318	4.50E-15	4.24E-14

430421N21F	1	96.59863946	74.51737452	-22.08126494	4.55E-15	4.29E-14
Mir7047	3	85.42342077	61.81768372	-23.60573705	4.69E-15	4.41E-14
Rtn4rl1	115	2.706617009	1.880693765	-0.825923244	4.79E-15	4.51E-14
Zfp185	43	26.86973907	21.14370251	-5.726036566	4.85E-15	4.56E-14
Tmem26	12	30.04750993	39.010101	8.962591066	4.91E-15	4.61E-14
933429O19F	4	87.87488285	66.99873085	-20.87615199	4.91E-15	4.61E-14
Boll	103	73.7486659	76.40475126	2.656085364	5.54E-15	5.20E-14
Hspa12a	30	29.01151076	26.32193954	-2.689571221	6.03E-15	5.65E-14
Irf8	33	7.958758408	4.463776862	-3.494981545	6.20E-15	5.81E-14
Cyp4f14	2	82.79801807	51.81550232	-30.98251575	7.08E-15	6.63E-14
Hhat	21	4.779237617	2.478785726	-2.300451892	7.11E-15	6.66E-14
Des	38	17.58616024	23.46927664	5.883116396	7.56E-15	7.07E-14
Chst11	77	2.860116883	5.299285289	2.439168407	7.57E-15	7.08E-14
Ngf	15	5.159184758	2.669456347	-2.489728411	7.64E-15	7.14E-14
Trip6	15	7.303679425	3.089535119	-4.214144306	7.82E-15	7.30E-14
Dock10	47	21.72056756	19.12930179	-2.591265765	8.07E-15	7.53E-14
Slc38a1	62	21.59269057	18.52481491	-3.067875656	8.34E-15	7.78E-14
Gm15867	45	3.638511542	1.675485377	-1.963026166	8.44E-15	7.86E-14
Smlr1	3	86.91719446	62.14886018	-24.76833428	8.43E-15	7.86E-14
333427G06F	11	28.49429246	38.36580573	9.871513263	8.45E-15	7.86E-14
Sema4b	37	10.09691094	6.556014484	-3.540896458	8.74E-15	8.13E-14
Cblc	28	15.10009024	21.51441125	6.414321012	8.84E-15	8.22E-14
Zfp449	17	23.38895353	16.00047688	-7.388476646	8.87E-15	8.23E-14
Odf3b	14	17.47956689	10.37482511	-7.104741783	9.13E-15	8.48E-14
Cftr	8	5.897854478	1.742297909	-4.155556569	9.27E-15	8.60E-14
Sgsm1	20	7.232590089	3.108133754	-4.124456335	9.52E-15	8.83E-14
Mir6921	4	83.5989887	68.41338622	-15.18560248	1.01E-14	9.37E-14
Slc22a2	4	79.4578508	60.6377346	-18.8201162	1.12E-14	1.04E-13
Tspan6	12	36.92243275	26.96506625	-9.957366501	1.15E-14	1.07E-13
Lcn12	2	66.34212306	27.93650794	-38.40561512	1.16E-14	1.07E-13
Il1rap	33	3.410247911	1.261477754	-2.148770157	1.17E-14	1.08E-13
Kcnf1	23	9.388178696	4.28860365	-5.099575046	1.28E-14	1.19E-13
Prph	33	14.65369465	11.47861034	-3.175084316	1.29E-14	1.19E-13
Adamts16	18	15.65754046	12.56432067	-3.093219794	1.36E-14	1.25E-13

Izumo1r	3	66.5960052	50.06275568	-16.53324952	1.38E-14	1.28E-13
Rnf128	86	19.6700435	16.88544584	-2.784597664	1.39E-14	1.28E-13
Abcd1	13	29.55431527	16.4287993	-13.12551597	1.41E-14	1.30E-13
)33427D14F	3	95.56405896	81.78933207	-13.77472689	1.42E-14	1.31E-13
Cxxc5	69	2.790238312	1.546059562	-1.24417875	1.44E-14	1.33E-13
Gsn	60	12.69153089	9.920422385	-2.7711085	1.46E-14	1.34E-13
Ptch2	17	6.619616802	3.457585983	-3.16203082	1.51E-14	1.38E-13
Dpysl3	47	16.88292974	14.46697506	-2.415954688	1.59E-14	1.46E-13
Pla2g4e	3	58.01608737	41.28847245	-16.72761492	1.62E-14	1.49E-13
Sds	17	36.80409037	28.9966641	-7.807426275	1.63E-14	1.49E-13
Tmem59l	16	64.9262434	72.51628247	7.590039071	1.68E-14	1.54E-13
Gphn	111	2.44618189	1.475670472	-0.970511419	1.69E-14	1.55E-13
Tspan2os	9	7.785061246	2.92128232	-4.863778926	1.73E-14	1.58E-13
Izumo2	2	52.30966217	22.78011204	-29.52955012	1.74E-14	1.59E-13
Apba2	124	62.11233116	65.50111781	3.388786647	1.77E-14	1.62E-13
Aifm1	25	24.92008676	17.9315678	-6.98851896	1.91E-14	1.74E-13
Snta1	79	3.019584211	1.930247215	-1.089336996	2.08E-14	1.89E-13
Slc44a5	25	47.98156961	40.55534374	-7.42622587	2.10E-14	1.91E-13
Vsx1	20	14.89495904	10.24213706	-4.65282198	2.13E-14	1.94E-13
Epha6	19	40.03473255	47.62358471	7.588852161	2.22E-14	2.02E-13
Adamts15	10	17.47289294	26.61977324	9.146880302	2.37E-14	2.16E-13
Mir6538	32	37.13516625	42.99480301	5.859636766	2.38E-14	2.16E-13
Kcnk5	5	6.811409043	15.23271354	8.421304495	2.70E-14	2.46E-13
Ptp4a3	86	4.409040923	2.756645194	-1.652395729	2.71E-14	2.47E-13
Isyna1	43	2.85434083	1.541373785	-1.312967045	2.78E-14	2.52E-13
Armcx3	9	38.92844411	23.6739525	-15.25449161	2.78E-14	2.52E-13
A1cf	5	71.29472953	53.83011149	-17.46461803	2.82E-14	2.56E-13
Nppc	62	6.327373191	4.953990535	-1.373382656	2.84E-14	2.57E-13
Shisa7	22	38.84379496	44.64992772	5.806132763	2.88E-14	2.61E-13
Bmp2	95	2.244564229	1.435603422	-0.808960807	2.89E-14	2.62E-13
Slc45a1	39	21.30525282	25.9175485	4.612295683	2.96E-14	2.68E-13
Gm5122	4	52.5332869	33.36232221	-19.17096469	3.04E-14	2.75E-13
Lclat1	52	2.530358405	1.086327706	-1.4440307	3.08E-14	2.78E-13
Prkcz	116	20.28497594	19.4478574	-0.837118543	3.11E-14	2.81E-13

Pcdhgc5	2	50.98039216	19.39393939	-31.58645276	3.12E-14	2.82E-13
Mir196a-1	9	33.70183647	22.37513843	-11.32669804	3.13E-14	2.82E-13
Dnajb5	1	53.68731563	26.39593909	-27.29137655	3.22E-14	2.90E-13
Fahd2a	6	1.407501204	10.25127894	8.843777741	3.34E-14	3.01E-13
Tmem171	22	18.28861379	13.79971076	-4.488903028	3.38E-14	3.04E-13
930412O13F	30	54.13787939	47.64122088	-6.496658513	3.38E-14	3.04E-13
Cldn14	11	61.49729735	48.69128378	-12.80601357	3.63E-14	3.26E-13
Eps8l2	24	21.34069512	16.54402965	-4.796665476	3.64E-14	3.27E-13
933427I22R	4	81.28450463	63.57490409	-17.70960054	3.70E-14	3.32E-13
Grp	8	10.03889515	2.253029997	-7.78586515	3.75E-14	3.36E-13
Ank1	180	22.23120982	25.96679933	3.735589504	3.86E-14	3.46E-13
Morc4	67	23.21839261	19.43962577	-3.77876684	3.92E-14	3.52E-13
Kcnip1	49	45.7008068	41.07772233	-4.623084469	3.93E-14	3.52E-13
Sh3bgr	9	16.8334465	8.156608486	-8.676838012	3.99E-14	3.58E-13
Kcnj4	117	6.892793212	6.669156025	-0.223637187	4.04E-14	3.61E-13
Tbc1d9	44	12.39915847	10.09754083	-2.301617646	4.07E-14	3.64E-13
.30012A19F	36	5.298227719	2.372452482	-2.925775237	4.11E-14	3.67E-13
Thbd	9	19.12907302	32.09611473	12.96704171	4.25E-14	3.79E-13
Pax3	25	35.8468801	42.7996257	6.952745602	4.92E-14	4.39E-13
Zmym3	25	26.38047446	19.77104635	-6.609428113	5.16E-14	4.60E-13
Rps6ka6	11	83.93584701	74.12864873	-9.807198286	5.25E-14	4.68E-13
Pdzd4	49	13.62833692	10.22676583	-3.401571091	5.35E-14	4.77E-13
Aff2	48	17.53284005	12.43646163	-5.096378418	5.39E-14	4.80E-13
Ptgfr	10	22.45528962	16.28993975	-6.16534987	5.71E-14	5.08E-13
Cdh11	39	58.74423561	51.95037035	-6.793865266	5.95E-14	5.29E-13
Cpne9	22	34.15729494	40.5955249	6.438229954	6.40E-14	5.69E-13
Rspo3	36	7.816075645	13.05595209	5.23987645	6.48E-14	5.75E-13
Zfhx2os	9	87.71485489	75.5533969	-12.16145799	6.90E-14	6.13E-13
Ttc9	63	4.623762268	3.282470698	-1.341291571	6.98E-14	6.19E-13
Sox7	80	15.04214946	16.29444946	1.252300007	7.00E-14	6.21E-13
Hmgn5	14	28.6647679	19.7918504	-8.872917503	7.27E-14	6.44E-13
Dnajc22	11	8.087270112	2.824325383	-5.262944729	7.35E-14	6.51E-13
Car11	15	14.31235341	25.01075395	10.69840054	7.41E-14	6.55E-13
'00057H15F	5	28.8926389	16.06030842	-12.83233048	7.51E-14	6.64E-13

Tmem132c	37	45.86373844	52.42711357	6.563375132	7.58E-14	6.70E-13
P3h2	63	2.922779997	1.583686558	-1.339093438	7.88E-14	6.96E-13
Kcnj11	26	36.35597659	44.278021	7.922044414	8.22E-14	7.26E-13
Csmd3	11	19.63803513	13.31278515	-6.325249981	8.31E-14	7.33E-13
Alx4	54	19.89922886	26.79062006	6.891391206	8.59E-14	7.58E-13
Cracr2b	7	37.07538546	24.39359899	-12.68178646	9.09E-14	8.01E-13
Esrrg	83	44.19439387	47.09989468	2.905500809	9.11E-14	8.03E-13
Igll1	1	84.97109827	50.62761506	-34.3434832	9.20E-14	8.10E-13
Pdha1	34	22.33530129	15.57889487	-6.756406418	9.28E-14	8.16E-13
Pcbp3	40	23.47650426	28.93406807	5.45756381	9.59E-14	8.43E-13
Lhx6	42	15.9680219	11.453225	-4.514796898	9.97E-14	8.76E-13
Mmp9	2	67.14285714	35.16340812	-31.97944902	1.00E-13	8.81E-13
Slc27a2	41	32.750876	37.30987479	4.558998793	1.06E-13	9.28E-13
Gad1	17	14.30646431	11.01632995	-3.290134355	1.06E-13	9.31E-13
Syn1	7	33.60024807	15.53024652	-18.07000155	1.07E-13	9.36E-13
Syt2	63	5.099817961	7.89552657	2.79570861	1.13E-13	9.87E-13
Sycp2	22	93.40950248	88.48082488	-4.928677607	1.15E-13	1.01E-12
Cldn16	6	85.20545729	70.9729324	-14.23252489	1.18E-13	1.03E-12
Usp27x	10	22.40982156	13.86777534	-8.542046221	1.21E-13	1.06E-12
Hoga1	5	84.93114454	68.02307622	-16.90806832	1.24E-13	1.09E-12
Tmem229b	68	3.055410692	1.623887245	-1.431523447	1.26E-13	1.10E-12
Lhx3	29	26.49304017	29.8494254	3.356385232	1.29E-13	1.12E-12
Pomc	5	34.10942995	22.93089064	-11.1785393	1.30E-13	1.13E-12
Uhmk1	50	2.947317343	1.994072506	-0.953244837	1.31E-13	1.14E-12
Lfng	56	14.69621994	11.31162692	-3.384593022	1.32E-13	1.15E-12
Slc16a2	33	30.65818341	24.69422495	-5.963958454	1.34E-13	1.17E-12
Ebf4	148	5.124070388	4.793388954	-0.330681434	1.40E-13	1.22E-12
Atp4b	4	69.37233121	55.2431518	-14.12917941	1.43E-13	1.25E-12
Pvrl1	94	2.376591418	1.468002437	-0.908588981	1.44E-13	1.25E-12
Mir7093	15	39.92705997	28.43588123	-11.49117874	1.48E-13	1.29E-12
Lrrc74b	11	7.074364865	2.13515341	-4.939211456	1.50E-13	1.30E-12
Ank3	5	56.76562326	42.43421053	-14.33141273	1.51E-13	1.31E-12
Wnt11	147	3.366487363	2.522830946	-0.843656417	1.53E-13	1.33E-12
Cetn2	15	26.59588452	19.44060052	-7.155283999	1.54E-13	1.33E-12

Pcdh8	32	42.82853098	49.32712731	6.498596327	1.54E-13	1.33E-12
Pak3	10	32.03314725	20.23360174	-11.79954551	1.56E-13	1.35E-12
Calml4	9	16.04579147	10.07677458	-5.969016891	1.58E-13	1.36E-12
Mir92b	16	13.10688977	6.999280805	-6.107608964	1.61E-13	1.40E-12
Arsi	48	42.40490047	39.43097095	-2.973929522	1.63E-13	1.41E-12
Platr22	7	44.26586234	29.5604734	-14.70538895	1.72E-13	1.49E-12
Mir431	11	92.44054751	82.85458237	-9.58596514	1.76E-13	1.51E-12
Ncan	6	7.693002975	18.0763496	10.38334663	1.75E-13	1.51E-12
Gnb3	7	73.85134511	58.91603612	-14.93530899	1.76E-13	1.52E-12
Mir6403	8	89.89581567	80.52663486	-9.36918081	1.77E-13	1.52E-12
330074K22R	126	2.967563944	2.026155692	-0.941408252	1.78E-13	1.53E-12
Mir26a-1	9	82.84595449	90.50060838	7.654653893	1.84E-13	1.59E-12
Lcn9	1	68.85714286	40.63492063	-28.22222222	1.88E-13	1.62E-12
Ackr2	5	90.76193033	80.39531278	-10.36661755	1.93E-13	1.66E-12
30201H02R	23	45.72754462	52.16979584	6.442251217	1.94E-13	1.67E-12
BC021785	5	85.52380952	68.74686203	-16.7769475	1.95E-13	1.67E-12
530080O11R	57	3.049036512	5.116652241	2.067615729	1.97E-13	1.69E-12
Hook1	57	3.049036512	5.116652241	2.067615729	1.97E-13	1.69E-12
Arhgef19	5	47.12645011	34.21643978	-12.91001033	2.01E-13	1.73E-12
Parp12	74	2.625338254	0.926327035	-1.699011218	2.03E-13	1.74E-12
Cav3	4	44.37794493	24.82128846	-19.55665647	2.15E-13	1.84E-12
Pappa	33	33.68744908	29.03738188	-4.650067207	2.24E-13	1.92E-12
Mn1	171	28.96871755	28.50606975	-0.462647806	2.25E-13	1.92E-12
Rai1	103	2.868475435	1.575385238	-1.293090197	2.33E-13	1.99E-12
Ifitm2	5	32.76340669	20.83570661	-11.92770008	2.34E-13	2.00E-12
Fcamr	2	78.06802078	54.22632396	-23.84169682	2.36E-13	2.01E-12
Vipr1	74	5.934279811	4.072830835	-1.861448977	2.39E-13	2.04E-12
Tescl	13	16.6365062	26.18728659	9.550780393	2.39E-13	2.04E-12
Fbrsl1	119	9.344487674	12.35134674	3.006859061	2.41E-13	2.05E-12
Susd5	44	33.4556475	28.67251802	-4.783129483	2.46E-13	2.09E-12
Hprt	61	18.31360318	14.40650003	-3.907103151	2.48E-13	2.11E-12
Irs3	15	21.74796125	30.2880091	8.54004785	2.54E-13	2.16E-12
Mir7006	6	88.78730855	95.54006062	6.752752067	2.57E-13	2.18E-12
Npr1	21	6.76919191	3.273012172	-3.496179737	2.58E-13	2.19E-12

Plin3	3	5.965811244	0.518709446	-5.447101798	2.60E-13	2.20E-12
Mapk4	47	5.154190968	4.167494699	-0.98669627	2.84E-13	2.41E-12
Sebox	4	78.89132554	58.44128923	-20.45003631	3.05E-13	2.59E-12
Rimkla	66	12.96582564	15.8389977	2.873172063	3.08E-13	2.61E-12
Aard	33	35.65284702	40.34947482	4.696627796	3.55E-13	3.01E-12
Ubash3b	92	3.898490106	6.330385379	2.431895273	3.65E-13	3.09E-12
Gyk	25	20.65107572	13.71954635	-6.931529371	3.70E-13	3.13E-12
Cdc42ep3	98	3.055648034	1.736499707	-1.319148327	3.81E-13	3.23E-12
Tnik	32	13.39326541	10.95048838	-2.442777035	3.83E-13	3.24E-12
Terc	37	11.8601493	9.589281026	-2.270868274	3.95E-13	3.33E-12
Mir466f-2	45	3.171248546	2.121229319	-1.050019227	3.96E-13	3.34E-12
Pvalb	1	95.40816327	70.45454545	-24.95361781	3.99E-13	3.36E-12
Pdk3	62	21.1283783	17.13299735	-3.995380951	4.04E-13	3.40E-12
Piwil2	7	93.42194391	85.46384368	-7.958100234	4.04E-13	3.40E-12
10051M20I	6	14.0290277	6.685454781	-7.343572923	4.04E-13	3.40E-12
Otp	20	42.94676137	49.34009563	6.393334259	4.10E-13	3.45E-12
Nyap1	1	54.38066465	27.73333333	-26.64733132	4.43E-13	3.73E-12
Uncx	57	29.25666556	35.12778028	5.871114723	4.51E-13	3.80E-12
Nmnat2	23	29.60968553	35.78909736	6.179411826	4.58E-13	3.85E-12
Egfl7	38	21.66313603	17.71922141	-3.943914618	4.98E-13	4.18E-12
Pabpc5	11	23.05318663	10.52385399	-12.52933264	5.01E-13	4.21E-12
Zbtb8b	31	16.98146423	24.12213325	7.140669022	5.17E-13	4.33E-12
C1qtnf1	13	61.05604927	53.31309553	-7.74295374	5.23E-13	4.38E-12
Stk33	40	19.55768404	25.51280727	5.955123228	5.33E-13	4.47E-12
Tdrd6	46	47.59594626	52.73715858	5.141212318	5.34E-13	4.47E-12
Tmem54	30	6.511819466	3.497099648	-3.014719818	6.45E-13	5.40E-12
Pkp3	16	59.07907974	50.91100118	-8.168078554	7.13E-13	5.96E-12
Xk	51	14.14486474	10.92113953	-3.223725209	7.18E-13	6.00E-12
Wdr44	42	19.21371798	12.43186639	-6.781851592	7.29E-13	6.09E-12
Arl5c	13	54.01091785	44.60681568	-9.404102161	7.45E-13	6.22E-12
Mb	6	93.8939711	85.36808908	-8.525882022	7.45E-13	6.22E-12
Slc25a53	18	25.34417297	18.97240297	-6.371769999	7.60E-13	6.34E-12
Mir196b	30	37.0336282	42.70197133	5.668343129	7.64E-13	6.37E-12
Lrat	5	29.94232722	16.45169011	-13.49063711	7.73E-13	6.45E-12

Kctd12	16	12.13513484	7.623511624	-4.511623219	7.74E-13	6.45E-12
Fam155a	76	40.05670001	42.71626017	2.659560163	7.87E-13	6.55E-12
Tcea3	53	4.05099215	2.990390236	-1.060601915	7.93E-13	6.60E-12
Igf2r	53	44.36888533	51.1707793	6.801893971	8.07E-13	6.71E-12
Morn3	11	46.6272279	36.73942234	-9.887805558	8.27E-13	6.87E-12
Mrap2	10	9.500608505	18.07157026	8.57096175	8.69E-13	7.22E-12
Dab2ip	117	7.799057642	6.388290433	-1.410767209	8.83E-13	7.33E-12
Rgma	70	15.94058832	14.30859126	-1.631997059	8.88E-13	7.36E-12
Cul9	31	43.45992971	48.44348692	4.983557212	9.15E-13	7.58E-12
Gm11213	5	73.75385089	57.05465803	-16.69919286	9.24E-13	7.66E-12
S1pr3	28	25.53712601	22.19909856	-3.338027451	9.40E-13	7.79E-12
Foxi3	42	42.63192099	38.44316133	-4.188759652	9.46E-13	7.83E-12
Nfkbia	40	5.728310806	2.664624876	-3.06368593	9.91E-13	8.19E-12
Zim3	23	83.55327407	77.04373086	-6.509543213	1.04E-12	8.58E-12
Dnah11	7	10.39032924	19.31029729	8.91996805	1.08E-12	8.92E-12
Nptxr	83	2.013350236	1.232859159	-0.780491077	1.09E-12	9.04E-12
Cd8b1	2	63.34256329	36.06709703	-27.27546626	1.10E-12	9.09E-12
Emd	18	17.96338187	10.58040926	-7.382972613	1.11E-12	9.17E-12
Sema6b	43	10.19492265	7.069143809	-3.125778843	1.11E-12	9.19E-12
Tnfsf15	8	36.20674951	22.18962558	-14.01712393	1.12E-12	9.23E-12
Tbx3	41	49.25891647	53.35614503	4.09722856	1.12E-12	9.26E-12
Trp73	84	18.413454	16.91808522	-1.495368776	1.14E-12	9.37E-12
Rap2c	84	18.1580473	15.12457288	-3.033474426	1.16E-12	9.57E-12
Slc6a19	11	86.81808427	76.89595572	-9.922128553	1.20E-12	9.89E-12
Zfp354a	12	8.110499168	3.64931945	-4.461179718	1.24E-12	1.02E-11
Gpsm3	6	37.09581121	51.05757879	13.96176758	1.28E-12	1.05E-11
Cd55b	7	50.68467895	39.8070574	-10.87762155	1.28E-12	1.05E-11
Abcc8	29	20.14808461	26.64964142	6.501556811	1.30E-12	1.06E-11
Atp1b1	68	2.914065733	1.042950644	-1.87111509	1.37E-12	1.13E-11
Adam2	10	53.0721019	44.1963404	-8.875761497	1.42E-12	1.17E-11
Wfdc5	3	68.0054398	43.95269736	-24.05274244	1.52E-12	1.25E-11
Hfm1	64	28.88591025	26.29293745	-2.592972802	1.54E-12	1.26E-11
Emb	50	19.16637832	23.05811639	3.891738073	1.62E-12	1.33E-11
Skor2	21	42.67295166	48.90713942	6.234187755	1.65E-12	1.35E-11

Kcna3	77	32.24866967	28.77173733	-3.476932338	1.70E-12	1.39E-11
Pou5f1	8	43.304071	33.64186843	-9.66220257	1.71E-12	1.40E-11
Cacng4	125	11.38603806	14.37845073	2.992412668	1.77E-12	1.45E-11
Drd3	5	90.60011874	77.46268657	-13.13743217	1.79E-12	1.46E-11
St5	18	33.17709458	41.15661862	7.979524033	1.80E-12	1.47E-11
Kank1	60	2.914190824	1.626375708	-1.287815116	1.82E-12	1.49E-11
Sync	21	8.138184172	3.805778231	-4.332405942	1.84E-12	1.50E-11
Krt71	6	41.86912428	32.44651134	-9.42261294	1.86E-12	1.52E-11
Krt5	5	80.86476409	71.48646209	-9.378301999	1.87E-12	1.52E-11
Neurog2	38	7.934961959	5.65195452	-2.283007438	1.98E-12	1.61E-11
Smarca5-ps	33	67.37304981	71.24008048	3.867030665	2.02E-12	1.64E-11
Nek5	13	19.31062068	14.28224215	-5.028378528	2.03E-12	1.65E-11
Adamts12	3	25.78616352	6.734006734	-19.05215679	2.12E-12	1.72E-11
Stxbp5l	35	18.56327305	14.10757642	-4.455696628	2.18E-12	1.77E-11
Ramp1	35	2.052523118	1.104194814	-0.948328304	2.19E-12	1.78E-11
Fgf4	60	22.25852967	20.28490425	-1.973625425	2.22E-12	1.80E-11
Gpr137c	73	2.315351668	1.499574607	-0.815777062	2.23E-12	1.81E-11
Ppm1n	35	5.595224747	3.471922388	-2.12330236	2.25E-12	1.82E-11
Gm20063	12	39.95465198	33.14864614	-6.806005834	2.37E-12	1.92E-11
Stac2	23	8.37337535	16.19928	7.825904653	2.48E-12	2.00E-11
Ecel1	37	10.36286123	15.21206408	4.849202856	2.54E-12	2.06E-11
933416E03R	6	71.95042066	61.65777011	-10.29265055	2.58E-12	2.08E-11
Nbea	137	2.778595155	2.023171361	-0.755423794	2.67E-12	2.15E-11
H2-Q7	3	11.56993466	3.832241862	-7.737692797	2.69E-12	2.17E-11
Rnf180	10	47.27104859	38.60795578	-8.663092813	2.80E-12	2.26E-11
Zbtb8a	20	3.141805852	1.133610053	-2.008195799	2.97E-12	2.40E-11
Tal1	63	18.18738967	16.03931525	-2.148074418	3.01E-12	2.43E-11
Apobec2	5	90.09284156	79.11116451	-10.98167706	3.12E-12	2.51E-11
'00116B05R	5	53.79945713	41.65021639	-12.14924075	3.28E-12	2.64E-11
Rbmxl2	66	74.34915627	77.84715214	3.497995878	3.31E-12	2.66E-11
Nefl	36	5.32675135	9.015831512	3.689080162	3.33E-12	2.68E-11
Pdzrn3	49	55.01011683	60.25978877	5.249671936	3.83E-12	3.08E-11
Tdrp	74	4.31276086	6.194565919	1.881805059	3.86E-12	3.10E-11
Nr2f2	143	3.402426743	2.706077847	-0.696348896	3.98E-12	3.19E-11

Olfm1	234	9.36962688	11.19276153	1.823134652	4.12E-12	3.30E-11
Gdpd3	4	48.05790126	33.61512935	-14.44277191	4.25E-12	3.40E-11
Mir434	3	93.53372418	83.3637507	-10.16997348	4.36E-12	3.49E-11
Acaa1b	19	5.744468904	11.75359964	6.009130737	4.51E-12	3.61E-11
Gm53	25	22.71338435	18.39427212	-4.319112232	4.56E-12	3.65E-11
Dpep1	11	61.09464515	51.79576307	-9.298882083	4.64E-12	3.71E-11
Rftn1	67	49.38759715	51.78541558	2.397818436	5.04E-12	4.03E-11
Flt3	22	6.527447729	3.327120205	-3.200327524	5.10E-12	4.07E-11
Nt5c1a	18	19.42189795	27.35682593	7.934927988	5.11E-12	4.08E-11
Cdh8	33	55.90188956	60.9162618	5.014372244	5.16E-12	4.11E-11
Tmem215	14	53.81228047	61.41120436	7.59892389	5.16E-12	4.12E-11
Esm1	10	45.81797094	54.45273659	8.634765652	5.19E-12	4.14E-11
Prss12	72	4.986827848	3.753566979	-1.233260869	5.50E-12	4.38E-11
Bcl2l2	17	4.452916477	2.071945431	-2.380971047	5.71E-12	4.55E-11
Ppp1r3b	9	31.18416434	22.64659926	-8.537565073	6.04E-12	4.81E-11
Grk1	8	97.1597034	90.98774491	-6.171958487	6.22E-12	4.95E-11
Brsk2	95	9.032301231	11.94316706	2.910865831	6.28E-12	4.99E-11
Fam221b	2	11.12159784	0.888941103	-10.23265673	6.32E-12	5.02E-11
Bmyc	35	10.39719633	7.656470898	-2.740725429	6.40E-12	5.08E-11
)30455G09F	2	77.69723093	43.63131313	-34.0659178	6.47E-12	5.13E-11
Rbakdn	4	65.21093286	48.87662441	-16.33430845	6.64E-12	5.26E-11
Lincred1	2	84.50635386	62.13499098	-22.37136288	7.30E-12	5.79E-11
Krt73	3	74.98857816	50.82621083	-24.16236734	7.54E-12	5.98E-11
Tbkbp1	16	6.80068922	3.656853923	-3.143835297	7.67E-12	6.07E-11
Myo3a	8	41.49287794	32.41266798	-9.080209962	7.77E-12	6.15E-11
Scn5a	131	18.99664761	17.40082869	-1.59581892	7.90E-12	6.25E-11
Tert	35	5.032584677	3.331439227	-1.701145449	7.91E-12	6.25E-11
Arhgap28	40	57.71947675	62.84162322	5.122146471	7.99E-12	6.32E-11
Hymai	49	51.43467536	55.06687661	3.632201248	8.07E-12	6.37E-11
Ptpn18	29	4.095088578	1.712612199	-2.382476378	8.12E-12	6.41E-11
Col9a1	5	91.3904326	78.72609111	-12.66434148	8.27E-12	6.52E-11
Fbln7	16	7.60079628	4.066600857	-3.534195423	8.50E-12	6.70E-11
Gjb1	12	75.14987529	83.97573177	8.825856485	8.98E-12	7.08E-11
)30504O13F	3	86.2949808	66.18652744	-20.10845337	9.25E-12	7.28E-11

Itgb8	7	57.14111432	67.30686366	10.16574934	9.37E-12	7.38E-11
Mir143	4	96.1979299	74.99981428	-21.19811562	9.44E-12	7.43E-11
Mmp23	48	51.29462016	54.6440809	3.349460738	1.04E-11	8.19E-11
Snx31	14	49.25118895	55.69230541	6.441116467	1.05E-11	8.23E-11
Foxo4	7	22.08282823	10.57657745	-11.50625078	1.06E-11	8.34E-11
Hspa1l	9	7.378211315	3.238990252	-4.139221063	1.06E-11	8.34E-11
Igf2bp3	16	41.33543217	49.62342736	8.287995187	1.10E-11	8.64E-11
Tmeff1	111	2.610235717	1.896928889	-0.713306829	1.11E-11	8.68E-11
ı21504E06F	8	34.16090209	46.91785042	12.75694833	1.11E-11	8.72E-11
Themis2	18	13.0283108	17.97571178	4.947400981	1.12E-11	8.81E-11
Ffar1	6	85.31615282	70.76983938	-14.54631344	1.14E-11	8.96E-11
Rgs3	7	81.29253197	61.91248711	-19.38004486	1.18E-11	9.27E-11
Btbd3	77	9.800608765	7.096360022	-2.704248744	1.19E-11	9.28E-11
Nnat	16	74.55656007	81.69633051	7.139770437	1.27E-11	9.92E-11
Gm16551	23	30.95683323	26.03774318	-4.919090043	1.29E-11	1.00E-10
Igfals	4	66.61833387	55.28253885	-11.33579502	1.29E-11	1.01E-10
Hoxa9	1	42.33870968	14.47963801	-27.85907167	1.29E-11	1.01E-10
Fezf1	17	48.35601035	56.67510676	8.319096408	1.29E-11	1.01E-10
Il27ra	19	4.879561416	2.701822872	-2.177738545	1.34E-11	1.05E-10
Prm1	11	79.17384248	87.77160052	8.597758045	1.36E-11	1.06E-10
700123I01R	3	60.15035013	43.50949198	-16.64085815	1.38E-11	1.07E-10
Bend4	44	14.62421609	11.43207806	-3.192138039	1.38E-11	1.08E-10
Chst15	44	1.904047516	3.705354403	1.801306886	1.43E-11	1.11E-10
Epha10	27	24.9248796	31.26558737	6.340707768	1.43E-11	1.11E-10
Xiap	17	26.48706696	18.77651792	-7.710549039	1.48E-11	1.15E-10
ı30012L18F	36	3.175537674	1.434194386	-1.741343287	1.51E-11	1.18E-10
Il1r1	20	5.098772754	2.19533176	-2.903440994	1.62E-11	1.25E-10
Lingo2	52	31.9972928	28.10858537	-3.888707431	1.64E-11	1.27E-10
Nlrp2	1	76.19047619	27.88461538	-48.30586081	1.70E-11	1.32E-10
ı32441J04R	3	69.69177018	50.38895504	-19.30281514	1.71E-11	1.33E-10
Zfp280c	45	25.19021237	20.37113781	-4.819074557	1.71E-11	1.33E-10
Prtg	115	6.737947998	5.90271327	-0.835234728	1.72E-11	1.33E-10
Vsnl1	2	45.12195122	19.29824561	-25.82370561	1.74E-11	1.35E-10
Ltb	6	52.51048444	42.10014567	-10.41033877	1.75E-11	1.35E-10

Iqsec3	33	51.97601959	46.43963657	-5.536383022	1.75E-11	1.35E-10
Sorcs3	76	57.82627143	60.87973385	3.053462416	1.76E-11	1.36E-10
;10524H06F	32	5.432350942	3.39959412	-2.032756822	1.83E-11	1.41E-10
Nfe2l3	27	8.58101234	4.702190081	-3.878822259	1.95E-11	1.50E-10
Zkscan2	21	44.31234412	54.23035889	9.91801477	1.98E-11	1.53E-10
Foxf2	58	3.628095085	1.714116656	-1.91397843	2.17E-11	1.67E-10
Fam46a	28	4.732127983	2.954968427	-1.777159555	2.23E-11	1.72E-10
)30011G23F	33	3.348980458	1.396500028	-1.95248043	2.28E-11	1.75E-10
Mir1905	44	88.70199636	90.42200325	1.720006891	2.32E-11	1.79E-10
Frem3	7	16.49859944	8.134234814	-8.364364626	2.36E-11	1.81E-10
Hs6st2	53	33.23558915	29.13422482	-4.101364325	2.36E-11	1.82E-10
Trpm3	10	21.11338929	27.96323449	6.849845202	2.41E-11	1.86E-10
Msmp	3	88.5051617	73.97532863	-14.52983307	2.58E-11	1.99E-10
Osgin1	7	14.82427563	8.315986417	-6.508289212	2.61E-11	2.00E-10
Xkr7	48	43.18653844	48.19404178	5.007503344	2.66E-11	2.04E-10
Gm15401	1	71.2	28.03738318	-43.16261682	2.66E-11	2.04E-10
Sphk1	127	6.193201119	4.512336249	-1.68086487	2.70E-11	2.07E-10
Lipg	22	6.909657313	3.655979223	-3.25367809	2.71E-11	2.08E-10
.10015C05F	3	59.31656995	41.96976023	-17.34680973	2.79E-11	2.13E-10
Cacna1e	20	42.14899003	37.33157629	-4.817413742	2.80E-11	2.14E-10
30119M05I	2	45.2991453	17.96296296	-27.33618234	2.83E-11	2.16E-10
Plekhg6	24	8.09609373	4.839445642	-3.256648088	2.89E-11	2.21E-10
Aym1	6	38.13223286	22.47924619	-15.65298667	2.91E-11	2.23E-10
Sh3kbp1	63	19.51711866	16.39631597	-3.120802694	2.92E-11	2.23E-10
Capn9	2	88.28462008	72.14444659	-16.14017348	2.93E-11	2.24E-10
Robo2	21	27.8120737	21.70298877	-6.109084931	2.95E-11	2.25E-10
Sptbn1	18	55.99179427	63.15229075	7.160496482	2.96E-11	2.26E-10
Igsf10	5	12.51814924	26.09067797	13.57252873	3.02E-11	2.30E-10
C2cd4b	36	11.54291076	9.280907943	-2.262002814	3.03E-11	2.31E-10
Tll2	17	9.21837014	5.954897759	-3.263472381	3.24E-11	2.47E-10
Rpl10l	16	94.05039059	87.9077182	-6.142672389	3.25E-11	2.47E-10
Pou3f4	8	72.02845165	54.78136358	-17.24708807	3.26E-11	2.48E-10
Tex101	21	89.388295	81.558774	-7.829520995	3.66E-11	2.78E-10
Gm17455	2	70.39969834	53.85738417	-16.54231417	3.88E-11	2.95E-10

Lrrtm2	5	67.5269957	86.8577868	19.3307911	3.92E-11	2.98E-10
Snap91	113	25.19284132	24.31999195	-0.872849372	3.97E-11	3.01E-10
Gm7325	13	77.38033438	70.87818038	-6.502154002	4.00E-11	3.03E-10
Gm1600	3	48.55288118	32.80884684	-15.74403435	4.11E-11	3.12E-10
Mmp3	1	92.88888889	68.5	-24.38888889	4.19E-11	3.17E-10
Wt1os	13	51.03311627	61.99292564	10.95980938	4.20E-11	3.18E-10
)33415F23F	14	91.61470783	82.96110673	-8.653601105	4.21E-11	3.19E-10
Abcb7	24	16.13331541	11.21934753	-4.913967881	4.26E-11	3.22E-10
Sox14	38	19.83948583	18.21696579	-1.622520044	4.28E-11	3.24E-10
Gucy2e	11	37.74748924	45.99575598	8.248266736	4.52E-11	3.42E-10
Olfr368	2	19.30430119	7.331844383	-11.9724568	4.54E-11	3.43E-10
Slc7a8	7	9.422101316	27.17878946	17.75668815	4.63E-11	3.50E-10
Fgf12	205	50.33285101	51.75139853	1.418547523	4.69E-11	3.54E-10
Fn3k	12	5.585854168	2.892131287	-2.693722881	4.70E-11	3.55E-10
Gabrg3	50	45.92409193	41.03967731	-4.884414626	4.86E-11	3.66E-10
Scrt1	47	11.67288096	8.511826287	-3.161054671	4.89E-11	3.69E-10
Tgm1	20	63.37350268	70.17564771	6.80214503	5.30E-11	3.99E-10
Apba1	51	3.805283837	2.547041324	-1.258242513	5.30E-11	3.99E-10
Neurod1	2	37.91208791	68.01730921	30.10522129	5.38E-11	4.05E-10
Pkd1l2	10	62.69776821	53.10321062	-9.594557583	5.52E-11	4.15E-10
Reep1	30	15.95162393	13.5028638	-2.448760123	5.56E-11	4.18E-10
Rhbdl1	6	17.23876079	9.90123172	-7.337529066	5.68E-11	4.26E-10
Cd19	4	43.50305421	30.42578287	-13.07727134	5.72E-11	4.29E-10
Slc16a10	29	2.479070209	1.087372269	-1.39169794	5.73E-11	4.30E-10
Tmem231	26	8.336062398	5.23810825	-3.097954148	5.76E-11	4.32E-10
Spata32	9	12.86535696	7.773145269	-5.092211689	6.16E-11	4.62E-10
Mir152	33	8.237416307	11.5225333	3.285116997	6.17E-11	4.63E-10
Npw	42	31.26216236	35.82453031	4.562367956	6.28E-11	4.71E-10
Stmn4	8	37.95098253	26.86873657	-11.08224595	6.36E-11	4.76E-10
Gm6583	6	91.10323776	81.07700394	-10.02623382	6.45E-11	4.82E-10
Gda	11	11.55608137	6.836153939	-4.719927432	6.58E-11	4.92E-10
Insl3	4	89.39331995	80.47968523	-8.913634716	6.60E-11	4.94E-10
Stra6	9	64.38431807	74.76976212	10.38544405	6.69E-11	5.00E-10
Pde5a	31	8.217685215	10.33434584	2.116660622	6.73E-11	5.03E-10

Spata18	21	12.8927424	8.935718003	-3.9570244	6.80E-11	5.07E-10
Htra3	25	31.40629606	36.98015403	5.573857968	6.89E-11	5.14E-10
Smim11	5	24.4083292	13.05855443	-11.34977476	6.95E-11	5.18E-10
Sstr5	17	9.957190909	4.917443072	-5.039747838	7.22E-11	5.38E-10
Tmem8	49	2.224862386	1.192220444	-1.032641943	7.27E-11	5.41E-10
Nfatc4	13	25.69630747	35.13092149	9.434614024	7.56E-11	5.63E-10
Lage3	12	22.43559982	15.0081908	-7.427409016	7.56E-11	5.63E-10
Lmo2	44	9.4311019	7.846118078	-1.584983821	7.58E-11	5.64E-10
933412E24F	2	98.29699003	81.86320755	-16.43378248	7.96E-11	5.91E-10
Rbm46	86	62.38788051	63.49146112	1.103580613	8.01E-11	5.95E-10
Sfmbt1	127	3.37072063	2.317958618	-1.052762011	8.36E-11	6.20E-10
Gpr162	29	3.324320721	2.029256578	-1.295064143	9.24E-11	6.86E-10
Cst12	2	68.51953468	45.75164555	-22.76788913	9.42E-11	6.99E-10
300002P13F	42	47.39483299	51.07707418	3.682241193	9.45E-11	7.01E-10
Gm19424	5	81.01656078	72.30585453	-8.710706249	9.55E-11	7.08E-10
Adgrb1	60	12.82276461	15.67972234	2.856957732	1.00E-10	7.44E-10
Rassf5	112	6.679545288	5.08618072	-1.593364567	1.06E-10	7.84E-10
Cyp2j6	15	27.32314122	34.93864267	7.61550145	1.07E-10	7.94E-10
Dock6	40	7.069863103	4.890006375	-2.179856729	1.08E-10	8.02E-10
Ndufc2	11	60.30807025	54.9947276	-5.313342647	1.12E-10	8.27E-10
Fshr	3	93.88185654	78.70327775	-15.17857879	1.13E-10	8.35E-10
Asphd2	1	66.36363636	25	-41.36363636	1.15E-10	8.49E-10
Adad2	11	85.26917249	75.64579001	-9.623382485	1.17E-10	8.65E-10
Doc2a	56	12.47556368	9.936878572	-2.53868511	1.23E-10	9.11E-10
Foxd2	87	2.683878635	1.589448517	-1.094430118	1.25E-10	9.20E-10
Zfp385a	29	3.788304373	7.172378684	3.384074312	1.25E-10	9.20E-10
Rgl1	44	1.486959149	3.1538599	1.666900751	1.28E-10	9.41E-10
Cbs	39	4.325737253	2.852685024	-1.473052229	1.28E-10	9.46E-10
Nrg2	51	2.265033603	1.351884757	-0.913148846	1.29E-10	9.51E-10
Gria2	23	46.8397791	41.94318838	-4.896590726	1.34E-10	9.85E-10
Caskin1	80	5.549807243	4.047818859	-1.501988384	1.35E-10	9.90E-10
Egflam	39	20.5945038	17.56922522	-3.025278581	1.37E-10	1.01E-09
Ftmt	6	81.75975863	65.54560759	-16.21415104	1.37E-10	1.01E-09
Vasn	5	14.08581277	3.805660345	-10.28015243	1.45E-10	1.06E-09

Mdga1	123	2.026238493	1.598389948	-0.427848545	1.47E-10	1.08E-09
Tmem121	18	2.776413152	1.503970693	-1.272442459	1.47E-10	1.08E-09
Fam189a2	99	3.404391074	2.687864866	-0.716526208	1.53E-10	1.12E-09
Wnt10a	15	10.63186866	6.562673973	-4.069194687	1.55E-10	1.13E-09
P2rx1	5	50.95359453	37.46298771	-13.49060682	1.61E-10	1.18E-09
Lurap1	20	5.517940518	2.289708882	-3.228231636	1.62E-10	1.18E-09
Gm14379	17	20.10461393	12.85684033	-7.247773601	1.69E-10	1.24E-09
Oasl1	10	33.58513298	24.92084439	-8.664288587	1.70E-10	1.25E-09
Slc44a4	9	56.07886963	63.99470513	7.915835501	1.72E-10	1.26E-09
Wnt2	35	14.15467545	19.2638447	5.109169248	1.74E-10	1.27E-09
Slc7a10	63	22.11536151	25.49991988	3.384558371	1.76E-10	1.29E-09
Ppp1r1a	44	11.32845737	15.88625394	4.557796573	1.79E-10	1.30E-09
Tgfb1i1	7	14.11069146	8.01589151	-6.094799954	1.83E-10	1.34E-09
Ppp1r2-ps3	9	64.90118672	73.38160385	8.480417129	1.90E-10	1.39E-09
Lpl	11	9.694110036	4.464962423	-5.229147613	1.91E-10	1.39E-09
Msantd3	113	2.893603139	1.792277697	-1.101325441	1.95E-10	1.42E-09
Fbln1	45	7.468165828	6.317067879	-1.151097949	1.99E-10	1.45E-09
930599N23F	55	19.75715632	23.69950871	3.942352386	2.00E-10	1.46E-09
Kcnq5	61	3.616927134	2.864167713	-0.752759421	2.16E-10	1.57E-09
Unc5c	75	48.92527957	52.27622461	3.350945045	2.17E-10	1.58E-09
Elk1	8	20.55310132	12.61224787	-7.940853451	2.18E-10	1.59E-09
Zfa-ps	16	89.92698268	81.63682385	-8.290158836	2.19E-10	1.59E-09
Mrc2	15	11.34233832	17.16225203	5.819913711	2.20E-10	1.60E-09
Tmem28	113	46.84823573	51.41161104	4.563375316	2.21E-10	1.61E-09
Mycbpap	16	13.42825418	7.22271549	-6.205538693	2.24E-10	1.62E-09
Morc2b	5	65.66195286	43.9729575	-21.68899536	2.36E-10	1.71E-09
Dok6	55	46.12389067	42.04553641	-4.07835426	2.36E-10	1.71E-09
Mir6948	17	93.03190562	88.26839795	-4.763507672	2.37E-10	1.71E-09
Sox10	5	38.73498886	27.00776998	-11.72721888	2.37E-10	1.71E-09
Gabra1	3	42.87662338	62.5415835	19.66496012	2.43E-10	1.76E-09
Sigirr	7	9.913398398	3.728430743	-6.184967654	2.44E-10	1.76E-09
Shroom2	65	18.35034952	14.75572061	-3.594628917	2.49E-10	1.80E-09
Hspa1a	9	6.743686782	2.875468836	-3.868217946	2.50E-10	1.81E-09
Sez6	91	6.620384113	5.676163983	-0.94422013	2.58E-10	1.87E-09

Fam132a	7	29.10535365	41.12566554	12.02031189	2.61E-10	1.88E-09
Cldn13	2	78.47082495	54.60752953	-23.86329542	2.65E-10	1.91E-09
Hrc	3	93.24004505	79.7050938	-13.53495125	2.65E-10	1.91E-09
Ikzf1	127	32.31443594	30.98724245	-1.327193482	2.72E-10	1.96E-09
Spon1	31	13.59077259	19.63949268	6.048720094	2.75E-10	1.98E-09
Tmem151b	50	7.516561433	10.15347906	2.636917629	2.77E-10	1.99E-09
Hoxb7	63	26.6053296	24.57027616	-2.035053449	2.78E-10	2.00E-09
Nkx3-1	25	23.45701518	18.2897605	-5.167254677	2.86E-10	2.06E-09
L1td1	17	26.78495101	33.87637175	7.091420734	2.87E-10	2.07E-09
Mir129-2	12	8.557877367	3.733143843	-4.824733524	2.89E-10	2.08E-09
Gpc4	41	36.46533693	31.38519501	-5.080141927	2.93E-10	2.10E-09
530026F06R	2	95.49595142	71.57394844	-23.92200298	3.09E-10	2.21E-09
Klhdc7b	11	56.57299057	46.33018574	-10.24280483	3.09E-10	2.22E-09
Dync1i1	12	42.59433854	31.20080898	-11.39352956	3.16E-10	2.27E-09
Nxph1	19	35.98886807	41.42195236	5.433084294	3.19E-10	2.28E-09
Hivep3	7	7.862984238	16.81997786	8.956993621	3.20E-10	2.29E-09
700024P16R	13	26.60399192	20.34153915	-6.262452771	3.23E-10	2.31E-09
Slc35c2	40	5.92107216	3.048597878	-2.872474283	3.30E-10	2.36E-09
700051A21R	10	22.97054047	36.70614035	13.73559988	3.35E-10	2.40E-09
Aif1l	63	2.365171599	1.626978497	-0.738193102	3.49E-10	2.50E-09
Hsf4	52	3.161020446	1.998843292	-1.162177154	3.54E-10	2.53E-09
Acot11	7	39.13050646	48.59322976	9.462723303	3.60E-10	2.57E-09
Prelid2	8	11.99778388	7.010143225	-4.987640653	3.84E-10	2.74E-09
Krt10	8	90.42300262	94.88947051	4.466467884	3.92E-10	2.79E-09
Esr1	9	26.57316651	19.22249773	-7.350668776	3.96E-10	2.83E-09
Ina	25	31.87387061	36.11292377	4.239053161	4.05E-10	2.89E-09
Phf8	50	21.20235609	16.59325006	-4.609106038	4.05E-10	2.89E-09
Pcdhga10	10	69.52423576	78.00251945	8.47828369	4.07E-10	2.90E-09
Huwe1	23	21.43919811	15.71667103	-5.722527082	4.10E-10	2.91E-09
Enox2	19	28.36136064	21.34918571	-7.012174922	4.14E-10	2.95E-09
Lrrn2	89	17.92085647	16.62997467	-1.290881796	4.19E-10	2.98E-09
Rtn4rl2	50	9.627299036	6.953172698	-2.674126338	4.22E-10	3.00E-09
Slc1a3	13	10.17453475	4.670222253	-5.504312492	4.30E-10	3.05E-09
Olfr734	2	87.98198198	59.936967	-28.04501498	4.32E-10	3.07E-09

Calhm1	11	77.09091653	66.74857942	-10.34233712	4.40E-10	3.12E-09
Adamtsl5	52	9.46962042	6.709401047	-2.760219373	4.40E-10	3.12E-09
Anks1b	49	5.603679774	7.69715621	2.093476436	4.40E-10	3.12E-09
Ube2a	54	19.80761673	16.10266642	-3.704950309	4.45E-10	3.15E-09
H1fx	68	4.302749438	7.194451952	2.891702513	4.45E-10	3.15E-09
Chrna4	72	8.085582927	10.51444885	2.428865923	4.51E-10	3.19E-09
Sec16b	6	48.64257992	33.38050393	-15.26207599	4.54E-10	3.21E-09
Jag2	123	4.515606218	2.722531098	-1.793075121	4.55E-10	3.22E-09
BB283400	35	15.17848481	20.91131165	5.732826839	4.61E-10	3.26E-09
Rps6ka3	52	21.10262543	17.57506612	-3.527559307	4.66E-10	3.29E-09
Col24a1	3	34.10858332	45.11206515	11.00348182	4.99E-10	3.52E-09
Evpl	25	16.49340388	12.69157548	-3.801828396	5.03E-10	3.55E-09
Mcoln2	42	11.09983241	13.97903751	2.879205096	5.06E-10	3.57E-09
Acsm3	3	52.88914344	30.18994415	-22.69919929	5.09E-10	3.59E-09
430430B14R	5	58.38125591	44.80565329	-13.57560262	5.11E-10	3.60E-09
Enpp6	1	61.23595506	31.02040816	-30.21554689	5.17E-10	3.64E-09
Lypd2	2	83.96890041	64.0625	-19.90640041	5.26E-10	3.70E-09
Ercc6l	20	20.43964697	13.47710287	-6.962544105	5.29E-10	3.72E-09
Ube2u	8	30.92904573	19.6443954	-11.28465033	5.38E-10	3.79E-09
Vav1	2	18.79330508	4.839109935	-13.95419515	5.42E-10	3.81E-09
Slitrk4	34	48.76640405	55.13497742	6.368573376	5.48E-10	3.85E-09
Tnp1	2	71.5743252	44.41113719	-27.16318801	5.56E-10	3.90E-09
Vwce	9	14.82122049	20.9388445	6.117624012	5.56E-10	3.91E-09
330179D12R	7	5.135630487	0.924036281	-4.211594206	5.57E-10	3.91E-09
Cdh2	101	20.22240551	22.40121125	2.178805737	5.65E-10	3.96E-09
Mir6910	7	89.04815531	81.68806127	-7.360094047	5.73E-10	4.01E-09
Nudt7	5	7.512600469	2.254956629	-5.25764384	5.84E-10	4.09E-09
Slc16a9	30	6.808839403	4.342647033	-2.46619237	5.86E-10	4.10E-09
700123K08R	2	62.17818428	38.46486888	-23.71331541	6.01E-10	4.21E-09
Dppa2	3	93.26843354	82.01903354	-11.2494	6.41E-10	4.48E-09
Dmgdh	15	66.35526116	72.26967177	5.914410612	6.48E-10	4.54E-09
Plxnc1	142	10.45221662	8.788820815	-1.663395806	6.50E-10	4.54E-09
Igbp1b	15	87.54172973	80.13836173	-7.403368004	6.69E-10	4.68E-09
Baiap3	10	6.137678563	13.45759057	7.319912003	7.17E-10	5.01E-09

Hand2	9	66.22719519	52.42243025	-13.80476493	7.38E-10	5.15E-09
Cdhr5	6	57.14818505	44.67707945	-12.4711056	7.41E-10	5.17E-09
Kcnd3os	43	29.64046552	27.16026289	-2.480202632	7.43E-10	5.18E-09
Zfp804b	8	15.04459734	11.87296292	-3.171634417	7.84E-10	5.47E-09
930402G23F	5	46.33474119	35.03587832	-11.29886287	7.86E-10	5.48E-09
Cacna1a	88	7.939243717	7.392073452	-0.547170265	8.08E-10	5.62E-09
Cables1	13	9.589923013	4.191615583	-5.398307429	8.18E-10	5.69E-09
921504A21F	30	25.59658846	30.73676354	5.140175076	8.26E-10	5.75E-09
Sprn	11	50.88258171	39.65565602	-11.22692569	8.27E-10	5.75E-09
Jph3	147	9.338005233	11.57605656	2.238051327	8.45E-10	5.87E-09
Gzmm	1	90.49180328	72.56857855	-17.92322473	8.58E-10	5.96E-09
Jade3	102	18.28396916	15.54210412	-2.741865035	8.79E-10	6.10E-09
Drd5	43	26.50292431	29.28053776	2.777613454	8.94E-10	6.20E-09
B3galt5	6	11.91669632	8.786509003	-3.130187313	9.18E-10	6.37E-09
Tcf21	1	45.52238806	13.66459627	-31.85779179	9.21E-10	6.39E-09
Cpne7	70	17.39100853	19.08181334	1.690804816	9.51E-10	6.59E-09
Cfap45	4	31.26137848	44.05383388	12.7924554	9.53E-10	6.60E-09
Mir7218	1	67.0212766	34.9112426	-32.11003399	9.68E-10	6.70E-09
Tnfsf14	7	47.89900407	35.50685932	-12.39214475	9.71E-10	6.72E-09
Fam83e	4	45.21224813	31.69213721	-13.52011092	1.00E-09	6.95E-09
230102O04F	8	31.56134004	45.89865752	14.33731748	1.01E-09	6.99E-09
Lrfn1	87	4.704811381	4.198895353	-0.505916028	1.04E-09	7.21E-09
Nkx1-2	14	23.74629592	20.45191169	-3.29438423	1.06E-09	7.32E-09
Oprm1	6	35.48882345	24.05121044	-11.43761301	1.07E-09	7.36E-09
4-Sep	22	7.646033243	4.405400682	-3.240632561	1.08E-09	7.46E-09
Ndst3	46	60.57243485	63.64113143	3.068696575	1.09E-09	7.49E-09
Ap1s2	67	25.07264916	22.46160703	-2.611042131	1.09E-09	7.53E-09
Il10ra	6	11.71171171	2.31884058	-9.392871132	1.11E-09	7.64E-09
310007B03F	4	46.81672595	34.20941208	-12.60731387	1.13E-09	7.75E-09
Grhl2	27	12.24202823	16.43242829	4.190400063	1.15E-09	7.95E-09
930013I19R	3	63.338899	48.87897298	-14.45992601	1.18E-09	8.10E-09
Cyp1a1	2	15.53616039	5.612980769	-9.92317962	1.18E-09	8.12E-09
Mir6917	9	73.38002981	62.69960074	-10.68042907	1.21E-09	8.29E-09
Nsg2	12	10.08734077	15.78509944	5.697758674	1.22E-09	8.37E-09

AI427809	3	88.97291405	77.58839601	-11.38451804	1.22E-09	8.39E-09
Nespas	4	11.8852459	0.912910619	-10.97233528	1.23E-09	8.46E-09
Hesx1	7	71.30253871	58.13843826	-13.16410045	1.25E-09	8.61E-09
Zcchc14	195	3.259385887	2.626450617	-0.63293527	1.27E-09	8.73E-09
Robo4	1	57.14285714	26.34146341	-30.80139373	1.28E-09	8.77E-09
Mir6354	1	98.41269841	61.76470588	-36.64799253	1.31E-09	8.97E-09
Uggt2	41	34.05853613	30.55920772	-3.499328414	1.31E-09	8.98E-09
Snx19	33	3.407753499	1.998947424	-1.408806075	1.31E-09	9.00E-09
Eya4	43	37.44072436	41.57619263	4.135468266	1.33E-09	9.08E-09
Iqsec2	48	14.17342192	10.17288146	-4.000540461	1.36E-09	9.28E-09
Rn45s	55	41.30523533	41.23908707	-0.066148257	1.37E-09	9.36E-09
Dhtkd1	13	7.605363308	3.580224769	-4.025138539	1.41E-09	9.66E-09
'30006G06F	10	88.67787514	78.74321055	-9.934664591	1.43E-09	9.77E-09
Cpb1	4	84.25688678	68.47325785	-15.78362894	1.45E-09	9.89E-09
'00016K19F	4	19.30954952	12.31629719	-6.993252337	1.45E-09	9.89E-09
)33404K13F	6	96.68126471	91.36220841	-5.3190563	1.45E-09	9.89E-09
Cd34	17	28.92998839	34.42458567	5.494597275	1.54E-09	1.05E-08
Jph2	2	60.74113856	39.64711988	-21.09401869	1.56E-09	1.07E-08
Azin2	53	5.452963929	2.786160129	-2.6668038	1.62E-09	1.10E-08
Celsr1	100	6.465611181	5.394949019	-1.070662161	1.69E-09	1.15E-08
Mgarp	14	20.67991951	26.24911159	5.569192079	1.71E-09	1.16E-08
Slc25a34	5	86.35989509	76.88713195	-9.472763139	1.75E-09	1.19E-08
Rhof	27	10.53245325	5.755499348	-4.776953907	1.79E-09	1.22E-08
Nog	112	13.22290489	16.93954339	3.716638496	1.80E-09	1.22E-08
Eln	10	4.857425516	2.372395964	-2.485029552	1.82E-09	1.24E-08
Eno2	25	12.91130293	18.33280435	5.42150142	1.85E-09	1.26E-08
Gpr179	11	93.34599238	88.96226168	-4.383730695	1.88E-09	1.27E-08
Trpm6	29	62.36360984	68.39347316	6.029863321	1.92E-09	1.30E-08
Ltbp3	2	40.87878788	23.04421769	-17.83457019	1.95E-09	1.32E-08
Prkx	32	22.14015912	17.21104467	-4.929114454	1.96E-09	1.33E-08
Mlph	15	6.039240082	3.702506948	-2.336733134	1.97E-09	1.33E-08
Klhl14	15	28.49596143	23.30342118	-5.192540248	1.97E-09	1.33E-08
Gria4	12	34.6042325	25.30937722	-9.294855275	1.98E-09	1.34E-08
Mir6901	2	93.16239316	78.40899796	-14.75339521	2.09E-09	1.42E-08

Fam83a	7	66.12489895	56.74910146	-9.375797491	2.10E-09	1.42E-08
Tomm20l	5	39.83349983	22.82513661	-17.00836322	2.18E-09	1.48E-08
933438B17R	3	86.12136995	67.74082826	-18.38054169	2.20E-09	1.49E-08
Ubqln2	53	16.69120266	12.39062346	-4.300579197	2.28E-09	1.54E-08
Wbscr25	10	23.60330762	30.9797152	7.376407585	2.31E-09	1.56E-08
430010I23R	8	18.52685092	12.03101773	-6.495833191	2.40E-09	1.62E-08
Ssh3	21	6.424589089	3.426750714	-2.997838375	2.42E-09	1.63E-08
Zfp462	15	9.809605001	3.937330398	-5.872274604	2.47E-09	1.67E-08
Armcx2	5	30.9862056	18.5464689	-12.4397367	2.53E-09	1.70E-08
Dlg4	10	17.76672119	13.05495003	-4.711771158	2.56E-09	1.72E-08
Mir6926	14	91.64411919	85.34361806	-6.300501127	2.64E-09	1.77E-08
933431E20R	24	6.468166567	10.84463834	4.376471774	2.64E-09	1.78E-08
Wfdc1	5	14.70230678	7.092999947	-7.609306837	2.72E-09	1.83E-08
921509C19R	3	57.20419412	42.86807155	-14.33612256	2.73E-09	1.83E-08
Slc7a14	24	45.6919252	42.60003929	-3.091885914	2.78E-09	1.87E-08
Kdm5c	26	2.530213933	1.119260534	-1.410953399	2.81E-09	1.88E-08
Rbmx2	25	23.73416995	18.10176888	-5.632401073	2.87E-09	1.93E-08
Rltpr	6	80.18252372	69.06516122	-11.1173625	2.89E-09	1.94E-08
Fermt2	110	2.446066556	1.651471843	-0.794594713	2.94E-09	1.97E-08
Sp7	4	45.00698374	31.28062626	-13.72635748	2.95E-09	1.97E-08
Map3k15	62	39.43259284	44.32314105	4.890548212	2.95E-09	1.98E-08
Jph4	2	24.29054471	40.13413003	15.84358531	2.99E-09	2.00E-08
Gm12830	8	25.63784842	17.39943769	-8.238410732	3.03E-09	2.03E-08
Itga7	23	6.64545573	11.64592722	5.000471493	3.03E-09	2.03E-08
Gm19668	6	66.73874985	58.35303038	-8.385719466	3.04E-09	2.03E-08
Hoxd10	36	57.97464443	52.42185742	-5.552787011	3.06E-09	2.05E-08
Nxph4	54	3.255782837	1.871447386	-1.384335451	3.06E-09	2.05E-08
Gm10857	9	94.08093094	83.64522401	-10.43570693	3.13E-09	2.09E-08
Gramd1b	31	15.92445973	12.82519798	-3.099261753	3.15E-09	2.10E-08
Clec14a	4	57.68706309	45.10273953	-12.58432355	3.15E-09	2.10E-08
Chn1	96	4.071883525	2.95447512	-1.117408405	3.21E-09	2.14E-08
Cbr1	7	2.874751241	6.451537892	3.576786651	3.21E-09	2.14E-08
Megf10	17	18.02184653	14.78637486	-3.235471665	3.23E-09	2.15E-08
Gm15455	12	90.38965736	81.7746026	-8.615054758	3.23E-09	2.15E-08

Rps4x	14	19.5990883	12.56720491	-7.031883398	3.27E-09	2.17E-08
Adad1	27	83.22667147	79.99608988	-3.230581594	3.28E-09	2.18E-08
Gpr83	45	27.67534662	25.95160011	-1.723746514	3.34E-09	2.22E-08
Dkk2	9	15.46501456	11.81854673	-3.646467834	3.35E-09	2.23E-08
Hebp1	27	3.930974876	2.262975847	-1.667999029	3.46E-09	2.30E-08
Sall4	50	25.61120405	22.82579115	-2.785412904	3.49E-09	2.32E-08
Slc6a8	57	16.35113803	13.48851476	-2.862623273	3.51E-09	2.33E-08
[illegible]30594O21[illegible]	9	82.92168396	76.83996196	-6.081721993	3.54E-09	2.35E-08
C1ql2	38	35.94536714	32.33026662	-3.615100525	3.58E-09	2.37E-08
Cct8l1	8	95.56130347	88.56170245	-6.999601024	3.58E-09	2.38E-08
Adgrb3	74	51.39255933	53.46168225	2.069122923	3.60E-09	2.38E-08
Spats2l	66	2.732080665	1.728487252	-1.003593413	3.60E-09	2.39E-08
Mir455	16	95.20590846	90.6602871	-4.54562136	3.65E-09	2.42E-08
Slc43a1	58	13.65734523	13.16272306	-0.494622166	3.71E-09	2.45E-08
Pfn4	6	25.86623753	18.52640415	-7.339833381	3.80E-09	2.51E-08
Spanxn4	3	87.12121212	62.3546362	-24.76657592	3.84E-09	2.54E-08
Mvb12b	35	2.762038946	1.376084614	-1.385954332	3.90E-09	2.58E-08
Nox4	39	45.18620036	42.08748936	-3.098710992	3.91E-09	2.58E-08
Pcdhga1	10	59.67068707	51.72621297	-7.944474106	4.04E-09	2.67E-08
[illegible]30426P16[illegible]	30	3.55282816	2.092721669	-1.460106491	4.10E-09	2.71E-08
Scarf1	3	65.07899098	47.37212193	-17.70686905	4.24E-09	2.80E-08
Igfbp7	94	2.477205347	1.640022082	-0.837183265	4.37E-09	2.88E-08
Hmx1	135	58.05771522	60.47531253	2.417597308	4.38E-09	2.88E-08
Slc13a3	21	24.71526915	21.77803773	-2.937231413	4.50E-09	2.96E-08
Elavl4	45	45.87021952	49.44633223	3.576112714	4.52E-09	2.97E-08
Slc2a5	2	98.66666667	75.02560164	-23.64106503	4.55E-09	3.00E-08
Lamp2	25	19.85864907	12.44309388	-7.415555192	4.58E-09	3.01E-08
Dll4	13	4.246294708	1.410803434	-2.835491274	4.60E-09	3.02E-08
Kcnd2	21	36.49734195	33.30737916	-3.189962787	4.66E-09	3.06E-08
Cldn17	5	97.9770807	92.34593234	-5.631148363	4.81E-09	3.16E-08
Maf	84	2.443274532	1.57423356	-0.869040972	4.82E-09	3.17E-08
Lrrc2	3	90.31080031	75.41708375	-14.89371656	4.83E-09	3.17E-08
Arc	36	9.240734107	7.266417672	-1.974316435	5.03E-09	3.30E-08
Naaladl1	3	72.6570176	59.84471581	-12.81230179	5.15E-09	3.38E-08

Nsg1	78	36.48091583	34.95398333	-1.526932494	5.16E-09	3.38E-08
Nrip2	4	54.29066394	40.43685518	-13.85380876	5.32E-09	3.49E-08
Prss39	3	78.87238932	67.41023311	-11.46215621	5.41E-09	3.55E-08
Gm16157	12	37.21882572	28.78902084	-8.429804883	5.44E-09	3.56E-08
Adgra3	99	2.71533495	1.913444938	-0.801890012	5.65E-09	3.69E-08
Celf3	4	25.47176742	40.80737302	15.3356056	5.69E-09	3.72E-08
Prps2	31	22.63759436	17.99564447	-4.641949898	5.70E-09	3.73E-08
Nme8	5	81.58628609	66.6308184	-14.95546769	5.75E-09	3.76E-08
.30060K24R	16	23.69840583	19.25958438	-4.438821449	5.83E-09	3.81E-08
P2rx6	7	6.507785822	2.137878629	-4.369907193	6.13E-09	4.00E-08
Atp11c	136	16.79066031	14.52944636	-2.261213957	6.21E-09	4.05E-08
Trappc2	34	16.71683537	12.74815206	-3.968683309	6.22E-09	4.06E-08
Nptx1	58	2.618680192	1.079924797	-1.538755394	6.27E-09	4.09E-08
700021F07R	3	87.80063291	69.99228841	-17.8083445	6.29E-09	4.10E-08
Sntg2	27	39.57965096	45.59156775	6.011916794	6.29E-09	4.10E-08
Aqp4	6	85.7494362	71.71333783	-14.03609837	6.33E-09	4.12E-08
Prss42	15	90.75893584	82.37815533	-8.380780506	6.37E-09	4.15E-08
930545E07R	1	91.17647059	48	-43.17647059	6.49E-09	4.23E-08
Fbxl22	4	69.96747171	81.37337921	11.4059075	6.62E-09	4.30E-08
Rag1	1	69.71608833	46.03773585	-23.67835248	6.65E-09	4.32E-08
Rhox6	3	94.94636964	84.96541054	-9.9809591	6.79E-09	4.41E-08
Grid2	62	37.27340977	39.62481926	2.351409484	6.83E-09	4.43E-08
Fam151b	6	8.556196619	4.286506385	-4.269690234	6.84E-09	4.44E-08
Hmgb3	11	28.060385	19.17219509	-8.888189913	6.85E-09	4.44E-08
Pla2g3	9	51.60732286	44.33521848	-7.272104379	6.85E-09	4.45E-08
Guca1b	3	98.39845827	88.84433226	-9.554126012	7.07E-09	4.58E-08
Mylip	62	17.14087397	15.62467544	-1.516198539	7.07E-09	4.58E-08
Sycp1	38	91.12959716	87.67547149	-3.454125661	7.19E-09	4.66E-08
Mertk	37	5.172587182	6.464537294	1.291950112	7.22E-09	4.67E-08
Ak8	30	7.988519149	5.653590578	-2.334928571	7.39E-09	4.78E-08
Has3	22	30.63750863	27.00767155	-3.629837076	7.40E-09	4.79E-08
Fxyd6	15	7.64296843	12.04846404	4.405495613	7.61E-09	4.92E-08
Fmnl1	113	1.909669745	1.466994703	-0.442675042	7.62E-09	4.93E-08
Irak1bp1	41	2.125438289	1.135656657	-0.989781632	8.75E-09	5.65E-08

Acat3	5	8.161459954	4.154331389	-4.007128566	8.79E-09	5.68E-08
Phldb3	36	3.721140525	2.324510672	-1.396629853	8.81E-09	5.68E-08
Bcat1	21	21.46881743	17.83928695	-3.629530478	8.86E-09	5.72E-08
Bend5	49	45.58339669	49.11592976	3.532533072	8.92E-09	5.76E-08
Dazl	37	94.57135309	90.88894358	-3.682409513	8.99E-09	5.80E-08
Tmtc1	66	16.32394343	15.06990409	-1.254039348	9.12E-09	5.88E-08
Pcp4l1	38	15.30232773	11.2928095	-4.00951824	9.24E-09	5.96E-08
B3gnt8	14	10.42672894	15.71595084	5.289221898	9.26E-09	5.96E-08
Dapp1	9	96.95557547	94.1771719	-2.778403566	9.26E-09	5.96E-08
Epcam	3	5.04290285	16.49484536	11.45194251	9.39E-09	6.04E-08
Syt16	29	21.55377857	18.31791245	-3.23586612	9.49E-09	6.10E-08
Arhgap42	39	24.05278999	28.88727023	4.834480243	9.51E-09	6.11E-08
B3gnt7	47	6.994183677	6.464035339	-0.530148338	9.60E-09	6.17E-08
Zfp180	11	44.36667955	37.78791268	-6.578766869	9.65E-09	6.20E-08
Saa2	1	66.31578947	26	-40.31578947	1.00E-08	6.43E-08
)30003O13F	10	33.13796037	27.11199691	-6.025963467	1.01E-08	6.49E-08
Igdcc3	50	13.28163643	17.80275508	4.521118652	1.01E-08	6.50E-08
)30433N12F	14	60.6070128	66.94355486	6.336542068	1.02E-08	6.56E-08
Ptf1a	29	29.26206173	25.43387852	-3.828183213	1.04E-08	6.68E-08
Mir124a-2	13	41.34660347	37.38931405	-3.95728942	1.06E-08	6.77E-08
Sstr3	8	36.55417286	27.64032509	-8.91384777	1.07E-08	6.88E-08
Lhx4	82	8.23433166	6.800501817	-1.433829842	1.08E-08	6.92E-08
'30460C07F	1	92.11045365	79.96219282	-12.14826083	1.10E-08	7.04E-08
Ddo	4	58.82525599	40.66456835	-18.16068764	1.10E-08	7.05E-08
Foxl2os	48	9.674670699	12.93299122	3.25832052	1.11E-08	7.12E-08
Sowahd	57	28.60241528	26.14264992	-2.459765361	1.15E-08	7.35E-08
Dsg2	29	2.794915507	1.640263581	-1.154651927	1.16E-08	7.40E-08
Rab39	2	23.05341055	44.41176471	21.35835415	1.17E-08	7.49E-08
Stk32c	82	13.65779837	12.82581848	-0.831979892	1.18E-08	7.50E-08
Mir184	1	44.81481481	23.00884956	-21.80596526	1.18E-08	7.53E-08
Ntn1	84	12.8842356	11.25976654	-1.624469062	1.20E-08	7.62E-08
Cnr1	92	40.1953381	44.28672879	4.091390694	1.20E-08	7.63E-08
.30114P18F	6	92.4521442	85.58892449	-6.86321971	1.22E-08	7.74E-08
)10001E11F	27	29.76053354	25.11757602	-4.64295752	1.22E-08	7.78E-08

Alx3	56	55.36099426	58.02949557	2.668501303	1.24E-08	7.91E-08
Rell2	18	4.926802403	12.6061697	7.679367297	1.26E-08	8.04E-08
Tcerg1l	107	45.31748252	47.6103159	2.292833382	1.27E-08	8.09E-08
Mir615	43	31.05929855	34.69918495	3.639886393	1.28E-08	8.13E-08
Agbl4	10	52.91138243	47.25029868	-5.66108375	1.28E-08	8.15E-08
Wnt8a	2	85.90909091	62.5	-23.40909091	1.30E-08	8.25E-08
Csta1	2	70.90746055	51.93749738	-18.96996316	1.35E-08	8.55E-08
Stard8	3	36.06557377	10.9800363	-25.08553747	1.35E-08	8.56E-08
Cd48	2	45.41284404	24.98436034	-20.4284837	1.35E-08	8.57E-08
Tnxb	5	86.56915213	77.61719224	-8.951959894	1.36E-08	8.61E-08
Neb	7	93.92661505	85.84646252	-8.080152522	1.36E-08	8.64E-08
Dbn1	94	2.644809135	1.991225169	-0.653583967	1.37E-08	8.66E-08
Pacsin3	44	19.90998501	23.41866086	3.508675847	1.37E-08	8.69E-08
Osbp2	27	15.08951107	13.27235082	-1.817160249	1.37E-08	8.69E-08
Trpv6	3	51.19892743	32.07334288	-19.12558456	1.37E-08	8.69E-08
Mdfi	33	4.357659263	2.822007295	-1.535651969	1.42E-08	8.98E-08
Ascl2	37	16.91533406	14.80557736	-2.109756698	1.44E-08	9.13E-08
30011O05R	10	38.54444008	29.49669219	-9.047747897	1.46E-08	9.19E-08
Sat2	6	7.566940221	3.283746687	-4.283193535	1.47E-08	9.27E-08
Dcst1	6	91.77836936	83.27085831	-8.507511045	1.49E-08	9.42E-08
Edar	5	11.72070107	5.769450311	-5.951250759	1.52E-08	9.59E-08
Prdm6	114	10.99027293	13.52080367	2.530530744	1.55E-08	9.77E-08
Gatm	15	51.51813718	48.00393799	-3.51419919	1.56E-08	9.82E-08
Pcdhga11	12	66.93952859	75.54622577	8.606697177	1.58E-08	9.97E-08
110039I08R	8	30.00277835	38.59918396	8.596405612	1.64E-08	1.03E-07
Mir181c	1	91.02040816	72.10144928	-18.91895889	1.64E-08	1.03E-07
Gdf2	5	64.85916333	53.69085846	-11.16830487	1.64E-08	1.03E-07
Pop5	42	8.248754319	5.950578281	-2.298176037	1.64E-08	1.03E-07
Crabp1	13	37.23249319	31.44133687	-5.791156315	1.64E-08	1.03E-07
Mir5130	67	29.41319882	27.26789342	-2.145305397	1.66E-08	1.04E-07
Rfx6	10	17.96288538	27.4828236	9.519938211	1.66E-08	1.04E-07
Mir7027	4	97.523785	83.79147813	-13.73230687	1.67E-08	1.05E-07
Bhlhe22	23	54.98028211	59.47034651	4.490064401	1.68E-08	1.05E-07
B4galnt3	45	4.866982437	3.643557215	-1.223425222	1.72E-08	1.08E-07

Dnajb3	20	43.74984426	51.07060586	7.320761597	1.74E-08	1.09E-07
Ogdhl	11	14.1259184	22.31414249	8.188224099	1.74E-08	1.09E-07
Coq4	12	4.221774246	1.472858306	-2.74891594	1.78E-08	1.12E-07
Synpo2	4	18.17258115	9.333011848	-8.839569298	1.80E-08	1.13E-07
Pabpc4l	28	17.43647531	20.70113713	3.264661822	1.83E-08	1.15E-07
Kit	12	21.77188928	16.53054731	-5.241341967	1.86E-08	1.16E-07
210011C24F	56	9.460019412	9.220035381	-0.23998403	1.87E-08	1.17E-07
Fgb	5	82.08223972	69.04695755	-13.03528217	1.90E-08	1.19E-07
Tbx21	13	9.285367118	13.16762674	3.882259626	2.06E-08	1.28E-07
Pkp1	28	6.068291834	3.101227024	-2.96706481	2.10E-08	1.31E-07
Ramp2	53	8.181901803	5.999618055	-2.182283748	2.12E-08	1.33E-07
Snrpn	9	54.95332923	42.83094176	-12.12238747	2.14E-08	1.33E-07
Snurf	9	54.95332923	42.83094176	-12.12238747	2.14E-08	1.33E-07
Pirt	6	66.00057817	54.65280385	-11.34777431	2.15E-08	1.34E-07
Vax2os	21	21.46459006	27.62897699	6.164386928	2.17E-08	1.35E-07
Cyp4f40	3	61.03529087	42.90463933	-18.13065154	2.17E-08	1.35E-07
Tst	46	4.50350881	2.388079305	-2.115429505	2.18E-08	1.36E-07
Ebf2	20	45.08851215	50.13942374	5.050911588	2.22E-08	1.38E-07
Dpp6	46	54.98830411	50.71101716	-4.277286955	2.28E-08	1.42E-07
Dlk2	27	3.955253121	1.885703333	-2.069549788	2.29E-08	1.42E-07
Fibcd1	61	6.426772653	4.946378301	-1.480394352	2.30E-08	1.43E-07
Dlx5	6	24.91099243	13.23190951	-11.67908292	2.32E-08	1.44E-07
Fam19a4	13	25.30063371	31.96967623	6.669042515	2.38E-08	1.48E-07
Nalcn	15	40.58083123	49.66557911	9.084747876	2.40E-08	1.49E-07
Sox9	50	3.30514259	1.752633868	-1.552508721	2.41E-08	1.49E-07
Hoxa5	45	87.67684978	90.44229765	2.765447876	2.44E-08	1.52E-07
Dok7	44	2.558735883	1.479812656	-1.078923228	2.52E-08	1.56E-07
Slc9a6	25	19.10435834	13.29453055	-5.80982779	2.55E-08	1.58E-07
Fgf16	55	33.87148482	30.5882289	-3.283255916	2.58E-08	1.60E-07
Smoc1	44	30.72664374	34.60365576	3.877012023	2.59E-08	1.61E-07
Celsr2	74	7.25899982	5.660946657	-1.598053163	2.63E-08	1.63E-07
AU023762	3	78.52475573	65.35062982	-13.17412592	2.73E-08	1.69E-07
Ppp2r2c	117	8.337078403	8.574180994	0.237102591	2.74E-08	1.69E-07
Tmem30b	91	47.59471054	49.83481484	2.240104298	2.74E-08	1.70E-07

430105I19R	14	14.39787833	8.742582673	-5.655295656	2.77E-08	1.71E-07
Fscn2	11	90.08664974	86.24611774	-3.840532003	2.78E-08	1.72E-07
Lrrc30	1	58.33333333	24.54545455	-33.78787879	2.78E-08	1.72E-07
Angptl6	1	98.9010989	83.79888268	-15.10221622	2.79E-08	1.72E-07
Pdzrn4	25	50.47862025	54.90441472	4.425794478	2.89E-08	1.78E-07
Prrg3	41	28.86891964	25.08658724	-3.782332406	2.91E-08	1.80E-07
Ifitm10	34	4.292622451	2.225757197	-2.066865255	2.92E-08	1.80E-07
Armcx5	25	22.93495282	17.76703264	-5.167920173	3.00E-08	1.85E-07
Gprasp1	25	22.93495282	17.76703264	-5.167920173	3.00E-08	1.85E-07
Mir466n	2	60.52320828	35.37699434	-25.14621395	3.03E-08	1.86E-07
Nol3	8	7.1467404	2.755020585	-4.391719814	3.08E-08	1.89E-07
930547E08R	1	73.75	32.25806452	-41.49193548	3.13E-08	1.93E-07
Bscl2	10	3.367615157	1.467713762	-1.899901395	3.16E-08	1.94E-07
Mir125a	5	84.51147679	74.32525788	-10.18621891	3.20E-08	1.96E-07
Frs3os	4	74.15014498	65.101747	-9.048397983	3.21E-08	1.97E-07
Cldn18	5	91.58606446	96.37882794	4.79276348	3.24E-08	1.99E-07
Spag17	7	17.79195003	15.28231351	-2.509636517	3.26E-08	2.00E-07
Mir7080	2	76.50713583	53.625	-22.88213583	3.34E-08	2.05E-07
Prdm16	121	2.418173677	1.667268961	-0.750904716	3.41E-08	2.09E-07
Neil1	5	93.71306878	85.86932215	-7.843746627	3.42E-08	2.10E-07
Pcdha1	3	46.29756209	68.59774213	22.30018004	3.46E-08	2.12E-07
Rapgef3	40	3.759052802	2.551598234	-1.207454568	3.46E-08	2.12E-07
Fstl4	88	5.935294957	5.541628725	-0.393666232	3.54E-08	2.17E-07
Arhgap24	18	13.3721948	21.06714055	7.694945743	3.60E-08	2.21E-07
Hoxa10	26	36.96511039	34.67504601	-2.290064376	3.63E-08	2.22E-07
Ggh	28	3.931431117	2.06319978	-1.868231336	3.67E-08	2.24E-07
Scimp	4	50.74133359	36.93181818	-13.80951541	3.72E-08	2.27E-07
30008D07R	4	33.38327254	23.24568238	-10.13759016	3.77E-08	2.30E-07
Rhoc	32	4.661050424	2.46678143	-2.194268994	3.78E-08	2.31E-07
Trim62	65	4.283107869	3.074346559	-1.20876131	3.78E-08	2.31E-07
Wdfy3	129	1.829536944	1.299033121	-0.530503823	3.78E-08	2.31E-07
Uba1	12	20.15601282	13.40432232	-6.751690506	3.81E-08	2.32E-07
Rcsd1	19	6.104455222	10.54081063	4.436355411	3.88E-08	2.37E-07
Acsl4	25	19.35311428	14.88117112	-4.471943158	3.90E-08	2.38E-07

Ccdc152	9	75.6066219	68.68217258	-6.924449318	4.17E-08	2.54E-07
[illegible]230312C02R	18	17.11568155	12.27188253	-4.843799017	4.24E-08	2.58E-07
Ctsa	41	6.471680268	4.696584052	-1.775096216	4.25E-08	2.59E-07
Rbm46os	6	32.72357724	23.1292517	-9.594325535	4.26E-08	2.59E-07
Casc4	56	4.702209462	2.799647478	-1.902561984	4.27E-08	2.60E-07
Wisp2	2	21.73913043	5.917538987	-15.82159145	4.28E-08	2.60E-07
[illegible]030039L03R	4	26.26842624	15.03838612	-11.23004012	4.31E-08	2.62E-07
Otud5	111	15.79469772	13.75348456	-2.041213162	4.38E-08	2.66E-07
Rhox13	15	97.64543115	94.57354376	-3.071887393	4.38E-08	2.66E-07
Med12	28	22.90553226	18.26373515	-4.641797108	4.53E-08	2.75E-07
Clcn4-2	66	3.733392572	2.239351605	-1.494040967	4.67E-08	2.84E-07
Mcts1	6	22.50505608	12.6361544	-9.868901681	4.70E-08	2.85E-07
Cyp4f39	22	34.72004554	32.84190011	-1.878145429	4.77E-08	2.89E-07
Edn3	31	39.42349342	43.72726027	4.303766845	4.80E-08	2.91E-07
Ethe1	6	55.01096491	68.56140688	13.55044197	4.86E-08	2.94E-07
Slc1a2	78	8.943854613	11.99542846	3.051573849	4.91E-08	2.97E-07
Gramd1a	51	3.098255348	2.273810743	-0.824444606	4.94E-08	2.99E-07
Gngt2	6	48.32573629	35.65961112	-12.66612517	5.09E-08	3.08E-07
Dbx2	59	38.32133979	35.83376792	-2.487571875	5.12E-08	3.10E-07
Gcsam	3	23.09202609	8.251310739	-14.84071535	5.16E-08	3.12E-07
Phox2b	4	38.61250818	28.18474405	-10.42776412	5.22E-08	3.15E-07
Rtn4r	88	3.004156875	2.117749002	-0.886407873	5.39E-08	3.26E-07
Cdsn	6	55.05357335	48.01582823	-7.037745117	5.39E-08	3.26E-07
Trps1	40	3.475275501	1.904125756	-1.571149745	5.53E-08	3.34E-07
Fndc4	56	3.286714969	2.452804655	-0.833910314	5.63E-08	3.40E-07
Adamtsl4	46	4.538842386	3.118945131	-1.419897255	5.84E-08	3.52E-07
Mansc4	13	49.91549996	56.52753792	6.612037966	5.92E-08	3.57E-07
Zdhhc14	112	3.999076841	3.312548088	-0.686528753	5.93E-08	3.58E-07
Srgap3	15	3.321895014	1.766842777	-1.555052237	6.12E-08	3.69E-07
Ednra	31	22.18398483	19.40564905	-2.778335783	6.12E-08	3.69E-07
Rfng	39	2.176288298	1.179404882	-0.996883416	6.32E-08	3.81E-07
Gsg1l	44	9.024249732	12.63270279	3.608453059	6.39E-08	3.85E-07
Pou3f2	8	21.9235337	15.43592856	-6.487605141	6.43E-08	3.87E-07
Ccdc149	36	2.239513686	1.732876693	-0.506636993	6.44E-08	3.87E-07

Fam213b	17	32.42292613	28.10720987	-4.315716259	6.46E-08	3.88E-07
Gm9839	2	84.92840444	69.57633875	-15.35206569	6.69E-08	4.02E-07
Pabpc2	7	98.38761742	93.87218232	-4.515435106	6.70E-08	4.03E-07
Erich4	2	69.3346574	50.51352819	-18.82112921	6.79E-08	4.08E-07
330052D11F	3	8.721167856	4.032301048	-4.688866809	6.79E-08	4.08E-07
Thbs1	2	16.54794709	27.46249705	10.91454995	6.81E-08	4.08E-07
Psd2	16	13.36236648	8.121783323	-5.240583156	6.85E-08	4.10E-07
Nrxn2	12	4.332022487	7.570486642	3.238464155	6.89E-08	4.13E-07
Meis3	30	2.787938121	1.326106564	-1.461831557	6.98E-08	4.18E-07
Adamts1	21	4.981102826	3.521832883	-1.459269943	7.01E-08	4.20E-07
Smad9	31	11.34835893	7.942001195	-3.40635774	7.21E-08	4.31E-07
Mctp2	4	42.92546831	26.80563253	-16.11983579	7.21E-08	4.32E-07
Tbx3os2	4	4.93308592	0.927761072	-4.005324848	7.23E-08	4.32E-07
Kcnt2	7	38.07998202	28.56500438	-9.514977642	7.24E-08	4.33E-07
Cpn1	16	11.54866317	9.092451638	-2.456211535	7.29E-08	4.35E-07
Gm5878	16	52.06003681	56.9409063	4.880869486	7.32E-08	4.37E-07
Mir7215	1	60	29.88505747	-30.11494253	7.32E-08	4.37E-07
Rpgr	27	16.31179173	11.70774644	-4.604045288	7.35E-08	4.39E-07
Samd11	1	40.52287582	15.59633028	-24.92654554	7.55E-08	4.50E-07
G6pc3	24	5.75672931	4.778292504	-0.978436807	7.69E-08	4.58E-07
Abcc6	5	29.14285714	15.97693205	-13.1659251	7.72E-08	4.60E-07
Akap7	11	87.17419817	80.75731734	-6.416880828	7.77E-08	4.63E-07
510028E06F	3	56.77575758	38.56787822	-18.20787935	7.99E-08	4.76E-07
Zcchc16	2	50.39819447	30.574795	-19.82339947	8.01E-08	4.77E-07
Krt35	15	89.10376423	84.14345302	-4.960311207	8.19E-08	4.88E-07
530088E08F	4	43.3670889	29.06198423	-14.30510467	8.25E-08	4.91E-07
Gm6994	2	83.33333333	54.46428571	-28.86904762	8.34E-08	4.96E-07
Smpd5	13	25.20552434	19.59331151	-5.61221283	8.45E-08	5.02E-07
Zfp334	18	6.793333671	4.551817762	-2.241515909	8.66E-08	5.15E-07
Clmp	29	22.31741723	28.20839553	5.890978305	8.79E-08	5.22E-07
110046J04R	4	7.452028509	2.062893226	-5.389135283	8.82E-08	5.23E-07
Arxes1	6	31.72540659	22.37720744	-9.34819915	8.86E-08	5.26E-07
Fut1	7	19.11773929	28.61142198	9.493682696	8.93E-08	5.29E-07
Med14	68	13.3202138	11.3172523	-2.0029615	8.93E-08	5.30E-07

Cck	6	7.265972504	3.677063477	-3.588909026	9.08E-08	5.38E-07
Six1	27	52.85986414	59.83071567	6.970851532	9.10E-08	5.39E-07
'00018B08F	18	79.94159795	73.76908885	-6.172509097	9.17E-08	5.43E-07
Stk32a	19	3.664948088	2.265363703	-1.399584386	9.37E-08	5.55E-07
Zfp667	31	2.230089642	1.208343066	-1.021746576	9.43E-08	5.58E-07
Pcsk1n	15	45.18889918	37.84596025	-7.342938928	9.51E-08	5.63E-07
Tmem91	23	21.66950023	25.8680474	4.198547174	9.58E-08	5.66E-07
Strc	2	89.88095238	73.59331476	-16.28763762	9.65E-08	5.71E-07
Lrrc4	33	71.25262517	74.72973131	3.477106143	9.67E-08	5.71E-07
Slc9a3	26	7.422999297	5.082439357	-2.340559941	9.69E-08	5.72E-07
Sh2d4a	7	19.14878819	11.50170534	-7.647082853	9.75E-08	5.75E-07
Hddc2	27	2.118843932	1.2096057	-0.909238232	9.92E-08	5.85E-07
AI463170	2	49.15957293	31.38540834	-17.7741646	9.98E-08	5.88E-07
Cdca7l	59	1.794676602	1.186027419	-0.608649184	1.00E-07	5.92E-07
Soga1	116	5.479156433	4.78862734	-0.690529093	1.03E-07	6.05E-07
AA467197	7	60.01195495	69.82539785	9.813442901	1.04E-07	6.11E-07
Entpd3	15	24.62288904	19.88984402	-4.733045019	1.08E-07	6.36E-07
Smim5	6	62.17525934	52.74093887	-9.43432047	1.10E-07	6.46E-07
Mapk8ip2	25	41.73548289	47.71701448	5.981531583	1.10E-07	6.47E-07
Ip6k1	51	3.496531989	2.117897222	-1.378634767	1.15E-07	6.74E-07
Col13a1	20	11.00780318	7.413331582	-3.594471599	1.15E-07	6.74E-07
Hoxd13	65	54.50139806	58.28222746	3.780829407	1.17E-07	6.86E-07
Syt13	12	7.777924005	3.368134506	-4.4097895	1.17E-07	6.89E-07
Cdk3-ps	1	49.27536232	18.10344828	-31.17191404	1.20E-07	7.05E-07
Prss35	9	46.39204991	55.41161774	9.019567832	1.21E-07	7.08E-07
Slc6a11	11	41.33957229	47.64100295	6.301430659	1.21E-07	7.13E-07
Mir5626	3	92.33038348	79.00277817	-13.32760531	1.24E-07	7.28E-07
Ell3	22	12.35445152	9.481649303	-2.872802215	1.24E-07	7.28E-07
Syt5	18	28.88800668	26.25867827	-2.629328418	1.26E-07	7.36E-07
Rps6kl1	28	31.51599135	27.80033319	-3.715658156	1.28E-07	7.49E-07
Zfp488	3	16.09047228	4.67143569	-11.41903659	1.29E-07	7.55E-07
Gal3st1	44	6.959820684	4.873439795	-2.086380889	1.30E-07	7.62E-07
Mir34b	17	5.49993787	2.437174237	-3.062763632	1.35E-07	7.87E-07
00064M15I	30	4.986221915	3.550394421	-1.435827494	1.35E-07	7.91E-07

Shisa9	29	18.36063689	15.03185906	-3.328777828	1.37E-07	8.03E-07
'00048M11I	6	94.82782114	88.72237027	-6.105450869	1.38E-07	8.08E-07
Cela3b	2	57.8679078	26.18984664	-31.67806116	1.38E-07	8.08E-07
Meikin	1	94.11764706	67.01030928	-27.10733778	1.38E-07	8.09E-07
Rtn2	49	4.867754476	4.101733654	-0.766020822	1.40E-07	8.17E-07
Myh11	17	19.53029973	23.82632341	4.296023678	1.42E-07	8.26E-07
Krt81	7	91.5193254	81.8977611	-9.6215643	1.43E-07	8.36E-07
Shisa8	88	8.363722313	11.09273965	2.729017341	1.49E-07	8.70E-07
Dnmt3l	4	78.97828014	63.33151946	-15.64676069	1.53E-07	8.91E-07
Mid1ip1	18	18.68439786	13.74668257	-4.937715296	1.54E-07	8.95E-07
Irak3	37	5.321621774	8.947461818	3.625840044	1.57E-07	9.12E-07
Dleu7	42	30.52147093	27.66598185	-2.855489081	1.59E-07	9.26E-07
Ppp2r5a	115	7.528672466	8.476851024	0.948178558	1.59E-07	9.26E-07
Mir2139	4	58.82247225	41.19740309	-17.62506916	1.61E-07	9.34E-07
Prkcdbp	15	4.046530082	2.041515222	-2.005014859	1.67E-07	9.72E-07
Nr2e1	28	29.24288668	34.30517196	5.062285281	1.77E-07	1.03E-06
Atp2a3	52	8.760727444	6.838954629	-1.921772815	1.81E-07	1.05E-06
Syt9	80	11.75609571	11.33941401	-0.416681691	1.83E-07	1.06E-06
Cask	69	20.19590712	17.11865332	-3.077253803	1.88E-07	1.09E-06
Disp1	38	1.523145238	0.937278753	-0.585866485	1.89E-07	1.10E-06
Pax2	77	14.99061024	14.67872783	-0.311882419	1.90E-07	1.10E-06
Prex1	84	4.392383083	3.649409198	-0.742973884	1.93E-07	1.12E-06
Nkx2-1	80	12.41631234	15.9945135	3.578201159	1.96E-07	1.13E-06
AW011738	51	1.981306028	1.434468032	-0.546837996	1.96E-07	1.14E-06
Sms	84	20.51032734	18.4019988	-2.108328542	1.97E-07	1.14E-06
Ltbp2	41	14.15715066	17.25443786	3.097287207	2.01E-07	1.16E-06
Sult2a4	5	93.19432563	85.13520745	-8.059118181	2.02E-07	1.17E-06
Ltbr	32	3.233730993	1.787623029	-1.446107964	2.04E-07	1.18E-06
)30544G11F	3	95.17582525	72.91666667	-22.25915859	2.04E-07	1.18E-06
Hoxa2	21	42.97668902	46.92710146	3.950412441	2.06E-07	1.19E-06
Pnliprp1	2	37.37722192	24.3599493	-13.01727261	2.09E-07	1.21E-06
Mir142b	13	63.44096111	56.77579098	-6.665170139	2.17E-07	1.26E-06
Atp6ap1	4	28.92063984	14.63637001	-14.28426983	2.18E-07	1.26E-06
Klhl29	83	37.99348851	36.09900302	-1.894485489	2.24E-07	1.29E-06

Cmc4	17	23.25189534	18.4322849	-4.819610441	2.37E-07	1.37E-06
Mtcp1	17	23.25189534	18.4322849	-4.819610441	2.37E-07	1.37E-06
Prr29	6	56.12690906	46.33928836	-9.787620707	2.38E-07	1.37E-06
Nes	33	3.174764234	2.214375915	-0.960388318	2.38E-07	1.37E-06
Bcap31	5	30.76696165	17.35180412	-13.41515753	2.39E-07	1.38E-06
Krt7	23	28.24568245	22.59122454	-5.654457913	2.42E-07	1.39E-06
Fam199x	44	19.11617068	15.30428603	-3.811884651	2.46E-07	1.42E-06
[illegible]30011L22R	48	2.351418847	1.564275514	-0.787143333	2.46E-07	1.42E-06
Bace1	29	3.119829151	1.763102165	-1.356726986	2.47E-07	1.42E-06
Pla2g2c	2	19.41706562	8.362831263	-11.05423435	2.51E-07	1.44E-06
Dusp15	50	5.52146611	7.490560955	1.969094844	2.53E-07	1.45E-06
Gm38426	18	76.14921067	80.079477	3.930266331	2.55E-07	1.46E-06
Fam149a	2	18.42100437	8.269350651	-10.15165371	2.57E-07	1.48E-06
Npffr1	26	14.17207582	18.78232552	4.610249697	2.57E-07	1.48E-06
Krtap15	2	91.66666667	67.72151899	-23.94514768	2.59E-07	1.49E-06
Itih1	3	74.35527896	59.12880202	-15.22647694	2.60E-07	1.49E-06
[illegible]10468N07R	22	4.668402507	8.901646793	4.233244286	2.60E-07	1.49E-06
Tmem132a	25	2.716612072	1.520683544	-1.195928528	2.68E-07	1.54E-06
Zp3	3	77.0899416	47.9020371	-29.1879045	2.69E-07	1.54E-06
Utf1	36	43.99761902	47.70674155	3.709122534	2.71E-07	1.55E-06
Gabre	6	26.19892569	17.25622076	-8.942704933	2.71E-07	1.55E-06
Ube2ql1	81	31.47327258	34.34387715	2.870604571	2.72E-07	1.56E-06
B4galnt1	139	2.366177445	1.627670694	-0.738506751	2.78E-07	1.59E-06
Bbs7	24	1.554811685	2.88455376	1.329742075	2.78E-07	1.59E-06
Htr6	82	5.474862791	4.795118908	-0.679743883	2.84E-07	1.62E-06
Fgf10	34	26.48254847	30.80878006	4.326231592	2.86E-07	1.64E-06
AU022754	89	1.679639016	1.320567456	-0.359071559	2.88E-07	1.64E-06
[illegible]10035E14R	5	72.84218081	61.14758749	-11.69459332	2.88E-07	1.64E-06
Rtkn	24	13.39054462	9.633220389	-3.757324233	2.91E-07	1.66E-06
Ssh2	88	2.028232122	1.220580423	-0.807651699	3.04E-07	1.73E-06
Plcb2	3	36.06771835	19.63755199	-16.43016636	3.05E-07	1.74E-06
Mir6991	81	92.64632932	93.77575981	1.129430491	3.05E-07	1.74E-06
Ripk3	16	20.58446121	16.13533491	-4.449126306	3.07E-07	1.75E-06
Gm12942	18	2.692615201	1.374017161	-1.31859804	3.11E-07	1.77E-06

Cdk16	48	13.15875531	10.64064951	-2.518105793	3.15E-07	1.79E-06
700049L16R	4	92.8969697	81.12302891	-11.77394079	3.17E-07	1.81E-06
Mrps23	52	26.185642	29.453188	3.267546001	3.18E-07	1.81E-06
Pdp2	3	38.20165107	54.39307187	16.1914208	3.23E-07	1.83E-06
Steap3	22	3.200482602	1.080466929	-2.120015673	3.26E-07	1.85E-06
230030E09R	22	33.55033728	27.85302095	-5.697316333	3.27E-07	1.86E-06
Pkdcc	98	3.096054739	2.46572923	-0.630325509	3.29E-07	1.87E-06
Slc16a11	10	52.44502231	64.41939089	11.97436858	3.34E-07	1.90E-06
Mir671	35	69.1511525	65.41557659	-3.735575905	3.43E-07	1.95E-06
Eml2	16	2.598596959	1.299823381	-1.298773577	3.45E-07	1.96E-06
430416O09R	5	73.18047926	79.82306678	6.642587515	3.61E-07	2.05E-06
Phyhipl	135	26.07500776	27.86362727	1.788619511	3.76E-07	2.13E-06
Tifa	12	22.07880142	17.69791441	-4.380887008	3.77E-07	2.13E-06
Vav3	109	2.267294671	1.436700016	-0.830594655	3.77E-07	2.14E-06
Thegl	43	17.1936087	19.35389926	2.160290555	3.79E-07	2.15E-06
933440J02R	4	81.04189233	69.90844625	-11.13344608	3.81E-07	2.16E-06
Gm6904	2	73.64550225	56.73963134	-16.90587092	3.83E-07	2.17E-06
Mir7676-2	4	76.6247888	63.30983117	-13.31495763	3.99E-07	2.26E-06
Mir7676-1	4	76.6247888	63.30983117	-13.31495763	3.99E-07	2.26E-06
Tmem145	37	3.737592204	2.191913223	-1.545678981	4.02E-07	2.27E-06
Map6d1	43	3.571201862	2.139761602	-1.43144026	4.07E-07	2.30E-06
Gbp7	8	7.090734277	3.857696306	-3.233037971	4.07E-07	2.30E-06
Hist3h2ba	4	4.310344828	18.99932387	14.68897904	4.20E-07	2.37E-06
Arhgef25	23	63.02552815	58.06708997	-4.958438188	4.21E-07	2.38E-06
Inpp5d	4	98.57645875	90.94488189	-7.631576863	4.38E-07	2.47E-06
Zfp423	124	13.3056245	12.92450666	-0.381117843	4.40E-07	2.48E-06
Gm10560	4	75.50578906	86.0143446	10.50855554	4.42E-07	2.49E-06
Ch25h	6	20.7140515	25.80779562	5.093744122	4.45E-07	2.51E-06
Epha8	95	7.440386586	6.103280507	-1.337106079	4.47E-07	2.52E-06
Nkpd1	9	48.05183209	39.77094752	-8.280884566	4.60E-07	2.59E-06
Tenm4	106	49.94734773	47.80488394	-2.142463791	4.60E-07	2.59E-06
Htr2a	5	20.23259128	10.72683631	-9.505754968	4.61E-07	2.59E-06
Slco4c1	14	23.20636405	19.40026629	-3.806097753	4.63E-07	2.60E-06
Cyth4	3	63.66767947	53.04166637	-10.6260131	4.69E-07	2.64E-06

Sim1	43	41.33031332	45.10133813	3.771024814	4.72E-07	2.65E-06
Pdyn	2	9.89678748	3.097919022	-6.798868459	4.74E-07	2.66E-06
Iqcf3	4	45.68636677	31.08150125	-14.60486552	4.81E-07	2.70E-06
Kansl3	72	2.998887489	1.703831762	-1.295055727	4.92E-07	2.76E-06
Dync2h1	12	39.92844942	49.56724159	9.638792173	5.11E-07	2.87E-06
930526I15R	79	1.248839044	1.81873293	0.569893886	5.19E-07	2.91E-06
Xlr3a	3	79.91334808	65.9541825	-13.95916558	5.36E-07	3.00E-06
Camk2b	74	17.80733449	21.55545962	3.74812513	5.55E-07	3.11E-06
Mcidas	66	12.46563483	11.71620809	-0.749426739	5.55E-07	3.11E-06
Foxb2	16	36.10828766	41.77385136	5.665563696	5.62E-07	3.15E-06
Ush2a	9	86.73745412	80.06049057	-6.676963555	5.66E-07	3.17E-06
310405F15R	3	10.82908901	2.906439247	-7.922649765	5.69E-07	3.19E-06
Reep2	9	48.87184623	55.7452217	6.873375473	5.72E-07	3.20E-06
Ntm	11	36.73643128	31.16587366	-5.570557617	5.75E-07	3.21E-06
Otof	8	22.89372998	13.08256062	-9.811169366	5.92E-07	3.31E-06
Rnf222	1	88.06584362	71.02803738	-17.03780624	5.93E-07	3.31E-06
Platr14	3	86.95823696	74.79873424	-12.15950272	6.03E-07	3.37E-06
Fam120c	22	16.33223948	12.52876073	-3.803478759	6.11E-07	3.41E-06
Msl3	65	16.70582057	13.40534689	-3.300473679	6.27E-07	3.50E-06
Gm10584	31	7.181178519	10.40501108	3.223832558	6.44E-07	3.59E-06
Mir1900	40	2.716016666	1.546450407	-1.169566259	6.48E-07	3.61E-06
Oxt	10	55.82969588	62.16009892	6.33040304	6.52E-07	3.63E-06
Tspan11	9	8.216478399	4.743672715	-3.472805684	6.54E-07	3.65E-06
Col26a1	23	22.07112298	17.83847941	-4.232643566	6.57E-07	3.66E-06
30159M07I	30	2.377047265	1.619544724	-0.757502541	6.58E-07	3.67E-06
Cacng5	2	40.89413876	28.07081807	-12.82332069	6.62E-07	3.68E-06
Slc36a2	2	40.8434369	26.15622815	-14.68720875	6.70E-07	3.73E-06
Ttc9b	50	10.56939498	8.777867425	-1.791527556	6.82E-07	3.79E-06
930562C15R	4	78.16955549	62.57278481	-15.59677068	6.91E-07	3.84E-06
Rhox9	3	92.4062093	80.41648105	-11.98972825	6.96E-07	3.87E-06
Osbpl7	15	8.241030262	3.915373368	-4.325656893	7.02E-07	3.90E-06
Mast1	7	12.48982672	18.18434924	5.694522516	7.04E-07	3.91E-06
Mir3104	4	60.24127675	50.14795731	-10.09331943	7.05E-07	3.91E-06
Tjp3	29	16.02394689	13.09342405	-2.930522838	7.34E-07	4.07E-06

700108J01R	5	71.35395208	61.69702543	-9.656926646	7.34E-07	4.07E-06
Mir6985	5	27.82703157	17.71491328	-10.1121183	7.36E-07	4.08E-06
Hes3	3	31.48607665	18.59266834	-12.8934083	7.43E-07	4.12E-06
Pcdhga2	6	58.27868672	69.37621152	11.0975248	7.44E-07	4.12E-06
Klhl13	15	24.09665388	33.04826066	8.951606786	7.49E-07	4.15E-06
130310I04R	8	64.51328196	55.0056528	-9.507629157	7.55E-07	4.18E-06
Rnf144a	24	36.43448637	41.07699584	4.642509472	7.62E-07	4.21E-06
Pitx2	25	39.50044979	36.28874164	-3.211708145	7.69E-07	4.25E-06
Prokr2	24	5.743444143	9.988900392	4.245456249	7.74E-07	4.28E-06
Acss3	6	28.60745292	23.24362272	-5.363830192	7.75E-07	4.29E-06
Nfasc	24	5.077679156	8.427238992	3.349559836	7.77E-07	4.29E-06
Fam159b	24	14.88073622	11.54185481	-3.338881406	7.80E-07	4.31E-06
Inpp5j	5	68.74571087	62.50430229	-6.241408578	7.82E-07	4.32E-06
Ppp1r3fos	27	29.71217054	24.62691721	-5.08525333	8.00E-07	4.42E-06
Mir5103	14	14.96250223	11.34908951	-3.613412726	8.02E-07	4.43E-06
Cpa4	4	74.37945416	65.27759969	-9.101854472	8.44E-07	4.65E-06
Crocc	47	19.52408014	16.83266635	-2.691413794	8.46E-07	4.66E-06
700123J17R	7	57.29813665	45.82232893	-11.47580771	8.46E-07	4.66E-06
Ccdc81	3	81.87120767	93.41446924	11.54326156	8.50E-07	4.68E-06
Psg17	1	57.69230769	23.88059701	-33.81171068	8.57E-07	4.72E-06
Armcx4	19	41.80468827	31.14503486	-10.65965341	8.58E-07	4.72E-06
Tenm2	7	84.10504053	75.71957105	-8.38546948	8.67E-07	4.77E-06
Arhgap6	18	24.75399598	19.15197629	-5.602019685	8.81E-07	4.84E-06
Sox2	34	37.14392423	33.70771696	-3.436207267	8.92E-07	4.91E-06
Kcne1	8	50.30656877	40.59733806	-9.709230714	8.94E-07	4.91E-06
Csf3	1	47.05882353	20.32085561	-26.73796791	8.94E-07	4.91E-06
Bsn	64	15.47165748	18.6800228	3.208365317	9.08E-07	4.99E-06
Astn1	12	60.85640727	53.20407826	-7.652329007	9.65E-07	5.30E-06
Wisp3	4	74.5686728	65.37471008	-9.193962718	9.76E-07	5.36E-06
Plxnb1	63	4.34634355	2.900541399	-1.445802151	9.79E-07	5.37E-06
Capn12	5	93.63977893	88.00218623	-5.637592696	9.80E-07	5.37E-06
Pfkp	9	5.74131385	2.024983445	-3.716330405	9.80E-07	5.37E-06
Adra1d	83	9.325221587	12.44450673	3.119285145	1.01E-06	5.55E-06
Chst5	32	22.73188213	19.89826583	-2.833616297	1.02E-06	5.56E-06

Psmb11	2	88.28125	69.12742924	-19.15382076	1.02E-06	5.56E-06
Prdm5	43	2.45488079	1.538613171	-0.91626762	1.02E-06	5.60E-06
Npc1l1	3	81.06060606	60.84839357	-20.21221249	1.05E-06	5.72E-06
Rab26	15	10.85627253	16.21194586	5.355673332	1.05E-06	5.72E-06
Ano9	1	95.08196721	64.60176991	-30.4801973	1.07E-06	5.87E-06
Adrb2	37	7.252920288	4.734567052	-2.518353236	1.08E-06	5.88E-06
Grid2ip	24	9.411663537	13.04396435	3.632300818	1.09E-06	5.95E-06
Otub2	48	2.262895283	1.215806261	-1.047089022	1.09E-06	5.95E-06
930018P22F	39	3.621638271	2.28699522	-1.334643051	1.12E-06	6.11E-06
Olfr71	2	63.53052982	47.96972523	-15.56080459	1.12E-06	6.11E-06
Cyp4f17	3	6.472249917	1.476260401	-4.995989516	1.12E-06	6.12E-06
Ccdc79	37	17.96424559	21.03120788	3.066962298	1.12E-06	6.12E-06
Shroom1	55	4.155706766	3.073989202	-1.081717564	1.13E-06	6.13E-06
Olfr225	3	97.29714912	84.58402432	-12.7131248	1.13E-06	6.16E-06
Coch	51	32.24451927	36.96161651	4.717097232	1.14E-06	6.20E-06
Ggt5	6	18.38048673	14.17664357	-4.203843162	1.14E-06	6.23E-06
Myl10	1	58.66666667	19.40298507	-39.26368159	1.15E-06	6.26E-06
Man2b2	17	1.679527123	0.314362082	-1.365165041	1.18E-06	6.43E-06
Sall1	18	48.30055514	54.65206994	6.351514804	1.19E-06	6.46E-06
Rnf26	24	1.277700981	2.204807989	0.927107008	1.20E-06	6.51E-06
Flt1	107	3.983720442	6.289555347	2.305834905	1.22E-06	6.61E-06
Usp9x	28	18.58710333	14.85335317	-3.733750158	1.22E-06	6.61E-06
700001O22F	31	3.939826384	2.479963546	-1.459862837	1.22E-06	6.61E-06
Tmprss12	11	95.06846458	90.00142154	-5.067043034	1.22E-06	6.62E-06
Fam101b	95	2.032881743	1.494323391	-0.538558352	1.23E-06	6.70E-06
Ptpre	54	1.829551964	1.311786335	-0.517765628	1.25E-06	6.79E-06
Zfp385b	72	15.24691727	18.78218349	3.535266223	1.25E-06	6.79E-06
Abl1	76	2.464383309	1.538754908	-0.925628401	1.26E-06	6.83E-06
Wdr86	27	21.90184042	19.63807563	-2.263764786	1.26E-06	6.84E-06
Kcng1	8	91.13007878	86.01719675	-5.112882026	1.26E-06	6.84E-06
Btbd9	51	2.161596684	1.255560065	-0.906036619	1.30E-06	7.02E-06
530039A03F	3	24.59490741	9.31056701	-15.2843404	1.31E-06	7.09E-06
Zfp951	5	81.94864475	68.39553429	-13.55311046	1.31E-06	7.09E-06
Mmp28	23	3.648512806	1.384759448	-2.263753358	1.33E-06	7.18E-06

Nkrf	21	21.94834285	16.84414537	-5.104197475	1.34E-06	7.22E-06
Gpr173	4	65.63108378	75.62152754	9.990443757	1.34E-06	7.25E-06
Wap	3	86.15270437	75.5398448	-10.61285957	1.38E-06	7.44E-06
Fam184a	30	32.30593243	29.14098156	-3.164950874	1.38E-06	7.45E-06
Ache	38	4.455561144	7.471409248	3.015848104	1.40E-06	7.54E-06
Cdc42bpb	30	1.66240693	0.957938535	-0.704468395	1.40E-06	7.54E-06
Mgmt	6	51.57401518	62.53225025	10.95823507	1.41E-06	7.63E-06
Ankrd34a	5	8.434550296	3.788187116	-4.64636318	1.42E-06	7.63E-06
Ush1c	1	80.68181818	95.16728625	14.48546806	1.42E-06	7.64E-06
Wfikkn2	6	61.08280104	53.16867742	-7.914123619	1.42E-06	7.64E-06
Pja1	11	20.58505057	14.99872353	-5.586327047	1.42E-06	7.68E-06
Smim10l2a	1	35.21126761	65.74074074	30.52947314	1.43E-06	7.69E-06
Srsf1	14	14.85782644	7.855123757	-7.002702685	1.44E-06	7.75E-06
Gjb2	53	2.676809562	3.982730148	1.305920585	1.46E-06	7.86E-06
730522E02R	52	11.17266807	10.22388493	-0.948783146	1.46E-06	7.86E-06
Ggt1	2	96.13259669	85.4670947	-10.66550198	1.46E-06	7.86E-06
Dlx2	64	23.08999698	25.84626315	2.756266171	1.46E-06	7.86E-06
St6gal2	62	58.78264124	55.32210405	-3.46053719	1.49E-06	7.99E-06
Mmp15	83	3.343769966	3.040321273	-0.303448693	1.50E-06	8.06E-06
Trpd52l3	2	69.69193548	55.59835645	-14.09357904	1.61E-06	8.62E-06
Col5a3	20	9.225381954	6.681665396	-2.543716558	1.62E-06	8.70E-06
Tril	61	24.24826852	20.6865877	-3.561680823	1.62E-06	8.71E-06
Nccrp1	3	40.23778154	53.05688829	12.81910675	1.64E-06	8.78E-06
930592I03R	1	71.42857143	30.76923077	-40.65934066	1.65E-06	8.84E-06
Kpna7	3	82.67419962	71.89672844	-10.77747118	1.66E-06	8.90E-06
Tmem108	69	32.30236465	34.91527199	2.61290734	1.67E-06	8.94E-06
Insm2	14	38.93422384	45.74669332	6.812469478	1.67E-06	8.95E-06
Muc20	12	64.43966829	58.7415033	-5.698164989	1.67E-06	8.95E-06
Cacna2d2	64	15.7478535	13.7413707	-2.006482799	1.68E-06	8.97E-06
Saa1	1	52.38095238	28.07881773	-24.30213465	1.68E-06	8.99E-06
Endou	3	58.83023251	47.46323589	-11.36699662	1.69E-06	9.02E-06
Entpd2	24	4.537656884	2.953804144	-1.583852741	1.71E-06	9.13E-06
Grip2	7	6.902393416	4.443845378	-2.458548038	1.72E-06	9.21E-06
Ltbp4	62	5.989253581	5.460307159	-0.528946422	1.75E-06	9.35E-06

Nradd	21	44.3307064	47.95370715	3.623000752	1.76E-06	9.39E-06
Cys1	3	9.749724972	15.89532749	6.145602519	1.77E-06	9.44E-06
Gypc	18	12.1941958	15.28425331	3.090057511	1.83E-06	9.79E-06
Tmsb4x	3	19.85815603	8.980520733	-10.8776353	1.84E-06	9.80E-06
Hes7	10	65.56489115	70.95015243	5.385261278	1.85E-06	9.87E-06
Gpx1	2	39.52466598	22.44080146	-17.08386452	1.89E-06	1.01E-05
Slc25a48	22	7.651133281	4.333116568	-3.318016713	1.92E-06	1.02E-05
Epha4	34	2.930205028	1.994331238	-0.93587379	1.93E-06	1.03E-05
'00125H03F	2	59.50721775	69.40729936	9.900081609	1.94E-06	1.03E-05
Adamts19	71	45.21538409	47.39771429	2.182330196	1.96E-06	1.04E-05
'30017C20F	3	85.84070796	69.33298365	-16.50772431	1.99E-06	1.06E-05
Rbm41	13	18.74523014	13.45581608	-5.289414066	2.02E-06	1.07E-05
Slc22a4	29	2.175225798	1.322391239	-0.852834559	2.02E-06	1.07E-05
Hoxaas3	10	60.15263501	52.52563935	-7.626995668	2.02E-06	1.07E-05
430426J06R	4	95.70619098	91.01344328	-4.6927477	2.05E-06	1.09E-05
Cdo1	39	51.62595648	54.92668758	3.300731104	2.10E-06	1.11E-05
Tomm40l	5	9.553891829	3.45710223	-6.096789599	2.10E-06	1.12E-05
Ripk4	56	2.764553287	1.861784996	-0.902768291	2.11E-06	1.12E-05
Gja4	20	3.987129676	2.542454626	-1.444675051	2.13E-06	1.13E-05
Lgr5	26	3.363983658	2.240466011	-1.123517647	2.16E-06	1.14E-05
Zar1	17	51.91853893	55.85158625	3.933047325	2.16E-06	1.14E-05
Adamts3	35	23.76961905	27.47363904	3.704019991	2.19E-06	1.16E-05
Kdf1	64	12.11638534	11.29685544	-0.819529901	2.26E-06	1.20E-05
Tsc22d3	48	23.22204808	20.47806218	-2.743985901	2.28E-06	1.21E-05
300005B03F	4	87.272479	82.03171315	-5.240765854	2.28E-06	1.21E-05
Gm14124	1	59.34065934	35.32338308	-24.01727626	2.29E-06	1.21E-05
Zbtb7c	108	4.603857484	4.043675267	-0.560182217	2.35E-06	1.24E-05
Smarca1	7	24.40495481	17.2972359	-7.107718919	2.36E-06	1.25E-05
930111H07F	2	79.62962963	50.16354445	-29.46608518	2.39E-06	1.26E-05
Nhsl2	46	20.44684784	25.12957169	4.682723848	2.42E-06	1.28E-05
Agtr1a	13	11.14717203	14.59224759	3.445075561	2.42E-06	1.28E-05
Stbd1	18	4.361472835	2.642693531	-1.718779304	2.43E-06	1.28E-05
Olfr506	1	85.71428571	55.64516129	-30.06912442	2.43E-06	1.28E-05
Wfdc15a	4	84.85971325	72.05736008	-12.80235317	2.47E-06	1.30E-05

Ldoc1l	24	48.83547324	43.34226022	-5.493213015	2.49E-06	1.31E-05
Tnni2	7	87.03825541	78.22610782	-8.812147591	2.49E-06	1.31E-05
530090E17F	20	3.207212891	1.96411107	-1.243101821	2.59E-06	1.36E-05
Spp2	7	76.86450616	69.92887686	-6.9356293	2.62E-06	1.38E-05
Csf2ra	13	50.51398986	47.44620184	-3.067788022	2.63E-06	1.38E-05
Pck1	3	73.42834298	59.68526039	-13.74308259	2.64E-06	1.39E-05
Kcna6	9	46.02207626	39.63199059	-6.39008567	2.66E-06	1.40E-05
Igsf21	29	38.85063224	35.99071109	-2.859921148	2.73E-06	1.43E-05
Emx1	60	12.63873577	15.62565254	2.986916767	2.74E-06	1.44E-05
Fmr1	61	20.60661608	18.03991106	-2.566705022	2.76E-06	1.45E-05
Msc	29	37.48108859	42.1598536	4.678765005	2.81E-06	1.48E-05
Arpin	26	4.04318938	2.849178624	-1.194010755	2.83E-06	1.48E-05
Rpl39	12	25.97154141	18.64427949	-7.327261923	2.84E-06	1.49E-05
Sh3d21	25	2.363565233	1.109687349	-1.253877884	2.86E-06	1.50E-05
Tro	5	35.11711103	44.34384353	9.226732497	2.89E-06	1.52E-05
Krt80	3	22.63021896	12.68060958	-9.94960938	2.97E-06	1.56E-05
)30406D18F	3	76.12599729	64.38605466	-11.73994263	2.98E-06	1.57E-05
Rln3	15	62.5843144	56.25989624	-6.324418155	3.01E-06	1.58E-05
Cldn11	26	28.83497301	24.82801368	-4.006959328	3.02E-06	1.58E-05
N4bp2l1	53	4.089628515	3.247499118	-0.842129397	3.07E-06	1.61E-05
430423G03F	8	79.02075994	70.73026269	-8.290497257	3.08E-06	1.61E-05
Rnf217	62	1.943920144	1.366545791	-0.577374353	3.09E-06	1.62E-05
)30515B02F	1	59.15492958	24.27184466	-34.88308492	3.12E-06	1.63E-05
Adgrv1	13	14.39332288	9.915572409	-4.477750473	3.16E-06	1.65E-05
Matn2	32	1.837851371	1.018095858	-0.819755513	3.20E-06	1.67E-05
Akr1b10	4	7.671042219	2.851983957	-4.819058263	3.31E-06	1.73E-05
Cacng6	14	45.40373731	49.80076481	4.397027504	3.31E-06	1.73E-05
Nkx2-4	113	36.14392543	38.50074315	2.35681772	3.33E-06	1.74E-05
Gm16070	3	35.8504921	52.91858679	17.06809469	3.40E-06	1.78E-05
Fzd8	53	2.367551879	1.38128611	-0.986265769	3.41E-06	1.78E-05
Nynrin	12	9.91526966	7.40638073	-2.50888893	3.42E-06	1.78E-05
Sgk2	6	45.01246444	30.36239516	-14.65006928	3.48E-06	1.82E-05
Ptpn5	14	4.109690011	6.714000602	2.60431059	3.52E-06	1.83E-05
Hykk	6	5.384140138	2.87680937	-2.507330767	3.73E-06	1.94E-05

Fam78b	135	2.493018668	1.762498387	-0.730520282	3.87E-06	2.02E-05
Mmp16	26	51.10935276	54.03192574	2.922572988	3.87E-06	2.02E-05
Lsr	70	5.384058329	4.191722617	-1.192335712	3.87E-06	2.02E-05
Olfm4	1	60.18518519	31.29251701	-28.89266818	3.89E-06	2.03E-05
Gm19402	4	58.77007521	45.03415803	-13.73591718	3.89E-06	2.03E-05
Trpc5	22	56.07730713	60.12966669	4.052359566	3.90E-06	2.03E-05
Ptgs1	15	4.155346291	7.519574599	3.364228308	3.92E-06	2.04E-05
Lrrc38	49	54.70337975	57.10164811	2.398268352	3.93E-06	2.04E-05
Sbpl	1	84.67741935	67.62589928	-17.05152007	4.02E-06	2.09E-05
Panx2	52	9.096308921	8.066332551	-1.02997637	4.13E-06	2.14E-05
Mpv17l	33	7.718509905	6.043903659	-1.674606246	4.19E-06	2.18E-05
Srsf12	41	21.10539963	24.96947928	3.864079649	4.20E-06	2.18E-05
Shroom4	36	16.03288184	13.00594977	-3.026932075	4.23E-06	2.20E-05
Mocs3	83	2.47853739	1.729428345	-0.749109046	4.28E-06	2.22E-05
Nckap5l	23	4.189669259	6.457085661	2.267416402	4.36E-06	2.26E-05
Robo3	15	11.58083898	15.82136993	4.240530952	4.43E-06	2.29E-05
Pcdhb3	2	76.5625	61.03286385	-15.52963615	4.47E-06	2.32E-05
Gm21221	3	70.32050283	54.43091431	-15.88958852	4.55E-06	2.36E-05
Gng8	19	17.36425784	13.34465924	-4.019598595	4.57E-06	2.36E-05
Trim72	6	52.55114258	45.02920812	-7.521934465	4.57E-06	2.37E-05
Arhgef6	2	50	27.72277228	-22.27722772	4.62E-06	2.39E-05
Syne3	32	5.666273701	2.8610402	-2.805233501	4.63E-06	2.40E-05
Etnppl	2	13.85526115	7.262376238	-6.592884917	4.76E-06	2.46E-05
Gm10046	6	10.4426254	18.52737484	8.084749437	4.89E-06	2.53E-05
Foxa2	52	5.271452237	4.347014978	-0.924437259	4.89E-06	2.53E-05
Lamc2	21	3.241492748	2.04804294	-1.193449808	4.90E-06	2.53E-05
930520P13R	2	51.61059981	33.39355616	-18.21704365	4.93E-06	2.54E-05
Gsx2	28	27.2192728	31.29598901	4.076716208	5.02E-06	2.59E-05
Cd81	13	4.393660816	1.454567874	-2.939092942	5.02E-06	2.59E-05
Olfml3	1	15.31531532	25.89820359	10.58288828	5.05E-06	2.60E-05
730507C01R	9	56.31627799	63.79528235	7.479004364	5.06E-06	2.61E-05
Prrt4	62	4.949480853	3.907821171	-1.041659682	5.07E-06	2.62E-05
Ifitm6	3	72.86669707	55.70473347	-17.1619636	5.09E-06	2.62E-05
Kif26a	32	9.017761911	7.366980538	-1.650781373	5.09E-06	2.62E-05

Gpr182	3	91.23258784	77.64944901	-13.58313883	5.16E-06	2.66E-05
Sorcs2	77	3.367651424	2.36127633	-1.006375093	5.33E-06	2.74E-05
Rab3c	17	24.86472165	21.75830309	-3.10641856	5.37E-06	2.76E-05
Casp7	23	2.363500156	1.334120863	-1.029379293	5.43E-06	2.79E-05
Dmtn	57	23.5800712	22.05717576	-1.522895432	5.46E-06	2.81E-05
Spata20	12	94.78913834	90.93841864	-3.850719707	5.48E-06	2.81E-05
Adck1	11	2.06828276	0.808189427	-1.260093333	5.51E-06	2.83E-05
Sgk1	112	13.65395334	11.20662723	-2.447326109	5.52E-06	2.84E-05
Sgk3	129	3.63806636	3.202495967	-0.435570394	5.59E-06	2.87E-05
Ndufa13	73	2.66069018	1.774189175	-0.886501005	5.59E-06	2.87E-05
Syt1	16	47.09460411	54.32960561	7.235001498	5.60E-06	2.88E-05
Prodh	15	6.435944429	3.444536451	-2.991407978	5.63E-06	2.89E-05
Asl	9	27.83534652	21.13649046	-6.698856056	5.69E-06	2.92E-05
Acvrl1	7	18.63354037	10.44552347	-8.188016904	5.71E-06	2.92E-05
Fgf9	38	1.830514821	4.988331976	3.157817156	5.77E-06	2.95E-05
Col6a1	2	42.84219704	23.78482513	-19.0573719	5.81E-06	2.98E-05
Dnmbp	92	1.952218844	1.257643171	-0.694575673	5.85E-06	3.00E-05
[illegible]30570G19[illegible]	22	63.70451646	66.18816281	2.483646352	5.88E-06	3.01E-05
Sox18	18	22.10099672	30.64717975	8.546183032	5.88E-06	3.01E-05
Ovol2	90	3.321717205	2.873912902	-0.447804302	5.89E-06	3.01E-05
Rtp2	6	84.42270205	76.78092046	-7.641781596	5.90E-06	3.02E-05
Trim2	47	9.673916883	7.508014414	-2.165902469	5.92E-06	3.03E-05
Calcoco2	4	39.81706061	26.24252536	-13.57453525	6.00E-06	3.06E-05
Gphb5	3	75.80595985	61.8083149	-13.99764495	6.02E-06	3.07E-05
Adgrb2	95	8.5861312	7.466615888	-1.119515311	6.15E-06	3.14E-05
Slc44a2	12	27.0259649	22.34633618	-4.679628723	6.18E-06	3.16E-05
Spin4	19	20.47870333	16.09220204	-4.38650129	6.19E-06	3.16E-05
Kcnh4	45	24.88731282	28.58665322	3.699340401	6.23E-06	3.18E-05
Tbx2	31	4.582252592	2.716628011	-1.865624581	6.26E-06	3.19E-05
Gm29766	28	94.03398423	91.56573154	-2.468252685	6.33E-06	3.23E-05
Orai3	18	1.423749282	2.381644282	0.957894999	6.34E-06	3.23E-05
Spag6l	15	43.23391941	47.58169524	4.347775825	6.44E-06	3.28E-05
Il22ra1	6	41.9462173	29.3687752	-12.5774421	6.50E-06	3.31E-05
Sybu	38	16.77433249	16.1977257	-0.576606786	6.51E-06	3.31E-05

Snca	9	11.01299772	6.937231146	-4.07576657	6.51E-06	3.31E-05
Cryba2	28	15.34364742	14.129249	-1.214398427	6.57E-06	3.34E-05
Tspan32	1	31.91489362	7.476635514	-24.4382581	6.63E-06	3.37E-05
Dsel	42	19.94105352	24.27458921	4.333535685	6.66E-06	3.39E-05
Snap25	15	23.7980443	29.0622774	5.2642331	6.68E-06	3.39E-05
Gm11992	16	3.110324879	1.824493143	-1.285831736	6.68E-06	3.39E-05
Piga	12	24.47646786	15.98223063	-8.494237225	6.75E-06	3.43E-05
Esr2	20	3.456450768	1.560324967	-1.896125801	6.76E-06	3.43E-05
Cux1	141	2.068586577	1.263240098	-0.805346479	6.82E-06	3.46E-05
Scarletltr	3	39.36946452	28.39775503	-10.97170949	6.91E-06	3.51E-05
Pknox2	50	4.993245017	3.78254253	-1.210702487	6.96E-06	3.53E-05
Hnf4g	4	11.08647771	5.693983301	-5.392494413	6.99E-06	3.54E-05
Dll3	28	19.46440424	22.54502966	3.080625422	7.01E-06	3.55E-05
Ccdc30	35	21.16906364	19.42485267	-1.744210964	7.04E-06	3.57E-05
Pcdhb2	7	31.11270895	20.99622552	-10.11648343	7.04E-06	3.57E-05
Dagla	45	2.004457175	1.421223621	-0.583233553	7.07E-06	3.58E-05
Ndufa4l2	1	16.10486891	31.68604651	15.5811776	7.09E-06	3.59E-05
Tgfb3	7	5.979656535	3.054470117	-2.925186418	7.20E-06	3.64E-05
Cd2	1	82.90598291	56.14035088	-26.76563203	7.52E-06	3.80E-05
Cfap43	28	18.2019033	20.33021333	2.128310031	7.53E-06	3.81E-05
Sacs	14	16.74478715	14.68181406	-2.062973094	7.56E-06	3.82E-05
Mmgt1	50	17.79326866	15.30238722	-2.490881434	7.66E-06	3.87E-05
Epn3	12	22.83372367	18.75978124	-4.073942421	7.70E-06	3.89E-05
Sema5a	23	48.35244226	51.8710624	3.518620131	7.74E-06	3.91E-05
Tmeff2	37	4.768984043	7.337870664	2.56888662	7.77E-06	3.92E-05
Tnfsf13b	4	39.68672375	48.53613662	8.84941287	7.80E-06	3.93E-05
Il11ra1	13	2.76094301	1.70802953	-1.052913479	7.80E-06	3.93E-05
Slc3a1	6	94.47168401	88.1985532	-6.273130808	7.81E-06	3.93E-05
Prkag2	59	7.017444948	5.214029775	-1.803415173	8.05E-06	4.05E-05
Myog	2	84.36629149	67.65633238	-16.70995911	8.09E-06	4.07E-05
Sycn	9	89.92646205	84.05435241	-5.872109644	8.29E-06	4.17E-05
Cldn19	11	96.84921378	92.73030785	-4.118905936	8.41E-06	4.23E-05
Sgol2a	46	5.48417321	4.276006127	-1.208167084	8.46E-06	4.26E-05
Dctn1	17	14.70577854	11.72184177	-2.983936769	8.63E-06	4.34E-05

Cyr61	36	2.128468751	1.241644323	-0.886824428	8.87E-06	4.46E-05
Spock3	28	29.38273375	27.89782931	-1.484904441	8.89E-06	4.47E-05
Aebp2	102	5.989919195	5.131409845	-0.85850935	8.90E-06	4.47E-05
Prex2	50	43.13661542	44.75088811	1.614272696	8.92E-06	4.48E-05
Mafb	74	46.17742819	44.27679347	-1.900634729	9.07E-06	4.56E-05
Eda	32	24.55591662	19.81944341	-4.736473213	9.11E-06	4.57E-05
Mospd3	29	3.220261444	1.92426622	-1.295995224	9.17E-06	4.60E-05
C1qb	3	58.14798117	43.38246158	-14.76551959	9.25E-06	4.64E-05
Proser2	49	2.31321004	1.600766335	-0.712443705	9.39E-06	4.71E-05
Tdrd1	30	84.40435901	82.08640261	-2.317956401	9.40E-06	4.71E-05
Ttc30a1	4	6.985957417	1.630252101	-5.355705316	9.41E-06	4.72E-05
Bdkrb1	2	95.77777778	88.27268565	-7.505092131	9.47E-06	4.74E-05
Chrna7	55	45.42021057	42.80286064	-2.617349925	9.48E-06	4.75E-05
Sox3	50	57.22876278	59.94772852	2.718965741	9.91E-06	4.96E-05
Gm10872	4	71.41897997	78.39976399	6.980784022	1.00E-05	5.01E-05
Gml2	5	96.57713645	92.14984182	-4.427294629	1.01E-05	5.06E-05
Gml	5	96.57713645	92.14984182	-4.427294629	1.01E-05	5.06E-05
Slc17a8	6	85.41713393	91.17286473	5.755730801	1.01E-05	5.06E-05
Scg2	9	5.943750466	3.218552592	-2.725197873	1.02E-05	5.09E-05
Ninl	39	2.289856875	1.40934121	-0.880515665	1.03E-05	5.15E-05
Rasl10b	60	9.236376042	7.806105856	-1.430270186	1.03E-05	5.16E-05
210420H20F	10	85.99781564	78.3860586	-7.611757033	1.04E-05	5.20E-05
Rusc2	54	10.48748236	12.62008095	2.132598586	1.05E-05	5.26E-05
Mir1231	8	92.21442537	88.74984492	-3.464580451	1.06E-05	5.28E-05
Espn	56	33.16160817	35.85123923	2.689631058	1.08E-05	5.40E-05
F8a	21	18.49722502	14.25839638	-4.238828644	1.10E-05	5.47E-05
Sorcs1	77	52.0984229	54.21403637	2.115613473	1.10E-05	5.50E-05
Aig1	60	1.814558703	1.285507413	-0.52905129	1.12E-05	5.57E-05
Yy2	18	89.71292242	92.82837622	3.115453803	1.12E-05	5.59E-05
Gpx3	2	45.09803922	62.28149036	17.18345114	1.14E-05	5.68E-05
Rab39b	3	27.10075348	17.65902489	-9.441728593	1.17E-05	5.81E-05
Slc10a4	35	18.10689857	16.98441083	-1.122487742	1.17E-05	5.83E-05
Pparg	85	4.379400186	3.514601043	-0.864799143	1.20E-05	5.95E-05
Zar1l	4	48.77045357	40.62590037	-8.144553206	1.21E-05	6.01E-05

Zfp772	2	6.036004996	0.9291559	-5.106849096	1.21E-05	6.01E-05
Uprt	14	18.84409608	14.83279492	-4.011301164	1.21E-05	6.03E-05
Tmem173	5	86.20982861	78.54112798	-7.668700629	1.26E-05	6.26E-05
Fxyd7	9	23.6950847	18.95062205	-4.744462646	1.26E-05	6.27E-05
Gm20751	9	93.24340294	88.76270555	-4.480697385	1.26E-05	6.28E-05
700025C18R	1	74.3315508	53.84615385	-20.48539696	1.27E-05	6.28E-05
Rab9	31	16.43536592	13.93306813	-2.502297787	1.29E-05	6.40E-05
Ranbp3l	7	48.50516734	40.00007806	-8.505089278	1.29E-05	6.40E-05
Tceanc	39	17.56164572	14.20504639	-3.356599324	1.30E-05	6.43E-05
Stx3	18	3.894916815	1.663289771	-2.231627044	1.30E-05	6.43E-05
Slc19a3	7	8.178222771	4.541721605	-3.636501166	1.30E-05	6.46E-05
Mir7236	32	16.3351207	14.80408745	-1.53103324	1.31E-05	6.47E-05
Hs3st6	58	6.342193433	5.531882654	-0.810310779	1.31E-05	6.50E-05
Col4a4	9	10.93484784	6.696189248	-4.238658591	1.32E-05	6.51E-05
310034G01R	58	1.564677511	1.092464443	-0.472213068	1.36E-05	6.71E-05
Rab40b	45	19.84124384	18.34980534	-1.491438505	1.36E-05	6.73E-05
Tfe3	9	28.54051163	18.56751596	-9.972995672	1.37E-05	6.76E-05
Sh3bp2	36	13.46332449	12.17902	-1.284304492	1.37E-05	6.76E-05
B4galt6	82	1.558345186	0.991383564	-0.566961622	1.38E-05	6.80E-05
Asprv1	9	80.93964859	71.25430488	-9.685343711	1.38E-05	6.82E-05
Arpc3	33	1.76537829	0.952902359	-0.812475931	1.39E-05	6.84E-05
Myo3b	4	79.69065111	70.92354903	-8.767102078	1.42E-05	6.98E-05
H60b	4	10.19617118	6.693501989	-3.502669189	1.42E-05	7.00E-05
Fbxw19	2	46.01722049	31.50831625	-14.50890425	1.43E-05	7.05E-05
Lhx9	42	49.57102363	53.27540902	3.704385391	1.45E-05	7.16E-05
Slc16a3	52	10.22723457	12.5321193	2.304884733	1.46E-05	7.20E-05
Slc8a3	41	21.11391983	19.91630908	-1.197610749	1.48E-05	7.30E-05
Rgs9bp	48	1.91819936	3.683303523	1.765104164	1.49E-05	7.35E-05
Phactr2	118	6.762149951	6.951172784	0.189022833	1.49E-05	7.35E-05
Ttc23l	8	70.16956948	73.59765564	3.428086168	1.50E-05	7.37E-05
Scarf2	96	5.677770357	5.58435797	-0.093412387	1.50E-05	7.39E-05
Pbx4	8	7.263659422	4.489008217	-2.774651204	1.50E-05	7.40E-05
Nipsnap1	13	2.13160002	0.833204328	-1.298395692	1.51E-05	7.41E-05
Larp6	68	7.698061839	9.722004535	2.023942695	1.51E-05	7.42E-05

Efemp1	9	51.56013781	45.78834909	-5.77178872	1.52E-05	7.45E-05
[illegible]00030M09[illegible]	4	90.15260401	81.95876354	-8.193840476	1.54E-05	7.54E-05
Olig2	6	12.01686982	18.30254978	6.285679962	1.56E-05	7.65E-05
Msrb2	3	8.73655914	3.297637795	-5.438921345	1.57E-05	7.71E-05
Npr2	99	18.85478563	18.77221361	-0.082572023	1.57E-05	7.72E-05
Diras1	15	4.919224137	3.013601352	-1.905622785	1.58E-05	7.76E-05
[illegible]930430L01[illegible]	16	3.398664272	1.421935809	-1.976728463	1.58E-05	7.76E-05
Mir34c	9	5.834418392	1.69698389	-4.137434502	1.59E-05	7.78E-05
Rab37	49	10.27009499	9.282371863	-0.987723123	1.60E-05	7.83E-05
Nkx2-3	23	37.62058815	40.85733038	3.236742232	1.62E-05	7.94E-05
Gabrr3	1	97.36842105	75.55555556	-21.8128655	1.63E-05	8.00E-05
Ly6k	2	39.34168383	55.19041142	15.8487276	1.64E-05	8.01E-05
Boc	28	30.44219718	30.04391972	-0.398277455	1.64E-05	8.03E-05
Dnajc15	37	2.413293362	1.562161852	-0.85113151	1.67E-05	8.18E-05
Scpep1os	1	85.24590164	62.73291925	-22.51298238	1.69E-05	8.26E-05
Tcp11l1	104	2.32089413	1.692231852	-0.628662278	1.69E-05	8.28E-05
Arap1	49	7.365248978	7.848370392	0.483121414	1.70E-05	8.32E-05
Gm7367	42	1.130613822	1.727973416	0.597359594	1.73E-05	8.46E-05
[illegible]930478L05[illegible]	2	87.79761905	74.69512195	-13.1024971	1.73E-05	8.46E-05
Adamts4	1	78.0141844	55.30726257	-22.70692183	1.74E-05	8.52E-05
Tcf7	96	9.114581917	8.41350112	-0.701080797	1.75E-05	8.54E-05
Myh10	16	12.92250344	9.533602461	-3.388900977	1.78E-05	8.67E-05
Uchl1	11	22.98077476	29.02922819	6.048453435	1.78E-05	8.68E-05
Gm15179	2	62.92134831	42.41557734	-20.50577097	1.78E-05	8.68E-05
Mecom	29	23.40600446	24.03299851	0.626994053	1.78E-05	8.69E-05
Slc35a2	28	13.82505062	10.51970146	-3.305349163	1.80E-05	8.79E-05
Pou2f3	24	14.13079475	20.90806527	6.777270521	1.81E-05	8.80E-05
Hlf	28	3.97126361	2.676903435	-1.294360175	1.83E-05	8.90E-05
Ccdc155	20	7.292782788	5.215502137	-2.077280651	1.83E-05	8.90E-05
Hist1h2aa	7	95.81779881	93.50532495	-2.312473858	1.83E-05	8.90E-05
Spred3	7	17.61327091	11.60176041	-6.011510497	1.86E-05	9.06E-05
Ak4	97	2.592855946	1.902824618	-0.690031328	1.86E-05	9.06E-05
Zim1	7	39.52691184	33.20012063	-6.326791211	1.88E-05	9.15E-05
Fcho1	30	5.422055673	3.64375925	-1.778296423	1.90E-05	9.22E-05

Taar4	5	97.51673958	93.08407381	-4.432665765	1.90E-05	9.23E-05
310002D06R	2	92.55102041	82.03463203	-10.51638837	1.91E-05	9.27E-05
Grhl3	15	5.822864438	8.920705244	3.097840806	1.91E-05	9.27E-05
932702P03R	8	91.61798417	87.54233656	-4.075647608	1.92E-05	9.31E-05
Ctsw	4	46.83155322	36.18216585	-10.64938737	1.92E-05	9.33E-05
Cygb	60	8.656990047	10.2453445	1.588354454	1.93E-05	9.38E-05
Crip3	1	22.58064516	5.333333333	-17.24731183	1.94E-05	9.42E-05
Cdv3	148	5.897078794	6.462939385	0.565860591	1.97E-05	9.55E-05
Spef2	4	35.0571608	26.73715508	-8.320005722	1.99E-05	9.63E-05
Zfp61	5	10.86956522	2.307692308	-8.56187291	1.99E-05	9.65E-05
Cc2d2a	5	10.82108367	3.399089805	-7.421993865	2.01E-05	9.74E-05
Alg13	23	10.5269045	7.218003931	-3.308900567	2.04E-05	9.89E-05
Lhx1	8	51.89792667	59.1414106	7.24348393	2.04E-05	9.89E-05
Sh2d4b	4	95.09569446	88.66557371	-6.430120749	2.04E-05	9.89E-05
Gatsl3	50	14.72182604	14.28125746	-0.440568574	2.05E-05	9.90E-05
Scube3	109	8.733472808	8.892629144	0.159156336	2.08E-05	0.000100669
Oxct2b	8	42.64161735	50.37489881	7.733281464	2.11E-05	0.000101922
Btbd18	22	95.97203774	94.09580126	-1.876236482	2.14E-05	0.000103166
Slc26a10	14	15.79463743	12.10768664	-3.686950789	2.14E-05	0.000103395
Lemd2	71	7.23939683	5.464590415	-1.774806415	2.14E-05	0.000103525
Tcf4	39	54.93373757	58.19902215	3.265284583	2.15E-05	0.000104018
Cdk5r1	134	1.880614834	1.242551497	-0.638063338	2.17E-05	0.000104834
Hipk1	75	1.530832218	1.215454655	-0.315377563	2.18E-05	0.000105394
Mpo	1	97.22222222	84.21052632	-13.01169591	2.19E-05	0.000105545
Acvr1c	50	12.11412751	10.06867282	-2.045454697	2.20E-05	0.000106256
Vstm2a	8	37.57954944	30.28045335	-7.299096093	2.23E-05	0.000107446
Nagpa	72	1.608180491	1.053482052	-0.554698439	2.23E-05	0.000107458
Aurkc	2	55.82191781	39.13043478	-16.69148303	2.24E-05	0.000107749
Vill	5	34.08788802	26.15630217	-7.931585845	2.24E-05	0.000107911
330162012R	59	1.766555472	1.216102483	-0.550452989	2.27E-05	0.000109037
Npb	11	9.959067123	6.891676461	-3.067390662	2.30E-05	0.000110439
Kcnh8	41	40.02374059	42.5519093	2.528168708	2.31E-05	0.000111241
Myo16	9	35.12674275	42.06191353	6.935170778	2.33E-05	0.000112206
Mt2	37	3.144657622	2.300034144	-0.844623479	2.41E-05	0.000115862

Aplp1	26	21.294363	24.55419618	3.259833172	2.43E-05	0.000116972
Cd6	7	71.23554385	64.2691118	-6.966432055	2.44E-05	0.000117121
C77080	109	4.664745828	4.039190162	-0.625555666	2.45E-05	0.000117445
Trp53cor1	6	15.15455809	11.29595337	-3.858604724	2.45E-05	0.000117565
Kcnc4	96	12.89114247	14.97158512	2.080442652	2.46E-05	0.000117859
Casq2	7	56.41138443	50.28713551	-6.124248911	2.46E-05	0.000117911
Rpl39l	15	90.08978771	82.13455538	-7.955232336	2.47E-05	0.000118245
Bcorl1	42	16.14650076	13.60618107	-2.540319682	2.47E-05	0.000118245
Wdr8	49	3.527022585	2.299722432	-1.227300152	2.48E-05	0.000118892
Kif26b	83	4.424045731	3.41814654	-1.005899191	2.52E-05	0.000120843
Mkrn3	28	57.01517932	59.89281741	2.877638091	2.53E-05	0.000120949
330017L17R	4	83.25804559	70.67516147	-12.58288412	2.54E-05	0.000121825
Hpcal4	2	10.43917264	4.023990983	-6.415181661	2.55E-05	0.000121986
330082N09F	5	95.74831423	86.78228986	-8.966024377	2.56E-05	0.0001226
330102I10R	11	92.26305852	89.226259	-3.036799514	2.57E-05	0.000122935
Selm	7	8.817015817	4.120631166	-4.696384651	2.59E-05	0.00012362
Prkcb	52	47.85144391	51.45178342	3.600339514	2.59E-05	0.000124016
)00092D14F	4	39.33394075	47.69534324	8.361402484	2.62E-05	0.000125223
Bnc1	110	41.08486093	42.29852454	1.213663604	2.63E-05	0.000125765
Foxo6	153	2.908586386	2.074880018	-0.833706367	2.64E-05	0.000125828
Nr5a1	5	90.06913785	80.78232932	-9.286808532	2.66E-05	0.000126933
Spata31d1a	4	94.12301597	79.11270539	-15.01031058	2.72E-05	0.000129993
Trf	10	15.90797781	11.76080839	-4.147169417	2.76E-05	0.000131548
Kif4	14	19.51973899	14.48176816	-5.037970836	2.82E-05	0.000134701
Btbd11	54	6.640984594	5.046617401	-1.594367193	2.84E-05	0.000135324
Cntn4	9	33.319133	40.01120264	6.692069636	2.90E-05	0.000138315
Cpm	30	4.307249014	3.930158552	-0.377090463	2.91E-05	0.000138878
Fads6	19	6.473810929	4.561930083	-1.911880847	2.93E-05	0.000139609
Mecp2	14	19.08047909	14.58147439	-4.499004701	2.95E-05	0.000140311
Pgbd1	6	7.414859528	3.113419309	-4.301440219	2.99E-05	0.00014231
Cct5	19	0.633053413	1.83191405	1.198860638	3.00E-05	0.000142553
Ttll7	19	2.149453917	1.636480468	-0.512973449	3.00E-05	0.000142625
Disc1	56	10.90326859	12.62012852	1.716859935	3.00E-05	0.000142625
Thoc2	33	18.62877333	16.18664804	-2.44212529	3.04E-05	0.000144478

i30036O11F	5	13.20599922	5.62462744	-7.581371784	3.05E-05	0.000145164
931431F19F	2	86	69.375	-16.625	3.06E-05	0.000145244
Sat1	30	19.63280497	16.47520915	-3.157595819	3.06E-05	0.000145316
Grb7	6	11.59935112	5.787547033	-5.81180409	3.06E-05	0.00014537
Mir1895	21	3.446061152	1.931764275	-1.514296876	3.07E-05	0.000145858
Lnx1	3	88.62345384	77.27554938	-11.34790446	3.10E-05	0.000146876
Tmem229a	90	57.55320451	59.48446885	1.93126434	3.11E-05	0.000147624
Myh4	3	54.35897436	37.89297068	-16.46600368	3.15E-05	0.000149125
Abcg8	6	12.84718967	7.303719427	-5.543470238	3.23E-05	0.000153108
Sox4	17	2.474611681	0.969659589	-1.504952092	3.24E-05	0.000153387
Adam33	14	16.53365235	13.55943058	-2.974221772	3.24E-05	0.000153676
Btg3	33	6.707026054	5.101832841	-1.605193213	3.25E-05	0.00015377
Ada	40	2.707527369	1.931742142	-0.775785226	3.27E-05	0.000154782
Ano7	8	56.01142939	49.10253401	-6.908895383	3.29E-05	0.000155614
Col4a3	12	10.52324695	6.246860389	-4.276386564	3.35E-05	0.000158392
Ece2	45	14.12224525	12.94428003	-1.17796522	3.39E-05	0.00016016
Myadm	16	3.812884975	1.933929694	-1.878955281	3.40E-05	0.000160513
Tspan2	53	3.570070016	3.163686914	-0.406383101	3.40E-05	0.000160529
Theg	1	51.52838428	33.66336634	-17.86501794	3.41E-05	0.000161245
Maged1	2	24.67961165	10.08403361	-14.59557804	3.41E-05	0.000161245
S1pr5	25	9.607453402	9.20222856	-0.405224842	3.44E-05	0.000162564
Syt12	47	2.007353966	1.545702516	-0.46165145	3.48E-05	0.000164054
Tmc5	13	57.01709347	50.09076045	-6.926333025	3.53E-05	0.000166709
Wdr31	11	2.744671966	1.457575986	-1.28709598	3.55E-05	0.00016753
Kcnk6	60	8.196081375	8.197079102	0.000997727	3.58E-05	0.000168794
Vcpkmt	45	24.02339026	21.45161143	-2.57177883	3.59E-05	0.000169019
Scarb1	11	12.8793463	7.552347766	-5.326998531	3.61E-05	0.000170267
Nfic	1	57.69230769	19.64285714	-38.04945055	3.62E-05	0.000170528
Slc35f3	64	9.468121838	10.96227193	1.494150088	3.62E-05	0.000170533
Fkbp1b	51	3.389830918	2.038876087	-1.350954832	3.63E-05	0.000171002
Dlx1as	16	43.35721297	46.82930454	3.472091571	3.65E-05	0.000171557
110012L19F	2	21.42857143	3.488372093	-17.94019934	3.65E-05	0.000171625
Ttc8	9	2.918775186	1.182970735	-1.73580445	3.66E-05	0.000172044
Slc25a43	24	20.4257228	16.25456058	-4.171162223	3.70E-05	0.000173967

Folh1	4	63.8736614	53.08771561	-10.78594579	3.78E-05	0.000177645
Clint1	80	1.779856882	1.287997866	-0.491859016	3.80E-05	0.000178589
Exoc3l	7	60.88712106	70.70019528	9.813074211	3.84E-05	0.000180493
Igfbp3	99	2.320115682	1.532588219	-0.787527463	3.86E-05	0.000181133
H2-T10	4	25.08679262	19.50110204	-5.585690579	3.87E-05	0.000181495
Cplx4	9	83.56979113	77.00977688	-6.560014252	3.87E-05	0.000181516
Rragd	13	11.2022714	8.456344998	-2.745926399	3.88E-05	0.000181841
Magea8	3	88.74154775	81.12012256	-7.621425199	3.93E-05	0.00018417
'00001P01F	13	91.08793815	86.85522909	-4.232709058	4.01E-05	0.000188058
Gm14169	64	5.903911328	4.690743155	-1.213168173	4.03E-05	0.000188968
Slc1a7	2	87.96464646	72.56637168	-15.39827478	4.08E-05	0.00019115
Gfpt2	23	26.78277254	23.33380229	-3.448970246	4.12E-05	0.000193031
Hmcn1	21	19.93191815	24.6018392	4.669921049	4.13E-05	0.000193461
Tspo	5	17.74227235	9.671431583	-8.070840764	4.14E-05	0.000193561
Tmem8b	28	2.6937143	1.680518791	-1.01319551	4.14E-05	0.000193685
Tmem179	13	29.13026947	36.81290739	7.682637923	4.18E-05	0.000195377
Klf3	127	6.655544715	5.935611638	-0.719933078	4.18E-05	0.000195377
Csrp2	50	4.830588706	2.921402527	-1.909186179	4.18E-05	0.000195562
Dkk1	7	26.32501644	19.42295891	-6.902057532	4.19E-05	0.000195756
Stxbp6	49	2.024956118	1.370700441	-0.654255677	4.19E-05	0.000195756
Agk	37	2.38291146	1.719319899	-0.663591561	4.22E-05	0.000197065
Kl	30	18.97711189	17.6185604	-1.358551497	4.24E-05	0.000197988
Peg12	65	60.38815276	62.45885465	2.07070189	4.34E-05	0.000202782
P2ry6	1	80	48.71794872	-31.28205128	4.35E-05	0.000202805
Mir328	5	95.35839167	88.10703048	-7.251361187	4.41E-05	0.000205882
Prkch	81	11.16435134	13.11208226	1.947730927	4.47E-05	0.000208502
Ms4a15	4	95.82301038	90.0341058	-5.788904577	4.50E-05	0.000209557
Rasal1	63	6.236444228	5.756563712	-0.479880516	4.50E-05	0.000209756
Kctd15	13	13.05278307	9.245531485	-3.807251587	4.53E-05	0.000211092
Irgm1	9	3.991811629	2.139789537	-1.852022092	4.55E-05	0.000211965
Map1b	29	2.752205576	1.467008524	-1.285197052	4.55E-05	0.000211975
Pfkfb4	23	2.940519219	1.686643556	-1.253875662	4.56E-05	0.000212417
Hoxc6	8	19.66202861	15.14875845	-4.513270165	4.58E-05	0.000213057
Cutal	1	28.97196262	16.18798956	-12.78397306	4.60E-05	0.000214082

Cdcp1	5	13.15972222	4.941176471	-8.218545752	4.61E-05	0.000214549
Mir124a-1hg	12	16.49116282	14.69501264	-1.796150179	4.72E-05	0.000219341
Vwa1	8	3.341548262	1.690240006	-1.651308256	4.72E-05	0.000219424
H2-K2	30	2.922606296	1.871988812	-1.050617484	4.74E-05	0.000220318
Creb3l3	5	33.07287094	23.45573585	-9.617135091	4.81E-05	0.000223396
Kctd14	20	6.406191656	4.880732473	-1.525459182	4.95E-05	0.000229859
Ctsh	10	3.4495286	1.408192812	-2.041335788	5.01E-05	0.000232398
Nipal4	14	20.54912269	17.825292	-2.723830693	5.07E-05	0.000235244
Slc7a3	14	60.39419025	69.68665468	9.292464431	5.08E-05	0.000235581
600014K23R	1	83.33333333	60.92715232	-22.40618102	5.16E-05	0.000239329
Rbmx	29	21.56667476	18.13608905	-3.43058571	5.23E-05	0.000242513
Emid1	39	14.5771164	13.1263414	-1.450774998	5.23E-05	0.000242536
Mir9-2	4	45.64793614	36.73610947	-8.911826668	5.28E-05	0.000244718
Foxc1	87	5.206791281	4.484513184	-0.722278098	5.30E-05	0.000245465
Tmem198	37	2.193134551	1.409845824	-0.783288727	5.30E-05	0.000245465
Nhsl1	4	5.727233384	2.166666667	-3.560566718	5.41E-05	0.000250414
Sparcl1	4	44.58747645	33.30339196	-11.28408449	5.42E-05	0.000250598
Tnf	3	93.05653296	84.77114357	-8.285389392	5.46E-05	0.000252277
Helt	17	7.280521476	13.4373932	6.156871729	5.54E-05	0.00025629
Lsmem1	1	43.63636364	11.29032258	-32.34604106	5.62E-05	0.000259872
Frmpd4	9	23.481249	31.71860166	8.237352658	5.68E-05	0.000262441
Hdhd3	21	4.11412685	2.590672757	-1.523454094	5.69E-05	0.000262719
Fbxw21	2	87.94984844	78.0187722	-9.931076246	5.73E-05	0.000264521
Creld1	59	2.446784533	1.911727776	-0.535056757	5.75E-05	0.00026519
Mir219a-2	66	91.9595009	93.44327702	1.483776122	5.81E-05	0.000267949
Tsen15	26	23.12110684	21.53929479	-1.581812052	5.84E-05	0.000269554
Zfp608	2	4.216998755	1.630746732	-2.586252022	5.93E-05	0.000273309
Tssk1	8	94.08377918	88.31148571	-5.772293462	5.93E-05	0.000273508
Mapk13	47	5.699445303	4.413277651	-1.286167652	6.02E-05	0.000277467
Esrrb	33	27.38348146	32.46199218	5.07851072	6.05E-05	0.000278594
Fgf14	11	49.97396509	44.45239995	-5.521565145	6.07E-05	0.000279534
930556M19R	27	3.077551219	1.641731985	-1.435819234	6.16E-05	0.000283646
Gpld1	2	23.83436139	13.80944939	-10.024912	6.20E-05	0.000285259
Papln	14	5.692933502	3.26344405	-2.429489453	6.20E-05	0.00028529

Ntrk2	27	43.042849	47.67913012	4.636281114	6.21E-05	0.000285745
Slco2a1	23	3.771087088	2.57132264	-1.199764448	6.23E-05	0.00028662
Fhdc1	89	3.131819533	4.074161246	0.942341713	6.25E-05	0.00028746
Mir7031	5	14.56994943	20.34286485	5.772915417	6.27E-05	0.00028834
Glrb	18	20.27234777	24.46553361	4.193185845	6.32E-05	0.000290315
Fezf2	20	22.90288697	18.64711179	-4.255775181	6.34E-05	0.000291403
Krt18	13	5.407053596	3.783893144	-1.623160453	6.35E-05	0.000291512
Dock1	36	2.144466549	1.396790864	-0.747675685	6.37E-05	0.000292525
Corin	4	15.92027505	23.38757594	7.467300897	6.39E-05	0.000293329
330444B04R	1	79.28802589	90.2173913	10.92936541	6.45E-05	0.000295832
700039E22R	42	16.23999501	15.68280335	-0.557191657	6.45E-05	0.000295832
Cd180	1	58.69565217	28.91566265	-29.77998952	6.47E-05	0.000296454
Cacnb1	58	5.424918979	4.364330278	-1.060588701	6.51E-05	0.000298512
Xlr	3	62.08229413	52.98285686	-9.099437268	6.65E-05	0.000304611
930483J18R	14	2.331553092	1.1706448	-1.160908292	6.71E-05	0.000307489
Dll1	71	2.579100923	1.706290017	-0.872810906	6.81E-05	0.000311604
Ttyh1	25	17.15901051	15.59498912	-1.564021389	6.86E-05	0.00031415
Mir7000	5	95.23317293	87.58787879	-7.645294146	6.87E-05	0.000314198
Csgalnact1	31	2.387408955	1.678500704	-0.70890825	6.90E-05	0.000315649
Dgkb	6	23.58854228	30.58202198	6.993479701	6.91E-05	0.000315799
Ccdc92	133	6.259084243	5.501128318	-0.757955925	6.98E-05	0.000318921
Ube2o	121	1.567383083	1.179806108	-0.387576975	7.03E-05	0.000321125
Zfp879	7	42.13026571	34.93333676	-7.196928941	7.11E-05	0.000325017
Mzb1	6	95.49887364	90.55357763	-4.945296012	7.12E-05	0.000325085
Cxx1b	4	28.65498385	19.52756553	-9.127418321	7.18E-05	0.000327776
Rnf125	3	23.07954428	13.23935786	-9.84018642	7.18E-05	0.000327776
BC037032	25	2.530065773	1.318754417	-1.211311356	7.20E-05	0.000328551
Cxcl3	9	14.70250899	11.8685435	-2.833965486	7.25E-05	0.000330852
Slc22a1	5	95.99565457	91.63717751	-4.35847706	7.28E-05	0.000332002
Abtb2	47	2.582021701	1.5116765	-1.070345201	7.30E-05	0.000332612
Tex13	14	90.61879761	86.45897252	-4.159825093	7.31E-05	0.000333265
Glra1	13	10.22349733	8.339238682	-1.884258649	7.33E-05	0.000334079
Itgal	5	43.16693649	54.23417037	11.06723388	7.36E-05	0.000335225
Rims4	54	17.09433949	22.078505	4.984165509	7.42E-05	0.000337764

330046C22F	1	53.125	35.95505618	-17.16994382	7.48E-05	0.000340659
Ccdc24	13	40.92476227	44.56351187	3.638749597	7.49E-05	0.000340923
Lipe	6	37.78677408	32.62366658	-5.163107495	7.58E-05	0.000344627
Zap70	14	84.32460393	78.88919116	-5.43541277	7.66E-05	0.000348497
Mirlet7c-2	5	69.04277581	74.23009566	5.187319844	7.68E-05	0.000349255
Sbspon	22	33.29357453	30.70048129	-2.593093239	7.69E-05	0.000349263
S100z	10	94.29320218	89.99070116	-4.302501013	7.71E-05	0.000350056
Vdr	35	3.06959909	2.295586792	-0.774012298	7.73E-05	0.000351011
Foxb1	29	25.01695939	27.95674991	2.939790519	7.77E-05	0.000352731
H2-K1	39	1.633516034	1.10230438	-0.531211654	7.78E-05	0.000353314
Lrp4	49	10.19785109	9.066149884	-1.131701205	7.79E-05	0.000353425
OC10263175	3	70.7286502	64.22155574	-6.507094459	7.79E-05	0.000353425
Mir6977	7	93.11641644	89.25863245	-3.857783989	7.80E-05	0.000353492
Myef2	60	1.561855917	2.208173353	0.646317436	7.80E-05	0.000353492
Crhr1	74	27.49181896	26.42927941	-1.062539546	7.80E-05	0.000353492
Mfap2	4	40.34691455	26.63816696	-13.70874759	7.87E-05	0.000356347
Mir8120	57	1.930617232	1.53778554	-0.392831692	7.95E-05	0.000360089
Epha1	33	2.893573583	1.879352357	-1.014221226	7.95E-05	0.000360126
Tifab	1	48	17	-31	8.03E-05	0.000363324
LOC432842	69	63.75230265	61.69894611	-2.053356536	8.05E-05	0.000364269
Eed	28	0.714055757	1.415522678	0.701466921	8.07E-05	0.000365079
Ptpn12	112	1.572796908	1.218638312	-0.354158596	8.19E-05	0.000370309
Lpcat2	35	34.83540765	36.63935266	1.803945012	8.25E-05	0.000373092
Ap1s3	15	18.56825249	21.07653468	2.508282189	8.27E-05	0.000373586
Ptges	13	13.43989762	9.769744563	-3.670153061	8.28E-05	0.000373979
Sel1l3	103	2.117138421	1.584808966	-0.532329454	8.44E-05	0.000381081
Mir10b	5	56.10879703	49.02425692	-7.084540109	8.51E-05	0.000384056
Pcdhac1	23	55.05454101	58.84465402	3.790113011	8.59E-05	0.000387621
410124H12F	1	18.00643087	7.482993197	-10.52343767	8.70E-05	0.00039244
Trim15	5	22.82100046	16.92228977	-5.898710695	8.76E-05	0.000395112
Tmem132b	87	13.61270596	15.37775302	1.765047059	8.79E-05	0.000396551
Hexb	39	4.317482012	2.54521096	-1.772271052	8.80E-05	0.000396845
Fut4-ps1	4	69.98180741	58.49269279	-11.48911462	8.84E-05	0.000398285
Zc3hc1	6	2.988870457	0.524934383	-2.463936074	8.90E-05	0.00040095

Dhcr7	6	2.550626166	0.783978012	-1.766648153	8.91E-05	0.00040132
Mapk10	4	52.98941799	50.77431882	-2.215099171	8.98E-05	0.000404401
Ndrg2	22	4.699451247	2.808260653	-1.891190593	9.06E-05	0.000407785
Clstn1	108	1.575143344	1.072617836	-0.502525508	9.07E-05	0.000407974
Otx1	82	2.896401386	2.019555517	-0.876845869	9.10E-05	0.000409235
Fam46c	66	2.233876248	1.555506355	-0.678369893	9.17E-05	0.00041231
Tnp2	1	55.55555556	81.9047619	26.34920635	9.24E-05	0.000415314
Rcan2	41	8.010971895	6.694327645	-1.31664425	9.32E-05	0.000418822
Sncb	26	14.35612568	12.88954129	-1.466584396	9.37E-05	0.000420817
Olfr633	1	88.63636364	65.04854369	-23.58781995	9.39E-05	0.000421737
Tsc22d1	161	4.479586	3.864975596	-0.614610403	9.40E-05	0.000422134
Mir6996	3	96.79894315	86.7910759	-10.00786726	9.41E-05	0.000422268
Nsun7	56	9.892349049	9.650412208	-0.241936842	9.58E-05	0.000429773
Larp4b	69	1.98512042	1.303237991	-0.681882429	9.59E-05	0.000430399
Yipf6	47	25.12120497	22.66437212	-2.456832848	9.60E-05	0.000430726
Ap1b1	81	6.091825005	4.95310492	-1.138720086	9.68E-05	0.000434184
Pcdha3	3	18.43296303	8.279758426	-10.1532046	9.69E-05	0.00043437
Zglp1	16	93.77364066	90.43097263	-3.34266803	9.81E-05	0.000439337
Atf6b	53	8.482393786	7.176023496	-1.30637029	9.98E-05	0.000447129
Ints5	39	14.87053912	13.03793909	-1.832600039	0.00010005	0.000448029
Mir5620	7	4.49105949	1.417159205	-3.073900285	0.00010275	0.000459969
Cx3cl1	20	10.50008501	6.726454899	-3.773630109	0.00010352	0.000463273
Sptssa	25	2.564901552	1.569076532	-0.99582502	0.00010382	0.000464474
Nme4	20	9.443254696	9.119604265	-0.323650431	0.00010393	0.000464814
Nadsyn1	9	2.199793224	0.643424955	-1.556368269	0.00010459	0.000467636
Sspo	2	76.26352762	64.22018349	-12.04334414	0.00010473	0.000468092
Rhd	11	75.4568419	68.71715498	-6.739686921	0.00010493	0.000468864
Zfp580	22	6.694492579	9.282557613	2.588065034	0.00010604	0.000473667
Pnky	47	19.9509175	18.08257972	-1.86833778	0.00010646	0.000475392
Dpf3	25	1.588532364	1.195790353	-0.392742011	0.00010872	0.000485359
Triqk	15	6.114320443	4.044947705	-2.069372738	0.00010897	0.000486335
Adamts14	55	1.987470131	1.30402636	-0.68344377	0.00010942	0.000488184
Pex3	30	1.404309151	0.630266805	-0.774042346	0.00010985	0.000489934
Trim71	16	8.930020862	7.241152481	-1.688868381	0.00011008	0.000490817

Mamld1	25	19.19804062	16.14586707	-3.052173552	0.00011228	0.000500463
Krtcap3	4	61.83431599	52.10540881	-9.728907175	0.00011327	0.000504726
Tshr	11	10.94482084	7.773944068	-3.17087677	0.00011372	0.000506577
Lrrc10	22	62.82685706	66.04678657	3.219929513	0.0001139	0.000506957
Slc6a2	69	46.62843902	44.52535108	-2.103087941	0.00011386	0.000506957
Cldn4	3	35.88240132	26.85884279	-9.023558534	0.00011387	0.000506957
Slc39a8	9	4.113953523	1.258835309	-2.855118214	0.00011446	0.000509165
Hoxb5os	1	88.33333333	72.98578199	-15.34755134	0.00011447	0.000509165
Gm5414	1	42.9787234	26.16033755	-16.81838585	0.00011517	0.000512126
Pnpla1	3	71.89811739	58.75889968	-13.13921771	0.00011537	0.00051286
Vwa7	6	28.76104941	21.92140883	-6.83964058	0.00011558	0.000513616
933421I07R	1	98.98989899	86.46616541	-12.52373358	0.00011568	0.000513933
Gaa	12	8.912898237	4.674417024	-4.238481213	0.00011636	0.000516752
Mylk2	7	36.15852916	28.6883899	-7.47013926	0.00011639	0.000516752
Susd4	74	9.441657939	9.036105149	-0.40555279	0.00011656	0.000517346
Usp11	14	18.06182836	14.1655529	-3.896275454	0.00011712	0.000519703
Lrrc23	2	17.87998958	7.443589409	-10.43640017	0.00011823	0.000524459
Phka2	13	21.54853273	15.8999494	-5.648583325	0.00011845	0.000525287
Hoxc9	34	33.49712037	30.73814352	-2.758976854	0.00011867	0.000526075
Plekhd1os	11	22.88040032	26.16985205	3.289451733	0.00012018	0.000532631
Haghl	9	9.604871203	6.575742562	-3.029128642	0.00012081	0.00053525
AI467606	3	10.05086719	5.485423181	-4.565444013	0.00012104	0.000536092
Six4	46	2.672604545	1.715347641	-0.957256904	0.00012134	0.000537297
Ccl1	2	69.80519481	57.45184184	-12.35335297	0.00012164	0.000538464
Nr0b1	7	47.58837464	36.02156075	-11.56681388	0.00012572	0.000556328
Mcmdc2	16	62.9377408	65.98878385	3.051043055	0.00012597	0.000557115
Gm9920	5	4.378481276	2.205619844	-2.172861432	0.00012596	0.000557115
Mfhas1	97	1.966071144	1.155742554	-0.81032859	0.00012833	0.000567215
Gnai1	40	3.393825455	2.765985258	-0.627840197	0.00012831	0.000567215
Plcxd3	6	15.88505684	12.63275957	-3.252297267	0.00012948	0.000572095
Arhgap36	28	47.41570957	43.7168596	-3.698849965	0.00013008	0.000574602
Camkv	20	16.09048418	14.18029442	-1.910189763	0.00013054	0.000576463
Acsf3	23	3.109459024	1.662962683	-1.446496341	0.00013093	0.000578003
Ribc2	6	32.19296209	38.61340113	6.420439041	0.00013191	0.000582152

Actrt3	15	4.880565499	3.630694427	-1.249871072	0.00013322	0.000587775
Sema3c	45	1.901547443	1.525133066	-0.376414378	0.0001336	0.000589242
700065J11R	6	95.36812743	91.30603746	-4.062089967	0.00013381	0.000590006
Polr2f	33	1.069986983	0.721447522	-0.348539461	0.0001343	0.000592007
Tssk6	60	2.816101903	2.047359498	-0.768742405	0.00013453	0.000592807
Slc38a5	1	31.95876289	58.49056604	26.53180315	0.00013545	0.000596691
Tagap	3	8.447848448	14.18257937	5.734730918	0.00013595	0.000598736
Scgn	9	31.00735177	28.41923757	-2.588114198	0.00013682	0.000602368
Ocln	27	4.900463753	6.730297418	1.829833665	0.00013692	0.000602621
Ptchd4	26	44.06520721	41.43336414	-2.631843065	0.00013707	0.000603111
Nrg3	104	63.56313922	62.2343896	-1.328749619	0.00013749	0.000604792
Col12a1	29	5.708047769	5.181490962	-0.526556806	0.00013767	0.000605379
Timm17b	18	14.37736509	11.23741741	-3.13994768	0.00013801	0.000606704
Hes6	48	2.583068979	1.881904539	-0.701164439	0.00013922	0.000611842
Fam195b	51	7.294556689	6.035015315	-1.259541374	0.0001395	0.000612893
Erp27	3	90.95988182	84.24459527	-6.715286551	0.0001406	0.000617532
Cmtm7	53	2.569268192	2.138852424	-0.430415768	0.00014113	0.00061967
B3gnt5	10	13.56956864	8.676443773	-4.893124872	0.00014252	0.000625579
Anapc5	23	1.412711762	2.283072803	0.870361042	0.00014476	0.000635219
Pthlh	69	2.059602261	1.626300542	-0.433301719	0.00014507	0.000636417
Hs3st5	27	11.55140742	10.6847578	-0.866649617	0.00014555	0.000638317
Osmr	23	1.922469397	1.252595732	-0.669873665	0.0001463	0.000641416
Tram1l1	48	64.03940527	66.42164465	2.382239381	0.00014724	0.000645331
Zcchc24	61	2.19182304	1.455672634	-0.736150407	0.00014753	0.000646418
Cpe	37	1.952537546	1.14435711	-0.808180437	0.00014929	0.000653956
Atxn7l2	27	1.655267541	0.771843953	-0.883423588	0.00014959	0.000655044
Fscn1	39	4.659622247	3.627349317	-1.03227293	0.00014976	0.000655611
Adora2a	34	3.135839637	4.958049286	1.82220965	0.00015332	0.000670989
Pkia	32	1.901341519	1.22425786	-0.677083659	0.00015347	0.000671428
Gpx8	10	6.441615343	3.057347913	-3.384267431	0.0001541	0.000674006
Fbxo27	13	42.53242729	41.95949098	-0.572936305	0.00015604	0.00068226
Fam229a	43	67.85518331	70.84187768	2.986694364	0.00015764	0.000689085
Ddn	19	5.115537447	3.293705454	-1.821831993	0.00015789	0.000689969
Tbc1d25	9	15.19180041	10.80056263	-4.391237782	0.00016021	0.000699881

Msn	28	14.93387081	12.29691697	-2.636953836	0.00016073	0.000701964
Rab33a	5	47.1812294	39.96453456	-7.216694841	0.00016226	0.000708421
Gm16432	4	97.74769305	94.07094368	-3.67674938	0.00016526	0.000721307
Nat3	2	74.41783998	65.51797631	-8.899863674	0.00016553	0.000722288
Aox4	5	26.90263584	18.28942297	-8.613212872	0.00016565	0.000722591
)30519K11F	102	24.91284484	23.95684697	-0.955997871	0.00016613	0.000724462
Dnajc11	106	3.204731531	2.610762533	-0.593968998	0.00016807	0.000732702
Gm128	21	88.53723104	90.5030549	1.965823863	0.00016824	0.000733238
Tnks1bp1	3	10.02734131	4.486859343	-5.540481965	0.00016844	0.000733865
Thnsl1	16	1.595314885	0.94194186	-0.653373025	0.00016879	0.000735185
Rgs8	12	32.92322406	29.18765009	-3.735573965	0.00017059	0.000742804
Fnbp1	106	10.12387345	9.154755436	-0.969118013	0.00017248	0.000750825
Xpot	63	5.439476647	6.53974188	1.100265233	0.00017297	0.000752716
Slc22a21	41	1.548255118	0.980562624	-0.567692494	0.00017441	0.000758778
Pfkm	5	27.5113747	19.2917619	-8.219612795	0.00017551	0.000763319
Mreg	82	1.727836728	1.197583265	-0.530253463	0.00017918	0.000779058
Ttc12	6	5.776765599	1.991546583	-3.785219016	0.00017933	0.000779466
Nat8l	59	20.0786625	19.62623283	-0.452429674	0.00017974	0.000781002
Tmem136	48	2.617692708	1.873411257	-0.74428145	0.00017982	0.000781127
Stra8	4	93.66207322	87.40985577	-6.252217452	0.00017992	0.000781357
Uhrf2	79	35.86933805	37.29203922	1.422701172	0.00018144	0.000787701
Myocd	26	43.15721916	39.8100981	-3.347121057	0.00018336	0.00079579
C4a	2	92.25352113	84.67037929	-7.583141837	0.00018475	0.000801612
Mir6364	1	89.8989899	69.91869919	-19.98029071	0.0001874	0.000812877
Sult2b1	24	3.628214338	2.385638712	-1.242575626	0.00018776	0.000814195
Mal	12	2.800159031	1.625305378	-1.174853654	0.000189	0.000819298
Slc25a5	21	24.69950962	21.32858203	-3.370927593	0.00018922	0.000820039
Mogat2	1	28.16091954	12.80788177	-15.35303777	0.00018937	0.000820441
Sall2	7	5.498591447	3.325806731	-2.172784716	0.00019125	0.00082833
Arsj	4	23.41916558	10.90043669	-12.51872889	0.00019181	0.000830514
Gm16702	3	36.84906159	25.33128013	-11.51778146	0.00019264	0.000833854
Ndst4	6	53.69192667	45.60889036	-8.08303631	0.00019367	0.000838079
.30019O22F	3	6.504881745	2.59879905	-3.906082695	0.00019582	0.000847124
Fam50a	8	22.65280722	17.41128103	-5.241526183	0.000199	0.000860612

Oacyl	3	50	68.75066454	18.75066454	0.00020079	0.000867909
930455C13F	6	95.42581273	89.7524675	-5.673345233	0.0002008	0.000867909
Ccdc132	7	1.842911967	0.874047446	-0.968864521	0.00020159	0.00087107
Magi2	36	23.04608766	26.42289183	3.376804178	0.00020204	0.000872756
Gpr37	22	42.24753441	47.08501586	4.837481452	0.00020237	0.00087392
Grm6	15	21.30789775	25.5938957	4.285997949	0.000203	0.000876374
Gss	20	2.559894766	1.329719638	-1.230175128	0.00020582	0.000888311
H2afb3	6	92.99619291	86.48877083	-6.507422086	0.00020969	0.00090473
Tmem181a	14	2.300811769	0.765766995	-1.535044775	0.00021408	0.000923148
Wdr90	34	1.550622067	0.901729878	-0.648892189	0.00021403	0.000923148
Rasgrf1	13	52.31652762	59.18708993	6.870562314	0.00021453	0.000924783
Lrrc8e	20	13.83383539	11.63960628	-2.194229117	0.00021537	0.000928134
Mir7040	7	92.60319176	85.57028788	-7.032903877	0.00021994	0.000947548
Csf2rb	5	66.39542621	58.63212568	-7.763300531	0.0002215	0.00095399
Emp2	48	2.304781816	1.750747736	-0.55403408	0.00022227	0.000957048
Mir6346	2	34.36724566	25.77589134	-8.591354316	0.00022297	0.000959763
Stom	15	3.25236125	1.801047848	-1.451313401	0.00022383	0.000963203
Kcnk13	8	9.064180706	6.487252356	-2.57692835	0.00022625	0.00097332
330018L16F	21	32.04479062	35.86022498	3.815434358	0.00022941	0.000986635
Gm28979	1	54.06976744	35.26570048	-18.80406696	0.00023319	0.001002581
Gpr3	29	3.62263432	1.815311468	-1.807322851	0.00023534	0.001011543
Pbx2	61	2.578293761	1.893061058	-0.685232703	0.00024042	0.001033069
Mir7008	2	47.5	28.12861439	-19.37138561	0.00024086	0.001034667
Bcl9l	25	3.859183824	3.123824574	-0.73535925	0.00024106	0.001035221
Gprasp2	4	30.40099329	41.04938272	10.64838943	0.00024489	0.001051334
Rbm43	34	1.881386312	0.834407786	-1.046978526	0.00024606	0.001056075
Kbtbd8	56	1.864952898	1.377458241	-0.487494656	0.00024681	0.001058953
Col19a1	5	35.77020797	28.6070442	-7.163163772	0.00024901	0.001068121
Clec1a	4	65.40296329	56.54259122	-8.860372069	0.00025011	0.001072209
Tmem119	3	6.818181818	17.14285714	10.32467532	0.00025007	0.001072209
Itga8	38	14.18287582	14.74055663	0.5576808	0.00025423	0.001089551
310030G06F	16	8.776326093	4.82698502	-3.949341073	0.00025486	0.001091935
Podxl	71	2.808305614	1.747013034	-1.06129258	0.00025526	0.001093305
Scgb1c1	2	16.66666667	7.961482961	-8.705183705	0.0002556	0.001094447

Sfxn3	11	2.912239523	2.203585478	-0.708654045	0.0002562	0.001096709
Gdf3	5	74.4818639	66.08629206	-8.395571836	0.00025657	0.001097949
Slc36a4	64	1.395632574	0.94095833	-0.454674243	0.00025771	0.001102525
Zrsr2	21	19.92404937	15.76137573	-4.162673637	0.00026028	0.001113186
Il13ra1	44	17.95678893	15.16537101	-2.791417928	0.00026097	0.001115817
Pxdc1	30	1.756248338	1.413608931	-0.342639407	0.00026314	0.001124758
Prdm2	93	1.73328413	2.592020011	0.858735881	0.0002661	0.001137066
Plekhs1	4	46.69709662	54.71194404	8.014847425	0.00026862	0.00114753
Haglr	31	47.86790227	45.46904192	-2.398860342	0.00026903	0.001148607
Gm6537	6	12.27082071	4.32308518	-7.947735533	0.00026898	0.001148607
Syt6	85	27.16249046	29.58199655	2.41950609	0.00027164	0.001159419
300017J02R	4	48.27711789	37.29224377	-10.98487412	0.00027293	0.00116428
Slc40a1	28	4.197808116	3.775985176	-0.42182294	0.00027294	0.00116428
Zfp444	28	3.433575861	2.015385397	-1.418190464	0.00027703	0.00118131
Slc24a4	71	7.104816553	8.961528751	1.856712199	0.00027709	0.00118131
Hap1	29	1.995676506	1.219797782	-0.775878724	0.00027726	0.00118166
'00012D16F	1	86.2745098	65.17857143	-21.09593838	0.00027903	0.00118888
Tmod1	72	2.170076202	1.616606141	-0.553470061	0.00028011	0.001193134
Nono	10	10.59927635	17.10883384	6.509557498	0.00028233	0.001202235
Steap1	95	2.235531465	1.81061366	-0.424917806	0.00028313	0.001205142
Sipa1l3	5	17.7162596	10.13372453	-7.582535069	0.00028318	0.001205142
Etv1	9	12.95161626	19.89823701	6.946620751	0.00028629	0.001218021
Klhl15	122	14.66800898	13.44427345	-1.223735528	0.00028795	0.001224749
Zmat1	24	18.61167291	15.29625348	-3.31541943	0.00029042	0.00123489
Ccdc69	29	3.185606842	2.701944421	-0.483662421	0.00029188	0.001240748
Ddah1	53	2.554013638	1.820648778	-0.73336486	0.00029394	0.001249118
Irak1	24	20.17715777	17.01957924	-3.157578524	0.00029639	0.001259168
Mettl7a1	8	5.247656373	3.797735787	-1.449920586	0.00029693	0.001261131
Wbp1l	3	71.07385172	87.51931234	16.44546063	0.00029753	0.001263276
Dpep2	5	73.48513302	80.34384216	6.858709139	0.00029877	0.001268206
Pnmt	5	57.15554229	50.65819985	-6.497342436	0.00029948	0.001270849
Gm5095	3	33.15755997	24.48586813	-8.671691845	0.00029959	0.001270923
Kcnk15	71	14.55459895	16.73367801	2.179079063	0.00030055	0.001274663
Lypd1	27	11.69222042	9.871548632	-1.820671791	0.00030205	0.001280622

Dusp23	35	3.926027354	2.85199567	-1.074031684	0.00030552	0.00129499
Camk2a	6	34.76458751	40.36760309	5.603015586	0.00030669	0.001299561
Arap3	25	5.964065193	4.498412765	-1.465652429	0.00030699	0.001300469
Mir26b	6	78.50834876	72.5559929	-5.952355855	0.00030818	0.001305127
Prr23a3	6	77.32911024	83.10686621	5.777755975	0.00031014	0.00131304
Brsk1	34	14.35679537	19.72967049	5.372875124	0.00031034	0.001313527
Lrrc7	10	73.13362035	67.37948638	-5.754133962	0.00031095	0.001315703
Mageh1	11	34.35680938	30.85968417	-3.497125212	0.00031321	0.001324879
Ptpn1	83	6.162281357	4.581409223	-1.580872133	0.00031578	0.001335359
Gm9731	4	89.27326659	79.4198972	-9.853369395	0.00031638	0.001337519
Ralyl	18	45.13027501	48.25144817	3.121173152	0.00031663	0.001338198
Lmod3	10	70.25588312	64.43136728	-5.824515832	0.0003214	0.00135797
Lingo1	2	97.34042553	92.09472468	-5.24570085	0.00032266	0.001362899
Olfr523	3	50.69834936	39.78724099	-10.91110837	0.00032309	0.001364306
Mir6968	18	94.76492872	91.84870393	-2.916224796	0.00032542	0.00137375
Tle6	7	22.10081438	16.65355169	-5.447262693	0.00032582	0.00137504
Mgat5b	109	10.5582392	10.03684656	-0.521392643	0.00032777	0.001382905
Cd59a	7	18.95687355	14.7130945	-4.24377905	0.00032809	0.001383858
Tmem63c	23	27.32519422	24.01577337	-3.309420853	0.00032898	0.00138719
Mir5132	25	20.23605084	17.15569748	-3.080353356	0.00032932	0.001388233
Cldnd1	61	3.206943006	2.679572457	-0.527370548	0.00032968	0.001389354
333411C07F	5	51.01123596	62.02629675	11.01506079	0.00033099	0.001394475
Kcna1	7	44.54749424	49.88396115	5.336466911	0.00033332	0.001403904
Hand1	24	57.12574852	60.28497915	3.159230624	0.00033389	0.001405893
Bod1	66	1.660705765	1.1472378	-0.513467965	0.00033624	0.001415391
Slc7a4	6	4.781854799	2.306432394	-2.475422405	0.00033906	0.00142682
Htra1	56	5.574665813	4.438859001	-1.135806812	0.00034019	0.001431157
10057M21I	35	2.189514866	1.03934825	-1.150166616	0.00034062	0.001432569
Lzts3	46	1.364924956	1.132988955	-0.231936001	0.00034102	0.001433857
Tspan7	24	17.36933694	14.61640793	-2.752929013	0.00034525	0.001451221
Taz	16	17.63065623	14.62527379	-3.005382444	0.00034546	0.001451668
Nmur1	2	46.94467706	37.38619557	-9.558481491	0.00034597	0.0014534
Gm10649	64	2.086572255	1.428671385	-0.65790087	0.0003472	0.001458157
Snrpb2	47	1.389341647	1.720174322	0.330832675	0.00035036	0.001470992

410004P03F	1	37.14285714	17.12328767	-20.01956947	0.00035067	0.001471904
Il17re	2	54.45544554	42.33333333	-12.12211221	0.00035245	0.00147896
Lyn	28	1.303075362	0.821356152	-0.48171921	0.00035345	0.001482726
Osgin2	111	1.483903058	1.116522749	-0.36738031	0.00035529	0.001489989
330070K13F	1	10.25641026	31.53153153	21.27512128	0.00035639	0.001494209
Kat2a	31	2.386170133	1.716148188	-0.670021945	0.0003569	0.001495899
Aldoart1	1	96.52173913	82.75862069	-13.76311844	0.00036028	0.001509628
Mir873a	2	93.4004884	83.33333333	-10.06715507	0.0003624	0.001518094
Sptan1	70	1.332926821	0.859127707	-0.473799114	0.00036266	0.001518737
Hs3st3a1	27	20.77796717	18.65785122	-2.120115952	0.00036364	0.0015222
330403A02F	61	10.59315379	10.3507616	-0.242392189	0.00036369	0.0015222
Phyh	8	2.860169492	6.969741494	4.109572002	0.00036522	0.001528155
Plin5	14	6.038185494	4.540371809	-1.497813685	0.00036601	0.001531015
Gfap	11	97.39635339	94.24332278	-3.153030605	0.00036676	0.001533723
Rasd2	96	20.15246013	21.70109016	1.548630024	0.00036727	0.001535405
Golm1	11	12.41845495	6.264912517	-6.153542432	0.00036795	0.00153781
Ppp1r42	3	46.17814085	57.23761723	11.05947638	0.00037139	0.001551763
Slfn9	12	4.53377056	2.080805145	-2.452965416	0.0003719	0.001553456
Ikzf5	33	1.535345105	1.090504561	-0.444840544	0.00037363	0.001559803
Lrr1	28	1.420839633	0.46390311	-0.956936523	0.00037356	0.001559803
Osbpl6	85	1.985480751	2.494595887	0.509115135	0.00037403	0.001561011
Fam83h	72	4.781026995	3.990483936	-0.790543059	0.00037484	0.001563967
Bambi	71	11.35505192	11.48324582	0.128193908	0.00037532	0.00156551
Hic1	126	29.93840807	29.50077761	-0.437630465	0.0003755	0.001565814
Grin3b	43	14.46578297	17.89019228	3.424409312	0.00037633	0.00156882
Fancd2	1	9.972299169	3.428571429	-6.54372774	0.00037704	0.001571332
Ercc2	38	1.281717386	0.839339262	-0.442378124	0.0003783	0.001576151
Vac14	28	9.913726397	6.731360374	-3.182366024	0.00037922	0.001579518
Tmem159	2	8.06402439	0.657894737	-7.406129653	0.00037953	0.001580353
Echdc2	35	2.247503987	1.339676889	-0.907827098	0.00038167	0.001588846
Wdr81	30	3.018441447	1.567938542	-1.450502905	0.00038562	0.001604815
Hes5	90	15.3801877	16.57844012	1.198252425	0.00038576	0.001604938
Lzts2	57	2.32912033	1.598761896	-0.730358434	0.00038607	0.001605794
Pphln1	20	1.476554538	0.871437297	-0.605117241	0.00038621	0.001605917

Hecw1	1	30.79847909	18.18181818	-12.61666091	0.00038775	0.001611847
Kif5c	72	4.921770108	4.411866609	-0.509903499	0.0003884	0.001613917
Drd4	18	21.55630271	19.70474567	-1.851557043	0.00038847	0.001613917
Dnal1	3	1.360544218	6.858237548	5.49769333	0.000391	0.001623975
Cacng8	3	85.49792978	75.28822206	-10.20970772	0.00039119	0.00162432
Gm15471	3	70.27255347	80.32833616	10.05578269	0.00039184	0.001626569
Sgip1	10	28.22461858	32.95399195	4.729373368	0.00039293	0.001630603
Smarca2	67	2.17688053	1.545503153	-0.631377377	0.0003943	0.001635848
Aldh1a3	47	12.17063181	11.64216189	-0.528469919	0.00039732	0.00164792
Mospd1	4	28.38416574	20.71766164	-7.666504103	0.00039902	0.001654478
Irf9	10	15.71153249	19.47679093	3.765258442	0.00040445	0.001676543
Zcchc10	6	31.64253682	23.48564012	-8.156896704	0.0004046	0.001676686
Acacb	1	76.98412698	57.2327044	-19.75142258	0.00040811	0.001690721
Spag8	11	39.98068522	43.38331323	3.402628005	0.00040905	0.001694178
Amer1	11	22.0341825	18.09922736	-3.934955141	0.00041006	0.001697841
33416M07I	14	91.77730668	88.27539986	-3.501906813	0.00041103	0.001701393
Hrh3	46	6.491265363	5.861804312	-0.629461051	0.00041143	0.00170256
230013L22R	15	1.801856459	1.01318951	-0.788666949	0.00041792	0.001728923
Cdc42bpg	54	2.009428648	1.547940406	-0.461488242	0.00041916	0.001733594
Cobl	77	2.46698546	1.891585539	-0.575399921	0.00041992	0.001736224
Tmc4	4	83.78594694	77.93966769	-5.846279249	0.00042182	0.001743617
Rsph14	6	87.8410126	81.87022177	-5.970790837	0.00042508	0.001756567
Zfp521	34	5.129213191	8.254346024	3.125132833	0.00042556	0.001758072
Kcnh3	100	17.19592368	16.5661236	-0.629800086	0.00042731	0.001764784
Zfyve28	69	7.376052179	6.855851475	-0.520200704	0.00042933	0.001772652
Sh3pxd2a	80	1.833810102	1.474378788	-0.359431314	0.0004318	0.001782334
Mir138-2	5	79.43281782	72.08468143	-7.34813639	0.00043349	0.00178882
Phf6	51	20.01991121	17.50273348	-2.51717773	0.00043432	0.001791725
Stx1b	7	15.64746963	9.811403779	-5.836065846	0.00044174	0.001821847
Cpsf4l	9	86.41544381	82.61499537	-3.800448438	0.00044265	0.001825083
Six6	29	24.70381641	23.35416159	-1.349654826	0.00044351	0.001828112
Sort1	143	2.911662114	2.432560091	-0.479102023	0.0004481	0.001846499
Mst1r	3	65.95875749	73.25781971	7.299062223	0.00044838	0.001847149
Otop1	17	6.908411439	8.912811326	2.004399887	0.0004491	0.001849582

Polr1e	16	1.580125313	1.04816044	-0.531964873	0.00045252	0.00186314
Rinl	4	74.66817496	82.27006076	7.601885796	0.0004548	0.001872026
Nphs1	6	29.52368427	24.21581666	-5.307867605	0.00045559	0.001874721
Zfp647	38	1.938497263	1.484697193	-0.45380007	0.000456	0.001875919
Cda	13	3.886335508	2.389687619	-1.496647889	0.00045728	0.001880642
Cyp7b1	16	59.19912093	61.95726207	2.758141138	0.00046226	0.001900373
Zfp579	19	4.389472359	3.535550557	-0.853921802	0.00046234	0.001900373
Fhod3	1	32.0754717	7.462686567	-24.61278513	0.00046266	0.001901188
Fbxw22	1	63.63636364	37.5	-26.13636364	0.00046982	0.00192951
)30524B15F	10	78.17511103	73.58658318	-4.588527848	0.00046976	0.00192951
Daglb	45	1.496390158	0.997104685	-0.499285473	0.00047271	0.001940819
Scube2	54	10.3468226	9.521452657	-0.82536994	0.00047339	0.00194308
Fam132b	14	7.751861413	4.746817469	-3.005043944	0.00047575	0.001952214
320431F20F	6	22.67237977	25.29055482	2.618175051	0.00047638	0.001954281
Arnt2	48	14.78924808	13.94205574	-0.847192336	0.00047698	0.001956163
Thg1l	13	1.545779985	2.244756531	0.698976546	0.00047832	0.001961117
Smo	52	1.853550972	1.469955857	-0.383595115	0.00048231	0.001976924
Nlrp5	2	68.69172354	57.4719878	-11.21973574	0.00048353	0.001981361
Frmpd1	72	1.874357487	1.207786765	-0.666570722	0.00048405	0.001982937
Klk9	10	93.89825663	89.98042885	-3.91782778	0.00048757	0.001996265
Rgcc	38	2.807821081	1.878615637	-0.929205445	0.00048747	0.001996265
Cbx7	56	1.426624027	1.057067592	-0.369556435	0.00048958	0.002003931
Abhd17c	121	1.014228117	1.274921281	0.260693164	0.00049943	0.002043677
Mis18bp1	55	1.39279504	0.832630812	-0.560164229	0.00050192	0.002053311
Peg3	8	72.52306101	76.87145673	4.348395724	0.00050253	0.002055199
Gal3st3	16	38.19808241	40.88252946	2.684447053	0.00050395	0.002060438
Dcxr	27	2.491342668	1.798263992	-0.693078676	0.000505	0.002064188
Brcc3	5	23.5443038	12.64705882	-10.89724497	0.00050521	0.002064471
Eps8	44	1.615878969	1.170027204	-0.445851764	0.00050749	0.002073209
H2-Bl	20	16.48918634	18.07428795	1.585101613	0.00050781	0.002073932
425401B19F	3	83.3972751	86.95780185	3.560526754	0.00050895	0.002077983
Irx3	73	2.383140773	1.727999543	-0.65514123	0.00051385	0.002097423
Kctd7	39	4.081778367	5.120616631	1.038838264	0.00051495	0.002101312
Polr3e	65	1.006321967	1.392068976	0.385747009	0.00051874	0.002116219

Ube2t	2	5.519480519	1.143647103	-4.375833416	0.00052896	0.00215731
Gm266	73	27.55151639	29.18297016	1.631453765	0.00053126	0.002166083
Fbxo36	43	1.553470198	1.4716593	-0.081810898	0.00053231	0.002169766
Prnd	4	33.41880342	27.45772947	-5.96107395	0.00053333	0.002173306
St8sia4	15	7.048225219	10.32115489	3.272929673	0.00053517	0.002180181
Prom2	23	17.97542906	21.2803971	3.304968034	0.00054262	0.002209957
Ube2i	37	0.645504411	1.406768051	0.76126364	0.00054564	0.002221626
Gm15816	20	5.084894893	3.977054471	-1.107840422	0.00054601	0.00222253
Stx1a	34	2.473617818	2.018841653	-0.454776166	0.00054649	0.00222385
Neurod2	10	46.70900595	51.96469378	5.255687828	0.00054973	0.002236419
Parp8	66	1.477630271	0.97035699	-0.507273282	0.00055149	0.00224293
Tbl1xr1	129	1.767272841	1.44551867	-0.321754171	0.0005589	0.002272458
Cttn	1	38.0952381	21.32701422	-16.76822388	0.00055946	0.002274109
Zfhx3	77	3.937076192	2.744473414	-1.192602777	0.00055997	0.002275532
Fam212b	22	2.256616269	1.520310528	-0.736305741	0.00056374	0.002290231
700026L06R	18	5.416374773	3.073312646	-2.343062127	0.00056398	0.002290594
Tlx2	78	16.21683773	18.54931588	2.332478144	0.0005655	0.002296118
Ppp4r4	104	5.927860373	5.626740385	-0.301119987	0.00056603	0.002297632
933413J09R	2	15.69767442	5.140186916	-10.5574875	0.00057018	0.00231384
Zfp354c	11	2.643848457	1.627384514	-1.016463943	0.00057292	0.002324315
Rbbp8nl	16	81.66120303	80.09430236	-1.566900664	0.00057602	0.002336235
930430F08R	22	1.546298944	2.116520999	0.570222055	0.00057907	0.002347962
Cpt1c	4	16.3015577	9.276220231	-7.02533747	0.00058561	0.002373815
Araf	10	21.2671586	17.03441643	-4.232742168	0.00058704	0.00237898
Mir7018	5	95.08653651	91.41483211	-3.6717044	0.00058744	0.002379928
033406O09R	1	35.36585366	15.6626506	-19.70320306	0.00058955	0.002387809
Lhx5	52	20.68316732	24.01632694	3.333159619	0.00058997	0.002388841
Arg2	24	4.767596835	3.042345457	-1.725251378	0.00059202	0.00239649
Fbn1	24	18.84908778	18.56133604	-0.287751732	0.00059905	0.002424279
Krt75	9	73.26811564	65.50410919	-7.764006446	0.0006024	0.002437189
Morn4	5	7.306704659	3.966165236	-3.340539423	0.00060639	0.00245262
700067K01R	30	95.29342186	95.79225265	0.498830782	0.00061197	0.00247453
Mir9-1	13	39.72975188	48.90415538	9.174403507	0.00061295	0.002477815
Gm7854	35	2.147490428	1.352322741	-0.795167688	0.00061714	0.002494063

Upk1a	2	29.56785663	41.07360793	11.50575131	0.00061811	0.002496673
310032G03R	18	20.13722834	16.46433016	-3.672898181	0.00061813	0.002496673
210417A02R	4	34.94889084	26.73984825	-8.209042583	0.00061931	0.002500751
Col9a3	35	22.78510121	25.16187414	2.37677293	0.00062084	0.002506239
Gm41	3	82.67556973	74.77517952	-7.90039021	0.00062317	0.002514971
Atp6v1c2	20	7.612592033	13.25443719	5.641845155	0.00062721	0.002530572
Gdf1	9	63.614954	58.41701413	-5.197939873	0.00063174	0.002548138
Tbx4	10	34.24828213	30.62565017	-3.622631955	0.0006403	0.002581974
Gm11981	1	98.8372093	92	-6.837209302	0.00064958	0.002618648
Pdcd6ip	59	2.525549141	1.345466494	-1.180082647	0.00065133	0.002625009
130043K22R	9	10.81478886	14.95790497	4.143116105	0.00065447	0.002636924
Golga7b	41	25.08908772	24.07676868	-1.012319035	0.00065501	0.002638399
Tbx10	2	81.21216244	72.51863144	-8.693531002	0.0006565	0.002643647
Trim14	16	13.48949954	12.9306118	-0.558887735	0.00065776	0.002647498
Ace	46	5.158227878	3.652903304	-1.505324574	0.00065782	0.002647498
Cryl1	18	2.725631083	1.409198751	-1.316432332	0.00065832	0.002648799
Gm2027	1	49.31506849	63.20541761	13.89034911	0.000666	0.002678975
Ptk7	42	1.443525667	1.22047365	-0.223052018	0.00066879	0.002689465
Mtfp1	27	2.404731499	1.673865272	-0.730866227	0.00067124	0.002698575
Dlg3	34	30.65155175	26.87805678	-3.773494964	0.0006754	0.002714569
Elfn2	75	30.55272397	31.94088924	1.388165264	0.00067796	0.002724107
Slc9a9	2	49.75474684	66.7733523	17.01860546	0.00067904	0.002727698
Dnase1l1	8	16.83653794	11.75961569	-5.076922249	0.00068316	0.00274349
700020D05R	17	91.28477166	91.7746631	0.489891434	0.00068485	0.002749499
Ofd1	32	13.95148386	11.92193134	-2.029552525	0.00068513	0.002749889
Gabrg1	4	45.75688354	56.99891405	11.24203051	0.00069117	0.00277336
Fads2	55	3.481156969	2.548105978	-0.933050991	0.0006925	0.002777951
Bche	1	13.91752577	4.210526316	-9.706999457	0.00069768	0.002797955
Kcnmb4	92	2.629082611	4.155992847	1.526910236	0.00069972	0.002805363
Samd12	3	8.714247527	3.459119497	-5.255128031	0.00070046	0.002807599
Hsd17b6	1	100	90.43478261	-9.565217391	0.000702	0.002812981
Gemin8	12	17.64415409	13.92800112	-3.716152973	0.00070379	0.002819374
Zfp85	8	3.463673182	1.965003431	-1.498669751	0.00070454	0.002821634
Ndufa1	27	19.44642727	16.9615308	-2.484896466	0.00070694	0.002830449

Usp38	88	1.825038933	1.448482193	-0.37655674	0.00070809	0.00283429
Zfp618	116	1.447059389	1.724391441	0.277332052	0.00070864	0.002835411
Zfpm2	67	1.866529051	1.325703355	-0.540825696	0.00070876	0.002835411
Prkce	124	1.497024474	1.146115867	-0.350908607	0.00071398	0.002855549
Dnm1	40	2.239546192	1.726610666	-0.512935527	0.00071718	0.002867548
)33428G20F	82	96.28448315	96.89239526	0.607912107	0.00071763	0.002868582
Mir7230	7	71.10669232	64.87436989	-6.232322427	0.00071802	0.002869353
Colgalt1	16	3.012102194	1.268186956	-1.743915238	0.00072007	0.002876749
Ppdpf	13	3.620394376	0.84058795	-2.779806426	0.00072173	0.002882591
Mboat7	21	0.723882433	1.453384492	0.729502059	0.00072303	0.002887013
Phc1	83	1.856676289	1.271853995	-0.584822294	0.00072545	0.002895875
Tdgf1	6	70.50995025	64.55202482	-5.957925427	0.00072851	0.002907316
Zmynd15	16	2.754641067	1.636736523	-1.117904544	0.00073447	0.002930278
Fam92b	2	25.68741407	13.95819093	-11.72922314	0.00074038	0.002953084
Cnr2	1	77.96610169	58.20895522	-19.75714647	0.00074548	0.002972611
Arg1	4	28.09906103	22.06450265	-6.034558375	0.00074945	0.002987096
C1ql1	90	28.51300276	30.4433056	1.930302835	0.00074952	0.002987096
)30068K23F	4	41.0939178	31.84785092	-9.24606688	0.00075948	0.003025978
Card6	3	3.774348023	1.096965522	-2.677382501	0.00076509	0.003047477
Cep41	17	1.858540609	1.420703307	-0.437837301	0.00076878	0.003061351
Srcin1	69	3.976424131	3.075366595	-0.901057536	0.00077955	0.003103408
Adcy8	30	5.096331042	6.288650215	1.192319172	0.00078187	0.003111798
Zkscan16	3	43.38659813	54.67054901	11.28395088	0.00079168	0.003149985
)30556N09F	3	95.10703364	86.13731053	-8.969723106	0.00079464	0.003160902
Grm7	28	59.3338935	61.90792467	2.574031172	0.000795	0.003161469
Aoc3	2	84.10748177	72.40147783	-11.70600394	0.00079696	0.003168403
Gart	61	1.705509395	1.166211957	-0.539297439	0.00079939	0.003177212
Fut4	21	3.351079734	2.503330848	-0.847748886	0.00080388	0.003194206
Brinp1	26	56.18367012	58.76345265	2.579782523	0.00080722	0.003206578
Spaca1	4	89.8281062	82.86516854	-6.962937662	0.00081049	0.003218712
Gm11744	4	78.15741306	70.10739398	-8.050019081	0.00081352	0.003229872
Chad	21	52.0185158	47.91558657	-4.102929221	0.0008153	0.00323607
Krt76	5	84.01290853	77.76179336	-6.251115165	0.00081683	0.003241242
Klf5	50	1.371638741	0.803323939	-0.568314803	0.00082617	0.003277429

Gna15	4	18.27501103	24.35855382	6.083542787	0.00082989	0.003291289
Spata24	13	7.760094077	4.764015645	-2.996078431	0.00083464	0.003309042
Rbm48	30	3.807115385	2.261659913	-1.545455471	0.00083481	0.003309042
Fgf15	46	10.96483425	10.83954441	-0.125289846	0.00084089	0.00333132
Htr7	95	16.17270871	15.43481845	-0.737890258	0.00084073	0.00333132
333412C05F	7	6.971135016	5.238989209	-1.732145807	0.00084589	0.003350216
Efcab12	4	27.10409194	19.91718832	-7.186903621	0.00086252	0.003415165
Krt23	7	23.49142182	34.74062357	11.24920175	0.00086966	0.003441593
Pcdhga8	11	80.11352612	75.31269214	-4.800833985	0.00086964	0.003441593
Lrif1	18	5.009148631	6.195325641	1.18617701	0.00087459	0.003460157
Fam221a	22	4.473925212	6.028517127	1.554591915	0.00087824	0.003473657
Mtus1	50	13.35840109	11.83512627	-1.523274816	0.00088032	0.003480959
Ifrd2	12	1.742760263	1.264235843	-0.47852442	0.00088421	0.003495406
Taf1b	9	3.909903518	2.436272249	-1.473631269	0.00088462	0.003496054
Far2	8	7.330045363	4.639364464	-2.690680898	0.00088498	0.003496548
Prdm14	9	39.1534305	33.77877169	-5.374658807	0.0008936	0.003529228
Sned1	51	2.175876171	1.822657826	-0.353218345	0.00089373	0.003529228
Dcaf15	50	6.175951525	4.273240852	-1.902710673	0.00089424	0.003530271
Tssk3	4	76.00555894	79.94151982	3.935960878	0.00089663	0.003538755
Gm5478	1	44.62809917	24.3697479	-20.25835127	0.00090463	0.003569394
Tktl2	10	95.29988573	92.77490077	-2.524984953	0.0009158	0.003612472
Rprl3	10	48.48581368	44.07742715	-4.408386529	0.00091661	0.003614711
Clpsl2	7	76.346799	69.5861504	-6.760648601	0.00091807	0.003619481
Rab3b	22	22.31482973	21.18284054	-1.131989189	0.00091898	0.003622106
Mturn	57	0.992491081	1.588852146	0.596361065	0.00092909	0.003660975
Entpd6	44	1.324665	1.901779375	0.577114375	0.00093034	0.003664912
Ccdc42	3	62.51870919	56.37796888	-6.140740307	0.00093263	0.003672947
Grik5	24	11.55058431	9.96678479	-1.583799524	0.00093809	0.003693479
Rasip1	3	85.00792039	78.45129161	-6.556628783	0.00093907	0.003696336
CK137956	15	50.83188723	54.66106548	3.829178248	0.00094008	0.003699308
Eml1	85	5.96080776	5.507129614	-0.453678146	0.00094484	0.003717047
Prdx6	23	0.997168116	1.601169338	0.604001222	0.0009491	0.003732802
Map3k9	87	1.473669398	1.962472214	0.488802816	0.00094956	0.003733603
Mir6407	3	96.3697073	99.43181818	3.062110879	0.00095014	0.003734877

AI507597	6	79.53336331	73.75879702	-5.774566283	0.00095691	0.003760505
Zeb2	51	1.033288224	1.500679202	0.467390978	0.00095988	0.003771158
Slc2a9	8	32.42446781	28.65353741	-3.770930401	0.00096021	0.003771441
Efna3	26	3.092674641	2.007122158	-1.085552483	0.0009612	0.003773826
Ar	7	24.60170226	32.28510174	7.683399486	0.00096133	0.003773826
Tyro3	109	1.734585351	1.283937567	-0.450647785	0.00096793	0.003798715
Trpm7	55	1.461944812	1.590770215	0.128825403	0.00098267	0.003855551
Pir	6	32.279311	26.72679595	-5.552515051	0.00098519	0.003864379
Evc2	1	29.67032967	10.89108911	-18.77924056	0.00099268	0.003892747
Farp1	135	2.880627626	1.536710002	-1.343917624	0.00099829	0.003913662
Gm3716	5	19.72618523	14.58029316	-5.145892074	0.00100153	0.003925333
Slc25a41	6	63.48888655	53.8950228	-9.593863756	0.00100558	0.003940163
Gm44	2	75.43103448	64.81516057	-10.61587392	0.00101297	0.003968039
Zrsr1	23	82.40985229	85.92356052	3.513708231	0.00101574	0.003977822
Smc1a	8	29.24130664	20.702267	-8.539039639	0.00101857	0.003987873
Celf4	36	2.34237199	1.599397363	-0.742974627	0.00102642	0.004017512
Igflr1	9	90.84470683	87.15058492	-3.694121912	0.00102816	0.004023258
Cubn	5	35.0126045	40.83785628	5.825251778	0.00103209	0.004037564
Ltbp1	72	4.531408673	3.803634868	-0.727773805	0.00103247	0.004037978
'00113H08F	7	19.5564724	16.32048425	-3.235988145	0.00103682	0.004053893
Vangl2	56	2.783831055	3.705818318	0.921987263	0.00104203	0.004073188
Loxhd1	8	80.91384412	72.92205438	-7.991789746	0.00105129	0.004108305
Dact3	34	3.391867946	2.62334304	-0.768524906	0.00105348	0.004115753
Dok3	10	58.00550949	54.51773317	-3.487776318	0.00105586	0.004123971
Bin3	25	1.925844866	1.171622048	-0.754222818	0.00105954	0.004137219
Arhgef5	42	2.426795713	2.063312983	-0.36348273	0.00106152	0.004143834
Nrsn2	18	37.64772572	36.06782915	-1.579896571	0.00106195	0.004144422
Hmgb1-rs17	6	82.47950089	79.82221083	-2.657290062	0.0010666	0.004160026
Dpy19l3	34	2.380139925	1.415668229	-0.964471696	0.0010668	0.004160026
Mir3057	6	95.7581572	92.38337159	-3.374785604	0.00106667	0.004160026
Nr1h3	13	4.627079371	3.269683084	-1.357396287	0.00106883	0.004166864
Pde10a	70	21.71958852	23.52146674	1.801878222	0.00107143	0.004175879
Rab19	3	18.36734694	8.441606398	-9.925740541	0.00107226	0.004177987
Dab2	6	6.381995382	3.019024936	-3.362970447	0.00107553	0.004189621

Pdf	57	0.72728412	0.952186504	0.224902385	0.00109875	0.004278931
Emilin3	69	28.55188899	30.35472769	1.802838702	0.00110018	0.004282241
Fam89a	61	6.934593298	6.258682321	-0.675910977	0.0011	0.004282241
Notch3	13	13.51018053	16.68471772	3.174537188	0.00110787	0.004311039
Fkbp9	47	1.712193918	1.405506934	-0.306686984	0.0011092	0.004315048
Mir1948	2	77.47274323	69.14167694	-8.331066296	0.00111659	0.004342043
Igfbp2	46	13.31509209	16.87300659	3.557914499	0.00111673	0.004342043
Hypk	16	1.021417698	0.436964274	-0.584453425	0.00112519	0.004373795
Chmp4c	12	5.463800269	3.353395229	-2.110405041	0.00112735	0.004381039
Kcnmb4os2	9	9.706554893	13.94323846	4.236683572	0.00113725	0.004418321
Rnf112	5	15.54477387	10.71415623	-4.830617643	0.00114304	0.004439637
Lrrc74a	3	85.75498575	77.59489303	-8.160092725	0.00114418	0.004442895
Aph1c	18	1.994315474	4.056539702	2.062224228	0.00114944	0.004462151
Limk2	79	11.86991855	12.94777042	1.07785187	0.00115609	0.004486766
Crygn	8	82.53221341	79.91521432	-2.616999083	0.00115783	0.004492332
Stoml1	14	2.041076456	0.962608515	-1.07846794	0.00116218	0.004508008
Adm	2	7.09201568	2.817041115	-4.274974565	0.00116252	0.004508163
Kcnn4	1	6.756756757	1.204819277	-5.55193748	0.00116424	0.00451362
Ildr2	44	0.824515602	1.347686736	0.523171133	0.00116801	0.004527031
Dlg5	76	2.267439271	1.83980821	-0.427631061	0.0011688	0.004528889
Cdkn1c	6	32.55060729	41.16280659	8.612199299	0.00118099	0.004574934
Erich6	8	16.5745029	19.38732406	2.81282116	0.00118658	0.004595386
Ints1	44	1.93385014	1.341057087	-0.592793054	0.00119019	0.004608128
Bcl11a	63	12.38936157	11.5634468	-0.82591477	0.00119431	0.004622879
Rgs2	17	1.439815111	0.671428896	-0.768386215	0.00119739	0.004633554
Dhh	23	7.495666446	4.666224402	-2.829442044	0.00119905	0.004638783
Ero1lb	47	1.836389974	1.130039072	-0.706350901	0.00120541	0.004662161
Lysmd2	74	4.005911449	1.983017507	-2.022893941	0.00121009	0.004678996
Nmu	14	32.46508775	29.94204088	-2.523046864	0.00121493	0.004695623
Mthfr	22	4.839285734	2.744284149	-2.095001584	0.00121503	0.004695623
Cbr3	47	2.981241132	2.426626408	-0.554614724	0.00121541	0.004695886
Crlf1	90	1.770823117	1.426627774	-0.344195343	0.0012265	0.004737466
)00008C10F	55	11.14764108	9.638909361	-1.508731719	0.00122692	0.004737867
Krt79	18	92.51459254	90.33033672	-2.184255816	0.00123075	0.004751384

Nav2	85	11.17193454	12.42609495	1.254160411	0.00123544	0.004768247
Arhgap39	50	11.19752162	8.577626989	-2.619894635	0.00124339	0.004797671
Man1b1	9	2.153239525	1.186100702	-0.967138823	0.00124595	0.004805796
Bpifb2	2	76.37280283	64.25062657	-12.12217627	0.00124615	0.004805796
Elmsan1	90	1.566073602	1.121357903	-0.444715699	0.00124829	0.004812777
Mavs	9	3.990618046	1.801728548	-2.188889498	0.00125152	0.004823948
Hepacam	2	95.32286599	87.3538961	-7.968969885	0.00125312	0.004828866
Arrdc1	27	9.454174729	7.475094148	-1.979080582	0.00125405	0.004831188
Ndufa11	21	1.717645356	1.086505314	-0.631140043	0.00126295	0.004864177
Ttyh2	39	1.91549173	1.356893285	-0.558598444	0.00127216	0.004898348
Wnk2	58	17.87673275	20.96660638	3.089873632	0.00127529	0.004909149
Gm6654	7	98.020688	94.1327736	-3.8879144	0.00127945	0.004923837
Ispd	38	1.584341696	1.152615431	-0.431726264	0.00128382	0.004939375
Syt15	34	19.26491994	18.84403135	-0.420888595	0.0012853	0.004943768
Psma8	8	65.30689184	71.35438638	6.047494539	0.00128853	0.004954912
Lypd8	2	55.52723497	43.07152665	-12.45570832	0.00128932	0.00495665
Rnf130	50	1.146419617	1.775469263	0.629049647	0.00129367	0.004972059
Trdmt1	26	1.784764149	1.235327213	-0.549436936	0.00129832	0.00498864
Mfng	4	21.39590447	15.63073674	-5.76516773	0.00130614	0.005017372
Gfra3	14	28.56628978	26.56384228	-2.002447499	0.00131402	0.005046297
Shc1	19	8.069075119	5.8046298	-2.264445319	0.00133119	0.005110909
Tmem47	23	53.44886874	56.29956938	2.850700636	0.00133333	0.005117782
Shf	36	2.780912946	1.708044842	-1.072868104	0.00133455	0.005121115
Tmem9	15	8.41433929	6.887137109	-1.52720218	0.00133698	0.00512911
Fam63b	51	1.282880198	0.718297947	-0.564582251	0.00135357	0.005191406
430411K18R	7	94.78234528	90.52223298	-4.260112305	0.00135508	0.00519584
Tmem37	20	3.466088148	1.404702793	-2.061385355	0.00135799	0.00520561
Ccng2	41	1.812254958	1.15626121	-0.655993748	0.00136679	0.005235276
Col4a2	36	1.680554519	1.058369159	-0.62218536	0.00136668	0.005235276
Cebpa	105	1.510391696	1.094555921	-0.415835774	0.00136628	0.005235276
Rnf224	4	84.90471229	89.4905098	4.585797508	0.00137111	0.005250435
Mtnr1a	10	11.06109909	15.66398767	4.602888579	0.00137393	0.005259872
Acss2os	2	95.11452242	90.25	-4.864522417	0.00137613	0.005266915
Fam110c	23	2.681047977	1.735001405	-0.946046572	0.00137947	0.005278299

Dhrs11	45	1.478546707	0.841332672	-0.637214035	0.0013812	0.005283556
'00001K23F	2	77.27272727	61.50956768	-15.76315959	0.00138547	0.005298508
Mir3102	8	89.09167471	83.50824234	-5.583432365	0.00138763	0.005305391
Olfr215	3	59.45372865	49.72376048	-9.729968177	0.00139247	0.00532249
Casr	4	47.981801	54.76226948	6.780468478	0.00140759	0.005378887
Pnldc1	13	95.014164	96.4315724	1.417408395	0.00140946	0.005384634
Crb3	27	4.367722363	2.881398638	-1.486323725	0.00141557	0.00540658
Cxcr2	1	90.75630252	75.78125	-14.97505252	0.001417	0.005410634
Cbln1	40	52.33686628	54.14519554	1.808329258	0.00141986	0.005420139
'00021N21F	4	56.13680819	47.88839725	-8.248410943	0.00142447	0.005436309
Ccdc14	18	2.061004053	0.827425714	-1.233578339	0.00142554	0.005438991
Spata13	6	5.590280668	3.002219982	-2.588060686	0.00142828	0.005448042
Dgka	30	2.5181851	1.335587574	-1.182597526	0.0014361	0.005476456
Mesp2	18	34.90803881	30.91996939	-3.98806942	0.00143789	0.005481853
Zfp503	36	4.419037335	3.173499888	-1.245537447	0.00145519	0.005546343
Zranb2	84	1.278131399	0.971972835	-0.306158564	0.0014559	0.005547613
Filip1	6	13.74490749	9.847680387	-3.897227108	0.00145808	0.005554495
Pim2	35	15.76792253	13.84061801	-1.927304521	0.00146028	0.005561426
Hoxa7	2	19.14893617	36.76470588	17.61576971	0.00146272	0.005569284
AU015836	5	82.75591892	75.94768665	-6.808232279	0.00146482	0.005575822
Tpd52l1	36	1.813226657	0.81610917	-0.997117487	0.00149192	0.005677511
Mapkapk3	18	7.386078277	5.223217004	-2.162861273	0.00149512	0.005688207
Cyp26c1	12	93.22698246	88.51594136	-4.7110411	0.00150226	0.005713897
Phc3	30	1.048213627	1.235498388	0.187284761	0.00150322	0.005716055
Gm13090	15	94.40526814	90.98798513	-3.417283016	0.0015044	0.005719077
Naa10	6	30.47915959	22.66733823	-7.811821366	0.00150651	0.00572561
Wipf3	2	70.95709571	63.66159629	-7.295499416	0.00152015	0.005775942
Hmgb4	4	82.4857812	77.1459417	-5.339839494	0.00152427	0.005790076
Hr	24	2.159297443	1.427070619	-0.732226824	0.00152486	0.005790848
Artn	8	8.546948369	5.547529263	-2.999419106	0.00152719	0.005798189
Bud13	28	1.52030993	0.934910497	-0.585399433	0.00152859	0.005801999
Hmgn3	9	4.669183663	2.803674323	-1.86550934	0.00154323	0.005856039
Hfe	3	8.823547785	4.810631439	-4.012916346	0.00155274	0.005890634
Lrrc52	1	84.21052632	70.94017094	-13.27035538	0.00155499	0.00589764

Asb6	20	2.65373522	2.102994917	-0.550740302	0.00156386	0.005929735
Scly	31	11.79486396	10.64965641	-1.145207552	0.00156497	0.005932399
930502E18F	3	88.47883167	81.61588229	-6.86294938	0.00158105	0.005991833
Gm6815	1	64.46280992	45.13888889	-19.32392103	0.00159331	0.006036733
BC048507	15	87.05495628	83.69530503	-3.359651248	0.00159615	0.006045927
Ccdc37	12	21.91502781	22.42147239	0.506444581	0.00159882	0.006054469
330045A20F	1	29.24528302	12.78195489	-16.46332813	0.00160284	0.006068149
Rbp4	60	1.611265156	1.35991782	-0.251347335	0.00162476	0.006149544
Kdm3b	53	2.251053798	1.750198341	-0.500855457	0.00162857	0.006162343
Spag6	17	8.053096587	10.90230072	2.849204137	0.00163474	0.006184121
Mir93	1	66.66666667	55.63380282	-11.03286385	0.00163823	0.006195709
Pcif1	62	0.901296124	1.29005804	0.388761916	0.00164565	0.006222191
Zcchc11	21	1.19384692	2.264438725	1.070591805	0.00165115	0.006241346
Frat1	48	1.661071935	1.174679381	-0.486392555	0.00165386	0.006249999
Abhd13	43	0.816352805	1.124795046	0.308442242	0.00165654	0.006258505
Apex2	16	18.22103763	22.00240462	3.781366988	0.0016577	0.006261302
Prmt2	8	2.636507946	1.192262967	-1.444244979	0.00166206	0.006276123
Sptbn4	119	5.352003714	4.889969609	-0.462034105	0.00166634	0.006290661
Chrnb4	17	8.37052872	7.052906253	-1.317622467	0.00166728	0.006292594
Asb5	3	97.164373	94.22717104	-2.937201956	0.00167561	0.006322418
Dhx15	55	1.191670186	0.775345431	-0.416324755	0.00167945	0.006335265
Rnf181	13	1.666757354	0.608798873	-1.057958481	0.001685	0.006352935
Tonsl	10	2.717709949	4.219054717	1.501344768	0.00168497	0.006352935
Gsdmd	11	1.858939067	0.860651011	-0.998288056	0.00169746	0.006398272
Ints4	7	2.051566932	0.400534045	-1.651032887	0.00169825	0.006399607
Sf3b2	28	2.005191081	1.513072098	-0.492118983	0.00170548	0.006425225
Spo11	23	87.79847964	85.87250894	-1.925970695	0.00172755	0.006506703
Klhl10	4	95.91702377	91.38095238	-4.536071393	0.00173823	0.006545239
Dtnb	109	1.993911616	1.498378551	-0.495533065	0.00174245	0.006559451
Adra1a	2	10.85106383	19.90613553	9.055071701	0.00175023	0.006587036
B3gntl1	12	2.933108345	2.004521875	-0.92858647	0.0017541	0.00659991
Tigd3	43	2.672236111	1.76419915	-0.908036962	0.00175614	0.006605904
Hoxb1	9	47.19126373	41.73622018	-5.45504355	0.00177515	0.006675705
Exoc8	95	1.01610503	1.138874321	0.122769291	0.00178722	0.006719374

Kcnma1	108	25.67043734	24.81106901	-0.859368325	0.00180141	0.006770977
BC026585	17	35.79104404	40.41845935	4.627415311	0.00181424	0.006817459
Mir191	40	1.725336383	1.167992521	-0.557343863	0.00182602	0.006859976
Fbxo22	24	2.454150734	3.520390341	1.066239608	0.00182863	0.006867992
Prodh2	2	74.70410628	64.27631402	-10.42779226	0.00183396	0.006886249
Catsper3	2	80.14308585	84.8605441	4.717458249	0.0018387	0.006902282
Hspa14	34	2.112966738	1.562986335	-0.549980403	0.00184049	0.006907259
Tmem255b	6	6.43505451	10.25799412	3.822939607	0.00185692	0.006967133
Tsc22d4	4	1.835045001	3.239285655	1.404240655	0.00186491	0.006995328
Tox2	4	76.50335227	70.22874682	-6.274605445	0.00187077	0.0070155
Msx2	39	41.41082674	44.21621379	2.805387048	0.00187788	0.007040358
P2ry14	1	78.8	65.87677725	-12.92322275	0.00188513	0.007065733
330009N23R	47	4.651097325	3.998872536	-0.652224789	0.00189042	0.007083779
Cartpt	15	26.25191604	28.85447595	2.602559914	0.0018936	0.007093865
Cnga3	8	50.59835651	48.68316096	-1.915195549	0.00189694	0.007104582
Cebpb	124	3.699964407	2.887716651	-0.812247756	0.00190486	0.007132411
Slc9a3r2	55	1.172242184	0.887403076	-0.284839108	0.00190707	0.007138848
Elf5	3	27.64380536	20.19019644	-7.45360892	0.00191093	0.007151494
Rbbp7	19	17.11496126	13.69930845	-3.415652807	0.00191389	0.007160751
Xirp1	9	85.00861066	77.26783293	-7.740777732	0.00192172	0.007186372
Slc3a2	17	2.707878612	1.324820353	-1.383058259	0.00192157	0.007186372
Plekha2	44	3.593035875	1.736642672	-1.856393203	0.00192735	0.007205577
Elmo3	4	10.04746731	6.893296209	-3.154171104	0.00193334	0.007226123
Ndnf	42	10.59675969	10.9730845	0.37632481	0.00193817	0.007242355
Zbtb46	131	2.521547422	2.048713572	-0.47283385	0.00194176	0.007253899
Lats1	61	1.004805907	1.339788049	0.334982142	0.00194787	0.007274419
Cdk17	92	0.979220036	1.437351505	0.458131469	0.00194824	0.007274419
Trappc1	14	2.373282536	0.934746471	-1.438536064	0.00195173	0.007285583
Pwp2	11	2.927985607	1.03473505	-1.893250557	0.00197912	0.007385954
)10001F23R	23	41.68351125	37.80238542	-3.881125828	0.00198576	0.007408872
Ramp3	1	22.63157895	14.43514644	-8.196432504	0.00200235	0.007468859
Lrrc14	39	1.875979559	1.385238574	-0.490740985	0.00201726	0.007522553
Rarb	15	26.89094773	24.71269581	-2.178251924	0.00202515	0.007550061
Gjb4	6	40.14547361	35.28669056	-4.858783055	0.00202691	0.007554715

Gm9199	8	93.33584934	90.30775943	-3.028089905	0.00202915	0.007561149
430076C15R	47	12.21179029	15.52815095	3.316360659	0.00203821	0.007591034
931409K22R	2	80.95238095	65.11428571	-15.83809524	0.00203805	0.007591034
Gm38404	5	68.62120006	58.92496851	-9.696231553	0.0020437	0.007609566
Paqr5	21	15.49589357	13.39124585	-2.104647716	0.00205467	0.007648464
730043L09R	4	0.751633987	3.277943776	2.526309789	0.00205886	0.007662098
Khdrbs3	122	57.32046706	58.85308312	1.532616059	0.00206134	0.007669405
Gapdhs	4	14.96030213	13.7905054	-1.169796727	0.00206475	0.007680124
Skap1	9	34.52782608	31.33332085	-3.194505233	0.0020713	0.007702566
Mir3087	8	96.50323059	94.41439499	-2.088835606	0.00207446	0.007710453
Fbrs	8	92.8381636	94.85083056	2.012666963	0.00207448	0.007710453
Rgs12	44	0.572330511	1.189050838	0.616720327	0.00209842	0.007797479
Vwa5b2	11	5.105390767	7.339267931	2.233877164	0.00210777	0.007830237
Lep	13	63.8453306	57.89283302	-5.952497575	0.00214223	0.007956227
Ddx42	18	1.736585125	1.124477823	-0.612107302	0.00214473	0.007959482
933406K04R	2	93.9516129	87.16216216	-6.789450741	0.00214439	0.007959482
Prkd2	1	5.166051661	0.977198697	-4.188852963	0.00214445	0.007959482
Ptpro	27	60.65902653	57.99838761	-2.660638919	0.00214553	0.007960447
Gpr26	15	50.81699171	48.45159065	-2.365401059	0.00214625	0.007961084
Aldh5a1	47	1.472379428	1.149906865	-0.322472562	0.00215192	0.007980096
Gng13	23	5.805835857	8.560141187	2.75430533	0.00217673	0.008070051
Ankrd53	2	31.33611955	41.4519685	10.11584895	0.00218263	0.008089894
Trmt10a	42	0.851959057	1.185880074	0.333921017	0.00219018	0.008115833
Pygl	6	11.60632664	9.820542101	-1.785784543	0.00219284	0.008123621
930487H11R	3	93.73781078	96.64666222	2.908851435	0.00219755	0.00813902
Anxa6	8	3.71132655	1.83379541	-1.87753114	0.00221504	0.008201758
Kcnb2	11	89.28039051	85.21779514	-4.062595363	0.00222181	0.008224716
130024F11R	20	2.185221237	1.44461961	-0.740601626	0.00222426	0.008231711
Ppp1r1b	42	7.534115037	9.044548711	1.510433675	0.00223349	0.008263806
Sox6	40	39.20774392	37.78808601	-1.419657904	0.00226124	0.008364381
Pcdhgb5	3	60.33131224	64.84192288	4.510610637	0.00227021	0.00839435
Cox6b2	8	56.75392682	52.61194938	-4.141977443	0.00227049	0.00839435
Fbn2	107	32.33528607	31.91924278	-0.416043298	0.00227188	0.008396668
Smim1	8	11.88662564	8.627020214	-3.259605428	0.00227226	0.008396668

Bid	41	1.991142157	0.86162248	-1.129519677	0.00227808	0.00841604
Col1a2	4	23.90651993	16.64776985	-7.258750086	0.00228384	0.008435202
Col9a2	13	30.33439296	35.57902045	5.244627494	0.00228811	0.008448851
Zfp389	2	6.14391336	1.724137931	-4.419775429	0.00228921	0.008450794
930006I01R	1	52.94117647	26.98412698	-25.95704949	0.00229044	0.008452015
Elovl4	40	10.22815663	13.92923194	3.701075306	0.0022907	0.008452015
Pcdh10	8	38.20482344	43.81200998	5.607186542	0.00229456	0.008464158
Drp2	1	68.85245902	50.66666667	-18.18579235	0.00230144	0.008487376
Fam73a	13	2.040241477	1.26893594	-0.771305537	0.00230531	0.008499509
Lamc3	20	36.48911	39.62723062	3.138120628	0.00231476	0.008532215
Cyp24a1	26	3.824688863	2.994503464	-0.830185399	0.00233708	0.00861234
OC10524586	74	35.14572121	36.30745408	1.161732866	0.0023437	0.008632411
Magix	4	79.83558128	69.83958175	-9.995999534	0.0023432	0.008632411
Wdr20	33	1.723411018	1.097082823	-0.626328195	0.00234771	0.008645003
Tspan13	41	2.786603999	2.00410158	-0.782502419	0.00234919	0.008648275
Ing1	171	8.163995846	7.626625569	-0.537370277	0.00237892	0.00875552
Hsp90aa1	81	0.839944122	1.120314324	0.280370203	0.00238768	0.008785578
Ndufaf3	57	1.568562572	1.140974664	-0.427587909	0.00238875	0.00878728
Capn15	7	99.19335323	97.10789947	-2.085453764	0.00239816	0.008819713
Ogt	3	14.84363082	7.552391668	-7.291239149	0.0024029	0.008834907
Mras	53	1.888391919	1.342055809	-0.54633611	0.00240681	0.008847063
Bhlhb9	14	39.95626002	35.81725191	-4.139008117	0.00240922	0.008853713
Katnb1	11	3.973129983	1.659513448	-2.313616535	0.00241379	0.008868306
Krt74	1	87.94326241	72.54901961	-15.3942428	0.00242821	0.008919019
Htr2c	3	61.07244607	69.76447384	8.692027764	0.00246845	0.009064576
Bsx	4	23.3902148	28.88369383	5.493479031	0.00247012	0.00906843
Efna2	44	2.825315439	1.536719225	-1.288596214	0.00247578	0.009086955
Trim25	12	6.694140111	4.19917807	-2.49496204	0.00248244	0.009109106
Mboat4	5	78.12412081	72.32616217	-5.797958634	0.00249219	0.009142594
700101E01R	33	1.040068062	2.605328587	1.565260526	0.00249707	0.009156747
Lif	44	4.851771351	3.815511173	-1.036260179	0.00249729	0.009156747
Slitrk3	6	46.39303772	51.1628564	4.769818684	0.00250073	0.009167037
Dpysl5	14	13.53643588	17.96761592	4.431180035	0.0025038	0.009173736
Fst	70	1.792699335	1.29614381	-0.496555525	0.00250325	0.009173736

Focad	22	2.239668591	1.463107388	-0.776561203	0.0025053	0.009176942
Kcnip2	43	28.91224052	31.30867028	2.39642976	0.00252293	0.009239183
Cpsf7	55	1.543374605	1.090790753	-0.452583852	0.00252989	0.009262361
Tubb4a	24	28.09152858	25.81329056	-2.278238015	0.00253685	0.009285541
Olfr328	1	96.15384615	85.03401361	-11.11983255	0.00255696	0.009356815
Zbtb4	43	2.109044752	1.603797134	-0.505247618	0.0025671	0.00939157
Pla1a	3	97.40544053	94.66996555	-2.735474979	0.00257925	0.009433685
Tbc1d16	36	2.558144413	1.768721158	-0.789423255	0.00259226	0.009478893
Moxd1	42	10.27569727	10.00046885	-0.275228419	0.00259707	0.009494117
Ak5	76	3.146437869	2.472828866	-0.673609002	0.00260701	0.009528098
Gm14858	4	37.1036036	48.69791667	11.59431306	0.00262422	0.009588614
Airn	7	53.64940825	47.75134728	-5.89806097	0.0026251	0.009589408
Cd37	1	36.97916667	23.15270936	-13.82645731	0.00263639	0.009628286
Pgf	24	3.223783176	2.103437929	-1.120345247	0.00264523	0.009658159
Apoa4	4	96.14645981	93.08749415	-3.058965657	0.00267687	0.009771253
Grin2b	11	62.82765198	57.34171992	-5.485932059	0.00270174	0.009859573
Tnfsf13	4	15.14981525	9.529204815	-5.620610431	0.00270592	0.009872361
Bcl6b	14	2.743126471	3.075388121	0.33226165	0.00271129	0.009889498
Stox1	35	1.505723905	2.972860761	1.467136856	0.00271579	0.009903469
Klhdc8b	5	30.33084711	24.71857354	-5.612273573	0.00271674	0.009904466
330011K09F	5	30.64173044	24.3009996	-6.340730843	0.00274814	0.010016437
Usp53	61	1.081629248	1.313283931	0.231654683	0.00276987	0.010093122
Ubl7	22	1.759945545	1.210960216	-0.548985329	0.00277725	0.010117529
Ccdc166	28	8.080009394	7.193255198	-0.886754196	0.00278373	0.0101386
930539J05R	32	2.270417213	1.478287805	-0.792129408	0.00278943	0.010156864
Scn3b	5	9.421756922	13.47098516	4.049228233	0.0027926	0.010165852
Cd3e	2	80.55555556	66.19670747	-14.35884808	0.00279401	0.010168466
Gtf2a1l	1	100	92.39130435	-7.608695652	0.00279855	0.010182475
Itpr1	31	2.331488736	2.72353806	0.392049324	0.00281029	0.010222657
Rbm10	22	19.40995018	16.44153639	-2.968413787	0.00281521	0.01023803
Ubox5	3	16.86646155	11.24542125	-5.621040305	0.00281959	0.010251387
Lyzl4os	4	96.72131148	89.44529392	-7.276017558	0.00285378	0.010373153
Nkap	28	21.06753983	18.02010661	-3.047433223	0.00287194	0.010436555
Nucb1	19	3.227650459	2.049898427	-1.177752032	0.00290777	0.010564138

Pptc7	44	1.235755656	0.873762567	-0.361993089	0.00291732	0.010596209
Zmym6	36	1.516672108	0.836162346	-0.680509763	0.00292397	0.010617752
Mapk15	2	93.40848932	84.30650132	-9.101988	0.0029471	0.010699096
Hephl1	1	84.34343434	73.04964539	-11.29378895	0.00295338	0.010719259
Crybb1	4	86.04735883	77.23141498	-8.815943851	0.00296726	0.01076694
St3gal5	44	1.262850572	1.56214199	0.299291417	0.00297319	0.010785798
B4galt5	106	1.625749905	1.177822682	-0.447927223	0.002975	0.010789719
Arhgef9	7	19.09054604	15.12667378	-3.963872261	0.00297909	0.010801859
Ccdc116	54	46.54791078	44.9321781	-1.615732678	0.002982	0.010807071
Zfp768	78	2.024362711	1.57181982	-0.452542891	0.00298179	0.010807071
Slc25a31	45	92.64453017	90.91496171	-1.729568461	0.00298337	0.010809382
Igsf5	8	40.27149533	35.37794843	-4.893546902	0.00300759	0.010894425
Fosb	41	1.876193703	1.417973471	-0.458220232	0.00301093	0.010903842
Platr20	4	89.72990191	84.33164007	-5.398261841	0.00302609	0.010956049
Mir711	12	84.31498152	80.54893148	-3.766050042	0.00304324	0.011015411
Tbc1d1	139	2.970239206	2.237773443	-0.732465763	0.00305228	0.011045419
Bnc2	140	46.35405127	45.60984373	-0.74420754	0.00305565	0.011054876
Mir129b	4	8.06704576	3.959979514	-4.107066245	0.00307611	0.011126156
Prr15l	3	41.26194607	33.10266585	-8.159280223	0.00309259	0.011183021
Actbl2	2	92.76039403	84.63276836	-8.12762567	0.00309749	0.011195749
Serping1	4	65.55389647	59.75101765	-5.802878824	0.00309764	0.011195749
Jam2	15	2.683990454	3.79699699	1.113006535	0.00309865	0.011196653
Cep112	39	2.048303111	1.490697032	-0.557606078	0.00310004	0.011198918
530026P05R	2	32.18390805	20.52023121	-11.66367683	0.00312114	0.011272367
Prkar1a	90	1.655250552	1.103592797	-0.551657754	0.00312339	0.011277729
Psen2	51	8.78238161	7.493923606	-1.288458004	0.00312594	0.011284155
Pdgfc	37	1.856821117	1.110546844	-0.746274272	0.00313569	0.011316546
BC023829	5	26.7585353	18.9053552	-7.853180102	0.00313822	0.011322914
Dip2c	69	1.496962873	1.083157108	-0.413805765	0.00313931	0.011324064
Sln	1	67.85714286	40.35087719	-27.50626566	0.00314456	0.011340191
Aifm3	36	1.497770286	2.730752542	1.232982256	0.00315342	0.01136934
Atp6v1e2	3	88.26463886	80.13994423	-8.124694629	0.00316227	0.011396164
Sec14l1	93	1.837633424	1.326651168	-0.510982255	0.00316241	0.011396164
Ntf5	27	4.5691798	5.83438696	1.26520716	0.00318711	0.011482363

Mir6931	3	97.43589744	91.71356758	-5.72232986	0.00319192	0.011496863
Mir6925	4	95.44174954	91.10055942	-4.341190124	0.00319717	0.011512944
Mir5108	1	83.78378378	64	-19.78378378	0.00319951	0.011518536
Ddx21	19	2.075881781	1.180885999	-0.894995782	0.0032104	0.011554906
Fam162b	5	45.38705739	36.46423418	-8.922823209	0.00321452	0.011566907
Ptprn	14	4.425692877	2.926267989	-1.499424888	0.00321958	0.011582296
Nip7	50	1.939051303	1.405519843	-0.533531461	0.00322057	0.011582998
Sox21	75	20.74634566	21.84455574	1.098210081	0.00323706	0.011639468
Gnaq	158	1.112713939	1.163071872	0.050357934	0.00323931	0.011644694
Slc23a1	3	73.15078213	85.7990479	12.64826577	0.00324021	0.011645072
Trip4	22	1.661897707	1.224607531	-0.437290176	0.00325372	0.011690761
Unkl	92	1.846889908	1.469918724	-0.376971183	0.00325692	0.011699407
Rnf152	52	1.730982342	1.362250569	-0.368731773	0.00326297	0.011718254
Mastl	33	1.585160074	1.147118789	-0.438041286	0.00326621	0.011727031
Impdh1	93	2.692621158	2.19516903	-0.497452128	0.00327048	0.011739471
Rpf2	23	1.654468526	1.197711707	-0.456756819	0.00328096	0.011774222
Slc7a7	12	59.92814323	56.84479422	-3.083349016	0.00329232	0.011812106
Rab3il1	37	3.118538389	2.479061325	-0.639477064	0.00330613	0.011857557
Rbm11	7	4.261227879	2.840323222	-1.420904656	0.00330661	0.011857557
Zfp148	42	1.826777348	1.302548503	-0.524228845	0.00331102	0.011870471
Pbp2	20	97.44829939	95.64156919	-1.806730192	0.0033332	0.011947094
Atp6v1g1	29	2.047032049	1.617953256	-0.429078792	0.00334449	0.011984616
Ecscr	3	8.035714286	3.06122449	-4.974489796	0.00336015	0.012037799
Ctf1	6	27.97438984	22.48043166	-5.493958187	0.00336672	0.012058411
Cxcl16	8	2.956002734	2.279509058	-0.676493676	0.00338096	0.012106443
Ddx4	50	95.37002847	93.85987376	-1.51015471	0.00338182	0.012106554
Eif1b	109	2.115334284	1.535465671	-0.579868613	0.0033902	0.012133625
510009L18R	75	1.034471894	1.278944023	0.244472129	0.00339165	0.012135854
Ppp1cc	24	0.873828512	1.485775787	0.611947275	0.00339454	0.012143223
Ihh	77	6.406933621	6.379300344	-0.027633277	0.00339812	0.012153058
Cxadr	46	2.020973299	1.393179023	-0.627794276	0.00340931	0.012190122
700010I14R	3	24.26668308	16.29790854	-7.968774539	0.00345064	0.012333987
Dusp5	106	0.991505969	1.259520282	0.268014312	0.00345123	0.012333987
Hltf	20	1.81218293	0.805434202	-1.006748728	0.00345331	0.012338424

Hoxc5	29	35.85861723	38.45493591	2.596318682	0.00346152	0.012364729
Cpxm1	24	35.71777912	33.5277119	-2.190067217	0.00349382	0.012477068
Cdx2	31	47.81182345	51.00838903	3.196565582	0.00351077	0.012534561
Sox11	101	28.64732689	29.97085592	1.323529028	0.00351375	0.012542142
Kcnj14	2	85.90686275	77.56924321	-8.337619535	0.00351682	0.012550054
Wif1	30	3.27501371	2.208792176	-1.066221534	0.00355423	0.012680452
Vat1l	13	36.70312759	34.22299196	-2.480135625	0.00360784	0.012868594
Dnaja3	37	2.03755566	1.302514226	-0.735041434	0.00362116	0.012912994
Car7	56	8.763789523	10.29561964	1.531830117	0.00362789	0.012933831
Ext2	38	2.077527079	1.338152954	-0.739374125	0.00363466	0.012952244
Slc41a2	48	2.258262357	1.464295492	-0.793966866	0.00363482	0.012952244
430078K24F	1	58.75	43.93305439	-14.81694561	0.00364977	0.013002368
Csn3	1	24.08376963	12.63157895	-11.45219069	0.00365179	0.013006398
Thumpd3	10	1.614035854	1.114147329	-0.499888525	0.0036592	0.013029635
Dnm3	21	4.31995013	5.67268592	1.35273579	0.00366924	0.01306222
Cdk1	30	2.306863373	1.763045101	-0.543818272	0.00368553	0.013117026
Klb	8	53.10335453	58.57374569	5.470391159	0.00368671	0.013118023
Gabra4	8	41.825276	36.88683863	-4.938437375	0.00369932	0.013159731
Chrdl2	22	7.267344947	5.464825731	-1.802519216	0.00371256	0.013203617
Wwc2	97	1.84701221	1.386838508	-0.460173701	0.00371796	0.01321963
'00121N20F	4	86.71278934	80.27305257	-6.439736772	0.0037218	0.013230074
Rap1gap2	65	19.70838016	21.93084284	2.222462684	0.00374003	0.013291644
Grik4	69	4.105247791	5.722193083	1.616945292	0.00374746	0.013314822
Lrrc31	1	68.1372549	53.80434783	-14.33290708	0.00375646	0.013343582
Krtap5-4	3	89.14876578	92.98844949	3.839683708	0.00375908	0.013349634
Gm38671	1	31.53846154	17.98245614	-13.5560054	0.00376144	0.013354785
Cebpd	83	1.774562522	1.203395685	-0.571166837	0.00377092	0.01338522
Eci1	46	4.922396233	4.220079389	-0.702316844	0.00378773	0.013441646
BC100451	5	91.06660262	87.60474325	-3.461859373	0.00379295	0.013456886
Zfp703	78	1.799588335	1.287086447	-0.512501888	0.0038037	0.013491769
Hist2h2aa2	11	2.456051978	1.78603253	-0.670019448	0.00380897	0.013507222
Ubl4a	47	18.28254921	16.83817906	-1.444370149	0.00385063	0.013651631
Etnk2	48	2.797175502	2.163149506	-0.634025996	0.00386427	0.0136967
Mfi2	6	16.07920101	20.6194478	4.540246788	0.00386602	0.013699581

Gm11651	1	48.96551724	32.43243243	-16.53308481	0.00387303	0.013721104
Taf1	9	17.08647396	14.26045791	-2.826016055	0.00391914	0.013881131
Mrpl42	17	12.95308243	10.95493156	-1.998150872	0.00392751	0.013905879
Gcat	15	0.870871192	1.799855375	0.928984183	0.00392803	0.013905879
Diexf	46	1.461124829	0.870706411	-0.590418418	0.00392934	0.013907157
Pax6os1	5	47.48486138	54.75836807	7.273506692	0.00393856	0.013934148
;10301B20F	53	1.209671348	0.893101368	-0.31656998	0.00393886	0.013934148
'32490B19F	1	15.59139785	27.63819095	12.04679311	0.00395599	0.013989424
Dkkl1	5	52.94531104	47.08375615	-5.861554883	0.00395639	0.013989424
Dhx29	44	1.26937987	1.755783721	0.486403851	0.00398257	0.014078604
Rbfa	31	7.572553899	6.82149837	-0.751055529	0.00398911	0.014098298
Msi2	36	2.460325471	1.702883823	-0.757441649	0.00399924	0.01413073
Dock2	5	59.49070263	65.68982213	6.199119501	0.00400775	0.014157375
Mttp	15	0.790043452	1.597265526	0.807222074	0.00404633	0.014290218
Foxa1	67	1.415645892	1.158127743	-0.257518148	0.00405302	0.014310403
AI317395	21	9.965704217	9.677403992	-0.288300225	0.00406481	0.014348562
Tmem132e	53	47.57493806	46.2548901	-1.320047962	0.00408488	0.014415965
Rilp	15	10.60393033	9.224971782	-1.378958553	0.00410039	0.014467218
Plcxd1	2	52.32990896	44.16216216	-8.167746795	0.00411593	0.014518538
Gltpd2	5	91.40734543	86.85051456	-4.55683087	0.00413077	0.014567402
Aldoa	60	3.198872165	2.399227606	-0.799644559	0.0041368	0.014583303
Mir7660	8	28.5356767	27.13945399	-1.396222706	0.00413727	0.014583303
Tpra1	39	0.802183199	1.095242212	0.293059013	0.00416722	0.014685363
Pcsk6	104	1.478204664	1.018212163	-0.459992501	0.00417938	0.014724649
Timm8a1	39	19.49016496	17.48013537	-2.010029593	0.00418705	0.014748136
Pde1b	29	9.183809809	12.9004193	3.716609488	0.00420411	0.014804676
Ralgds	148	2.926728024	2.657306588	-0.269421437	0.00420713	0.014811771
Pax7	26	19.01453463	18.4824295	-0.532105137	0.00423001	0.014888726
Magea2	6	75.28618372	69.05166606	-6.234517666	0.00423987	0.014919878
L3mbtl1	12	4.801046644	3.792666281	-1.008380363	0.00424112	0.014920694
Lad1	49	3.000975102	1.885054602	-1.1159205	0.00424313	0.014924172
Atp5sl	6	2.779309922	1.303744957	-1.475564965	0.00428042	0.015051723
Mir686	3	1.824817518	2.872062663	1.047245145	0.0042963	0.01510394
Sin3b	22	1.636787931	0.886239598	-0.750548334	0.00430193	0.015120139

Snhg11	5	19.96464739	17.38244328	-2.582204103	0.00430716	0.015134887
Fasl	1	83.74384236	92.62672811	8.882885746	0.00430864	0.015136447
Miip	19	2.046507371	2.60163174	0.555124369	0.00431037	0.015138908
Brd8	3	0	1.320261438	1.320261438	0.00431761	0.015159211
Rab36	11	5.266762144	2.648061725	-2.618700419	0.00431822	0.015159211
Mir292b	3	89.19154229	83.10049969	-6.091042594	0.00432787	0.015182197
Abca3	68	1.781819571	1.256169262	-0.52565031	0.00432759	0.015182197
Mir291b	3	89.19154229	83.10049969	-6.091042594	0.00432787	0.015182197
Cdca3	16	3.511146648	2.197031378	-1.31411527	0.00432892	0.015182261
Rrnad1	9	0.911301885	2.444140904	1.532839019	0.0043401	0.015217829
932443I19R	3	68.28028457	62.46176827	-5.818516298	0.00434243	0.015222378
Tmem178b	5	61.58962922	56.58628313	-5.003346089	0.00434421	0.015224951
Zfp958	9	2.478013531	0.895036143	-1.582977388	0.0043456	0.015226186
Wnt3	60	15.53117633	17.5078004	1.976624064	0.00437183	0.015314435
Tatdn1	12	2.040813689	1.51067163	-0.530142059	0.00437622	0.01532618
Pltp	7	6.750164333	2.527780312	-4.222384021	0.00439071	0.015373247
Arl5a	30	1.596888525	0.868719844	-0.72816868	0.00439999	0.015402077
Ogfod1	14	2.411191566	1.470401007	-0.940790559	0.00442063	0.015470626
Crim1	71	1.207413782	1.424078277	0.216664496	0.00442603	0.015485817
Trim44	136	1.92733592	2.434983339	0.507647419	0.00443437	0.015511327
Pdlim2	17	7.264037065	6.668493454	-0.595543611	0.00445128	0.015566756
Gpbar1	12	72.19729344	67.5043366	-4.692956839	0.00445754	0.015584921
Islr	7	78.84280296	72.98911688	-5.853686073	0.00447384	0.015638199
Brwd3	4	25.97370706	17.0262571	-8.947449961	0.00450172	0.015731896
Tmem44	14	1.463513658	0.514636853	-0.948876805	0.004507	0.015746609
Tlx1	45	17.34944814	18.13252272	0.783074573	0.00452527	0.015806653
Ocrl	7	10.60493741	8.135641456	-2.469295952	0.00452718	0.015809583
Creb3l1	31	5.623317784	7.326041661	1.702723878	0.00455168	0.015891369
Map2	20	3.706847623	2.678984804	-1.027862819	0.00455376	0.015894815
Gjc1	149	1.570276024	1.346708051	-0.223567973	0.00456526	0.015931191
Zmiz1	84	1.863334464	1.267839478	-0.595494986	0.00457278	0.01595362
Vim	24	1.55091838	0.771909531	-0.779008848	0.00458922	0.01600719
Tcp11	30	8.146302222	7.579561715	-0.566740507	0.00459151	0.016011375
Trim66	5	50.89066464	45.89615602	-4.994508618	0.00460865	0.016067299

Ptdss2	48	2.36552688	1.843758822	-0.521768058	0.00462374	0.016116085
Shcbp1l	24	54.642938	57.60074005	2.95780205	0.0046358	0.01615428
Arhgap31	30	2.172463669	1.485633354	-0.686830315	0.00465292	0.016210089
Mt1	21	2.937009468	2.099638104	-0.837371365	0.00466352	0.016243191
Il10rb	23	2.057014898	1.19091052	-0.866104377	0.00468865	0.016326843
Rhoj	16	11.6352621	16.74326147	5.107999368	0.0047039	0.016376053
App	50	1.389951845	1.013272145	-0.3766797	0.00470623	0.016380286
532454L22F	4	51.81835534	43.15807351	-8.660281821	0.00471311	0.016396432
Foxred2	37	3.340696667	5.123136108	1.782439441	0.00471239	0.016396432
Peli2	81	1.126344862	0.891807702	-0.23453716	0.00471552	0.016400925
Kcne3	11	10.39001117	15.68634196	5.296330783	0.00472012	0.016413056
Tesc	46	12.81925527	14.3783962	1.559140929	0.00472654	0.016431481
Klf6	23	2.603919292	1.303151128	-1.300768163	0.00473052	0.016441429
Neurog3	11	11.38275337	15.06114439	3.678391017	0.00474375	0.016483495
Sh3rf2	11	62.0753641	64.8635723	2.788208201	0.00474516	0.016484498
Bod1l	55	0.83151206	1.564479948	0.732967888	0.00474774	0.016489541
Tle4	170	1.496899879	1.085865213	-0.411034666	0.00475598	0.016514243
Atp10d	10	1.794289515	0.550408233	-1.243881281	0.00480139	0.016667997
Ccdc109b	9	2.021266429	1.271279185	-0.749987244	0.00480766	0.016685813
Mir6999	12	94.19388337	91.61422245	-2.579660924	0.00481658	0.016712813
'00049G17F	12	4.297941845	3.005610611	-1.292331234	0.00483161	0.016760991
Mroh6	4	90.60992496	95.82876154	5.218836578	0.00484264	0.016795307
Ephx4	42	8.80051232	11.16514547	2.364633147	0.00484864	0.016812136
'00094J05R	6	94.71918392	91.67274077	-3.046443148	0.00486065	0.016849779
Zbtb34	61	1.673848807	1.403318207	-0.2705306	0.00486428	0.016858396
Ccdc181	16	1.602853182	0.733004534	-0.869848648	0.00486808	0.016867576
Add2	3	26.26882868	34.3995075	8.130678813	0.0048735	0.016882384
Cachd1	78	1.579945219	1.296896643	-0.283048577	0.00487932	0.01689855
Uck2	93	1.129256193	1.339950008	0.210693816	0.00489636	0.016953543
Lmtk3	31	2.539179563	1.972920293	-0.566259271	0.0049133	0.017008209
Iah1	24	1.144302998	1.626234978	0.48193198	0.00492001	0.017027416
Rpl10a	78	2.117551403	1.745684963	-0.371866439	0.00492379	0.017032445
BC016579	10	8.067037879	11.78128948	3.714251599	0.0049237	0.017032445
Ccdc96	92	2.333742558	1.726908893	-0.606833665	0.00493161	0.01705548

Rap1gds1	132	1.07501263	1.149710798	0.074698168	0.00493776	0.017072729
Dus2	29	1.208010541	0.548065682	-0.659944858	0.0049423	0.017084412
Usp13	66	39.59658737	41.02948714	1.432899767	0.00495717	0.017126123
Xrcc6bp1	29	0.90162533	1.414365803	0.512740473	0.00495623	0.017126123
Apoc2	6	40.64651561	35.20560454	-5.440911075	0.00495787	0.017126123
Kat6b	85	2.735628046	1.950573389	-0.785054657	0.00496569	0.017149114
Arhgap27	11	14.16330769	12.30344582	-1.859861867	0.00496859	0.017153375
Mir7b	12	3.648310969	1.814477831	-1.833833137	0.00496927	0.017153375
Snrnp200	21	1.349334104	0.805772284	-0.54356182	0.00497763	0.017178205
Pigh	14	0.629873648	2.43636304	1.806489392	0.00499614	0.017238029
Emc3	9	2.539078007	0.851567977	-1.687510031	0.00502027	0.01731721
Upk1b	3	32.62311762	25.93627339	-6.686844235	0.00507391	0.017498144
'00052K11F	73	1.384272076	1.052618221	-0.331653854	0.00507683	0.017504079
Nos3	15	83.36182865	84.52874181	1.166913155	0.00508128	0.017515315
;10009E07F	57	4.435763055	4.244306511	-0.191456543	0.00509009	0.017541552
Wnt7a	28	2.403582493	1.792195844	-0.611386649	0.0051043	0.01758642
Frk	2	14.72277722	9.261790841	-5.460986382	0.00510935	0.017599657
Olfr750	3	77.63402216	69.90551996	-7.728502199	0.00511444	0.01761306
Tpmt	14	3.294196921	1.487582787	-1.806614134	0.00511868	0.017623528
Golph3	68	0.784679579	1.225950828	0.441271249	0.00512642	0.017646043
Pnmal2	22	27.64584776	25.05349704	-2.592350721	0.00513891	0.017684889
Mybpc2	1	58.66666667	34.92063492	-23.74603175	0.00515527	0.017737031
Mrps6	53	1.821306194	1.238385925	-0.582920269	0.00519714	0.017876908
Sppl2a	3	4.337218212	1.573254671	-2.763963541	0.00520807	0.017910286
Purg	86	3.243522828	2.543417712	-0.700105115	0.00521779	0.017939512
Mapk9	58	1.758635314	1.189944031	-0.568691283	0.00522625	0.017960188
Nipal3	15	2.926965363	1.447520961	-1.479444403	0.00522591	0.017960188
Rhpn2	5	3.323449825	3.965597937	0.642148112	0.0052331	0.017979508
'00001L05F	9	16.08206894	20.56801799	4.48594905	0.0052526	0.018042278
Rrs1	35	25.29171052	24.40513157	-0.886578947	0.00527513	0.018115441
Naprt	8	11.62406791	10.36073059	-1.263337315	0.00529501	0.018179436
Dhx58	3	95.16749241	88.96884803	-6.198644374	0.00530765	0.018218598
Dgkd	115	1.204090508	1.342595937	0.138505428	0.0053117	0.018228232
Iqcc	17	1.541289615	0.860722507	-0.680567108	0.0053137	0.018230818

Gm4262	3	19.88295747	11.95265168	-7.93030578	0.00531809	0.018241608
Emilin2	63	6.527125095	7.83455674	1.307431645	0.00535288	0.018356665
Bclaf1	42	1.638480831	1.333273948	-0.305206883	0.00535941	0.018374771
Gnat2	6	86.68456405	81.25610175	-5.428462298	0.0053633	0.018383803
Nufip1	22	0.930932897	1.375985748	0.44505285	0.00539325	0.018482147
Rnase13	4	95.06380686	89.65056734	-5.413239516	0.00541425	0.018549774
Myt1l	12	36.51635812	34.62032006	-1.896038058	0.00543642	0.01862139
Zkscan1	26	1.268845529	0.922225607	-0.346619922	0.00543805	0.018622641
Rnf220	29	18.64190645	20.58774726	1.945840806	0.00545109	0.018662928
Arl6ip1	16	1.803925802	0.86157776	-0.942348042	0.00546741	0.018714449
Aldh7a1	95	4.660676195	5.772647347	1.111971152	0.00547549	0.018737711
Rprd2	21	1.479698814	0.705695946	-0.774002868	0.00549076	0.018785587
Nfatc1	184	1.501143863	1.329573199	-0.171570663	0.00549811	0.018806343
Ndufa3	14	3.349582533	2.103464503	-1.246118031	0.00550331	0.018819755
Pdia5	69	1.793607512	1.692404329	-0.101203183	0.00550478	0.018820383
Actr1a	7	2.780068557	1.454225398	-1.325843159	0.00551405	0.018847706
Mir5046	48	93.9462957	94.54909814	0.602802442	0.005532	0.018895849
Gm5424	22	6.718817959	8.241673014	1.522855055	0.00553089	0.018895849
Sqrdl	13	1.534867709	2.41090035	0.876032641	0.00552994	0.018895849
Tmsb15b1	4	37.53921482	47.69664955	10.15743473	0.00553737	0.018905393
Tmsb15l	4	37.53921482	47.69664955	10.15743473	0.00553737	0.018905393
Itfg2	11	1.770703421	1.014308709	-0.756394711	0.00554118	0.018913997
Cdc25a	36	1.42413515	0.907568324	-0.516566826	0.0055569	0.018963261
Vcl	65	1.435397967	0.89562029	-0.539777677	0.00555942	0.01896745
Cbln3	19	1.134403957	1.682954631	0.548550674	0.00558708	0.019057386
Ube2d2b	7	98.54568977	96.3737954	-2.171894365	0.00559729	0.019087746
Large	101	1.423183951	1.25831569	-0.164868261	0.00560902	0.019120729
Tmc6	21	4.062503793	3.145963127	-0.916540666	0.00560956	0.019120729
S1pr2	14	3.644099502	2.591994223	-1.05210528	0.0056139	0.019131053
Chrd	85	2.741990078	2.234609854	-0.507380224	0.00561899	0.019143981
Tcte2	103	1.887925948	1.682046486	-0.205879463	0.00562254	0.019151622
Med23	40	1.686442643	1.167869605	-0.518573038	0.00562669	0.019155247
Pno1	23	2.866031824	2.498767541	-0.367264282	0.00562752	0.019155247
'30013B05F	19	10.28400893	8.919105265	-1.364903667	0.00562732	0.019155247

330007P06F	7	18.49830889	14.86565665	-3.632652241	0.00565444	0.01924239
Rundc3a	37	5.356964694	5.53175434	0.174789647	0.00567776	0.019314508
Unc45b	2	77.61561562	84.29637891	6.680763293	0.00567826	0.019314508
Npy	25	17.58174433	17.60550966	0.023765324	0.00568146	0.019320905
Ppib	54	1.120916263	0.81880341	-0.302112854	0.00570914	0.019410558
Clns1a	61	7.837974467	7.06593752	-0.772036947	0.00573496	0.019493801
Sars	12	1.380615655	0.355792931	-1.024822723	0.0057372	0.019496921
H2afz	95	1.265256806	1.468355523	0.203098717	0.005765	0.019586859
Erbb3	57	1.90556443	1.557289465	-0.348274965	0.00577714	0.019623541
Sctr	8	18.5205511	16.67867506	-1.841876041	0.00578741	0.019653888
Dcaf7	63	1.565141999	1.286067235	-0.279074765	0.00579993	0.019691847
Spag7	32	1.823779542	1.13443291	-0.689346632	0.00580954	0.019719901
Gramd3	13	2.672846965	1.736462925	-0.93638404	0.00581572	0.019736323
Ssmem1	4	91.36904762	82.02232606	-9.346721555	0.00582778	0.019772683
Scamp1	77	1.553318628	1.171955894	-0.381362734	0.00583549	0.019794258
Mob4	69	1.191928771	1.51988043	0.327951659	0.00585858	0.019868003
Mfap3	19	2.033680007	1.373905813	-0.659774194	0.00586926	0.019899617
Paqr9	134	5.362162639	6.319929234	0.957766594	0.00591722	0.020057593
930407G08F	4	67.83898305	61.56820631	-6.270776746	0.00592553	0.020081103
Ptpn23	62	1.396791056	1.029250939	-0.367540116	0.00593805	0.020118881
Ifnlr1	18	2.936435846	1.451654911	-1.484780936	0.00594068	0.020122913
Zfp867	10	2.12272933	1.075981801	-1.046747529	0.00594198	0.020122913
Vash1	35	1.956689724	1.224592245	-0.732097479	0.00596107	0.020182923
Ppp2ca	88	1.41679247	1.077695983	-0.339096487	0.00596454	0.020189987
Scn4b	24	5.993234878	5.003566994	-0.989667884	0.00596977	0.020203048
Myl1	2	85.91549296	74.3902439	-11.52524906	0.00598578	0.020252543
Ptar1	100	0.870389463	1.04254829	0.172158827	0.00603531	0.020415446
Rnf182	38	9.849623713	9.673407108	-0.176216605	0.00603928	0.020424156
Tppp	39	2.163148973	1.201131969	-0.962017004	0.00606949	0.020521606
Fah	46	3.705325823	2.855265548	-0.850060276	0.0061063	0.020641299
G6b	5	23.07982349	30.26053041	7.180706916	0.00612616	0.02070365
Fsip1	4	4.647045505	2.374223646	-2.272821859	0.00619043	0.020913339
Ttc17	53	1.287497112	1.7388012	0.451304088	0.00619106	0.020913339
Pin1rt1	5	97.88375835	96.39092304	-1.492835311	0.0062041	0.020952569

Hmga1-rs1	55	1.677941963	1.482825654	-0.195116309	0.00625341	0.021114249
Slc25a17	7	0.66626089	2.184375579	1.518114688	0.00626887	0.021156718
Dcc	13	45.98263974	50.52080196	4.53816222	0.00626787	0.021156718
Trub1	9	3.079141507	1.732016636	-1.347124872	0.00629281	0.021232639
Zfp606	39	1.533238217	1.096790862	-0.436447355	0.00633559	0.021372051
Txnl4a	27	2.076088389	1.067394858	-1.008693531	0.0063508	0.021418447
Flnc	37	4.173303671	2.90921843	-1.264085241	0.00637816	0.021505765
333419F23R	36	2.517131951	2.221163936	-0.295968015	0.00638154	0.021507278
Map3k8	36	2.517131951	2.221163936	-0.295968015	0.00638154	0.021507278
Wdr48	85	1.16997283	1.302395132	0.132422303	0.00639988	0.021564126
Mirlet7e	13	85.35604991	82.22245121	-3.133598695	0.00641887	0.02162315
Fsbp	3	92.41715879	96.15050919	3.733350395	0.00642438	0.02163676
Mir215	3	96.82125544	93.57227446	-3.248980981	0.00643355	0.021662656
Tmem106c	54	1.426976724	1.079061122	-0.347915602	0.00650044	0.021882863
Tie1	1	74.07407407	62.40310078	-11.6709733	0.00652578	0.021963154
Nprl2	14	1.874618507	2.485355206	0.610736698	0.00652935	0.021970129
Enpep	1	51.78571429	28.16901408	-23.6167002	0.00654973	0.022033625
11-Sep	80	1.112003032	1.345873957	0.233870926	0.00656927	0.022094324
Xlr3c	4	71.93144499	66.37043447	-5.561010518	0.00659472	0.022174818
Fn3krp	11	2.531761016	1.837513197	-0.694247819	0.00660493	0.02220406
Ppm1f	5	4.473267433	2.746046866	-1.727220567	0.00663634	0.022304562
Zfp648	2	52.49982346	59.59949291	7.099669451	0.00665826	0.022373085
Tnip2	31	0.974170273	1.660171985	0.686001712	0.0066627	0.022382901
Flt4	26	2.104691458	1.208604022	-0.896087436	0.00669662	0.02249168
Hrsp12	12	1.942539079	0.795511663	-1.147027416	0.00672574	0.022576306
Mir7235	14	82.92965973	79.06914463	-3.860515104	0.00672643	0.022576306
C1qtnf2	15	11.20077845	11.81209723	0.611318786	0.0067256	0.022576306
Mir7118	7	97.94983626	96.58238165	-1.367454608	0.00674471	0.022632504
Greb1	18	12.89621338	9.565506303	-3.330707073	0.00675392	0.02265821
Sptb	54	2.989090256	4.224632598	1.235542342	0.00675966	0.022672287
Hoxd3	26	48.50166371	50.95673404	2.455070334	0.00676236	0.022676147
Csrnp2	64	1.775002311	1.419740332	-0.355261979	0.00680811	0.02282434
Galnt15	2	74.19738863	63.82264475	-10.37474388	0.00681916	0.022856172
Mir365-1	1	98.97260274	95.37366548	-3.598937259	0.00682303	0.022863939

Pak2	42	1.005445412	1.166291982	0.16084657	0.00685371	0.022961495
Mms22l	66	1.349158719	1.007781044	-0.341377675	0.00686362	0.02298945
Shtn1	32	2.655086461	1.664274368	-0.990812093	0.00687052	0.023007311
Wnt1	32	6.899636635	8.354906956	1.455270321	0.00688449	0.023048835
Prox1	52	40.95259904	40.36069502	-0.591904017	0.00688824	0.023056136
Mrpl9	22	2.866057904	2.056444862	-0.809613042	0.00690242	0.023098318
S1pr4	1	44.44444444	33.42036554	-11.02407891	0.0069049	0.023101348
Nfib	109	1.787231113	1.582368829	-0.204862285	0.00692301	0.023156667
Cdnf	27	2.980167393	1.949272277	-1.030895116	0.00694146	0.023213067
Ptchd3	19	65.06393779	61.86236924	-3.201568557	0.00694526	0.023220477
Hspa4l	51	1.558050225	1.001481878	-0.556568347	0.00698536	0.023341222
Thsd7b	33	50.27027044	52.83598816	2.565717719	0.0069869	0.023341222
AI661453	33	1.93420288	1.466137766	-0.468065114	0.00698773	0.023341222
Fhad1os1	1	54.62962963	36.75213675	-17.87749288	0.00698438	0.023341222
Rspo1	66	13.71581497	14.11526755	0.399452576	0.00700333	0.023388007
Psd	9	8.707502593	8.623250094	-0.084252499	0.00700744	0.023396407
Mir6950	2	28.54302066	19.3267674	-9.216253257	0.00702831	0.023460737
Cers4	6	20.06828849	28.01106283	7.942774341	0.00703322	0.023468559
Nfil3	110	1.321059236	1.237003903	-0.084055334	0.00703385	0.023468559
Lcat	7	89.36503794	92.15546002	2.790422081	0.00705037	0.023518345
Iffo2	83	2.164107959	1.38388355	-0.780224409	0.00706731	0.023569462
Coro6	9	91.92177617	88.33108861	-3.590687557	0.00707171	0.023578794
Irs1	45	2.23812724	1.634336724	-0.603790516	0.00707903	0.023597823
Mir1928	4	78.69520196	71.33048884	-7.364713124	0.00711517	0.023712938
Rab3ip	90	1.296006964	1.894188571	0.598181607	0.00715146	0.023828467
)00026A02F	2	92.43481325	84.94468085	-7.490132398	0.00715327	0.023829092
Grin2d	44	4.067350998	2.391913798	-1.6754372	0.0071903	0.023947009
Uchl1os	7	10.00207381	7.544093491	-2.457980321	0.00720203	0.023980632
Flad1	12	3.515263306	1.885069714	-1.630193592	0.00721416	0.024015571
)31428F04F	16	10.44373495	10.40928392	-0.034451033	0.00722092	0.024032595
Prrc2c	33	1.523447867	0.745113161	-0.778334706	0.00728022	0.024224464
Aida	3	18.05205844	13.47021646	-4.58184198	0.00728309	0.024228522
Rbm27	28	1.066283082	1.762008909	0.695725827	0.00728965	0.024244869
Elf4	10	22.67306664	19.08013059	-3.592936057	0.00732006	0.024340487

Mill2	3	4.633157697	2.38730239	-2.245855307	0.0073456	0.024417348
Ywhaz	8	31.73173367	29.7803582	-1.951375474	0.0073465	0.024417348
Mtg1	44	1.801212655	1.112054864	-0.689157792	0.00734908	0.024420393
Rbms1	135	1.568968896	1.311287009	-0.257681888	0.00737423	0.024498415
Rasgrp1	16	14.50644587	17.56809665	3.06165078	0.00737715	0.02450255
Rpl37rt	10	98.96454655	98.11474209	-0.849804459	0.00739009	0.024539997
Lrrc8c	95	0.975299119	1.187136071	0.211836951	0.00739329	0.02454506
)15Ertd621(	64	0.834107293	1.095395809	0.261288516	0.0074015	0.024566766
Gm10190	94	45.29330393	45.20870514	-0.084598792	0.00741046	0.024590944
Catsperg1	7	89.95726321	92.30295912	2.345695913	0.00744445	0.024698129
Krt19	8	6.14799365	3.510635506	-2.637358144	0.00744779	0.024703629
Vpreb3	2	74.92979243	64.82518323	-10.1046092	0.0074987	0.024866864
Cldn34c1	20	73.91427122	69.48865737	-4.42561385	0.00750589	0.024885094
Palld	37	5.942448046	5.229848663	-0.712599384	0.00755216	0.025032859
Efna5	126	1.605657776	1.214882367	-0.390775409	0.00756719	0.025077013
Ybx2	112	5.293424318	5.108329053	-0.185095264	0.00757774	0.025106282
Rpl3l	4	77.96153479	84.60518597	6.643651183	0.00758076	0.025110611
Plekho2	48	2.359702493	1.806009775	-0.553692718	0.00763377	0.025280512
110099E03R	1	89.13043478	78.125	-11.00543478	0.00764347	0.025306937
Acsl1	78	1.47606207	1.202348871	-0.273713199	0.00769669	0.025477388
Fendrr	86	5.606238952	7.139103164	1.532864211	0.00774436	0.025629398
Ccser2	86	1.053451176	1.355280627	0.301829451	0.00774762	0.025634409
N6amt2	14	2.684372975	1.97180025	-0.712572724	0.00775345	0.025647894
230323A14R	12	19.37942867	22.51798341	3.138554741	0.00775663	0.025652659
Vangl1	79	1.413425063	1.18873518	-0.224689883	0.00776958	0.02568968
Rbpms2	79	5.659231198	5.48153469	-0.177696508	0.00777654	0.025706917
Rac2	2	66.29437284	54.33054879	-11.96382405	0.00779492	0.025761847
Saysd1	35	1.994360179	1.583964505	-0.410395674	0.00783875	0.025895041
Card14	9	59.1011823	52.48690802	-6.614274288	0.00783786	0.025895041
930503F20R	2	62.84109149	49.74378048	-13.09731102	0.00787569	0.02600746
Sdhaf1	70	1.560552506	1.346368359	-0.214184146	0.00787632	0.02600746
Mir3081	60	27.71811836	28.07643349	0.358315129	0.00790644	0.02610103
Uap1	121	1.839619475	1.422968609	-0.416650866	0.00796567	0.026290646
Gm6277	122	1.716975331	1.343703512	-0.373271819	0.00797537	0.026316739

430531B16R	9	27.92868921	35.18517707	7.256487866	0.00798103	0.026329505
Rqcd1	42	1.77806561	1.33400006	-0.44406555	0.00798576	0.026339185
Ppfia1	53	0.69686519	1.188512443	0.491647253	0.00798946	0.026345453
Cyth1	44	1.393772471	1.863504082	0.469731611	0.00799877	0.026370257
Wnt4	159	8.583752247	9.902221435	1.318469188	0.00801755	0.026426225
Gpd1	1	16.04938272	4.62962963	-11.41975309	0.00802796	0.026454596
Dusp22	59	1.64120553	2.063731289	0.42252576	0.00803284	0.026464738
Patl1	61	0.76917847	1.002223929	0.23304546	0.00803551	0.026467571
Fbxo41	22	6.957314964	8.382192904	1.42487794	0.00804181	0.026482403
Nphp3	64	1.901408938	1.29074748	-0.610661458	0.00805501	0.02651989
Cldn23	54	2.021430922	1.59406078	-0.427370142	0.00810076	0.02666456
Snapc1	28	2.112247091	1.166675178	-0.945571913	0.00813635	0.026775686
Ppp2r4	24	1.876975077	1.280148022	-0.596827054	0.00814131	0.026782156
Trabd	61	1.625040086	1.214444757	-0.410595328	0.00814197	0.026782156
Marveld1	42	0.526286797	0.845370921	0.319084124	0.00818092	0.02689823
Ccdc88a	81	0.974238959	1.208985677	0.234746718	0.00818028	0.02689823
Srsf5	30	2.060298243	1.503454647	-0.556843596	0.00819414	0.026935665
St6galnac5	13	23.7256717	20.35536237	-3.370309336	0.00821621	0.02700217
Plekhg5	44	1.596689776	2.237665072	0.640975296	0.0082365	0.027062799
Zkscan5	12	1.922238026	2.812009836	0.889771811	0.00825657	0.027122656
Dhx35	32	2.089994938	1.605025047	-0.484969892	0.00830092	0.027262231
190002F15R	13	1.454746448	1.071128009	-0.383618438	0.00831482	0.027301792
Pin1	28	1.661444692	1.900091927	0.238647235	0.00833805	0.027371934
Slc25a20	16	1.796050977	0.916115861	-0.879935116	0.00835107	0.027408556
Gm15706	63	1.26314539	0.93569468	-0.32745071	0.00836816	0.027458499
Lcn6	1	53.84615385	71.42857143	17.58241758	0.00837159	0.027463597
Slc5a7	17	37.49868761	37.13133527	-0.367352346	0.00837869	0.02748076
Fras1	32	5.014856057	4.32096967	-0.693886387	0.00839928	0.027542145
Snrnp70	22	1.302369059	1.051953202	-0.250415857	0.00844413	0.027683002
Pi4kb	52	1.242628304	1.325785439	0.083157135	0.00846381	0.027741341
Pcbp2	78	1.556496456	1.372047353	-0.184449103	0.00849517	0.027837899
Hif3a	26	4.116084271	2.894773812	-1.221310459	0.00853624	0.027966231
Ube2e2	30	1.190232031	1.743235551	0.553003521	0.00858522	0.028120444
Fmn1	14	9.336152284	7.82577342	-1.510378864	0.00859304	0.028139774

Enpp2	1	45.28301887	20.83333333	-24.44968553	0.00859661	0.028145168
Znfx1	51	0.973457568	1.226661778	0.25320421	0.00860314	0.028160257
Apold1	4	93.33594371	88.37802795	-4.957915757	0.00861576	0.028195293
Lypla2	8	3.198244784	1.730796335	-1.467448449	0.00863651	0.0282569
Lrrcc1	13	3.048507437	2.46339375	-0.585113687	0.00863914	0.028259206
Sart3	90	1.079877498	1.283431519	0.203554021	0.00865112	0.028292077
Ppp1r8	30	1.234150977	1.833139052	0.598988075	0.00865938	0.028312773
Ube2z	81	1.00571999	1.227479627	0.221759637	0.00867731	0.028365097
)30041F14F	68	1.496513944	1.015479584	-0.48103436	0.00868246	0.028375578
Plk4	55	1.377511061	0.984158928	-0.393352133	0.00874116	0.028561088
Rnf2	95	1.905896463	1.46539769	-0.440498772	0.00876588	0.028635463
Nxph3	70	3.071363866	2.687842666	-0.3835212	0.00878613	0.02869524
Slc1a4	43	1.617903642	1.826218613	0.208314971	0.00879939	0.028732157
Crip1	16	2.502825278	1.814125996	-0.688699282	0.00884483	0.028874082
Sbf1	49	1.469860689	1.167973083	-0.301887606	0.00884747	0.028876295
Hemk1	14	2.019533152	1.478271307	-0.541261844	0.00886249	0.028918884
Trex2	1	98.88888889	91.7721519	-7.11673699	0.00886541	0.02892196
Arih1	137	1.296670342	0.992050159	-0.304620182	0.00890992	0.029060706
Lrrc4c	4	38.10495323	33.74718442	-4.35776881	0.00892898	0.029109933
Unc45a	16	1.150308524	1.71930331	0.568994786	0.00892764	0.029109933
Zswim7	40	1.342399309	1.89024661	0.547847301	0.0089581	0.029198399
Cdk2ap2	44	1.696138196	0.979442139	-0.716696057	0.00896577	0.029216887
Clic5	16	3.873318415	3.032305678	-0.841012738	0.0089729	0.02923364
Gm609	2	77.04126427	62.5	-14.54126427	0.0089956	0.029301094
Mir1199	50	1.738053704	1.171410228	-0.566643476	0.00899792	0.029302144
Manea	42	1.609065268	1.273728715	-0.335336553	0.00900951	0.029333377
Chmp2a	19	39.93833667	38.42589077	-1.512445897	0.00904204	0.029426243
Lancl3	7	25.44348612	21.51109539	-3.932390739	0.0090417	0.029426243
Ipo9	35	0.916651947	1.821859199	0.905207252	0.00904818	0.029439676
Hgf	4	20.66708769	16.84409371	-3.822993973	0.00906766	0.029496513
Mtdh	69	0.869423969	1.099480145	0.230056176	0.00912358	0.029671858
S100a1	5	3.338943366	2.156392264	-1.182551102	0.00913823	0.029712901
Isoc2b	9	5.257477317	3.389522555	-1.867954762	0.00916741	0.029801185
Zdhhc1	17	1.305022895	1.949900142	0.644877247	0.00917271	0.029805224

Hoxc4	2	14.01869159	6.402912263	-7.615779326	0.00917244	0.029805224
Gtf2e2	37	1.086980866	1.363585729	0.276604863	0.00919854	0.029880157
Camk2n2	98	1.789740385	1.352572371	-0.437168014	0.00919985	0.029880157
Mir7053	5	94.86289296	91.00200685	-3.860886104	0.00921645	0.029927448
Dmrt1	36	44.56747252	46.96425106	2.396778545	0.00922864	0.02996041
Msh3	56	1.726136933	1.187591862	-0.538545071	0.00923421	0.029971849
Hes2	8	4.114963737	6.53227492	2.417311184	0.00924939	0.030013693
Shprh	57	1.366808592	1.002954416	-0.363854177	0.00925119	0.030013693
Rab44	4	80.03130989	84.53809892	4.50678903	0.00926661	0.030057094
Mslnl	7	77.27994398	73.93088401	-3.349059971	0.00930717	0.030181962
Tph1	8	4.272484658	2.346481825	-1.926002834	0.00931841	0.030211741
Mir6952	13	96.4606397	95.53669361	-0.923946086	0.00933416	0.030256124
Slc25a14	23	19.25118988	16.55265834	-2.698531547	0.00935009	0.030301051
Cort	6	94.5116623	89.51170912	-4.999953182	0.00936794	0.030352219
Kifc5b	8	1.85335137	1.142751329	-0.710600041	0.00937264	0.030360726
Agap1	107	1.345070383	1.165463779	-0.179606604	0.00940523	0.030459584
Rpl31	32	1.907032156	1.337204826	-0.569827329	0.00941102	0.030471591
Med13	64	1.693084157	1.094547492	-0.598536664	0.00941917	0.030491266
Nop10	7	96.91240444	95.46505987	-1.447344569	0.00942765	0.030511985
Abca12	1	93.10344828	100	6.896551724	0.00943355	0.030524358
Dusp26	25	33.04218793	35.36140758	2.319219654	0.00944045	0.030539938
Ppfia2	7	19.76353224	24.35131348	4.587781245	0.00944756	0.030556204
Ccdc40	30	3.095771062	1.752482391	-1.343288671	0.00948819	0.030680846
Mir1957b	3	91.51009317	94.03508772	2.524994552	0.00949123	0.03068392
AW551984	3	19.1665261	11.07966457	-8.086861531	0.00949924	0.030703059
Tusc3	44	1.179969904	1.541676512	0.361706608	0.00950402	0.030711734
Grin2a	28	60.74190235	57.61225631	-3.129646036	0.00951052	0.03072597
Pwwp2b	131	3.797197436	3.082398246	-0.71479919	0.00953567	0.030800437
Supt5	32	1.645277522	1.277355401	-0.367922121	0.00956053	0.030873946
Pold3	34	1.077720003	0.738120539	-0.339599463	0.00958741	0.030953927
Garem	104	1.690590466	1.422616407	-0.267974059	0.00959779	0.030980645
Wdr93	10	1.949898443	3.111895496	1.161997053	0.00967246	0.031214804
Lingo4	4	13.25713466	12.66361723	-0.593517436	0.00969464	0.031276179
Kitl	60	2.306961997	1.789740032	-0.517221964	0.00969574	0.031276179

Mir1982	5	38.87381202	34.65477058	-4.219041436	0.00971067	0.031317442
[illegible]00003G13[illegible]	1	69.60784314	83.56164384	13.9538007	0.00973107	0.031376345
Mab21l2	2	72.33747042	78.55643254	6.218962122	0.00975071	0.031432753
Scmh1	49	1.47990115	1.100635169	-0.379265981	0.0097652	0.031472556
Pde6c	8	94.6594523	92.74556327	-1.913889032	0.00982408	0.031655366
Ppara	79	3.389319129	4.524656428	1.135337299	0.00984926	0.031729549
Pard3	103	1.66592414	1.411133739	-0.254790401	0.00985288	0.031734232
Snord100	1	0	3.521126761	3.521126761	0.00986358	0.031761728
Nova2	27	6.017488275	7.931607326	1.914119051	0.00987199	0.031781817
Nr1i3	1	91.56626506	77.52808989	-14.03817517	0.00988498	0.031816671
Sun1	72	1.41523137	0.949041377	-0.466189993	0.009901	0.031861252
P2ry1	22	12.99491067	11.35270975	-1.642200917	0.0099301	0.031947872
Tstd1	5	81.64475901	72.97284024	-8.671918778	0.00998829	0.032128057
Etfb	12	1.430574122	3.006972976	1.576398854	0.01000339	0.032169578
Megf9	85	1.767870064	1.43753584	-0.330334224	0.01001603	0.032203148
Ndufaf7	9	0.723521384	1.445228102	0.721706717	0.01002347	0.032220018
Gan	104	1.052810474	0.858534748	-0.194275726	0.01004547	0.032283682
Bambi-ps1	15	4.346879905	3.227295392	-1.119584513	0.01004846	0.032286209
Psph	5	1.185025586	3.144016227	1.958990641	0.01005983	0.032315656
Glis1	95	4.629928017	4.319229426	-0.310698591	0.01008006	0.032373545
Nr0b2	4	54.08108991	48.13989163	-5.941198278	0.01009461	0.032413204
Bmp4	4	30.53245011	40.02215487	9.48970476	0.01012025	0.032488417
Mir684-1	5	65.67832726	58.48726544	-7.191061822	0.01013868	0.032540446
Fbxw5	20	2.256915247	1.684447279	-0.572467967	0.01014613	0.032557255
Rin2	5	2.426082141	0.821608947	-1.604473194	0.01017541	0.032644058
Usp44	3	47.04981376	58.37857224	11.32875849	0.01018145	0.032656295
Pitx3	33	11.8594272	10.69509133	-1.164335863	0.0102069	0.032730792
Zcrb1	58	1.240273572	1.086200456	-0.154073116	0.01021338	0.032744391
Vegfa	23	1.997930244	1.563963111	-0.433967133	0.01022558	0.032776353
Clybl	15	2.120473473	1.729620588	-0.390852886	0.01024479	0.032830754
[illegible]00047M11[illegible]	3	93.95499771	86.34406168	-7.610936031	0.01025539	0.03285756
Akr1b8	11	8.142013363	10.01018796	1.868174594	0.0102603	0.032866118
Enho	14	2.779449221	1.842832116	-0.936617105	0.01026488	0.032873611
Rrm2	21	1.173264581	0.558397635	-0.614866946	0.01031086	0.033013643

500023N17F	28	1.587330035	1.173223724	-0.414106311	0.0103399	0.03309941
Msh4	15	94.55797431	91.94577861	-2.612195691	0.01035002	0.033124565
Ptpru	72	1.785112808	1.529094653	-0.256018155	0.01036384	0.033161569
Ly6h	90	21.76107218	23.79593545	2.034863264	0.01038115	0.033209714
Incenp	60	1.342899172	1.033514842	-0.30938433	0.01042393	0.033339313
Spatc1	4	90.67148819	84.32400932	-6.347478866	0.0104644	0.033461446
Faim2	5	72.56108974	63.13785225	-9.42323749	0.01046822	0.033466359
Ndufs1	18	0.963022299	2.058455165	1.095432866	0.01047846	0.033491824
Sult4a1	39	44.37473911	42.71430478	-1.660434335	0.01048516	0.033505935
Cyfip2	59	13.57291451	15.50404038	1.93112587	0.01054425	0.033687429
Ccdc113	5	86.34191282	82.17139774	-4.170515074	0.01055712	0.033721182
Hmx2	51	3.04475304	4.473984642	1.429231602	0.0105661	0.033742524
Mettl22	24	22.80616052	20.82943825	-1.976722274	0.01058603	0.033798837
Rpl30	30	1.410220133	2.052180336	0.641960203	0.01060041	0.033837388
5-Sep	73	2.18830326	1.687987323	-0.500315936	0.01061593	0.033879546
Odc1	84	8.949956147	9.797056044	0.847099898	0.01063548	0.033934558
Pard6g	45	7.054211548	6.282207955	-0.772003593	0.01065418	0.033986848
330151L19F	52	1.278276792	0.922729753	-0.355547039	0.010659	0.033994816
Klc4	14	6.869219679	4.909685238	-1.959534441	0.01069038	0.034087498
Stradb	32	1.579066818	2.399185659	0.820118841	0.0106955	0.034096419
Srf	88	1.676141104	1.340234416	-0.335906689	0.01071582	0.03415121
Rgs9	5	2.933693742	1.35364689	-1.580046853	0.01071735	0.03415121
Sppl2b	44	1.856815836	1.339274333	-0.517541504	0.01074412	0.034229083
Slmo1	27	1.447102856	0.995166955	-0.451935902	0.01074766	0.034232942
Sgcb	17	5.120524102	8.154067955	3.033543853	0.01080451	0.034406552
Tvp23b	29	2.129881694	1.495891356	-0.633990339	0.01082101	0.034451625
S1pr1	10	14.82979228	20.51773002	5.687937739	0.01082573	0.034459162
30035M05F	2	6.18729097	4.06504065	-2.122250319	0.01085355	0.034540229
Igfbp6	18	81.35088338	83.17813103	1.827247653	0.01094123	0.034811714
'00027A15F	1	70.11494253	54.41860465	-15.69633788	0.01102837	0.035081369
Oas2	2	46.74447174	39.70449583	-7.039975913	0.01103427	0.035092529
Arhgef15	2	76.83758113	70.90484794	-5.932733197	0.01104599	0.03512219
330454E08F	29	1.399834175	0.837889855	-0.56194432	0.01111011	0.035318393
Gm14207	54	2.448465972	1.958976934	-0.489489038	0.01111296	0.03531982

Mfsd1	9	1.820128699	0.55011822	-1.270010479	0.01112404	0.035347362
Mb21d2	38	1.803061226	1.123537313	-0.679523913	0.0111379	0.035383766
Slc41a1	19	1.44700265	0.789138046	-0.657864605	0.01116489	0.035461833
Tmod2	21	14.35875663	13.07751667	-1.281239959	0.01117935	0.035500068
Cln6	46	1.086689465	0.848972108	-0.237717357	0.01120152	0.03556277
Ppp2r1a	35	1.73108238	0.996816675	-0.734265705	0.0112461	0.035696572
Nlgn2	2	41.63059027	49.42091929	7.790329018	0.01129608	0.035847464
Zbtb5	56	1.610093244	1.155956615	-0.454136628	0.01130401	0.03586489
Creb3l4	7	61.3420058	57.81575349	-3.526252304	0.01132497	0.035923622
Tuba1c	21	1.702052933	1.063699909	-0.638353024	0.01135563	0.036013064
Mical2	6	3.262855598	1.616513256	-1.646342342	0.01137955	0.036081146
Scn9a	6	38.59097456	46.64614122	8.055166657	0.0113871	0.03609727
Cdkl5	33	18.93972231	16.79547986	-2.144242454	0.01140211	0.036137058
Il15	11	2.075989124	3.527951373	1.451962249	0.01140632	0.036142579
Polq	19	2.173936082	1.709475477	-0.464460604	0.011422	0.036184475
500009L16F	4	17.97498057	12.39793051	-5.577050063	0.01142656	0.036186477
Rbm33	95	1.25919854	1.021550756	-0.237647784	0.01142757	0.036186477
Fblim1	21	4.228673914	3.845268235	-0.383405679	0.0114328	0.036195241
Elmod2	43	1.227829501	0.846214459	-0.381615041	0.01144373	0.036219663
Ube2d1	83	1.520806657	1.337697491	-0.183109166	0.01144545	0.036219663
Med16	10	0.518685567	2.471907936	1.953222369	0.01150176	0.036382147
Cemip	10	1.257149819	2.133832104	0.876682285	0.01149929	0.036382147
Plekho1	127	1.271110884	1.063877107	-0.207233777	0.01157049	0.036584511
Cnksr3	57	1.180572154	0.785102512	-0.395469642	0.01157072	0.036584511
Lactbl1	3	66.91989187	75.4198941	8.500002231	0.01157741	0.036597791
Fig4	2	9.418587579	4.236378713	-5.182208866	0.01162283	0.036733445
Gata5os	7	95.99114888	94.01839724	-1.972751632	0.01163561	0.036765922
Mir7116	2	100	97.65317421	-2.346825787	0.01170417	0.036974599
Sec1	7	95.70767875	98.3489652	2.641286442	0.0117447	0.03709464
Ctnna2	43	48.64562407	46.11668001	-2.528944056	0.01177328	0.037176919
Smtnl2	37	3.656994964	2.527652448	-1.129342516	0.01178962	0.037220486
Mmp24	100	2.810862106	2.098436541	-0.712425564	0.01179907	0.037242308
Tmbim1	52	1.077616116	1.496895446	0.41927933	0.01191557	0.037601942
Kcnj10	18	16.33137874	13.25495671	-3.076422025	0.01195287	0.037711544

Irs4	27	53.53590889	51.03124882	-2.504660062	0.01199414	0.037833609
Zfp266	21	0.957434788	1.610819916	0.653385128	0.01199833	0.037838694
Unc93b1	8	23.46067287	26.20443564	2.743762766	0.01200206	0.037842329
Anp32b	115	1.343197916	0.980252925	-0.362944991	0.01202573	0.037908825
Epb4.1l1	62	2.182863636	1.336177437	-0.846686199	0.01205451	0.037991385
Gata4	51	2.840329106	2.261311619	-0.579017487	0.01211226	0.038165192
Lims2	22	4.9609722	7.137256355	2.176284155	0.01211993	0.038181163
Pcsk1	6	25.15798487	24.84389453	-0.31409034	0.01213778	0.038229177
Casp9	12	2.449087512	1.249897408	-1.199190104	0.01215773	0.038283793
Ftsj3	16	1.611413254	0.993738851	-0.617674403	0.01216305	0.03829234
Sft2d1	12	2.424512801	1.584865569	-0.839647232	0.01220663	0.038421311
Elac2	20	2.835159327	2.015988978	-0.819170349	0.01222157	0.038460057
Ide	1	96.72131148	100	3.278688525	0.01222742	0.038470221
Grm4	1	83.58208955	65.33333333	-18.24875622	0.01224012	0.038501927
Tmem51os1	39	1.532444895	1.226016726	-0.306428169	0.01226491	0.038571644
Dennd2d	14	6.371287181	8.536309535	2.165022353	0.01231095	0.038708149
Psme2	17	1.85473226	2.720851471	0.866119211	0.01231477	0.038711864
)30515G01F	99	1.034446506	1.185699772	0.151253266	0.01233653	0.038771939
Zfp551	3	20.83731132	15.435132	-5.402179327	0.01236631	0.038857212
Sertm1	22	42.48492386	45.50820166	3.023277795	0.01237877	0.038888039
Sh3gl2	16	28.98413166	26.1412872	-2.842844457	0.0123828	0.038892368
Edn1	2	0	3.689299979	3.689299979	0.01239087	0.038909386
Hunk	79	5.123898996	5.671712167	0.547813171	0.01241548	0.03897834
Atp5j2	18	1.717280997	1.026123684	-0.691157313	0.01242044	0.038985566
Sun3	10	3.451790512	2.431351906	-1.020438606	0.01244354	0.039049737
Lpgat1	94	1.251663562	1.484982585	0.233319023	0.01245788	0.039086358
Tango2	25	1.100408482	1.503163806	0.402755324	0.01247904	0.039144379
;10009B22F	40	2.148541599	1.159985088	-0.988556511	0.01256892	0.03941789
Fam84b	12	0.979163005	1.593150182	0.613987177	0.01262779	0.039594059
'00011E24F	9	91.26166392	94.73676852	3.475104599	0.01263801	0.039617634
Ranbp9	225	0.997273182	1.129987246	0.132714064	0.01266832	0.039704184
Suox	34	2.040744254	1.291034447	-0.749709807	0.0126784	0.039718791
Vamp5	42	1.791387517	1.540223785	-0.251163732	0.01267722	0.039718791
Saxo2	30	1.054290821	1.378484393	0.324193571	0.01275178	0.039940159

Blvra	24	2.249624111	1.324050104	-0.925574008	0.01277414	0.04000165
Ptk2	99	1.253480835	1.162521683	-0.090959152	0.01277975	0.040010693
Adssl1	31	1.987852776	1.363659449	-0.624193327	0.0127921	0.040040815
Mmd	67	1.386389491	1.454138053	0.067748562	0.01282554	0.040136911
Rab3d	24	1.467960773	0.816177727	-0.651783046	0.01287165	0.040264051
Selt	45	1.351278915	0.86649096	-0.484787955	0.01286911	0.040264051
Adam22	31	16.91528205	20.39503394	3.479751894	0.01291057	0.040377163
Adra2b	39	14.47125643	17.58142862	3.110172186	0.01294749	0.040484002
Chgb	17	11.03016311	10.94381567	-0.086347444	0.01296693	0.040536154
Lrsam1	4	1.7578125	2.521728759	0.763916259	0.01297396	0.040549501
H2-L	43	3.815283928	3.26991248	-0.545371448	0.01298874	0.040587032
Abcc3	12	4.964813197	6.081544801	1.116731604	0.01303082	0.040709857
Prkrip1	26	1.377432907	0.9711116	-0.406321307	0.01305344	0.040764762
Zfp865	32	2.365967532	1.765322412	-0.600645121	0.01305395	0.040764762
Psmd5	18	2.304763289	2.883832672	0.579069383	0.01311306	0.040940648
Scp2	14	1.280202984	0.367588099	-0.912614885	0.01313792	0.041009546
Fam110a	63	2.391086575	1.565142594	-0.825943981	0.01318174	0.041137584
Cdc40	15	0.907519914	1.928773954	1.02125404	0.01319493	0.04116999
Atp8b2	55	1.663875579	1.516577051	-0.147298528	0.0132054	0.041193879
Pde9a	21	2.146008894	1.64227598	-0.503732915	0.01322431	0.041244099
Sema3d	6	13.16771692	8.893480906	-4.274236017	0.01330616	0.041490566
Pcdh1	6	3.789693989	1.6095497	-2.18014429	0.01334916	0.041615804
B3galt4	39	2.482650852	2.080031361	-0.402619492	0.01337329	0.041673312
Elovl5	70	0.981166706	1.126470547	0.145303841	0.01337105	0.041673312
Dact2	59	21.69150116	21.32926075	-0.362240414	0.01341798	0.041803699
Cript	9	1.131186245	2.578445116	1.44725887	0.01347885	0.041984431
Jmjd1c	134	1.16819141	1.023837041	-0.14435437	0.0135011	0.042044809
Ctrl	2	94.82142857	89.18496387	-5.636464703	0.01351221	0.042070465
Arhgap33	61	3.398170165	4.668752475	1.27058231	0.01352753	0.042109242
Creg1	31	0.909506462	1.535392532	0.62588607	0.01353944	0.04213739
Rgl3	20	15.69188167	18.03998216	2.348100491	0.01362891	0.042406828
Elp4	23	1.46341988	1.250774421	-0.212645459	0.01367331	0.042535962
Terf2	3	2.018131652	3.905373628	1.887241977	0.01370127	0.04261391
Wdr20rt	16	93.0721954	91.37120615	-1.700989254	0.01370427	0.042614212

Mir99ahg	7	2.397971871	1.917951905	-0.480019967	0.01370818	0.042617343
Xkr4	85	28.5128382	30.46142548	1.948587273	0.01372421	0.042658134
Glis2	77	1.31909415	1.633842013	0.314747862	0.01375393	0.042741454
Rpl35	66	1.581035202	2.080510923	0.499475721	0.01375842	0.042746372
Ica1	32	1.373408037	0.947789643	-0.425618394	0.01377241	0.042780772
Rnh1	40	1.874132212	1.438607577	-0.435524635	0.01379449	0.042840285
Mri1	39	1.568331435	1.161638472	-0.406692963	0.01387478	0.043080502
Zfp932	4	4.21592185	2.249056604	-1.966865246	0.01390566	0.043167255
Rtel1	18	1.656844782	0.88902091	-0.767823872	0.01391383	0.043183503
Rasgef1a	5	2.676429941	1.179238427	-1.497191513	0.01394493	0.04327086
Svop	15	4.795654779	6.08324797	1.287593192	0.01395516	0.043284298
Mettl7a3	2	37.62012987	29.6477173	-7.972412574	0.01395433	0.043284298
Mast4	41	27.67034242	29.41441713	1.744074702	0.01399319	0.043393079
Snx8	47	1.523327853	1.993944679	0.470616826	0.01400748	0.043428209
Lonp2	16	2.323503819	1.387644642	-0.935859176	0.01403229	0.043495947
Fyttd1	68	1.226512648	1.074202082	-0.152310566	0.01403918	0.043508111
Ufsp1	23	2.140391894	1.69788066	-0.442511233	0.01405555	0.043549652
Cbfb	85	1.154446593	1.263965782	0.109519188	0.01417537	0.043911626
Tmem246	14	1.301556179	2.187700075	0.886143896	0.01418228	0.043923763
Trim26	66	1.578389586	1.311728981	-0.266660605	0.01419033	0.043939404
Swsap1	1	12.67605634	2.53164557	-10.14441077	0.01419953	0.043958639
Car2	74	2.281498571	2.518622042	0.237123472	0.01423169	0.044048894
Cib2	44	6.175641484	8.410213652	2.234572168	0.01431472	0.044296528
Exosc6	59	0.92539984	1.063143233	0.137743393	0.01432463	0.044317856
.30017N08F	9	58.11750432	60.42671997	2.309215653	0.01437744	0.04447188
'00046A07F	4	41.80501315	36.23272842	-5.572284728	0.01441492	0.044578401
Rab31	76	1.853525454	1.519058206	-0.334467248	0.01441884	0.04458112
Car3	17	25.27439936	23.50618231	-1.768217049	0.01442316	0.044585084
Vstm4	47	56.27789786	57.32976213	1.051864266	0.01443296	0.044605988
Gm5617	32	5.929365733	8.432681969	2.503316236	0.01447038	0.044712213
Ptprk	51	1.449471344	0.935868597	-0.513602746	0.01451616	0.044834797
Caln1	20	56.84940336	57.63745327	0.788049904	0.01451588	0.044834797
Dmrt3	84	10.95446352	12.95910468	2.004641165	0.0145194	0.04483537
Timp2	73	1.618316203	1.722474984	0.104158781	0.01455997	0.044951201

Tmem14a	7	0.886699507	2.02876571	1.142066203	0.01459241	0.045033116
Znhit6	5	0.306122449	1.312911446	1.006788997	0.01459264	0.045033116
Cyp2s1	15	3.017971719	2.000938224	-1.017033495	0.01463083	0.045141469
Cat	2	5.303030303	0.689655172	-4.613375131	0.0146671	0.045243857
Phip	71	1.146722256	0.907558608	-0.239163648	0.01470654	0.045355996
Clcf1-pold4	9	2.273460906	0.920962156	-1.35249875	0.0147469	0.0454709
Srbd1	34	8.826324435	7.481639881	-1.344684554	0.01475669	0.045491525
Syce2	36	6.653267828	6.671861783	0.018593955	0.01478694	0.045575201
Src	113	1.454735877	1.158118638	-0.296617239	0.01480077	0.045608253
Mprip	115	0.960017778	1.085656814	0.125639037	0.01482957	0.045687407
Gtf3c2	2	2.083333333	0	-2.083333333	0.01483492	0.045694281
Il17ra	70	3.082369993	2.18116741	-0.901202584	0.0148384	0.045695409
Rps9	6	0.293525459	1.761541608	1.468016148	0.01484859	0.045717198
'00080O16F	4	83.72594378	78.50710971	-5.218834072	0.01487382	0.045785281
Gpbp1	128	1.852169625	1.433710801	-0.418458823	0.01488401	0.045807044
Cnot6	42	0.832588387	1.229491594	0.396903207	0.01504549	0.0462943
Arsk	7	3.970971766	6.57357847	2.602606704	0.01505066	0.046300505
Optc	1	72.09302326	62.71676301	-9.37626025	0.01507102	0.046353419
Procr	9	1.65157051	1.19702012	-0.454550389	0.01507741	0.046363361
Thsd7a	21	4.066112519	3.267332936	-0.798779583	0.01512586	0.046502598
Gnpda2	29	2.719597039	1.908719039	-0.810878	0.01516925	0.046626222
Rom1	37	7.772583239	6.177314023	-1.595269216	0.0151987	0.04670697
Tbca	43	1.176064301	0.808588088	-0.367476213	0.01525735	0.046877386
Epo	9	36.95821098	35.02416261	-1.934048374	0.01528368	0.046948454
Zkscan4	5	5.295983087	2.582525937	-2.71345715	0.0152912	0.046961717
Dcaf13	22	1.658609803	0.971539056	-0.687070747	0.01529793	0.046972545
Hspa8	70	1.639158325	1.43073105	-0.208427275	0.01532946	0.047059505
Rin1	13	2.448470297	1.958714546	-0.48975575	0.01535905	0.047140491
Cmtr1	22	1.415059842	0.980173147	-0.434886696	0.01541568	0.047304421
310010J17R	56	1.56976421	0.911376357	-0.658387853	0.01542192	0.047313657
Tec	34	2.072352044	1.612779044	-0.459573001	0.0154557	0.0474074
Rnf5	20	0.715136823	1.724064993	1.00892817	0.01547729	0.047463701
Pitpnm2	119	1.692648376	1.921330979	0.228682603	0.01548675	0.047482763
Pycr1	5	3.835346091	2.019480519	-1.815865572	0.01550623	0.047532559

Cnksr1	15	9.013408449	7.315357959	-1.69805049	0.01554047	0.047627575
Bola3	50	1.755148966	1.949818044	0.194669077	0.0155505	0.047648355
Nt5c3	63	0.721001102	1.03177563	0.310774529	0.01558463	0.047742965
Fam187b	19	12.52840524	11.55458785	-0.973817391	0.01562912	0.04786928
D8Ertd738e	13	2.160879921	1.261167517	-0.899712405	0.01570669	0.048096814
Ncam1	14	3.622353716	2.011834375	-1.610519341	0.01572788	0.048151664
Cep126	49	2.249107182	1.797376861	-0.451730322	0.01573555	0.048165081
Eif2b3	30	8.037450982	7.433176548	-0.604274434	0.01574216	0.048175267
Slfn5	8	30.3274533	34.61091715	4.283463851	0.0157921	0.048318017
Zpbp2	12	2.191379006	1.42058336	-0.770795646	0.01582164	0.048398311
Mir6935	37	0.928455007	1.554705165	0.626250158	0.015851	0.048478026
Birc6	58	1.183739865	1.250475615	0.06673575	0.01586985	0.048525556
Snhg12	11	19.02961731	17.02400248	-2.005614837	0.01588526	0.048562555
310024B03F	6	1.115506332	0.431190995	-0.684315337	0.01590949	0.048626496
Myo6	57	1.636694636	1.894747395	0.258052759	0.01597188	0.048807036
Csmd1	61	55.84114516	56.81041625	0.969271087	0.0160416	0.049009882
Zfp30	19	2.573629495	2.085147603	-0.488481891	0.01608392	0.049128929
Hbs1l	30	1.6091629	1.12295472	-0.48620818	0.01610093	0.049170661
Bloc1s4	39	1.242351759	0.661341769	-0.58100999	0.01612147	0.049223143
Eif3k	6	0.957373072	2.252766726	1.295393654	0.01614043	0.049270785
Mbp	44	17.38437208	15.95167131	-1.432700774	0.01615545	0.049306365
Fem1a	32	0.985331028	1.471925487	0.486594459	0.01616363	0.049321084
En1	34	37.87445564	36.18365867	-1.690796969	0.01617095	0.049333163
Mir3569	69	2.602861953	1.78748444	-0.815377513	0.01621911	0.049469817
Dctn4	28	1.479385218	0.87482306	-0.604562158	0.01624109	0.049516252
Gm2a	13	4.944925352	6.631205074	1.686279721	0.01623778	0.049516252
932416K20F	1	89.91596639	80.44280443	-9.473161959	0.01628469	0.049638867
Dock8	120	1.286596571	1.071018852	-0.215577718	0.01633008	0.049766904
Zfp28	20	1.25181767	1.92936464	0.67754697	0.01636135	0.049851848
Pls3	8	23.55811285	20.10303419	-3.455078665	0.01637736	0.049890272
Setd7	50	1.712776748	1.09842108	-0.614355668	0.01638475	0.049902404
Rasal3	5	66.33494908	61.40122471	-4.933724366	0.01640972	0.049968106
Cldn1	5	10.31215507	8.486994462	-1.825160609	0.01649071	0.05020429
Stk19	46	1.631840348	1.271762413	-0.360077935	0.01655657	0.05039433

Tnfaip8l3	21	21.14979337	20.32768382	-0.822109551	0.01657714	0.050446498
Myl12b	77	1.58803133	1.196559104	-0.391472226	0.01668225	0.050755813
Mir684-2	2	95.51282051	88.7755102	-6.737310309	0.01670235	0.050806445
Ccdc64	157	1.465584201	1.143747085	-0.321837116	0.01686406	0.051287727
Slc5a8	4	33.0615942	38.99860398	5.937009779	0.01692507	0.051462603
Rsrp1	55	1.366827787	1.114744821	-0.252082966	0.01696934	0.051586525
930429F24R	9	2.039584975	0.825911861	-1.213673114	0.01697316	0.051587442
Ints2	9	2.207977208	0.696586929	-1.511390279	0.0169838	0.051609089
700056E22R	68	1.202350259	1.27126375	0.068913491	0.01699804	0.051641668
Snrpa1	34	1.194210372	0.922798106	-0.271412267	0.01710369	0.051941494
430020K01R	72	1.067020327	1.253545667	0.18652534	0.01710381	0.051941494
Ppp2r5e	98	1.437345358	1.110830845	-0.326514513	0.01711809	0.051963347
Kif6	15	17.90259133	22.9037615	5.001170164	0.01711729	0.051963347
Sec63	33	0.987231447	1.413987518	0.426756071	0.01712587	0.051976209
Abcd2	5	11.77604203	8.309323947	-3.466718088	0.01715487	0.05205348
Rgsl1	5	95.56235134	86.91882432	-8.643527017	0.01716638	0.052068201
700125H20R	8	0.947013248	2.073146529	1.126133282	0.01716682	0.052068201
Sirt7	20	1.594938048	1.033378926	-0.561559122	0.01718911	0.052125014
Fgf17	17	35.62241442	34.69624951	-0.926164915	0.0172064	0.052145199
Gm1564	24	84.12103949	81.91074824	-2.210291253	0.01720642	0.052145199
Rnf148	1	70	48.14814815	-21.85185185	0.01720178	0.052145199
Uvssa	47	1.736026882	1.269773315	-0.466253567	0.01722052	0.052177153
Fut10	25	1.640393185	0.729506008	-0.910887177	0.01723171	0.052189486
Acin1	10	1.892690237	1.173484955	-0.719205281	0.01723101	0.052189486
Zfp273	6	2.16354911	1.517548323	-0.646000786	0.01723741	0.052195996
Ndufv3	2	0	1.428571429	1.428571429	0.0172465	0.052212744
S100a11	20	2.18517292	1.688506753	-0.496666167	0.0172589	0.052230712
Mpdz	65	1.92091056	1.611706798	-0.309203762	0.01725956	0.052230712
Il17c	5	93.60683761	88.50809521	-5.098742398	0.01728221	0.052288472
Gabrq	6	47.46285039	39.94154568	-7.521304713	0.01729124	0.052305033
Hdac8	3	20.73107031	13.90166728	-6.829403037	0.01730078	0.052323086
Nfkb1	109	1.938595671	1.318130593	-0.620465078	0.01733128	0.052404535
Skida1	22	4.420727478	1.979350958	-2.44137652	0.01734768	0.05244333
Runx1	51	1.53593855	1.372936873	-0.163001678	0.01736657	0.052489606

Nkain1	72	1.196988554	0.992521215	-0.204467339	0.01743258	0.052678265
Zbtb48	35	2.710063979	2.433862642	-0.276201337	0.01745273	0.052704401
Kif5b	57	1.501179617	1.302436325	-0.198743291	0.0174546	0.052704401
Agpat9	30	1.813836702	1.336432776	-0.477403926	0.01745559	0.052704401
Mrpl3	21	19.70317174	16.88377339	-2.819398357	0.01745324	0.052704401
Ssna1	23	1.265095928	0.875231873	-0.389864055	0.01745964	0.052705781
Inppl1	30	1.53544794	1.035299326	-0.500148614	0.0174715	0.052730731
Pam	78	2.133269878	1.695705688	-0.43756419	0.01755925	0.052984677
Chsy3	93	1.570369462	1.490423272	-0.07994619	0.01759032	0.053067494
Smg7	64	1.203899012	1.408008498	0.204109486	0.01761794	0.053128984
Pxdn	14	2.982135993	2.128790377	-0.853345616	0.01761605	0.053128984
Eogt	34	1.843437467	1.20731547	-0.636121997	0.01763319	0.053164045
Pip4k2a	70	0.941460593	1.197944357	0.256483764	0.0176498	0.053203185
Usp35	16	66.5192531	68.94189449	2.422641386	0.01773005	0.053434117
Fxyd1	1	40.4040404	25.2173913	-15.1866491	0.01774855	0.053478899
Qars	17	1.3893062	0.531254703	-0.858051497	0.01779336	0.053602923
Gcc1	8	1.788220318	0.866581777	-0.921638541	0.01782162	0.053666019
Adm2	3	46.80853217	39.95231395	-6.856218221	0.01782028	0.053666019
Cd1d1	4	7.750837872	11.58558568	3.834747804	0.01783723	0.053702004
Pantr2	8	33.95477297	27.12221002	-6.83256295	0.01785182	0.053734895
Pde6d	27	1.963236128	1.234963219	-0.728272908	0.01789001	0.053838809
Ppp2r2b	15	14.29048282	15.76542602	1.474943194	0.01789861	0.05385363
Mfsd6	69	1.467532709	1.098949171	-0.368583538	0.01790983	0.053876359
Gm8580	1	83.56164384	92.46575342	8.904109589	0.01791864	0.053891792
Angel2	76	1.188907717	0.846123418	-0.342784298	0.01793627	0.053929857
Tfcp2	52	1.607717138	1.339617604	-0.268099534	0.01793864	0.053929857
Ankrd27	30	1.852056135	2.317304961	0.465248826	0.01794866	0.053948936
Lypd6	59	27.38484063	30.65245322	3.26761259	0.01796674	0.053992218
Mtfr2	25	1.55375064	1.020453426	-0.533297214	0.01797665	0.054010913
Zfp954	3	4.081444629	0.843881857	-3.237562772	0.01799491	0.054054715
Kctd5	46	1.62366559	1.339142398	-0.284523193	0.01803293	0.054157845
Ero1l	87	1.286561359	0.870868314	-0.415693045	0.01804323	0.054177678
Zfp800	158	1.290542795	1.300258397	0.009715602	0.01805008	0.054187163
Mpp3	77	2.595954753	1.616858435	-0.979096318	0.01805403	0.054187939

Mfsd5	67	1.13599461	1.279198598	0.143203987	0.01808021	0.054255405
Snx5	93	1.164815277	1.302112261	0.137296984	0.01811239	0.054340878
Rab30	4	2.489630459	1.366176236	-1.123454223	0.01816925	0.054500333
Adat2	13	1.167021166	0.313866055	-0.853155111	0.0181809	0.054524134
Tex2	64	1.25661783	1.561993396	0.305375566	0.01821983	0.054629714
Nr4a1	45	1.978382129	3.055012432	1.076630303	0.01824706	0.054700183
Recql4	28	1.456963814	0.990163642	-0.466800172	0.01831136	0.054881706
Usp22	30	1.515315695	0.880008249	-0.635307445	0.01834804	0.054980411
Gna13	78	1.879142819	1.248832483	-0.630310337	0.01837585	0.055052521
Per1	6	0.483091787	2.31004156	1.826949773	0.01841308	0.055152808
Lingo3	56	14.64700212	16.51165024	1.864648114	0.01842439	0.055175421
Khnyn	30	1.300481888	1.711353258	0.41087137	0.01842832	0.055175919
Tcf15	46	21.98336947	23.85301538	1.86964591	0.01846608	0.055277707
Gnl3l	25	19.4774898	16.86712123	-2.61036857	0.01847922	0.05530576
Lrch3	63	1.79808537	1.381623962	-0.416461409	0.01851073	0.055388749
Stoml2	22	1.252666348	1.524291018	0.27162467	0.01853533	0.055451056
Atat1	14	2.376163932	1.682579928	-0.693584004	0.01856576	0.055530774
Gm15908	56	2.205838166	1.864266503	-0.341571663	0.01859093	0.055594727
Dhrs1	7	1.839037253	0.62467998	-1.214357273	0.01860376	0.055621757
Edc4	22	0.912519528	1.951441514	1.038921986	0.01863147	0.055685058
Ctsb	25	1.270291549	0.63913738	-0.631154169	0.01863252	0.055685058
Aspm	26	1.48731925	0.974352292	-0.512966958	0.0186761	0.055803926
Epha3	17	26.49191323	25.73249555	-0.759417681	0.01888435	0.056414692
Fgd4	40	1.368278612	1.008515954	-0.359762658	0.01889607	0.056438218
Ttll10	2	30.31914894	23.21981424	-7.099334695	0.0189201	0.056498487
Snrpd2	3	3.289370123	1.442307692	-1.847062431	0.01898777	0.056677486
Nbeal2	63	1.244698714	0.974474791	-0.270223923	0.01898426	0.056677486
Ccdc74a	83	6.875941107	6.420213177	-0.45572793	0.01900447	0.056714065
Nubpl	12	1.464889591	1.188083433	-0.276806157	0.01900775	0.056714065
Noxa1	12	71.99852108	69.58843691	-2.41008417	0.01902947	0.056767312
Armc1	12	1.906175007	2.146168884	0.239993877	0.01903832	0.056782189
Copz2	21	2.607897604	3.392652483	0.784754879	0.01910729	0.056976297
Cyp20a1	35	1.923765079	1.102984226	-0.820780854	0.01912561	0.057019353
Tfdp1	91	1.445431772	1.043266306	-0.402165465	0.01914026	0.057051434

Ap2a1	58	1.676684085	1.259830984	-0.416853101	0.01914823	0.057052016
Tnfsf9	11	0.634060967	1.69151613	1.057455163	0.01914807	0.057052016
Grasp	44	1.581960199	1.382620295	-0.199339905	0.01915553	0.057062155
Zfp574	46	2.043543231	1.608702313	-0.434840918	0.01918451	0.057136885
Ets2	66	1.3417087	1.609818074	0.268109374	0.0191886	0.057137478
Ophn1	34	15.41307832	14.16001126	-1.253067055	0.01921032	0.057167368
Zfp709	30	4.857754184	2.637584101	-2.220170083	0.019205	0.057167368
Wfdc2	1	26.93726937	19.06077348	-7.876495892	0.01920892	0.057167368
Galnt1	220	1.049316184	1.184105171	0.134788988	0.019231	0.057217295
Pdgfb	38	1.636990625	1.145035185	-0.49195544	0.0192419	0.057238125
Mirc35hg	85	2.740271164	2.342059249	-0.398211915	0.0192632	0.057289856
Mknk1	5	2.772354883	1.397822644	-1.374532239	0.01927977	0.057325744
Slc25a3	56	1.228872585	1.580906435	0.35203385	0.01928308	0.057325744
Ric3	40	51.23921574	53.45560513	2.21638939	0.0192924	0.057341848
Cops3	45	4.660678879	5.370431514	0.709752635	0.01933719	0.057463326
Rsad1	18	1.908786956	1.270849909	-0.637937047	0.01937597	0.057566898
Ccpg1	26	0.687484243	1.347386527	0.659902284	0.01938195	0.057573034
530011O06F	1	46.37681159	30	-16.37681159	0.01943958	0.057732524
Vldlr	42	4.425755114	4.852195491	0.426440377	0.01947265	0.057819046
Arhgef40	73	3.137915044	2.102248926	-1.035666118	0.01948066	0.057831115
Gm10637	4	19.91212325	17.04948325	-2.862639995	0.01962883	0.058235634
Gm9866	3	89.05184569	85.19018778	-3.86165791	0.01962768	0.058235634
Pitx1	63	24.24636825	24.39324035	0.146872097	0.01962435	0.058235634
Ksr2	44	9.816880527	10.5187507	0.701870174	0.01963536	0.058243225
Cpne5	61	7.716083503	9.113628102	1.397544599	0.01967398	0.058346008
Nrde2	3	0	2.046783626	2.046783626	0.01970574	0.058428389
Nt5c2	101	11.22776863	11.98989226	0.762123631	0.01976924	0.058604811
Tmem120b	55	1.443044054	1.165916871	-0.277127182	0.01979257	0.058662123
Frmd8	2	5.681818182	1.685393258	-3.996424923	0.01989006	0.058939174
Bloodlinc	3	22.67379774	29.92565326	7.251855516	0.01989991	0.058946059
Rnf10	40	2.408507279	1.298226841	-1.110280438	0.01990042	0.058946059
Zfp236	128	1.105694161	1.238896172	0.133202011	0.0199734	0.059138345
Sprtn	66	0.96121343	1.080446951	0.119233521	0.01997165	0.059138345
310030O07F	18	8.468017774	10.44250486	1.974487085	0.01998222	0.05915252

Lrig1	86	1.418728194	1.171918439	-0.246809755	0.01998908	0.059160915
St8sia6	58	10.97376448	10.86724496	-0.106519521	0.02007157	0.059393069
Kcnq2	71	15.08373765	17.12310584	2.039368188	0.02009521	0.059451025
Uba5	41	1.260757635	1.813015438	0.552257803	0.02010607	0.059459193
AF067063	3	88.56952859	83.82838284	-4.741145756	0.02010338	0.059459193
Slc17a6	10	38.1778526	41.55961208	3.381759474	0.02014915	0.059574569
Mrps9	14	2.158231644	1.674302086	-0.483929558	0.02021641	0.05976141
Zfp354b	3	3.315118397	1.088490224	-2.226628173	0.02024149	0.059811429
Cyb5b	56	10.14620097	8.810930925	-1.335270046	0.02024087	0.059811429
Zfp945	10	5.868310533	3.991761411	-1.876549122	0.02028704	0.059921913
Rnf17	19	88.0055744	85.8878331	-2.117741297	0.02028667	0.059921913
Tspan15	24	2.768913051	1.665954944	-1.102958107	0.0203974	0.060235739
Adgrl3	22	3.680740459	2.721151697	-0.959588762	0.02045003	0.060379015
Fam73b	31	1.568435865	1.408320677	-0.160115189	0.02051588	0.060561272
Slc4a10	4	68.59469899	62.54598406	-6.048714934	0.02053674	0.060610635
Plscr3	14	1.984335174	1.261772002	-0.722563172	0.02060529	0.060800724
Tmed3	49	2.196897975	1.792549906	-0.404348069	0.02062804	0.06085563
Zfp62	24	1.178239762	1.646354171	0.46811441	0.02069152	0.061026408
Thoc6	18	1.477044211	0.880931772	-0.596112439	0.02069425	0.061026408
Tspyl3	3	53.05673009	43.25955734	-9.797172748	0.02070513	0.061046236
Copb1	37	1.32913603	1.169316455	-0.159819574	0.02070962	0.061047187
Prss56	3	61.77777778	70.0909324	8.313154625	0.02071415	0.061048303
Nt5c	30	1.471888494	0.976506366	-0.495382128	0.02073262	0.061090458
Smc2os	13	2.921399799	2.033762744	-0.887637055	0.02076062	0.061160685
Klf17	1	91.66666667	97.70114943	6.034482759	0.02076761	0.061168988
Chp2	5	86.20728291	80.5432111	-5.664071814	0.02077459	0.061177256
Mpped2	48	46.00728071	44.93150045	-1.075780261	0.02081505	0.061284109
Fgf18	149	1.908225374	1.50424825	-0.403977124	0.02083426	0.061328385
C1qtnf5	14	72.64710663	74.99113401	2.34402738	0.02084789	0.061356191
Nckap5	20	41.48828544	43.96216505	2.47387961	0.02086619	0.061397733
Abhd17b	129	1.243252072	1.062364845	-0.180887227	0.02087829	0.061421011
330406C13R	16	2.183992207	1.963262999	-0.220729207	0.02091494	0.061516489
Cep170b	114	1.461449973	1.64246917	0.181019197	0.0209228	0.061527288
Mir127	4	95.28121739	92.62323962	-2.657977771	0.02093351	0.061546441

Gpat2	11	84.12866835	82.57441332	-1.554255031	0.0209762	0.061659599
Ece1	26	1.767635475	1.048327454	-0.719308021	0.02099796	0.061711194
Unc79	20	32.6486331	29.46771427	-3.180918824	0.02101648	0.061753276
Amotl2	24	0.969259926	1.954352763	0.985092837	0.0210349	0.061785006
Mir7003	2	93.97216864	97.54998523	3.577816585	0.0210357	0.061785006
Rgs10	96	1.96340574	1.402301027	-0.561104713	0.02109051	0.061933587
Pstpip2	72	8.496366789	8.179755316	-0.316611473	0.02109973	0.061948271
Zdhhc6	33	0.890283252	1.250039619	0.359756367	0.02113728	0.062046091
Ldoc1	9	83.29998094	77.83142781	-5.468553133	0.02116872	0.06212595
Myl6b	5	3.217391799	1.091123449	-2.12626835	0.02123698	0.06231382
Praf2	5	16.95023148	21.79621721	4.845985724	0.02126384	0.062380167
Mael	43	95.01616448	94.71368579	-0.302478692	0.02139025	0.062738459
Mpc2	53	1.644053504	1.133062753	-0.510990751	0.02146294	0.062939092
Gm16740	23	2.086845936	1.250507822	-0.836338115	0.02148881	0.063002367
Bmp6	96	13.16588477	14.20442294	1.038538162	0.02150825	0.063042912
Rnf39	40	2.198770413	1.889104043	-0.30966637	0.02151123	0.063042912
Rcbtb1	101	1.166713115	1.267508639	0.100795524	0.02152104	0.063059046
Axl	12	4.235602224	3.170814331	-1.064787893	0.02160277	0.063285889
Tfb2m	53	1.450029054	1.30621401	-0.143815044	0.02167963	0.063498392
Pecam1	1	69.23076923	50.72463768	-18.50613155	0.02170868	0.063570786
Mir5123	1	26.44628099	15.86538462	-10.58089638	0.02174172	0.063654825
Suv420h2	26	2.407545188	1.310449998	-1.097095191	0.02176437	0.063708426
Epm2a	101	1.436912223	1.125592144	-0.31132008	0.02177373	0.06372311
Pla2g7	11	5.437393392	4.16817951	-1.269213881	0.02183884	0.063900925
Bcar1	121	2.331594595	2.013630482	-0.317964113	0.02184501	0.063906246
Mei1	19	49.83623514	48.5669157	-1.26931944	0.02187831	0.063978141
Phkg2	6	4.60952202	3.369767136	-1.239754885	0.02187446	0.063978141
Sub1	65	1.470080996	1.373961841	-0.096119155	0.02189155	0.064004108
Rcc1	58	1.380648445	1.47037432	0.089725874	0.02198183	0.064255245
Elmo1	63	1.203832712	1.371650577	0.167817864	0.02198711	0.064257875
Tbc1d2b	45	0.790166549	1.241148626	0.450982077	0.02200507	0.064297558
Hist1h3d	6	2.557052302	4.106507372	1.549455069	0.02209145	0.06453712
Icam5	21	2.201038513	1.819846494	-0.38119202	0.02215518	0.064710406
Uck1	14	0.896675689	1.262291748	0.365616059	0.02220892	0.064854462

Wasf3	49	60.89387913	58.42888946	-2.464989676	0.02227223	0.065017293
Rbm28	43	1.79839361	1.588078717	-0.210314893	0.02227354	0.065017293
Cox6c	31	1.569937434	1.251498688	-0.318438746	0.02228597	0.065040637
Stat2	9	0.717287466	2.321670257	1.60438279	0.02235436	0.065227264
Mir149	7	62.11891498	65.02934673	2.910431746	0.02244277	0.065472207
Nnt	29	1.578412608	1.459240303	-0.119172305	0.02255215	0.065778225
Gm11110	5	0.9919089	2.257295174	1.265386274	0.02255957	0.065786799
Cipc	34	1.416370592	0.96562894	-0.450741651	0.02256926	0.065801958
Acer3	72	1.716562454	1.266761808	-0.449800646	0.02262915	0.065963485
›00012K07F	21	27.16523104	29.96428064	2.799049603	0.02264006	0.065982176
Epor	3	35.53970213	27.23558434	-8.304117794	0.02268931	0.066109317
Tbc1d22bos	2	98.69791667	96.70683518	-1.991081483	0.0226972	0.066109317
Gpn1	40	2.125362477	1.682611371	-0.442751106	0.02269657	0.066109317
Eepd1	28	1.259919584	0.887786247	-0.372133336	0.02276447	0.066292094
Skiv2l2	32	1.18908587	1.623685765	0.434599895	0.02279404	0.066365014
Fam150b	30	39.24751565	38.40136436	-0.846151298	0.02284415	0.066497737
Mtap7d3	7	37.57912391	33.30927022	-4.269853688	0.02285143	0.066505722
Qsox1	32	3.045330267	2.379246178	-0.666084089	0.02293113	0.066724439
Nt5dc2	3	94.37465308	90.87681498	-3.4978381	0.02293829	0.066732058
Haus5	16	1.190816604	1.872284169	0.681467564	0.02297944	0.066832214
Prickle1	91	1.25047623	1.035100336	-0.215375894	0.02298183	0.066832214
ı30005L14F	79	1.153026107	1.191476829	0.038450722	0.02299417	0.066854851
Xrcc4	17	1.855077918	1.119046317	-0.736031601	0.02302432	0.066929245
Prkcq	26	46.00402807	42.73954781	-3.264480263	0.02302976	0.066931795
Slc25a23	42	1.906764059	1.536743764	-0.370020295	0.02305904	0.067003647
Rbpj	123	2.708571575	2.857305682	0.148734108	0.02307036	0.067023253
Ap1m1	9	1.804488116	1.088832086	-0.715656029	0.02315565	0.067257717
Smg5	59	2.220004431	1.937065529	-0.282938902	0.0231708	0.067288417
'00029P11F	9	93.81166394	92.47267753	-1.33898641	0.02324171	0.067480982
Tm6sf2	2	8.981289778	4.612650322	-4.368639456	0.0233117	0.067670801
Gpr157	4	2.188843765	0.617283951	-1.571559814	0.0234303	0.068001623
Onecut2	55	2.888063788	2.361819602	-0.526244186	0.02355096	0.068338297
Abhd14a	12	1.111024693	2.108477678	0.997452985	0.02365731	0.068633324
Zfp563	14	2.684445778	2.98803744	0.303591662	0.02366661	0.068646723

210013O21F	8	15.73206174	11.82544569	-3.90661605	0.02367363	0.068653533
Tmem154	8	26.58881416	30.25675042	3.667936265	0.02368398	0.06866699
Psmc5	21	1.790642558	1.395672242	-0.394970316	0.02368763	0.06866699
Hspd1	72	1.647178131	1.103996541	-0.54318159	0.02370742	0.068703082
933421O10F	46	1.121296846	0.958684965	-0.162611881	0.02370945	0.068703082
Lpar1	41	1.468762391	0.977560457	-0.491201935	0.02372476	0.068733878
Pld1	39	1.301593276	0.906579688	-0.395013588	0.0237369	0.068755487
Plgrkt	9	5.415828094	3.92007443	-1.495753663	0.02377806	0.068861106
Pmm1	50	2.405421924	1.940825108	-0.464596817	0.0237961	0.068899767
Abhd14b	9	3.276046182	2.759501195	-0.516544987	0.02388456	0.069142256
Adarb1	82	1.581109746	1.026113435	-0.554996311	0.02394859	0.069313925
Rbm3os	30	15.62447543	18.40462558	2.780150156	0.02398123	0.06939471
Platr10	3	92.83381278	89.1977692	-3.636043579	0.02398741	0.069398914
Gng5	105	1.494742784	1.253328984	-0.241413799	0.02399243	0.069399761
H1f0	123	1.871843479	1.607453179	-0.2643903	0.02401796	0.069459895
Slc37a2	10	1.923472457	1.314094549	-0.609377908	0.024023	0.069460802
Grhpr	15	2.905445813	1.783376479	-1.122069335	0.02407335	0.069592646
Rita1	38	1.645376109	1.237799941	-0.407576167	0.02409211	0.069633185
Phyhip	9	59.98008556	56.54547977	-3.434605793	0.02415164	0.069791498
Batf	3	46.92007797	37.98220827	-8.937869705	0.02415847	0.069797476
Fam109a	46	1.664018485	1.127389332	-0.536629153	0.02419298	0.069883418
Gabpb2	36	1.596294375	1.025573735	-0.57072064	0.02422916	0.069974161
Eid2b	36	5.260077911	3.110595929	-2.149481982	0.02423899	0.069988759
Fam167a	15	1.21048896	0.594983504	-0.615505456	0.02428349	0.07010347
Lix1l	30	1.840511353	1.387329397	-0.453181957	0.02438011	0.070368568
Alk	8	10.80019913	8.50284272	-2.297356407	0.02439688	0.070403115
Sugp2	12	1.126243269	0.59625316	-0.529990108	0.02449385	0.070669043
Josd1	73	1.482634589	1.201734571	-0.280900018	0.02456487	0.07086002
Fer	83	12.60679528	12.19939274	-0.407402537	0.02460386	0.070958537
Jam3	56	44.40286244	41.5982155	-2.804646942	0.02468948	0.071191464
Cttnbp2	53	32.37201281	31.49324149	-0.87877131	0.02469751	0.071200645
Gtf2i	59	1.160918663	1.481297791	0.320379129	0.02471435	0.071235196
Pdss1	49	0.968374888	1.159546535	0.191171647	0.02472655	0.071256356
Polr2i	4	2.440511163	4.080091285	1.639580121	0.02474833	0.071301433

Dhodh	46	1.407800749	1.107460392	-0.300340357	0.02475191	0.071301433
Cd302	21	6.024597455	5.708568222	-0.316029233	0.02476759	0.071318612
Fbxo18	29	1.751256569	1.421257058	-0.329999512	0.02476471	0.071318612
Prss40	1	85.18518519	72.34042553	-12.84475965	0.02477887	0.071337092
Zfp93	1	1.877934272	5.813953488	3.936019216	0.02481452	0.071425696
Gm5126	28	26.79014144	28.70386853	1.913727088	0.02482255	0.071434823
Mycl	62	1.621449025	1.898574448	0.277125423	0.02483227	0.071448777
Ssbp3	98	0.713414354	0.920011221	0.206596867	0.02487267	0.071550986
Gpalpp1	33	1.196629538	1.560228301	0.363598763	0.02490995	0.071644174
Xlr4b	1	68.55345912	79.31034483	10.75688571	0.02496858	0.07178466
Kis2	3	42.85447192	52.9217853	10.06731338	0.02496682	0.07178466
Gpr25	43	5.432086761	8.99445875	3.562371989	0.02497504	0.071789161
Snord23	6	95.55218579	97.64058041	2.088394616	0.02509094	0.072108206
Nphp1	24	1.677699134	1.022554699	-0.655144436	0.02512663	0.072169768
Fam72a	33	1.733723772	1.17777927	-0.555944502	0.02512712	0.072169768
Fam181a	19	44.12623774	47.89672679	3.77048905	0.02512597	0.072169768
Slc16a1	113	1.336064886	1.359061196	0.022996309	0.02514549	0.072208413
Tlk2	28	1.443923307	1.848367054	0.404443747	0.02516943	0.072263014
Ube2k	64	1.677859992	1.362825854	-0.315034138	0.02518781	0.072301619
Cby1	23	1.533199762	1.288719086	-0.244480676	0.02526673	0.072499815
;32404H12F	45	1.705225229	1.399795631	-0.305429598	0.02526248	0.072499815
Rgp1	35	1.206337252	1.281953263	0.07561601	0.02541342	0.072906461
Derl3	6	19.90866841	15.4332583	-4.475410112	0.0254259	0.072928014
Kctd8	28	27.74340865	25.75256854	-1.990840114	0.02543759	0.072947284
Xkr8	9	2.391167232	3.004951594	0.613784362	0.02545455	0.072981648
Nfkb2	73	1.767271425	1.468222411	-0.299049014	0.02546408	0.072994721
Srp9	51	1.762547103	1.235975238	-0.526571865	0.02558471	0.073326169
Plcg2	44	6.152786435	5.927410829	-0.225375606	0.0256438	0.073481175
Cwc22	16	1.718571426	0.909163864	-0.809407561	0.02577221	0.073834713
Sfr1	29	0.765955957	1.14406206	0.378106103	0.02581224	0.073934972
Bpi	5	62.91905194	58.56137327	-4.357678675	0.02587556	0.074101878
Heg1	68	1.171539661	1.262069183	0.090529522	0.02588668	0.074119254
Fam171b	34	2.153423016	1.277704374	-0.875718642	0.0258941	0.074126018
Iqsec1	22	8.244026037	8.008895408	-0.235130629	0.02591244	0.074164059

Chaf1a	43	1.566640823	1.337035689	-0.229605135	0.02593792	0.074215975
Pramel1	19	83.37650909	81.84057165	-1.535937441	0.02594069	0.074215975
33439K11F	7	11.60266945	19.89390411	8.291234651	0.02597635	0.074274532
Aurkaip1	8	1.474271403	3.421399752	1.947128349	0.02597534	0.074274532
Prkra	36	2.022609157	1.642559072	-0.380050085	0.02597004	0.074274532
Wdyhv1	45	1.784700197	1.404986458	-0.379713739	0.02600976	0.074355596
Dnajc3	96	1.019570133	1.233424385	0.213854252	0.02605169	0.074460952
Ghr	17	8.58087364	9.504715603	0.923841963	0.02607168	0.074503582
Mob2	68	1.835430712	1.192396982	-0.64303373	0.02608136	0.074516712
Parp11	32	1.890846096	1.388279603	-0.502566493	0.02610207	0.074546872
Rbm44	7	94.25712537	91.98241105	-2.274714328	0.02610079	0.074546872
Ptprj	92	1.118874831	0.778576006	-0.340298825	0.02611139	0.074558966
Sash1	79	1.394774036	1.123712485	-0.271061551	0.0262167	0.074845106
Tmem57	145	1.336847999	1.174753244	-0.162094755	0.0262775	0.075004078
Il6st	103	1.437539127	1.514803943	0.077264816	0.0263316	0.075143899
Pias1	49	1.78086314	1.942476917	0.161613777	0.02636458	0.075223393
Crkl	50	0.936286521	1.285309035	0.349022515	0.02638926	0.075279167
Mpc1	78	1.414443871	1.225464869	-0.188979001	0.02645726	0.075458465
Hmx3	103	28.68920371	29.16114174	0.471938032	0.02648721	0.075529211
Nap1l5	17	85.01438054	86.43125207	1.416871526	0.02662078	0.075895342
Tomm70a	58	1.463225782	1.173714179	-0.289511603	0.026626	0.075895487
Heatr5b	33	1.284279886	0.953968774	-0.330311112	0.02668803	0.076057511
Dnajc8	52	1.74839272	1.223940853	-0.524451867	0.02675446	0.076232034
30006K11F	33	2.991530157	2.186162034	-0.805368123	0.02677457	0.076274503
Emx2os	10	45.35960648	49.08816583	3.728559349	0.02678053	0.076276696
Ciita	5	79.71484011	75.50527733	-4.209562782	0.0267977	0.076310786
Ncaph2	33	1.906890409	1.456746644	-0.450143765	0.02686599	0.076490409
Zdhhc9	11	7.329913033	5.917929743	-1.41198329	0.02688465	0.076522868
Dyrk2	153	1.442723612	1.264574453	-0.178149159	0.02688782	0.076522868
Lims1	77	1.309849986	0.992117852	-0.317732134	0.0269743	0.076754088
Cgrrf1	26	2.198399216	1.520562394	-0.677836822	0.0270259	0.076886025
Atn1	136	1.467234666	1.304444102	-0.162790564	0.02703116	0.076886076
Bahd1	150	1.583806492	1.263421415	-0.320385076	0.02705534	0.076939941
Mtap	24	1.370592496	1.887084344	0.516491848	0.02707706	0.076971868

Klhl25	40	1.97908216	1.347113878	-0.631968281	0.02707306	0.076971868
Acot9	19	18.48188535	16.59351052	-1.888374829	0.02718108	0.077230934
Rab10os	32	4.535737093	5.327489043	0.79175195	0.02718248	0.077230934
Ube3a	38	0.812192236	0.43966569	-0.372526546	0.02718398	0.077230934
Limk1	79	4.282829116	3.184763815	-1.098065301	0.02724566	0.077391163
Spr	25	0.850956326	1.465948519	0.614992192	0.0272706	0.077447032
Col14a1	2	10.51441195	15.73597181	5.221559858	0.02729794	0.077498152
Alox15	10	36.73741245	34.14482732	-2.592585123	0.02730445	0.077498152
Abcg3	1	100	95.90163934	-4.098360656	0.02730198	0.077498152
Tinagl1	6	15.08129037	11.07004945	-4.011240918	0.02739367	0.077736362
Gm16982	4	97.54901961	93.67088608	-3.878133532	0.02743003	0.077824501
Rhoh	1	55.6561086	45.77464789	-9.88146071	0.02744293	0.077830995
Tha1	32	2.083859432	1.917444012	-0.16641542	0.02743874	0.077830995
Csnk1e	86	1.553372084	1.23854917	-0.314822915	0.02749818	0.077968927
Asb13	29	2.178426708	2.416175499	0.237748791	0.02750219	0.077968927
Acpt	8	96.91203863	95.73781366	-1.174224978	0.0275089	0.077972888
Gm6213	2	35.50373768	45.52845528	10.0247176	0.0275263	0.07800714
Serpini1	7	2.372450191	4.092471377	1.720021187	0.02756651	0.078106008
Cdh13	6	47.6342519	50.65341348	3.01916158	0.02759382	0.078168297
Rab11fip1	46	1.591280147	1.133110409	-0.458169738	0.02768206	0.07840311
St3gal4	14	1.62037944	0.614254874	-1.006124565	0.02774615	0.078569476
Gpr27	179	14.80381622	15.14357373	0.339757508	0.02782475	0.078776833
Mrpl37	42	1.407940683	1.278901636	-0.129039048	0.02784884	0.078814644
Il15ra	35	2.655717944	1.94238907	-0.713328874	0.02784759	0.078814644
110059E24F	48	1.375122272	0.956888502	-0.41823377	0.0279004	0.078945329
Ap4m1	7	0.876449753	2.018543897	1.142094144	0.02792007	0.078985753
Ntsr1	20	25.79836942	29.35978152	3.561412108	0.02793423	0.078995342
Klhl7	19	1.402302449	0.995133349	-0.4071691	0.02793384	0.078995342
Il4ra	12	1.959177857	1.204401525	-0.754776332	0.02802431	0.079219547
Acot1	1	55.9602649	64.26666667	8.306401766	0.02802331	0.079219547
Arl14	1	41.86046512	26.53061224	-15.32985287	0.02803678	0.079239534
Csnk1g3	67	1.222984671	1.237002053	0.014017382	0.02806209	0.079295795
Mir6970	5	93.54835068	95.7323823	2.184031615	0.02811228	0.079422334
Tmem25	48	4.830727687	4.180966724	-0.649760963	0.02815624	0.079531223

Mrpl1	8	0.681344628	1.679234448	0.99788982	0.02818044	0.079584259
Hist1h2ba	3	98.01561378	96.29974007	-1.715873712	0.0282315	0.079713115
Gzma	3	81.11111111	71.14427861	-9.966832504	0.02826618	0.079795666
Cnot11	58	1.00832656	1.167263304	0.158936744	0.02831247	0.079895384
Slc4a4	91	2.028800991	1.604123217	-0.424677774	0.02831783	0.079895384
Kiz	7	2.06321448	1.331296377	-0.731918102	0.02831141	0.079895384
Smarca5	31	5.945717263	6.422800867	0.477083604	0.02843078	0.080198619
Pde4dip	27	4.329910366	3.96284	-0.367070366	0.02845092	0.080240016
H2afy2	66	15.52053115	16.51364718	0.993116029	0.02849843	0.080358568
310021B22R	37	1.57222695	1.26815972	-0.30406723	0.02854157	0.080456836
Fam134a	50	1.451218965	0.846891072	-0.604327893	0.02854425	0.080456836
Etaa1	38	1.345194374	1.609518303	0.264323929	0.02859584	0.080586774
Ctu1	11	42.81091732	44.94279509	2.131877769	0.0286055	0.080595277
Cars2	48	1.214315408	1.420251356	0.205935948	0.02860984	0.080595277
Cdip1	21	0.991119969	0.675004708	-0.316115261	0.02865531	0.080679317
Rab4b	19	2.054299144	1.273428245	-0.780870899	0.02865617	0.080679317
Mcm2	20	2.747334615	2.031189475	-0.71614514	0.02864916	0.080679317
Slc25a19	3	3.842215133	0.83585423	-3.006360903	0.02871599	0.080832231
Sirt1	131	1.500368017	0.968999014	-0.531369003	0.02876002	0.080925137
Nedd4l	90	1.438350281	1.611811741	0.17346146	0.02875828	0.080925137
Cdx4	6	64.91865968	70.88778632	5.969126634	0.02880201	0.08102774
Slc18b1	20	1.807609382	1.298199339	-0.509410042	0.02881073	0.081036734
Taf10	44	1.103488604	1.009180716	-0.094307888	0.02881651	0.081037453
Sh2b1	24	1.247221373	1.741302966	0.494081593	0.02886306	0.081142716
Abca4	8	78.0694583	72.71743329	-5.352025009	0.028865	0.081142716
Fbxl13	12	2.026818216	3.898356353	1.871538137	0.0288838	0.081180026
Chrna3	16	33.08034523	36.32864939	3.248304161	0.0289058	0.081226289
Rala	82	0.907727578	1.059108083	0.151380505	0.02892664	0.081269306
Commd9	5	0.666215301	2.654953289	1.988737987	0.02906773	0.081650062
Wdr34	19	11.9258918	9.718761896	-2.207129905	0.02913504	0.08180781
Nelfe	60	0.989669012	1.142447795	0.152778783	0.02913324	0.08180781
Slc1a1	47	8.927109822	10.06030097	1.133191152	0.02914781	0.081827997
Wnk4	30	2.845718609	2.333765959	-0.51195265	0.02918322	0.08191174
Acer2	21	5.751169406	4.069460057	-1.681709348	0.02923685	0.082015209

Gpr101	9	47.53019663	51.28600108	3.755804445	0.029233	0.082015209
Sema4c	129	1.4855088	1.604160891	0.118652092	0.02922741	0.082015209
Plod3	64	1.56644236	1.166903676	-0.399538684	0.02925168	0.082041119
Pcm1	28	1.082208165	0.664556529	-0.417651636	0.02930071	0.082162921
Trim45	41	1.681499583	1.378553081	-0.302946503	0.02937813	0.082364288
Arrdc5	3	95.73117995	98.72286079	2.991680839	0.02942459	0.082478786
Spag1	9	1.080605383	0.116346713	-0.96425867	0.02943776	0.082499929
Nfe2l1	45	4.23367818	3.658574724	-0.575103456	0.02946102	0.082549352
Pdlim5	82	1.54275617	1.682307539	0.139551369	0.02958732	0.082887427
Zfp276	57	0.988871591	1.351391339	0.362519748	0.02964924	0.083045031
Syne4	9	87.74647946	84.91039212	-2.836087347	0.02966749	0.083080292
BC022687	35	5.050452125	3.689770819	-1.360681306	0.02967953	0.083098166
Ctgf	12	2.472779695	1.082395689	-1.390384006	0.02968707	0.083103394
Tspan18	82	3.173327834	2.730008586	-0.443319248	0.02987962	0.083610523
Mir25	6	52.25940711	48.82780775	-3.43159936	0.0298791	0.083610523
Rnf185	8	17.31040992	13.81247224	-3.497937683	0.02993585	0.083751904
Oxa1l	20	1.848878827	0.902915676	-0.94596315	0.02995035	0.083776488
Ttc38	18	5.598591729	4.75184444	-0.846747289	0.0299746	0.083827171
Col27a1	65	1.794663828	1.28367593	-0.510987898	0.0299799	0.083827171
Mir3103	1	78.53881279	70.06802721	-8.470785575	0.03000982	0.083894852
Snrpg	5	3.446461397	1.780101752	-1.666359644	0.03005574	0.084007237
Dclk2	65	8.42025784	8.142906772	-0.277351068	0.03010056	0.084116488
H2-Q1	40	21.47370419	21.38302446	-0.090679728	0.03011535	0.084141777
Chac1	18	4.397048851	2.774107038	-1.622941813	0.03012573	0.084154763
Plxdc2	25	23.97817837	22.03490078	-1.943277594	0.03014504	0.084192664
Cep131	70	2.062462759	1.474070375	-0.588392383	0.03016826	0.084241506
Fat4	73	36.80393237	37.97419081	1.170258437	0.0302204	0.084371038
Vps36	42	1.047070758	1.343448216	0.296377458	0.03024734	0.084430185
Chd3	22	1.194812193	0.73647651	-0.458335683	0.03038577	0.084800467
Supt7l	1	5.154639175	10.90909091	5.754451734	0.03042948	0.0849063
Slc35a3	62	1.139227295	1.071244543	-0.067982752	0.03044039	0.084920596
Mir5134	3	97.03407344	98.58438457	1.550311134	0.03046073	0.08496118
Nov	19	23.82190764	27.72357094	3.901663293	0.03047601	0.08498763
Mllt10	64	1.299946279	1.529639423	0.229693144	0.03052095	0.085096789

Zfpm1	115	1.034431876	0.881198582	-0.153233294	0.03058185	0.085250399
Cln8	7	3.191701156	2.264721794	-0.926979362	0.03061594	0.085329225
Tcaim	23	0.888144412	1.424991763	0.536847351	0.03068705	0.085511149
00092M07I	1	98.96907216	96.81372549	-2.155346675	0.03070187	0.085536213
Atp5l	23	0.799431065	1.826742922	1.027311857	0.03071872	0.085566906
Syt8	2	47.00876306	38.45500572	-8.553757344	0.03072558	0.085569791
Nipbl	104	1.495130776	1.17917343	-0.315957347	0.03076911	0.085674739
Dph6	21	0.728927822	0.996227876	0.267300054	0.03084577	0.085871906
Gm5129	42	1.713033295	1.194782901	-0.518250394	0.03088799	0.085973138
Rnaseh2b	32	1.693076196	1.202503965	-0.490572231	0.03091966	0.086044953
Mir6896	3	71.87106727	79.09421358	7.223146309	0.03093264	0.086048444
Alyref2	27	1.731952464	1.354069896	-0.377882568	0.0309302	0.086048444
Mir210	33	6.782133182	5.742575419	-1.039557763	0.0309728	0.086143839
Mir7036	1	100	96.22641509	-3.773584906	0.03103211	0.086290669
Faf2	12	1.434729948	2.505042833	1.070312885	0.03103736	0.086290669
Gpr137b-ps	11	1.747810839	1.054702633	-0.693108206	0.03115431	0.086599427
Rsph6a	6	73.22185061	77.70317502	4.48132441	0.03126604	0.086893539
Nudt3	135	1.462851325	1.124252889	-0.338598437	0.03136009	0.087138408
Mir7685	3	87.61044453	83.21223802	-4.398206509	0.03140034	0.087200708
Jmy	77	1.235563272	1.501235056	0.265671784	0.03139521	0.087200708
Minos1	9	2.447546118	2.286016981	-0.161529137	0.03138973	0.087200708
Myo1a	2	90.48566376	85.18046859	-5.305195176	0.03140699	0.087202689
Slc24a1	4	94.96014031	92.14196468	-2.818175624	0.03143212	0.087222945
Tef	41	1.03393606	1.626273966	0.592337906	0.03142876	0.087222945
Amt	1	87.17948718	74.19354839	-12.98593879	0.03142497	0.087222945
)30625G05F	2	60.14908257	53.21247587	-6.9366067	0.03146796	0.087301501
Cnot4	36	2.291868864	1.391662364	-0.9002065	0.03147233	0.087301501
430429K09F	42	2.114047293	1.402548415	-0.711498879	0.03149734	0.087354369
Ttc14	95	14.69116328	14.39117333	-0.299989946	0.03153644	0.087446291
Tirap	33	1.64068977	1.064326608	-0.576363162	0.03155394	0.087478267
Scarna2	13	1.113635467	0.619269512	-0.494365956	0.03173487	0.087963268
Nwd1	3	6.436731956	4.606393799	-1.830338157	0.03179226	0.088105683
Rgs17	28	5.163398596	4.496503799	-0.666894798	0.03180115	0.088113697
Mir124a-3	139	9.231572332	10.42931953	1.197747196	0.03191705	0.088418109

Pafah1b3	26	1.630553103	0.83103289	-0.799520213	0.03196494	0.08853408
Rpa3	15	1.353901268	1.58392764	0.230026372	0.03203551	0.088712787
Maml3	19	1.392322821	1.277074738	-0.115248083	0.03205771	0.088757511
Dchs1	51	38.89498223	40.45390713	1.558924905	0.03207252	0.08878176
Zdhhc7	92	1.192842202	1.399501297	0.206659095	0.03209472	0.088826471
Nr3c2	36	2.457732301	1.991574191	-0.46615811	0.03212839	0.088902871
Xlr5c	1	92.57142857	85.9030837	-6.668344871	0.0321791	0.089026424
Mcoln1	15	1.100757038	0.530603367	-0.570153671	0.03221772	0.089116465
Fstl5	16	36.82445643	36.29687742	-0.527579012	0.03222399	0.089117017
Fbxo3	34	1.858840693	1.468591205	-0.390249488	0.03232969	0.089392153
Eif2ak4	21	1.598658339	2.00058441	0.40192607	0.03233566	0.089392153
9-Mar	86	1.636085884	1.364299848	-0.271786037	0.03241273	0.089588309
Coro2a	69	1.458835466	1.147011111	-0.311824355	0.03243175	0.089624
H6pd	56	5.558897199	6.711455262	1.152558062	0.03243897	0.089627094
Mbtps2	29	21.274132	19.68508382	-1.58904818	0.03247833	0.08971893
Zbtb24	27	3.282754755	2.240241095	-1.04251366	0.03252664	0.089835469
Parva	7	3.54540382	2.170408692	-1.374995128	0.03253333	0.089837054
Ranbp17	110	55.5726396	56.67316515	1.100525553	0.03257228	0.089927672
Stx2	28	1.560940699	1.396117786	-0.164822913	0.03258616	0.089932152
Cxx1c	14	28.963029	27.13537025	-1.827658748	0.03258463	0.089932152
Kmt2c	190	1.240091026	1.017699568	-0.222391459	0.03260436	0.089965477
Mycn	30	3.671547415	3.177404951	-0.494142463	0.03265516	0.090073721
Arid5a	17	0.593094701	0.859733757	0.266639056	0.03265587	0.090073721
Fam102a	87	1.459597872	1.282464074	-0.177133798	0.0326684	0.090091368
Gdf11	88	1.797651653	1.472637009	-0.325014644	0.0326819	0.090111647
Macrod2	74	1.694169131	1.461432598	-0.232736533	0.03269555	0.090132357
Hn1l	16	1.398486297	1.940610553	0.542124257	0.03274193	0.090243262
Dpyd	10	12.18967511	11.80819615	-0.381478969	0.03275468	0.090261454
Gsg2	74	1.126753586	1.289466317	0.162712731	0.03282058	0.090426072
Ppap2b	30	1.566768057	1.297322104	-0.269445953	0.03285845	0.090513399
Cdyl	166	1.942593883	1.695319287	-0.247274597	0.03293968	0.090720142
Fam209	1	92.11618257	86.07594937	-6.040233206	0.03304479	0.090992548
Rela	109	1.899414544	1.31358202	-0.585832524	0.03305748	0.091008504
Tex19.1	11	95.28812529	92.88807239	-2.400052902	0.03306299	0.091008504

Gnaz	69	1.93282844	2.436454239	0.503625799	0.03308117	0.091041475
Hist2h3c1	7	3.438371602	2.745144834	-0.693226768	0.03311489	0.091117165
Armc5	43	5.731793628	7.407741707	1.675948078	0.03315634	0.091214124
Aaed1	9	0.113378685	0.906148795	0.79277011	0.03325859	0.091478258
Bhmt2	1	91.52542373	78.88888889	-12.63653484	0.03331142	0.091606407
Sema3e	14	4.785087699	3.693775101	-1.091312598	0.03343066	0.091917076
Rnf207	14	6.419522282	4.794971605	-1.624550678	0.03344026	0.091926258
C1galt1	117	1.101317561	1.128734804	0.027417242	0.03350572	0.092088947
Galnt3	97	1.827747263	1.495115548	-0.332631716	0.0335419	0.092171127
Pcdh17	2	14.3442623	7.916666667	-6.427595628	0.03360935	0.092339195
Dcun1d4	49	1.601056179	1.273458306	-0.327597873	0.03372477	0.092621613
Zfp623	56	1.298532232	1.034087746	-0.264444486	0.03372259	0.092621613
Pklr	8	16.25409227	20.63778429	4.383692019	0.03376039	0.092702078
Brat1	14	2.371732559	1.22553633	-1.14619623	0.03378475	0.09275161
Eef1d	74	1.198128657	1.339390728	0.141262071	0.0338748	0.092981452
Pcdh12	1	83.87096774	74.44444444	-9.426523297	0.03392742	0.093108458
Sec22a	45	1.380240096	1.04356774	-0.336672356	0.03394673	0.093144028
Vrtn	10	54.40054921	51.85617128	-2.544377931	0.03402086	0.093312549
Heatr1	12	1.841835378	0.734987928	-1.10684745	0.03401966	0.093312549
Metap1	78	1.502748141	1.110689585	-0.392058556	0.03404911	0.093355137
Pradc1	81	0.972955804	1.272653393	0.299697589	0.03404302	0.093355137
Magee1	6	25.95839618	21.47092542	-4.487470756	0.03409658	0.09345058
Dgke	49	1.159700711	1.346660251	0.18695954	0.03409913	0.09345058
Dcbld2	93	1.130217686	1.447271169	0.317053482	0.03410303	0.09345058
Map1lc3b	33	1.65097958	1.359415055	-0.291564525	0.03412021	0.093480207
Zfp324	13	1.213447761	1.886001805	0.672554043	0.03415542	0.093559209
Phka1	36	16.09446335	14.09509892	-1.999364434	0.03420714	0.093650711
Tfap2a	55	33.2954958	34.21348609	0.917990294	0.03420797	0.093650711
Tcirg1	31	1.656989594	1.278833024	-0.378156571	0.03420417	0.093650711
Cnn2	52	1.353807242	1.597469782	0.24366254	0.03422539	0.093663443
330050I16R	70	1.055080791	0.963825888	-0.091254903	0.03422112	0.093663443
Ppm1j	51	3.546423875	2.695892768	-0.850531107	0.03425213	0.093719135
Pgr	2	39.36170213	33.38235294	-5.979349186	0.03439019	0.094079354
Cbx4	109	1.665996643	1.369473195	-0.296523448	0.03441937	0.094141629

Dnah7a	20	73.60310147	75.65579956	2.052698085	0.03445371	0.094217985
Onecut3	43	2.017100012	1.806587637	-0.210512375	0.03446869	0.094241383
Zfp326	58	1.552047978	1.283752221	-0.268295757	0.03453502	0.094405166
Hnrnpa0	153	1.157496164	0.944608669	-0.212887495	0.03458247	0.094517258
2-Sep	23	1.485657689	1.133573767	-0.352083922	0.03459094	0.094522807
Figla	2	95.36934185	97.6744186	2.305076755	0.03461791	0.094578899
Scnn1a	3	52.33897894	46.96742308	-5.371555853	0.03464968	0.094648058
Cxx1a	3	27.87736311	20.72169312	-7.155669989	0.03469103	0.094743369
Kif24	11	0.68864757	1.165686758	0.477039188	0.03473771	0.094853219
Rft1	20	1.716761934	1.48671797	-0.230043964	0.0348778	0.09521801
Lrrc73	55	4.091234463	3.575135542	-0.516098921	0.03495331	0.095406404
Exosc1	42	1.493160779	1.22257505	-0.270585729	0.03498184	0.09546653
Klrg2	54	1.536729951	1.190790043	-0.345939908	0.03502475	0.095565844
Zdhhc5	40	1.363391513	0.765975484	-0.597416029	0.03503847	0.095567742
Pde12	23	1.090700847	1.764292055	0.673591208	0.03503522	0.095567742
Faap24	5	0.939982348	2.071926303	1.131943955	0.03504585	0.095570111
Fkbp10	19	4.011856553	5.233025058	1.221168505	0.03520095	0.095975227
Gm10658	3	5.055296489	3.458290831	-1.597005658	0.03525396	0.096101914
Gdpd2	5	40.13944343	33.11667634	-7.022767094	0.03526308	0.096108912
Mir7046	4	94.95015488	92.95619937	-1.993955506	0.03532595	0.096262401
Srek1ip1	7	1.137602638	0.536616162	-0.600986476	0.03533671	0.096273827
B4galt2	73	2.127672015	1.726495126	-0.401176889	0.03536763	0.096340185
Hibadh	34	0.767573098	1.356568981	0.588995883	0.03541938	0.096463235
Qrfp	3	14.97560976	9.68707483	-5.288534926	0.03553753	0.096767069
Limd1	65	2.837038892	2.1839037	-0.653135191	0.03554536	0.096770433
Hid1	34	1.838611026	2.125806746	0.28719572	0.03557442	0.096831584
Stx16	25	2.049996356	1.647308063	-0.402688293	0.03567537	0.09708834
Amz2	10	1.481153953	2.308241312	0.827087359	0.03584689	0.097537049
Smarcd3	46	1.68989213	1.322317579	-0.367574551	0.03587931	0.09760715
Scrn2	13	3.058045382	2.266626666	-0.791418717	0.03600252	0.097924197
Ptpn4	24	1.182069012	1.182904941	0.000835929	0.03605154	0.098039345
Caap1	63	0.705803427	1.076039841	0.370236414	0.03616409	0.098327183
Cfl2	90	1.281753554	1.155083057	-0.126670496	0.03618102	0.098355
Cnih4	42	1.160087933	0.83991035	-0.320177583	0.03619479	0.098374207

Pla2g12a	70	0.968795771	1.206514865	0.237719094	0.03626105	0.098536049
Park7	21	1.415046398	0.911111219	-0.503935179	0.03632915	0.098702831
Adgrl4	5	85.52380952	80.90962412	-4.614185408	0.03639914	0.098874675
510008E11F	7	2.076521216	1.049313793	-1.027207424	0.03643256	0.098928815
Pak4	51	2.158906766	1.923860151	-0.235046615	0.03642993	0.098928815
Aprt	27	1.846138073	1.225123874	-0.621014199	0.03645427	0.098969478
Ifi47	5	9.671503319	6.603088669	-3.06841465	0.03651527	0.099105794
Stard6	61	2.856779183	1.815578682	-1.041200501	0.03651799	0.099105794
Rnf20	13	2.059473726	1.179694971	-0.879778755	0.03657077	0.099222495
700088E04F	37	1.312353034	0.995864431	-0.316488603	0.03657452	0.099222495
Arhgap12	66	1.276132075	1.484716718	0.208584644	0.03661731	0.099320224
Rps6ka4	61	1.309106027	1.338573665	0.029467639	0.03663714	0.099355642
Gm20939	4	0.860297591	1.89341326	1.033115669	0.03667068	0.099428218
Ctu2	79	1.231361089	1.261803828	0.030442739	0.03672616	0.099560254
Adgra2	112	1.306242917	1.265953786	-0.040289131	0.03686506	0.099918332
B4gat1	43	0.942274158	1.211923996	0.269649837	0.03692253	0.100055621
Chic1	2	20.76023392	14.63661552	-6.123618402	0.03696414	0.100149875
Slc22a8	1	66.9527897	57.57575758	-9.377032124	0.03701222	0.10026164
Atg7	12	2.093133495	2.793168026	0.70003453	0.03704233	0.100324694
Rpl26	19	2.754330875	2.309943119	-0.444387756	0.03713163	0.100547997
Ppl	31	2.988914193	2.790673937	-0.198240256	0.0371775	0.10065363
Inadl	38	0.995992041	1.2271427	0.231150659	0.03719183	0.100673849
Rnaseh2c	20	1.533197081	1.186260196	-0.346936885	0.03723643	0.100775972
Popdc2	9	84.46657557	88.20682373	3.740248159	0.0372623	0.100827389
Nacc2	92	2.367001721	1.987499926	-0.379501795	0.03729893	0.100907915
Ndufab1	22	0.720508417	1.127400524	0.406892107	0.03732042	0.10094745
Snord104	18	1.080047353	1.567473043	0.48742569	0.03735142	0.101012655
.10017D15F	8	53.3333295	51.33125531	-2.002074186	0.03744871	0.101257116
Thbs2	2	21.33463691	17.41998927	-3.914647642	0.03750833	0.101399631
Thnsl2	13	4.048283135	2.358644019	-1.689639117	0.0375203	0.101413307
Ccar1	58	1.248793998	1.045686604	-0.203107394	0.03752972	0.101420102
Tead3	80	4.330061008	3.591279057	-0.738781952	0.037547	0.101448101
Sesn3	30	1.237401451	1.615912794	0.378511343	0.03769108	0.101818647
Srrm4	32	50.83291154	47.25799478	-3.574916765	0.03773228	0.101911198

Tmem74b	52	5.832718925	7.441558933	1.608840008	0.03774105	0.10191611
Dleu2	62	1.258641198	0.937717329	-0.320923869	0.03781941	0.10210894
Nup210l	8	91.67617344	90.1562943	-1.519879136	0.03790712	0.102326918
;10040J01R	38	2.403454269	3.184068417	0.780614148	0.03792621	0.102359611
;10042L04F	2	10.66666667	21.34831461	10.68164794	0.03794736	0.102397862
Mir7658	1	95.89041096	100	4.109589041	0.03801607	0.102564396
Insr	39	1.454068097	1.015837036	-0.438231061	0.03803053	0.102584568
Ddx24	19	1.342613213	1.08085281	-0.261760403	0.03803834	0.102586779
110082J24R	17	31.83088186	28.58555414	-3.245327715	0.03823449	0.10309681
Lrrc24	47	6.816583126	6.789482475	-0.027100651	0.03830493	0.103267777
Wwc1	79	1.93000068	1.607337159	-0.32266352	0.03840791	0.103526378
Gm2061	55	31.58469592	32.14869925	0.564003337	0.0384636	0.103638412
Cirbp	46	1.41383659	1.15141621	-0.26242038	0.03846049	0.103638412
Dcun1d1	111	1.02502238	1.025298578	0.000276198	0.03860904	0.104011209
Rnf111	85	1.05145319	1.139116006	0.087662816	0.03869825	0.104232407
Bax	42	1.878551957	1.469598313	-0.408953644	0.03872748	0.104291995
;10010K14F	49	1.406600833	1.054889598	-0.351711236	0.03873661	0.104297427
Nudt8	52	1.907545583	1.705361334	-0.202184249	0.03876069	0.10434311
Gtse1	17	3.122798977	2.290452535	-0.832346442	0.03888889	0.104649831
Ppp2r3c	9	1.834406056	1.155840756	-0.6785653	0.03888826	0.104649831
Pitpnb	66	1.17397111	1.341618242	0.167647132	0.03891954	0.104713112
Ptov1	36	1.112119298	0.583349901	-0.528769397	0.03894147	0.1047529
Scaf4	110	1.768100395	1.295468702	-0.472631693	0.03896214	0.104789295
Rbm22	34	1.236208677	1.825738288	0.58952961	0.03905436	0.105018078
Hexim1	10	1.667679482	2.279422931	0.611743448	0.0390811	0.105070724
Tmed5	40	1.1910632	0.673721928	-0.517341272	0.03910516	0.105110274
Map2k4	87	1.184430189	1.278473335	0.094043146	0.0391173	0.105110274
Ano5	10	52.81860286	50.04351619	-2.775086673	0.03911152	0.105110274
Miat	20	21.09666281	23.95066712	2.854004309	0.03915692	0.105191625
331440E17F	147	1.187531242	0.940410536	-0.247120707	0.03916191	0.105191625
Dram1	17	2.531053995	1.443251963	-1.087802032	0.03920073	0.105276619
Agap2	2	29.92915956	22.9261469	-7.003012661	0.0392321	0.105341586
Gpr89	27	1.931192711	1.314660453	-0.616532258	0.0393066	0.10550414
Igf2bp2	76	1.939618845	1.266683291	-0.672935553	0.03930701	0.10550414

Lect2	2	36.66666667	28.29131653	-8.37535014	0.03933658	0.105548824
Sdccag3	59	1.026801907	1.234351097	0.20754919	0.03933805	0.105548824
Hmgn2	46	4.212736859	6.000568858	1.787831999	0.03942634	0.105748694
Cyp4b1-ps2	1	75.47169811	61.79775281	-13.6739453	0.03942695	0.105748694
Swap70	34	1.431108761	0.909245552	-0.521863209	0.03949665	0.105916266
Ldb1	126	1.230438189	1.347333343	0.116895154	0.03951588	0.10594849
Tbc1d22a	51	1.329381117	1.045921232	-0.283459886	0.03955853	0.106043466
Med17	43	0.96235093	1.296772186	0.334421257	0.03964234	0.106229307
Cdk5rap1	15	1.307486777	0.811387461	-0.496099316	0.03963776	0.106229307
Sos1	121	1.321066397	1.053872647	-0.26719375	0.03966668	0.106275133
Pnkd	55	2.1025637	1.411654728	-0.690908972	0.03972809	0.106400802
Txnrd3	73	1.533210759	1.308100591	-0.225110169	0.03972783	0.106400802
Mamdc2	5	4.469379461	3.884419298	-0.584960163	0.03977906	0.106516958
Kif11	29	1.367834335	1.162989344	-0.204844991	0.03978598	0.106516958
Sestd1	101	1.243551109	1.099166307	-0.144384802	0.03986536	0.106710003
Acsl6	48	9.746365914	9.764954941	0.018589027	0.0400517	0.107189241
Nr2f6	158	1.278898077	1.110822794	-0.168075283	0.04008248	0.107252065
Rap2b	47	2.103440751	1.646630231	-0.456810519	0.0402464	0.107671041
Mir702	10	96.85164349	94.54987482	-2.301768671	0.04027292	0.107722355
P3h4	59	2.524516382	2.023238853	-0.501277529	0.04028409	0.107732604
Irf2bp2	170	0.936310052	0.976091922	0.039781869	0.04030776	0.107776262
Tecr	9	1.499887239	0.631894481	-0.867992758	0.04035942	0.107894712
Phactr3	29	50.05666774	53.43488818	3.378220439	0.04040407	0.107994399
Olfr1033	7	40.35069745	35.65173944	-4.698958015	0.04041549	0.108005269
Psmd1	42	3.553311227	3.76469518	0.211383953	0.0404355	0.10803906
Mtf2	34	0.885688546	1.236631867	0.35094332	0.04047528	0.108125663
Ndufs2	22	0.994691762	1.810645903	0.815954141	0.04052042	0.10822654
Snx27	63	1.625114265	1.433586801	-0.191527464	0.04067234	0.108612548
Gm20110	1	100	94.54545455	-5.454545455	0.04072439	0.108731747
Traf3	94	1.11068362	1.132185283	0.021501663	0.04075272	0.108787609
Sp9	41	32.80204242	34.84685121	2.04480879	0.04085966	0.109053219
Osbpl8	91	1.266928325	1.095755112	-0.171173213	0.04088145	0.109091534
Kctd2	71	1.478201926	1.605095647	0.126893721	0.04090056	0.109122688
Tmed1	10	0.825710232	1.390382986	0.564672754	0.04095778	0.10925549

Slc2a8	67	1.794461729	1.410826423	-0.383635305	0.04112395	0.109658877
Htr4	20	32.59986852	32.18950172	-0.41036681	0.0411178	0.109658877
Usp26	13	96.15898491	94.32514935	-1.833835557	0.04115351	0.109717759
Cltc	26	2.71560593	2.609218626	-0.106387303	0.04116404	0.109725914
Gm8773	11	17.0640625	14.71989686	-2.344165635	0.04119433	0.109786692
Acox3	32	1.798155117	1.340847681	-0.457307436	0.04124924	0.109873167
Myo1c	81	2.01288795	1.636951911	-0.375936039	0.04124448	0.109873167
Syna	3	44.87399686	39.66590302	-5.20809384	0.04124814	0.109873167
Aff4	134	1.183837542	0.856954704	-0.326882838	0.04129219	0.109967625
Scfd2	25	2.332527784	1.848550248	-0.483977536	0.0413153	0.11000921
Btbd7	109	1.144456884	1.196116482	0.051659598	0.04134914	0.110079325
Fam76a	92	1.008787975	1.100748668	0.091960693	0.04139687	0.110175795
Pex14	12	2.322249588	1.665231344	-0.657018243	0.04140355	0.110175795
Nr2c2ap	23	1.141494848	1.493810137	0.352315288	0.0414079	0.110175795
Ubxn7	38	1.571645417	1.423755475	-0.147889941	0.04142913	0.11018804
Mycbp	78	1.296642343	1.085889981	-0.210752362	0.04143503	0.11018804
Dusp28	17	1.215255333	2.282999923	1.06774459	0.04142493	0.11018804
)30028A08F	6	3.332667542	2.067868948	-1.264798594	0.04151987	0.110393658
Mir6995	3	95.86206897	92.46498599	-3.397082971	0.04165073	0.110721533
Igtp	8	52.23820752	48.58821459	-3.649992937	0.041789	0.11106896
Snord32a	3	24.9382716	18.51404976	-6.424221841	0.04190972	0.111349482
Mir5121	3	24.9382716	18.51404976	-6.424221841	0.04190972	0.111349482
Prom1	18	39.56058853	39.97946317	0.418874642	0.04197839	0.111511744
Gpr50	8	67.32236195	63.09774955	-4.224612405	0.04200025	0.111549615
Cep72	23	2.323443846	1.436180773	-0.887263073	0.042014	0.111565946
Zfp558	6	1.667927219	0.902123823	-0.765803397	0.04202343	0.111570788
Pcdha8	3	78.04710072	71.86296092	-6.184139794	0.04204186	0.111599537
Aimp1	15	1.811183592	1.335722538	-0.475461054	0.0420744	0.111645516
Ebpl	12	2.47545096	1.145776144	-1.329674816	0.04206737	0.111645516
Cystm1	49	1.531010472	1.225085953	-0.305924519	0.0421253	0.111719976
Fmnl3	78	1.431149969	1.526297166	0.095147197	0.04212372	0.111719976
Sgpp1	84	1.307004651	0.830265169	-0.476739482	0.04211159	0.111719976
Twistnb	46	1.208863527	0.852794159	-0.356069368	0.04218238	0.111851139
Nfe2l2	18	3.007053746	2.109750364	-0.897303383	0.04220918	0.111901996

Usp45	20	2.204968819	1.24233041	-0.962638409	0.04222481	0.111902985
Slc35d1	139	1.215542562	1.320252817	0.104710256	0.0422189	0.111902985
Qdpr	42	1.276869221	1.182008792	-0.094860429	0.04229923	0.112079973
Dsp	64	1.250542374	0.817519547	-0.433022827	0.04230809	0.112083216
Gprc5b	29	3.037721894	2.241141152	-0.796580742	0.04233631	0.112103792
Mir3107	7	79.83845285	75.83964726	-3.998805594	0.04233657	0.112103792
Gm7904	6	95.6506368	94.22012209	-1.430514716	0.04233878	0.112103792
Tmem198b	15	1.120365673	0.862457646	-0.257908027	0.04247147	0.11241918
Tex14	6	98.37289331	96.38314754	-1.989745771	0.04247321	0.11241918
Nelfb	28	1.320643187	0.721514778	-0.599128408	0.04252833	0.112544765
Tmem161a	3	89.6561868	91.39515455	1.738967754	0.04255011	0.112582086
Trabd2b	77	8.107355563	6.886150411	-1.221205152	0.04259682	0.112685373
Fam193a	137	1.168426335	1.307494758	0.139068423	0.04272017	0.112991306
Stk4	27	2.111326053	1.629342085	-0.481983967	0.04274407	0.113034158
Stim2	78	2.573446078	1.630481196	-0.942964881	0.04278501	0.113122031
Ube2b	71	1.499063573	1.156959938	-0.342103636	0.04282788	0.113214978
Csnk2b	59	1.70157047	1.141272504	-0.560297966	0.04287532	0.113279942
Clic4	67	1.166695713	1.204978573	0.03828286	0.04287562	0.113279942
Fundc2	4	11.66666667	16.46870287	4.802036199	0.04286495	0.113279942
Lrrfip2	85	1.19872828	0.917224847	-0.281503434	0.0429085	0.113346413
Btg2	60	1.463035566	1.689606249	0.226570683	0.0429599	0.113461762
Ctbs	6	5.05981161	3.349060577	-1.710751033	0.04300799	0.113568334
Trappc10	57	1.67949034	1.339133172	-0.340357168	0.0430293	0.113604157
Ciart	40	3.120636425	2.433207328	-0.687429096	0.04311293	0.11380449
Ccnb1	1	3.484320557	7.189542484	3.705221926	0.04312834	0.113824674
Cisd2	90	1.047159716	1.036720263	-0.010439453	0.04317611	0.113930282
Xlr3b	4	79.2759324	72.44824196	-6.827690439	0.04329759	0.114230285
Exph5	6	9.021495974	11.15313858	2.131642604	0.04330868	0.114234218
AA387883	5	45.36376625	49.09894927	3.735183013	0.04331465	0.114234218
Plxna4	74	1.621271487	1.142841634	-0.478429853	0.04334363	0.114290098
Zmpste24	20	2.442587964	2.030855617	-0.411732347	0.04336578	0.114312891
Rimbp2	1	17.77777778	26.06837607	8.290598291	0.04336785	0.114312891
Eif3c	42	1.290450556	0.931007603	-0.359442954	0.04341768	0.114403123
Psrc1	5	1.639708606	0.663091483	-0.976617123	0.04341404	0.114403123

Atp13a2	54	1.302455524	1.360683745	0.058228221	0.04343779	0.114435573
Tnpo2	23	2.276939045	1.663273999	-0.613665046	0.04346147	0.114477411
Synj2	79	2.228206174	1.954393142	-0.273813031	0.0435459	0.114679218
Zbtb37	68	1.643589772	1.857181352	0.213591581	0.0435949	0.114767058
Gas5	68	1.643589772	1.857181352	0.213591581	0.0435949	0.114767058
Vps28	23	1.167653016	1.874268994	0.706615978	0.04364445	0.114876883
Entpd1	10	21.51060424	17.54130606	-3.969298183	0.04368351	0.114959081
Zfp459	2	16.15179252	22.68295693	6.531164405	0.04370558	0.114996545
Gm6588	1	33.51648352	24.28571429	-9.230769231	0.04376942	0.115143854
Iqcb1	12	1.093473999	1.326173259	0.23269926	0.0437854	0.115144619
Stambp	11	1.107668749	1.209106985	0.101438236	0.04378049	0.115144619
Hgs	20	1.397148967	0.899018289	-0.498130677	0.04382354	0.115219621
Tmem82	2	15.74412118	12.89133572	-2.852785464	0.04382963	0.115219621
Pex13	11	1.04923917	1.678986193	0.629747023	0.04386412	0.115289656
Sssca1	5	4.998355241	3.958445587	-1.039909654	0.04387325	0.115292988
Fmnl2	86	1.603249527	1.249427667	-0.353821861	0.04388621	0.115306399
Rere	57	1.849266569	1.578986157	-0.270280412	0.04392164	0.115358187
Atp6v1d	6	0.698564418	1.65369901	0.955134592	0.04391653	0.115358187
Asb7	7	1.667129961	2.355693495	0.688563535	0.04396702	0.11545669
Pkd1l3	3	64.27586207	71.54931991	7.273457838	0.0439933	0.115505052
Fignl2	52	78.9996011	79.34813412	0.348533023	0.04405554	0.115596609
Mir6359	7	2.86841696	1.624138573	-1.244278388	0.04405075	0.115596609
Pold1	15	2.063567717	1.376378533	-0.687189184	0.04403952	0.115596609
Mrpl40	15	1.976791704	1.488645651	-0.488146053	0.04405969	0.115596609
Fbl	33	1.374404696	0.871014314	-0.503390382	0.04412119	0.115737284
Nop9	32	1.52348133	1.007301518	-0.516179812	0.04413466	0.11575193
Braf	53	1.060596159	0.744984584	-0.315611575	0.04414646	0.115762174
Ndor1	18	1.460574819	2.201178875	0.740604056	0.04423696	0.11597876
Tpst1	69	5.655184479	5.583265147	-0.071919333	0.04424671	0.115983594
Atp2c1	75	1.32742288	1.009308777	-0.318114103	0.04425486	0.115984251
Gclm	101	1.009456797	1.154034091	0.144577294	0.04430632	0.116098379
Pole2	30	1.333806296	1.560464858	0.226658563	0.04432397	0.116123883
Scarb2	33	0.741415039	1.358286031	0.616870992	0.04436392	0.116207819
Ect2l	5	6.535392674	4.369755153	-2.165637521	0.04442191	0.116338956

Zbtb2	87	1.267455947	1.337133047	0.069677099	0.04446089	0.116420263
Slc29a4	57	35.86285878	37.16597215	1.303113365	0.04447345	0.116432362
Mir3113	1	88	93.30708661	5.307086614	0.04450908	0.11650485
Tomm22	49	2.014857495	1.285673212	-0.729184283	0.04456557	0.116631918
Rhbdd3	35	1.224948745	1.664532794	0.43958405	0.04463408	0.116790386
Extl3	60	1.659469148	1.303083009	-0.356386138	0.04470533	0.116955974
L3mbtl4	17	56.20763102	54.39684046	-1.810790555	0.04473101	0.117002281
Cabin1	34	1.125490672	1.472371979	0.346881307	0.04476999	0.117083371
Khk	20	2.212957501	2.561877369	0.348919868	0.04483531	0.117233315
Kcnq4	118	6.625244539	7.09859263	0.473348091	0.04484365	0.117234221
Iqch	53	1.397607334	1.578886208	0.181278874	0.04487795	0.117302999
Nfkbil1	3	1.609986691	0.824499411	-0.785487279	0.04488725	0.117306418
Hcfc1	8	17.38047085	14.91225981	-2.468211035	0.04491517	0.11733758
Mast2	117	1.125581379	1.0885279	-0.037053479	0.04491105	0.11733758
Clspn	21	1.95999995	1.266671868	-0.693328082	0.04494787	0.117402101
Gadd45gip1	21	1.559367729	1.963599246	0.404231517	0.04501433	0.117554783
)33407K13F	2	19.40789474	14.06973308	-5.338161653	0.0450878	0.117725696
30016M11F	9	1.753218822	1.242009016	-0.511209805	0.04518624	0.117961719
Capn1	25	13.46348904	12.39736037	-1.066128671	0.04532143	0.118293615
Hnrnph1	28	0.763714209	1.158346497	0.394632288	0.04536457	0.118385149
Papss2	41	1.68601055	1.425624194	-0.260386356	0.04544735	0.118580077
Nsf	59	1.076269364	1.192907376	0.116638012	0.04550883	0.118719388
Slc16a14	29	10.9641244	10.29565538	-0.668469024	0.04552844	0.118743429
Btaf1	100	1.22258996	0.845368204	-0.377221756	0.04553423	0.118743429
Synj2bp	19	2.159522456	2.331765991	0.172243535	0.04566074	0.119031037
ynj2bp-cox1	19	2.159522456	2.331765991	0.172243535	0.04566074	0.119031037
Ryr1	9	34.576149	29.91668291	-4.659466086	0.04572727	0.119149776
Scamp5	41	2.496103467	1.530783921	-0.965319546	0.04571478	0.119149776
Tgfbi	2	4.430379747	0	-4.430379747	0.04573065	0.119149776
Vpreb1	1	99.27272727	97.20670391	-2.066023362	0.04583398	0.11939779
Tmem110	62	1.463388233	1.577708069	0.114319836	0.04587573	0.119485358
BC030336	45	1.001783496	0.89534479	-0.106438706	0.04594326	0.119639991
Phf5a	12	1.828759234	0.691266209	-1.137493025	0.04603995	0.119849261
Zfp697	12	3.200850262	2.026147574	-1.174702688	0.04603886	0.119849261

Rbx1	23	3.20654527	2.801531971	-0.405013299	0.04604871	0.119850806
Nr6a1	40	1.564997189	1.941043222	0.376046032	0.04606391	0.11986908
Eya1	14	3.290957462	2.581123629	-0.709833833	0.04608918	0.119913493
Prrx2	74	1.843228721	1.499629115	-0.343599606	0.04609732	0.119913493
Mettl10	3	3.569226025	0.974534073	-2.594691952	0.04617519	0.120094776
Xpnpep1	54	2.093493332	1.676589304	-0.416904028	0.04620322	0.120146393
Gm10516	40	2.535545427	1.968981955	-0.566563473	0.04622784	0.120189103
Pxmp2	44	1.561007121	1.202180215	-0.358826906	0.04624752	0.120197692
Ttc39b	42	1.585034482	1.22359381	-0.361440672	0.04624006	0.120197692
Slc8a2	10	6.769339617	4.682993778	-2.086345839	0.04625586	0.120198056
Pfn2	113	1.64611885	1.486680095	-0.159438755	0.04640676	0.120568836
Zfr	71	1.488557667	1.199797084	-0.288760582	0.04641644	0.12057265
Etv3	56	0.999167872	1.029661009	0.030493137	0.04646275	0.120671562
Qrich1	37	1.583548742	1.394223713	-0.189325029	0.04651632	0.120789323
Acly	38	2.199364105	2.173770409	-0.025593696	0.04652936	0.120801814
Decr2	15	2.020276219	2.881376295	0.861100076	0.04656945	0.120884507
Map2k3	48	1.602325119	1.288888745	-0.313436374	0.04657872	0.120887175
i00002D24F	1	71.69811321	55.05617978	-16.64193343	0.04662681	0.120990592
Pbdc1	10	2.351403158	3.335702246	0.984299087	0.04668083	0.121109357
Gtf2f2	26	0.812254	1.004320198	0.192066198	0.04669612	0.121127613
Mpzl2	14	2.788559648	2.402345911	-0.386213737	0.0468353	0.12146715
Trpc6	4	25.26441214	32.24301509	6.978602949	0.04686056	0.121511196
Mettl25	39	1.595936235	1.309035944	-0.286900291	0.04687569	0.121528952
Rbm12b1	5	2.152276295	2.440672712	0.288396417	0.04694254	0.121666398
Ass1	17	3.134199935	2.249118356	-0.885081579	0.04694529	0.121666398
Ythdc2	55	1.636454041	1.344317421	-0.29213662	0.04696916	0.121706782
930522O17F	3	70.97273996	65.31058734	-5.662152618	0.04710103	0.122026925
Clip1	69	2.770255087	1.600887707	-1.169367379	0.04725557	0.122384094
Mzt1	134	1.125897334	0.873632814	-0.252264521	0.04725132	0.122384094
C5ar2	1	28.47222222	19.30693069	-9.165291529	0.04736404	0.122643357
Hdgfrp2	26	1.619629295	1.011492327	-0.608136968	0.04743277	0.122799647
Eif2b5	31	1.839898548	1.146214109	-0.693684439	0.04747165	0.122878637
Rftn2	6	58.13952774	64.66364341	6.524115667	0.04753516	0.123021337
Id3	26	1.473491517	1.092807433	-0.380684084	0.04757444	0.123101269

Tubb3	32	3.770129585	2.277477149	-1.492652436	0.04767607	0.123321259
Fxyd2	2	28.39506173	13.85542169	-14.53964004	0.04767626	0.123321259
Orc5	10	0.277777778	2.415019933	2.137242156	0.04785408	0.123759387
Impg1	2	96.58119658	88.32335329	-8.257843288	0.04787246	0.123785118
Fbxo4	43	1.315646099	1.511569554	0.195923455	0.04791114	0.123863293
Fars2	36	1.281905165	1.064364742	-0.217540423	0.04793544	0.123904291
Coa3	7	0.631536906	1.604064716	0.97252781	0.04794864	0.123916587
Aldh1l2	7	9.218908744	13.10568696	3.886778218	0.04796504	0.123937136
Hus1b	5	94.38986341	91.71012078	-2.679742633	0.04798491	0.123966643
Gm5294	10	71.47708035	73.45103516	1.97395481	0.04799487	0.123970562
Raver1	32	1.871709938	1.711528995	-0.160180943	0.04801216	0.123993389
Apln	25	18.20992548	17.00617182	-1.203753663	0.04803743	0.124036819
Arl6ip4	39	2.024412972	1.03704764	-0.987365331	0.04807116	0.124102066
Taok2	10	1.523376623	1.875907782	0.352531159	0.048102	0.124159853
Flcn	27	0.61407072	0.89829461	0.28422389	0.04812346	0.124171553
Ndufa12	37	1.768518701	1.344264598	-0.424254103	0.04811952	0.124171553
Eef1b2	45	1.874433388	1.650593304	-0.223840084	0.04817355	0.124278954
Fbxo42	77	1.175681858	0.853082899	-0.322598959	0.04819178	0.124304138
Gas2l3	19	2.781755925	2.11826965	-0.663486275	0.04820088	0.124305755
Kras	129	1.13234769	0.900644513	-0.231703176	0.04821899	0.12433059
Ano2	4	20.96685082	17.98140588	-2.985444946	0.04826526	0.124428049
Gpr75	9	18.0332314	21.60619511	3.572963705	0.04830406	0.124506196
Hyal2	28	2.240986623	1.518712734	-0.722273889	0.04833515	0.124542587
Fem1b	86	1.184797893	1.105532941	-0.079264951	0.04833405	0.124542587
Eif3l	40	3.525943295	4.735145756	1.20920246	0.04838835	0.124657757
G6pd2	1	95.90163934	89.43089431	-6.470745035	0.04843612	0.12475892
N4bp2	42	1.737589477	1.520038182	-0.217551295	0.04851721	0.124930267
Twf1	31	1.484779299	1.188222556	-0.296556743	0.04851967	0.124930267
Cyp4x1os	5	12.85360746	7.665577342	-5.18803012	0.04854116	0.124941771
Cyp4x1	5	12.85360746	7.665577342	-5.18803012	0.04854116	0.124941771
Mbip	13	1.647697397	1.101380928	-0.546316469	0.04863653	0.125165273
230217C12R	5	8.057962294	5.520485135	-2.537477159	0.04867672	0.125246753
Timm13	39	11.37233418	11.06740459	-0.304929586	0.04870472	0.125296831
Gpr139	22	50.38101739	47.92562965	-2.455387747	0.04872838	0.125335722

Bsdc1	20	1.775476386	2.181700646	0.406224261	0.04893545	0.125846278
Mir6956	2	95.1597017	91.69239359	-3.467308113	0.04910632	0.126263549
Dnaaf5	87	1.379995604	1.077299795	-0.302695808	0.04913684	0.126319909
Ppp3r1	82	1.166064785	1.360647911	0.194583126	0.04916118	0.12636034
Npnt	95	1.780758088	1.39883954	-0.381918548	0.04919515	0.126425506
Snora21	24	1.599948395	1.126922081	-0.473026314	0.04927396	0.126605859
Lin28a	6	9.276724447	7.36801418	-1.908710267	0.04931644	0.126692841
Zscan25	13	87.79884236	86.49284309	-1.305999261	0.04936679	0.126799982
Arl5b	73	1.153514704	1.293571067	0.140056363	0.04950412	0.127130451
Gm16833	1	87.27272727	96.2962963	9.023569024	0.0495606	0.127253242
Socs5	86	1.556701271	1.37542376	-0.181277512	0.04957565	0.127269601
Bcl3	70	1.406244411	1.018388974	-0.387855437	0.04960606	0.127325397
Flt3l	11	5.090557674	3.692250401	-1.398307273	0.04964579	0.127405092
Nat14	9	63.72773276	66.62894521	2.901212444	0.04979633	0.127769083
Epb4.1l4b	215	1.493842145	1.232578623	-0.261263522	0.04988026	0.127962058
Cdk7	29	1.34066389	1.930597993	0.589934103	0.04989744	0.127983741
Arf3	69	1.363327629	1.441919821	0.078592191	0.0500297	0.12830056
Bcl9	63	1.793352314	2.092987914	0.2996356	0.05010702	0.128476394
Atxn1l	14	2.509301511	0.957175406	-1.552126105	0.05016286	0.128597102
Deaf1	64	2.103085707	1.670031529	-0.433054179	0.05018468	0.128608099
Mir194-1	6	95.72190456	94.12058061	-1.601323949	0.05018268	0.128608099
Lpcat1	37	1.184774305	0.55576886	-0.629005445	0.05021967	0.128675304
700007L15R	16	0.963492808	1.380038488	0.41654568	0.05024915	0.128706141
Mir7014	7	82.85247896	74.58575404	-8.266724924	0.05024925	0.128706141
Actn3	12	43.03463798	45.24682753	2.21218955	0.05025858	0.128707571
Snhg9	7	0.793650794	0.072150072	-0.721500722	0.05034518	0.128906845
Cks1b	14	2.849575228	2.078101	-0.771474228	0.05038719	0.128991907
Cped1	5	5.587214399	7.96628887	2.37907447	0.0504741	0.129146816
430402I18R	65	1.050920074	0.987991405	-0.062928669	0.05045967	0.129146816
Fbxo7	50	1.282034466	0.938007256	-0.34402721	0.05046897	0.129146816
Pigt	17	1.326784583	0.686778787	-0.640005796	0.05051797	0.12923651
Pi4k2b	44	1.241816764	0.827481378	-0.414335386	0.05063871	0.129522827
Tmem52	6	82.30719406	77.7808498	-4.526344257	0.05081273	0.129945286
Prmt7	21	1.425640792	0.992726448	-0.432914345	0.05084358	0.130001517

Tekt2	12	84.62378486	82.0629771	-2.56080776	0.05085287	0.130002613
Pdcl3	41	2.17648538	1.936266994	-0.240218387	0.0508654	0.130011993
Pex6	86	1.379582423	1.128723818	-0.250858605	0.05094693	0.13019771
Stam	28	0.991717567	0.465152805	-0.526564762	0.05100603	0.130326034
Prelid1	11	1.741927655	0.923458547	-0.818469108	0.05120884	0.130821457
Gm5069	30	2.389883145	1.91033731	-0.479545835	0.05124315	0.130863543
Syap1	17	31.21879952	27.5172518	-3.701547726	0.05123547	0.130863543
Med22	12	1.105755382	1.073547547	-0.032207835	0.05126754	0.130903053
Zfp771	46	1.67894891	1.717422118	0.038473209	0.051292	0.130923586
Adam15	56	2.754590715	3.011486139	0.256895424	0.05129342	0.130923586
Nobox	1	88.24940048	83.57142857	-4.677971908	0.05132972	0.130993445
Mroh1	38	8.361996575	6.580576662	-1.781419913	0.05139955	0.131148851
Phkb	11	1.181646756	0.932523063	-0.249123693	0.05144894	0.131229225
)21531C22F	29	1.54682562	1.90796198	0.36113636	0.05144562	0.131229225
30309D02F	47	1.084197393	0.619400439	-0.464796953	0.05157179	0.131519717
Gtf2ird1	36	2.804380762	2.368103503	-0.436277259	0.05166222	0.131717638
2-Mar	9	1.639566396	1.264832794	-0.374733602	0.05166735	0.131717638
Mir7083	4	95.55421167	91.99548506	-3.558726605	0.05170689	0.131772635
Zfp664	116	1.502500848	1.320399638	-0.18210121	0.05170315	0.131772635
Rgs22	8	9.239581111	8.008851673	-1.230729438	0.05173688	0.131802801
Kank2	36	1.035100147	1.513009546	0.477909399	0.05174483	0.131802801
Polr2g	8	2.162320738	1.402989707	-0.759331031	0.05174567	0.131802801
330080D01F	27	19.21489704	17.47976421	-1.735132829	0.05180328	0.131903758
Slc9a3r1	49	1.436054674	1.108737023	-0.32731765	0.05179673	0.131903758
Gm7120	48	5.53053069	4.457142207	-1.073388484	0.05182742	0.13194232
Pcsk7	84	1.319310346	1.288773719	-0.030536627	0.05186568	0.132016836
Mocos	31	1.487943586	1.622037889	0.134094303	0.05190203	0.132086429
Birc5	40	1.235443959	1.24921985	0.013775891	0.05196052	0.132212355
Bnip3l	64	1.34243859	1.037623721	-0.304814868	0.05197273	0.132220499
Kpna1	112	1.436109385	1.31746992	-0.118639465	0.05201561	0.132306642
Kidins220	16	0.848131379	1.626866164	0.778734785	0.05206003	0.132396699
Gm13807	1	73.56321839	81.55737705	7.994158658	0.05210302	0.132450726
Zfp426	20	1.07022581	1.771512376	0.701286566	0.05210297	0.132450726
Hvcn1	7	48.68773861	46.94329224	-1.744446372	0.0521086	0.132450726

Slc25a40	45	1.079652601	0.909660149	-0.169992452	0.05211738	0.132450726
Nap1l1	56	1.451689789	1.229728638	-0.221961151	0.05212698	0.132452174
Gm10789	17	34.83919174	33.63659963	-1.202592108	0.05223814	0.132711645
Cabp1	10	69.30645501	71.69069228	2.384237264	0.05225508	0.132731694
Rasl11b	43	0.88244051	1.18613118	0.30369067	0.05227408	0.132756985
Kctd11	42	1.761453886	2.092623496	0.331169609	0.05228319	0.132757149
Hif1a	45	1.196964211	0.839917336	-0.357046876	0.0523479	0.132898463
Srrt	30	2.282488778	1.643464866	-0.639023913	0.05240571	0.133022215
Lat	15	87.29435399	85.04433241	-2.250021577	0.05244595	0.133101333
Trove2	43	1.438722107	1.649104483	0.210382376	0.05246715	0.133109099
Tor1a	44	1.269341539	1.38394121	0.114599671	0.05246375	0.133109099
Aars	7	1.167313226	1.965097534	0.797784307	0.05249412	0.133154481
Fzd1	44	1.596307747	1.222403882	-0.373903866	0.05250329	0.133154741
Parm1	27	2.103102229	1.306217056	-0.796885172	0.05253032	0.13320026
Plekhd1	33	9.43249109	11.0575285	1.625037412	0.05258428	0.133314039
Tmem199	29	1.46724096	1.19215615	-0.27508481	0.05266205	0.13348816
Bora	71	1.236187896	0.88842118	-0.347766716	0.05276133	0.133716703
Guf1	45	1.48106745	1.131126276	-0.349941174	0.05283243	0.133873792
Tspan5	85	1.888012018	2.529848584	0.641836566	0.05287776	0.133965515
Tmem194b	23	1.814809363	1.03404768	-0.780761683	0.052912	0.134029131
Mcrs1	19	1.282989787	1.565234317	0.282244529	0.05295205	0.134107417
Tdg	12	0.467722654	0.9913051	0.523582446	0.05298705	0.134172895
Arf2	25	0.963220323	0.467017306	-0.496203017	0.05304668	0.134300724
Plxnb2	56	1.787078267	1.426688506	-0.360389761	0.05313093	0.134490824
Mpp5	85	1.489346206	1.268343811	-0.221002396	0.0532524	0.134775058
Hacd1	72	2.114713249	1.474345483	-0.640367766	0.05349443	0.135364255
Pgm3	18	1.723591961	2.277506528	0.553914567	0.05353919	0.135454156
)21507P07F	51	2.728241596	2.410507394	-0.317734202	0.05358283	0.13551801
Nle1	46	1.573762555	0.962205504	-0.611557051	0.0535829	0.13551801
Rfc4	33	1.005290493	1.394701017	0.389410524	0.05362951	0.135612511
Ccdc42b	1	53.94736842	44.88888889	-9.058479532	0.05368179	0.135721335
Ndrg1	40	5.791064234	3.522022781	-2.269041452	0.05374964	0.135869454
Hoxb2	5	61.68455699	55.7402528	-5.944304192	0.05376858	0.135893416
Klf4	69	1.363718179	1.46943906	0.105720881	0.05377764	0.135893416

Glb1l2	7	21.66423623	20.07909787	-1.585138359	0.05380957	0.13595069
Actn1	50	2.122243823	1.803158186	-0.319085637	0.05382333	0.135962057
Ep300	106	1.351125243	1.113272383	-0.237852859	0.05386766	0.1360506
Pebp1	46	6.89250155	6.216159868	-0.676341682	0.05396445	0.136271601
Senp2	58	1.432243613	1.305672152	-0.126571461	0.05410029	0.136591136
700104L18F	2	82.50314692	77.49405234	-5.009094577	0.05415555	0.136707138
Cyyr1	7	39.88216606	37.56883015	-2.313335908	0.05417443	0.136731268
Brms1	31	1.187026634	1.487234792	0.300208158	0.05433535	0.137113835
Rabgef1	69	3.74582661	3.225475101	-0.520351508	0.05442097	0.137295175
Fbxw2	49	1.352527176	1.41944586	0.066918683	0.05442593	0.137295175
Bud31	29	1.166516452	1.619743476	0.453227024	0.05445005	0.13733243
Gmfg	27	1.329721435	1.00527344	-0.324447996	0.05460738	0.137705575
Smarce1	31	0.773142018	1.230927857	0.457785839	0.05464764	0.137783424
Pvrl2	34	1.998272112	2.386922769	0.388650657	0.05468451	0.137852676
Pgm2	32	0.614722485	1.064280638	0.449558153	0.05482712	0.138164726
Tle3	24	1.265233491	0.605823674	-0.659409817	0.05482649	0.138164726
Ooep	8	6.572570457	5.919055346	-0.653515112	0.05494683	0.138442624
Zfp3	61	1.529631359	1.053304991	-0.476326368	0.05503228	0.138634108
Itgb1bp1	28	1.889910071	1.191699282	-0.698210789	0.05517266	0.138963899
Cnpy2	10	1.329697329	2.394940483	1.065243155	0.05532379	0.13932063
Mir1961	2	43.68421053	35.54216867	-8.142041852	0.05533626	0.139328132
Fiz1	15	0.866722652	1.03826286	0.171540209	0.05554711	0.139811036
730408K05F	14	1.468047055	0.858746768	-0.609300287	0.05554013	0.139811036
Rbbp4	63	1.184152865	1.422686513	0.238533649	0.05559055	0.13986465
Ulk3	44	1.556308381	1.16554065	-0.390767731	0.05558779	0.13986465
Armc2	13	5.761990673	4.213638665	-1.548352008	0.05559701	0.13986465
Vgf	44	9.561132367	8.096604153	-1.464528214	0.05569816	0.140095105
H2-M5	2	75.18796992	81.79347826	6.605508336	0.05574655	0.140192772
Hgsnat	22	1.38804542	1.022838436	-0.365206984	0.0557802	0.140205313
Prss48	10	93.7122758	92.14514194	-1.567133857	0.05577626	0.140205313
Sez6l	39	55.03319881	56.61719634	1.58399753	0.05576383	0.140205313
Fam19a3	21	29.60298443	28.12011315	-1.482871272	0.05586324	0.140389998
Fam64a	32	1.382356643	1.708778951	0.326422308	0.05603044	0.140786076
Myh14	20	24.05780295	26.01196039	1.954157446	0.05609906	0.140934361

Hhip	20	19.14881922	17.12537565	-2.023443565	0.05616445	0.141055092
Perp	54	5.197125533	4.511494947	-0.685630586	0.05616634	0.141055092
Gzf1	23	1.737131674	1.472739238	-0.264392436	0.05626568	0.141280384
10044O15R	2	5.679674099	3.423215399	-2.256458701	0.05631161	0.141356603
Pgm1	44	2.136862151	1.719194831	-0.41766732	0.0563153	0.141356603
Timm50	36	1.21353819	0.958284677	-0.255253513	0.05644021	0.141645917
Rpl22	11	59.08426167	60.68456469	1.60030302	0.05646679	0.141688385
Klf7	41	1.209874606	1.060798334	-0.149076272	0.05648957	0.141721304
Zfp825	3	4.82146736	3.110962678	-1.710504681	0.05650228	0.141728958
Eif1ad	59	1.230767574	1.152660421	-0.078107153	0.05651219	0.141729599
Cdc27	47	1.111654982	0.82802898	-0.283626002	0.05656232	0.14183108
Duoxa1	16	43.54654249	40.24316662	-3.303375862	0.05658795	0.141871117
Stxbp5	84	1.340213202	0.914825122	-0.42538808	0.05661931	0.141889355
Cbr2	6	86.08282508	82.31676673	-3.766058352	0.05660593	0.141889355
Nmrk1	6	3.609330415	1.871088166	-1.738242249	0.05662424	0.141889355
Ubr5	103	0.967880095	1.090959583	0.123079488	0.056678	0.141999821
Ythdf2	63	1.2798186	1.098866723	-0.180951877	0.05675842	0.14215276
Rfx4	21	25.30515624	24.3097389	-0.995417331	0.0567506	0.14215276
Galk1	15	1.967659111	1.579392144	-0.388266967	0.05678419	0.142193049
Kcmf1	128	1.067562377	1.161776531	0.094214153	0.05680901	0.142230923
Chrnb1	24	1.506163906	1.789687668	0.283523762	0.0569229	0.142491757
Prss23	72	2.753176943	2.466742687	-0.286434257	0.05695533	0.14252431
Trim67	56	5.087461748	5.614884667	0.52742292	0.05694902	0.14252431
Gon4l	4	0.462962963	1.742976554	1.280013591	0.0571588	0.1429847
Eps15l1	22	1.131133189	0.551619256	-0.579513932	0.0571558	0.1429847
Bmper	83	1.928159586	1.663218414	-0.264941171	0.05724039	0.143164392
St6gal1	22	2.774210037	2.056718572	-0.717491465	0.05733429	0.143350389
Rab32	23	44.47303171	45.8177573	1.344725594	0.05733389	0.143350389
D2Wsu81e	21	2.36529505	1.739875402	-0.625419648	0.05738814	0.143460577
Sirt4	3	0.508905852	2.517744744	2.008838891	0.0574307	0.143542512
Cd9	27	1.31953596	1.1364391	-0.18309686	0.05759363	0.143925221
Aldh1b1	26	17.04727988	17.577908	0.530628119	0.05764203	0.144021637
Dnpep	67	0.813661124	0.942320571	0.128659447	0.05767515	0.14407987
Hs3st1	18	1.678214865	1.253375673	-0.424839192	0.05769698	0.144109878

Pafah2	4	61.04166667	56.00446529	-5.037201373	0.05773766	0.144186934
Ifngr2	34	2.116790741	1.180234032	-0.936556709	0.05775453	0.144204529
Utrn	1	54.45544554	40.86021505	-13.59523049	0.05784485	0.144405474
'32416N19F	61	1.384504547	1.167163783	-0.217340764	0.05790981	0.144543041
Draxin	34	10.35179005	9.224604836	-1.127185218	0.05793166	0.144572978
Psmd2	42	1.152149582	1.307917736	0.155768155	0.05797509	0.144656761
R3hcc1	19	1.6072713	0.990189402	-0.617081899	0.05799661	0.14466161
Gm11696	24	1.105043847	1.564650187	0.45960634	0.05799675	0.14466161
Zc3h13	33	1.462088189	0.954053808	-0.508034381	0.05800669	0.144661813
Rad51b	15	1.710779292	1.072975392	-0.6378039	0.05803001	0.144690914
Pax9	9	3.639167938	2.657348178	-0.981819759	0.05803808	0.144690914
Mnd1	22	1.118130327	1.963462415	0.845332088	0.05807296	0.144753292
Mir6905	8	86.11192414	85.09048077	-1.021443375	0.0581817	0.144999718
Nsmce1	29	11.0885532	9.757772938	-1.33078026	0.05824025	0.145120979
Csrnp1	3	55.86149353	61.95288806	6.091394528	0.05827858	0.145191824
Msl1	129	1.62865994	1.42460208	-0.20405786	0.05833771	0.14531448
Abi3	1	48.93617021	37.5	-11.43617021	0.05839178	0.145424473
Nrbp1	97	1.376845982	1.229580386	-0.147265596	0.05845915	0.145567537
AI464131	7	2.254953266	3.297929553	1.042976287	0.05856014	0.145769553
C87436	14	2.386195707	1.448124426	-0.938071281	0.05855721	0.145769553
Wnt6	53	8.004619624	7.437518172	-0.567101452	0.05857414	0.145779668
Rnf40	34	1.719403259	1.558879344	-0.160523915	0.05860107	0.145821968
Mia3	60	2.011689953	1.548207451	-0.463482502	0.05862998	0.145869152
Vps51	14	1.950083658	1.676365847	-0.273717811	0.05865374	0.145903548
Clcc1	13	0.856444319	0.503271322	-0.353172997	0.05868264	0.145950695
Lgr6	17	2.52832341	3.564892051	1.036568641	0.05882462	0.146279026
Ranbp2	70	0.876547322	0.950125681	0.073578359	0.05885612	0.146332556
Mad2l1	32	1.331464175	1.830295382	0.498831207	0.05896383	0.146575513
St7	70	0.88944153	1.189021073	0.299579543	0.05905328	0.146773012
Mafk	43	1.635830715	1.371570986	-0.264259729	0.05911737	0.146907415
Rnf133	2	76.47058824	66.30952381	-10.16106443	0.05916972	0.147012616
Stk17b	100	1.343547326	0.956044075	-0.387503251	0.05921118	0.147090709
Med24	1	7	3.533568905	-3.466431095	0.05922343	0.147096247
Rcn2	33	0.859230868	1.371337532	0.512106665	0.05925338	0.147145737

Rerg	46	53.13425276	51.98852715	-1.145725618	0.05929776	0.147231028
Sec22b	16	1.848171537	1.300703158	-0.547468379	0.05942	0.147509584
Wdr82	70	1.508441446	1.143056263	-0.365385182	0.05944332	0.1475425
Mrps26	42	1.389940111	2.014944797	0.625004686	0.05948323	0.147616595
Gm16796	24	1.339686549	0.871030298	-0.468656251	0.05950183	0.14763779
Wdsub1	32	1.458341573	1.611270688	0.152929114	0.05951627	0.147648637
Fbxl20	125	1.639036034	1.263004688	-0.376031346	0.05971588	0.148118791
Fam102b	63	1.083057583	0.916144976	-0.166912607	0.05973818	0.148149081
Lrrc41	34	0.987342235	1.45186816	0.464525925	0.05975131	0.148156584
Sema7a	100	6.69207159	6.080931427	-0.611140163	0.05976661	0.148169502
Daam1	12	0.587930039	1.232904946	0.644974907	0.05978364	0.14818667
Has2	4	6.882652934	5.345644857	-1.537008077	0.05982152	0.14825554
Psmb8	13	2.56560319	1.188012282	-1.377590908	0.0598776	0.148369458
Glis3	66	1.531133618	1.332426355	-0.198707263	0.05999219	0.148628296
Ctsl	35	1.875383543	1.160849925	-0.714533618	0.06001468	0.14865891
Trh	15	11.29381938	12.83605212	1.542232745	0.06008793	0.148815236
Mfsd7c	27	6.244874691	8.114497105	1.869622414	0.06020627	0.149083154
Itpka	100	1.61440377	1.102712109	-0.511691661	0.06022563	0.149105937
Kntc1	4	0	1.058467742	1.058467742	0.06024921	0.149139156
Ckap2l	19	1.39885127	0.974953879	-0.42389739	0.06026884	0.149162586
Nasp	21	1.014686649	1.293685883	0.278999234	0.06051214	0.149739474
Cpne1	65	1.587480117	1.499270627	-0.08820949	0.06055989	0.149807122
Rbm12	65	1.587480117	1.499270627	-0.08820949	0.06055989	0.149807122
Ccne2	154	1.264391237	1.329756655	0.065365418	0.06059503	0.149868785
Mif	15	1.67500671	2.546249126	0.871242416	0.06063874	0.149951608
Npm3-ps1	8	93.46038299	95.82284638	2.362463389	0.06066966	0.150002791
Rbm17	77	1.39638502	1.075245334	-0.321139686	0.06069871	0.150049343
Cdr2	42	1.813794761	1.424049564	-0.389745197	0.06075947	0.15017423
Luc7l3	67	1.358479602	1.103086845	-0.255392757	0.06078097	0.150202075
Depdc7	42	1.955412389	1.377059219	-0.57835317	0.06083119	0.150300871
Exoc6b	32	0.848110274	1.691821095	0.84371082	0.06089794	0.150440475
Tfdp2	35	1.300901049	1.659500233	0.358599184	0.06092003	0.150469712
Slc26a2	37	1.050655042	1.195847486	0.145192445	0.06094443	0.150504645
Fam192a	33	1.234221173	1.100027	-0.134194174	0.06098054	0.15056848

Kdm6a	60	1.639737236	1.255990695	-0.383746541	0.06100963	0.150614969
Rtkn2	37	2.538300081	2.035850757	-0.502449324	0.06104273	0.150671329
Fam69a	65	1.067348988	1.109581726	0.042232739	0.06113933	0.150884391
Tgfbr3l	1	43.10344828	34.03361345	-9.06983483	0.06121627	0.151048847
110009E18F	12	2.499330894	1.395970581	-1.103360313	0.06126349	0.151086124
Ppfibp2	26	9.463411743	7.911654465	-1.551757278	0.06127256	0.151086124
Ccdc150	12	1.52000727	1.856562719	0.336555449	0.06126878	0.151086124
Gm11423	2	4.915830739	2.511312783	-2.404517956	0.06124864	0.151086124
Pecr	5	3.366499808	2.539219082	-0.827280725	0.0613206	0.151179169
Zfp335	71	5.866290111	5.574634379	-0.291655732	0.06142189	0.151352623
Eml3	49	1.541402694	0.97926191	-0.562140784	0.06141454	0.151352623
Ormdl1	37	0.960653922	1.13268186	0.172027938	0.06140219	0.151352623
Exosc9	13	0.617901719	1.023121451	0.405219732	0.06156989	0.151691843
530601H04F	9	1.748450996	3.47995364	1.731502644	0.06164315	0.151846842
Cdkn2b	47	1.864771078	1.599028959	-0.265742119	0.06166224	0.151868374
Ctbp2	119	11.68591848	11.47427739	-0.211641095	0.06174481	0.152046206
Pmpca	15	1.817858394	1.024011014	-0.79384738	0.06182422	0.152216208
Wbscr22	55	1.555831283	1.189439474	-0.366391809	0.06183967	0.152228704
Rhbg	21	1.786939408	2.111890815	0.324951408	0.06198429	0.152559126
Fam129b	59	2.397842401	1.800727034	-0.597115367	0.06203598	0.152660747
230014O12F	3	66.78310642	61.96942975	-4.813676673	0.0620538	0.152663199
Obscn	2	83.6236091	81.2962963	-2.327312799	0.06205779	0.152663199
Prrxl1	36	41.87141398	41.97193743	0.100523451	0.0620731	0.152675281
Pkn2	123	1.354723744	1.038542188	-0.316181556	0.06213839	0.152810245
Kansl1l	147	1.485200818	1.339260105	-0.145940712	0.06215415	0.152823382
Gcnt2	16	2.268467806	1.515642731	-0.752825075	0.0622005	0.152911727
Nadk2	90	1.281463347	1.050867433	-0.230595914	0.06234429	0.153239556
D5Ertd579e	57	2.017620343	1.607577106	-0.410043236	0.06254015	0.153642053
Chst2	91	23.72386576	23.65175604	-0.072109722	0.06254993	0.153642053
Rrm1	6	2.652213805	1.492126635	-1.16008717	0.06251907	0.153642053
Samm50	31	1.278052333	1.524680725	0.246628392	0.06253574	0.153642053
Tnpo3	12	1.576601474	1.416023768	-0.160577706	0.0626917	0.153964529
Nudt12	10	1.757204765	2.429490197	0.672285432	0.06277669	0.154147449
Rhno1	66	0.917450006	1.298250715	0.380800708	0.06280351	0.154187497

Cdc20	60	1.721822889	1.534671027	-0.187151862	0.06285358	0.154218912
Comtd1	23	1.421955794	1.634261799	0.212306005	0.06284779	0.154218912
Ntn5	3	42.79413857	38.23809524	-4.556043331	0.06285834	0.154218912
Phf10	47	1.063152752	0.875336331	-0.187816421	0.06285118	0.154218912
Glt28d2	28	1.680837485	2.163739638	0.482902154	0.06291192	0.154324566
Tmem86a	52	1.373644653	1.34250586	-0.031138793	0.06293102	0.15434561
Rufy3	117	3.142737001	2.847730654	-0.295006347	0.06298562	0.154408674
Col8a2	34	14.54566728	16.46072668	1.915059402	0.06299985	0.154408674
Cdc6	10	1.870498081	2.532124472	0.661626391	0.06296943	0.154408674
Fzd2	2	0.729927007	2.482708409	1.752781402	0.06300935	0.154408674
Gm10767	2	1.030927835	0	-1.030927835	0.06299992	0.154408674
'00123O20F	32	1.169036428	1.468082098	0.29904567	0.06305427	0.154492962
Stk40	37	1.674081067	1.756096553	0.082015486	0.06311856	0.154624656
Psmg4	2	3.021978022	5.326202852	2.30422483	0.06316973	0.154724197
Pla2g10os	1	48.63013699	38.18181818	-10.4483188	0.06320706	0.154789788
Gnai2	40	1.41327376	1.466162266	0.052888506	0.06334132	0.155092705
Plk1	2	6.666666667	1.879699248	-4.786967419	0.0635543	0.155558452
Fam136a	51	0.901905831	1.054378198	0.152472367	0.06354238	0.155558452
Stk11	64	0.884683338	0.967840982	0.083157645	0.06356334	0.155558452
.30317F20R	82	1.321711803	1.157236932	-0.164474871	0.06357684	0.155565538
.30050O18F	2	65.27061856	72.77695278	7.50633422	0.06366561	0.15575679
Myo5b	106	1.714482199	1.41906761	-0.295414589	0.06373615	0.155903349
'00025G04F	49	1.97979332	1.228445338	-0.751347981	0.06380366	0.156042497
Kcnj5	3	10.02350843	8.819828572	-1.203679859	0.06383868	0.156102112
BC049730	1	57.33333333	41.79104478	-15.54228856	0.06389277	0.156208337
)30429B21F	63	2.402838914	1.872039244	-0.530799669	0.06409536	0.156677542
Pgpep1	73	1.70474726	1.458782087	-0.245965173	0.06414889	0.156777357
Veph1	5	27.89424893	23.05789784	-4.836351088	0.06415756	0.156777357
Ncor2	104	1.04507603	0.877909313	-0.167166717	0.06418093	0.156808358
Capzb	54	1.706906683	1.416300903	-0.29060578	0.06425964	0.156922267
)30455D15F	32	1.270501921	1.039238382	-0.231263539	0.06425847	0.156922267
Actr10	36	2.317483107	1.812395011	-0.505088095	0.06424728	0.156922267
Iqgap3	16	1.557282721	1.040593238	-0.516689483	0.06428743	0.156964009
Zfp873	21	1.572429269	1.292274917	-0.280154352	0.06433972	0.157065555

Dvl2	21	6.725110562	7.368354907	0.643244345	0.0644278	0.157254424
Gnb1	12	1.962930381	1.019546821	-0.94338356	0.06449092	0.15738229
Rgs19	66	1.558803991	1.225286161	-0.33351783	0.06451965	0.157426233
Usp39	28	0.717338773	1.175650433	0.458311659	0.06453529	0.157438205
Ccrl2	4	3.06851979	0.92361405	-2.14490574	0.06457006	0.157444502
933407E24R	1	44.73684211	60	15.26315789	0.0645675	0.157444502
Ift20	37	1.157707502	0.976098926	-0.181608576	0.06456422	0.157444502
Prkab2	61	1.4136778	1.251790849	-0.16188695	0.06464057	0.157491741
Ngrn	22	0.788295373	0.90320763	0.114912257	0.06460737	0.157491741
Mrpl2	34	2.327450578	1.692256732	-0.635193846	0.06463309	0.157491741
Zfp58	4	3.184842584	4.916219059	1.731376476	0.06463376	0.157491741
Gtf2e1	41	1.842442627	1.604087709	-0.238354917	0.06464309	0.157491741
Dnah6	10	30.42976173	33.88072179	3.450960059	0.06474163	0.157705631
Ppfia3	40	1.562533028	2.312188106	0.749655078	0.06479665	0.15781345
Ppp1r3c	21	14.2038831	14.3554063	0.151523201	0.06481347	0.157828217
Faah	73	1.519665714	1.58317459	0.063508876	0.06485918	0.157876943
Prkcd	25	1.520344211	1.204358193	-0.315986018	0.06486576	0.157876943
Astn2	73	47.00627594	47.87937277	0.873096826	0.06486308	0.157876943
Smim4	20	1.497214142	1.079332173	-0.41788197	0.06496408	0.158090033
Fbxo43	42	1.871841465	1.744042796	-0.127798669	0.06497867	0.158099318
Sumo3	82	0.986040828	1.038124163	0.052083335	0.06500991	0.15814912
310068J16R	43	1.685672411	1.262533341	-0.42313907	0.06511233	0.158372003
Necap2	45	1.553082562	1.366670803	-0.186411759	0.06512934	0.158387119
Klhl38	4	32.80716439	36.54597758	3.738813192	0.06524708	0.158647165
Fam46b	30	8.234114204	6.44818804	-1.785926165	0.06528638	0.158716414
Cct2	66	1.491884727	1.048616846	-0.44326788	0.06533024	0.158796732
Rpl15	39	1.995637432	1.368682958	-0.626954474	0.06541877	0.158985601
Zfp229	9	2.111176501	1.517963221	-0.593213281	0.06552562	0.159218909
Ptpn6	4	64.28424609	60.26676181	-4.017484279	0.06562983	0.159445714
Kcnh7	12	26.86480755	31.07891297	4.214105423	0.06568443	0.159538806
Mpzl3	10	1.801584119	2.003073468	0.201489349	0.06568989	0.159538806
Tap2	11	4.927360372	3.527116545	-1.400243827	0.06574078	0.159635986
Nacc1	61	1.094335031	1.231759746	0.137424715	0.06584277	0.159857171
Ptprs	102	4.612049292	4.2061823	-0.405866992	0.06586922	0.159894945

Actr3	67	1.506179407	1.158098875	-0.348080532	0.06600411	0.1601429
'00001D01F	2	49.97474747	40.45550847	-9.519239	0.06599404	0.1601429
Nek6	54	1.037711683	0.629926159	-0.407785524	0.066003	0.1601429
Gemin4	12	1.694588542	1.207064988	-0.487523554	0.06608503	0.16028623
Usp4	52	1.341910152	1.293098184	-0.048811967	0.06608001	0.16028623
Eif4g3	75	5.155064868	4.303910257	-0.851154612	0.06623749	0.160629477
Trappc8	77	1.548736116	1.008070801	-0.540665316	0.06625649	0.160649004
Rad54l2	62	1.476429394	1.625266615	0.148837221	0.06628149	0.16068306
Lrrc58	78	1.001678795	0.771693078	-0.229985717	0.06644334	0.160995634
Tssc4	44	1.47616528	0.988096666	-0.488068615	0.06644211	0.160995634
Ddx49	10	1.62211595	1.53510667	-0.087009279	0.06643423	0.160995634
Ccni	32	0.645012435	0.747753957	0.102741521	0.06646793	0.161028625
Gm15972	12	2.419638606	1.885682869	-0.533955737	0.06648693	0.161048082
Slc39a11	13	2.913122701	2.168220998	-0.744901703	0.06652636	0.161115095
Ddx39	55	1.272024205	1.513847102	0.241822897	0.06653656	0.161115095
Pex26	4	3.459624952	2.400914634	-1.058710318	0.06663306	0.161322148
Tmem129	24	0.573097071	1.180837452	0.607740381	0.06667163	0.161388909
Rnf13	22	1.520777043	1.439624697	-0.081152347	0.06671062	0.161456651
Rcbtb2	31	1.560623641	1.63263205	0.07200841	0.06689632	0.161879396
Usp1	95	0.952374204	0.944045741	-0.008328462	0.06706708	0.162265841
Psme1	14	1.626913558	1.941618443	0.314704885	0.06710056	0.16232009
Pax5	5	73.02325581	78.60869565	5.585439838	0.0671679	0.162456205
Ppargc1b	63	1.386877034	1.760984631	0.374107597	0.0672563	0.162643204
Sfmbt2	21	31.34681661	34.16876136	2.821944753	0.06734533	0.16283166
Nup54	46	1.224988307	1.564240383	0.339252076	0.06737063	0.162865997
Gm14634	2	13.5606455	8.205128205	-5.355517292	0.06741702	0.162951294
Ints3	31	1.043398845	1.512459591	0.469060746	0.06744137	0.162983301
Plbd2	46	1.43059704	1.162382179	-0.26821486	0.06748428	0.163060153
Dennd6a	38	0.86700524	0.679764023	-0.187241217	0.06752136	0.163096035
P4ha2	55	1.975380635	1.556131639	-0.419248996	0.06751666	0.163096035
Dnajc27	14	0.993820388	1.616893156	0.623072768	0.06756537	0.163175494
Rit1	11	1.609868286	2.554376379	0.944508093	0.06760583	0.163246324
St7l	17	2.398626463	1.748078136	-0.650548327	0.06764676	0.16331829
Il17rb	25	8.569517983	9.384612877	0.815094894	0.06768153	0.163375355

Rab15	73	11.50486892	12.42425428	0.919385355	0.06772713	0.163458532
Mir193a	123	1.525339802	1.213386497	-0.311953305	0.06782336	0.163663879
Vbp1	7	19.17500924	15.72380951	-3.451199734	0.06787678	0.163765846
Zfp710	83	2.265275691	1.868736229	-0.396539462	0.06800947	0.16403508
Ssh1	74	1.897948715	1.50577224	-0.392176475	0.06801073	0.16403508
Sh3tc1	4	2.895687875	1.798363459	-1.097324416	0.06812434	0.164282091
Rad54l	5	1.40775921	0.711046056	-0.696713153	0.06816371	0.164296046
[illegible]10003B16R[illegible]	5	1.40775921	0.711046056	-0.696713153	0.06816371	0.164296046
Hmgcr	48	1.004757558	0.861082896	-0.143674662	0.06814759	0.164296046
Zfp512	2	1.103061336	0	-1.103061336	0.06820452	0.164367402
Phospho2	11	1.076744386	1.850792762	0.774048375	0.06824589	0.16443307
Eif5	74	6.422533442	5.276364859	-1.146168584	0.06825418	0.16443307
Glyctk	3	92.64014389	91.15817579	-1.481968101	0.06829691	0.164509017
Psmc3ip	34	2.593660781	1.971328329	-0.622332452	0.06842635	0.164793748
Slc12a9	25	1.398989682	1.726643694	0.327654012	0.06844791	0.164818605
Micall2	44	1.986076529	1.47967114	-0.50640539	0.06848164	0.164872787
Tmem147	44	1.386971017	0.825274674	-0.561696344	0.06856187	0.165038847
Rictor	122	1.09837471	1.309266723	0.210892013	0.06859077	0.165081342
Mmp14	7	1.932619566	1.065459166	-0.867160401	0.06861201	0.165105376
Taf15	55	2.625130716	1.706968442	-0.918162274	0.068735	0.165374221
Raly	55	1.921725958	1.938826433	0.017100475	0.06876393	0.165416695
Cops7a	58	1.2195222	1.569799684	0.350277484	0.06882547	0.165537617
Gng12	77	1.343918762	1.406740678	0.062821916	0.0688562	0.16558437
Ctcfl	3	89.18761482	89.2817475	0.094132678	0.06889573	0.165652287
Per2	69	0.946784827	0.929781154	-0.017003672	0.06892862	0.165704233
St3gal6	26	2.344924356	1.997470319	-0.347454037	0.06905697	0.165985577
Sgpp2	28	6.521273933	7.50379423	0.982520296	0.06936249	0.166675426
Mettl13	27	1.612385909	1.250282835	-0.362103074	0.06936669	0.166675426
Tmem114	22	12.60868662	12.53583967	-0.072846953	0.06945389	0.166857626
Fam163b	29	21.91798491	23.56801669	1.650031778	0.06954513	0.167028231
Sipa1l1	130	1.281359453	1.429888069	0.148528615	0.06954767	0.167028231
Lsg1	15	1.956550781	0.964857655	-0.991693126	0.06958373	0.167087482
Ppil4	35	1.232624843	1.129527132	-0.103097711	0.06962688	0.167163748
Tada3	17	1.719389354	1.22346063	-0.495928724	0.06965313	0.167199418

Plcd3	31	3.110965347	2.705073514	-0.405891833	0.06973082	0.167358528
Mcph1	54	1.265340858	0.942302054	-0.323038803	0.06976151	0.167404814
Tmem184b	81	1.302951173	1.214846125	-0.088105048	0.06978477	0.16743325
Igsf23	4	48	54.30088014	6.300880138	0.06989757	0.167676477
Gm2762	2	83.87115296	75.13209323	-8.739059738	0.06993182	0.167703787
Spop	36	0.995927105	1.299916311	0.303989207	0.06992318	0.167703787
Alkbh3	27	2.074899058	1.868834341	-0.206064716	0.06999357	0.167780937
930404I05R	11	1.668935066	0.971858063	-0.697077003	0.0699827	0.167780937
Ppp1r14a	43	4.618394327	4.549588431	-0.068805896	0.06999829	0.167780937
Slc31a2	24	1.958452312	1.578913709	-0.379538604	0.07001569	0.167795238
Asrgl1	7	0.360144058	1.086956522	0.726812464	0.07022853	0.168266441
Cwc27	10	1.47047915	0.99510034	-0.47537881	0.07023524	0.168266441
Mroh2b	1	74.57627119	58.49056604	-16.08570515	0.07032099	0.168444366
Cmpk2	20	2.562074286	1.727881051	-0.834193235	0.07035035	0.168487194
Eif2b2	39	1.628371259	1.057767676	-0.570603583	0.07055318	0.168945388
Dnajc9	33	1.386314389	0.976191561	-0.410122828	0.07064177	0.169129926
Gm14295	3	12.79669763	15.49974033	2.703042707	0.07085781	0.169619506
Npm2	2	82.7688172	73.28767123	-9.481145971	0.07092507	0.169752818
Ttbk2	53	1.476620645	1.152614721	-0.324005924	0.0710022	0.169909711
Mdm1	54	1.44301653	1.739538998	0.296522468	0.0710153	0.169913332
Lzic	45	1.250808471	1.50742307	0.256614599	0.07106732	0.170010094
Sdc3	89	1.783644563	1.581372749	-0.202271814	0.07111293	0.170091473
Dpagt1	15	1.308688611	1.50778882	0.199100209	0.07114339	0.170136595
Fam172a	26	1.333396936	0.806587602	-0.526809333	0.0711745	0.170183257
Rptn	1	98.0952381	93.58974359	-4.505494505	0.07119732	0.170190338
Lsm4	12	2.664640038	1.910762157	-0.753877881	0.07120066	0.170190338
Cited2	65	1.551313268	1.655443179	0.104129911	0.07124095	0.170258902
Fam135b	8	58.68068455	54.51843768	-4.162246863	0.07133927	0.170466128
Ppp4c	45	1.278327267	1.015415302	-0.262911965	0.07149726	0.170815816
Tpm4	24	0.751973606	0.677356947	-0.07461666	0.07153727	0.170883584
Rbbp9	10	2.688994406	1.952081313	-0.736913093	0.07158021	0.17095833
Cdk12	31	1.13653629	0.935218824	-0.201317466	0.07163149	0.171052974
Ubap1	29	1.387264999	1.440948522	0.053683523	0.07164434	0.171055823
Plcg1	95	1.095294337	1.088996846	-0.006297491	0.07179646	0.171391117

Syncrip	96	1.750176405	1.320236448	-0.429939956	0.07182442	0.171418739
Mob3c	24	1.397219918	0.964827965	-0.432391954	0.07183139	0.171418739
Uqcc2	5	1.271787849	1.821507639	0.54971979	0.07190291	0.171561524
Rps17	39	0.818182912	1.023947733	0.205764822	0.07193838	0.171618243
Tmie	2	10.48458841	7.103068172	-3.381520242	0.07208912	0.17194989
Klhdc4	38	2.873277845	1.873500348	-0.999777497	0.07215747	0.172056987
Mir7075	19	2.078728974	1.542439914	-0.53628906	0.07215374	0.172056987
Mir6945	28	96.93501632	95.94453519	-0.990481136	0.07225872	0.172270426
Kcnab3	13	3.754118097	3.132639647	-0.62147845	0.07230857	0.172361268
Hspa1b	5	1.794871795	0.487804878	-1.307066917	0.0724	0.17253256
Cdk2ap1	57	1.011484421	1.020837483	0.009353062	0.07240395	0.17253256
110038F14R	44	2.104689355	2.00322295	-0.101466404	0.07245303	0.172611388
Ncbp2	29	1.40539829	0.84800619	-0.5573921	0.07246055	0.172611388
Cirh1a	18	0.7835554	1.490731674	0.707176274	0.07251347	0.172709408
Ppa1	52	1.13266327	1.456100649	0.323437379	0.07252711	0.172713849
Mir8099-1	3	87.35689262	81.16500332	-6.191889299	0.07262658	0.172894614
Mir8099-2	3	87.35689262	81.16500332	-6.191889299	0.07262658	0.172894614
Arfgap2	72	1.129941835	1.502255764	0.372313929	0.07267669	0.172985848
Tmem74	11	22.80323807	21.28698095	-1.516257124	0.07272723	0.17307807
Foxa3	51	5.551953134	6.251986159	0.700033025	0.07278959	0.173198025
Pttg1	13	14.34262509	8.037643296	-6.304981793	0.07280124	0.173198025
Fa2h	69	2.386387307	2.117550423	-0.268836884	0.07287236	0.173339109
Calm3	48	1.575087471	1.180544749	-0.394542721	0.07298472	0.173554623
Zbtb42	86	3.578494157	3.131341853	-0.447152304	0.07299844	0.173554623
.10020A21R	1	4.519774011	1.470588235	-3.049185776	0.07299661	0.173554623
Mrps34	22	1.204080875	0.866654924	-0.337425951	0.07301765	0.173572172
Exog	39	1.174137644	0.854724296	-0.319413348	0.07305135	0.173624146
Camk2d	85	1.504403437	1.164359355	-0.340044081	0.07307158	0.173644111
Bre	39	0.782244735	0.950282759	0.168038025	0.0732623	0.174069146
430020J02R	1	7.917888563	4.624277457	-3.293611106	0.07333979	0.174225046
Adam10	82	1.28416406	1.074187373	-0.209976687	0.07338552	0.174305452
Rnf135	24	1.358637968	0.702413017	-0.656224951	0.07361979	0.174833588
Gpr146	62	1.376106849	1.637768117	0.261661268	0.07364274	0.174859806
Cdc37	22	1.379255951	1.44587221	0.066616258	0.07366109	0.174875083

Slc52a3	8	42.79739603	38.61541756	-4.181978461	0.07368175	0.174895833
AW822252	2	72.50990379	66.80556731	-5.704336485	0.07371506	0.174946586
Dhx37	51	1.940052352	2.329361186	0.389308834	0.07372787	0.174948693
Fabp5	10	4.189137263	3.607079501	-0.582057762	0.07380793	0.17511035
Mapk1	45	1.232382994	0.919151579	-0.313231414	0.07389598	0.175290926
Trrap	70	1.224564755	1.004662603	-0.219902152	0.07423661	0.17607047
Ift43	30	1.664550807	1.833781824	0.169231017	0.07428628	0.176131331
AA465934	13	0.655677912	1.545587198	0.889909285	0.07428023	0.176131331
Sytl4	4	13.21790293	9.064289433	-4.1536135	0.07432388	0.17619203
Ttll11	140	1.052663634	1.229203572	0.176539938	0.07437162	0.176276709
Mrps18c	3	1.268742791	1.856349513	0.587606722	0.07443033	0.176364568
Polr3d	24	1.089862018	0.7546199	-0.335242119	0.07443272	0.176364568
Capn5	24	6.335173126	4.73149743	-1.603675696	0.07448453	0.17645882
Ift27	24	2.130903508	1.35905514	-0.771848367	0.07453424	0.176548087
Blvrb	14	21.22191681	19.77055835	-1.451358451	0.07455989	0.176580345
700028E10F	29	1.038967699	1.37575563	0.33678793	0.07467633	0.17682758
Gne	44	1.226185188	1.508249327	0.282064139	0.07478166	0.177048407
Lef1	79	15.58329807	15.44106503	-0.142233042	0.07486884	0.17718702
Krtcap2	26	2.207644505	1.720628982	-0.487015523	0.07486307	0.17718702
Peo1	10	1.01712973	0.52685122	-0.49027851	0.07487643	0.17718702
Rnf14	54	1.680873901	1.403137582	-0.277736319	0.07505833	0.177560192
Nme7	4	0.577641862	0.105042017	-0.472599845	0.07504902	0.177560192
Degs2	8	12.66435655	15.53641633	2.872059775	0.07507738	0.177576634
Mettl16	11	2.775261228	1.898362913	-0.876898315	0.07515687	0.177735996
500011B03F	10	3.180514985	4.877400404	1.696885419	0.07527618	0.177989471
Zfp398	69	1.305536755	1.139823381	-0.165713374	0.07540071	0.178255196
Evx2	3	47.96645702	41.74461799	-6.221839028	0.07542693	0.178265947
Kdm3a	2	1.041666667	2.822580645	1.780913978	0.07542955	0.178265947
Timm22	15	1.547445109	1.081520279	-0.46592483	0.07548319	0.178335264
Kcng3	178	8.959434939	9.753345111	0.793910171	0.07548083	0.178335264
Olfr517	1	89.13043478	75.86206897	-13.26836582	0.07559754	0.178576686
Sgol1	51	1.579887682	1.135411002	-0.44447668	0.07570911	0.178811436
Slmo2	64	1.497616416	1.220183498	-0.277432918	0.07572936	0.178830481
Ccnt1	55	1.323584259	1.166387567	-0.157196691	0.07575758	0.17886835

Med6	11	2.1228431	1.559568631	-0.563274468	0.07577462	0.17887978
Usp34	117	1.495339982	1.293567805	-0.201772177	0.07591727	0.179158887
Alox12b	15	30.41704429	32.4187882	2.001743909	0.0759052	0.179158887
Slc39a4	8	14.40654644	12.02101927	-2.385527174	0.07598227	0.179283457
Zfp617	34	1.414514156	1.219122512	-0.195391645	0.07600844	0.179316366
Dhps	42	1.292913163	1.344008069	0.051094906	0.07609332	0.179487775
Lrrk2	25	1.392795001	0.871551468	-0.521243534	0.0762608	0.179853909
Mospd2	2	25.43673012	18.32868184	-7.108048285	0.07639152	0.180104302
Cnot2	72	1.08773901	1.095917573	0.008178563	0.0763873	0.180104302
Pxk	38	1.475619408	0.999539091	-0.476080317	0.07647441	0.180270756
Slc6a4	38	3.537581875	2.902467245	-0.63511463	0.07654037	0.180397262
Tamm41	31	1.190396909	0.739980736	-0.450416173	0.07656169	0.180418539
Prpf40b	58	1.588573386	1.708324247	0.119750861	0.07657436	0.180419426
Wsb1	39	1.883899714	1.575841734	-0.30805798	0.07671293	0.180716883
Parpbp	18	1.289432517	0.859319598	-0.430112919	0.07681831	0.180936103
Fam92a	58	2.063957777	1.613778745	-0.450179032	0.07686214	0.181010283
Nop14	8	2.477997587	1.893732741	-0.584264846	0.07698215	0.181263802
Tnpo1	121	1.062075301	0.763665275	-0.298410025	0.07716834	0.181673068
Syngr2	34	0.659165594	1.448642534	0.78947694	0.07719897	0.181716024
Crnde	65	1.682930686	1.38123837	-0.301692316	0.07723915	0.181781432
Dot1l	98	0.978002861	1.215638443	0.237635581	0.07734169	0.181993572
Adam6a	3	96.42857143	92.52434904	-3.904222393	0.0774579	0.182237822
'30455P16F	15	1.905409758	2.373173684	0.467763926	0.07748833	0.182280179
Rbm15	32	1.610044287	1.077584445	-0.532459842	0.07764827	0.182627138
Htt	7	2.68898995	0.908967576	-1.780022374	0.07767358	0.182657379
'00049E22F	4	36.20017656	32.84628064	-3.353895923	0.07769329	0.18267446
Pex19	2	2.216066482	2.864663422	0.64859694	0.07776979	0.182825049
Trim36	107	11.51307655	12.04131331	0.528236757	0.0777867	0.182835497
Cyb561d1	31	1.466917119	1.889892898	0.422975779	0.07799776	0.183302238
Papd4	43	1.544441262	1.065102488	-0.479338774	0.07809314	0.183496989
Slit2	39	1.558884989	0.999616549	-0.55926844	0.07814161	0.183581493
Caskin2	32	2.561396611	2.256351838	-0.305044773	0.0781727	0.183625139
Kat7	41	1.094874427	0.897410577	-0.19746385	0.07821681	0.183669938
Dtd2	9	0.661234191	1.149626949	0.488392759	0.07821348	0.183669938

Rpl7	74	1.174336282	1.380859212	0.206522931	0.07827073	0.183737752
Sec24d	36	0.702072572	0.956085823	0.254013251	0.07826255	0.183737752
Adam17	33	1.40521302	1.29448662	-0.1107264	0.07838517	0.183976976
Cds2	49	1.160014054	1.394944887	0.234930833	0.07848285	0.184176772
Baz1a	48	1.386104159	1.257714099	-0.12839006	0.07851034	0.184182367
Acp2	14	2.229820732	2.963524734	0.733704002	0.07850912	0.184182367
Psmd12	12	1.414013262	1.059700219	-0.354313043	0.07853672	0.184214817
Matn4	1	46.90721649	55.37190083	8.464684332	0.07868236	0.184526915
310062O18F	81	1.380690342	1.111459298	-0.269231044	0.07875879	0.18467664
Galk2	5	2.819168872	1.146403797	-1.672765075	0.07893029	0.18504921
Fus	37	1.709075495	2.024881343	0.315805848	0.07897488	0.185124195
D17H6S53E	11	1.509412395	1.430074901	-0.079337494	0.07904848	0.185267121
Mrpl48	102	1.456433955	1.293068531	-0.163365424	0.0791496	0.185474487
Serpina3n	2	12.01332637	21.15211521	9.138788846	0.07937195	0.185936144
Tgs1	31	1.551635475	1.162730097	-0.388905378	0.07936848	0.185936144
Haus4	32	1.303174383	0.860672885	-0.442501498	0.07940352	0.185959975
Ccdc85c	129	1.529181012	1.478641624	-0.050539388	0.07940747	0.185959975
Tfap2b	1	23.17073171	17.30205279	-5.868678921	0.07944853	0.186026452
Rab26os	2	4.355769231	3.873239437	-0.482529794	0.07948113	0.18607308
Agpat1	35	1.964636302	1.421982978	-0.542653324	0.07952901	0.186155484
Slc12a6	64	1.333565314	1.124496998	-0.209068316	0.07962445	0.186349148
Ppp1r3g	2	45.08547009	48.72532895	3.639858862	0.07975159	0.186616932
Tars2	13	2.266000127	1.489835127	-0.776164999	0.07990269	0.186910891
Zfp317	10	0.507469312	1.291796575	0.784327263	0.07989124	0.186910891
)10003K11F	4	45.65363026	40.08837644	-5.565253811	0.08003854	0.187198833
Rce1	102	1.419559546	1.076846414	-0.342713132	0.08016372	0.187461734
Polr3b	39	1.534040181	1.344103942	-0.189936239	0.08026922	0.187678553
Rnps1	32	1.181484861	0.855507518	-0.325977343	0.08028487	0.187685224
Apeh	51	1.823437353	1.675880246	-0.147557107	0.08040493	0.187935957
Srr	11	0.316539521	0.845321993	0.528782472	0.08045728	0.188028376
Murc	6	79.7262759	81.56826775	1.841991852	0.08051584	0.188135267
Irf1	64	1.032875981	0.809977407	-0.222898575	0.08056189	0.188212882
Hist1h1a	11	8.541306745	7.574136232	-0.967170513	0.08062194	0.188285561
Dnaic2	3	29.44514121	25.19254128	-4.25259993	0.08062417	0.188285561

Pkd1	36	1.110088016	0.870687202	-0.239400814	0.08063149	0.188285561
Ttpal	36	30.82295824	30.47615097	-0.346807273	0.08074338	0.188516848
Serinc3	40	1.283325197	1.087727875	-0.195597322	0.08084693	0.188698554
Atf6	17	1.34936668	1.835154731	0.485788051	0.08084527	0.188698554
Mir7034	12	95.59545566	94.74560846	-0.849847195	0.08096662	0.188947857
Dnajc21	83	2.383011931	2.27865031	-0.104361621	0.08105258	0.189006777
Psmg2	67	1.478876726	1.227331932	-0.251544794	0.08105627	0.189006777
Shisa5	25	1.825439326	1.224781288	-0.600658037	0.08103455	0.189006777
Cbfa2t2	49	1.392069914	0.880250666	-0.511819248	0.08102208	0.189006777
410006H16F	1	3.947368421	11.86440678	7.917038359	0.08103557	0.189006777
Lpcat3	66	1.720906314	1.25559099	-0.465315324	0.0812514	0.189431699
Wbp11	54	1.466014737	1.283410512	-0.182604225	0.08129174	0.189495625
Ndrg4	15	6.204841695	4.955814954	-1.249026741	0.0813965	0.189685203
Crtc1	36	1.711587558	1.659029423	-0.052558135	0.08139892	0.189685203
Cd2ap	62	1.100741054	0.913185237	-0.187555817	0.08142953	0.189726414
Gpr132	4	58.23820735	62.89458803	4.656380684	0.08155153	0.189980509
Ddx51	33	0.996332686	1.039709303	0.043376617	0.08188296	0.190722319
Bysl	18	1.388979129	1.506912874	0.117933745	0.0819903	0.190911709
Elovl7	59	1.669439419	1.407876012	-0.261563406	0.08197939	0.190911709
Pdk2	34	1.503788129	1.443662003	-0.060126127	0.08213601	0.19122067
Ost4	37	1.244073776	0.820330375	-0.423743401	0.08222046	0.191386906
430319G15F	43	1.491128866	1.915840408	0.424711543	0.08231423	0.191549856
Kdelc1	1	3.181818182	1.030927835	-2.150890347	0.08231657	0.191549856
Gm10069	7	2.857411439	4.483167607	1.625756168	0.08238953	0.191689219
Son	51	1.218214258	0.931407784	-0.286806473	0.08243662	0.191768375
Tuba1a	18	0.870724505	1.020246143	0.149521638	0.08258853	0.192091314
Phf2	188	1.366294038	1.213208838	-0.1530852	0.08262581	0.192147574
Pcdhgb2	5	65.08301313	59.49556317	-5.587449962	0.08272671	0.192321245
Lrtm1	4	89.6355472	86.38636	-3.2491872	0.08271865	0.192321245
Tcf12	178	1.056189636	1.104693502	0.048503865	0.08275579	0.192358378
Ccdc66	18	1.39059172	1.813487778	0.422896058	0.08284231	0.192528994
Dennd5b	67	3.112961661	3.249408738	0.136447077	0.08287323	0.19257035
Sycp3	28	92.70145046	90.91304543	-1.788405029	0.08296899	0.192762347
Chrdl1	12	31.27275625	36.46735225	5.194596004	0.08324683	0.193377235

Plekha7	73	1.370776711	1.501316378	0.130539667	0.08332946	0.193538551
C2cd2	48	1.056323039	1.239608943	0.183285905	0.08356255	0.194049197
Clpb	31	1.3665711	1.082273219	-0.284297881	0.08382989	0.194639206
Gp1bb	4	48.87755102	49.38763621	0.51008519	0.08392027	0.194818238
Hspg2	31	2.354540437	2.664390554	0.309850117	0.08400265	0.194978631
Mcm4	34	1.398669386	1.114419146	-0.28425024	0.0841098	0.195165591
510010F05F	103	1.137015827	1.213641798	0.076625971	0.08409951	0.195165591
Atxn3	15	1.392997305	0.731539802	-0.661457503	0.0842279	0.195408732
Pcdh7	24	55.54875907	54.21838546	-1.33037361	0.08424473	0.195416878
Dock11	52	13.97628691	13.27767402	-0.69861289	0.08431077	0.195539156
Mtm1	5	19.90908724	16.55644286	-3.35264438	0.08443127	0.195739781
Calhm2	7	58.78844706	55.79538435	-2.993062711	0.08442503	0.195739781
Tmem259	80	1.165735052	1.133094688	-0.032640364	0.08443729	0.195739781
Exoc3	2	2.272727273	0	-2.272727273	0.08446204	0.195766226
Isoc2a	5	1.30914974	1.989285086	0.680135346	0.08447651	0.195768849
Map4k2	56	1.064837362	1.090380252	0.02554289	0.08452401	0.195817082
Slc5a3	35	1.610090436	1.758516752	0.148426316	0.08452265	0.195817082
Gm561	16	1.667716526	1.202998717	-0.464717809	0.08457929	0.195914208
Chmp6	34	2.789587159	2.285667994	-0.503919165	0.08469933	0.196161301
Cwf19l2	1	0	1.063829787	1.063829787	0.08477894	0.196314682
Ambra1	22	0.697299977	0.9444824	0.247182423	0.08494038	0.196657482
Kdm7a	112	1.024497665	0.834690975	-0.18980669	0.08507547	0.196924979
Adgrg6	95	1.952795413	1.828514249	-0.124281164	0.08508276	0.196924979
Lrrc16b	60	2.134685625	2.16646043	0.031774805	0.08512601	0.196994013
Ercc8	23	1.241713967	1.6552733	0.413559333	0.08521004	0.197157389
Ntn3	50	14.04529999	15.75327956	1.707979567	0.08531979	0.197380205
Slc25a30	52	1.478660209	1.39414378	-0.084516429	0.08540681	0.197550363
Hmces	35	0.981685069	0.873716787	-0.107968282	0.08550398	0.197743957
Cmtm4	73	1.439024599	1.216425424	-0.222599175	0.08587337	0.198536563
Exosc8	89	1.329096617	1.179718879	-0.149377738	0.08587376	0.198536563
Suclg1	25	2.565128811	2.243983302	-0.321145509	0.08594253	0.19866425
Mid1	18	17.17272505	14.58830394	-2.584421107	0.08597259	0.198702433
Atp13a1	59	1.102645405	1.191279773	0.088634368	0.08603084	0.198805752
Ankrd24	3	5.497325916	9.294278863	3.796952947	0.08609803	0.198929692

Dhrs4	1	7.777777778	1.724137931	-6.053639847	0.08614969	0.199017716
310041H14F	38	2.210184996	1.589802372	-0.620382624	0.08620089	0.199042003
H19	7	47.85801837	51.95638915	4.09837078	0.08618191	0.199042003
Atp6v1f	18	2.609923938	1.790464115	-0.819459823	0.08619211	0.199042003
Sv2b	18	25.79956787	27.83312069	2.033552827	0.0863124	0.199268126
Apc	79	1.514520663	1.306640993	-0.20787967	0.08640645	0.199453862
230219D22F	66	0.943991618	0.999137664	0.055146046	0.08646688	0.199530592
Amer2	27	13.89302496	21.40463781	7.511612843	0.08645456	0.199530592
Foxp4	66	0.907162256	0.935707324	0.028545068	0.08648068	0.199531064
Slc47a2	35	10.72085816	10.62984007	-0.091018091	0.08660377	0.199783646
Sigmar1	15	1.046559086	1.544969316	0.498410229	0.08665141	0.199862125
Tm7sf2	8	1.491956736	0.558862233	-0.933094503	0.08668137	0.199899792
Ppp2r1b	42	1.652602105	1.249250851	-0.403351253	0.08676079	0.200051525
Hcn2	103	2.784485584	2.338221552	-0.446264032	0.08679909	0.200108376
Tor1aip1	31	1.058471105	0.693214956	-0.365256149	0.08683613	0.200155741
Klc3	36	2.119004363	1.793643602	-0.325360762	0.08686055	0.200155741
Ankrd50	51	1.356287902	1.176735924	-0.179551978	0.08685075	0.200155741
Crtac1	109	23.29896846	24.35798292	1.059014459	0.08688831	0.20016648
Rnf6	46	1.130445164	1.330684485	0.200239321	0.0868925	0.20016648
10065P20F	42	1.679473369	1.116530488	-0.562942881	0.08694235	0.200218471
Mrpl39	58	1.248763683	1.494370347	0.245606664	0.08693726	0.200218471
Crem	58	0.784452983	1.055754753	0.27130177	0.08698255	0.200279596
Mir8109	126	2.105323887	2.395849041	0.290525154	0.08700553	0.200301085
Rrn3	50	1.100756932	1.100732926	-2.40E-05	0.08704892	0.200369547
Gfra2	35	42.53776933	40.01246811	-2.525301216	0.08706429	0.200373498
Dctd	41	4.665768207	4.46903647	-0.196731737	0.08719094	0.20063351
Steap2	84	1.656475954	1.425397863	-0.231078091	0.08728863	0.200826809
Akap13	37	1.15898255	0.885392008	-0.273590542	0.08732507	0.200879138
Grik2	17	36.3617134	37.65012093	1.288407524	0.0874079	0.201006659
Irf7	4	2.169058638	2.845539299	0.676480661	0.08739823	0.201006659
Mvd	19	1.840910925	1.34889376	-0.492017164	0.08744089	0.201051016
Tmem63a	43	1.740262626	1.342078805	-0.398183821	0.08745755	0.201057824
Srm	66	1.12154404	1.41392562	0.29238158	0.08747459	0.20106549
430440P10F	6	76.07448573	79.82394077	3.749455047	0.08750165	0.201096205

Tmem161b	20	1.744722324	1.188869609	-0.555852715	0.08759318	0.201275034
Kcnmb2	4	51.21334877	49.83157993	-1.381768836	0.08770358	0.201466351
St6galnac6	45	9.434835844	8.903456374	-0.53137947	0.08771763	0.201466351
Lyrm2	5	3.021291078	2.266190719	-0.755100359	0.08771445	0.201466351
1-Mar	5	55.99845275	58.41175898	2.413306229	0.08773479	0.201474217
Lefty1	5	94.05012571	89.25423856	-4.795887143	0.08779716	0.201585914
Ncapd3	5	1.124530201	2.89218203	1.767651828	0.08784376	0.201598269
Tmco6	22	2.248070381	3.000340289	0.752269908	0.08783461	0.201598269
Trim3	55	1.244677722	0.95244766	-0.292230062	0.08782687	0.201598269
Cul4a	126	1.094074809	1.30906871	0.214993901	0.08791269	0.201724909
Kif1c	67	1.147768961	1.286655685	0.138886724	0.08800786	0.201911717
Emx2	1	54.01234568	47.84482759	-6.167518093	0.08828799	0.202522746
Fat1	1	95.38461538	98.52941176	3.14479638	0.08830271	0.202524843
Erf	52	1.90944638	1.612100102	-0.297346278	0.08835557	0.202614419
Atg2b	55	1.4859835	1.100644192	-0.385339308	0.08846899	0.202811105
Actl6a	27	1.466998432	0.924051597	-0.542946835	0.08846171	0.202811105
Apob	2	88.37209302	93.69462025	5.32252723	0.08855899	0.202954027
Mir760	95	1.174052037	0.973014624	-0.201037413	0.08855204	0.202954027
Mrps11	6	1.646129212	3.440252948	1.794123736	0.08869377	0.20323117
Bcor	71	15.23037119	15.83999338	0.609622184	0.08871314	0.203243803
Bdp1	22	1.096157474	1.447355339	0.351197865	0.08872905	0.203248538
Ccer1	4	91.09267583	93.16875653	2.076080698	0.08878835	0.203352632
Mib1	30	1.374188478	1.902939904	0.528751426	0.08893844	0.20366458
Stk24	91	1.236553228	1.454919299	0.218366071	0.08896695	0.203698093
Dzip1	57	13.94797747	15.84357726	1.895599789	0.08906633	0.203893809
Camk2n1	145	1.688812263	1.417714897	-0.271097365	0.08912389	0.203993766
Tefm	17	1.967833472	1.464916568	-0.502916904	0.08929099	0.204312479
L3hypdh	38	3.057331975	2.674832513	-0.382499463	0.08928446	0.204312479
Mir6957	7	86.4051191	83.81708443	-2.588034667	0.08950201	0.204763406
310403D21F	5	14.94299789	11.6115007	-3.331497188	0.0895279	0.20479071
Cycs	13	1.602330907	1.832197637	0.22986673	0.08959336	0.204908499
Ppp1r14b	48	0.940516824	1.081230973	0.14071415	0.08967166	0.205055634
Rttn	3	3.144654088	1.58045977	-1.564194318	0.08970534	0.20510069
Vars	82	1.250533637	1.026937971	-0.223595665	0.08981567	0.205316692

10007C09F	66	1.241925718	1.065975541	-0.175950177	0.0898278	0.205316692
Fbxo8	17	1.227401421	1.24222675	0.014825329	0.08984721	0.205329078
Map1lc3a	62	1.342086601	1.097925305	-0.244161296	0.08993308	0.205461304
300005D01F	3	5.108032537	2.741256686	-2.366775851	0.08992786	0.205461304
Met	44	1.632514765	1.133247344	-0.499267422	0.08995157	0.205471558
Loxl1	1	73.86363636	62.80991736	-11.05371901	0.09002313	0.205603017
Ppp1r7	58	1.531800781	1.38729053	-0.14451025	0.09004	0.205609543
Usp2	73	1.963886238	1.610269642	-0.353616596	0.09008097	0.205671099
Mylk3	2	84.14984472	76.3830945	-7.766750217	0.09018794	0.205851266
Mir5122	10	2.87070456	2.159329253	-0.711375306	0.09017806	0.205851266
Lrrc57	21	1.446836411	1.520880463	0.074044052	0.09023099	0.205917496
Bbs2	7	1.975107617	2.125524539	0.150416922	0.09029487	0.205967191
Cdkn2aip	124	1.150363919	1.228429023	0.078065104	0.09028878	0.205967191
Klc2	49	2.887031172	1.705366617	-1.181664556	0.09028231	0.205967191
Mir6980	6	87.77239213	81.48962908	-6.282763049	0.09036275	0.20609
Akirin2	98	1.200082465	0.987473549	-0.212608916	0.09039114	0.206122697
Smim19	77	1.035175384	0.883176429	-0.151998954	0.09042474	0.206167282
Etv5	53	1.09738814	1.111361518	0.013973378	0.09052734	0.206366108
Nfat5	84	1.460409437	1.167183451	-0.293225986	0.09055036	0.206366108
Atp10a	40	1.307062869	0.933783209	-0.373279661	0.09056493	0.206366108
Slc22a5	42	1.18962958	0.997723527	-0.191906052	0.0905682	0.206366108
Cdh3	38	33.45507291	33.16805473	-0.287018175	0.09062638	0.206441575
Sfxn5	4	3.342293017	1.702251052	-1.640041965	0.09062946	0.206441575
Cilp2	9	13.12823961	12.33542559	-0.792814022	0.09071379	0.20656954
Anapc7	38	1.267987416	1.687510888	0.419523471	0.09070849	0.20656954
Igsf1	2	28.61827227	19.11375661	-9.504515661	0.09076562	0.206655506
Epb4.1l3	79	50.08011892	50.91692911	0.836810183	0.09083924	0.206791025
Pdss2	46	1.145913273	1.02785147	-0.118061803	0.09091621	0.206934148
Cep55	20	1.708741344	1.113616831	-0.595124514	0.09103287	0.207167535
Anxa11	48	1.698885366	1.658675452	-0.040209913	0.09105171	0.207178288
Ccdc85a	7	36.9147188	39.58027951	2.66556071	0.09114279	0.207353389
Lrp5	129	3.00031692	2.82415755	-0.176159371	0.09120817	0.207469954
Ceacam16	5	90.08915193	87.65727958	-2.431872348	0.0912739	0.207587294
Srprb	10	0.984770764	2.381269138	1.396498374	0.09139068	0.207820687

Dda1	62	0.976406321	1.09485911	0.118452789	0.09164509	0.208308898
Slc5a1	9	39.21371435	37.43920174	-1.774512614	0.09164704	0.208308898
Taf13	25	2.113533287	1.336241049	-0.777292237	0.09164797	0.208308898
Aox1	1	31.19266055	21.64179104	-9.550869506	0.09171112	0.208420152
Znrf4	43	82.4129023	80.41089325	-2.002009048	0.09179949	0.208588693
Tpgs1	53	1.312534686	1.025024619	-0.287510067	0.09184159	0.208652035
Gm10373	10	2.016478083	1.607253018	-0.409225065	0.09201673	0.209017569
Rbsn	12	1.123026013	1.790851712	0.667825698	0.09209409	0.209160915
Trmt6	7	2.479079558	1.83418566	-0.644893899	0.09214108	0.209235264
Hras	57	1.543770178	1.166585134	-0.377185045	0.09217017	0.209268933
Ppih	27	1.969961505	2.110218536	0.140257031	0.09222915	0.209370432
Nrp1	10	1.580519217	0.538843495	-1.041675722	0.09230526	0.209510799
Fhl3	47	1.740914449	1.295759527	-0.445154922	0.09235787	0.209597795
Topbp1	52	1.392848845	1.067586796	-0.32526205	0.09244792	0.209769724
Tdh	12	27.2204909	27.76989841	0.549407508	0.092467	0.20978057
5-Mar	83	1.35303046	1.370195561	0.017165102	0.0925129	0.209852258
Rtca	51	1.448382645	1.592828741	0.144446096	0.09258728	0.209988518
Zfp874b	1	8.888888889	20.68965517	11.80076628	0.0926141	0.210016902
H2afy3	9	98.78914652	97.81443956	-0.974706962	0.09281718	0.210444885
Akr1a1	24	1.851288122	1.280034466	-0.571253655	0.09302759	0.210889372
Phgdh	19	2.74945903	2.565081617	-0.184377413	0.09322446	0.211303012
Tmem151a	57	2.201180981	1.580664587	-0.620516394	0.09325712	0.211344393
Pigv	65	1.22571335	1.326618059	0.100904709	0.09337332	0.211575051
310029C07R	27	1.81488649	1.312774534	-0.502111956	0.09338882	0.211577523
Pik3r4	30	2.624255387	2.082118473	-0.542136914	0.09344286	0.211667258
Pdgfra	18	2.256178521	1.91180694	-0.344371581	0.09365042	0.212104677
Mgat4a	81	1.646277067	1.281521976	-0.364755091	0.0937291	0.212250115
Prps1l1	3	87.79342723	93.20621992	5.412792689	0.09394959	0.21271661
Fbxo25	59	1.318064602	1.011137997	-0.306926605	0.09419052	0.213196322
Dfna5	20	2.044457267	2.913533092	0.869075824	0.09418825	0.213196322
Flywch1	32	1.47722821	0.971115251	-0.506112958	0.09420953	0.213206459
Gm3336	8	12.27627637	13.93108542	1.654809053	0.09427567	0.213323233
Cldn15	2	97.27066412	93.54596935	-3.724694765	0.09445755	0.213668901
Trmt112	39	1.273073368	0.891519195	-0.381554173	0.09445113	0.213668901

Bckdha	1	4.511278195	0.99009901	-3.521179186	0.09475423	0.214306974
Inpp5a	89	1.771462819	1.630749114	-0.140713705	0.09487571	0.214548654
Foxs1	19	74.11353325	72.64482558	-1.468707669	0.09492565	0.214628498
Slc2a1	33	1.524684032	1.064298285	-0.460385748	0.0950376	0.214848529
Uchl5	82	1.251920726	1.425749669	0.173828942	0.09522695	0.215243418
Ap4e1	23	0.849749205	1.278440608	0.428691403	0.09530194	0.215379752
Ppp3ca	92	1.541039958	1.423958964	-0.117080995	0.09535112	0.215457694
Mxd1	115	1.375422973	1.323034798	-0.052388175	0.09553309	0.215816533
Fam219a	69	1.746468216	1.344910876	-0.40155734	0.09553982	0.215816533
Ccdc110	4	10.26865501	7.562263689	-2.70639132	0.09555404	0.215816533
Zfp712	2	0.27173913	1.171525276	0.899786145	0.09577874	0.216290729
Socs3	43	0.938637932	1.261108095	0.322470163	0.09589993	0.216531096
Ggnbp1	26	29.6631672	26.50922357	-3.153943635	0.09592617	0.216557016
Carns1	2	32.21749226	27.96897275	-4.248519514	0.09605749	0.21676274
310408I11R	98	1.446664513	1.21140931	-0.235255203	0.09606161	0.21676274
Pced1a	38	1.346373709	1.397525702	0.051151993	0.09603638	0.21676274
330023K18F	15	2.28901779	1.872100028	-0.416917762	0.0962549	0.21716549
Vprbp	20	0.568096115	0.985373518	0.417277403	0.09632005	0.21727907
Pus1	18	1.228229784	0.849616553	-0.378613232	0.09635723	0.217329547
Zfp808	10	1.137771701	2.355403951	1.21763225	0.09642362	0.217445863
Hist1h2bh	4	1.194852941	1.943895838	0.749042897	0.09647785	0.217534711
Pik3c2b	59	0.966375534	1.191430331	0.225054797	0.09670408	0.218011322
Intu	28	1.956620508	1.679812396	-0.276808113	0.09675875	0.218101066
Cdyl2	127	1.526305155	1.600158578	0.073853423	0.09680457	0.218170837
Lonrf1	75	1.143502227	0.975555227	-0.167947	0.0969601	0.218454255
Slc6a13	2	35.41437779	31.44673484	-3.967642955	0.09694587	0.218454255
Gm5577	37	5.117913085	5.351852602	0.233939517	0.09701178	0.218537127
Ippk	53	1.443204232	1.147854399	-0.295349833	0.09705355	0.218597656
Rtp4	3	3.840877915	2.404978631	-1.435899284	0.0971419	0.218763081
Itm2c	24	0.832686578	0.976222307	0.143535728	0.09718366	0.218823539
Parn	53	8.534225291	7.656194968	-0.878030323	0.09731217	0.219069748
Mfap4	4	76.66628959	73.34444698	-3.321842615	0.09733375	0.219069748
Mettl14	7	2.370329643	1.298460902	-1.071868742	0.09733779	0.219069748
Firre	3	19.06607376	12.14746372	-6.918610045	0.09741254	0.219170755

Fbf1	22	1.181843419	0.709228851	-0.472614568	0.09741165	0.219170755
Ino80	56	1.475397874	1.180364687	-0.295033187	0.09756963	0.219490537
Rp1l1	7	89.80397757	91.27773478	1.473757208	0.09768428	0.219714765
Pdcd2	67	1.481201469	1.17912282	-0.302078649	0.09770792	0.219734245
30309D14F	50	35.65028275	37.36888541	1.718602664	0.09775463	0.219805623
Mettl21b	27	2.682481272	2.307650254	-0.374831017	0.09777553	0.219818919
Polr3gl	16	1.177247848	1.985638254	0.808390406	0.09786715	0.219991193
Acaa1a	25	1.914882282	1.339670315	-0.575211967	0.09792066	0.220077768
Mkrn1	12	1.796957203	2.322816628	0.525859425	0.09805801	0.220352712
Ankle2	59	0.996393967	1.114970718	0.118576751	0.09817575	0.220583513
Ccdc117	57	1.258380896	1.187146714	-0.071234182	0.09825833	0.220735253
Eea1	71	1.148491725	1.322809306	0.174317581	0.0984248	0.221075384
Angpt4	2	15.08578431	21.39285714	6.307072829	0.09846424	0.221106491
Lin7c	59	0.964428771	1.377231391	0.41280262	0.09846878	0.221106491
Tjap1	8	68.23274434	66.50599065	-1.726753688	0.09850737	0.221159298
Atf2	19	1.6656161	1.692158851	0.026542751	0.09864529	0.221411166
Dnajc14	45	1.283941038	1.098652987	-0.185288051	0.09864973	0.221411166
Pdhx	5	1.643835616	3.638583964	1.994748348	0.09868112	0.221413884
S100g	1	71.42857143	79.41176471	7.983193277	0.09867476	0.221413884
Ift140	34	1.525648852	0.939098071	-0.586550781	0.09870457	0.221432629
Zfp513	30	1.658074246	1.277860486	-0.380213761	0.09879267	0.221496879
Samd4	74	4.424699432	4.209502476	-0.215196956	0.09879359	0.221496879
Birc2	20	1.685034457	1.202558402	-0.482476054	0.09876694	0.221496879
Chl1	1	43.33333333	56.52173913	13.1884058	0.09876138	0.221496879
Gns	47	1.597667561	1.200541311	-0.39712625	0.09896533	0.221848037
Rnf122	31	2.611003563	2.386145839	-0.224857724	0.09899743	0.221886088
Pllp	36	2.987429427	3.815283966	0.82785454	0.09902992	0.221925015
Pus10	22	1.254490582	1.500974454	0.246483873	0.09913409	0.222124553
Acyp2	8	8.480952995	7.677120594	-0.803832401	0.09918626	0.22220751
Cnp	50	9.959841588	9.455748995	-0.504092592	0.09934732	0.222534366
532427E13F	19	1.919926574	1.109008154	-0.81091842	0.09938249	0.222579168
Bet1	11	1.084597243	2.109690428	1.025093185	0.09944953	0.222695317
Armt1	27	1.282309444	1.454332938	0.172023494	0.0994748	0.222717909
Llgl2	57	1.279328948	1.030418812	-0.248910136	0.09956261	0.22288052

Rsrc2	16	0.545259581	1.151802955	0.606543374	0.09959601	0.222921269
Abhd11	8	1.462183852	2.41022658	0.948042728	0.09977081	0.22327846
Gm16576	34	1.37590799	1.30129833	-0.074609659	0.09989916	0.223531607
Vps37c	44	1.38504606	1.136682021	-0.248364039	0.09993849	0.223585526
Gpc3	35	36.4019885	37.92433928	1.522350786	0.09998363	0.223652405
310001H17F	3	0	1	1	0.10000858	0.223674129
Usp10	65	0.992289575	0.633983731	-0.358305844	0.10008022	0.223800231
Cmc1	48	16.69534918	16.76506635	0.069717168	0.10024942	0.224144445
Banf1	75	1.421537957	1.270318983	-0.151218974	0.10031526	0.224245225
Cdk5rap2	36	1.411904214	0.879751456	-0.532152758	0.10032506	0.224245225
Adat1	11	1.101190051	2.014234742	0.913044691	0.10079108	0.22525257
Yeats4	28	0.733254616	0.828688274	0.095433658	0.10093398	0.22553756
Zfp933	1	5.381165919	2.469135802	-2.912030117	0.1009841	0.225615205
Zfp575	3	55.25906736	59.61035393	4.351286572	0.10104389	0.225714412
Slc4a8	61	1.614786915	2.000183638	0.385396723	0.10111293	0.225834272
Nup37	24	1.338661834	0.839853787	-0.498808047	0.10128653	0.226153148
Plekhj1	59	1.204754575	1.265717831	0.060963256	0.10127986	0.226153148
Stk16	25	0.899906598	1.411862629	0.511956031	0.10139464	0.226360098
Prmt3	68	0.780739196	0.848307168	0.067567972	0.10149244	0.226543975
Ifitm7	7	11.10577564	15.17539236	4.069616721	0.10161601	0.226785288
Fancf	25	1.146619823	0.747135377	-0.399484446	0.10167117	0.226873896
Rhov	58	5.626617588	5.735350618	0.108733031	0.10168986	0.226881095
Itgb4	45	2.186900816	1.609786892	-0.577113924	0.10172767	0.226930964
Cct8	25	1.197533969	1.34885387	0.151319901	0.10202641	0.227562785
Mthfsl	1	2.985074627	9.302325581	6.317250955	0.10207839	0.227644125
Dtx3l	18	2.252902423	1.69554075	-0.557361673	0.10238779	0.228264736
Ftsj2	5	0.870519044	0.5627416	-0.307777444	0.10238084	0.228264736
Enpp1	30	1.610030108	0.784230908	-0.8257992	0.10246203	0.22839554
Ddx55	59	1.032940904	1.186519254	0.15357835	0.10261697	0.228706163
Myo10	69	1.745780043	1.378853756	-0.366926287	0.1026402	0.228723207
Rmi2	45	1.099089761	1.146640604	0.047550843	0.10265956	0.228731611
Ncoa7	79	1.466339037	1.2995788	-0.166760238	0.10271303	0.228815996
Bmp1	63	3.780805567	3.432300081	-0.348505485	0.10275253	0.228869242
Pabpc4	36	1.976527224	2.113237021	0.136709797	0.1030044	0.229395445

Gpam	77	1.733230072	1.4134498	-0.319780272	0.10302044	0.229396339
Rab5a	101	1.36226005	1.017487069	-0.344772981	0.10304791	0.229422702
Fnip2	94	1.072627044	1.208924555	0.13629751	0.10308007	0.229459481
130218I03R	9	96.69707716	96.24288742	-0.454189743	0.10322136	0.229739146
Ccdc87	11	2.368451883	1.572024207	-0.796427676	0.10329062	0.229858439
00002H07F	10	2.123452338	1.32282218	-0.800630157	0.10339983	0.230031695
Foxo1	76	1.407212727	1.375182861	-0.032029866	0.10338624	0.230031695
Rint1	19	1.730156692	1.301018681	-0.429138011	0.10345178	0.230041771
Relt	53	1.072388008	0.671174413	-0.401213595	0.10346056	0.230041771
Timm8b	18	1.407019377	1.047476789	-0.359542588	0.10346706	0.230041771
Bmi1	137	1.268430411	1.465565684	0.197135273	0.10342921	0.230041771
Mrpl28	15	0.728905597	0.867630225	0.138724627	0.10356236	0.230158752
Rps19	74	4.910031407	4.692494026	-0.217537381	0.10354988	0.230158752
Papl	29	17.59297351	17.76207784	0.169104331	0.10356673	0.230158752
Pcdh20	11	39.03357641	42.05719361	3.023617209	0.1035982	0.230193814
Secisbp2	53	0.954156373	0.63286404	-0.321292333	0.10363638	0.23024379
Dip2a	90	1.483190015	1.111076013	-0.372114002	0.10366908	0.230281581
Wdr33	17	0.796674444	1.149106818	0.352432373	0.10371616	0.230351281
Ssc4d	3	67.23646724	71.19927388	3.962806644	0.10387509	0.230669359
Nudt17	29	2.79604758	2.056910018	-0.739137562	0.10393142	0.230759515
Ovca2	44	2.562284223	1.893500509	-0.668783714	0.10396018	0.230788446
Clasp1	12	1.668279093	1.322719858	-0.345559235	0.10399377	0.230828099
Il6ra	76	7.002082827	5.842385184	-1.159697643	0.10402276	0.230836926
Epha2	46	1.384873639	1.349348739	-0.0355249	0.10402921	0.230836926
Nxt2	15	31.41415967	33.86377808	2.449618407	0.10407001	0.230892543
Myl12a	48	2.095254865	1.752960968	-0.342293897	0.10408663	0.23089452
Whrn	39	2.258857255	1.732932753	-0.525924502	0.1042015	0.231113142
Zfp341	75	1.79152082	1.376218141	-0.415302679	0.10421669	0.231113142
Henmt1	27	35.86700593	37.01823734	1.151231404	0.10442808	0.231546932
Foxp1	49	5.465449767	5.639726966	0.174277199	0.10445041	0.231561452
Usp28	94	1.547220968	1.351323972	-0.195896996	0.10469795	0.232075185
Camkk1	80	2.124327072	1.734279172	-0.3900479	0.1047234	0.232096531
Fosl2	72	0.981216325	1.147222981	0.166006656	0.10477639	0.232178921
10403A07F	27	1.75452252	1.32208986	-0.43243266	0.10506669	0.232787036

Ttc41	17	11.66957654	11.98940125	0.319824705	0.10511497	0.232823715
Mrto4	19	1.328567597	1.018769305	-0.309798292	0.10510543	0.232823715
Wrn	84	1.497635738	1.334590793	-0.163044945	0.10514727	0.232860096
Ndufb8	27	1.381521349	1.267443398	-0.114077951	0.10518307	0.232904238
Sf3b5	18	1.490239273	0.845547697	-0.644691576	0.10527343	0.233069143
Pqlc3	25	2.358825734	1.941269119	-0.417556615	0.10529736	0.233086971
Zc3h12a	44	1.507316799	1.090300961	-0.417015837	0.10532765	0.233118855
Tmem177	22	2.48530331	2.362600708	-0.122702602	0.1054091	0.233263929
Polg	98	1.279588367	2.073500087	0.79391172	0.10544843	0.233284955
Tmem63b	41	1.437592377	1.094957595	-0.342634782	0.1054504	0.233284955
Ermp1	73	1.106811293	0.991149027	-0.115662266	0.10552949	0.233424752
Krcc1	43	1.346574716	1.522317374	0.175742658	0.10556224	0.233461982
Zbtb11	81	1.085908058	1.173586629	0.087678571	0.1055995	0.233509211
Mtss1l	91	3.54240897	3.12105849	-0.42135048	0.10564955	0.233584666
Ier2	32	1.660198419	0.98975243	-0.670445989	0.10573	0.233692115
Pkn1	93	1.296040686	0.963929053	-0.332111633	0.10572453	0.233692115
Snap23	24	1.617569376	1.05727615	-0.560293226	0.10583874	0.233897232
Tsc22d2	191	1.233032283	1.170507271	-0.062525012	0.1058737	0.233939277
Dirc2	76	1.459510078	1.385145422	-0.074364656	0.10602698	0.234242686
Lats2	105	0.94653025	1.001314859	0.05478461	0.10604855	0.234255065
Dixdc1	28	0.838346383	1.333359585	0.495013202	0.10622043	0.234599431
933427E11R	24	2.265334023	2.159175162	-0.106158861	0.10628171	0.234634625
Ppp1r21	65	1.328696309	1.498170864	0.169474555	0.10625792	0.234634625
Hadh	30	1.536650378	1.46563574	-0.071014638	0.10628434	0.234634625
Nab2	49	1.710283194	1.417419622	-0.292863572	0.1063179	0.234660245
Krtap16-1	6	81.51782453	83.68075397	2.162929439	0.10632792	0.234660245
Ddx11	12	0.612382	0.232774674	-0.379607325	0.10640047	0.234785042
Commd6	13	1.890869852	1.244823921	-0.646045932	0.10644551	0.234849115
Itpkc	35	7.045449139	5.265790926	-1.779658213	0.10666964	0.235308222
Arfip2	17	1.647544218	1.509776713	-0.137767505	0.1068288	0.235623893
Aldh18a1	33	2.46042314	1.955483874	-0.504939267	0.10689577	0.235700762
Dctn6	5	0.829493088	1.799242424	0.969749337	0.1068845	0.235700762
Bgn	5	73.62441202	77.55491228	3.930500261	0.10700015	0.235895458
Csf1	30	0.900886953	0.951650921	0.050763968	0.10702329	0.235911034

Hoxc11	31	24.51571075	25.51328627	0.997575517	0.10710307	0.236051428
Wdr74	20	1.970610415	2.179358041	0.208747626	0.10719813	0.236225456
Mir6988	3	89.70355436	91.41706924	1.713514882	0.10727041	0.236349251
Mir713	5	6.561003861	6.01441867	-0.546585191	0.10738802	0.236572851
Ankrd46	37	1.414055315	1.551265025	0.13720971	0.10745387	0.236682382
Nedd9	6	1.997476871	0.31152648	-1.685950392	0.10752973	0.236797705
Stap2	4	19.03567447	15.52860198	-3.507072492	0.1075385	0.236797705
Gm13034	16	70.80720615	70.93607598	0.128869826	0.10764431	0.236995139
Phlpp1	98	1.133218602	1.024902295	-0.108316307	0.10766856	0.237012958
Frmd4a	43	1.812896959	1.530978602	-0.281918356	0.10772481	0.237101228
Git2	81	1.340558936	1.037708679	-0.302850257	0.10775743	0.237137455
Ubxn8	43	1.609578036	0.994559352	-0.615018684	0.10778267	0.237157438
Pdap1	48	0.974534945	1.227823165	0.25328822	0.10782659	0.237218498
Hmha1	7	12.00971304	10.55644822	-1.453264817	0.10792464	0.237398624
Atf7ip2	54	23.04200984	22.27687688	-0.765132957	0.10797403	0.237471658
Ndufb6	22	1.364310097	1.812040046	0.447729948	0.10807491	0.237657908
Phf19	78	1.712600657	1.383521372	-0.329079285	0.10824233	0.237990393
Gpr108	18	2.368477293	2.187586448	-0.180890845	0.10828457	0.238047604
Rnasek	2	3.977272727	0.961538462	-3.015734266	0.10834482	0.238073068
Tpd52	69	1.24496036	0.916803698	-0.328156662	0.10833658	0.238073068
Zkscan17	50	2.838764214	2.189163872	-0.649600342	0.10831528	0.238073068
Sbds	9	1.153085348	2.475482025	1.322396678	0.10839785	0.238118271
Etv6	60	0.972391916	0.806798327	-0.165593589	0.10838706	0.238118271
Ypel3	40	1.388179619	1.071577778	-0.316601841	0.10853166	0.238376517
Slc6a6	42	0.813904285	0.896827301	0.082923016	0.1085661	0.238416492
Cgn	25	1.419110203	1.427469449	0.008359246	0.10858619	0.238424921
Sik2	104	1.788047119	1.346250023	-0.441797097	0.10860572	0.238432132
Prdx2	37	1.341432561	1.658641087	0.317208526	0.10898992	0.2392398
[illegible]32438H23[illegible]	8	63.29960274	60.28251628	-3.017086455	0.10901121	0.239250739
Morn5	9	2.916212819	1.56592895	-1.350283869	0.10908861	0.239349017
Mbnl1	98	1.203615033	1.032211898	-0.171403135	0.10908829	0.239349017
Pabpc1l	7	96.61036259	94.28673725	-2.32362534	0.10919605	0.239513129
Kcnh6	23	3.346400297	2.8159578	-0.530442498	0.10919406	0.239513129
Clp1	19	1.314386438	0.893439383	-0.420947055	0.10928154	0.239664823

Med8	30	1.753188659	1.341241557	-0.411947102	0.10951343	0.240101599
Slc25a38	47	1.791754681	1.605251886	-0.186502795	0.10949732	0.240101599
Dnajc10	48	1.317763956	1.240348369	-0.077415587	0.10958662	0.240226192
Cd38	2	8.374183007	4.938271605	-3.435911402	0.10982216	0.240699593
Fam3a	13	15.03733862	17.29334926	2.256010646	0.10983539	0.240699593
Creb3l2	23	1.03465045	0.835784086	-0.198866364	0.10998199	0.240984872
Ccdc173	17	0.843775779	1.324586848	0.480811068	0.11002636	0.24099872
Mir3093	35	31.41920513	34.11267486	2.693469731	0.11003758	0.24099872
Ccdc162	1	98.13084112	95.6043956	-2.526445517	0.11003715	0.24099872
Ankrd32	44	1.160833865	1.359575247	0.198741382	0.11006001	0.241011885
Abhd16b	6	93.84383579	93.84545682	0.00162103	0.11010754	0.241079978
Ffar2	2	49.0899042	42.39372048	-6.696183723	0.11015823	0.241154983
Ky	37	44.11189911	43.15894438	-0.952954733	0.11023562	0.241288406
Tbcd	20	1.241273451	1.175938171	-0.06533528	0.11025684	0.241298856
Mok	30	1.962444197	1.46710414	-0.495340057	0.11032029	0.241329751
Sqstm1	11	1.069495886	2.406078759	1.336582873	0.11031142	0.241329751
BC024978	50	1.24580391	1.004911669	-0.240892241	0.11030939	0.241329751
410004B18R	78	1.549098075	1.333313065	-0.21578501	0.1103702	0.241362851
Itga2b	9	63.24144933	65.38676328	2.145313959	0.11038477	0.241362851
333417C18R	4	1.96965415	1.310043668	-0.659610482	0.11035783	0.241362851
Mir8112	45	1.262994826	1.592394641	0.329399815	0.11042752	0.241420356
Marcksl1	67	2.738783388	1.950529549	-0.788253839	0.11053717	0.241624078
Stard3nl	23	0.976987115	1.224230479	0.247243364	0.11056336	0.241645339
BC029214	13	1.270824763	2.728873239	1.458048476	0.11058943	0.241666322
Txk	2	50.88303282	43.91951744	-6.963515383	0.1107006	0.241873229
Upk2	2	45.93686263	49.83822797	3.901365345	0.11079636	0.242046412
Ahi1	62	1.977816343	1.303747793	-0.67406855	0.11084363	0.242113636
Arfgap3	40	1.393014165	1.032137063	-0.360877102	0.11108846	0.242484634
Arl8b	49	1.135593363	1.00691086	-0.128682503	0.11104913	0.242484634
Piezo2	62	50.01138964	50.7184282	0.70703856	0.1110961	0.242484634
Tcta	11	1.175584626	0.669110701	-0.506473925	0.1110507	0.242484634
Aplf	11	1.196706636	0.717225747	-0.479480889	0.11106739	0.242484634
Tmem106a	10	2.214170967	1.806112866	-0.4080581	0.11114108	0.242546735
Gtpbp4	22	1.441385177	1.475222556	0.033837379	0.11121577	0.242644198

Casp8ap2	11	0.587106482	1.43608373	0.848977247	0.11121881	0.242644198
Mlh1	32	0.89688298	1.369727876	0.472844896	0.11126741	0.242714143
Cfap44	3	25.1984127	29.07997939	3.881566696	0.11137584	0.242914545
Astl	3	29.55712206	26.28326957	-3.273852495	0.11145232	0.243045224
)30440G07F	1	91.25	82.92682927	-8.323170732	0.11161271	0.243354834
Zfp53	15	2.482364514	1.765093767	-0.717270747	0.11163736	0.243354834
Ccdc15	21	2.005766206	1.655970051	-0.349796155	0.11165701	0.243354834
Fam208b	49	1.30338234	1.075832101	-0.227550239	0.11166063	0.243354834
Rnf115	50	1.199319516	1.001417904	-0.197901612	0.11182029	0.243657759
Herpud2	76	1.555878718	1.411808356	-0.144070362	0.11183283	0.243657759
Hlx	60	1.075830348	1.453964339	0.378133991	0.11190129	0.243770706
Cenpe	21	1.261784766	0.639358793	-0.622425973	0.11197897	0.243867524
Pnma3	10	76.94866161	73.95036163	-2.998299984	0.11196764	0.243867524
Loh12cr1	40	1.522594002	1.342492714	-0.180101289	0.11228516	0.244498055
Nup210	77	1.030182777	0.786282788	-0.243899989	0.11231082	0.244517656
Acta1	8	29.48931185	32.04992631	2.560614464	0.11249712	0.244886912
Tgif1	52	1.584078351	1.781114535	0.197036184	0.11257895	0.245015729
Rab34	96	1.631786829	1.453867011	-0.177919818	0.11258969	0.245015729
Tceb2	51	1.622204038	1.766017704	0.143813666	0.11263345	0.245074619
Mir762	43	1.771340316	1.458527729	-0.312812586	0.11265307	0.245080972
310015A10F	46	1.567490775	1.624825502	0.057334727	0.11268629	0.245116898
Paqr7	23	0.866757421	1.207051909	0.340294488	0.11272004	0.245117648
Egfr	36	2.01810945	1.715261002	-0.302848447	0.11270601	0.245117648
Gm16845	33	0.778872942	1.13884276	0.359969817	0.11286957	0.245370086
Ttc21a	18	2.432565338	2.408839487	-0.023725851	0.11286786	0.245370086
Esyt1	44	3.009674169	2.662216204	-0.347457964	0.11292459	0.245453327
Jagn1	27	1.439732107	0.899008246	-0.540723862	0.1129695	0.245502247
Wdr6	33	1.802770143	1.339611719	-0.463158424	0.11298056	0.245502247
Coro7	11	2.721402863	2.014487333	-0.70691553	0.11309605	0.245691526
Taf11	18	0.863262911	1.084997843	0.221734932	0.11310115	0.245691526
Nudt4	113	1.194664469	1.188390369	-0.0062741	0.11312332	0.245703325
Cstf3	34	0.998758222	1.079800629	0.081042407	0.11325474	0.245952349
Bcas2	24	0.695081989	0.613492711	-0.081589278	0.11331959	0.246056771
Mfn1	28	1.532569322	1.248114305	-0.284455017	0.11336231	0.2460767

Dgcr6	49	3.133717435	3.160323317	0.026605882	0.1133593	0.2460767
Gucy2c	1	33.80782918	28.03234501	-5.775484168	0.11343285	0.246120607
Brf1	82	1.217077739	0.991390514	-0.225687225	0.11343017	0.246120607
Nmd3	55	1.504662591	1.214532338	-0.290130253	0.11340085	0.246120607
Tmem150c	23	2.950006421	2.332832334	-0.617174087	0.11345477	0.24613178
Immt	31	1.162484389	1.312370255	0.149885866	0.11377478	0.246789532
Erlin2	35	4.349464939	3.296658862	-1.052806076	0.11381268	0.246835244
Cd164	37	1.08379553	0.788793748	-0.295001783	0.11382987	0.246836047
Tubgcp3	35	2.118257789	1.240365338	-0.877892451	0.11391181	0.246940739
Mir5131	107	1.029369831	1.113201752	0.083831921	0.11389718	0.246940739
Pum1	71	1.031313199	1.285703333	0.254390135	0.11396034	0.247009467
Cdc16	55	1.384216247	1.148953309	-0.235262938	0.11416683	0.247420487
Ttf2	13	1.645523219	1.29445355	-0.351069669	0.11427318	0.247614397
Hdlbp	108	1.326775103	1.193666218	-0.133108885	0.11437449	0.247748802
700021F05R	36	1.058212381	1.458968356	0.400755975	0.11438586	0.247748802
Nhlrc2	44	1.213168067	1.07177499	-0.141393077	0.11437445	0.247748802
Mesdc2	13	0.735384214	1.567292236	0.831908022	0.11445992	0.24787262
Gm11538	19	33.21450896	33.40307021	0.188561255	0.11452283	0.247972261
Ap3b1	13	0.59477761	0.893100189	0.298322579	0.11466435	0.24824206
Asb4	7	52.328956	56.07548429	3.746528289	0.11473909	0.248367234
Xylt2	95	1.182170929	1.505196615	0.323025686	0.1148616	0.248567647
Zfp84	8	2.000735178	0.901412668	-1.09932251	0.11486556	0.248567647
Tmem56	77	7.096092337	6.682511625	-0.413580712	0.11490357	0.248613242
Cnnm4	58	1.41205737	0.84277357	-0.5692838	0.11496151	0.248701922
Ptpn11	105	1.513118665	1.370511625	-0.142607039	0.11511553	0.248994445
Prrt3	11	22.06555018	20.49260575	-1.572944422	0.11513066	0.248994445
Sugt1	28	1.771206871	1.167312287	-0.603894583	0.11519201	0.249090426
Lrrc8a	66	1.235486915	0.908989524	-0.326497391	0.11529156	0.249268947
Cckbr	4	10.42041146	8.54337789	-1.877033569	0.11545561	0.249586865
Ano10	29	1.269278959	1.280396299	0.01111734	0.11556069	0.249771398
Scaf8	80	1.330006009	1.295395489	-0.03461052	0.11557502	0.249771398
Atp6v0a1	35	0.878159376	1.087721175	0.2095618	0.11565584	0.249909254
Cdh26	1	66.91729323	58.37563452	-8.541658715	0.11586647	0.250312561
Hexim2	48	2.190687337	1.82443671	-0.366250627	0.1158766	0.250312561

Cenpl	16	2.173563376	1.467208779	-0.706354597	0.11619043	0.250952089
Rffl	63	1.283224522	1.371457042	0.08823252	0.11620686	0.250952089
230307C23R	2	1.041666667	2.67878498	1.637118313	0.11626824	0.251010753
Zscan29	23	1.498953266	1.752849971	0.253896705	0.11625367	0.251010753
Zkscan6	48	0.937232128	1.0670123	0.129780172	0.11639736	0.251252562
Dlx1	29	1.210387775	1.855818952	0.645431178	0.11671178	0.25183326
Pcolce2	78	1.954352289	1.735004886	-0.219347403	0.11670951	0.25183326
Tet1	2	78.47884917	83.76623377	5.287384599	0.11671787	0.25183326
Ei24	57	1.633415981	1.787855305	0.154439324	0.11676552	0.251899039
Sde2	50	0.929712585	1.116379061	0.186666476	0.11692542	0.252206917
Stmn1	41	1.501422238	1.19199403	-0.309428207	0.11699927	0.252329103
Traf7	20	2.03645957	1.476009017	-0.560450553	0.11703858	0.252339709
Trmu	12	1.618502916	1.402002968	-0.216499948	0.11703803	0.252339709
Alg14	12	2.066414284	1.456781213	-0.609633071	0.11707708	0.252385633
Ttc7b	88	1.703170294	1.382698395	-0.320471899	0.11727123	0.252767051
Fam53a	72	1.34260608	1.090160602	-0.252445478	0.11729523	0.252781652
Rabep2	41	1.564926982	1.706920327	0.141993345	0.1173236	0.252805649
Pcdh9	30	43.71131927	41.81721205	-1.89410722	0.11737426	0.252869831
Esrp2	42	2.115372434	1.862355895	-0.25301654	0.11738785	0.252869831
Pkm	25	1.614887477	1.698940865	0.084053388	0.11742737	0.252917846
Ubc	40	1.061469274	1.636161887	0.574692613	0.11767655	0.253417334
Atrnl1	107	1.289271207	1.291391146	0.00211994	0.11776696	0.253571806
Dock4	48	1.092475968	1.209838217	0.117362249	0.11778284	0.253571806
Pip5k1a	16	2.688966046	1.636239065	-1.052726981	0.11781233	0.253598087
BC051226	55	1.5825208	1.240777543	-0.341743256	0.11787812	0.25370249
Rbpjl	2	21.57894737	28.65168539	7.072738025	0.11790995	0.25373377
Ssbp4	20	0.458572178	0.72682827	0.268256092	0.11797366	0.253796438
Rsbn1	20	1.548636505	1.032893557	-0.515742948	0.11796557	0.253796438
Gm16998	23	1.169903011	1.689641615	0.519738604	0.11818345	0.254135991
Zfp51	16	1.449157203	1.286191763	-0.16296544	0.11816124	0.254135991
Synm	67	1.557548539	1.408710625	-0.148837914	0.118174	0.254135991
AU015228	5	95.48376709	92.73117173	-2.752595365	0.11820982	0.254155442
AW209491	12	3.202641986	2.12650158	-1.076140407	0.11827504	0.254258409
Fam83d	46	1.230798281	0.950030265	-0.280768016	0.11842414	0.25446682

Ubtf	248	1.54486576	1.429663514	-0.115202245	0.11844135	0.25446682
Zfp740	86	1.353718152	1.249338102	-0.104380049	0.11843206	0.25446682
Cdk14	122	1.36954865	1.17368338	-0.19586527	0.11839284	0.25446682
Kansl1	111	1.174348006	1.282788686	0.108440679	0.11848048	0.25451362
Akap11	18	7.408130766	9.303302328	1.895171563	0.11869525	0.254937668
530066F11F	68	1.500864284	1.380219522	-0.120644762	0.11874854	0.254977468
330024D21F	7	89.59435364	85.64791507	-3.946438578	0.11874352	0.254977468
Ssrp1	14	1.615388442	1.62768637	0.012297928	0.11885512	0.255168997
Slc2a3	3	57.11213518	51.9379845	-5.174150681	0.11887262	0.255169219
Tax1bp3	32	1.545552282	1.100904109	-0.444648173	0.11896958	0.255340005
Atp8b1	89	24.1053462	24.7959422	0.690595997	0.11909981	0.255544764
Zfp872	5	63.74860836	60.80342171	-2.945186652	0.1190925	0.255544764
Nap1l3	21	23.34188939	25.14897284	1.807083447	0.11912171	0.255554382
Smap2	48	1.776362639	1.593828216	-0.182534423	0.11932618	0.255955607
Commd4	4	4.658735821	3.633893809	-1.024842012	0.11945663	0.256197986
Ptbp2	83	1.300414293	1.549493323	0.24907903	0.11953255	0.256308048
Cacna1g	83	1.967948109	2.297055163	0.329107054	0.11954288	0.256308048
Poc1a	19	1.005283752	1.465568584	0.460284832	0.11984853	0.256925843
Pard6a	15	1.164249569	1.542745451	0.378495882	0.11987353	0.256941895
Tmem43	10	1.121038133	1.72277733	0.601739197	0.11989657	0.256953746
Atp6v0e	25	1.586624293	2.070308671	0.483684379	0.12006897	0.25728563
Apmap	17	1.269137518	0.937051648	-0.332085871	0.12012123	0.257360038
Sertad3	23	1.547881812	1.869593527	0.321711714	0.12039719	0.257902899
Stat1	51	1.533402116	1.771314934	0.237912818	0.12040976	0.257902899
Hcfc1r1	8	1.449805068	0.247524752	-1.202280316	0.12053756	0.258138968
Wbscr27	17	1.885946556	1.340473316	-0.545473241	0.12059508	0.258186788
Lipt2	27	1.375371202	0.89406817	-0.481303032	0.12058582	0.258186788
Abca1	36	5.452204719	6.106051963	0.653847245	0.12067825	0.258327159
Crbn	50	1.978393575	1.691151268	-0.287242306	0.12080711	0.258478662
Raph1	68	1.005678439	0.789301261	-0.216377178	0.12087233	0.258478662
Apitd1	34	1.315434137	1.301473378	-0.013960759	0.12086721	0.258478662
Casz1	55	2.329542432	2.161009018	-0.168533415	0.12082328	0.258478662
430421F17F	2	68.58974359	61.64502165	-6.944721945	0.12084621	0.258478662
700015F17F	3	93.05420091	88.03872054	-5.015480371	0.1207803	0.258478662

Rybp	166	1.30777656	1.405019238	0.097242678	0.12079527	0.258478662
Rab27b	7	1.147136256	1.036481199	-0.110655056	0.12096876	0.258647183
Lbr	155	1.13146399	1.015569445	-0.115894545	0.12101314	0.258704363
Cdhr1	3	6.554299347	5.075757576	-1.478541771	0.12104716	0.258739411
Gosr2	28	1.432919168	1.653641588	0.22072242	0.1210873	0.258787512
Agbl5	29	1.574868568	1.209618713	-0.365249854	0.12112255	0.258825155
Ilkap	36	1.327861544	1.066511509	-0.261350035	0.12115931	0.258866003
Tbc1d15	46	1.423450521	1.399178732	-0.02427179	0.12131789	0.259167082
Zfp64	20	1.153212272	0.816124221	-0.33708805	0.12137616	0.259253833
Fam175b	49	1.259124331	1.456948916	0.197824586	0.12149572	0.259471421
Tmc8	13	22.8636851	24.73852635	1.87484125	0.12153815	0.259524278
Fam83g	62	4.446035033	5.016429602	0.570394568	0.12156213	0.259537709
Arhgef17	22	2.149874131	1.634056013	-0.515818117	0.12172689	0.259826709
Mblac2	54	1.818837273	1.539193089	-0.279644184	0.1217329	0.259826709
Edrf1	22	1.245523912	1.4847807	0.239256788	0.1218461	0.260030489
Dennd1b	79	0.987220597	1.185406822	0.198186225	0.12191828	0.260124131
Tmtc3	46	1.112049341	1.59533937	0.483290029	0.12194316	0.260124131
Lpp	27	2.270123318	1.979717798	-0.29040552	0.12193668	0.260124131
Msra	29	1.337315268	1.473075586	0.135760318	0.12202656	0.260226388
Man1a	37	1.14703857	1.363558847	0.216520277	0.1220236	0.260226388
Med1	40	1.600804969	1.945524737	0.344719768	0.12228467	0.260718721
Eme2	1	7.258064516	4.930966469	-2.327098047	0.12229296	0.260718721
'00003G18F	13	5.74838655	6.168330743	0.419944194	0.12240219	0.260913671
Mynn	25	2.476597858	2.023766323	-0.452831535	0.12244691	0.260971096
G0s2	4	44.21040501	49.71357819	5.503173183	0.12252566	0.261101003
Slc11a2	63	1.873194074	1.715752329	-0.157441745	0.12256075	0.261137865
Zdhhc8	64	2.57116289	2.156588309	-0.414574581	0.12258894	0.261147639
Mrpl57	52	1.036888911	1.043008428	0.006119517	0.12260093	0.261147639
Diablo	83	1.33987455	1.137042632	-0.202831918	0.12266875	0.261254172
Gm5086	2	69.74851897	65.91998344	-3.828535526	0.12276259	0.261416077
Ndufa4	54	1.027864127	0.730543006	-0.297321122	0.12309093	0.26207724
Fam168b	91	1.049273877	1.038620895	-0.010652983	0.12329649	0.262461616
Mc4r	3	16.36363636	14.96296296	-1.400673401	0.12330724	0.262461616
Zfp784	18	1.499949495	1.051259621	-0.448689874	0.12334124	0.262495926

Rpl22l1	57	1.143534926	0.977071209	-0.166463717	0.1234	0.262582887
Rras	34	1.592025745	1.357215435	-0.23481031	0.12350782	0.262774219
Mapk6	116	1.386944378	1.337170007	-0.049774372	0.12356063	0.262826663
F3	56	2.710185198	2.138950098	-0.5712351	0.12356829	0.262826663
Gfod1	91	1.403905386	1.038069905	-0.365835481	0.12360877	0.262836566
Zgrf1	13	0.734661608	1.017318178	0.28265657	0.12359589	0.262836566
Otop3	3	17.45938745	13.01084651	-4.448540936	0.12364092	0.26286682
Mms19	58	1.305545058	1.396176908	0.090631851	0.12382413	0.263218197
Hip1	17	2.167748913	1.99857991	-0.169169003	0.12386199	0.263260558
Cib1	36	1.487756132	1.491228658	0.003472526	0.12406583	0.263655618
Spata5l1	55	1.199259649	1.003918258	-0.195341391	0.12408843	0.263665463
Fosl1	21	0.798119131	1.094437808	0.296318677	0.12423265	0.26389547
Pign	4	1.19760479	0.388601036	-0.809003754	0.12421669	0.26389547
Chmp1b	19	1.506262952	0.947661321	-0.558601631	0.12431267	0.264027228
Ggcx	9	1.218800943	0.934095516	-0.284705427	0.12434427	0.264056121
310033P09F	9	0.615332205	1.284750157	0.669417952	0.12438204	0.264098117
Usp47	73	1.18412012	1.014255511	-0.169864609	0.12442134	0.264143345
Tbc1d31	25	2.12979396	1.658192842	-0.471601117	0.12450358	0.264250412
Ipp	39	1.536169242	1.858812425	0.322643183	0.12450779	0.264250412
Zfp950	8	3.841773495	3.403522576	-0.438250918	0.1245622	0.264327663
Abcb1a	14	2.651019896	2.126736395	-0.524283501	0.12467107	0.264520435
Ppt2	57	2.041962798	1.683647509	-0.358315289	0.12472824	0.264603477
Slc35a1	32	1.407577813	1.429482984	0.02190517	0.12475144	0.264614432
330033J07R	14	41.46560121	42.51304132	1.047440112	0.12480712	0.264694283
Klhl9	61	1.083112633	0.925334634	-0.157777999	0.12520863	0.265507437
Mir3100	4	84.60391865	81.8287962	-2.775122451	0.12523563	0.265526314
Hivep2	59	1.353358845	1.561378552	0.208019707	0.12531125	0.265648261
Nup107	28	0.966718309	1.421081222	0.454362913	0.12539163	0.265741886
Osr2	59	3.722346111	4.389110386	0.666764275	0.12538982	0.265741886
Gm7102	5	46.64941346	44.35417453	-2.295238928	0.12561576	0.26617843
Aph1b	5	0.11299435	0.570179113	0.457184763	0.12572534	0.266372167
Plekha8	67	1.297644322	1.740197233	0.442552911	0.12582574	0.266546392
Serp2	64	1.73705917	1.461445808	-0.275613362	0.12589142	0.266647041
Fbxo31	26	0.850664146	1.029793235	0.179129089	0.12605829	0.266947185

Hist1h4c	5	2.268617886	0.869565217	-1.399052669	0.12606951	0.266947185
Tgfbr1	63	1.601536521	1.356305806	-0.245230715	0.12618547	0.267154176
Isy1	37	1.486908081	1.068027669	-0.418880412	0.12648538	0.267750501
Fbxo5	56	1.554285564	1.256315475	-0.297970089	0.12652084	0.267786941
Cc2d1a	17	1.521186262	1.700750108	0.179563845	0.12660602	0.267928579
Fhl2	41	3.378935336	2.337348121	-1.041587215	0.12670593	0.268101339
Dnajb1	87	0.957640217	1.082216921	0.124576704	0.12684013	0.268342199
Lrrc15	25	11.08456144	11.34070868	0.256147246	0.12685633	0.268342199
Dcaf12	18	0.894134782	1.261151332	0.36701655	0.12688907	0.26837277
Rars2	15	2.094876014	1.325997825	-0.768878189	0.12694004	0.268441865
Vash2	77	1.500493814	1.339934135	-0.160559679	0.12711056	0.268698316
Ankhd1	74	1.209596773	1.094773397	-0.114823377	0.12708815	0.268698316
Dnajc30	56	1.096219698	0.776243216	-0.319976483	0.12711624	0.268698316
Slc6a7	10	21.84471819	21.43365441	-0.411063784	0.12720861	0.268854836
Nedd4	69	1.490281311	1.165496817	-0.324784494	0.12733523	0.269083696
Taf12	11	0	1.100501843	1.100501843	0.12736832	0.269114855
Mir7036b	45	1.323140068	1.237003074	-0.086136994	0.12745186	0.269136362
Samd15	22	1.20232311	1.074151047	-0.128172063	0.12744401	0.269136362
'00060C20F	6	41.99012667	38.43195898	-3.55816769	0.12744225	0.269136362
Xlr4a	1	69.02654867	60	-9.026548673	0.12740575	0.269136362
)30012K11F	30	2.512755886	2.104987575	-0.407768311	0.12753266	0.269268231
Slu7	22	1.505530225	1.672909129	0.167378905	0.12757691	0.269322906
Brix1	26	1.790120898	1.464176648	-0.32594425	0.12762865	0.269393388
Gps1	112	1.247189267	1.19070326	-0.056486006	0.12768817	0.269480248
Gpt2	38	2.491714334	2.082544922	-0.409169412	0.12778184	0.269639152
Brd9	20	1.973085578	1.436629714	-0.536455865	0.12798163	0.270021911
Mff	29	1.497654735	1.27859656	-0.219058174	0.12809927	0.270231259
Rnf25	15	1.623989001	1.614069623	-0.009919378	0.12817751	0.270320532
)30579G24F	5	1.471039336	0.482983422	-0.988055914	0.12817843	0.270320532
'00031P21F	19	1.944140851	1.695313326	-0.248827525	0.12825344	0.270367393
Gemin2	5	3.473512517	3.580172875	0.106660358	0.12824263	0.270367393
Tcerg1	66	1.162209145	0.991693401	-0.170515744	0.12825592	0.270367393
Abhd10	14	1.278425259	1.344237841	0.065812582	0.12829615	0.270413353
Rps20	23	1.290348164	0.790697687	-0.499650477	0.12837894	0.270548975

Rab8b	26	2.187818212	1.792243369	-0.395574843	0.12850113	0.27076761
Tmem88b	1	95.58823529	92.01877934	-3.569455951	0.12852525	0.270779542
Ndufb10	12	2.596054365	2.09149549	-0.504558875	0.12856976	0.270834424
Klhl18	30	1.379090181	1.663430322	0.284340141	0.12861724	0.27087546
Uaca	38	2.478450099	2.127485419	-0.35096468	0.12862616	0.27087546
-30548M08I	98	2.076038897	1.785319362	-0.290719535	0.12865041	0.270887667
Rad51	29	1.639236537	1.432255478	-0.206981059	0.12869179	0.270897054
Dach2	10	25.10118836	23.02110431	-2.080084058	0.1286753	0.270897054
Setd8	22	1.392520002	1.153367173	-0.239152829	0.12873013	0.270938895
Rogdi	39	0.923857871	1.438711922	0.514854051	0.12875518	0.270952757
Brd4	108	1.106990664	0.940582031	-0.166408632	0.12897071	0.27128901
Oaf	61	1.347844957	1.130650278	-0.21719468	0.12898892	0.27128901
Gm4971	3	87.97036615	85.01508296	-2.95528319	0.12893806	0.27128901
Mettl6	7	1.694907442	2.304957546	0.610050104	0.12898684	0.27128901
Uqcrb	3	0.687285223	0	-0.687285223	0.12900835	0.271290995
BC052040	6	2.199174322	0.946115288	-1.253059034	0.1290574	0.271355253
Efnb1	20	21.16758347	19.82429836	-1.343285111	0.1291437	0.271458901
Ndufv1	29	4.237376432	3.717821232	-0.519555199	0.12913077	0.271458901
Phf14	56	1.523647063	1.565347165	0.041700102	0.12919606	0.271530081
Ube2g2	70	2.41326834	1.753135651	-0.66013269	0.12925244	0.271609676
Uhrf1bp1	52	1.222430085	1.269405984	0.046975899	0.12934386	0.271762856
Mbnl2	6	0.975557461	2.465578423	1.490020961	0.12950893	0.272070736
Gpi1	42	11.57466682	11.8914517	0.316784872	0.12965314	0.272334696
Arhgap29	80	21.57459275	23.34849211	1.773899363	0.12979943	0.272602952
BC005624	34	2.135723113	1.701164123	-0.43455899	0.12991614	0.272809011
Tshz2	5	0.652212233	1.452980785	0.800768551	0.13008093	0.273076908
Tmprss2	6	29.24262481	26.80671604	-2.435908775	0.13007711	0.273076908
Kank3	12	3.512511367	2.547430986	-0.965080381	0.13014437	0.273171014
Zfp592	147	1.46531062	1.184945858	-0.280364762	0.13016836	0.273182274
Depdc1a	3	1.507069561	0.629127428	-0.877942133	0.13030181	0.273359792
430041D05F	63	15.69797194	16.93881882	1.240846874	0.13030616	0.273359792
Prpf19	45	9.272197029	8.619994892	-0.652202137	0.13030883	0.273359792
Klc1	66	1.278553287	0.883080249	-0.395473039	0.13035666	0.273421051
Igf1r	149	1.160225311	1.063482614	-0.096742697	0.130421	0.273516893

Aurka	14	1.397499377	1.192678588	-0.20482079	0.13044944	0.273537458
Hdac7	60	1.208398811	1.176316223	-0.032082588	0.13047498	0.273551914
Ppif	67	1.303732141	1.017806488	-0.285925654	0.13051814	0.273603305
Gtf3c3	24	2.036226934	2.671668203	0.635441269	0.13096342	0.274464578
230400D17F	9	0.364973191	1.219674046	0.854700855	0.13098511	0.274464578
Fv1	3	4.487179487	2.164502165	-2.322677323	0.13097212	0.274464578
Pan2	6	0	0.744390088	0.744390088	0.13104102	0.274542545
Gm11985	3	62.15745054	57.2623434	-4.895107136	0.13109605	0.274618621
Ufsp2	4	0.252525253	1.424590164	1.172064911	0.13112924	0.27464895
Cln5	78	1.292991362	1.375851135	0.082859773	0.13116626	0.274661153
Myo5a	25	1.223242813	0.951226003	-0.27201681	0.13120994	0.274661153
Grb2	95	1.089763061	1.177492213	0.087729152	0.13120921	0.274661153
Gdap1l1	20	21.49369802	21.82976256	0.336064534	0.13118283	0.274661153
Ccdc55	59	1.437492188	1.36778986	-0.069702327	0.13124305	0.274691273
Slc35c1	35	2.00112387	1.801646581	-0.199477289	0.13130763	0.274787239
Arfip1	53	1.605759501	1.649545291	0.04378579	0.13156471	0.275285984
Vps9d1	53	0.952794969	1.051112929	0.098317959	0.13159812	0.275316622
Znhit3	11	1.536381813	1.227656196	-0.308725617	0.1316243	0.275332136
Ptpmt1	45	1.547501474	1.182100217	-0.365401257	0.13171099	0.275441849
Aqp2	5	92.24796057	90.10843112	-2.139529447	0.13171429	0.275441849
Asah1	44	0.891062419	1.042422152	0.151359733	0.13186438	0.275716433
330175E14F	4	83.46347217	81.11034478	-2.353127387	0.13201621	0.275994561
Kbtbd3	40	1.765717771	1.684266272	-0.081451499	0.13205117	0.276028338
Id2	11	1.587229067	1.476505865	-0.110723202	0.13210317	0.276094636
Lrrtm1	39	58.76952007	57.42932109	-1.340198979	0.13212052	0.276094636
Dhdh	4	52.15517241	46.73076923	-5.424403183	0.13217868	0.27617685
Nup153	73	1.047634566	1.185620976	0.13798641	0.13229711	0.276353663
Npdc1	35	1.843198279	1.782530106	-0.060668173	0.13230097	0.276353663
Rad51d	10	2.434176344	1.584776276	-0.849400067	0.13241795	0.276553459
Zbtb44	70	0.944110941	1.422829277	0.478718336	0.13243432	0.276553459
Map6	64	8.18017962	8.585609995	0.405430374	0.13246045	0.276568666
Cdh1	46	6.091384308	5.800125321	-0.291258987	0.13258885	0.276797385
000079G21F	2	20.74468085	26.92090395	6.176223104	0.13261546	0.276813557
Sdr39u1	27	2.783955873	2.612893568	-0.171062305	0.13275544	0.27706632

Zic2	86	19.99336015	19.94957121	-0.043788942	0.13287256	0.277173917
Rasgrp4	5	7.197834323	8.107231214	0.909396891	0.13288255	0.277173917
'30056A06F	79	16.22275518	17.93620576	1.713450582	0.13286877	0.277173917
Sstr2	38	2.614486528	2.193233349	-0.421253179	0.13287465	0.277173917
Vsig2	3	86.90887344	80.09486349	-6.814009949	0.13300342	0.277386607
)10111I01R	25	1.716354051	1.474122738	-0.242231313	0.1331095	0.277568396
'30025D16F	26	1.939534235	1.402393369	-0.537140866	0.13312947	0.277570587
Pkp4	97	1.335213974	1.501378157	0.166164183	0.13347746	0.278256598
Irak4	26	1.684188882	1.957317605	0.273128723	0.13383193	0.278916319
Slc45a3	79	1.279260839	1.190784624	-0.088476215	0.13383038	0.278916319
Ddost	53	1.487257966	1.167180077	-0.32007789	0.13388543	0.278988183
Unc119	43	1.708230937	1.316564886	-0.391666051	0.13396332	0.279110859
Wiz	28	2.622476561	2.213597937	-0.408878624	0.13399054	0.279127938
Aldh16a1	9	0.962668141	1.259742367	0.297074227	0.1341622	0.279445864
Sugct	18	2.44136404	2.053476987	-0.387887053	0.13427843	0.279648258
Ids	10	20.51299747	18.06093776	-2.452059706	0.134304	0.279661834
Sec11a	15	0.352468996	1.180186924	0.827717928	0.13455654	0.280147931
Snai3	7	0.700411744	1.416691078	0.716279335	0.13472956	0.280423456
Mlec	79	1.701923945	1.435106766	-0.266817179	0.1347271	0.280423456
Abcb9	50	1.977357975	1.774117915	-0.20324006	0.13480353	0.280423456
Tmem19	42	1.402209363	1.176463829	-0.225745534	0.13480204	0.280423456
Fam114a2	43	1.721396012	1.280192887	-0.441203125	0.13477825	0.280423456
Mrps15	6	1.233830637	2.032274078	0.798443441	0.13476024	0.280423456
Tmem248	59	1.093949223	0.79663753	-0.297311693	0.13496951	0.280728919
Tax1bp1	59	1.116499665	0.947824213	-0.168675452	0.13508948	0.280891109
Cdc42ep5	6	3.605088347	2.602801313	-1.002287034	0.13506816	0.280891109
Mysm1	23	0.825213373	0.898216418	0.073003045	0.13510491	0.280891109
Mgea5	99	1.226192199	1.343813688	0.11762149	0.13519787	0.28104456
Axin2	79	1.683759669	1.61073264	-0.073027029	0.13537197	0.281366616
Bco1	5	84.7130031	85.7913841	1.078381007	0.13542869	0.281444635
Tollip	32	1.449431576	1.090656731	-0.358774845	0.13548403	0.281479923
Rcn1	65	40.32336742	38.06633834	-2.257029086	0.13548218	0.281479923
Pcca	18	1.702788094	1.049166512	-0.653621582	0.13552784	0.281491228
Cd2bp2	26	12.91862561	10.75931862	-2.159306993	0.13552715	0.281491228

Rpl37	62	1.790352464	1.551577692	-0.238774772	0.13567069	0.281748039
Gm17769	13	0.765750087	0.614511877	-0.15123821	0.13575159	0.281836276
300009A05F	35	1.499628815	1.255025817	-0.244602998	0.1357495	0.281836276
Setd4	19	0.757241697	1.38558336	0.628341663	0.1357792	0.281853735
Gdpgp1	12	1.102421873	0.834180144	-0.268241729	0.13581513	0.28188843
Mir7076	3	97.16779503	98.12609783	0.958302802	0.13584292	0.281906234
Tgoln1	7	2.086032926	1.690426346	-0.39560658	0.13592963	0.282046303
510015P09F	19	1.729307865	1.943974523	0.214666659	0.1362867	0.282747223
Zfp644	79	1.3535808	1.180093485	-0.173487315	0.13635595	0.282850891
Tex13a	2	87.62376238	80.93877551	-6.684986866	0.13645416	0.28297462
Mbd2	99	1.132037297	0.987484509	-0.144552788	0.13643882	0.28297462
Cntfr	22	8.266280631	8.695898027	0.429617396	0.13653582	0.283103943
Zfp280d	78	1.460241501	1.27559208	-0.184649421	0.13657526	0.283105722
Mpzl1	92	1.596707593	1.408264186	-0.188443407	0.13657074	0.283105722
Arap2	59	1.179458201	0.905750607	-0.273707594	0.13662764	0.283174283
Zfp677	4	12.42166447	10.39868209	-2.022982382	0.13671589	0.283317175
230056J06R	16	0.989212008	1.192749654	0.203537647	0.13683799	0.283530163
Hhatl	15	61.43166105	60.19262295	-1.239038098	0.13686904	0.283554462
Junb	56	1.35052023	1.420492571	0.069972341	0.13689172	0.283561421
Pes1	4	2.255941078	1.813384124	-0.442556954	0.13717801	0.284114345
Wdr75	45	1.214383022	1.551902872	0.33751985	0.1373703	0.284444071
Timm10	6	1.667566129	1.825744918	0.158178789	0.13737598	0.284444071
Taf8	16	1.433175568	1.4751552	0.041979632	0.13758644	0.284839657
Gspt1	41	0.763015951	1.238024062	0.475008111	0.13768009	0.284993313
Pnpla6	4	36.84372798	37.18071141	0.336983431	0.1377222	0.285040268
Gm29683	22	58.94978181	60.69633063	1.746548826	0.13777878	0.285092909
Cdk2	52	1.370477115	1.054912797	-0.315564318	0.13778649	0.285092909
Bcl10	51	1.585778014	1.250683031	-0.335094983	0.13802532	0.285546815
Fam227a	27	1.455588477	1.082119682	-0.373468796	0.13807491	0.285609145
Pex11a	37	1.787155751	1.535502528	-0.251653223	0.13829438	0.286022787
Flot2	61	14.35923716	13.92294777	-0.436289384	0.13834089	0.286078671
Pfkl	26	1.408203575	0.746915331	-0.661288243	0.13836546	0.28608915
Foxf1	105	6.112660244	5.786929475	-0.325730768	0.13861046	0.286555352
Rnft1	42	1.364847596	1.04762712	-0.317220475	0.13863174	0.28655898

Arrdc3	19	1.219291185	1.497342246	0.278051061	0.13867183	0.286601469
Cstf1	20	1.665983034	1.472703333	-0.193279702	0.13870696	0.286633703
Rab3gap2	21	1.163335837	0.937617261	-0.225718576	0.13879686	0.286738724
Txn1	12	0.814802352	1.205175373	0.390373021	0.13879521	0.286738724
Fbxw9	25	1.243716087	0.901716894	-0.341999193	0.13890586	0.286923513
Ppwd1	32	1.429579064	1.004311458	-0.425267606	0.13897563	0.287027225
Cdc14b	63	1.460833769	1.277663997	-0.183169772	0.13917531	0.287399167
B3galnt1	39	13.79034259	16.31948878	2.529146188	0.13935713	0.287714313
Ythdf3	60	1.187847307	0.903120634	-0.284726673	0.13936714	0.287714313
Ylpm1	49	1.376385077	1.274898361	-0.101486716	0.13945353	0.287816875
Tbc1d2	21	1.428823969	0.83099516	-0.597828809	0.13945604	0.287816875
Sacm1l	56	1.413120955	1.076474863	-0.336646092	0.13976544	0.288414856
Tmem53	17	0.780965535	1.294407074	0.513441538	0.13986186	0.288531172
Bcar3	25	1.272221621	1.505803549	0.233581928	0.13985859	0.288531172
Ube2r2	81	1.46895813	1.599884809	0.130926679	0.13988079	0.288531172
Pex1	44	1.214559832	1.057135372	-0.15742446	0.13993656	0.288605641
Fam173a	28	3.019331334	2.202324607	-0.817006727	0.14005151	0.288802119
Atp6v1e1	34	1.452512986	1.239964937	-0.212548049	0.14012854	0.288847804
Odf2l	42	1.006535914	1.174610428	0.168074513	0.14009864	0.288847804
Phf23	113	3.138466307	2.564395923	-0.574070384	0.14013272	0.288847804
Lcorl	95	1.156257194	0.959896274	-0.19636092	0.14027688	0.289104343
Canx	28	1.697161724	1.282588817	-0.414572907	0.14033248	0.28917831
Plcb3	30	4.30691701	2.985114858	-1.321802152	0.14038803	0.289252161
Prdx3	9	0.984226137	1.585626827	0.601400691	0.1404336	0.289305446
Rnf24	73	1.339293271	1.413625565	0.074332294	0.14047037	0.289340573
Icmt	91	1.201127305	1.036799063	-0.164328242	0.1406346	0.28963819
Gorab	10	1.129046455	0.625177913	-0.503868543	0.14071845	0.289770203
Mkl2	142	1.424357009	1.439719353	0.015362344	0.14083467	0.289968726
Tsta3	14	1.954974432	2.319424875	0.364450443	0.14087413	0.289968726
Rab43	84	1.536352321	1.408334442	-0.128017879	0.14085805	0.289968726
Fam20a	18	5.606328718	4.299444083	-1.306884635	0.14106382	0.290318447
Cpped1	25	2.32507475	1.639245731	-0.685829019	0.14110634	0.29036523
Ascc3	89	1.334729285	1.100200211	-0.234529074	0.14118755	0.290410157
Tbx6	9	19.10233079	17.42307872	-1.679252077	0.14118048	0.290410157

Usp32	60	1.826747287	1.317848059	-0.508899228	0.14118215	0.290410157
Krt12	5	96.43736695	95.31316876	-1.124198191	0.14135764	0.290719265
Anpep	11	92.11682038	90.14560234	-1.971218033	0.14140043	0.290766521
Itgav	71	1.396204864	1.206530654	-0.18967421	0.1414723	0.290873556
Papss1	45	1.281331119	1.137191385	-0.144139734	0.1415741	0.2910013
Zfp397	45	1.26506524	1.439428513	0.174363274	0.14155904	0.2910013
Brox	8	2.290877523	1.46239883	-0.828478693	0.14159901	0.291011742
Abcc9	5	33.1213614	28.72821098	-4.393150412	0.14174932	0.291274226
Thsd4	2	84.19644294	81.01679595	-3.179646995	0.14176643	0.291274226
Fndc5	28	8.421624313	8.120868584	-0.30075573	0.14190157	0.291511073
Gorasp2	96	1.385627041	1.154110788	-0.231516254	0.14192802	0.291524609
Tpbg	17	2.185376444	1.722113917	-0.463262527	0.1419682	0.291566318
Gas2l1	39	1.873139487	1.643165013	-0.229974474	0.142024	0.291640095
Ntpcr	17	4.073666793	4.157334001	0.083667209	0.14215297	0.291779737
Trp53rka	32	12.22619199	12.75435412	0.528162127	0.14217154	0.291779737
Slc44a1	153	0.988639993	0.954609987	-0.034030006	0.14211237	0.291779737
Safb	90	0.804784032	0.684770343	-0.120013689	0.14215939	0.291779737
Dna2	37	0.657086954	1.295543142	0.638456188	0.14225475	0.291909697
Cisd1	49	1.602994271	1.108172387	-0.494821884	0.14236068	0.292086216
Paip1	108	1.470583468	1.229409362	-0.241174106	0.1423964	0.292118664
Oaz2	12	2.699151023	2.252122626	-0.447028397	0.14258834	0.292471528
Wac	104	1.358440592	1.028523651	-0.329916941	0.1426309	0.292517935
Gm21283	3	8.159722222	6.126600423	-2.033121799	0.1426518	0.292519924
Angptl4	5	1.95029937	1.363290175	-0.587009195	0.1429097	0.293007807
Ccny	78	1.408899097	1.141107254	-0.267791843	0.1429713	0.293093163
Lypla1	51	1.280867624	0.913102922	-0.367764702	0.14303209	0.293135888
Gm15612	62	1.507945665	1.221825313	-0.286120353	0.1430318	0.293135888
Tmsb10	28	1.233122832	1.444738016	0.211615184	0.14320034	0.293439707
Drap1	46	1.527673196	1.376308161	-0.151365035	0.14339018	0.293746689
Cntn1	15	55.25644382	58.81118554	3.554741723	0.143386	0.293746689
Pear1	10	0.565664557	1.618484416	1.052819859	0.14351979	0.293971159
Cdkn1b	45	1.011226673	0.766403581	-0.244823092	0.14356194	0.294016453
Iba57	36	0.994497576	1.223032434	0.228534857	0.14395385	0.294777944
Zfp707	38	1.432757124	1.445278732	0.012521608	0.14398804	0.294806816

Cdk8	71	8.584600637	9.153648805	0.569048168	0.14403689	0.2948657
Rmi1	80	1.168603216	1.398336004	0.229732788	0.14408905	0.294871462
Gnai3	35	1.870033549	1.233597094	-0.636436455	0.14408791	0.294871462
Cenpf	21	1.343998503	1.28965634	-0.054342163	0.14409999	0.294871462
Gtf2ird2	54	1.205554141	1.354988836	0.149434694	0.14414836	0.294929316
Fn1	41	1.57286728	1.391923927	-0.180943353	0.14437549	0.295352852
Cmtm3	46	5.104375829	5.032038373	-0.072337457	0.14440367	0.295369321
Ralb	28	1.244188416	1.726638961	0.482450546	0.14443335	0.295388848
Hpdl	17	3.411256767	2.729717904	-0.681538864	0.14456687	0.295579526
Pex5	49	1.246576801	1.674700156	0.428123355	0.14455592	0.295579526
Rapgefl1	81	2.176015287	1.64299443	-0.533020856	0.14465362	0.295715681
Elp5	44	2.414109267	2.86804346	0.453934193	0.14469784	0.295764893
Atp5g3	49	1.320637895	1.399170352	0.078532456	0.14489992	0.296136684
Ostf1	66	1.452335589	1.106178135	-0.346157454	0.14501721	0.296252607
Nphs2	6	37.17657649	35.4179553	-1.758621193	0.14499072	0.296252607
Gm16675	33	1.686750957	1.532228642	-0.154522315	0.14500975	0.296252607
Tbc1d4	46	1.661793768	1.450049447	-0.211744321	0.14505663	0.296291895
Snrnp40	25	1.80693888	1.391383288	-0.415555592	0.1450911	0.296321056
Ten1	16	1.282879218	1.298956176	0.016076958	0.14518115	0.296463703
Brca2	32	0.996359202	1.101533866	0.105174664	0.14520222	0.296465474
Klhdc1	35	0.835629992	0.564836774	-0.270793218	0.14526768	0.29655787
Fam53b	83	1.547015705	1.993715794	0.446700089	0.14534993	0.296684497
Map3k1	134	1.200087754	1.189855976	-0.010231779	0.14540094	0.296747337
Rps24	19	0.790786825	1.170066897	0.379280072	0.14546739	0.29684168
Pgk1	32	14.93381948	16.28812033	1.354300849	0.14555306	0.296892631
Asun	26	0.893507531	1.517762516	0.624254985	0.14554327	0.296892631
Fbxl3	121	1.116757249	0.947524661	-0.169232588	0.14552699	0.296892631
St3gal2	12	3.985458149	3.490881221	-0.494576928	0.14570136	0.297153817
Spry2	61	1.753700888	1.27936281	-0.474338077	0.14618476	0.298044104
Mfsd9	6	3.388025023	4.708977768	1.320952745	0.14621825	0.298044104
Sh2d3c	3	23.84340045	22.68294294	-1.160457511	0.14621913	0.298044104
Proser1	33	1.268837768	1.061430285	-0.207407484	0.1461724	0.298044104
Micu1	17	1.478278632	1.376044254	-0.102234378	0.14629038	0.298147921
Dhx33	34	1.271797479	1.408729946	0.136932466	0.14646591	0.298464211

Tprn	43	1.214571875	0.874113038	-0.340458837	0.14648771	0.298467191
Gin1	10	3.239640959	1.931717043	-1.307923915	0.14651738	0.298486191
Desi2	14	1.91674369	1.395129283	-0.521614407	0.14660437	0.298621941
Fam179b	43	1.74653867	1.346590742	-0.399947927	0.14671107	0.298797806
Aldh2	4	4.779813634	3.019166518	-1.760647116	0.14676834	0.298872968
Wdr62	51	1.268389667	1.492958936	0.22456927	0.14686699	0.299032352
Syde1	23	15.72429856	14.38423726	-1.340061298	0.14701332	0.299288772
Rpp21	23	1.422504143	1.342712309	-0.079791834	0.14711421	0.299411086
Map2k2	61	1.207334542	1.471362611	0.264028069	0.14711196	0.299411086
Cep350	38	1.207826251	0.811956158	-0.395870093	0.14717277	0.299481464
Gmip	41	2.315975436	1.938361393	-0.377614044	0.14718961	0.299481464
Nop2	30	1.833944716	1.251601278	-0.582343438	0.14731823	0.299660057
Chtop	8	2.531414233	2.021585269	-0.509828965	0.147303	0.299660057
Zfp40	9	2.315164561	2.002041161	-0.313123399	0.14738337	0.29975102
Trim33	28	0.815433103	0.878142099	0.062708996	0.14748684	0.299878322
Gm14164	3	92.34234234	95.75012801	3.407785666	0.14747543	0.299878322
Ucn3	2	39.8092869	34.10204082	-5.707246083	0.14752913	0.299922762
BC037704	25	1.252304074	1.104110718	-0.148193357	0.14755506	0.299933923
Nbas	23	1.607641687	1.923195181	0.315553494	0.14763642	0.300018574
Tle1	48	0.972057918	0.756925339	-0.215132578	0.1476376	0.300018574
Chd6	71	1.901825651	1.626191245	-0.275634407	0.14767507	0.300053175
Ppp2r5b	12	2.886640334	2.744184182	-0.142456152	0.14776052	0.300143674
Hdac2	77	1.343190576	1.456341655	0.113151079	0.14775261	0.300143674
Cndp2	45	1.773075265	1.829644857	0.056569591	0.14782861	0.300198858
Jup	51	1.572855705	1.266917304	-0.305938401	0.14780989	0.300198858
B3gat3	31	1.509942769	2.005788222	0.495845453	0.14786128	0.300223659
Tmbim6	38	4.011261913	3.51491051	-0.496351403	0.14790562	0.300272139
Cyb5d2	20	1.24823379	0.873551823	-0.374681966	0.14793851	0.300297372
Matr3	21	0.319035604	0.872504487	0.553468883	0.14831379	0.300980834
Hbegf	11	1.086742449	1.249375829	0.162633379	0.14831623	0.300980834
Mrpl12	32	1.945772366	1.559476928	-0.386295438	0.14835663	0.301021176
Rapgef4	29	20.57152054	22.08526472	1.513744181	0.1484302	0.30112883
Sowahc	24	1.232827469	1.414127709	0.18130024	0.14850021	0.301187564
Usp33	23	1.084995643	1.306410058	0.221414415	0.14849378	0.301187564

Tnrc6b	48	1.091841081	1.019958499	-0.071882582	0.14855015	0.301247228
Fxyd4	1	22.3628692	17.1875	-5.175369198	0.14872574	0.301561633
Dag1	109	1.568133889	1.320410388	-0.247723501	0.14880181	0.301632513
Ubxn4	46	1.056382438	1.393781259	0.337398821	0.14879758	0.301632513
Hint3	17	0.586301339	1.230248695	0.643947356	0.14887268	0.301731149
Mir6936	51	1.193951848	0.933475526	-0.260476322	0.14889159	0.301731149
Rasa3	23	0.565007468	0.870326715	0.305319247	0.14899389	0.301896765
Gpr88	2	84.87651332	80.73502867	-4.141484649	0.1490723	0.301975202
Rps6kb1	54	1.785123089	1.482007142	-0.303115948	0.14907376	0.301975202
Ghitm	50	1.296465195	1.155285715	-0.14117948	0.14911125	0.302009458
Col5a1	40	1.406065249	0.946545805	-0.459519444	0.14922032	0.302188656
Bace2	23	4.850700898	6.14464868	1.293947782	0.149309	0.302326515
Zbtb7b	1	5.226480836	2.857142857	-2.369337979	0.14941774	0.302504953
Qrich2	1	95	97.64150943	2.641509434	0.14947418	0.302577483
Scaf1	79	1.789760732	1.93953596	0.149775228	0.14953246	0.30265369
Eif4ebp2	83	1.363640215	1.105431797	-0.258208417	0.14961774	0.302784541
Ccdc39	7	35.68885366	39.82758795	4.138734292	0.14967514	0.302858932
Lysmd4	14	1.393725481	2.117168669	0.723443188	0.14974838	0.302943846
Antxr2	35	1.487356991	1.483319961	-0.004037029	0.14975839	0.302943846
Mpg	44	3.048260499	2.937341841	-0.110918659	0.1498446	0.303076454
Wars2	10	2.136744089	1.486816831	-0.649927258	0.14987728	0.303100763
Idh3b	25	1.021674207	1.207061534	0.185387327	0.14997327	0.303253099
Wdr55	3	1.793721973	0.52246604	-1.271255933	0.15004275	0.303351784
Cops5	28	1.258878714	1.304923558	0.046044844	0.15008131	0.303387954
Zfp568	48	0.996520067	1.264071751	0.267551684	0.15013941	0.303421802
Hist1h1e	34	0.94906625	1.295554293	0.346488043	0.15012508	0.303421802
Rhobtb3	97	1.200753274	0.935883955	-0.26486932	0.15018474	0.303471607
Mtmr3	71	1.24777086	0.928893657	-0.318877203	0.15024804	0.303557732
Mrpl23	36	2.122560627	1.826257177	-0.29630345	0.15060337	0.304108134
Rpp14	4	1.108058608	0	-1.108058608	0.15056127	0.304108134
Ppp2cb	72	0.691159589	0.943221665	0.252062076	0.1506014	0.304108134
700023F06F	4	95.58807816	93.59335501	-1.994723151	0.15057757	0.304108134
Sorbs1	51	1.414739955	1.775830736	0.361090781	0.1506293	0.304118642
Pfkfb3	72	3.713571512	3.383228424	-0.330343089	0.15067252	0.304164056

Smn1	29	1.349664383	1.291830793	-0.05783359	0.15085879	0.304498191
Bin1	36	1.606878731	1.435713166	-0.171165565	0.15104839	0.304797645
Pcid2	120	0.985343959	1.176615776	0.191271817	0.15104869	0.304797645
Ctsf	29	20.23141922	18.60340921	-1.628010012	0.15117661	0.304971892
Spag4	21	1.437191815	1.111171881	-0.326019934	0.15117081	0.304971892
Arvcf	59	2.481480272	2.47797761	-0.003502663	0.15125099	0.305077483
Gm364	4	90.88579463	87.28271778	-3.603076855	0.15127053	0.305077483
Med20	8	0.859834712	0.182334833	-0.677499879	0.15134341	0.305119393
Secisbp2l	34	1.377945264	1.206044118	-0.171901146	0.1513521	0.305119393
Mtch1	24	1.829598413	1.368352765	-0.461245648	0.15135369	0.305119393
Msh6	45	1.532673738	1.280059789	-0.252613949	0.15177052	0.305900373
Txnrd1	68	1.412624271	1.416667944	0.004043673	0.15178971	0.305900373
Cog1	27	1.6477068	1.127767311	-0.519939489	0.15186617	0.305900373
310428I15R	22	1.36339534	1.643408309	0.280012969	0.15184413	0.305900373
Dmxl1	65	0.81668744	0.841710315	0.025022875	0.15181308	0.305900373
Mir190a	3	77.60502139	79.68142968	2.076408291	0.15184755	0.305900373
Rad23b	120	1.460226803	1.187697495	-0.272529307	0.15195959	0.30604653
Smim14	84	1.059601829	1.225963025	0.166361196	0.15249984	0.307092444
Racgap1	54	1.17494988	1.290308638	0.115358759	0.15253955	0.307115793
Fkbp1a	54	1.696973478	1.595877659	-0.101095819	0.15255329	0.307115793
Kcne1l	14	58.43224823	58.60322468	0.170976443	0.15264407	0.307256388
Gpr156	33	37.01397123	37.45579993	0.441828703	0.15270139	0.307329602
Ubr2	52	1.443010216	1.199080629	-0.243929587	0.15272861	0.307342235
Copg2	80	1.63754957	1.690495121	0.052945551	0.1528032	0.30740803
530005A16R	22	1.726848271	1.206622082	-0.520226189	0.15278359	0.30740803
Pgm5	38	16.86277368	15.64167383	-1.221099843	0.1528257	0.307411151
Ift22	4	3.006754807	1.97790715	-1.028847657	0.15290826	0.30753507
Plod2	80	1.751519035	1.617952736	-0.1335663	0.1529908	0.30765891
Exosc3	20	1.450746015	1.811569488	0.360823473	0.1530694	0.307774786
Tinf2	37	1.161675796	0.752463341	-0.409212456	0.1531378	0.307870135
Ndel1	36	1.458346501	0.804586848	-0.653759653	0.15316687	0.307886382
Bcam	2	23.25206398	19.37069422	-3.88136976	0.15337254	0.308257579
Sart1	65	1.068179021	0.996387898	-0.071791123	0.15354494	0.308561833
Med30	40	0.680201483	0.862686796	0.182485313	0.15400659	0.309447169

Prim1	14	2.014630469	1.460152052	-0.554478417	0.15413011	0.309619215
Ccdc177	10	12.42427422	10.11580642	-2.308467796	0.15413441	0.309619215
Dennd6b	45	1.201574738	1.14978807	-0.051786668	0.15416265	0.30963356
Armc9	33	1.843415207	2.39284246	0.549427253	0.15434565	0.309958682
Ric8	22	1.543009708	1.306476385	-0.236533323	0.15442716	0.310079935
Gabpa	65	1.157369732	1.461049876	0.303680144	0.15455129	0.310286725
Epha7	25	1.063839887	0.796955107	-0.26688478	0.15463262	0.310407528
Gli1	5	3.046052388	4.11213676	1.066084372	0.1547435	0.310553896
Plod1	32	1.347468703	1.04475201	-0.302716694	0.15474786	0.310553896
Rnf146	34	1.074837657	1.095474686	0.02063703	0.15493992	0.310896806
Hnrnpu	74	1.042938641	1.037371675	-0.005566965	0.15497282	0.310920315
Arl13b	20	1.253458078	1.921735519	0.668277441	0.15512506	0.311140663
Nrxn3	17	43.04222928	44.80918876	1.766959482	0.15512265	0.311140663
Ccnd2	6	10.73268921	15.09856631	4.365877097	0.15515139	0.311150951
2-Mar	33	1.621496351	1.053286681	-0.56820967	0.15519791	0.311201703
Dpm3	8	7.022319612	7.48199001	0.459670398	0.15536642	0.311497041
Neurl2	8	2.390822793	3.454702973	1.06388018	0.15552612	0.311774621
Ccar2	12	2.365048287	2.346862818	-0.018185469	0.15558727	0.311854608
Rpusd2	31	1.643476653	1.197394527	-0.446082126	0.15578802	0.312214337
Ap1g1	74	1.560550559	1.252315014	-0.308235546	0.15589543	0.312364819
Zfp467	42	6.918067774	6.716606316	-0.201461458	0.15590568	0.312364819
Rprd1a	19	1.705445748	1.141653824	-0.563791924	0.15604486	0.312600985
Prkaa1	125	1.151078119	1.189664044	0.038585925	0.15613376	0.312722293
Mrgpre	4	4.85098004	3.967243529	-0.883736511	0.15614803	0.312722293
Toporsos	12	1.005532482	1.688131137	0.682598656	0.1562229	0.312829529
Slc38a9	5	2.26665045	3.950850967	1.684200517	0.15625797	0.312857062
Lrrc61	5	1.058201058	1.187160563	0.128959504	0.15631918	0.312922562
Icam4	5	4.128161224	3.499438908	-0.628722316	0.15633333	0.312922562
Fbxl18	21	1.419473784	1.704609018	0.285135234	0.15640986	0.312994209
Mir5617	2	57.19211344	62.99435028	5.80223684	0.15641179	0.312994209
)33439C10F	23	2.320207017	1.373495025	-0.946711991	0.15645486	0.313037723
Rnf138	92	1.161853456	1.380099419	0.218245963	0.15665264	0.313390705
Gid4	27	1.076405493	1.096152937	0.019747445	0.15670518	0.313453086
Gstm1	2	49.16666667	44.00224215	-5.164424514	0.15690758	0.313815157

Rpl12	35	1.13561408	1.216952269	0.081338189	0.15704725	0.314041913
Rnd2	55	19.50567346	18.85439412	-0.651279341	0.15708516	0.314041913
Ttc30b	14	1.618401106	1.002143898	-0.616257208	0.15708323	0.314041913
Dbnl	40	1.656518335	1.295554789	-0.360963546	0.15714219	0.314113129
Ap1ar	130	1.18647612	1.301444551	0.114968432	0.15717319	0.314132304
Rbm47	36	2.172197006	1.87840177	-0.293795236	0.15727428	0.314291542
[illegible]10012O05[illegible]	11	0.847982556	0.499212072	-0.348770484	0.15729621	0.31429256
Nuf2	29	1.74615321	1.302335401	-0.443817809	0.15758308	0.314822898
Rwdd3	9	0.433690013	1.666912459	1.233222446	0.15765606	0.314925808
Lrrc59	54	2.069515712	1.477554844	-0.591960868	0.15783487	0.3151543
Taf6	3	2.089390845	0.833052232	-1.256338613	0.15782888	0.3151543
Map2k5	53	1.706370418	1.426007501	-0.280362917	0.15782259	0.3151543
Tacstd2	19	39.2084909	39.97366097	0.765170073	0.15797186	0.315384912
Gm2011	3	1.078578579	2.936536828	1.857958249	0.15804504	0.315448814
Cpeb2	132	1.327853713	1.307565443	-0.02028827	0.15804686	0.315448814
Atp7a	10	19.89731262	17.87428288	-2.023029738	0.15811422	0.315540331
Parvb	29	2.482408206	2.289239856	-0.19316835	0.15814448	0.315557812
Pias4	28	1.707217361	0.872423128	-0.834794233	0.15823858	0.315659713
Foxo3	86	1.435092127	1.084047126	-0.351045001	0.15822617	0.315659713
Phactr1	15	45.84795946	48.60418632	2.756226859	0.15835695	0.315852904
Tead1	62	2.509436589	2.003704848	-0.505731741	0.15865366	0.316401705
Cir1	19	1.81701506	1.013789441	-0.803225619	0.15874402	0.316532896
Rtn4	80	1.726952675	1.295754716	-0.431197959	0.15876258	0.316532896
Acads	37	2.042261149	2.180673518	0.138412369	0.15888523	0.31673439
Plce1	8	98.0597761	97.39287102	-0.666905081	0.1589847	0.316803578
Il23a	2	42.29901961	47.4137931	5.114773496	0.15895295	0.316803578
Irgm2	5	3.850606909	3.676675012	-0.173931898	0.15897863	0.316803578
Rsph4a	3	37.27263436	41.89479334	4.622158981	0.15901751	0.316825933
Ccbe1	25	1.618865961	1.130951735	-0.487914226	0.1591685	0.317083698
Cmss1	11	1.081355958	0.900871602	-0.180484355	0.15932379	0.317349975
Sox15	4	39.85483401	41.11360969	1.25877568	0.15940193	0.31746253
Galc	45	1.552942705	1.545472762	-0.007469943	0.15942401	0.317463421
Ppapdc1a	51	43.07396109	43.86578114	0.791820051	0.15945909	0.317490195
Ncald	40	1.861100619	1.54824922	-0.312851399	0.15960747	0.31774251

Grk5	45	2.035271547	1.508476797	-0.52679475	0.15967959	0.317842964
Gm16701	20	1.925903498	2.7685041	0.842600602	0.15995206	0.318329333
Rnpep	42	0.824327365	0.926417602	0.102090238	0.15996732	0.318329333
Mtmr14	35	1.268033238	0.899574644	-0.368458594	0.15999282	0.318336898
Lpin1	42	5.989521736	5.481697228	-0.507824508	0.16020557	0.318716993
Foxn2	70	1.046402805	1.210547099	0.164144294	0.16025586	0.318773823
Ppp6r1	48	0.912284072	0.905076667	-0.007207405	0.16031353	0.318845322
Sbk1	91	1.214830729	0.948807185	-0.266023544	0.16033588	0.318846546
Apbb1ip	13	18.32375541	15.57934228	-2.744413121	0.1605404	0.31921
Tmem62	39	0.917242833	0.946438013	0.029195181	0.16058615	0.31925772
Snora78	21	1.12694918	0.743448722	-0.383500458	0.16068845	0.319417821
Mapk1ip1	35	0.859754741	1.047558428	0.187803687	0.16081791	0.319631861
Treml1	4	81.99202174	77.72308004	-4.268941699	0.16087233	0.319696716
Uap1l1	39	3.157798856	2.629186308	-0.528612548	0.1609571	0.319821864
Pex11b	36	2.310976291	1.145287712	-1.165688579	0.16101441	0.319849126
Itgb3	47	1.924667062	1.498037103	-0.426629958	0.16100818	0.319849126
Cdc42bpa	55	1.439770615	1.49746202	0.057691405	0.16107395	0.319924094
Snora36b	2	90.08091246	83.11543529	-6.965477169	0.16111749	0.319926711
Btrc	17	1.004713469	1.472408716	0.467695246	0.16114184	0.319926711
Zcchc4	20	1.559951565	1.011995743	-0.547955821	0.16112297	0.319926711
Ddx56	14	1.189876802	0.844025263	-0.345851539	0.16116248	0.319926711
St6galnac3	7	47.31923811	49.50432027	2.18508216	0.16118718	0.319932468
Dhrs7b	21	2.555518415	1.520305069	-1.035213346	0.16124997	0.319970537
)30579G18F	6	68.97412355	67.59040115	-1.383722404	0.16124276	0.319970537
Ggn	7	5.304113028	2.995787442	-2.308325586	0.16128561	0.319997987
Arid4b	51	1.65502382	1.203568446	-0.451455374	0.16137827	0.320138539
Arl14epl	3	92.67939815	89.77260528	-2.906792868	0.16142633	0.320190589
Wdr78	31	0.872361367	0.973876102	0.101514734	0.16148389	0.320194369
Aip	15	1.04770261	1.149223035	0.101520425	0.16147774	0.320194369
Scrt2	53	4.470231053	6.850008417	2.379777365	0.1614937	0.320194369
Cdc34	101	1.106450005	1.335517747	0.229067742	0.16159254	0.320347066
Ccnyl1	100	1.529298212	1.288173701	-0.241124511	0.16174032	0.320596721
Pdcd5	47	1.02504202	1.167623061	0.142581041	0.16188422	0.320838598
Brip1os	39	1.055211902	0.932401288	-0.122810614	0.16220619	0.321389908

Dock5	74	1.346660879	1.231598269	-0.11506261	0.16219217	0.321389908
Pgam5	65	1.052703972	1.182331571	0.129627599	0.16238729	0.321531651
Adprhl2	27	23.66738313	24.75663731	1.089254188	0.16232788	0.321531651
Jak2	105	0.927950181	0.900750126	-0.027200055	0.16236559	0.321531651
Mir219b	28	96.21971826	96.60942298	0.389704726	0.16234511	0.321531651
Ssr1	105	1.247487044	1.0754292	-0.172057844	0.16230549	0.321531651
Mrps33	40	0.99074134	0.957891485	-0.032849855	0.16265983	0.321984394
Acd	5	1.738437002	0.327890882	-1.410546119	0.16264978	0.321984394
Fbxl5	87	0.955605927	0.92670931	-0.028896617	0.16270979	0.32203986
Eif4enif1	144	1.331613615	1.212189965	-0.11942365	0.16298384	0.322538753
410131K14R	38	0.878278995	1.049521608	0.171242613	0.16303017	0.322586935
Abcd4	16	2.05076136	1.336097363	-0.714663997	0.16306754	0.322617371
Map3k7	59	0.706407494	0.897166216	0.190758722	0.163115	0.322624284
Rbl2	12	0.584447229	1.17943884	0.59499161	0.16309413	0.322624284
Abhd2	28	1.870867406	1.577189046	-0.293678361	0.16316043	0.322670642
Esyt2	51	0.953533638	1.192986727	0.239453089	0.16319453	0.322694597
Slc46a1	2	0.485436893	1.632734003	1.14729711	0.16343151	0.323119646
Tagln2	9	1.050370835	1.806746915	0.75637608	0.1634775	0.323167017
Hspbp1	5	2.648426404	3.130331963	0.48190556	0.16356035	0.323287257
Tmem97	20	1.667998856	1.564453194	-0.103545662	0.16359396	0.323310141
Srpr	58	1.422118449	1.085693548	-0.336424901	0.16371249	0.323413713
Kcnmb4os1	6	82.23097812	78.91076992	-3.320208206	0.16368248	0.323413713
Itpr2	40	3.233555049	3.083876924	-0.149678125	0.16369479	0.323413713
Fam150a	27	19.37727542	20.0016937	0.624418283	0.16377907	0.323501686
Cnnm2	52	1.545913404	1.599820862	0.053907458	0.16388558	0.323668505
Cdk10	29	1.479544092	1.177238733	-0.302305359	0.16403732	0.323924583
700106J16R	5	22.23690572	26.28982129	4.052915569	0.16405955	0.323924897
Olfr550	1	91.33333333	95	3.666666667	0.16408503	0.323931624
Zcchc17	5	1.760710081	2.250555517	0.489845437	0.16427104	0.324255218
Morn2	28	1.309578851	1.727830503	0.418251652	0.16439046	0.324447294
Srms	6	5.862704215	8.584900298	2.722196084	0.16448363	0.324587509
Sdf4	58	1.121368068	1.250574617	0.129206549	0.16470912	0.324945091
Slc35e2	35	1.279987793	0.975704969	-0.304282824	0.16470608	0.324945091
Kctd10	22	1.306243462	1.284386386	-0.021857076	0.1648602	0.325199432

Gse1	261	1.331165337	1.148707752	-0.182457585	0.16493037	0.32529412
Hspa12b	7	10.23434283	8.856538342	-1.377804492	0.16522464	0.325830721
Cyb561	83	1.667759752	1.192325298	-0.475434455	0.1652861	0.325908117
Ostc	29	1.732775321	1.622034811	-0.11074051	0.1654055	0.326099737
Creb1	97	1.137114459	0.998161229	-0.13895323	0.16563379	0.326505941
)30006K02F	5	11.23608459	14.41546855	3.179383963	0.16576685	0.326687779
Msh2	42	1.299562972	1.515596644	0.216033672	0.16577056	0.326687779
Cant1	63	3.050642436	2.804840105	-0.24580233	0.1658715	0.326842814
'00019N19F	1	22.58064516	17.68488746	-4.895757701	0.16590694	0.326868752
Fxn	14	0.737046913	1.233773421	0.496726508	0.16599206	0.326992544
Igsf11	49	1.345771722	1.566733252	0.22096153	0.16604527	0.327009564
Anxa1	1	5.737704918	2.34375	-3.393954918	0.16602972	0.327009564
)30032P07F	24	2.119768733	2.28850116	0.168732428	0.16618576	0.327115739
Ddb2	9	1.845676436	1.638147068	-0.207529367	0.16618556	0.327115739
Zbed3	13	0.849733357	0.365597495	-0.484135862	0.16615068	0.327115739
Krtap3-1	2	91.59865696	88.91333408	-2.685322886	0.16618835	0.327115739
Fbxw11	94	1.23348816	1.389602541	0.156114381	0.16624667	0.327150484
Sema4d	46	1.05183635	0.759909421	-0.291926929	0.16625059	0.327150484
Ephx3	9	34.52972108	36.95049918	2.420778104	0.16639049	0.327381877
Gmds	95	1.66537909	1.452616331	-0.212762759	0.16646506	0.327484686
Elp2	11	2.236461613	1.148359793	-1.088101821	0.16652413	0.327556985
Txndc16	12	1.495217973	1.80290485	0.307686878	0.16668476	0.327829008
Il11	10	20.23798651	19.93239675	-0.305589767	0.166725	0.327864207
Ndufb7	30	1.548122565	1.548679272	0.000556708	0.1669699	0.328275719
Wipf2	26	5.052136585	4.690743965	-0.361392619	0.16697901	0.328275719
Wdr45	8	17.18288479	16.33543663	-0.847448162	0.16703611	0.32834398
Lrrc16a	59	1.290422151	1.143889061	-0.14653309	0.16713509	0.328494559
Odf2	102	2.161136125	2.360325891	0.199189766	0.16717249	0.328524057
Pank2	32	0.811433184	0.631382162	-0.180051022	0.16738409	0.328895845
Cd99l2	4	24.44255801	27.02381192	2.581253909	0.1675578	0.329193083
Zfp36	18	2.071758754	1.459983061	-0.611775693	0.16770522	0.32943861
Cluh	68	1.582818327	1.635772248	0.05295392	0.16775306	0.329466453
Ly6d	1	98.30508475	93.44262295	-4.862461795	0.1677643	0.329466453
'00113A16F	47	1.239429647	1.519648125	0.280218477	0.16783363	0.329514409

Kirrel2	17	10.45952516	10.99591102	0.536385869	0.16782044	0.329514409
Snf8	27	1.533139849	1.900238948	0.367099099	0.1680253	0.329846584
Fndc3c1	17	72.93010033	71.19557801	-1.73452232	0.16807383	0.329889864
Nkx6-3	5	93.92139888	93.09806648	-0.823332407	0.16809231	0.329889864
Ap4s1	23	1.1312175	1.469217242	0.337999742	0.16820103	0.330011832
Nid1	4	27.62428048	30.49498055	2.870700068	0.16819875	0.330011832
Htr1f	39	61.81391487	59.03851446	-2.775400405	0.16822192	0.330011832
Wdr26	137	1.569181632	1.349176161	-0.220005471	0.16827149	0.33006495
Haus2	1	10.19736842	7.235142119	-2.962226302	0.1683244	0.330110159
Zfp94	11	1.176918939	0.630514735	-0.546404204	0.16833953	0.330110159
Zswim1	14	0.901958686	0.810622035	-0.091336651	0.168413	0.330210087
Stamos	20	1.154829408	0.70098702	-0.453842388	0.16859196	0.330484417
P2rx2	13	50.75719527	53.80954559	3.052350326	0.16859795	0.330484417
Phlda2	29	2.119922293	1.935427077	-0.184495217	0.16906183	0.331349447
Sugp1	9	3.365145547	1.621924389	-1.743221158	0.16913118	0.331441103
Siah1b	29	22.86345962	21.96503277	-0.898426845	0.16934751	0.331820713
Mest	6	80.24954191	82.01953002	1.769988104	0.16943212	0.33194219
Mir7648	19	0.571820948	1.166297195	0.594476247	0.16946337	0.331959077
Lrrc36	8	30.1140565	34.84450417	4.730447667	0.16951351	0.332012971
Bfar	9	1.647885328	1.786769637	0.138884308	0.16988595	0.332558411
Nkx2-2	16	50.66591535	51.79540463	1.129489279	0.16986318	0.332558411
Usp48	6	0.64516129	1.121130813	0.475969523	0.16992797	0.332558411
030617O03F	3	4.032542405	3.03681787	-0.995724535	0.16982698	0.332558411
Fgfr1op2	32	1.210272124	0.997596258	-0.212675866	0.16986015	0.332558411
Ubxn1	22	1.404343664	1.544763016	0.140419352	0.1699132	0.332558411
Mir6418	1	11.40939597	7.065217391	-4.344178582	0.1699711	0.332583878
7-Mar	120	1.205076796	1.013370711	-0.191706086	0.17003164	0.332583878
Upp2	144	1.427031275	1.206740919	-0.220290355	0.17001444	0.332583878
Rab12	87	1.362099941	1.179747848	-0.182352093	0.17001192	0.332583878
Cebpzos	29	1.255820662	1.581541985	0.325721323	0.17012457	0.3327213
Gfpt1	31	1.238337245	1.585340947	0.347003703	0.17027303	0.33296728
Mir7688	3	1.875338753	3.50877193	1.633433176	0.17036109	0.33309508
Mir1291	3	100	98.24561404	-1.754385965	0.17050409	0.333324185
Snx32	27	1.762879831	1.511466207	-0.251413624	0.17052369	0.333324185

Kdm8	26	0.852254291	0.864941103	0.012686811	0.17077089	0.333727665
Ube2d3	46	1.543103239	1.284424222	-0.258679017	0.17077559	0.333727665
Ap1s1	28	2.143713648	1.645458162	-0.498255486	0.17089619	0.333829969
Hoxc12	25	41.63586249	46.11439177	4.478529275	0.17085105	0.333829969
Gdpd5	47	1.340787971	1.428649095	0.087861124	0.17088637	0.333829969
Shcbp1	11	2.100776115	1.699366518	-0.401409597	0.17128039	0.334535926
Pdlim7	18	1.741727248	1.265978382	-0.475748866	0.17135683	0.334640691
330403K07F	2	14.1156143	14.33691756	0.221303264	0.17161039	0.335091267
Yipf3	37	1.412849733	1.01043847	-0.402411263	0.17167582	0.335174428
Bbs1	18	26.42711324	26.84375475	0.416641515	0.17182506	0.335395613
Otub1	34	1.381971268	0.974872258	-0.40709901	0.17183482	0.335395613
Ppie	17	2.840927576	2.231462779	-0.609464797	0.17186916	0.33541801
Dgkz	79	2.445486057	2.271100469	-0.174385588	0.17198025	0.335548741
700001G11F	52	1.432341469	1.510045876	0.077704407	0.17198188	0.335548741
Kcnb1	71	41.42942174	41.61110854	0.181686799	0.17209031	0.335715668
Klrg1	1	93.67088608	91.07611549	-2.59477059	0.17221129	0.335907014
310067B10F	15	2.194289452	1.995384123	-0.198905329	0.17229778	0.336031049
Usf2	101	1.435112183	1.160482926	-0.274629257	0.17246785	0.336194056
Brap	56	1.378208149	1.148943121	-0.229265028	0.17243652	0.336194056
510040B10F	15	0.688331929	0.757678209	0.06934628	0.17249152	0.336194056
Ltn1	39	1.187971954	1.381168893	0.193196939	0.17249591	0.336194056
310009A05F	4	0.981395349	3.064156697	2.082761348	0.17240797	0.336194056
Morc3	81	1.207494679	1.099421345	-0.108073334	0.17258601	0.336280321
Klhl12	18	2.095402833	1.853663329	-0.241739504	0.17257333	0.336280321
Slc25a47	2	94.14893617	91.61184211	-2.537094065	0.17275957	0.336573815
Dync1li1	84	1.580695419	1.34034859	-0.240346829	0.17281296	0.336633134
B9d1	14	10.36238766	9.54229781	-0.820089855	0.17284534	0.336651514
Cog6	34	1.275507671	1.019125328	-0.256382343	0.1729299	0.336726831
Rassf10	71	11.95554735	13.38504948	1.429502137	0.17291036	0.336726831
Fkbp3	30	1.373842292	0.985375615	-0.388466677	0.17304751	0.336866423
Rspo2	57	28.6303289	28.85082021	0.220491303	0.17302547	0.336866423
Pald1	60	2.267537202	1.973037266	-0.294499936	0.17328659	0.337287099
Doc2b	92	23.92130826	24.6473046	0.725996334	0.17333114	0.337329066
Dkc1	3	24.50980392	28.17113696	3.661333043	0.17336886	0.337357724

Cbll1	45	1.668346401	1.386640247	-0.281706154	0.17350442	0.337487248
Dner	18	33.76362759	36.02960623	2.265978636	0.17348594	0.337487248
Edf1	62	1.409508347	1.041585786	-0.367922561	0.17348778	0.337487248
Zfr2	31	1.416811696	1.145148137	-0.271663559	0.173538	0.337507837
Slc48a1	39	1.749131804	1.404536606	-0.344595198	0.17356455	0.337514731
210016F16R	8	2.924533521	2.343068141	-0.58146538	0.17362692	0.337546564
Derl2	33	1.180514731	0.812688893	-0.367825837	0.17361089	0.337546564
Trappc5	18	2.433639143	1.546958993	-0.886680149	0.17366086	0.337567809
Hspb6	8	4.504383104	3.475010865	-1.029372239	0.17400872	0.338199192
Card10	94	2.384445329	2.092791364	-0.291653965	0.1741869	0.338500669
Il12a	11	61.31753107	59.60162401	-1.715907064	0.17421413	0.338508759
Klf8	10	51.94207393	54.98196884	3.039894911	0.17433462	0.338698031
Nuak1	118	1.715188375	1.56023144	-0.154956935	0.17464425	0.339254664
Edaradd	24	4.542020773	5.874016041	1.331995268	0.17469862	0.339315361
Vps35	38	1.185904727	1.321045456	0.135140729	0.17478602	0.339440182
Snrpa	52	1.262529616	1.090023596	-0.17250602	0.174849	0.339517565
Timm23	11	2.037869884	1.441217218	-0.596652666	0.17489686	0.339565557
Mrps36	39	1.279575834	1.415484863	0.135909029	0.17492146	0.339566298
Spag9	103	1.297998699	1.120993289	-0.17700541	0.17494352	0.339566298
Asph	58	1.090958088	0.982270936	-0.108687152	0.17498092	0.339567558
Slc50a1	14	1.283887317	1.092310821	-0.191576497	0.17499045	0.339567558
Zfp868	12	1.094689865	1.650900126	0.556210261	0.17556883	0.340644851
Thap4	54	0.843474867	1.044527748	0.201052881	0.17568877	0.340832502
Ppp3cc	88	0.858584913	0.841262915	-0.017321998	0.1760193	0.341428588
Scaf11	120	0.96514591	1.005618082	0.040472172	0.17607367	0.341443766
Syne2	43	1.378502392	1.121481393	-0.257020999	0.17607268	0.341443766
Siva1	19	2.344371315	1.905834504	-0.438536811	0.17615577	0.34155785
Rnf34	34	1.214521278	1.364666361	0.150145083	0.17627655	0.341701724
Dgki	62	12.08417614	12.94029374	0.856117608	0.17626788	0.341701724
Flrt2	23	20.88958851	20.43555654	-0.454031971	0.17651614	0.342120975
Erap1	31	1.872987751	1.371636959	-0.501350792	0.17660415	0.342246346
Fastkd1	8	0.908295582	0.740532433	-0.167763148	0.17676398	0.342510839
Commd5	5	2.922123559	1.697724892	-1.224398666	0.17682643	0.342586613
Zbtb8os	18	0.909792001	0.620669353	-0.289122649	0.17701703	0.342910604

Rapgef1	116	1.438148093	1.446536411	0.008388318	0.17704983	0.34292889
Def8	19	1.636024376	2.539575461	0.903551085	0.17707595	0.342934204
Polr2d	22	0.89194445	1.180382931	0.288438481	0.17716154	0.343054699
Inpp5k	18	1.135504634	1.408954688	0.273450053	0.17719838	0.343080747
Zfp961	10	7.282882248	6.644668269	-0.638213979	0.17730962	0.343244234
Mrps30	38	0.97656398	1.050502719	0.073938739	0.1773296	0.343244234
Dzip1l	6	3.618759365	5.211846903	1.593087538	0.17737924	0.343295044
Zfp68	32	1.73125529	1.736043666	0.004788376	0.17753659	0.343554261
Kin	6	1.204819277	0.166666667	-1.03815261	0.17761317	0.343611817
Mif4gd	88	1.592418124	1.420175474	-0.17224265	0.1775946	0.343611817
Slc25a32	44	1.148132092	0.852043097	-0.296088995	0.17767544	0.343686974
Zswim8	14	0.944928263	0.917339426	-0.027588837	0.17769991	0.343688996
Pomp	28	0.792811075	0.902700668	0.109889593	0.17773326	0.343708209
Lrrc28	26	1.690239532	1.872272287	0.182032756	0.17780063	0.343793188
Calm2	36	1.245009233	1.440509126	0.195499892	0.17787144	0.343884782
Adamts17	55	2.630403328	2.538998843	-0.091404485	0.17811951	0.344304776
Unc13b	76	1.495085319	1.365145478	-0.129939841	0.1781356	0.344304776
Arntl	59	1.689856644	1.270299583	-0.419557061	0.17828099	0.344540405
Egr4	83	12.3819391	12.74227489	0.360335786	0.17832553	0.34458109
Rpl19	58	6.425321205	5.664276684	-0.761044521	0.17840476	0.344688801
Dos	84	1.33498556	1.115367539	-0.219618021	0.17850155	0.344830404
Pigb	23	1.152244289	0.970608835	-0.181635455	0.17855073	0.344879997
Lipt1	4	2.344367281	2.598533293	0.254166013	0.17867214	0.345069093
Lrrc40	6	1.118859588	1.113314162	-0.005545426	0.17873625	0.345112302
Ube2c	22	1.454198258	1.081082744	-0.373115514	0.17874155	0.345112302
Gclc	56	1.023251352	0.860770997	-0.162480355	0.17879908	0.345132554
Spg20	47	1.307834671	1.396860508	0.089025837	0.17879112	0.345132554
Prdm1	14	1.668484656	0.9568325	-0.711652156	0.178848	0.345181573
Cdc123	10	1.429289241	2.260290483	0.831001242	0.17899904	0.345364736
Ddx19b	27	1.113497504	0.871180101	-0.242317403	0.17898451	0.345364736
Ccbl2	22	1.519561963	1.287978572	-0.231583391	0.17901351	0.345364736
Htr5a	10	24.06294733	29.13571692	5.072769586	0.17909907	0.345417552
Trim24	48	1.295459958	0.771014976	-0.524444982	0.17925274	0.345417552
Hif1an	17	1.295521451	0.955635208	-0.339886244	0.17921488	0.345417552

Tmed9	39	1.723025977	1.305671973	-0.417354004	0.17923389	0.345417552
Abca7	23	8.754097514	7.073142116	-1.680955398	0.17924823	0.345417552
Hyal3	14	1.417877762	1.655701255	0.237823492	0.17922559	0.345417552
Lrpap1	14	1.514365392	1.936462236	0.422096844	0.17919937	0.345417552
Lrrc8d	154	1.828742498	1.868445302	0.039702804	0.17916835	0.345417552
Mtpn	22	1.15478641	0.920020023	-0.234766387	0.17910453	0.345417552
Ido1	18	46.24443421	47.4078147	1.163380494	0.17947548	0.345785986
Trnau1ap	35	1.309519748	1.251471642	-0.058048106	0.17949106	0.345785986
Nup62	5	1.287128713	1.600381151	0.313252438	0.17957238	0.345897226
Snord49a	3	2.25376875	0.649350649	-1.604418101	0.17963937	0.345980852
Ccl25	2	2.980148761	1.905885718	-1.074263043	0.17976561	0.346133121
Fam58b	10	1.72370706	2.06556665	0.34185959	0.17974843	0.346133121
Rxrg	2	11.9047619	6.779661017	-5.125100888	0.179903	0.346310217
Scaper	24	2.429140455	1.601829097	-0.827311358	0.17990478	0.346310217
Snapc4	15	2.846168397	2.354510476	-0.491657921	0.18003453	0.346423646
Gadd45b	41	1.617999462	1.444719701	-0.17327976	0.18000289	0.346423646
Slc35d2	27	1.870266589	1.533046655	-0.337219935	0.180034	0.346423646
Clasp2	97	0.986235422	1.002747893	0.016512471	0.18007479	0.346455683
BC031361	57	1.439549703	0.899918764	-0.539630939	0.18025877	0.346764189
Pacs1	77	1.108767665	0.91336284	-0.195404824	0.18029493	0.346788283
Fstl1	28	33.22799362	35.15134862	1.923354995	0.18043963	0.347021118
Parl	24	1.425468361	1.448815726	0.023347365	0.18051094	0.347112766
Mir190b	5	67.980482	68.08538105	0.104899048	0.18058415	0.347162567
Golga1	32	1.020004447	1.153536152	0.133531705	0.18057797	0.347162567
Clvs2	10	53.68173706	55.49713426	1.8153972	0.18063045	0.347206089
Cep78	73	0.687465743	0.742915279	0.055449537	0.18068104	0.347233508
Ptges3l	55	0.910274508	1.118123734	0.207849226	0.18069204	0.347233508
BC005537	78	1.627129546	1.404004533	-0.223125013	0.18074693	0.347293508
Dnttip2	46	1.155101169	0.865140406	-0.289960763	0.18078618	0.347319876
Rdh1	2	33.86656746	27.87691604	-5.989651423	0.180808	0.347319876
Lap3	50	3.087318204	3.016017308	-0.071300895	0.18086289	0.347379851
732471J01R	2	34.33868763	39.07263162	4.733943994	0.18089337	0.347392928
Gm28042	11	18.01901172	18.53548711	0.516475387	0.18117685	0.34784627
Jmjd7	11	18.01901172	18.53548711	0.516475387	0.18117685	0.34784627

Got1	37	1.368112506	1.253357911	-0.114754595	0.18129619	0.348029857
.90002N15F	163	1.085452046	1.134580549	0.049128503	0.1813773	0.348140025
Ercc6l2	20	1.359121133	0.974398646	-0.384722487	0.18144408	0.348222658
Nom1	18	1.394551424	0.855551365	-0.53900006	0.18156919	0.348371632
Wdr38	7	7.827489655	11.06116741	3.233677757	0.18155104	0.348371632
Bhlha9	13	23.64085206	24.61363293	0.972780868	0.18163006	0.348442866
Gm13157	2	2.173913043	3.426406989	1.252493946	0.18172646	0.348582235
Arel1	10	1.275291566	1.664999543	0.389707977	0.18190411	0.348877387
Mme	28	12.98590207	12.7745372	-0.211364878	0.1821248	0.349157358
Zbtb3	3	1.088435374	1.621315193	0.532879819	0.18214526	0.349157358
Mtl5	45	1.161013821	1.149369691	-0.01164413	0.18213315	0.349157358
Mib2	7	2.116437958	0.840336134	-1.276101824	0.1821019	0.349157358
Sco2	33	2.156145428	2.501590497	0.345445069	0.18225917	0.34933007
Rcor3	98	1.672563286	1.572708496	-0.09985479	0.18235785	0.349473558
Cdc42ep1	74	1.824164925	1.405634727	-0.418530198	0.18239508	0.349499261
Eif3f	20	1.652236668	1.318638906	-0.333597762	0.18242651	0.34951385
Ptpra	69	0.965364274	0.996788166	0.031423892	0.1824641	0.349540226
Setdb1	11	0.32208795	0.699221109	0.377133159	0.1825603	0.349678877
Mir6516	13	6.288858343	4.348295373	-1.94056297	0.18261876	0.349733172
Gm5434	13	98.02142272	97.63100595	-0.390416769	0.18263632	0.349733172
Nit1	6	2.243589744	1.282775769	-0.960813975	0.18273312	0.349872882
Nefh	36	11.68871679	11.30861211	-0.380104675	0.182863	0.35007589
Pigm	33	1.188406451	1.279403761	0.09099731	0.18291515	0.350130044
Use1	13	1.324127123	1.685458371	0.361331248	0.18303457	0.350312945
Eif6	27	1.681215037	1.468124032	-0.213091005	0.1830601	0.350316106
Klk5	3	27.00812473	23.45016954	-3.557955184	0.18309367	0.35033467
Il12rb1	3	54.53832151	51.12781955	-3.410501963	0.18332975	0.350694917
Siae	11	1.259106587	1.521572019	0.262465433	0.18332493	0.350694917
Lsm10	1	1.149425287	0	-1.149425287	0.18348509	0.350854868
Gm14322	3	50.20131334	46.89289805	-3.308415288	0.18348324	0.350854868
Tmem98	24	1.504606571	1.345455908	-0.159150663	0.18345998	0.350854868
)10109A12F	21	1.265319517	1.431256967	0.16593745	0.18356805	0.350956665
Coprs	59	0.973064811	1.054915532	0.081850721	0.18358616	0.350956665
Rps29	3	0.574712644	2.360066834	1.78535419	0.18367087	0.351072853

Mrpl21	8	1.909924992	1.898250575	-0.011674417	0.18371594	0.351113267
Scoc	43	1.349687735	1.241574518	-0.108113217	0.18375038	0.351133361
Fads3	79	1.606766185	1.67311866	0.066352475	0.18391502	0.351315498
Spats2	107	1.205778506	1.337190047	0.131411541	0.18391752	0.351315498
Capn7	60	1.282957117	1.305089562	0.022132445	0.18389374	0.351315498
Nanp	35	1.259230791	1.480559756	0.221328965	0.18395754	0.351346206
Gfer	51	1.114836215	1.22106914	0.106232925	0.18400615	0.351393312
Rps6kb2	10	1.499721735	1.518649869	0.018928134	0.18417662	0.351673087
Tmem109	19	1.122018307	1.300738246	0.178719939	0.18433066	0.351921425
Gfm2	15	0.974724363	1.574803518	0.600079155	0.18438331	0.351945413
Orc3	12	1.643354575	1.171451575	-0.471903	0.18439119	0.351945413
Orc2	48	1.709689406	1.283618137	-0.426071269	0.18442387	0.351962002
Kdm4d	7	0.592193077	1.004474273	0.412281196	0.18454146	0.352094838
Ykt6	58	1.235766502	1.032423432	-0.20334307	0.18452395	0.352094838
Prcc	35	1.729558655	1.26528407	-0.464274586	0.1846391	0.352235331
Tom1l1	27	5.697460769	6.15750493	0.460044161	0.18478649	0.352424871
[illegible]00004C02F	36	1.072732451	0.615569806	-0.457162645	0.18477583	0.352424871
Gcfc2	65	1.215911069	1.027644768	-0.188266301	0.18510565	0.352987698
Fbxo33	149	1.199026301	1.081483992	-0.117542308	0.18517113	0.353066677
AW146154	7	0.777633684	1.52316042	0.745526735	0.18523531	0.353143163
Tubgcp5	11	2.036054831	1.336071136	-0.699983695	0.18530927	0.353192394
Mllt11	19	2.227828938	2.07536993	-0.152459009	0.18529412	0.353192394
Tsr3	30	0.977784158	1.12759415	0.149809992	0.18537994	0.353235317
Vps33a	24	22.00189302	22.5970421	0.595149073	0.18537463	0.353235317
Oma1	38	1.34868415	1.477625772	0.128941622	0.18543048	0.353239898
Fam204a	15	0.742272482	0.43765303	-0.304619452	0.18542634	0.353239898
AW554918	47	1.245521515	1.414412825	0.16889131	0.18567282	0.353655631
Col2a1	48	24.50100806	25.60721592	1.106207859	0.18582609	0.353901635
Ttf1	37	1.488974999	1.427754228	-0.061220771	0.18596559	0.354121352
Csk	82	5.3181501	4.428371506	-0.889778593	0.18610863	0.354347736
Opn3	32	1.287853087	1.534210252	0.246357165	0.18620674	0.354488549
Nol10	20	1.62347843	1.588501642	-0.034976788	0.18633396	0.354684723
Aldh4a1	16	1.88548223	2.622027228	0.736544998	0.18647309	0.354903511
Exoc7	19	0.85520083	1.633887786	0.778686956	0.18651313	0.354933696

Tctn3	19	1.776949245	1.240078403	-0.536870842	0.18655525	0.354967808
Prdm9	15	0.976781955	0.476538148	-0.500243807	0.18661912	0.355043296
Pgs1	41	1.828794291	1.743570303	-0.085223988	0.18665747	0.355070234
Rsph3b	10	1.480238562	1.110785754	-0.369452808	0.18674388	0.355188562
Cttnbp2nl	55	1.591862013	1.530637439	-0.061224574	0.18680984	0.355267967
Kat8	6	9.309985635	7.095428762	-2.214556873	0.18687881	0.355353073
Oxld1	24	1.380928265	1.704648661	0.323720396	0.18696194	0.355458729
Pds5a	207	1.104554659	0.956875225	-0.147679435	0.18698282	0.355458729
Stip1	86	1.552222888	1.349987066	-0.202235822	0.18701066	0.355465593
Cd63	54	2.170546885	1.624422269	-0.546124617	0.18706419	0.355521302
Erlin1	56	1.214214605	1.008958423	-0.205256182	0.18710286	0.355548738
Atp5e	25	1.089841715	1.330014966	0.240173251	0.1871929	0.355673782
Gpd2	66	1.860608705	1.674445121	-0.186163584	0.1877107	0.356611446
Dnajc5	59	1.047619429	1.250346321	0.202726892	0.18780496	0.356744338
Plvap	4	46.75544278	43.32206374	-3.43337904	0.18790707	0.356892094
Psors1c2	1	14.60674157	8.8	-5.806741573	0.18797876	0.356982058
Skiv2l	28	0.939514537	1.408366487	0.468851951	0.18818245	0.357198522
Naa15	67	1.44558609	1.118599885	-0.326986205	0.18819012	0.357198522
Rnmt	14	2.302352375	3.282978029	0.980625654	0.18814451	0.357198522
330415G19F	14	2.097138038	1.416751259	-0.680386778	0.18815068	0.357198522
Zfp91	5	1.549866114	0.692127597	-0.857738517	0.18831051	0.357334603
Zfp91Cntf	5	1.549866114	0.692127597	-0.857738517	0.18831051	0.357334603
Mir3082	14	62.62316284	63.85474475	1.231581915	0.18836128	0.35738472
Syp	1	89.71962617	94.68085106	4.961224896	0.18847595	0.357556059
Plekhm3	35	1.017755305	1.310187298	0.292431993	0.18865234	0.357844416
Pim3	111	1.087661199	0.873546371	-0.214114828	0.18881548	0.358049617
Smyd4	14	0.398436515	0.796854734	0.398418219	0.18880624	0.358049617
330408C21F	88	0.712021278	0.844744153	0.132722875	0.18883372	0.358049617
Cog5	15	1.391023867	1.675785085	0.284761218	0.18887183	0.358075605
Nek2	49	1.380887912	0.932909937	-0.447977975	0.18894795	0.358127386
Catsper2	41	1.031083437	1.241431403	0.210347966	0.1889394	0.358127386
Zfyve26	7	1.980745706	1.466130293	-0.514615413	0.18899572	0.358141698
Gpc1	127	1.585461174	1.559130214	-0.02633096	0.18900431	0.358141698
Xpa	66	1.082552079	0.970471447	-0.112080632	0.18904428	0.358171182

Msrb1	14	3.065937771	3.200188734	0.134250963	0.18914927	0.358323832
[illegible]10025M15[illegible]	89	1.222218535	1.338070257	0.115851722	0.18926911	0.358504564
Smc2	14	1.554577235	1.0352415	-0.519335735	0.18929913	0.358515151
Ankzf1	5	1.234567901	0.215053763	-1.019514138	0.18939576	0.358651874
Anapc1	27	1.720520546	1.819611006	0.099090459	0.18955319	0.358857369
Gm8363	6	93.167307	89.45977275	-3.707534247	0.18953293	0.358857369
Ndst2	29	0.78007949	0.86687737	0.08679788	0.18961794	0.358933643
Gm8221	3	71.91484085	75.86018634	3.945345486	0.18969536	0.359033888
Nfatc2ip	31	1.508842142	1.203868177	-0.304973965	0.1897643	0.35911805
Fubp3	14	1.856178054	1.418527969	-0.437650085	0.18988814	0.35930606
Xxylt1	57	1.649839065	1.232024053	-0.417815012	0.18991506	0.359310671
Snx14	6	1.032908203	0.254452926	-0.778455277	0.19001444	0.35940601
Arfgef2	25	1.382170018	1.835949405	0.453779388	0.18999472	0.35940601
Ccdc71	35	1.356851569	1.134032141	-0.222819427	0.19013968	0.359550223
Fam65c	2	14.11464273	17.46209841	3.347455674	0.1901385	0.359550223
Zdhhc3	20	1.031537141	1.600728539	0.569191398	0.19027044	0.35975111
Gm166	7	1.48261664	2.032590094	0.549973454	0.19029528	0.359751725
Prrc2a	66	1.29052978	1.61438268	0.3238529	0.19037991	0.359865355
Wdr1	81	1.175759907	1.265801336	0.090041429	0.19044495	0.359941935
Pih1d1	4	1.572327044	1.843965306	0.271638262	0.19050361	0.360006428
Coro1b	15	1.395781488	1.430188225	0.034406737	0.19059733	0.360137172
Sclt1	20	0.800957424	0.648333128	-0.152624296	0.19063042	0.360153308
Eva1b	9	18.87789835	16.25904738	-2.618850979	0.19069649	0.360193499
Trim11	54	1.212726066	1.075354017	-0.137372049	0.19070078	0.360193499
[illegible]30111N05[illegible]	17	3.299457404	2.630207506	-0.669249898	0.19080575	0.360345381
[illegible]10008L16[illegible]	12	1.375804542	1.108426461	-0.267378081	0.1908816	0.360442235
Arid1a	162	1.232353566	1.246572626	0.01421906	0.19126222	0.361114496
Sf3b1	38	1.286692042	1.48206661	0.195374568	0.19139251	0.361312481
[illegible]30027C09[illegible]	17	1.213479618	0.883446438	-0.33003318	0.19141633	0.361312481
Rcl1	78	1.807181981	1.366998092	-0.44018389	0.19161664	0.36164406
Crnkl1	22	1.227002393	1.191450129	-0.035552264	0.19166017	0.361679689
Crispld2	14	28.67004027	31.27880614	2.608765873	0.19177617	0.361852058
Cd93	3	63.68845316	66.74650966	3.058056498	0.1918115	0.361872192
Scara3	44	2.413037447	1.838199793	-0.574837654	0.19192027	0.361981179

Nupr1l	26	1.152604217	1.123230108	-0.02937411	0.1919096	0.361981179
Tbl3	19	1.581204242	1.354118714	-0.227085527	0.19194327	0.361981179
Dctn2	43	1.304245749	1.082812583	-0.221433166	0.19196806	0.361981402
Zfp839	56	1.265197092	1.158987439	-0.106209654	0.19206998	0.362127053
Rab28	55	1.984198626	1.64028617	-0.343912456	0.19244859	0.36279427
Hmgb1	65	1.728297129	1.129612527	-0.598684602	0.19273192	0.363271391
Snord58b	31	1.68083756	1.261022246	-0.419815313	0.19277595	0.363271391
Rpl17	31	1.68083756	1.261022246	-0.419815313	0.19277595	0.363271391
Gm14420	2	10.51136364	13.14636554	2.635001907	0.1928937	0.363446608
Dimt1	24	4.429515654	4.747879015	0.318363361	0.19307308	0.363691186
Dmr	40	1.579026226	1.507178367	-0.071847859	0.19306856	0.363691186
Csnk1d	53	1.530284683	1.484391107	-0.045893576	0.19315859	0.363805556
Snora65	1	6.25	9.333333333	3.083333333	0.19326648	0.363962046
Mirlet7i	184	1.26654126	1.175926687	-0.090614573	0.19331108	0.363999323
Cpz	14	14.72614468	11.68204265	-3.044102033	0.19348108	0.364272685
)30432K21F	16	1.393046118	1.390380323	-0.002665796	0.1939087	0.365030944
Mief1	40	1.214358375	1.14289431	-0.071464064	0.19423236	0.365593331
Psmb7	13	1.76694769	1.222031131	-0.544916559	0.19429672	0.365667577
Cramp1l	29	1.428871667	0.990960174	-0.437911493	0.19446373	0.365934945
Upf3b	4	22.49057111	25.43308945	2.942518335	0.19451549	0.365985425
Gpx4	74	3.405914143	3.177745548	-0.228168595	0.1945534	0.366009821
Myh9	52	0.994512769	1.078747341	0.084234572	0.19463197	0.366110702
Psme4	73	1.41996454	1.358020665	-0.061943875	0.19470616	0.366203298
Stk38	57	1.324543573	1.24983722	-0.074706353	0.19482979	0.366341911
Zmynd11	66	1.376772656	1.143566653	-0.233206003	0.19480574	0.366341911
Alg3	12	2.049296967	1.609604265	-0.439692702	0.19497715	0.366572029
Tmem117	62	1.487644335	1.160835224	-0.326809111	0.19516457	0.366877396
Gcnt4	36	0.942315536	0.814109529	-0.128206007	0.19529376	0.367073226
Ubald2	23	0.759660614	1.17194523	0.412284616	0.19542088	0.367124055
Smad3	80	1.227076214	1.079148571	-0.147927643	0.1953662	0.367124055
Rae1	64	1.325057086	0.955830474	-0.369226612	0.19540587	0.367124055
Snord14a	2	88.24045634	89.98108589	1.740629552	0.19537265	0.367124055
Ldlrad3	83	1.140669057	1.144050782	0.003381726	0.19552968	0.367281428
Tmem5	40	0.915279075	0.607237477	-0.308041598	0.19589308	0.367817409

Ncapg	52	1.035232298	0.825349355	-0.209882943	0.19589596	0.367817409
Coro1c	105	1.128999947	1.267523155	0.138523208	0.19586213	0.367817409
Dgkeos	4	1.502438325	0.506756757	-0.995681568	0.19591528	0.367817409
?10015D19F	18	1.349547017	1.567729928	0.218182911	0.19620772	0.368319307
;10009B15F	6	1.894977169	0.386603061	-1.508374108	0.19629032	0.368427247
Asns	10	1.406929412	0.964822593	-0.442106819	0.1964391	0.368654665
Sec23ip	29	1.360124942	1.572167464	0.212042522	0.19646173	0.368654665
Lsp1	2	12.54385965	7.920792079	-4.62306757	0.19655779	0.36878776
Cbfa2t3	138	6.417169141	6.406446616	-0.010722525	0.19660994	0.368838446
Avl9	66	1.177007284	0.953336469	-0.223670815	0.19681335	0.369099574
Dusp8	60	2.389831643	1.945904011	-0.443927632	0.19684975	0.369099574
Map4k1	2	0.862068966	2.564102564	1.702033599	0.19683179	0.369099574
Ccdc61	1	9.411764706	16.21621622	6.80445151	0.19683984	0.369099574
Usp5	18	2.829534728	2.236030913	-0.593503814	0.19715221	0.369619463
Gtpbp2	13	1.088930292	1.673748534	0.584818242	0.19743909	0.370110023
Lbx2	8	15.37728957	15.1405077	-0.236781866	0.19746725	0.370115525
Ears2	10	0.786187036	1.532220912	0.746033876	0.19756961	0.37026008
Zfp191	96	1.094666613	1.285631868	0.190965255	0.19761686	0.370301333
Cep120	58	1.535040766	1.164401863	-0.370638903	0.19816128	0.371173511
Ehhadh	15	10.08942042	9.922952693	-0.166467723	0.19816707	0.371173511
Gpr176	46	23.07381591	24.9323486	1.858532689	0.19822938	0.371173511
Amigo1	94	1.574050887	1.290531854	-0.283519033	0.19823407	0.371173511
Cldn10	13	19.72901007	18.45581344	-1.27319663	0.19811021	0.371173511
Lekr1	22	1.621648298	1.273269738	-0.348378559	0.19820953	0.371173511
Gm14137	1	31.32911392	26.70807453	-4.62103939	0.19828208	0.371177696
St6galnac4	36	1.70822406	1.713312067	0.005088007	0.1982869	0.371177696
Nelfa	37	1.227018317	0.878793942	-0.348224375	0.19833196	0.37121469
Btg1	55	1.66400147	1.264893957	-0.399107513	0.19836018	0.371220163
Ppm1a	88	1.632297848	1.231950189	-0.400347659	0.19866251	0.37173854
Abhd5	93	1.148230264	0.905881254	-0.24234901	0.19877503	0.371901669
Tpp1	1	5.612244898	3.083700441	-2.528544457	0.19883441	0.371965337
Klhl36	47	1.846056277	1.832478394	-0.013577882	0.1989513	0.372100102
Rad21	66	1.258923218	1.436736858	0.17781364	0.19895716	0.372100102
D8Ertd82e	72	1.865316548	1.261827247	-0.603489301	0.19898971	0.372113544

Arhgap10	61	1.116141337	0.996685233	-0.119456104	0.1990765	0.372133568
720483E21F	9	0.912551971	0.924096915	0.011544944	0.19904984	0.372133568
Ehd4	11	1.120861852	0.180018002	-0.94084385	0.19905238	0.372133568
Dynlrb1	12	2.217611321	2.106379866	-0.111231454	0.19942026	0.372728683
Zc2hc1c	4	22.14631603	19.87537205	-2.270943983	0.19948887	0.37276197
Hnf1b	65	2.29270154	2.007832157	-0.284869383	0.19948489	0.37276197
Aga	8	0.871656536	0.740202412	-0.131454124	0.1997114	0.373082768
Eif3i	19	0.700630882	1.08806055	0.387429668	0.19969019	0.373082768
Cers2	67	1.598491976	1.416045922	-0.182446054	0.19980577	0.373177963
Fam189b	2	2.544017418	3.903987808	1.35997039	0.19981322	0.373177963
Ly6i	2	98.37065174	94.09722222	-4.273429517	0.19997559	0.373433667
Nudt22	7	7.577187866	4.950787691	-2.626400175	0.20007321	0.373568434
Ier5l	60	1.585399901	1.254076188	-0.331323713	0.20010817	0.373586167
Telo2	4	1.801794129	2.670454545	0.868660416	0.20027394	0.373848083
Zfp770	12	1.116741622	0.68822648	-0.428515142	0.20045387	0.374136363
Ndufa8	31	1.478085643	1.518546053	0.04046041	0.2006594	0.374472342
Dcun1d5	56	1.4721785	1.430264739	-0.041913761	0.20087544	0.374827852
700060E02F	28	1.594953493	1.182940425	-0.412013068	0.20094396	0.374908027
Traf2	50	1.181454138	1.004380583	-0.177073555	0.20109343	0.375139208
Sfswap	18	1.334386879	1.107447274	-0.226939605	0.20114826	0.375193799
Best3	3	84.39554055	86.30175049	1.906209941	0.20136295	0.375498777
Ggnbp2	34	1.833174496	1.526661081	-0.306513415	0.20134485	0.375498777
930413G21F	78	1.258919503	1.368932308	0.110012805	0.2015854	0.375865839
.30034C11F	6	0.985663082	1.317173272	0.331510189	0.20185585	0.376322283
Socs1	95	1.505156848	1.31272115	-0.192435697	0.20188534	0.376329458
Clcn7	25	4.900523061	5.230278108	0.329755046	0.20201876	0.376530322
Ccdc134	38	4.473713681	3.49664456	-0.97706912	0.20212801	0.37663828
Acss2	37	1.174608054	1.403970689	0.229362635	0.20210988	0.37663828
Naa60	47	1.763786981	1.123203473	-0.640583508	0.20237631	0.377053072
Lyzl4	44	6.825082961	7.552749251	0.72766629	0.20253653	0.377291688
Tpgs2	63	1.309187151	1.12930411	-0.179883041	0.20255581	0.377291688
Ppil6	8	0.792202804	0.285388128	-0.506814676	0.20264293	0.377384282
Yeats2	41	1.112541687	1.215675676	0.103133989	0.20265695	0.377384282
Tnfrsf1b	5	2.545743618	1.447653374	-1.098090244	0.20274022	0.377491438

Nudcd1	24	1.609881761	1.136124569	-0.473757192	0.20325244	0.378397144
Fchsd2	85	1.329255298	1.488946675	0.159691377	0.20383375	0.379431236
Piezo1	88	3.423176505	3.191113353	-0.232063151	0.2040823	0.37980953
Cdc45	11	1.349251867	0.701379313	-0.647872554	0.20408874	0.37980953
t(ROSA)26Sc	88	1.29357614	1.249953074	-0.043623066	0.20414368	0.37986361
Nek9	38	1.160799597	1.077424205	-0.083375391	0.20422394	0.379921169
Cs	47	1.09022388	1.649852354	0.559628474	0.2042264	0.379921169
Mir343	3	99.65277778	98.66666667	-0.986111111	0.20427779	0.379955784
Osbp	111	1.116976822	1.181482632	0.06450581	0.20432269	0.379955784
Nudt5	16	1.740058182	2.087985848	0.347927666	0.20431479	0.379955784
Ppat	29	1.361979706	1.369853531	0.007873825	0.20442061	0.38008971
Smcr8	17	1.312832346	1.08713072	-0.225701626	0.20457775	0.380333696
Plekhg3	45	0.826382475	1.044717396	0.218334921	0.20469738	0.380507896
H2afx	62	1.110067398	0.960151012	-0.149916386	0.20473412	0.380527981
Eprs	68	1.184706171	1.424287411	0.23958124	0.20479974	0.380601743
Snd1	30	1.699912445	1.505464786	-0.194447659	0.20497667	0.38087642
Rab11a	24	1.56785955	1.10236854	-0.46549101	0.20499945	0.38087642
Fam84a	18	3.94564027	3.586069233	-0.359571037	0.20502808	0.380881383
Appl1	67	0.962451895	0.775749592	-0.186702303	0.20505796	0.38088867
AU019823	7	0.313971743	1.019572553	0.705600811	0.20508559	0.380891776
Dnph1	41	1.769837187	1.599511654	-0.170325533	0.20514309	0.380950353
Ppp1r13b	111	3.723336007	3.439240322	-0.284095685	0.20524342	0.381049307
Cdkl1	26	1.833375139	2.174237368	0.340862229	0.20524831	0.381049307
Erc1	45	1.231754745	1.524929019	0.293174274	0.20552791	0.381520117
Fanca	14	0.72504116	1.166696306	0.441655146	0.20558288	0.381573896
Itpa	19	1.847274539	1.567823141	-0.279451398	0.20567225	0.381691482
Cep152	48	1.01154538	1.212878537	0.201333157	0.20596135	0.382179677
Pcdhgb4	5	55.72097797	58.75248383	3.031505865	0.20603359	0.382265381
Naa50	84	0.981188573	1.207693209	0.226504635	0.20631684	0.382742525
Lrrc4b	5	5.79697142	5.533263033	-0.263708387	0.20635828	0.382771006
Rtbdn	26	15.4147838	14.92359601	-0.491187786	0.20656958	0.383114511
Abcf1	15	1.193351451	0.549178305	-0.644173146	0.2067364	0.383375455
Eef2k	23	3.87534772	2.562017206	-1.313330514	0.20685351	0.383544158
Mir219c	44	1.447940931	1.212150236	-0.235790694	0.20688076	0.383546206

Anapc2	5	1.456858117	1.866784723	0.409926606	0.2069762	0.383561001
Hsp90ab1	44	1.041228966	1.212257764	0.171028799	0.20695325	0.383561001
Kif20a	23	1.255360501	1.231278091	-0.02408241	0.2069241	0.383561001
Ccdc50	26	0.92248789	1.105006523	0.182518633	0.20699329	0.383561001
Prmt10	11	1.582262576	1.231132202	-0.351130375	0.20709854	0.38370757
Rpl7l1	12	1.348404504	0.793776402	-0.554628103	0.2072624	0.383962685
700105P06R	64	1.199311867	1.290855638	0.091543771	0.20734164	0.384060996
Nup50	115	0.969465835	1.138632202	0.169166367	0.20746498	0.38420065
Pou2f2	12	1.934041077	1.1066433	-0.827397777	0.2074694	0.38420065
Rpf1	56	1.17928828	1.350248427	0.170960147	0.20759126	0.384377804
Pgam1	40	0.801290875	0.853252688	0.051961813	0.20765665	0.384450368
Tubgcp4	52	1.375921769	1.41402338	0.038101612	0.20820588	0.385418575
Gm1821	11	0.199194745	0.779568085	0.580373341	0.20842513	0.385727125
Ubb	11	0.199194745	0.779568085	0.580373341	0.20842513	0.385727125
Nhp2l1	52	0.86813252	0.675916151	-0.19221637	0.20848511	0.385789476
Trp53tg5	1	91.89189189	88.12785388	-3.764038011	0.20858953	0.38588538
Tbc1d9b	31	0.978738424	0.667069361	-0.311669063	0.20857716	0.38588538
Tubb5	30	1.073137012	1.274023258	0.200886246	0.20869949	0.386040131
Lmbrd1	20	1.364158521	1.463954488	0.099795967	0.20891794	0.386395501
Myl4	10	1.740443366	1.047548512	-0.692894854	0.20951509	0.387451089
00029M09R	50	1.536762981	1.273570429	-0.263192552	0.20970364	0.387729024
Ubxn6	43	1.591690513	1.583128486	-0.008562027	0.20974465	0.387729024
Bnip3	19	3.862982144	2.513866056	-1.349116088	0.20973463	0.387729024
Pdgfrb	3	27.14502385	24.09747008	-3.047553776	0.20981485	0.387809937
Dyrk3	44	1.111969093	0.990187894	-0.121781199	0.20993565	0.387886646
Spock1	55	53.50479239	51.89512281	-1.609669572	0.20989177	0.387886646
Acadm	29	1.900253009	1.344447717	-0.555805291	0.20991157	0.387886646
Adamts10	17	1.196659305	1.450408612	0.253749307	0.20998852	0.387887443
Homer3	64	1.414746735	1.261996869	-0.152749866	0.20998895	0.387887443
Sephs1	74	1.360836219	1.17410581	-0.186730409	0.21005782	0.387965828
Lhx2	98	10.47992914	11.08074614	0.600817009	0.21021049	0.388198941
Fam101a	7	15.51023855	18.0609298	2.550691243	0.21026368	0.388248303
Ucn	16	42.32511408	43.49142428	1.166310203	0.21044067	0.388526219
Zfp655	61	0.896562975	1.065569185	0.169006211	0.21048328	0.388556

Naglu	16	1.578625646	1.58468977	0.006064124	0.21067029	0.388852315
Slc20a2	69	1.334970728	1.177577187	-0.15739354	0.21070015	0.388858502
Hspa4	65	1.470157137	1.180715495	-0.289441642	0.21080438	0.389001952
Slc7a6	81	1.489788106	1.484719635	-0.005068471	0.21092059	0.389167444
Emc9	13	1.454854961	1.756017809	0.301162848	0.21097627	0.389221252
BC004004	35	8.739421103	7.902671316	-0.836749786	0.21120458	0.389348744
Lrrc45	10	1.4873017	2.014909334	0.527607633	0.21113514	0.389348744
Ak3	40	1.021536704	1.024498774	0.002962071	0.21109046	0.389348744
Ift57	9	1.375661376	1.743289948	0.367628573	0.21120224	0.389348744
Stra13	10	1.4873017	2.014909334	0.527607633	0.21113514	0.389348744
Ino80b	16	1.47914825	0.762175603	-0.716972647	0.21119585	0.389348744
Zc3hav1	33	0.571765763	0.957753612	0.385987848	0.21138751	0.389621062
Dip2b	94	1.962205387	2.079827525	0.117622138	0.2114054	0.389621062
Clps	7	95.27298851	91.23089735	-4.042091152	0.21145803	0.38966912
Utp20	15	1.528122692	1.288301341	-0.239821352	0.21162791	0.389933201
Dmbx1	31	6.007896154	5.437676332	-0.570219822	0.21171262	0.390040303
Chka	50	1.903354494	1.325087893	-0.578266601	0.21174865	0.390057711
Lrrc71	5	79.54462165	78.24910409	-1.29551756	0.21185592	0.390109878
'00042G07F	6	96.76844556	97.76507973	0.996634162	0.21185673	0.390109878
Mlh3	7	2.657156335	3.383932301	0.726775966	0.21182315	0.390109878
Cdkn2d	56	1.898952838	1.399814326	-0.499138512	0.21201638	0.390354872
E2f3	122	1.653993136	1.439688924	-0.214304212	0.21221265	0.390667221
Tln1	34	1.983181216	1.354747227	-0.628433989	0.21244717	0.391049905
Apip	16	1.116768806	1.606614917	0.489846112	0.21276772	0.391590802
Uqcc1	8	0.691573034	1.143350628	0.451777594	0.21279991	0.391600945
Chd7	138	0.894716489	0.934594381	0.039877892	0.21294827	0.391824813
Otud6b	24	1.157341077	1.288528191	0.131187115	0.21300452	0.391846818
Slc9a8	39	1.263147051	1.332877673	0.069730622	0.21303631	0.391846818
Zscan20	16	1.730749292	1.68248503	-0.048264262	0.21304034	0.391846818
Celf2	10	23.4313016	22.46627641	-0.965025189	0.21309161	0.391892005
Kcna5	10	32.50872322	36.83089304	4.322169821	0.21318488	0.392014402
Cenpj	31	1.253485939	1.443618657	0.190132719	0.21335913	0.392285667
Ddx46	66	1.555338765	1.292127748	-0.263211017	0.21346197	0.392425584
l30008F23F	12	1.776610361	2.229765921	0.45315556	0.21388492	0.393153873

Osbpl11	88	1.309499424	1.171329582	-0.138169842	0.21398017	0.393279685
Kif16bos	4	6.744480502	9.690163582	2.94568308	0.2140357	0.393332476
Abcb10	80	1.383537603	1.148336141	-0.235201462	0.21409662	0.393395174
Dopey1	107	1.462770496	1.331792831	-0.130977666	0.21413804	0.393422018
Ncl	21	1.549858164	1.244425212	-0.305432952	0.21440719	0.393867196
Fhod1	30	1.752086694	1.406415247	-0.345671447	0.21444024	0.393878596
Sae1	33	1.074611741	0.836071736	-0.238540005	0.21452625	0.393987265
Epm2aip1	15	1.081452199	0.709885756	-0.371566444	0.21455917	0.393998402
Mcm7	12	2.224586415	1.973988678	-0.250597738	0.21465808	0.394130436
Fzd7	28	1.372280174	1.76237672	0.390096546	0.21468479	0.394130436
Wipi2	63	0.930807754	1.188685245	0.257877491	0.2150406	0.394722316
Zfp65	2	2.667140825	5.931855501	3.264714676	0.21506099	0.394722316
B4galt7	20	2.112529188	1.609577964	-0.502951225	0.21530249	0.394891859
Slc26a6	26	1.873832017	1.437632523	-0.436199493	0.21528483	0.394891859
Mtmr6	30	2.695569577	2.18953986	-0.506029717	0.21525186	0.394891859
Sh3bp5	25	1.70552841	1.197263537	-0.508264873	0.21531483	0.394891859
Ttll13	4	2.755762129	1.968808357	-0.786953772	0.21525788	0.394891859
Zfp938	6	2.689949249	2.777442272	0.087493023	0.21522503	0.394891859
Pten	142	1.596588883	1.414796958	-0.181791925	0.21564169	0.395441906
Rasl12	8	8.175770308	9.732110673	1.556340365	0.21569881	0.395497223
Faap20	11	1.849350459	1.355539865	-0.493810594	0.21580445	0.395641488
Nup43	2	1.041666667	4.411764706	3.370098039	0.21590428	0.395775061
Slc26a5	11	23.10096898	21.73117898	-1.369790001	0.21596093	0.39582947
Psmd14	29	1.713113655	1.424792436	-0.288321219	0.21630473	0.396410096
Kxd1	40	1.316020145	1.229874112	-0.086146033	0.21634103	0.396427115
Akr1e1	4	3.253112899	2.787964796	-0.465148104	0.21639672	0.396430142
Otud3	65	1.396905496	1.25916782	-0.137737676	0.21639508	0.396430142
Ush1g	24	28.76973505	27.22846316	-1.541271894	0.21657875	0.396714084
Senp5	46	1.602941651	1.253472766	-0.349468885	0.21667684	0.39684423
Rfx8	35	35.57376003	35.09235062	-0.481409408	0.21672807	0.396888522
Hook2	12	1.511728068	1.012852113	-0.498875955	0.21704041	0.397410896
Gm15217	2	82.06293596	73.04304534	-9.019890616	0.21716379	0.397587209
Naca	19	0.785990553	0.805411985	0.019421432	0.21733373	0.397848691
Gadd45a	30	0.674602819	1.004564348	0.329961528	0.21744639	0.398005275

Upf1	56	0.853995193	0.942159695	0.088164502	0.21758374	0.398207018
Ptges3	67	1.075103798	0.895920218	-0.17918358	0.217664	0.398304211
Hivep1	95	1.036473477	0.798904331	-0.237569146	0.21787469	0.398590345
Zfyve1	39	1.871393605	1.738809348	-0.132584257	0.21785782	0.398590345
Dusp7	95	1.284707169	1.244767714	-0.039939455	0.21793972	0.398659526
Nolc1	41	1.214316647	0.933526326	-0.28079032	0.21796684	0.398659526
Egln1	171	1.459553665	1.346626308	-0.112927358	0.2180591	0.398778561
Zfp42	8	89.74502687	89.01055508	-0.734471788	0.21811636	0.398833585
Cdt1	26	1.365670405	1.045005062	-0.320665342	0.21834489	0.399201717
310013P06F	96	1.272372915	1.341584107	0.069211192	0.21855968	0.399517239
Tgfbrap1	7	0.429645542	0.824944308	0.395298766	0.21860655	0.399517239
Pih1h3b	9	52.19334628	49.77628264	-2.417063637	0.21862637	0.399517239
Pdxk	37	1.773881818	2.213702965	0.439821147	0.21862179	0.399517239
Rtfdc1	21	1.884187747	1.329336434	-0.554851313	0.21871065	0.39962147
Dpy19l1	166	1.307885563	1.106767961	-0.201117602	0.21882867	0.399747114
Arl4c	142	1.212217608	1.061667112	-0.150550496	0.21886114	0.399747114
110004F10F	22	11.31273799	11.701721	0.388983005	0.21883935	0.399747114
Coq3	35	1.551144479	1.676845131	0.125700652	0.21894927	0.39985831
Acsl3	83	1.021190674	1.059822528	0.038631854	0.2190479	0.399938886
Utp14b	83	1.021190674	1.059822528	0.038631854	0.2190479	0.399938886
Nup188	20	1.632994292	1.152612789	-0.480381503	0.21916105	0.400095702
Gnb2l1	30	1.586354957	1.466853276	-0.119501681	0.21918879	0.40009656
Naa30	97	1.548513898	1.381307411	-0.167206486	0.21923307	0.400127623
Smyd5	19	1.632274598	1.091104907	-0.541169691	0.21936477	0.400318192
Igsf3	87	1.507821519	1.533580207	0.025758688	0.21951985	0.400551397
Abca9	3	26.6093613	30.57251908	3.963157781	0.21962788	0.400685094
Olfr329-ps	1	98.42105263	96.55172414	-1.869328494	0.21964774	0.400685094
Ambp	3	86.96640256	83.54964651	-3.416756043	0.21980089	0.400914636
Epas1	59	2.769433532	2.683461609	-0.085971923	0.22009056	0.401393108
Iqck	41	1.265988438	1.179265494	-0.086722944	0.22024746	0.401579436
Gorasp1	10	1.411850856	2.156016567	0.744165711	0.22024276	0.401579436
Exd2	5	0.516150516	1.760935143	1.244784627	0.22043714	0.401875335
Pofut1	52	1.121168433	1.005464433	-0.115704	0.22053793	0.402009146
Asf1a	70	1.298514045	1.044963023	-0.253551022	0.22062877	0.402124775

Clpp	12	1.527736344	1.248120899	-0.279615445	0.22066769	0.402145757
Ubxn2b	31	1.568435151	1.374286067	-0.194149084	0.22075054	0.402246794
Nfkbid	2	11.44932238	6.027170788	-5.422151588	0.22094881	0.402558082
Rhebl1	15	1.687658922	1.060044321	-0.627614601	0.22103394	0.402663197
Trip11	31	1.025405083	1.231274465	0.205869382	0.22161311	0.403668168
Eif2d	6	2.071404404	1.923863834	-0.147540571	0.22164929	0.403683953
Nabp2	11	1.640258068	1.976065339	0.335807271	0.22171134	0.403746853
Hsd11b2	53	1.956073111	1.906488219	-0.049584892	0.2218252	0.403853977
Hmgcs1	43	1.433217106	1.126154108	-0.307062998	0.22181078	0.403853977
Vipas39	8	0.887483995	1.954098471	1.066614476	0.22233786	0.404686897
Tnip1	3	92.88218872	95.24928519	2.36709647	0.22233682	0.404686897
Cntrob	9	0.991508771	0.78998102	-0.20152775	0.22244077	0.404723607
Mtmr1	37	15.86188884	15.29940807	-0.562480774	0.22244067	0.404723607
Ttc13	38	1.897062966	1.487694776	-0.409368191	0.22239336	0.404723607
Zfp646	19	1.113896215	2.00063659	0.886740375	0.22250586	0.404791843
'00120K04F	22	90.26819223	90.63681768	0.368625442	0.22262651	0.404961126
Atl3	46	1.626893697	1.869807579	0.242913882	0.22266372	0.404978614
Dbt	19	2.540449469	1.873547661	-0.666901808	0.222738	0.405029354
230020J21R	18	1.073365779	1.28669517	0.213329391	0.22274682	0.405029354
Fdxacb1	30	1.479726687	1.251430512	-0.228296175	0.22279187	0.40506108
Wdr70	36	1.067419738	1.215579268	0.14815953	0.22291936	0.405242657
430025E21R	22	1.060670245	1.065984181	0.005313936	0.22300544	0.405348911
Sphkap	2	10.78431373	8.928571429	-1.855742297	0.22310307	0.405476161
Nfix	136	1.781915377	1.167934404	-0.613980973	0.22326736	0.405724492
Grip1os2	3	70.09801193	74.68797565	4.589963717	0.22344375	0.405994757
Nr2c1	25	1.425755169	1.524525834	0.098770665	0.22349982	0.406004864
Carhsp1	51	1.881196241	1.623907398	-0.257288843	0.22350465	0.406004864
Aen	28	0.638908271	1.098825202	0.45991693	0.22362592	0.406174863
Fbxw7	107	5.321330288	5.573806775	0.252476487	0.22388512	0.406595329
Mafg	106	1.429090405	1.283502415	-0.14558799	0.2239903	0.406736004
Myd88	17	2.748279669	1.917169544	-0.831110126	0.22405332	0.406800095
Hgh1	55	3.384333668	3.184441175	-0.199892493	0.22410617	0.406845724
Errfi1	69	1.251899904	1.209509827	-0.042390077	0.22423913	0.407036731
Xpo4	60	1.071281896	1.100925493	0.029643597	0.22438122	0.407244277

Arfrp1	26	1.807532508	1.878322255	0.070789747	0.22480126	0.407956181
Sucla2	60	1.43639042	1.239316679	-0.197073741	0.22483287	0.407963098
Mllt4	155	1.506732459	1.187456587	-0.319275872	0.22491536	0.408011859
Ube2e1	63	1.373729009	1.16664386	-0.207085149	0.22489514	0.408011859
Cstf2	63	20.4273744	19.11660504	-1.310769353	0.22497158	0.40801297
Mitd1	20	1.06511099	1.137135468	0.072024478	0.22494488	0.40801297
30102H24F	104	1.337464159	1.143888993	-0.193575166	0.22518637	0.408352043
Glmn	22	1.257652948	0.969049584	-0.288603364	0.22555245	0.408965356
Myo1d	56	1.574719205	1.405990752	-0.168728452	0.22599704	0.409720855
Nkiras1	43	1.508518217	1.336029177	-0.17248904	0.22609668	0.409850859
Eif5b	106	1.148623607	1.306847405	0.158223798	0.22617315	0.409938835
Col25a1	9	50.12048648	54.61919916	4.498712679	0.22621838	0.409970168
Cdan1	62	1.192278233	0.887169951	-0.305108282	0.22625889	0.409992959
Fkbp8	32	2.172957431	1.996610382	-0.176347049	0.22630113	0.410018867
Idh2	65	1.37554139	1.399462708	0.023921318	0.22637697	0.410105643
Mrps17	9	2.574899471	1.370158472	-1.204740998	0.22656432	0.410394383
Hagh	49	1.474471523	1.338765156	-0.135706367	0.22669206	0.410575075
Foxd2os	46	1.543314171	1.559561898	0.016247727	0.22731077	0.411543269
Aes	62	1.799553179	1.608370663	-0.191182516	0.2272842	0.411543269
Nr1d2	60	1.381473992	1.330414141	-0.051059852	0.22726681	0.411543269
Ctso	16	2.046737385	1.43040804	-0.616329345	0.22755475	0.41193417
Ydjc	5	1.703383163	1.116606219	-0.586776944	0.22759076	0.411948543
Ccna2	66	1.167859645	1.159337584	-0.008522061	0.2276405	0.411987749
Oxnad1	9	1.813104984	0.921120913	-0.891984071	0.22768101	0.412010257
Zfp511	21	0.841347128	1.12171676	0.280369632	0.22785163	0.412221834
Cse1l	48	0.825273293	0.928094964	0.102821671	0.22785412	0.412221834
Krr1	27	1.023167955	1.362128612	0.338960657	0.22808766	0.412593484
Rab27a	6	3.439554148	2.017033477	-1.422520671	0.22819537	0.412737437
Traf5	48	1.512018716	1.076136108	-0.435882608	0.22836561	0.412742914
Cks2	35	1.148764418	1.188975689	0.040211271	0.22843699	0.412742914
Pcyt2	70	19.34737984	18.81777356	-0.529606281	0.228276	0.412742914
Tmem261	27	1.662182288	1.465097434	-0.197084854	0.22845154	0.412742914
Atl2	66	1.381796441	1.320597033	-0.061199408	0.22838401	0.412742914
Fh1	51	1.101181525	0.926339593	-0.174841932	0.22836863	0.412742914

Ppcs	32	0.83646765	1.06110934	0.224641689	0.22836747	0.412742914
Ascl1	26	19.63520784	19.71161405	0.07640621	0.22843123	0.412742914
Slc35e1	130	1.104623524	0.92681597	-0.177807554	0.22830568	0.412742914
Ube2n	129	1.258441847	1.239173457	-0.01926839	0.22872313	0.413182722
Pianp	28	11.61465373	12.1944242	0.579770468	0.22881433	0.413296581
Ctdspl	125	1.499812699	1.246699353	-0.253113346	0.22901558	0.41359789
Gulp1	23	42.92104629	41.85334683	-1.067699463	0.22903751	0.41359789
Chordc1	38	1.709805544	1.221948733	-0.487856812	0.22910826	0.413674747
Psma4	7	2.854892914	1.708427231	-1.146465683	0.22949572	0.414217446
700030J22R	22	1.428732289	1.251181388	-0.177550901	0.22952174	0.414217446
)10001A14F	22	1.765448706	1.367875908	-0.397572798	0.22949924	0.414217446
Usmg5	33	0.944854748	0.831966596	-0.112888152	0.22944897	0.414217446
Pafah1b1	115	1.332856965	1.369143366	0.036286401	0.22966082	0.414368153
Pip4k2b	65	1.559553856	1.284302494	-0.275251362	0.22966173	0.414368153
Fam220a	40	1.533288071	1.637885262	0.104597191	0.22990878	0.414711924
Fam171a1	70	1.486727792	1.403677172	-0.08305062	0.22988776	0.414711924
Tsfm	44	1.751083296	1.455246261	-0.295837035	0.23004015	0.414846893
Cux2	20	16.72198035	16.16798747	-0.553992876	0.23003918	0.414846893
230110C19F	11	1.972526998	1.321902915	-0.650624083	0.23010394	0.414910931
Ctr9	32	1.106645508	0.974772049	-0.131873459	0.23039252	0.415380248
430083A17F	58	1.518357786	1.391933161	-0.126424625	0.2304573	0.415446003
C2cd3	33	1.427345559	1.659333285	0.231987727	0.23051264	0.415494722
Ppp1r16a	36	1.459761709	1.199206367	-0.260555342	0.2305674	0.415542376
Snrpd1	23	1.255600801	1.263561703	0.007960902	0.23062071	0.415571937
931429I11R	35	24.46281089	26.08784425	1.625033364	0.23064044	0.415571937
Suclg2	60	1.307474507	1.190798396	-0.116676112	0.23082616	0.41585551
Ubfd1	32	1.928364527	1.552496183	-0.375868344	0.23090332	0.415943453
Ncoa2	147	1.520545947	1.308869216	-0.211676731	0.23093232	0.415944621
Zhx2	45	1.423980899	1.697131341	0.273150442	0.23106813	0.416087092
BC048403	42	1.130672075	1.102329972	-0.028342102	0.23106741	0.416087092
Mis12	22	1.323897732	0.854625159	-0.469272573	0.23130521	0.416411802
Pcdhga9	8	56.06574472	54.26873617	-1.797008556	0.23129145	0.416411802
Mir7033	1	100	98.14814815	-1.851851852	0.23135558	0.416451389
Vsig10	69	1.325640783	1.278636697	-0.047004086	0.23143891	0.416545053

330013P04F	3	4.761904762	2.976190476	-1.785714286	0.23146438	0.416545053
Cpne3	49	1.1665323	1.170216103	0.003683803	0.23149818	0.416554786
Nabp1	25	1.438732054	1.548095218	0.109363164	0.23159955	0.416686102
Xpo6	69	1.305869365	1.358351073	0.052481708	0.23166426	0.416751417
Mir99b	9	86.10556141	87.65206997	1.546508561	0.23179123	0.416928713
Zfp748	4	1.766556939	3.811651804	2.045094865	0.2319803	0.417217664
Usp19	45	1.296714708	1.290995511	-0.005719197	0.23211044	0.417349426
Rnft2	21	0.77324112	0.80025373	0.027012611	0.23208335	0.417349426
30411M01I	4	12.78865891	11.74749483	-1.041164084	0.23214144	0.417354021
Sash3	2	57.30645161	63.44888461	6.142432996	0.23228697	0.417564498
Prr5l	19	3.632175387	2.57371397	-1.058461417	0.23243929	0.417787134
Dusp18	2	2.205882353	1.239669421	-0.966212931	0.23249675	0.41781764
Ccdc107	22	1.18774729	1.24536163	0.05761434	0.23251321	0.41781764
Sumf1	12	0.91508713	0.280862965	-0.634224165	0.23268858	0.418081573
Myod1	17	42.60061407	40.67919301	-1.921421065	0.23284561	0.418312505
930021J03R	77	1.328126945	1.116489975	-0.21163697	0.23300566	0.418548789
C8g	1	7.468879668	4.87804878	-2.590830888	0.23314171	0.418741909
Dhcr24	25	5.090730857	4.482008615	-0.608722242	0.23322456	0.418839457
Rad52	43	1.910183837	1.578180556	-0.332003282	0.23335288	0.419018624
Nme1	13	1.588673028	1.387646111	-0.201026918	0.2334932	0.41921928
Lmcd1	9	2.606299967	1.802636716	-0.803663251	0.2335392	0.41925058
Ensa	11	2.025645997	1.375732269	-0.649913727	0.23387952	0.419758815
Pxylp1	64	1.744945323	2.155210795	0.410265472	0.23385924	0.419758815
Slc8b1	4	1.803665396	0.739686411	-1.063978985	0.23392275	0.419785066
Atg4d	36	1.751436903	1.466224314	-0.285212589	0.23395661	0.419794491
Ndufs6	21	1.249322472	1.452394491	0.20307202	0.23407066	0.419896441
Lrfn3	27	5.318598352	6.345792201	1.027193849	0.23404854	0.419896441
Aamp	19	1.910299545	1.497338556	-0.412960989	0.23436303	0.420369518
Ube3b	19	1.666716772	1.709298869	0.042582096	0.23489369	0.421218387
Smad4	144	1.030584679	1.117728998	0.087144319	0.23489199	0.421218387
Malt1	89	1.719648444	1.419825476	-0.299822969	0.23509655	0.421530641
Hirip3	9	0.451009895	1.198581678	0.747571783	0.2352435	0.42174259
Tk1	6	1.577129497	2.242370847	0.66524135	0.23549105	0.42193089
Cpsf2	61	1.342385845	1.553789186	0.21140334	0.23539215	0.42193089

Zzz3	169	1.116320861	0.942810144	-0.173510717	0.23546938	0.42193089
Kat6a	103	0.888779072	0.819394772	-0.0693843	0.2354137	0.42193089
Ppfibp1	99	1.444390253	1.203766221	-0.240624032	0.2354923	0.42193089
Rpl23	22	1.40989355	1.125037707	-0.284855843	0.23557045	0.421967872
Suz12	98	1.233950826	1.095394842	-0.138555984	0.23555194	0.421967872
Gm15941	2	29.21337676	25.18022972	-4.033147045	0.2356443	0.421997134
Cep85l	43	1.838999089	1.417360331	-0.421638758	0.23564036	0.421997134
Gm12359	48	1.174333241	1.001629954	-0.172703287	0.23575058	0.422135945
Zhx1	43	1.645786035	1.571630199	-0.074155836	0.2358207	0.422209983
Pskh1	11	1.639383351	1.179072332	-0.46031102	0.23586356	0.422235191
Mtfr1	43	1.361373495	1.565786288	0.204412793	0.23609174	0.422415658
Snw1	6	0.773206122	0.142450142	-0.63075598	0.23607643	0.422415658
Gpaa1	27	8.388538976	7.888306936	-0.50023204	0.23605607	0.422415658
)30402H24F	64	0.894673705	1.044558107	0.149884403	0.2360521	0.422415658
Hnf1a	1	28.75	20.73170732	-8.018292683	0.2361083	0.422415658
130011E15F	61	1.041141422	1.193736883	0.152595461	0.23617264	0.422479253
Tmem39b	41	1.54703783	1.685100794	0.138062964	0.23628003	0.422568336
Ppp1r13l	14	1.732881215	1.615305886	-0.117575329	0.2362792	0.422568336
Kbtbd11	72	5.00767783	5.140384425	0.132706595	0.23648064	0.422875574
Bet1l	25	1.336271969	1.185429048	-0.150842921	0.2367483	0.423052115
Smc4	28	1.636198013	1.066871199	-0.569326814	0.23663705	0.423052115
Klhl24	52	1.243691183	1.027925443	-0.21576574	0.23675235	0.423052115
Zfand3	112	1.351729228	1.069870175	-0.281859053	0.23664672	0.423052115
Scrn3	5	1.033313852	1.237404442	0.204090591	0.23668096	0.423052115
Gosr1	9	3.718073815	2.623033057	-1.095040757	0.23671884	0.423052115
Tpm1	79	2.477941047	2.454044626	-0.023896421	0.23706031	0.423550828
Rpl28	30	1.267028095	1.232818978	-0.034209117	0.23716552	0.423687217
Ccdc59	54	1.244080448	1.280940243	0.036859795	0.23720904	0.423713376
Rtf1	75	1.478248149	1.731004375	0.252756226	0.23726989	0.423770499
Ackr3	3	12.15085708	9.209419208	-2.941437876	0.23748315	0.424099764
Kmt2a	103	1.300180067	1.441083317	0.140903249	0.23763027	0.424310848
Xrn1	33	1.593233655	0.932212579	-0.661021076	0.23772555	0.424429342
Suv420h1	43	1.321345103	1.127510317	-0.193834786	0.23780226	0.424514645
Cdipt	7	3.632543511	3.571538218	-0.061005292	0.23783584	0.424522945

Slc7a5	40	1.105264953	0.67658749	-0.428677463	0.23800763	0.42477791
Nras	32	1.387771422	1.037629629	-0.350141793	0.23808619	0.424866449
Eva1c	72	1.654074978	1.473698272	-0.180376706	0.23813002	0.424892981
Ints8	49	1.254041887	1.030126819	-0.223915068	0.23827482	0.425075663
Abhd12	51	1.248012222	1.209615142	-0.03839708	0.23829034	0.425075663
Zfp964	7	1.924807863	1.149108292	-0.775699571	0.23859979	0.425575955
Cdk13	102	1.155253302	0.853710918	-0.301542385	0.23879536	0.425736006
Hapln2	1	89.3129771	93.57798165	4.265004552	0.2388636	0.425736006
Eif4ebp3	14	79.7873016	79.03956166	-0.747739942	0.23880513	0.425736006
430093F15F	2	46.14290495	40.64825695	-5.494648001	0.23881058	0.425736006
Bcat2	15	2.417383105	1.86231295	-0.555070154	0.23880948	0.425736006
Cecr5	44	1.29248874	1.461963402	0.169474662	0.23884454	0.425736006
Ubl3	76	1.374041028	1.1211504	-0.252890628	0.23910927	0.426122101
Ptges2	33	1.702712023	1.562672908	-0.140039115	0.23923578	0.426295782
Srgap1	82	2.015969236	2.021459506	0.005490269	0.2395787	0.426803178
Ubr1	31	1.260724492	1.645280716	0.384556224	0.23955639	0.426803178
H2-M10.3	1	69.3877551	75.51020408	6.12244898	0.23968209	0.426935547
Ncstn	19	1.585914194	1.204181359	-0.381732835	0.23981797	0.427125733
Ddt	24	1.733462013	1.841099621	0.107637608	0.23999366	0.427386762
Tusc1	126	1.730575782	1.829708174	0.099132392	0.24018085	0.427668221
Dtl	13	1.677431597	1.28964423	-0.387787367	0.24026137	0.42775968
Them4	21	0.882244136	1.078444161	0.196200026	0.24031076	0.427795715
Ism1	54	1.44505181	1.139270176	-0.305781635	0.24044535	0.427935702
Maz	111	1.320844184	1.287412883	-0.0334313	0.24044772	0.427935702
Fam135a	87	1.425327747	1.2442334	-0.181094346	0.24056725	0.428096519
Camkk2	92	2.03679304	2.144781373	0.107988333	0.24068263	0.428249892
Clock	245	1.35545373	1.457841199	0.102387469	0.24072946	0.428281297
Lemd3	114	1.146042798	1.183124068	0.03708127	0.24085014	0.428444056
Lrrc14b	7	43.57643942	41.46345145	-2.112987963	0.24109682	0.428830871
Tardbp	37	1.239909909	1.005070216	-0.234839693	0.24130299	0.428989594
510007P14F	2	2.734375	3.416646146	0.682271146	0.24123717	0.428989594
Tes	34	1.302574334	1.077133583	-0.225440751	0.24128456	0.428989594
Ormdl3	40	1.48767662	1.274400425	-0.213276195	0.24126533	0.428989594
Tnc	1	22.72727273	16.27906977	-6.44820296	0.24141777	0.429141653

Zfp619	26	1.113951307	1.551187381	0.437236074	0.2414759	0.429192994
Skil	202	1.401371233	1.221055125	-0.180316108	0.24167251	0.429490425
Fam96b	41	1.560037028	1.405251648	-0.15478538	0.24221946	0.430341838
Agtrap	43	1.963062804	1.602128931	-0.360933873	0.24219338	0.430341838
Pcnp	46	0.860316315	0.902541537	0.042225221	0.24223958	0.430341838
Dazap2	44	1.332267897	1.444588245	0.112320347	0.24237411	0.430388103
330188P03R	4	45.59067677	41.55234104	-4.038335728	0.24238294	0.430388103
10047M10I	89	1.360440466	1.293454595	-0.066985871	0.242359	0.430388103
N4bp3	22	3.369272766	2.705368922	-0.663903844	0.24235837	0.430388103
Jun	44	1.470986246	1.158703382	-0.312282864	0.24245344	0.430461194
Paox	22	1.48070405	1.650091781	0.169387732	0.24254973	0.430580051
Bop1	55	1.594303359	1.37507087	-0.219232488	0.24265976	0.430723276
Alg12	67	1.371325208	1.554712202	0.183386994	0.24270112	0.430744594
Actn4	57	1.220715138	1.014672008	-0.20604313	0.24311704	0.431302474
Atp9b	40	1.03943279	0.678072105	-0.361360685	0.24314439	0.431302474
Dgkq	19	2.0001952	1.309061572	-0.691133628	0.24315695	0.431302474
Arhgef16	69	2.225202094	1.909621883	-0.315580211	0.24305377	0.431302474
Ubxn2a	112	1.248231003	1.093706203	-0.1545248	0.24316242	0.431302474
Nr5a2	3	12.3812193	17.40767905	5.026459746	0.24331055	0.431495842
Snx15	26	1.214935176	1.028115997	-0.186819179	0.24333025	0.431495842
Wwp2	43	1.33019998	1.001109069	-0.32909091	0.2434094	0.431584041
Ppap2a	49	1.253161206	1.090665021	-0.162496184	0.24343996	0.431586072
730008H23R	23	0.548530361	0.672976877	0.124446516	0.24354393	0.43171825
Dcaf10	98	1.176767249	1.246022027	0.069254778	0.24376309	0.432054543
Irf2bp1	31	1.243373748	1.651713805	0.408340056	0.24392655	0.432245563
Tpst2	7	1.306260163	1.750475822	0.444215659	0.24395923	0.432245563
Kctd13	60	1.287083286	0.957198748	-0.329884539	0.24393011	0.432245563
Hip1r	73	1.303280849	1.234285105	-0.068995743	0.24423747	0.432686293
Atmin	54	1.266388429	0.970579786	-0.295808643	0.24431043	0.432763302
Gpkow	14	14.39970008	13.62517125	-0.774528835	0.24434718	0.432776159
900060B14R	10	0.193705736	0.75602702	0.562321284	0.24449708	0.432884903
Zfat	46	1.091344886	1.224009811	0.132664925	0.2444869	0.432884903
Brd3	156	1.235195027	1.101607466	-0.133587562	0.24446258	0.432884903
330069E16R	3	3.065279578	2.85455599	-0.210723588	0.24455592	0.432936858

Prkci	54	0.978878504	0.853847977	-0.125030527	0.24463324	0.433021483
Ipo8	10	3.645980258	2.773429657	-0.872550602	0.24476253	0.433198091
Ccl17	2	94.61028192	91.98915446	-2.621127461	0.24490415	0.433396469
Pcx	47	1.340248686	1.244700088	-0.095548597	0.24507269	0.433642424
Efcab10	3	35.73333333	31.65374677	-4.079586563	0.24522306	0.433834422
Gna11	75	0.981654928	0.952082625	-0.029572302	0.24526989	0.433834422
Nck1	101	1.408691951	1.282667673	-0.126024278	0.24526677	0.433834422
Snx18	57	0.992825127	0.84331496	-0.149510167	0.24541413	0.433984921
Ucp3	6	97.52408815	96.19310861	-1.330979538	0.24540841	0.433984921
Aifm2	11	17.66875417	17.2082538	-0.460500369	0.24545446	0.434003952
Gm6787	9	23.24051569	21.58498232	-1.655533364	0.24556231	0.434128987
Ahnak	52	2.696026937	2.235121853	-0.460905084	0.24558435	0.434128987
Mrpl33	23	0.909546378	1.055767115	0.146220737	0.24572012	0.434264371
Gpr137b	11	1.291806829	1.404169109	0.112362281	0.24571832	0.434264371
Rps15a	7	1.49946181	1.072537238	-0.426924572	0.24586788	0.434473173
Extl1	10	6.688283774	6.314224811	-0.374058963	0.24607074	0.434779297
Rplp2-ps1	9	92.66299946	90.4905811	-2.172418356	0.24620606	0.434913646
Bub3	40	1.565862816	1.532358148	-0.033504668	0.24618727	0.434913646
Rpl13	77	1.254196831	1.360410373	0.106213542	0.24631446	0.435052763
Enpp4	50	1.110345301	0.817598043	-0.292747258	0.24640786	0.435165349
Man2a2	87	1.241438426	1.076164174	-0.165274252	0.24651787	0.435307242
Pts	31	0.938073054	1.251472507	0.313399453	0.24659933	0.435398695
Ephb6	58	1.525753833	1.398705092	-0.127048741	0.24665662	0.43544745
Yes1	99	1.19269129	1.193146235	0.000454946	0.24669813	0.435468343
Irgq	5	2.61253581	2.470711719	-0.141824091	0.2467551	0.435516513
Dxo	48	1.040027021	1.25264295	0.212615929	0.2467963	0.435536857
Leprotl1	93	1.363064632	1.341890559	-0.021174074	0.24700053	0.435844851
Ago1	24	1.685195722	1.80332321	0.118127488	0.24737636	0.436455532
Tmem184c	10	0.652020202	1.558728221	0.906708019	0.24743297	0.436502935
Zfp235	8	0.88028169	1.553799296	0.673517606	0.24754845	0.436654152
Tfcp2l1	36	2.19120958	2.067373117	-0.123836463	0.24758122	0.436659474
Fam3c	72	0.943795859	0.776281665	-0.167514195	0.24766115	0.436747949
Pnpla2	1	36.95652174	47.82608696	10.86956522	0.24786236	0.437050257
Rabl2	21	1.728864738	1.294901425	-0.433963313	0.24810379	0.437423414

Lta4h	24	0.883929618	0.898898983	0.014969365	0.24829231	0.437703186
Ring1	64	1.498522732	1.253377722	-0.245145009	0.24836107	0.437745736
Ncoa3	89	0.774810725	1.122811567	0.348000842	0.24837611	0.437745736
Clcn6	32	1.979466333	1.867820647	-0.111645686	0.24854953	0.437998781
Mis18a	10	1.102718528	0.714514013	-0.388204514	0.24858112	0.438001851
Stag1	120	1.203360788	1.142427872	-0.060932916	0.24868698	0.438135768
Dync1i2	33	1.349137105	1.200282308	-0.148854797	0.2487998	0.438281906
Rab33b	65	0.946949679	1.098515997	0.151566318	0.2488855	0.438380254
Gbe1	74	1.17579508	1.159189114	-0.016605966	0.24917579	0.438786217
Map2k7	23	3.444578646	2.718066131	-0.726512516	0.24914958	0.438786217
Gm4980	35	11.24789868	11.03309366	-0.214805015	0.24939117	0.439080551
Ndufb2	29	1.273898622	1.346552643	0.072654021	0.24940278	0.439080551
Sbf2	126	1.453957896	1.193186501	-0.260771395	0.24947895	0.439161961
Stk35	36	2.26376237	2.007928906	-0.255833464	0.24964735	0.439384399
Nucb2	33	1.182711237	1.06921525	-0.113495987	0.2496652	0.439384399
Tecpr2	36	1.195879453	1.426452226	0.230572773	0.24969758	0.439388686
Vps16	8	0.80625855	0.525771726	-0.280486824	0.24989259	0.439679116
Ubac2	53	1.772432841	1.499027445	-0.273405396	0.24992604	0.439685251
)30048N14F	65	0.903868128	1.168518728	0.2646506	0.25011288	0.439942382
Alkbh1	24	0.946403233	1.059364494	0.112961261	0.25013216	0.439942382
Zfp622	62	1.396978588	1.059738298	-0.33724029	0.25020103	0.440010773
Il17rc	7	58.74795276	55.18976655	-3.558186215	0.25051536	0.440457992
Ugdh	46	1.316002379	1.04843919	-0.267563189	0.25050653	0.440457992
Tbc1d22b	48	2.051134046	2.214230426	0.16309638	0.25066373	0.440666064
Eif1a	16	1.991239351	2.21610051	0.22486116	0.25088581	0.441003644
Mir688	6	4.425521628	2.980318589	-1.44520304	0.25098727	0.441107403
510016A17F	13	38.63188297	41.42804773	2.796164765	0.25100496	0.441107403
Tom1l2	38	1.607065556	1.335589428	-0.271476128	0.25120915	0.441413363
Mir7037	4	96.72918565	97.54492474	0.815739091	0.25142898	0.441468579
310454H06F	13	1.205458405	1.228818589	0.023360183	0.25145116	0.441468579
Zdhhc18	42	1.443163128	0.996222875	-0.446940254	0.25138871	0.441468579
Abcf2	69	1.3698704	1.448899853	0.079029453	0.25133341	0.441468579
Tmem17	44	1.221966629	1.177843043	-0.044123587	0.25144122	0.441468579
Trpm2	11	12.55764163	14.33358014	1.775938505	0.25127651	0.441468579

Cav1	5	3.071034683	4.594905493	1.52387081	0.25135736	0.441468579
Whamm	66	0.945812099	1.042633827	0.096821729	0.25149135	0.441486303
ʼ10014B01F	2	3.574424564	3.037164508	-0.537260056	0.25155323	0.441489312
Znrf3	105	1.093393225	1.227557104	0.134163879	0.25152436	0.441489312
Relb	66	1.473052199	1.47078473	-0.002267469	0.25171644	0.441722917
Atrip	28	1.322147413	1.299715181	-0.022432232	0.25198233	0.44213663
'00008J07R	36	1.427103208	1.248563152	-0.178540056	0.2521148	0.442149301
Cntnap5a	6	58.63108796	56.42615775	-2.204930214	0.25220047	0.442149301
Dpp7	6	3.655566376	2.440719062	-1.214847313	0.25210907	0.442149301
Tmem60	34	1.033109885	0.872976511	-0.160133374	0.25219779	0.442149301
Dyrk1a	228	0.934277627	0.992983672	0.058706045	0.25204863	0.442149301
Psmb4	39	1.403449184	1.520467054	0.117017869	0.2521495	0.442149301
Crispld1	3	6.355218855	4.167997445	-2.18722141	0.25212575	0.442149301
Thop1	27	0.821265424	0.747666934	-0.07359849	0.25234559	0.442246164
Fem1c	57	1.790813831	1.412311986	-0.378501845	0.25230365	0.442246164
Fam43a	58	1.069079286	1.171339437	0.102260151	0.25234613	0.442246164
Rpl34-ps1	41	0.997409599	1.035807882	0.038398283	0.25252133	0.442447527
Psma2	12	0.999411322	1.522034187	0.522622866	0.25249357	0.442447527
Rhbdf1	94	0.989095629	0.996344617	0.007248987	0.25273296	0.442765448
Brf2	20	0.964816515	1.2630008	0.298184286	0.25280131	0.442832338
Camk2g	75	1.62548712	1.502492456	-0.122994665	0.25295488	0.442995572
Phactr4	33	1.435165155	1.532512798	0.097347643	0.25295224	0.442995572
Znhit2	90	1.232432713	1.109334909	-0.123097804	0.25320912	0.443334996
Paqr8	43	1.813747749	1.482103474	-0.331644275	0.25318355	0.443334996
Cpt1b	27	86.52110366	85.37590278	-1.145200879	0.25332145	0.443478757
Casp8	25	1.464391696	1.81474626	0.350354563	0.25343192	0.443619234
Cab39l	30	1.154355538	1.270815744	0.116460206	0.25377223	0.444161937
Wtap	123	1.238780736	1.038530893	-0.200249843	0.25391168	0.444353012
)30513N10F	60	1.279590075	1.202725114	-0.076864961	0.25409203	0.444615597
Wdr53	27	1.379611858	0.965759933	-0.413851925	0.25430467	0.444934633
Mettl1	53	2.405225567	2.207306859	-0.197918708	0.25471839	0.44553642
Cmtm2b	1	96.71361502	94.63087248	-2.08274254	0.25473971	0.44553642
Slc35b1	12	0.806788895	1.374749161	0.567960265	0.25468762	0.44553642
Ano4	3	15.73518994	14.01583105	-1.719358885	0.25479252	0.445575672

Tmed7	60	1.052313838	1.098375557	0.046061719	0.25491256	0.445732478
Irf2bpl	74	1.166913948	1.031366167	-0.13554778	0.25495246	0.445749121
Dcbld1	48	2.072821278	1.777099737	-0.295721541	0.25511636	0.445982546
Luzp1	44	1.37012567	1.521385765	0.151260095	0.25519788	0.446071908
Rhot1	59	1.005972854	0.761289927	-0.244682927	0.25544447	0.446449754
Klhl17	26	1.033840168	0.802857354	-0.230982815	0.25548762	0.446472002
Esyt3	43	15.79723534	16.63588427	0.838648922	0.25565574	0.446712591
Asnsd1	26	0.775623983	0.913385068	0.137761085	0.2558192	0.446838583
Nae1	34	1.120258985	0.838466995	-0.28179199	0.25578132	0.446838583
Kdm4a	9	2.435543261	1.981409723	-0.454133538	0.25579604	0.446838583
Sike1	42	0.933506791	0.885585852	-0.047920939	0.2559922	0.447087548
Rfwd3	14	1.15075844	0.483165722	-0.667592718	0.25605137	0.447137669
Tmub2	18	1.211927033	0.517828761	-0.694098272	0.25617832	0.447306127
Ndufb4	21	1.200497311	1.040203494	-0.160293817	0.25641772	0.447601745
Emc6	15	1.450704651	0.98120906	-0.469495591	0.25643914	0.447601745
Dnajc2	58	1.278899664	0.971557541	-0.307342124	0.25643402	0.447601745
Tmcc3	91	1.882401519	1.977434961	0.095033442	0.25648858	0.447634799
Gm2115	35	3.97927013	4.159821948	0.180551818	0.2566483	0.447807028
Ubap2l	22	1.124277243	1.554117647	0.429840405	0.25664232	0.447807028
Itga2	14	1.764838051	1.378306073	-0.386531978	0.25674583	0.447923954
Aars2	22	1.526594846	1.486103785	-0.040491061	0.25693904	0.448160976
Ubxn10	15	21.42229566	20.29905567	-1.123239992	0.25694278	0.448160976
Rabepk	11	1.913579924	1.474573419	-0.439006506	0.25699071	0.448191303
Crtc3	113	1.056618843	1.252235131	0.195616288	0.25706114	0.448260869
Nrep	30	1.445264214	1.188036831	-0.257227384	0.25710717	0.448287854
Chek1	23	1.625981867	1.392918992	-0.233062875	0.25718932	0.448377812
Insc	10	44.71589713	45.9495412	1.233644075	0.2573489	0.448602721
10062M04I	28	1.230847924	1.314611108	0.083763184	0.25741231	0.44865997
Zbtb22	20	1.291223471	1.216594422	-0.074629049	0.25764045	0.448897653
Habp4	44	1.169620909	1.206821566	0.037200657	0.25762697	0.448897653
Plekhh3	25	2.075543475	1.900790663	-0.174752812	0.25759332	0.448897653
Sema4g	42	7.230009396	7.088399759	-0.141609637	0.2577566	0.44900452
Bbs12	20	1.112013125	0.943451467	-0.168561658	0.25777291	0.44900452
Sorbs2os	8	90.81728127	92.88788115	2.070599881	0.25785429	0.44900452

Snora26	49	1.555370167	1.870912565	0.315542398	0.25785478	0.44900452
Mau2	24	1.510112257	1.349610377	-0.16050188	0.25780286	0.44900452
H2-T3	2	68.79830399	65.38461538	-3.413688609	0.25789079	0.449013944
Cul7	31	6.207185189	6.116947064	-0.090238125	0.25826109	0.449605318
Zfhx2	24	2.649344617	2.01745177	-0.631892847	0.25848428	0.449940496
Psme3	46	2.031697478	2.012606687	-0.019090791	0.25864599	0.450168574
Tns1	70	2.69896656	3.147934697	0.448968137	0.25868654	0.450185753
Ulk1	81	1.073755084	1.093462983	0.019707899	0.25876362	0.450266492
Ppm1k	7	1.84151565	1.391015607	-0.450500043	0.25889551	0.450442577
Laptm4b	37	1.556899107	1.176970283	-0.379928824	0.25899433	0.450561093
Gm14403	2	17.62867647	15.07983714	-2.548839328	0.2590451	0.450595993
Clec16a	43	0.997350821	1.143439976	0.146089155	0.25914233	0.450711696
Gpr155	28	1.826404547	1.411273594	-0.415130953	0.25931676	0.450961617
Nek3	1	7.272727273	14	6.727272727	0.25936766	0.450996685
Adgrl1	137	1.496516136	1.382965977	-0.113550159	0.25942684	0.451046139
Adnp2	22	2.175140441	1.750689404	-0.424451036	0.25984698	0.451669575
Sephs2	38	1.274888885	1.325197612	0.050308727	0.2598194	0.451669575
Ppapdc2	48	1.223421038	1.253845298	0.03042426	0.25989339	0.451696738
Ier3	28	1.38966492	1.017099972	-0.372564949	0.26030247	0.45235415
Polr2k	5	2.582465899	1.098034767	-1.484431133	0.26033781	0.452361989
Itga6	79	1.02484216	1.283546633	0.258704473	0.26059062	0.452747663
Ankrd11	141	0.966040969	0.938633126	-0.027407843	0.26068966	0.452866121
Npm1	14	1.628848074	1.53130744	-0.097540634	0.26085414	0.453098202
Arf5	64	1.280233755	1.301260119	0.021026363	0.26091272	0.453122718
Manf	57	1.038118746	1.235218966	0.19710022	0.26093001	0.453122718
Dlgap4	141	3.335484727	4.106615309	0.771130582	0.26101719	0.453194261
Rnaseh1	12	1.310265145	1.255398638	-0.054866507	0.26103298	0.453194261
Commd7	31	1.179614321	0.880209836	-0.299404485	0.26149898	0.4539496
Gnas	217	18.55101155	18.23509822	-0.31591333	0.26159982	0.454070951
Tnfrsf12a	30	1.681793418	1.171305452	-0.510487967	0.26170747	0.454204075
Rrp15	9	1.919616774	1.618668259	-0.300948515	0.26197401	0.454612885
Prr14	27	2.91665977	2.587883952	-0.328775818	0.26214612	0.454857767
00123M08I	8	2.469030427	2.079212255	-0.389818172	0.26240569	0.455148383
Cyb5r4	32	1.218753118	1.079842259	-0.138910858	0.26235137	0.455148383

Zfp367	101	0.944731605	1.057121307	0.112389703	0.26240666	0.455148383
Dhx8	4	1.274839275	0.520833333	-0.754005942	0.26244539	0.455161767
Thbs3	15	2.368510162	1.365730184	-1.002779979	0.26253263	0.455259262
Zfp653	45	0.935567033	1.100052797	0.164485764	0.26257355	0.455276416
Phb2	18	1.288248254	1.794572902	0.506324647	0.26270255	0.455446271
Farsa	18	1.274756629	0.893866972	-0.380889656	0.26299576	0.45590074
Dcaf5	60	0.897216018	1.116974715	0.219758697	0.26308547	0.456002379
Tomm5	9	2.19275618	1.331868461	-0.860887718	0.26333733	0.456385023
Sf3b3	10	1.381838887	1.120799401	-0.261039486	0.26345795	0.456540141
Cenpw	22	1.08766367	1.395483866	0.307820196	0.26352122	0.456595863
Pars2	10	1.393400538	1.588163305	0.194762767	0.26369589	0.456844576
Taok3	31	12.68435372	13.31044961	0.626095896	0.26373634	0.45686071
930511A02R	16	56.38178762	56.73668107	0.354893446	0.26378544	0.456864727
Mtrr	34	1.011467092	1.694700102	0.68323301	0.26380093	0.456864727
Slc25a33	87	1.190368965	1.144143283	-0.046225682	0.26402308	0.457195492
Armc7	20	1.639401367	1.132016605	-0.507384761	0.26426512	0.457560628
00003M02I	20	4.609804436	5.73966968	1.129865244	0.26441766	0.457770743
Ccdc23	63	1.282342044	1.58826628	0.305924236	0.264494	0.457848879
Bcl2	73	1.651596913	1.687794498	0.036197584	0.264624	0.458019887
Mtmr4	31	1.300934995	0.988779019	-0.312155976	0.26483349	0.458328421
Dennd4a	31	1.753156857	1.148522386	-0.604634471	0.26509213	0.458721933
Rpl36	34	1.552611997	1.25822752	-0.294384477	0.26512755	0.458729123
Hist2h2ab	7	3.88966658	3.187881083	-0.701785497	0.26519025	0.458754804
Nxnl2	24	12.80019246	15.07294924	2.272756785	0.26520492	0.458754804
Ahcyl1	43	0.995217729	0.866516314	-0.128701414	0.26544698	0.459119406
Rps18	45	1.099140063	1.007194216	-0.091945847	0.26556589	0.459204865
Coro1a	28	15.73413885	16.58986216	0.855723306	0.26562157	0.459204865
Exoc1	11	2.628046164	3.310014602	0.681968438	0.26561834	0.459204865
Bag6	44	1.058946568	1.265686622	0.206740054	0.26554278	0.459204865
Os9	3	1.815439462	2.524665415	0.709225952	0.26565312	0.459205309
Arpc5	131	1.300259084	1.138476114	-0.16178297	0.26573002	0.459284128
Exoc5	7	0.998695172	0.474433316	-0.524261856	0.26583708	0.459343942
Hnrnpr	71	1.174536621	1.246953181	0.07241656	0.26585853	0.459343942
Mrm1	20	1.234342581	1.022771595	-0.211570987	0.26580719	0.459343942

AI837181	28	1.833226054	1.303620368	-0.529605686	0.26598642	0.459456708
Cyp39a1	12	1.637840136	1.348350021	-0.289490115	0.26598552	0.459456708
Srgap2	66	1.55113922	1.342533238	-0.208605982	0.26607336	0.459552784
Zmym2	38	1.535321868	1.286260958	-0.24906091	0.26622328	0.459703505
Lace1	31	1.101047474	1.348968343	0.247920869	0.26621534	0.459703505
Uggt1	51	1.648045163	1.358013504	-0.29003166	0.26632714	0.459774635
Pqlc1	17	9.114424525	10.10432866	0.989904136	0.26629695	0.459774635
Dok5	18	52.58056658	54.82196073	2.241394152	0.26658992	0.460174152
Rab24	32	0.992143454	1.169653675	0.177510221	0.26674797	0.460261624
Mycbp2	19	1.980078428	1.601950594	-0.378127834	0.26677061	0.460261624
[illegible]10002D24[illegible]	17	1.006926394	0.714738404	-0.29218799	0.26669158	0.460261624
Dok4	49	1.48284723	1.088691666	-0.394155564	0.26679742	0.460261624
Ugcg	109	1.119391976	1.32581645	0.206424474	0.26670543	0.460261624
Aldh3a2	19	1.740059097	1.14127536	-0.598783738	0.26683882	0.460278926
Tpp2	37	1.677580333	1.167949783	-0.50963055	0.26708332	0.460592397
Zfp120	2	2.595211313	5.470162749	2.874951436	0.26705737	0.460592397
Cul4b	2	91.48606811	93.21309576	1.727027644	0.26719163	0.460725034
Chid1	26	1.140156636	0.705986559	-0.434170077	0.26723305	0.460742305
Rbmxl1	64	0.923612905	0.882311762	-0.041301143	0.2672878	0.460782561
[illegible]30116N13[illegible]	53	1.170713477	1.116709521	-0.054003956	0.26732317	0.460789398
Arsg	61	6.298620952	5.292454106	-1.006166846	0.2674255	0.460911648
Esf1	2	2.5	0.740740741	-1.759259259	0.26749839	0.460983132
Uevld	15	2.283120702	2.585298978	0.302178276	0.26754366	0.461007012
Pole4	44	1.189025383	1.091683784	-0.097341599	0.26763685	0.461113436
Itprip	36	1.090059795	1.199666476	0.109606681	0.2679325	0.46151444
Prkaca	83	1.293717661	1.147364543	-0.146353118	0.26792079	0.46151444
Kif23	46	1.37991773	1.390007552	0.010089822	0.26803606	0.461638633
Mplkip	57	2.7109498	3.630098814	0.919149014	0.26826665	0.461948771
Med31	1	2.659574468	4.812834225	2.153259757	0.26827909	0.461948771
Zwilch	18	0.964362608	1.601819783	0.637457175	0.26831751	0.461960711
Lpar2	15	4.137492891	3.381649847	-0.755843045	0.26844198	0.462084388
Epb4.1l4a	53	2.072646973	1.701101202	-0.371545771	0.26845232	0.462084388
Tasp1	32	0.73041879	0.980925585	0.250506795	0.26855191	0.462201595
Tulp1	3	83.64613926	79.38974754	-4.256391716	0.26881281	0.462596368

Tnfrsf25	28	37.91286394	39.09516365	1.182299714	0.26891634	0.462720253
Acyp1	11	0.761687654	0.225861095	-0.535826558	0.2690374	0.462874294
Nsun5	17	1.586101639	1.639852547	0.053750908	0.26930906	0.463233055
Ube2h	41	0.946831893	1.100913462	0.154081569	0.26930247	0.463233055
Grpel2	24	0.921500672	0.912803324	-0.008697348	0.269371	0.46328528
Etnk1	101	2.098369725	1.459092485	-0.63927724	0.26996395	0.464250682
Suco	59	1.658380296	1.705787185	0.047406888	0.27007781	0.464392058
Ptrh2	14	2.307207305	2.573343773	0.266136468	0.2702414	0.464618892
Ate1	69	3.347172883	2.416569029	-0.930603855	0.27036963	0.464784903
Crtap	14	1.325807334	1.72310483	0.397297496	0.27041176	0.46480288
Tmem251	16	1.926835948	1.605591698	-0.32124425	0.27052253	0.464829937
Rpap2	16	1.184783961	0.747501738	-0.437282223	0.2705136	0.464829937
Rundc1	24	1.027419481	1.184293418	0.156873937	0.27047768	0.464829937
Lca5l	33	1.769001826	1.269790786	-0.49921104	0.27058335	0.464830331
Slit3	36	47.51327043	47.55932518	0.046054751	0.27058612	0.464830331
230035I16R	4	3.19029251	1.339285714	-1.851006796	0.27087475	0.465271696
Mir1932	78	1.747016187	1.414756133	-0.332260054	0.27093342	0.465317993
Col4a5	3	18.29521853	21.35416667	3.05894814	0.27099553	0.465370209
Ctif	17	1.644249678	1.394424451	-0.249825227	0.27110213	0.46549879
Vmp1	34	1.584052002	1.727595582	0.14354358	0.27122284	0.465651568
Spen	142	1.372521963	1.224180542	-0.148341421	0.27144477	0.465978063
Vcpip1	37	1.486309477	0.951827855	-0.534481622	0.27172775	0.466354738
Cobll1	167	1.378886211	1.40449778	0.02561157	0.27169924	0.466354738
Rpl29	16	2.148409763	1.581016758	-0.567393004	0.27190601	0.466561455
Mir5619	31	1.102605496	0.877531111	-0.225074386	0.27191179	0.466561455
Proser3	2	5.163043478	2.923976608	-2.23906687	0.27205823	0.46664903
Gm38670	2	5.163043478	2.923976608	-2.23906687	0.27205823	0.46664903
Arhgef28	52	1.58710509	1.476431742	-0.110673348	0.27205455	0.46664903
930593A02R	27	1.917616452	2.26069304	0.343076587	0.27227305	0.466962923
Ddx50	40	1.297356032	1.241439729	-0.055916303	0.27232562	0.466998496
Smc1b	10	73.0330128	71.07229386	-1.960718938	0.27240287	0.467076391
Gatad2a	171	1.439532126	1.2554217	-0.184110425	0.2724508	0.467083176
Gins4	34	1.098503233	1.296690453	0.198187219	0.27247049	0.467083176
Kdm2a	132	1.792050632	1.7249427	-0.067107932	0.27259956	0.467195269

Gm4944	6	0.839237853	1.533465906	0.694228054	0.27258763	0.467195269
Zfp786	22	1.386448469	1.299038463	-0.087410007	0.27268997	0.467295647
Anxa3	4	4.313832504	3.29809667	-1.015735834	0.2727776	0.467391235
Adgre5	40	18.40829048	19.20806188	0.7997714	0.27294884	0.467575443
10-Mar	12	21.50535902	23.81727232	2.3119133	0.2729334	0.467575443
D11Wsu47e	23	1.242717816	0.840518017	-0.402199798	0.2733484	0.467718357
Tmem38a	23	0.906398594	1.023355304	0.11695671	0.27320564	0.467718357
Dpf2	23	1.737988542	1.25809489	-0.479893653	0.27331484	0.467718357
Kcnd1	5	88.22571291	80.00565897	-8.220053933	0.27332406	0.467718357
Msto1	25	1.930810861	1.408669415	-0.522141446	0.27332939	0.467718357
Clk2	44	1.469827483	1.517855072	0.048027589	0.27338288	0.467718357
Clk2-scamp3	44	1.469827483	1.517855072	0.048027589	0.27338288	0.467718357
Tapbp	12	35.6389468	34.678241	-0.960705805	0.27327125	0.467718357
Acot8	27	1.042639531	1.135363856	0.092724325	0.27316703	0.467718357
Flot1	25	1.919512476	2.033828601	0.114316125	0.27324721	0.467718357
Pim1	141	1.180605685	1.043568317	-0.137037367	0.27320454	0.467718357
Mrps14	15	1.307105361	1.261053051	-0.04605231	0.27354173	0.467935566
Nudt21	14	0.842501471	0.830989626	-0.011511845	0.27365154	0.468068856
Vars2	38	1.704713101	1.373263059	-0.331450042	0.27378557	0.468243531
Slfn8	1	25.27472527	19.04761905	-6.227106227	0.27383051	0.468265802
Tekt4	7	33.68630447	34.03856129	0.352256821	0.27392585	0.468374256
Dcaf11	31	1.212338228	1.308853844	0.096515616	0.27398992	0.468429224
Actb	60	1.704286047	1.681847307	-0.02243874	0.27418979	0.46862889
Manbal	43	1.895426527	1.798878739	-0.096547788	0.27418083	0.46862889
Mars	49	1.621159986	1.409573443	-0.211586543	0.27420251	0.46862889
Kif13a	77	1.415929695	1.210251477	-0.205678218	0.27431618	0.468713968
Trappc13	16	1.44979158	1.045122305	-0.404669274	0.27430897	0.468713968
Dlat	54	1.488389129	1.073031062	-0.415358067	0.2746049	0.469098054
Dph7	4	3.741758242	3.110748454	-0.631009787	0.27458471	0.469098054
Sec14l5	8	4.54651745	3.25631938	-1.29019807	0.27468442	0.469179283
Eif4ebp1	58	2.237482554	1.659866413	-0.577616141	0.27472442	0.469192993
Tmprss13	4	11.72043011	10.37000366	-1.350426443	0.27486559	0.469215636
Adap1	109	1.22343868	1.04959909	-0.17383959	0.27479229	0.469215636
Eef2kmt	21	1.207159533	1.478851203	0.27169167	0.27482527	0.469215636

Zfp787	64	1.470157914	1.201217312	-0.268940602	0.27484536	0.469215636
Xpr1	39	1.563465396	1.216783895	-0.3466815	0.27511063	0.469529667
Adamts18	29	24.19972178	26.73605638	2.536334599	0.27511354	0.469529667
430571L13F	20	2.330560536	1.979318368	-0.351242168	0.27520562	0.469577593
Deptor	61	1.269141923	1.377720806	0.108578883	0.27518883	0.469577593
Tyw5	27	1.314715827	1.05824302	-0.256472807	0.27542689	0.469884464
Tia1	11	1.268884891	1.265685884	-0.003199007	0.27544951	0.469884464
Ccdc9	26	2.176063917	1.240752371	-0.935311546	0.27555507	0.470009897
Mdm4	29	2.202999038	1.780417212	-0.422581827	0.27567705	0.470018684
Sf3a3	20	1.450822046	1.536227321	0.085405276	0.27563047	0.470018684
Tank	45	1.413187994	1.186364211	-0.226823783	0.27567315	0.470018684
Ddx6	72	1.408475707	1.118098993	-0.290376714	0.27568835	0.470018684
Brdt	2	96.07520993	94.16646933	-1.908740596	0.27580174	0.47013189
Abca2	119	1.041594676	0.956513957	-0.085080719	0.27581882	0.47013189
Trip13	27	1.765096885	1.32087217	-0.444224715	0.27595781	0.470314158
Gbf1	4	2.861895488	3.758938394	0.897042905	0.27627103	0.47047649
Map2k3os	20	1.407287093	1.084508174	-0.322778919	0.27627749	0.47047649
Wdfy2	21	1.56285472	1.669211646	0.106356926	0.27625451	0.47047649
500020E01F	99	0.953272153	1.163871989	0.210599835	0.27611876	0.47047649
Mink1	64	0.906394539	0.974275778	0.067881239	0.27612485	0.47047649
Cry2	22	1.685072859	1.222757741	-0.462315118	0.27618995	0.47047649
Cptp	17	0.794038799	1.127816817	0.333778018	0.27619546	0.47047649
Pitpnm3	84	2.582356211	2.073106506	-0.509249704	0.27657431	0.47087265
Scai	23	1.061064952	0.919087471	-0.141977481	0.27655604	0.47087265
Tnrc18	31	1.164768024	1.13142559	-0.033342433	0.27668208	0.471001482
Lasp1	48	1.266207157	1.012411228	-0.253795929	0.27675579	0.471052332
Socs4	44	1.415875596	1.256273179	-0.159602417	0.27677615	0.471052332
Napb	18	2.040879489	1.833002559	-0.20787693	0.27694935	0.471197175
30028M14I	1	58.97435897	50	-8.974358974	0.27695759	0.471197175
Lxn	14	2.416942517	2.013928	-0.403014517	0.27693117	0.471197175
Ttll5	13	1.252808961	1.091109291	-0.16169967	0.27729703	0.47171998
Gls2	34	1.000581176	1.143554553	0.142973376	0.27739096	0.471770395
Fam126a	49	1.111782356	1.333996132	0.222213775	0.27736181	0.471770395
Dand5	9	1.437073347	0.866030826	-0.57104252	0.27744443	0.471806645

Lmnb1	66	1.172307877	1.006915592	-0.165392286	0.27757205	0.471859623
Areg	22	2.413929685	2.227116629	-0.186813056	0.27753125	0.471859623
Jade2	12	1.28477309	1.416989921	0.132216831	0.27756578	0.471859623
Fbxl2	48	1.21641577	1.011961801	-0.204453969	0.27770599	0.472032633
Ap3d1	35	1.243491742	1.267751567	0.024259825	0.27780348	0.472143644
Zfp811	2	28.62909486	27.1071115	-1.52198336	0.27787479	0.472210147
Ier5	2	1.648351648	1.957232168	0.30888052	0.27795046	0.472235975
Arih2	33	1.695195371	1.323789058	-0.371406313	0.27798653	0.472235975
Rbm12b2	5	1.408450704	2.079207921	0.670757217	0.27798316	0.472235975
.30040H23F	4	8.246228926	10.52631579	2.280086863	0.27802518	0.472246957
Trim59	126	1.238452682	1.200153408	-0.038299274	0.27840632	0.472839629
Atad2	84	1.178887447	1.056101329	-0.122786118	0.27881067	0.473471568
Zfp26	20	1.297669722	1.825220431	0.527550709	0.27895963	0.473660097
Top2b	78	1.12394821	1.098763499	-0.025184711	0.27898625	0.473660097
Myl6	1	2.390438247	4.05904059	1.668602343	0.27905241	0.473717609
Tjp1	85	1.651333641	1.662597094	0.011263453	0.27919189	0.473789956
Ccdc151	12	46.32330775	47.91542402	1.592116277	0.27916544	0.473789956
Atxn2	126	0.952443321	0.842867697	-0.109575624	0.27918073	0.473789956
Napg	17	1.065655709	1.496152104	0.430496395	0.27927792	0.473881149
Mboat1	24	1.8389308	1.759107022	-0.079823777	0.2796215	0.474299601
Oat	51	2.032989869	2.055403148	0.022413278	0.27956486	0.474299601
510037L13F	13	2.53309914	2.00727706	-0.52582208	0.27961725	0.474299601
Cdk18	39	2.38172408	1.865397138	-0.516326942	0.27967014	0.474327287
Mien1	35	1.469299295	1.495747952	0.026448657	0.27971643	0.47435097
Med27	22	2.551699953	2.176215564	-0.375484389	0.27992963	0.474657661
Cers6	38	1.028953056	1.330194282	0.301241226	0.28010718	0.474794132
Paqr3	23	1.747208614	1.678115632	-0.069092982	0.2801048	0.474794132
Zfp607	10	2.105820979	1.879337695	-0.226483285	0.28008078	0.474794132
Serbp1	43	1.357396191	1.153958656	-0.203437535	0.28018944	0.474868583
Pfkfb2	12	1.713723906	2.412929522	0.699205615	0.28021583	0.474868583
Psmd4	17	1.945160086	1.318341197	-0.626818889	0.28036554	0.475067422
Pcnxl2	35	21.28559932	21.40661328	0.121013959	0.28041468	0.475095825
Spata7	13	4.160167283	3.467210877	-0.692956406	0.28065795	0.475453099
Xpnpep3	39	1.432918983	1.403628572	-0.02929041	0.28073024	0.475520668

Serpine1	7	2.368530148	1.737218018	-0.63131213	0.28079728	0.47555099
Tmem243	77	1.085671669	1.026305669	-0.059366	0.28081296	0.47555099
Gm20257	18	1.734789324	1.556717643	-0.178071681	0.28098541	0.475788135
Gnl3	19	1.369376082	1.121301562	-0.248074519	0.28109508	0.47580911
Orc6	17	0.561587751	0.495012561	-0.06657519	0.28103838	0.47580911
Myo5c	38	40.68551775	39.0178996	-1.667618145	0.28108959	0.47580911
Pde2a	64	4.144973187	3.341039363	-0.803933824	0.28129885	0.476099108
Snip1	58	1.198580438	0.996093465	-0.202486973	0.28146443	0.476252862
Sipa1	18	1.385208289	1.912248606	0.527040317	0.28148706	0.476252862
'30559C18F	32	2.45009388	2.194118635	-0.255975244	0.28142951	0.476252862
Cluap1	11	0.903395172	1.105170055	0.201774883	0.28152906	0.476269013
Zer1	36	2.870281544	2.603145586	-0.267135958	0.28168799	0.476482947
Slc25a39	74	0.974095658	1.162910126	0.188814468	0.28180484	0.476625667
Klf2	9	1.510238462	1.735949046	0.225710584	0.28205117	0.476932337
Hspa13	10	1.801971798	1.492631382	-0.309340416	0.28203658	0.476932337
Pomgnt2	25	1.457033775	1.269524973	-0.187508802	0.28212071	0.476994969
Zfp251	12	1.12623262	1.650101137	0.523868517	0.28224561	0.477151161
Cldn7	23	23.23376407	26.34752436	3.11376029	0.28228213	0.477157928
Samd8	59	1.845548181	1.495176926	-0.350371256	0.28242972	0.477352418
Btbd8	52	1.105617142	1.116203552	0.01058641	0.2825775	0.477547189
Fam134b	49	2.157894135	2.029808295	-0.12808584	0.28277725	0.477829727
Znrf2	150	1.03327634	0.950844421	-0.082431919	0.28287915	0.477892006
)31408D14F	1	31.19266055	25	-6.19266055	0.28287924	0.477892006
Cdc42se1	35	1.656681596	1.564125746	-0.09255585	0.28293376	0.477929082
Ctbp1	102	1.378713359	1.094933573	-0.283779787	0.28298887	0.477967155
Klhl32	53	6.999148649	8.457385569	1.45823692	0.28306987	0.478048948
Sh3bp5l	2	1.257861635	1.906779661	0.648918026	0.28321184	0.478233669
Fam133b	74	1.128109126	1.111744139	-0.016364987	0.28348879	0.478646238
Galt	11	3.182939245	2.332694778	-0.850244467	0.28352578	0.478653625
Rsf1	37	1.272523738	1.086972521	-0.185551217	0.28367445	0.478849515
Dock9	113	2.203006961	2.595863571	0.39285661	0.28372183	0.478874404
Ing4	7	1.809739381	1.771804468	-0.037934913	0.28375717	0.478878972
Dynlt1a	14	2.021320875	1.086414609	-0.934906266	0.28380826	0.478910123
Cdca7	16	1.11963657	0.849574255	-0.270062315	0.2840792	0.479222659

700066J03R	1	100	99.26470588	-0.735294118	0.2840731	0.479222659
P2ry2	18	1.54222065	0.767396757	-0.774823893	0.28409145	0.479222659
Rbm26	61	1.40973413	1.204402104	-0.205332026	0.28422345	0.479390226
Bsg	7	1.706300635	1.552660705	-0.15363993	0.28456759	0.479915502
Tomm40	57	1.158793782	1.459788388	0.300994606	0.28468062	0.479995803
931406C07R	26	0.798523877	0.975338936	0.176815059	0.28465447	0.479995803
Mtrf1l	34	0.731255059	1.097044913	0.365789854	0.28477675	0.480102717
Rrp12	17	10.28875903	11.51030774	1.221548713	0.28492178	0.480292047
Set	90	1.181328551	1.303964578	0.122636027	0.28541542	0.481068908
Tmem206	53	1.271433845	1.066117466	-0.205316378	0.28563378	0.48125644
Cables2	46	1.607511172	1.411472826	-0.196038346	0.28559322	0.48125644
Epb4.1l4aos	54	2.249669482	1.50472368	-0.744945802	0.2856409	0.48125644
Kbtbd7	43	1.098131124	0.910188799	-0.187942325	0.28565787	0.48125644
Haus6	25	1.006874979	1.318235082	0.311360103	0.28587675	0.481530582
Csnk1g1	17	2.484965086	1.932679233	-0.552285853	0.28588622	0.481530582
Dusp16	71	1.207167865	1.090167582	-0.117000283	0.28600034	0.481667511
Tuba8	1	32.32323232	25.92592593	-6.397306397	0.2860907	0.481709116
Natd1	38	1.43657445	1.095314674	-0.341259776	0.28608253	0.481709116
Ppp4r1	208	1.09523718	1.108819628	0.013582448	0.28616847	0.481729508
Fbxw17	16	2.336369264	2.256415893	-0.079953371	0.2861501	0.481729508
Trmt1l	7	1.508738734	1.47484946	-0.033889275	0.28629551	0.481777566
Mob1b	26	1.75104859	1.72397562	-0.027072969	0.28626411	0.481777566
Vhl	47	1.300743734	1.403780709	0.103036974	0.28627668	0.481777566
Fnta	62	0.89723067	0.930826562	0.033595892	0.28637405	0.481854469
Zswim4	41	2.047921448	1.904443873	-0.143477574	0.28644575	0.481903064
310069G16R	28	43.09402983	41.39730769	-1.696722141	0.28646861	0.481903064
Paip2b	16	1.187776224	0.745686384	-0.44208984	0.28664917	0.482030028
Bad	40	0.957443486	1.047504009	0.090060523	0.28658685	0.482030028
Zfp532	18	1.793994019	1.770676512	-0.023317507	0.28667548	0.482030028
Prr3	57	1.08377731	0.924289267	-0.159488043	0.28666303	0.482030028
Stambpl1	43	1.604102545	1.300542519	-0.303560026	0.28671036	0.482033434
Crct1	4	68.46285869	66.14340249	-2.319456204	0.28720788	0.482814578
Chtf8	76	1.114583039	1.236442302	0.121859263	0.28748062	0.483162361
Cblb	49	1.104435627	0.910469849	-0.193965778	0.28746011	0.483162361

Gm15708	1	74.53703704	78.65168539	4.114648356	0.28751581	0.483166165
Dexi	15	2.425577152	1.760156813	-0.66542034	0.28761095	0.483270711
Gnpda1	33	1.428917943	0.963168905	-0.465749039	0.28772509	0.483351801
Mark2	60	1.900237557	1.749223968	-0.15101359	0.28769636	0.483351801
Zfp445	36	1.263581385	1.429092704	0.16551132	0.2879784	0.483721957
700084E18R	21	1.687318083	1.939697392	0.252379309	0.28814162	0.483885338
Chchd4	14	0.815695295	0.505865137	-0.309830158	0.28811831	0.483885338
Mfsd4	41	3.523685705	3.197383386	-0.32630232	0.28830554	0.484105222
Gid8	66	1.113029356	0.941269366	-0.17175999	0.2885693	0.48438183
Dnajc17	7	0.357142857	1.169678398	0.812535541	0.28856676	0.48438183
Polr3k	11	1.420467546	0.889446476	-0.531021071	0.28851996	0.48438183
Jtb	45	2.252246391	2.315595146	0.063348755	0.28862051	0.48441238
Usb1	53	1.170778265	1.27077678	0.099998515	0.28867838	0.484454086
Map3k12	30	1.725847865	1.731863197	0.006015332	0.28873206	0.484488768
Spred2	12	1.62005703	2.456562092	0.836505062	0.28896388	0.484822322
Pnck	17	15.08339027	16.06257611	0.979185837	0.28906144	0.484875131
Adrbk1	79	1.442654061	1.312800888	-0.129853172	0.28904243	0.484875131
Ercc1	20	1.373572755	1.507990411	0.134417656	0.28912838	0.484931976
Amotl1	24	3.662140034	3.210218436	-0.451921598	0.28935886	0.485263078
030443J06R	5	0.555555556	2.564102564	2.008547009	0.28944584	0.485353475
Ccdc174	3	2.063301282	1.193145213	-0.870156069	0.28953435	0.485446425
Mapt	41	1.187507944	0.97078495	-0.216722995	0.28962819	0.48549281
Ptch1	129	1.309540901	1.269423304	-0.040117597	0.28960003	0.48549281
Dbndd2	31	1.636066009	1.147366181	-0.488699828	0.28966473	0.485498601
Herc6	3	2.065435281	0.872196645	-1.193238636	0.28971005	0.485519101
Fbxl19	42	2.636030239	2.388651591	-0.247378648	0.28974361	0.485519905
Tssc1	14	1.594416209	1.159000338	-0.435415871	0.28988134	0.48558346
Wwp1	76	1.156452109	1.182372751	0.025920642	0.28984847	0.48558346
Rps15	61	1.274881966	1.221947701	-0.052934265	0.28988484	0.48558346
Becn1	43	1.261798212	1.150049697	-0.111748516	0.28991391	0.48558346
Tmem150b	2	21.46212566	25.36231884	3.90019318	0.28996162	0.485607951
Nsa2	8	0.997018666	1.051378768	0.054360102	0.29010887	0.485799109
Oxct2a	6	15.44117647	12.97893822	-2.46223825	0.29038853	0.486181567
Gm10125	78	0.985184773	1.023597575	0.038412801	0.29040353	0.486181567

Rnf145	79	1.193361428	1.100798634	-0.092562794	0.29048082	0.486230234
Hectd1	164	0.870106527	0.859298249	-0.010808278	0.29049887	0.486230234
Kdsr	50	2.19851449	1.921131568	-0.277382921	0.29058461	0.486262804
Glul	38	1.399258487	1.453862819	0.054604332	0.29055378	0.486262804
S100pbp	12	1.519790037	1.549415489	0.029625452	0.29069304	0.486388796
Rbm42	40	1.198463717	1.203976159	0.005512442	0.29084353	0.486576057
Ivns1abp	54	1.501784441	1.016433097	-0.485351344	0.29087128	0.486576057
Atp6v1g2	7	1.781190117	2.288181221	0.506991104	0.29094214	0.486619762
Cdc73	85	0.839985613	0.971544136	0.131558523	0.29103006	0.486619762
Adh5	52	1.388726933	1.061096486	-0.327630448	0.29100983	0.486619762
Wdr5b	5	2.684282794	2.905766399	0.221483605	0.29097037	0.486619762
Cd274	2	2.84436562	4.024628555	1.180262936	0.29126233	0.486952659
Sp4	113	1.304572821	1.111260936	-0.193311885	0.29135785	0.487001374
Ugp2	57	1.027001603	1.144983632	0.117982028	0.29133102	0.487001374
Crip2	67	1.574148009	1.378775345	-0.195372664	0.29164226	0.487354342
)21536K21F	15	2.273589598	1.478518811	-0.795070787	0.29165512	0.487354342
Ahsa1	12	0.912168817	1.530654533	0.618485716	0.29166865	0.487354342
Kifap3	17	1.167050914	1.250049862	0.082998948	0.2917403	0.48741856
Ccdc62	33	1.638146633	1.091562736	-0.546583897	0.29204016	0.487775643
Yars2	42	0.886974069	1.084657959	0.19768389	0.29205376	0.487775643
Snrpe	46	0.578871145	0.617485594	0.038614449	0.29199189	0.487775643
Dtx2	4	2.836021505	4.771505376	1.935483871	0.29220096	0.487965954
Tbk1	68	1.450153183	1.342238401	-0.107914782	0.29229575	0.48806871
R74862	2	3.939262087	3.513149268	-0.426112819	0.29260013	0.488452965
Fbxo6	32	1.32780268	1.434416608	0.106613928	0.29261289	0.488452965
Man2c1os	7	0.760431154	0.440249433	-0.320181721	0.29262573	0.488452965
Tbrg3	10	1.012891566	1.349835661	0.336944095	0.29266121	0.488456622
Soat1	25	0.574710102	0.739291099	0.164580997	0.29283303	0.488627232
Cd24a	56	1.571648072	1.384985411	-0.186662661	0.29286333	0.488627232
Lincpint	15	2.173489297	2.06741452	-0.106074776	0.29281686	0.488627232
Trim56	4	4.767887849	4.025712344	-0.742175505	0.29293912	0.488642565
Ubald1	72	1.294223395	1.133685457	-0.160537938	0.29291672	0.488642565
Lincenc1	4	80.15298634	77.87163703	-2.281349311	0.2930344	0.488745939
P4ha1	81	1.556111905	1.386738436	-0.169373469	0.29320681	0.48892236

Pdlim1	70	4.339869084	3.671529706	-0.668339378	0.29320458	0.48892236
Xylb	5	3.141940048	2.136761494	-1.005178555	0.29331045	0.489039608
Taf2	38	2.166230297	1.431750518	-0.734479779	0.29337475	0.489091239
Zxdc	56	1.139019708	1.224634635	0.085614927	0.29343592	0.489137637
Xab2	12	1.55725938	2.147634621	0.59037524	0.29362662	0.489399931
Wisp1	2	36.6114149	31.46551724	-5.14589766	0.29379111	0.489475074
Gm20337	22	1.606714657	1.3321699	-0.274544757	0.29374443	0.489475074
430408G22R	5	7.867160617	6.344405694	-1.522754923	0.29380513	0.489475074
Scyl2	33	1.151267382	0.954342696	-0.196924685	0.29373593	0.489475074
Bmp2k	155	1.15821581	1.029995671	-0.128220139	0.29394094	0.489645743
Bhlhe40	19	2.797554033	2.089692516	-0.707861517	0.29406166	0.489784541
Bcan	73	14.43442946	15.30763127	0.87320181	0.29409102	0.489784541
Trib1	103	1.588801597	1.33547129	-0.253330308	0.29419507	0.489902231
Lig1	11	0.767113261	0.483320135	-0.283793126	0.29424062	0.489912131
Sfxn4	2	0.806451613	3.246753247	2.440301634	0.29426779	0.489912131
Bag5	65	1.218663147	1.056013736	-0.162649412	0.29433659	0.489971085
Xrcc3	11	1.127469761	0.244062244	-0.883407517	0.29466129	0.490455966
Vwc2	18	44.0271353	48.64319105	4.616055743	0.29472828	0.4904562
Dopey2	50	1.257351681	1.247880108	-0.009471573	0.29471767	0.4904562
Zfyve21	48	1.218582485	1.17159357	-0.046988915	0.29497015	0.490712882
Ptcd2	22	1.432556358	1.269173575	-0.163382783	0.29495852	0.490712882
Tmed2	85	1.181917685	1.106923732	-0.074993953	0.29501629	0.490712882
Mrps35	52	0.959564068	1.056221462	0.096657395	0.29499832	0.490712882
Adar	26	4.497898074	3.591805969	-0.906092105	0.29510081	0.490797831
Pink1	74	1.522814263	1.323216853	-0.199597411	0.29520789	0.490920275
Anapc11	44	0.730037174	0.763979348	0.033942174	0.29534697	0.491040268
Ptpn2	70	1.257451681	1.157546633	-0.099905048	0.29534497	0.491040268
Prdm15	111	1.24567146	1.257228559	0.011557099	0.29561757	0.491386189
Slc25a35	29	1.656894832	1.982316714	0.325421883	0.295622	0.491386189
Nrg1	4	87.64858766	88.65274131	1.004153645	0.29568007	0.49142704
Rhot2	14	2.001176092	2.458041405	0.456865313	0.29581276	0.491591889
Ift81	30	1.710220302	1.202097587	-0.508122714	0.29592988	0.491683876
Bcl7c	48	1.145481193	0.983130524	-0.162350669	0.29593512	0.491683876
Commd3	19	0.565778472	0.696418903	0.130640431	0.29599742	0.491731696

Fignl1	19	0.462568727	0.852270096	0.389701369	0.29636734	0.492290504
Col6a2	13	1.829473729	1.820610331	-0.008863398	0.296498	0.492435133
Lamtor1	45	1.342279505	1.076047649	-0.266231857	0.2966222	0.492435133
AI197445	2	6.767549391	10	3.232450609	0.29655946	0.492435133
Pm20d2	9	1.888313447	1.135059269	-0.753254178	0.29661562	0.492435133
Tomt	45	1.342279505	1.076047649	-0.266231857	0.2966222	0.492435133
Gpbp1l1	79	1.289239987	1.36126691	0.072026923	0.29697062	0.492846293
Ncdn	16	1.34439901	1.585417884	0.241018875	0.29695138	0.492846293
Ctc1	15	0.954752525	0.66673759	-0.288014935	0.29694056	0.492846293
Sh3bgrl3	18	2.372028265	1.728545022	-0.643483243	0.29748752	0.493648302
Tcf7l1	82	2.548404556	2.599842456	0.051437901	0.29789612	0.494270429
)30544D05F	9	48.42997262	50.68280089	2.252828269	0.29805635	0.494368587
Sirt2	28	1.447382588	1.85491093	0.407528342	0.29804061	0.494368587
Pyroxd1	23	1.517247709	0.992562229	-0.52468548	0.29799994	0.494368587
Pop1	37	1.439359375	1.421952471	-0.017406904	0.29823915	0.494615884
Gk2	2	96.95820318	98.5244641	1.566260914	0.29827315	0.494616359
Ttc37	4	0.563909774	1.226993865	0.663084091	0.29835114	0.494689787
Hmox2	24	1.141043401	0.826306844	-0.314736557	0.29867512	0.495171014
Ruvbl2	7	3.209529324	1.252821495	-1.956707829	0.29894141	0.495500544
Dse	40	1.343221237	1.569503449	0.226282211	0.29894099	0.495500544
Srsf2	84	1.177592534	0.97710453	-0.200488004	0.2990387	0.49560582
Wnt10b	23	14.66903845	16.53636691	1.867328462	0.29913214	0.495704697
Cd3eap	24	2.12180191	1.89541937	-0.226382541	0.29927491	0.495885271
Gm38413	2	85.95559846	82.23529412	-3.720304338	0.29938056	0.495892343
Snrnp27	11	0.728887564	1.365910808	0.637023244	0.29935265	0.495892343
Agtpbp1	170	1.072759266	1.172197163	0.099437897	0.29932373	0.495892343
)33412E12F	20	1.773997064	1.671090347	-0.102906717	0.29953195	0.496087107
Zfp687	79	1.429849925	1.672948597	0.243098672	0.29960569	0.496153247
Diap2	43	15.17722649	15.68804081	0.510814319	0.2996861	0.496230395
Ndfip1	87	1.345705964	1.260155333	-0.08555063	0.29973711	0.496258861
Cars	18	0.987587258	0.71874092	-0.268846338	0.29991802	0.49650237
Fis1	24	2.796890326	1.873802953	-0.923087372	0.30002446	0.496622547
Ampd2	65	1.652029703	1.482968105	-0.169061598	0.30007182	0.496644928
Acot6	7	5.129224016	5.354207789	0.224983774	0.30021046	0.496818344

Zfp319	96	1.287907311	1.236312832	-0.051594479	0.3002768	0.496872095
Hiat1	79	1.013036089	1.141229135	0.128193046	0.30036837	0.496911554
Sema6c	22	1.555638489	1.277755285	-0.277883204	0.3003442	0.496911554
Cxcl5	8	6.726972374	6.040768366	-0.686204007	0.30071649	0.497431377
Usf1	61	1.40062702	1.514002403	0.113375383	0.30082566	0.497555878
Zfp113	16	1.121466466	1.602162943	0.480696477	0.30091104	0.497641009
Leo1	10	2.623598338	2.134322409	-0.489275929	0.30105121	0.497650428
Lgalsl	105	1.181443551	1.03592239	-0.14552116	0.3010524	0.497650428
Cyb561a3	5	1.17936521	1.900645161	0.721279952	0.30098002	0.497650428
Tagln	19	65.52767825	63.76038059	-1.767297651	0.30099501	0.497650428
Rbm3	17	16.47490699	19.88014037	3.405233376	0.30119149	0.497824275
Mdm2	66	4.74152761	4.190539902	-0.550987708	0.30125465	0.497872595
Rab13	16	4.913941056	4.015377952	-0.898563104	0.30189533	0.498852567
Metap1d	28	1.369400564	1.383053376	0.013652812	0.30191561	0.498852567
Snord42b	7	1.744803572	2.520929259	0.776125687	0.3020156	0.498905426
Rpl23a	7	1.744803572	2.520929259	0.776125687	0.3020156	0.498905426
Tiprl	12	0.670960816	0.957715573	0.286754757	0.30217807	0.499100517
Pusl1	6	2.428971399	1.881035942	-0.547935457	0.30220172	0.499100517
Sp2	49	1.233112526	1.362159719	0.129047193	0.3023486	0.499286903
Zfp36l1	7	0.757689799	1.060070079	0.302380281	0.30280734	0.499988174
Camk4	50	4.922408831	5.182245772	0.259836941	0.30287788	0.500026387
Zwint	8	2.316662262	0.810657596	-1.506004666	0.30289863	0.500026387
Lman2l	35	1.439658267	1.262492176	-0.17716609	0.30297508	0.50009633
Rps4l	21	87.92712008	86.34403715	-1.583082936	0.30303221	0.500134358
Wdr12	37	1.103827365	1.260021718	0.156194352	0.30306992	0.500140352
Rnf167	8	0.974557697	1.322083249	0.347525552	0.30341138	0.500462263
Slc25a13	38	1.899750808	1.671904688	-0.22784612	0.30344327	0.500462263
Sdhd	3	2.683178535	3.452473845	0.769295311	0.30352883	0.500462263
Mab21l1	3	74.72798368	76.69551206	1.967528383	0.30338181	0.500462263
Tmem107	31	1.002708223	1.211389795	0.208681573	0.30353783	0.500462263
Parp9	8	2.19302434	1.917030177	-0.275994163	0.30349756	0.500462263
)33428I22R	9	28.67164659	31.64038855	2.968741958	0.30347028	0.500462263
Tbccd1	13	0.752929233	0.383484503	-0.36944473	0.30347957	0.500462263
Mtfr1l	74	1.811422881	2.013039756	0.201616875	0.30361013	0.500525233

Fuca1	44	1.319537941	1.397301555	0.077763614	0.30377649	0.500643871
Axin1	77	1.276870021	1.297797038	0.020927017	0.30377271	0.500643871
Ccdc12	22	1.710218715	1.67799404	-0.032224674	0.30378445	0.500643871
Zfp119b	6	0.616355704	1.003219277	0.386863573	0.30423041	0.501322513
Dusp12	27	1.533618149	1.015876896	-0.517741252	0.3043236	0.501419769
Lig3	70	1.363233543	0.914914037	-0.448319506	0.3044169	0.501517193
Wfs1	54	1.975959841	1.555847658	-0.420112184	0.30458709	0.501684916
Gm5464	41	1.341546653	1.131541009	-0.210005645	0.30458686	0.501684916
Aasdhppt	64	1.201406729	1.052624652	-0.148782077	0.30463412	0.501706075
Mir1945	37	0.96794387	1.25349671	0.28555284	0.30477316	0.501742433
Glb1l	9	0.575884691	1.146321753	0.570437062	0.30479297	0.501742433
Otud4	151	1.096997695	1.066726614	-0.030271082	0.30470162	0.501742433
Sypl2	75	19.61168212	20.51368201	0.901999884	0.30476993	0.501742433
Ssfa2	76	1.059528839	1.030842602	-0.028686237	0.30491832	0.501892478
Paqr4	43	1.635171996	1.470443069	-0.164728928	0.30513226	0.502188281
Rab2b	14	1.210863206	1.210303674	-0.000559532	0.30550732	0.502657373
Spata2	58	1.475552205	1.296416107	-0.179136098	0.30552004	0.502657373
Fam168a	10	1.553756693	2.29770519	0.743948497	0.30546727	0.502657373
Dpysl4	36	40.5326918	41.22571331	0.693021513	0.30561727	0.502760967
Iars2	55	1.34801408	1.523552554	0.175538474	0.30568349	0.502813538
Ccsap	45	3.153548249	2.661032966	-0.492515283	0.30577803	0.502856305
Eri2	29	0.689411547	0.520709965	-0.168701582	0.30575152	0.502856305
Srl	1	93.76854599	91.82692308	-1.941622917	0.30583766	0.502898007
Rabgap1	41	1.831291046	2.107500101	0.276209055	0.3060793	0.503238958
Mfn2	26	1.403702688	1.323350807	-0.08035188	0.30621624	0.503359269
Xpo5	18	1.940591022	1.672179981	-0.268411041	0.30622108	0.503359269
Camta2	50	1.22252713	1.420068087	0.197540957	0.3062859	0.503409419
Dynlt1b	3	5.237376847	5.492426243	0.255049397	0.30641258	0.503561232
Arhgef4	33	2.117648606	2.042783973	-0.074864633	0.30649449	0.503607242
230319C09F	23	3.052409628	2.380584105	-0.671825522	0.30651255	0.503607242
Arl4d	29	1.424984002	1.481250947	0.056266945	0.30654354	0.503607242
Mir2861	9	1.668989471	1.770851771	0.1018623	0.3067957	0.503908681
Mfsd7b	51	1.251377752	1.140179102	-0.111198649	0.30676937	0.503908681
Top3b	46	1.67554707	1.650859889	-0.024687182	0.30689921	0.504022269

Rhobtb2	26	0.99240969	1.324663172	0.332253482	0.30710821	0.504309079
Cyc1	54	1.385156128	1.312456925	-0.072699203	0.3071748	0.504361986
Eva1a	21	4.112148685	3.927391065	-0.18475762	0.30749753	0.504835398
Tuba1b	33	0.675142488	0.9280622	0.252919713	0.30784874	0.505355459
Erich2	25	2.904537819	2.83135963	-0.073178189	0.30789375	0.505372802
Rab11fip3	53	1.318534225	1.050093568	-0.268440657	0.30795269	0.50541301
Dhdds	3	2.693602694	2.322824273	-0.37077842	0.30811363	0.5056206
Gins1	13	1.232543013	1.054417313	-0.1781257	0.30815239	0.505627658
Mrpl20	11	0.652991383	1.209971245	0.556979862	0.30819135	0.505635056
Taf1a	7	3.5734891	2.47107987	-1.10240923	0.30825335	0.505668849
i30013K17R	10	96.79710944	96.15860302	-0.638506422	0.30828087	0.505668849
Enc1	79	1.144244966	1.066143441	-0.078101526	0.30831691	0.505671429
Ncoa4	13	2.137849639	2.636726437	0.498876797	0.30844498	0.505824947
Fam185a	23	1.101104552	1.214898927	0.113794375	0.308486	0.505835692
Ept1	35	1.422945089	1.78200869	0.359063601	0.30857708	0.505928502
Epb4.1l5	59	1.303004843	1.135251864	-0.167752979	0.3087233	0.506111678
Hsf1	66	1.270044486	1.417800009	0.147755523	0.30883587	0.506239681
Usp16	63	1.254954487	1.398519721	0.143565233	0.30887992	0.506255328
Gpatch2	42	1.481891144	1.177632981	-0.304258163	0.30908251	0.506515026
Trpc4ap	46	1.576784243	1.517689658	-0.059094585	0.30910741	0.506515026
Klhdc3	20	1.47301293	1.555257267	0.082244337	0.30919629	0.506604105
Rnf103	51	0.922847152	0.929786835	0.006939683	0.30923296	0.506607627
Bap1	27	0.900707554	1.048251455	0.147543901	0.30939314	0.506756878
Cpsf1	39	1.366643159	1.111506954	-0.255136205	0.30938482	0.506756878
Mmgt2	61	1.924149255	1.670671639	-0.253477617	0.30944453	0.506784496
Nfyb	78	1.032440762	0.898854882	-0.13358588	0.30954796	0.506897299
Sulf1	6	18.56263205	14.00246597	-4.560166082	0.30961796	0.50695536
Crebrf	59	0.772658899	0.82353482	0.05087592	0.30980296	0.507201674
Gtf2h1	68	1.335693967	1.036777342	-0.298916625	0.30988385	0.507277517
Foxj2	90	1.54425921	1.534854162	-0.009405049	0.30993436	0.507303598
Ankrd35	1	98.15668203	99.14285714	0.986175115	0.30998505	0.507329978
Zfp609	87	1.725803373	1.736780654	0.01097728	0.3100221	0.507334033
Tmem50a	52	1.809649505	1.492726444	-0.31692306	0.31011196	0.507424495
'00049A03R	5	1.471491228	0.612244898	-0.85924633	0.31016028	0.507446983

Mir7032	1	97.17514124	98.62385321	1.448711968	0.31024571	0.507482389
Nptn	41	1.409995188	1.730403354	0.320408166	0.31025109	0.507482389
Dscc1	35	1.615319027	1.608176096	-0.007142932	0.31034996	0.507587527
Prkar2b	75	1.407850753	1.253847116	-0.154003637	0.31050472	0.507727465
Ctnnal1	53	1.064757666	1.179645802	0.114888137	0.31047799	0.507727465
Gm20324	58	1.499027067	1.256666366	-0.242360701	0.31060042	0.507827358
Mtg2	42	1.46518509	1.291626439	-0.173558651	0.31077399	0.508054531
Smim15	35	1.279286584	1.141755197	-0.137531388	0.31090182	0.508206891
Ptpn21	39	1.366444605	1.127158801	-0.239285804	0.31101512	0.508335477
Hpcal1	43	1.606438622	1.568638813	-0.03779981	0.31119701	0.508576115
Qser1	70	1.388275612	1.533479063	0.145203451	0.31127104	0.508640453
Add3	37	1.12148375	1.06127617	-0.060207581	0.31145578	0.508828997
Ltv1	25	1.789779853	1.464001919	-0.325777934	0.31144796	0.508828997
Ccdc22	7	18.85165442	16.95619858	-1.895455838	0.31151659	0.508871701
Sdc4	33	1.780466726	1.776811744	-0.003654982	0.31155856	0.508883607
Sez6l2	16	6.340615868	6.08026274	-0.260353128	0.31181519	0.509246083
Ginm1	44	5.469810156	5.214523068	-0.255287088	0.31185934	0.509261518
Tmbim4	20	1.360135375	1.305735075	-0.0544003	0.31201144	0.509453194
Azi2	41	2.066519021	1.753557899	-0.312961122	0.31226896	0.509760232
Dohh	8	0.847005469	0.885775335	0.038769866	0.31224147	0.509760232
Akt2	11	0.942449045	1.063193857	0.120744811	0.31233616	0.509813209
Itga1	23	2.53137914	2.372949191	-0.158429949	0.31237619	0.50982169
Rbck1	27	1.521978093	1.659420863	0.13744277	0.31244558	0.50982169
Ghsr	14	25.62850794	27.00717346	1.378665525	0.31241554	0.50982169
Zdhhc16	34	1.5194425	1.562579438	0.043136937	0.31272115	0.510165141
Kif17	82	1.707973237	1.138969798	-0.569003438	0.3127256	0.510165141
Rpl24	30	1.537553357	1.499030701	-0.038522656	0.31277371	0.510186901
Rnf11	94	1.249815645	1.323289265	0.07347362	0.31291094	0.510354025
Slc35f6	20	2.594033655	2.175069929	-0.418963726	0.31300465	0.510450132
Hps1	28	1.36392564	1.352407754	-0.011517885	0.31310599	0.51055865
Mtus2	88	4.13891719	5.092042287	0.953125097	0.31321389	0.510677851
Esd	4	1.684782609	0.531914894	-1.152867715	0.31325294	0.510684773
Atp1b3	94	8.112370319	8.43519272	0.322822401	0.31341284	0.510888687
Zfp108	5	12.47944314	11.39707478	-1.082368359	0.31348438	0.510948549

Rnf113a2	10	5.646790191	4.652011287	-0.994778904	0.31370147	0.511245594
Phtf1	13	0.957019819	0.790983534	-0.166036285	0.31395968	0.511609596
Ube2w	89	5.138155047	4.455428955	-0.682726092	0.31404289	0.511688363
Ptpn3	31	2.388590021	1.773799744	-0.614790277	0.31409921	0.511723302
Guk1	18	1.518738594	1.099505475	-0.419233119	0.31418706	0.511809598
Slc35a5	11	0.5021886	1.200907166	0.698718567	0.31473586	0.512646695
Acad9	26	1.073319409	1.027020068	-0.046299341	0.31490359	0.512862956
Gstt3	10	6.599186544	7.576065396	0.976878852	0.31498245	0.512922445
Psma3	41	2.030856181	1.753125437	-0.277730744	0.31501002	0.512922445
Vps13a	99	1.283239862	1.20362065	-0.079619212	0.31523302	0.513228599
930523C07R	13	43.10248588	43.9140087	0.811522818	0.31550923	0.513621307
Gigyf2	54	1.240537318	1.221683871	-0.018853448	0.31566451	0.513817084
Sf3a1	35	1.352772113	1.677912206	0.325140093	0.3159041	0.514150043
Smg6	21	1.573471999	1.065052307	-0.508419693	0.31599117	0.514234713
Sumo2	96	1.039923737	1.036776242	-0.003147495	0.31609534	0.514290162
Nol6	19	1.249880706	1.667952384	0.418071678	0.31607117	0.514290162
Ciapin1	39	0.818808808	0.988812736	0.170003928	0.31615134	0.51432425
Ube2d2a	45	1.748298721	1.649131686	-0.099167035	0.31623782	0.514350892
Ubtd1	116	1.500647121	1.461747398	-0.038899723	0.31620838	0.514350892
Casc3	40	1.706563428	1.307493788	-0.39906964	0.3165763	0.514844364
Sp3os	18	1.526499127	1.494545471	-0.031953656	0.31671643	0.515015164
Hnrnpl	63	1.130652627	1.27234123	0.141688603	0.31682459	0.515133971
Cebpg	79	1.350623946	0.995008149	-0.355615797	0.31689781	0.515195931
Lcmt1	8	1.211056745	1.184712307	-0.026344438	0.31694906	0.515222164
Mir1893	37	0.706489413	0.94568963	0.239200217	0.31715927	0.515506771
Rasgrp2	32	2.696051937	2.789264368	0.093212431	0.31737613	0.515687893
Notch2	62	1.288141882	0.978035228	-0.310106654	0.31736065	0.515687893
Copz1	6	3.169480197	2.423428711	-0.746051485	0.31730732	0.515687893
Bbip1	8	0.977215329	1.169400441	0.192185112	0.31742329	0.515707406
Txndc11	76	1.124563919	1.161043108	0.036479189	0.31746615	0.515719951
Marf1	27	1.438415657	1.511852988	0.073437331	0.31769642	0.516036887
933430I17R	7	1.117608582	0.716553288	-0.401055294	0.31778896	0.516130075
Chat	6	13.10575934	14.78381053	1.678051198	0.31795185	0.516337475
Lamc1	119	1.386339017	1.468731524	0.082392507	0.318167	0.516629688

Rab3a	27	2.600476252	2.446040922	-0.15443533	0.31840792	0.516802335
Slc35f4	19	40.43434319	40.35406327	-0.080279916	0.31840228	0.516802335
Epn2	72	1.930633558	1.673410851	-0.257222707	0.3184142	0.516802335
Ccdc137	19	1.173155658	1.002953559	-0.1702021	0.31831392	0.516802335
Spata33	15	1.82105431	1.916908705	0.095854395	0.31862947	0.517094534
BC003331	63	1.241697918	1.201692687	-0.04000523	0.31867427	0.517110062
Cdk4	9	1.86439815	1.690168897	-0.174229253	0.31883737	0.517317517
Cyfip1	31	1.403856701	1.300305136	-0.103551565	0.31901582	0.517492605
Ppp1r15a	13	1.462902993	0.775799477	-0.687103516	0.31898884	0.517492605
Polr3h	58	1.923946845	1.473008084	-0.450938761	0.31914183	0.517638449
Parp4	23	1.605521129	1.325579705	-0.279941424	0.31917628	0.517638449
Lztr1	46	1.19811574	1.023220578	-0.174895162	0.3196239	0.518307111
Ift88	42	1.620279317	1.158269875	-0.462009442	0.31970454	0.518380596
Gpsm2	40	1.231370847	0.968294359	-0.263076488	0.31982759	0.518522817
Tnrc6c	54	0.876648736	0.780475648	-0.096173088	0.31988253	0.518554603
Zfp263	33	1.38303463	1.323443065	-0.059591565	0.32011105	0.51886773
Brwd1	132	1.571454147	1.443104632	-0.128349515	0.32025758	0.518942677
Kcng4	2	6.259390241	5.063321103	-1.196069138	0.32021522	0.518942677
Mid2	3	95.35834536	96.24380039	0.885455032	0.32026338	0.518942677
Tdp2	77	1.257804379	1.096991329	-0.160813051	0.32042078	0.519083079
B3gnt4	3	93.88150559	92.62827804	-1.25322755	0.32039466	0.519083079
Ddias	46	1.6054184	1.723656319	0.118237919	0.32058216	0.519251737
Mtx1	58	1.179520149	1.131530333	-0.047989816	0.32063105	0.519251737
Mtpap	11	1.168183612	1.372601428	0.204417815	0.32062439	0.519251737
Yipf2	63	1.33729451	0.992744408	-0.344550103	0.32080414	0.519474729
Dad1	33	1.371062414	1.096139874	-0.27492254	0.32088984	0.519498844
Ecsit	29	1.264189954	1.832366164	0.568176211	0.32087483	0.519498844
Pddc1	20	2.54777657	2.138363919	-0.409412652	0.32099502	0.519611791
Plekhg4	47	82.48487051	82.2718979	-0.212972608	0.32112953	0.519772197
Pdgfa	143	1.463818287	1.281327346	-0.182490941	0.3212915	0.519976995
Tnfrsf11a	70	4.791269129	4.656650496	-0.134618633	0.32133997	0.5199981
Aptx	28	1.433722615	1.089916105	-0.34380651	0.32148397	0.520173749
Kpna6	17	2.024378787	2.072947293	0.048568506	0.32152008	0.520174825
Mphosph9	55	1.289190145	1.577985766	0.288795621	0.3217849	0.52043112

Rnf139	59	0.98763499	1.121675592	0.134040602	0.32173015	0.52043112
Trnp1	34	3.95614272	5.410123309	1.453980589	0.32174963	0.52043112
Asphd1	13	4.09394384	5.589322259	1.49537842	0.3223356	0.5212069
Sdhc	16	1.801310695	1.151290679	-0.650020016	0.32230345	0.5212069
Hsd17b11	11	1.171949513	1.791377331	0.619427818	0.32238374	0.521227307
Gm2109	4	4.420666572	4.466945996	0.046279425	0.32243316	0.521244172
Pxmp4	21	2.17198775	2.336472967	0.164485217	0.32246522	0.521244172
Egr2	28	1.646369938	2.067115423	0.420745485	0.32264546	0.521420647
Ncor1	59	1.175857525	1.092933319	-0.082924206	0.32262962	0.521420647
Cdk11b	37	1.039529309	1.144183342	0.104654032	0.32279896	0.521611274
Klf16	150	1.494022186	1.325427867	-0.168594318	0.32286052	0.521653307
Hectd2	92	1.865681367	1.609039085	-0.256642282	0.32296899	0.521771106
Ubn1	78	1.112260047	1.288066293	0.175806246	0.32332343	0.522056339
330032B11F	1	45.45454545	49.41176471	3.957219251	0.32325967	0.522056339
Mir22	6	3.132235383	2.085927692	-1.046307691	0.32330056	0.522056339
Osbpl2	69	1.056784007	1.095432831	0.038648823	0.32319241	0.522056339
Npepps	69	1.417693132	1.430638739	0.012945607	0.32332215	0.522056339
Cadm4	83	2.511423362	2.28309164	-0.228331722	0.32336479	0.522065679
Smtn	11	2.85647847	2.349561526	-0.506916944	0.32340808	0.522078127
Zmynd19	98	1.373077217	1.520304848	0.147227631	0.3234493	0.52208723
Gtpbp10	10	1.522939994	1.193148442	-0.329791552	0.32356431	0.522215425
Rassf1	39	1.23559163	1.514857462	0.279265832	0.32365419	0.522303051
Tma7	13	1.493523142	1.491484136	-0.002039006	0.32375264	0.522404463
Npepl1	61	1.5630696	1.614621414	0.051551814	0.3240984	0.522904882
Rrp7a	23	0.813281849	0.760259571	-0.053022278	0.32429916	0.523093557
Pms2	17	0.937332244	1.430362362	0.493030118	0.32432228	0.523093557
Taf1d	26	0.555608898	0.79644806	0.240839162	0.32425748	0.523093557
Coa4	9	0.740740741	0.820589013	0.079848272	0.32501121	0.524121354
Mzf1	2	3.539823009	1.655629139	-1.88419387	0.32506535	0.524121354
Rabac1	1	4.545454545	2.272727273	-2.272727273	0.32506668	0.524121354
931440J10R	76	1.526805888	1.187751643	-0.339054245	0.32517467	0.524198137
BC017158	21	1.114397776	1.143918761	0.029520985	0.32518575	0.524198137
Muc1	1	3.076923077	1.470588235	-1.606334842	0.32537522	0.524423079
Ppp1r2	29	17.17437779	16.3475169	-0.826860884	0.32539677	0.524423079

Galnt10	46	1.447856484	1.291628494	-0.15622799	0.32553205	0.524525885
Ankrd39	26	1.44018782	1.940076067	0.499888247	0.3255297	0.524525885
Zc3hav1l	12	2.683830955	2.623010819	-0.060820136	0.32558024	0.524545945
Chaf1b	51	1.390576123	1.145719678	-0.244856445	0.32574865	0.524586885
Tyw3	37	1.798003666	1.544090092	-0.253913573	0.32565391	0.524586885
Alg10b	3	0.247831475	0	-0.247831475	0.32572227	0.524586885
Zak	72	1.318888961	1.354965185	0.036076224	0.325738	0.524586885
Crtc2	53	1.264507942	1.430138585	0.165630643	0.32611528	0.52511968
Zmynd8	9	30.23497142	32.68175762	2.446786198	0.32642611	0.525370821
Glt1d1	16	13.39242336	11.90156793	-1.490855432	0.32660862	0.525370821
Gnb4	66	1.275259505	1.299855743	0.024596239	0.32645367	0.525370821
Hspa2	38	2.367582025	2.461291972	0.093709948	0.32649455	0.525370821
Ghrh	3	91.93121693	90.47904122	-1.452175708	0.32656037	0.525370821
Ttc5	3	1.877934272	1.62601626	-0.251918012	0.32642052	0.525370821
Irf4	12	17.8437115	17.57637627	-0.267335229	0.32662928	0.525370821
Bag3	31	1.508276832	1.281609645	-0.226667187	0.32655869	0.525370821
310058I24R	17	1.289768428	1.184339917	-0.105428511	0.32636071	0.525370821
Echs1	6	2.050776465	2.733585198	0.682808733	0.32661935	0.525370821
Shroom3	86	5.027821569	5.673582067	0.645760499	0.32686531	0.525462484
Cmas	60	1.769212869	1.619772753	-0.149440116	0.3268164	0.525462484
Cdkl3	27	2.776708081	2.456820003	-0.319888078	0.32676816	0.525462484
Vti1a	31	1.271570962	1.692967788	0.421396826	0.32678429	0.525462484
510206C17R	70	1.435897989	1.070389001	-0.365508988	0.32684729	0.525462484
Spg21	17	0.783658731	1.217183432	0.4335247	0.32707719	0.525687912
Inafm1	16	3.254487184	2.632960914	-0.62152627	0.32704561	0.525687912
Khdrbs2	48	49.59212061	50.67694799	1.084827382	0.32714725	0.525729334
Myc	42	1.162674374	1.350819733	0.18814536	0.32717461	0.525729334
Wdr25	11	1.303514866	1.378139493	0.074624628	0.3272904	0.525742677
Tcl1b1	1	23.61111111	18.5840708	-5.027040315	0.32727081	0.525742677
Rps6ka5	13	8.403648055	8.755500775	0.35185272	0.32725178	0.525742677
Polr2a	59	1.627258478	1.433052264	-0.194206214	0.32749886	0.526019946
Aatf	72	1.21709796	1.273055494	0.055957534	0.32761962	0.526156325
Clcf1	33	1.808249525	1.281900447	-0.526349078	0.32775864	0.526307755
Cenpm	39	1.120088385	1.133120573	0.013032187	0.32778565	0.526307755

Srsf7	42	1.210193387	0.986048156	-0.224145231	0.32785627	0.526363549
Mir1938	62	1.197741391	1.099741746	-0.097999645	0.32809088	0.526682599
Stx18	37	1.597245562	1.403607999	-0.193637563	0.3282746	0.526689417
30578M01I	3	4.686158587	4.350067412	-0.336091174	0.32817633	0.526689417
Exd1	20	1.777912925	1.433933852	-0.343979073	0.32824126	0.526689417
Mtor	49	1.476160164	1.136463966	-0.339696198	0.32820306	0.526689417
Snx29	64	2.880147528	2.560484874	-0.319662654	0.32824041	0.526689417
Exosc4	32	1.232667126	0.921201293	-0.311465833	0.32835672	0.526759071
Impa1	37	1.450440636	1.303567023	-0.146873613	0.3283898	0.526759071
'00110K17F	10	88.81701134	89.54302782	0.726016482	0.32852105	0.526912001
410016O06F	111	1.010636606	1.049565764	0.038929158	0.32868702	0.52712058
Pias2	69	1.129083578	1.23717141	0.108087832	0.32883675	0.527169465
Dnaaf1	26	1.101632916	1.089694481	-0.011938435	0.32885531	0.527169465
Cd320	3	2.732822733	3.643844943	0.91102221	0.32881702	0.527169465
AU022252	12	2.28947511	1.663422839	-0.626052271	0.3288612	0.527169465
510020C07F	8	1.445676542	0.726310073	-0.719366469	0.32894433	0.527245118
Caml	17	1.435335915	0.997040145	-0.43829577	0.32908097	0.52740653
Tmem131	159	1.112065848	1.112934121	0.000868272	0.32921237	0.527559498
Pibf1	58	1.401933994	1.173606441	-0.228327553	0.32925788	0.527574809
Tor3a	28	1.293836681	1.515020261	0.22118358	0.32942957	0.52773467
Nprl3	33	1.139395525	1.122212105	-0.01718342	0.32942017	0.52773467
Sh3bgrl2	7	8.823490237	7.985695143	-0.837795094	0.3295527	0.527874292
310433D01F	6	53.73763735	54.74512811	1.007490757	0.32961237	0.52791224
Trnt1	11	1.096441786	0.811859734	-0.284582052	0.32976659	0.528098373
Psmg3	6	0.560263653	0.955228763	0.39496511	0.32980057	0.528098373
Map7d1	69	1.225200005	1.129140097	-0.096059908	0.32992805	0.528244873
Eif3d	58	1.120945751	1.275280552	0.154334801	0.33001471	0.528325979
Dcaf8	42	1.196447378	0.754886742	-0.441560636	0.33015351	0.528490523
Cerk	38	1.331695215	1.759585606	0.427890391	0.33025628	0.528597374
430306N03F	4	97.45762712	93.75	-3.707627119	0.33052254	0.528965829
10018M11I	47	1.047306016	1.142759197	0.095453181	0.33065974	0.529070003
Gm1673	57	11.73633381	13.79334403	2.057010219	0.33062468	0.529070003
Tmed10	14	0.336134454	0.833802693	0.497668239	0.33093789	0.529457323
Kif1bp	45	1.323938345	1.093287901	-0.230650444	0.33103477	0.529496859

Gm10814	2	98.36984872	97.00892857	-1.360920151	0.33102643	0.529496859
Fam167b	3	66.25747021	64.16673245	-2.09073776	0.33108171	0.529514227
Col1a1	1	93.75	89.36170213	-4.388297872	0.33115713	0.529577126
Napa	38	1.140302593	1.286848111	0.146545519	0.33126331	0.529645838
BC003965	76	1.149369956	1.217781833	0.068411877	0.33127228	0.529645838
Zfp472	13	2.081832481	1.611134383	-0.470698098	0.3313614	0.529701038
Mob3b	18	1.169460095	0.888266272	-0.281193823	0.33137901	0.529701038
Baz2a	43	1.526447551	1.675961032	0.149513481	0.33148528	0.529813206
Eif4g1	71	1.497336719	1.518170243	0.020833524	0.33176334	0.530199871
Elfn1	28	16.35403239	15.85996173	-0.494070661	0.33196545	0.530465106
Zfp747	14	1.764332431	1.62970079	-0.134631642	0.33225848	0.53087554
Fam214b	25	2.231361122	2.437571282	0.20621016	0.33275982	0.531618688
Sdhaf4	10	0.744305079	1.194561022	0.450255943	0.33279736	0.531620773
Acvr1	128	1.49658911	1.33861853	-0.15797058	0.33300151	0.531888997
Vat1	66	1.640568725	1.51493817	-0.125630555	0.33310391	0.531967532
Gtf2h5	11	0.496770989	0.162337662	-0.334433326	0.33312319	0.531967532
Cyb5a	15	1.759142713	1.487060775	-0.272081939	0.33322084	0.532065565
Med29	15	0.50925592	1.56824283	1.05898691	0.33330816	0.532147091
Txnl4b	25	0.793747627	0.999625827	0.2058782	0.33352426	0.53243418
Tcp11l2	29	1.817657476	1.80758963	-0.010067847	0.33364713	0.532538173
Tcea1	45	1.563544005	1.289260088	-0.274283917	0.33366198	0.532538173
Rnaseh2a	10	2.555215485	3.533333333	0.978117848	0.33369946	0.532540058
Hps6	31	1.641690376	1.376489811	-0.265200565	0.33383249	0.532694423
Fam71e1	32	2.105212831	1.839559233	-0.265653598	0.3338724	0.532700189
Gm2518	65	1.125829754	0.930135929	-0.195693825	0.33408821	0.532986562
Zhx3	100	1.249346773	1.407926451	0.158579679	0.33415893	0.533041435
Ahsa2	54	1.789646672	1.365982697	-0.423663976	0.33421238	0.533068749
Nhlrc1	13	42.62753552	44.35188998	1.724354461	0.33425626	0.533080797
)30579K19F	14	1.529949162	1.365216849	-0.164732313	0.33435926	0.533187105
Mir7062	14	95.11051766	94.42960494	-0.680912727	0.33454978	0.533432948
Cnep1r1	16	1.292855916	1.202967756	-0.08988816	0.33459492	0.533446967
Samd10	33	1.549830612	1.109976362	-0.43985425	0.33487642	0.533721802
Cenpk	39	1.376317424	1.037778876	-0.338538549	0.33481483	0.533721802
Tlr3	7	0.767018217	0.976958525	0.209940309	0.33486871	0.533721802

Pccb	24	0.949120148	0.987589636	0.038469488	0.33500774	0.533840151
310026B05R	105	1.041233953	0.886484337	-0.154749616	0.33502344	0.533840151
Mta1	116	1.433729474	1.539295328	0.105565854	0.33510407	0.53391065
Zfp661	11	4.12630866	5.078947224	0.952638564	0.3353409	0.534114003
Hps5	36	1.414796056	1.04865635	-0.366139706	0.33527205	0.534114003
Atp5s	22	1.339721723	1.006188031	-0.333533692	0.33533166	0.534114003
Ablim1	35	6.703163899	5.920668563	-0.782495337	0.33554243	0.534319008
Smarcd2	61	1.108974486	1.210302847	0.101328361	0.33553154	0.534319008
Asmt	8	98.84848215	99.24667986	0.398197704	0.33568171	0.534482788
Man1a2	134	1.159997179	1.083993459	-0.076003719	0.33598446	0.534674757
Fnip1	16	0.45329036	1.093318296	0.640027937	0.33592957	0.534674757
Ctcf	129	1.070847196	1.121300338	0.050453142	0.33594902	0.534674757
Abhd15	74	2.3023711	2.20611242	-0.096258679	0.33594482	0.534674757
Nob1	40	1.19256457	1.053263621	-0.139300949	0.33597673	0.534674757
Exosc2	9	1.145044381	0.87338394	-0.271660441	0.33631853	0.535103974
Cops7b	5	2.163781563	2.174779113	0.01099755	0.33644747	0.535103974
Tmtc4	51	1.440624275	1.105489227	-0.335135047	0.33647297	0.535103974
Slc9a7	36	24.96146615	24.16709274	-0.794373414	0.33638839	0.535103974
Pak1ip1	38	1.240162781	1.42383451	0.183671729	0.33640781	0.535103974
Mrpl10	8	0.533853545	0.881702753	0.347849208	0.33635326	0.535103974
Llph	4	1.982252298	1.153005464	-0.829246834	0.33668267	0.535337131
Ywhab	40	1.174863044	0.838085485	-0.33677756	0.33669254	0.535337131
530027J09R	36	14.12100437	15.30635443	1.185350058	0.33674111	0.535356346
Lrp12	80	1.603632398	1.481108477	-0.122523921	0.33717678	0.535953867
Rcor1	69	1.455279112	1.170259272	-0.28501984	0.33719	0.535953867
Smim20	43	1.632797248	1.335119102	-0.297678146	0.33727864	0.536006976
530068E07R	42	0.917263925	1.097238789	0.179974864	0.337333	0.536006976
Eif2ak2	26	1.594498624	1.245995918	-0.348502707	0.33730434	0.536006976
Tmem150a	58	1.635229914	1.806106384	0.17087647	0.33755543	0.536302342
Cep70	40	1.30957501	1.242296974	-0.067278037	0.33775656	0.536447634
Bloc1s5	34	1.090281992	0.826990112	-0.26329188	0.33770433	0.536447634
Zfand2b	46	1.369334654	1.145120751	-0.224213903	0.33773849	0.536447634
Rai14	32	2.107711476	1.495868671	-0.611842805	0.33789422	0.536521626
Gemin7	44	2.159400302	2.763166934	0.603766632	0.33791283	0.536521626

Gamt	12	2.307596176	1.850275247	-0.457320929	0.33791268	0.536521626
Eapp	33	1.077509307	1.390122035	0.312612728	0.33795695	0.536533627
Rangap1	11	2.684857303	2.195160917	-0.489696386	0.33803151	0.536593932
Ap2m1	29	1.791748191	1.513663969	-0.278084222	0.33823909	0.53683432
Tma16	50	2.344805971	1.708621126	-0.636184844	0.33831553	0.53683432
Gcc2	5	0.881595882	1.936842105	1.055246224	0.33832928	0.53683432
Hnrnpd	118	1.057995043	1.013578393	-0.04441665	0.33829019	0.53683432
Mrpl50	2	2.586206897	1.616628176	-0.969578721	0.33846655	0.536994064
Vapb	44	0.886020417	0.864601193	-0.021419224	0.33860557	0.537156549
Vav2	90	0.997441507	0.984389566	-0.013051941	0.33867213	0.537204063
Gm15417	37	1.498003583	1.066120215	-0.431883367	0.33876804	0.537277906
)30001C03F	22	1.990380411	1.516744213	-0.473636198	0.33879191	0.537277906
Chmp2b	44	1.665114346	1.250322299	-0.414792047	0.33882871	0.537278196
Tob2	32	1.747830749	1.481655444	-0.266175305	0.33891326	0.537336811
Smg1	83	0.975713145	1.013765883	0.038052738	0.33893891	0.537336811
Hsph1	62	1.552623902	1.398740475	-0.153883427	0.33902344	0.537412758
Lpcat4	93	1.132427088	1.010064085	-0.122363003	0.33914907	0.537437742
H2-DMa	5	3.479938272	2.470423924	-1.009514348	0.33913397	0.537437742
Lmbr1	8	0.295868347	0	-0.295868347	0.3390869	0.537437742
Ppp1ca	78	1.888126224	2.070890216	0.182763992	0.33927158	0.537573825
'00096K18F	57	1.319373024	1.379302719	0.059929695	0.33947629	0.537840107
Baiap2	124	1.416263607	1.439247389	0.022983782	0.33957486	0.537938196
Akap8	61	1.253070084	1.431842161	0.178772076	0.3397063	0.538033734
Ube2j2	49	1.068907439	1.37496939	0.306061952	0.3397085	0.538033734
Nsun3	30	1.972035249	1.601028991	-0.371006258	0.34002195	0.5384276
Col4a1	25	1.162745643	1.145665386	-0.017080256	0.34003057	0.5384276
Gtf3c6	5	0.601503759	1.359079517	0.757575758	0.34008285	0.538452277
Klra8	4	83.67021277	77.68165395	-5.988558812	0.34075795	0.53946296
Pde6b	2	35.73369565	31.96933962	-3.76435603	0.34116406	0.540047612
Arhgef10	22	1.698626179	1.295643655	-0.402982525	0.34126753	0.540153132
Mybl2	48	1.013593461	1.193226001	0.179632539	0.34140389	0.540252416
E2f6	61	8.790090828	8.939737707	0.149646879	0.34139482	0.540252416
Erich1	29	1.631039034	1.459671106	-0.171367928	0.3414965	0.540340692
Gatc	8	1.140929065	1.521056113	0.380127049	0.341653	0.540530042

Txndc15	9	2.149592171	1.586216904	-0.563375267	0.34171895	0.540576091
Mir207	18	1.649628564	1.49171471	-0.157913855	0.34180117	0.540647882
Gtf2f1	5	0.698689956	0.716410981	0.017721025	0.34184842	0.54066433
Plat	9	2.026888436	3.444450679	1.417562243	0.34194174	0.540695381
Ikbkb	34	1.532914008	1.58702191	0.054107902	0.34191582	0.540695381
Micu3	57	1.277859797	1.087726802	-0.190132995	0.34210293	0.540775444
Btbd10	92	1.415958202	1.545677286	0.129719084	0.34203732	0.540775444
Lsm14a	79	1.084233046	0.89202925	-0.192203796	0.34209726	0.540775444
Ppp6r2	72	1.20091994	1.319769365	0.118849425	0.34230541	0.541037225
Ankrd42	32	0.994093171	0.935680275	-0.058412896	0.34239197	0.541115758
Pcyox1	20	1.01917458	0.970287998	-0.048886582	0.34258551	0.541363312
Tmx3	30	1.086556683	1.46576271	0.379206027	0.34263652	0.54138562
Actl10	8	67.46794947	69.46760251	1.999653041	0.34269194	0.541414886
Emp3	2	4.603174603	2.222018601	-2.381156002	0.34304382	0.541679228
Zfp871	12	1.730332994	1.809924103	0.07959111	0.3429169	0.541679228
Itga5	16	1.413959787	1.375366915	-0.038592872	0.34303021	0.541679228
Nxf1	61	1.234608763	1.016769498	-0.217839264	0.34298022	0.541679228
Ppp6r3	31	1.53891927	1.368316005	-0.170603266	0.34295133	0.541679228
Gm16861	8	1.384810703	0.88436154	-0.500449163	0.34325485	0.541895824
Nudt16l1	8	1.384810703	0.88436154	-0.500449163	0.34325485	0.541895824
Aarsd1	15	1.429622801	0.879764837	-0.549857964	0.34337326	0.542024447
Spire2	90	1.309187815	1.297673965	-0.01151385	0.34341645	0.542034309
Zkscan8	3	0	0.660066007	0.660066007	0.34351324	0.542115776
Dnaaf3	12	13.08079812	14.10212041	1.021322297	0.34355594	0.542115776
Exoc4	27	2.92671308	2.083166357	-0.843546722	0.3435789	0.542115776
Cacul1	56	1.351353698	1.465999339	0.114645641	0.34363508	0.542146138
Shb	64	1.043589787	1.091954081	0.048364294	0.34386497	0.542450505
Phrf1	50	1.195984266	1.24992786	0.053943594	0.34392146	0.542481294
Numb	63	1.604734856	1.5724472	-0.032287657	0.34420842	0.542875575
'00012D01I	2	5.662878788	4.042920736	-1.619958052	0.34427008	0.542914474
Abhd4	31	1.71477475	1.499937527	-0.214837223	0.3444903	0.543203387
Ccdc93	12	2.722853592	3.122920727	0.400067135	0.34466001	0.543239115
)30005H10I	24	1.037980624	1.350176579	0.312195954	0.34466104	0.543239115
Tmem185b	70	2.109876175	1.614605617	-0.495270557	0.3446271	0.543239115

D3Ertd254e	55	2.163426355	1.721332536	-0.44209382	0.34455565	0.543239115
Itgb5	27	1.274880074	1.025284045	-0.249596029	0.34470296	0.543246836
Slc38a10	15	1.341226943	1.443363612	0.102136669	0.34485326	0.54342533
l7Rn6	15	1.804682243	1.863779182	0.05909694	0.34502462	0.543550244
Atoh1	33	34.90568641	35.80404425	0.89835784	0.34499995	0.543550244
Rc3h2	44	1.41606285	1.204561508	-0.211501341	0.34504521	0.543550244
Add1	41	1.256549362	1.220788028	-0.035761334	0.34508069	0.543550244
Galnt2	98	1.284755316	1.039895084	-0.244860232	0.34529431	0.543828352
Umad1	33	1.459919293	1.466835189	0.006915896	0.34544455	0.544006577
Utp3	63	1.285399862	1.446529096	0.161129234	0.34562376	0.544113619
Nans	34	0.988616953	0.741605614	-0.247011339	0.34559049	0.544113619
Fam181b	21	58.80273536	59.8095427	1.006807347	0.34556964	0.544113619
Nme6	14	1.679069574	1.685038994	0.00596942	0.34576308	0.544274553
930583K01F	26	1.376861555	1.38779322	0.010931665	0.34590483	0.544439291
Plekhm1	31	1.472113157	1.170012185	-0.302100972	0.34597274	0.544487766
Cnrip1	22	28.29513193	30.94511113	2.649979197	0.34609947	0.544570402
Ggta1	28	1.477025821	1.375406395	-0.101619426	0.34606796	0.544570402
Fbxo30	86	1.531861836	1.63482145	0.102959614	0.3461996	0.544604707
Ednrb	20	40.11565401	41.12283567	1.007181666	0.34623261	0.544604707
Emg1	17	1.135126297	1.104980328	-0.030145969	0.34622366	0.544604707
Zadh2	101	1.360733658	1.264648649	-0.096085009	0.34635169	0.544733626
Chrm4	48	3.778384602	4.747440985	0.969056383	0.34641599	0.544776366
Pdcd10	35	1.831989157	2.096775483	0.264786325	0.34652612	0.544891153
Phax	27	2.542495772	2.191843399	-0.350652374	0.34657144	0.544904027
'00030K09F	7	0.272975432	0.773594636	0.500619204	0.34668139	0.544960117
'00045H11F	46	1.117474299	1.196133364	0.078659064	0.3466632	0.544960117
Mir219a-1	20	16.50720758	17.98225828	1.475050699	0.34674601	0.545003318
Zbtb12	94	1.676623957	1.459544477	-0.217079481	0.34695103	0.545208767
330013E15F	9	1.205132201	1.011570426	-0.193561775	0.34694329	0.545208767
Ociad1	20	1.184603705	1.013733472	-0.170870233	0.34728464	0.545412064
Glt8d1	28	2.012287165	1.163961927	-0.848325238	0.34727521	0.545412064
Traf6	68	1.425228928	1.431881276	0.006652348	0.34736102	0.545412064
Abcb1b	14	0.865924486	0.994499864	0.128575378	0.34713801	0.545412064
Mief2	23	1.033911094	0.797735659	-0.236175435	0.34737775	0.545412064

Pip5k1c	56	1.31860366	1.223683513	-0.094920147	0.3473149	0.545412064
Nfu1	17	0.899902514	0.659978916	-0.239923598	0.34720289	0.545412064
Polr3a	35	1.106774556	1.420618547	0.31384399	0.34719988	0.545412064
Pelp1	21	2.092826853	1.985733859	-0.107092995	0.34759973	0.54568993
Gm14057	3	95.26270457	96.16539224	0.902687675	0.3476291	0.54568993
Psmb3	9	1.707482332	0.735818047	-0.971664285	0.34770279	0.545722004
Atf7ip	20	1.602804036	0.83844651	-0.764357526	0.34772392	0.545722004
Gata3	26	10.79629253	11.04757396	0.251281424	0.34798239	0.546069259
Ccrn4l	42	1.043678956	0.838772641	-0.204906315	0.34807196	0.546151412
Glod4	40	1.243568632	1.221167595	-0.022401036	0.34823113	0.546284325
Mrpl11	6	3.215781515	3.060735881	-0.155045633	0.3482297	0.546284325
Nqo2	8	2.209937817	2.18181391	-0.028123907	0.34833356	0.546386601
Elf1	51	0.866391021	0.979641616	0.113250595	0.3484163	0.546457971
[illegible]10043G02[illegible]	60	1.300235451	1.485696982	0.185461531	0.34861497	0.546711125
Appl2	18	1.046753745	1.603312623	0.556558878	0.34875273	0.546788832
Ikbkap	11	3.274567848	2.327009961	-0.947557886	0.34876068	0.546788832
Sin3a	61	1.453728192	1.553811624	0.100083432	0.3487763	0.546788832
Zfp639	64	1.560873618	1.252996736	-0.307876882	0.34895583	0.547011845
Lias	33	1.187624615	0.924030092	-0.263594523	0.3490362	0.547079381
Nsl1	22	0.867292731	0.777233545	-0.090059187	0.34918274	0.547192175
Idi1	3	2.032520325	0.366300366	-1.666219959	0.3491734	0.547192175
Kif3b	46	6.458802385	5.873505242	-0.585297143	0.34923764	0.547202977
Bloc1s6	8	1.417530808	1.36442069	-0.053110118	0.34926421	0.547202977
Mad1l1	40	1.60748615	1.88583855	0.2783524	0.34931683	0.547226992
Gm16023	25	0.8582455	0.574917	-0.2833285	0.34941699	0.547325461
Pnpt1	14	1.446897181	1.293994563	-0.152902618	0.34950103	0.547398674
Ap3m1	17	1.770244129	1.995924792	0.225680663	0.34991549	0.547989315
Fastkd5	34	1.827908727	1.98262287	0.154714143	0.35030708	0.548544027
Fbp2	15	22.25593689	21.73005961	-0.525877284	0.3503904	0.548615955
Galnt11	57	1.069115414	1.03887008	-0.030245334	0.35051111	0.548746406
Pld3	7	1.703357937	1.350320975	-0.353036962	0.35075187	0.54877204
Poldip2	10	1.910088123	2.158030265	0.247942142	0.35065858	0.54877204
Saal1	13	1.31050117	0.866436123	-0.444065047	0.35073407	0.54877204
Troap	35	1.944022669	1.871760344	-0.072262325	0.35070551	0.54877204

Dctn3	10	1.221353212	1.095277033	-0.126076178	0.35068667	0.54877204
Cnot3	58	0.912981533	0.873645375	-0.039336158	0.35074772	0.54877204
Ino80e	14	1.596059683	1.171172475	-0.424887208	0.35082738	0.548787901
Cdkn2c	133	1.462191893	1.261151499	-0.201040394	0.35086451	0.548787901
Ppp1r18	39	1.294597999	1.476009645	0.181411646	0.35087421	0.548787901
Foxj1	21	1.779567154	1.618568074	-0.160999079	0.35099135	0.548912617
Rbm38	49	2.697419412	2.62422286	-0.073196552	0.35108769	0.549004773
Zfp382	27	1.234952863	1.146838547	-0.088114315	0.35115675	0.549054253
Atg5	49	1.054633204	1.105097574	0.05046437	0.35127828	0.549166222
Znhit1	27	1.732709154	1.410123399	-0.322585755	0.35130321	0.549166222
Atp5d	32	1.030240921	0.83390206	-0.19633886	0.35139261	0.549247457
Arhgap17	49	1.661500036	1.385805604	-0.275694431	0.35166614	0.549616466
Slc26a8	19	8.694227432	9.226292519	0.532065087	0.35205814	0.549877713
Tkt	18	1.122319243	1.040358544	-0.0819607	0.35204994	0.549877713
Uxt	6	20.99228879	19.48894715	-1.503341639	0.35196064	0.549877713
Mocs2	42	1.258165477	1.346214914	0.088049437	0.35204322	0.549877713
Gtf3c4	20	0.569272822	0.88452313	0.315250308	0.3519014	0.549877713
230072C01F	6	20.99228879	19.48894715	-1.503341639	0.35196064	0.549877713
Reep6	42	2.62417486	2.123855117	-0.500319742	0.35223905	0.550043192
Tnfrsf18	1	96.15384615	93.95604396	-2.197802198	0.35223293	0.550043192
Gm12338	3	91.41500849	94.30078084	2.885772349	0.35245718	0.550149626
Zbtb1	98	1.188015259	1.018389325	-0.169625934	0.35243267	0.550149626
Rmnd5b	49	1.399744944	1.335104027	-0.064640917	0.35241394	0.550149626
Atp5j	108	1.069727742	1.255343565	0.185615823	0.35234726	0.550149626
Dclk3	41	20.1678671	21.93039036	1.762523268	0.35249536	0.550150698
330041J22R	53	2.626921655	2.385126231	-0.241795424	0.35262466	0.550293982
Mycs	11	49.26788718	48.14188626	-1.126000913	0.35266438	0.550297436
Poc1b	49	1.496984372	1.350427434	-0.146556938	0.35278103	0.550393839
Ttc27	11	1.167347967	2.084804915	0.917456948	0.35280118	0.550393839
Socs7	115	1.404396304	1.394919755	-0.009476549	0.35287615	0.550452281
Fam20c	121	1.16861514	1.163797401	-0.00481774	0.3530289	0.550632024
700030C10F	45	72.08277469	72.95961163	0.876836945	0.35313533	0.55072648
Ogfr	24	0.950983989	0.826962218	-0.124021771	0.35316452	0.55072648
Adsl	32	1.252403214	1.149277849	-0.103125365	0.35332156	0.550912818

Tcf25	47	0.970952681	1.003152784	0.032200103	0.35352933	0.551178213
Nsmf	35	10.43240002	10.08694342	-0.345456608	0.35374377	0.551453952
Bmpr2	104	1.396723475	1.356169645	-0.04055383	0.35381845	0.551511788
Serinc5	25	1.97416018	1.507885088	-0.466275092	0.35394031	0.551525969
Tmem70	67	1.303229265	1.099042068	-0.204187198	0.35393006	0.551525969
Fuz	8	1.057469861	0.893099149	-0.164370712	0.35392662	0.551525969
Mir1903	8	1.398440312	1.760653508	0.362213196	0.35413686	0.551656499
Arid4a	49	1.413731949	1.573634049	0.1599021	0.35410528	0.551656499
Tspan12	27	4.309565838	3.608810012	-0.700755826	0.35406917	0.551656499
Cdkl2	19	0.823495196	0.39722534	-0.426269856	0.35425234	0.551777826
B2m	7	1.42086773	1.03030119	-0.39056654	0.35441059	0.551907146
Zfp410	39	1.413451572	1.018891752	-0.39455982	0.35437683	0.551907146
Ndufs7	3	6.431329782	4.240017792	-2.191311991	0.35473631	0.552355749
Nrbp2	6	79.65679516	80.54519785	0.888402688	0.35512564	0.552785996
Nrbf2	74	1.298152744	1.161736011	-0.136416733	0.35507308	0.552785996
Trmt12	23	1.892055508	1.713592864	-0.178462644	0.35511493	0.552785996
Zfp36l2	30	1.077731563	1.063203269	-0.014528294	0.35533565	0.552995581
Dcp1a	32	1.68440173	1.355425551	-0.328976179	0.35530548	0.552995581
Pik3ap1	34	6.576023745	7.242605568	0.666581823	0.35539086	0.553022849
Cep85	27	1.984124661	1.757428507	-0.226696154	0.35543948	0.553039868
Map3k11	24	1.675653092	1.427306699	-0.248346393	0.355602	0.553143323
Mak16	50	1.330124334	1.28800319	-0.042121144	0.35558489	0.553143323
Dnmt1	50	1.452904111	1.615132077	0.162227966	0.35565676	0.553143323
Mir1907	10	44.60368905	44.32478387	-0.278905183	0.3556228	0.553143323
Pcgf1	36	1.720586682	1.576286798	-0.144299884	0.35572507	0.553190931
Cog8	50	0.822656386	0.822594736	-6.16E-05	0.35603365	0.553526193
Rnu12	11	1.355937261	0.841914948	-0.514022313	0.35609154	0.553526193
Mettl9	66	1.826953917	1.468939424	-0.358014493	0.35609017	0.553526193
Tspan14	85	1.244632032	1.190253152	-0.054378881	0.35602037	0.553526193
Rpl5	59	1.283350098	1.227916985	-0.055433112	0.35617584	0.55359859
Dync1h1	50	1.426293033	0.976143755	-0.450149279	0.35633329	0.553784651
Frzb	9	10.4627077	9.150991679	-1.311716021	0.35640295	0.553834253
Cit	5	8.292613636	5.357213062	-2.935400575	0.35707725	0.554823338
Marcks	7	1.210653753	0.981425974	-0.229227779	0.35717194	0.554911705

Rbm4b	42	1.277030539	1.27812683	0.001096291	0.35726685	0.555000395
Fbxo21	38	1.448785442	1.260332614	-0.188452828	0.35731591	0.555017852
Bzw1	49	0.967504305	0.86598105	-0.101523256	0.35761621	0.555425518
Ube2l3	26	1.49720727	1.268218361	-0.228988909	0.35771909	0.555526508
A630020A06	10	3.017295481	3.062871609	0.045576129	0.35785676	0.555632483
Lin52	14	1.452204489	1.269898697	-0.182305792	0.35786306	0.555632483
Gpn2	23	1.655861102	1.398777805	-0.257083297	0.35793508	0.555685498
Polb	35	1.749245364	1.429387597	-0.319857768	0.35800951	0.555742253
Dnaaf2	26	1.55751497	1.225958833	-0.331556138	0.35827227	0.556088066
Fam65a	84	1.552038427	1.261567276	-0.290471151	0.35830807	0.556088066
Tnr	3	29.66101695	33.98969831	4.328681358	0.3584718	0.556228514
Cdk5rap3	27	3.080680118	3.331000264	0.250320146	0.35847438	0.556228514
Sh3bp4	72	1.648666497	1.476483789	-0.172182709	0.35854291	0.556256906
Rbfox2	56	1.328199423	0.894966459	-0.433232964	0.35856849	0.556256906
110008F13R	97	1.359066058	1.187465848	-0.17160021	0.35903597	0.556923237
Rnf44	145	2.134247945	1.732608627	-0.401639319	0.35928355	0.557126743
Parp14	15	1.010025063	1.013938524	0.003913461	0.35929024	0.557126743
Tpx2	10	1.819839861	1.770708368	-0.049131493	0.35929697	0.557126743
Gjd2	7	47.62052007	50.14880952	2.528289454	0.35931903	0.557126743
930584F24R	1	86.15384615	80.28169014	-5.872156013	0.35947061	0.557185145
Tdp1	35	0.692240562	0.977678848	0.285438285	0.35940306	0.557185145
Tmem260	56	1.376175974	1.076391833	-0.299784141	0.35946352	0.557185145
Tpr	2	4.573170732	2.3860799	-2.187090832	0.35968584	0.557342136
Fam57a	84	1.277436442	1.402734321	0.125297879	0.35964183	0.557342136
Gm6093	2	96.03174603	93.43418426	-2.597561769	0.35968517	0.557342136
Avpi1	8	2.033950935	2.261537866	0.227586931	0.35986293	0.557557662
Dut	26	1.667555811	1.097388769	-0.570167042	0.3602724	0.558133154
Mir6914	7	94.94233723	96.09524397	1.152906739	0.36050709	0.558437778
Trmt10c	33	1.239060042	0.933183333	-0.30587671	0.36063226	0.558513748
BC002163	11	27.04778554	27.46976308	0.421977543	0.36062208	0.558513748
Gm8439	1	27.52293578	22.11538462	-5.407551164	0.36087266	0.558827075
Tex10	92	0.99950502	0.984870385	-0.014634635	0.36102399	0.559002425
Kdelr2	74	1.086658718	1.02281643	-0.063842288	0.3613294	0.559416293
Commd1	18	1.591844213	1.673233488	0.081389275	0.36146589	0.55950954

Gstcd	33	0.981354533	1.032288324	0.050933791	0.36144233	0.55950954
Ptrhd1	32	1.643037301	1.072005849	-0.571031452	0.36161914	0.559628692
Ccl11	1	93.75	97.61904762	3.869047619	0.36160452	0.559628692
Mrpl30	11	1.449864815	1.554588695	0.10472388	0.3618072	0.559815953
Ptdss1	32	1.257740572	1.453973959	0.196233386	0.36185459	0.559815953
Nol12	34	1.37341098	1.252473448	-0.120937533	0.3618431	0.559815953
BC029722	24	1.218763674	1.472699734	0.25393606	0.36192805	0.559870571
Fam118b	25	1.438427984	1.472851462	0.034423478	0.36199102	0.559908945
Camsap3	14	1.284546833	1.192590005	-0.091956828	0.36203495	0.559917876
Dnajb11	40	1.252226445	1.114049841	-0.138176604	0.36215278	0.560041089
Rnase10	1	90.68322981	87.6344086	-3.048821212	0.36229831	0.560207104
Arcn1	31	1.138082208	0.881062315	-0.257019893	0.36241361	0.560267311
Ptbp1	4	1.734279077	0.841612864	-0.892666214	0.36239253	0.560267311
Ninj1	79	1.485024265	1.610164825	0.12514056	0.36250236	0.560345483
Trim23	7	0.926738166	0.456095107	-0.47064306	0.36284924	0.5608226
Plrg1	8	0.465726921	0.093283582	-0.372443339	0.36293816	0.560900949
Pabpn1	32	1.50478726	1.673676131	0.168888871	0.36297884	0.560904754
Tmem256	45	1.519859085	1.110828847	-0.409030239	0.36330235	0.561345545
Gm527	109	1.259678754	1.36001173	0.100332976	0.36352166	0.561566148
Sntb2	73	0.993918755	1.033307134	0.03938838	0.36350944	0.561566148
Mir1949	5	2.371262269	1.778265615	-0.592996654	0.36365238	0.56170895
Fam193b	56	1.838748983	1.569008978	-0.269740005	0.36373468	0.561776944
Mir130a	5	91.60862722	90.60486513	-1.003762088	0.36383483	0.561818462
Fam103a1	36	1.546500188	1.381126929	-0.165373259	0.36383814	0.561818462
Foxred1	25	1.10059719	0.92937353	-0.17122366	0.36394387	0.561890112
430037G07R	23	1.210256964	0.815051076	-0.395205888	0.36396112	0.561890112
Gatsl2	37	2.036752516	1.800699178	-0.236053338	0.36406884	0.561997282
Lrig2	50	1.383008051	1.414627768	0.031619717	0.36433069	0.562235748
300002I08R	5	3.573177748	3.033449673	-0.539728076	0.36429821	0.562235748
Tram1	20	1.899083215	1.191597516	-0.707485699	0.36433827	0.562235748
330006A16R	54	6.101296312	5.462790713	-0.6385056	0.36458401	0.562555812
Fam228b	5	3.376507722	3.604036715	0.227528993	0.36468412	0.56265112
Arpc5l	50	0.919968548	1.006715761	0.086747213	0.3648159	0.56277539
Fos	35	1.715126481	1.654149051	-0.06097743	0.36484137	0.56277539

Atg101	40	1.105046696	1.020806618	-0.084240078	0.36489333	0.562796374
Gm6644	24	0.896551732	0.679602931	-0.216948801	0.36500593	0.562804457
Kcnk3	70	10.07563166	10.42351701	0.347885347	0.36496058	0.562804457
Smdt1	49	1.601035862	1.576862863	-0.024172999	0.36501363	0.562804457
Cog4	12	2.807068184	2.929383715	0.122315531	0.36513478	0.562872963
Polm	24	1.445969288	1.104941597	-0.341027691	0.3651057	0.562872963
Kctd20	75	1.157584304	1.16570642	0.008122115	0.36526232	0.563010422
Srpk1	82	1.259849216	1.200396465	-0.059452751	0.36536102	0.563073488
Ly96	8	1.895858857	1.667803306	-0.228055551	0.36537997	0.563073488
Snrnp48	30	1.804035095	1.613636073	-0.190399022	0.36558317	0.563327463
Tmub1	26	1.871881685	1.814292359	-0.057589326	0.36603261	0.563960782
Lrrfip1	100	1.473006931	1.342098224	-0.130908708	0.3661712	0.564093323
Cnih2	55	2.797271533	3.13775336	0.340481827	0.36619552	0.564093323
Dlst	83	1.697198506	1.425410126	-0.27178838	0.36632997	0.564191319
Enox1	88	26.11535407	27.73828214	1.622928069	0.36633603	0.564191319
Vps39	17	1.905338986	1.308306023	-0.597032964	0.36652291	0.564242253
Dlx6os2	8	37.0901372	40.07675271	2.986615513	0.36648278	0.564242253
Uso1	63	1.580118572	1.107025357	-0.473093215	0.36646318	0.564242253
Zfp942	6	0.977686694	1.082558005	0.104871311	0.36649773	0.564242253
Dhx40	14	0.805740551	1.062195538	0.256454987	0.36679583	0.564603163
Sharpin	10	1.15398679	1.764466201	0.610479411	0.36687594	0.564638527
Cbx8	36	1.427225515	1.701352651	0.274127136	0.36689576	0.564638527
Zfp59	8	1.998373427	1.778938402	-0.219435025	0.36704247	0.564805077
Tgif2	27	1.688671943	1.64639644	-0.042275503	0.36723714	0.56501867
Exosc5	15	1.340600511	1.087353978	-0.253246533	0.36725828	0.56501867
Egf	6	16.09507004	17.09150802	0.996437986	0.36740078	0.565125133
Lrba	97	1.357011795	1.381976396	0.024964601	0.36742936	0.565125133
Crls1	90	1.034434154	1.201584709	0.167150555	0.36744302	0.565125133
Slc35e4	13	2.488244479	2.687473971	0.199229491	0.36751282	0.56517324
Glrx5	51	1.194562576	1.041662328	-0.152900248	0.36756894	0.565200317
Irx5	69	1.776169577	1.668731904	-0.107437673	0.36773318	0.565393621
Rexo2	41	1.292462415	1.185248021	-0.107214394	0.36788485	0.565533658
Kpna2	42	1.695423577	1.425327023	-0.270096554	0.36793988	0.565533658
Ston1	3	28.74129353	26.63690476	-2.10438877	0.36793905	0.565533658

Rev3l	72	1.335596226	1.353426384	0.017830158	0.36820804	0.565886543
Zfp524	40	1.254345639	1.03221569	-0.22212995	0.36827548	0.565930916
Rnf19b	89	0.923754168	0.803820075	-0.119934093	0.36835491	0.565993713
Hsd17b4	29	1.536291726	1.760497704	0.224205977	0.36864975	0.566387447
Trim52	3	91.43730887	89.13015873	-2.307150138	0.36877764	0.566524612
Rad9b	11	1.326092355	1.368457248	0.042364893	0.36908828	0.566942464
Mex3a	57	1.543216158	1.731892924	0.188676767	0.36917279	0.566953579
Mat2a	62	1.358555677	1.133029021	-0.225526656	0.36915765	0.566953579
Mpst	16	2.178698904	1.39213314	-0.786565764	0.36924615	0.567006903
Babam1	25	8.427425902	8.398958515	-0.028467387	0.3692885	0.567012604
Wbp2	3	3.307888041	4.090206359	0.782318318	0.36937081	0.567079644
Sdhaf2	27	1.837520573	1.70804433	-0.129476244	0.36959045	0.567357493
Vamp8	6	1.19047619	0.708941264	-0.481534926	0.36964601	0.567383428
Mir7672	51	1.340373165	1.538598037	0.198224872	0.36979743	0.567556485
Mir1249	1	90.45454545	92.70072993	2.246184472	0.36991371	0.567629398
Tmem222	54	1.295408666	1.317606067	0.022197401	0.36992231	0.567629398
Tubgcp2	44	1.64362717	1.304018298	-0.339608872	0.37000855	0.567643011
Snhg10	38	1.8393648	1.777023936	-0.062340864	0.36997491	0.567643011
Sod2	54	4.428093144	4.012417496	-0.415675648	0.37014567	0.567725442
Nln	12	1.659556727	1.93157381	0.272017082	0.37011029	0.567725442
Aggf1	54	0.920379504	1.069233549	0.148854045	0.37017834	0.567725442
Itfg3	15	1.804559713	1.266253018	-0.538306695	0.370424	0.568042816
30072M18I	64	1.040455204	1.001551852	-0.038903352	0.37051323	0.568120282
Wdr4	46	1.38364998	1.343292123	-0.040357856	0.37064865	0.568260256
Cul5	63	1.070683209	1.077657095	0.006973887	0.37068197	0.568260256
430038I01R	14	1.689426752	1.829161625	0.139734872	0.37085269	0.568462594
Abhd1	12	1.155000632	1.102653508	-0.052347124	0.37091937	0.568505413
Plek2	9	1.683543391	1.923850174	0.240306783	0.371334	0.569081476
Zfp652os	95	1.12735594	1.056432959	-0.070922981	0.37150834	0.569289203
'00007G11F	15	2.555631795	1.861072087	-0.694559707	0.37187932	0.569798183
Kiss1r	18	1.14341403	1.830665752	0.687251722	0.3720793	0.569985558
Emc4	22	1.399086477	1.264156138	-0.134930339	0.37206879	0.569985558
Mir698	6	88.86256521	86.93390889	-1.928656319	0.37212304	0.569993058
Tnfaip8l1	12	0.496991188	0.086805556	-0.410185633	0.37228567	0.570182645

Tspan9	63	1.94888316	1.632315518	-0.316567642	0.37243769	0.570355943
Dhx38	31	1.01792563	1.260702255	0.242776625	0.37251789	0.570419228
AI593442	2	33.11460184	30.21084822	-2.90375362	0.37284148	0.570594199
Gtpbp6	19	1.305587093	0.772238006	-0.533349087	0.37276627	0.570594199
Tor1aip2	57	1.030228523	0.782473937	-0.247754586	0.37294323	0.570594199
Mlst8	39	1.10096373	1.350418212	0.249454482	0.37292027	0.570594199
Rsg1	8	1.355970995	0.753894969	-0.602076026	0.37271876	0.570594199
Ptprr	10	27.33174942	24.71846688	-2.61328254	0.37281905	0.570594199
Fkbp5	90	1.052148193	0.887279811	-0.164868382	0.37293571	0.570594199
'00017D01F	2	96.94332887	94.66666667	-2.276662204	0.37287961	0.570594199
Rsph1	1	19.32773109	15.34090909	-3.986822002	0.37299705	0.57061705
Ankrd13c	109	1.567387759	1.432189988	-0.135197771	0.37305443	0.570645329
Mvb12a	37	1.051419929	0.901836655	-0.149583274	0.37322366	0.570844698
Slmap	68	1.734388362	1.342219256	-0.392169106	0.37341226	0.571073631
Rhbdd1	28	4.209201424	4.277435765	0.068234341	0.3734612	0.57108896
Nol8	9	0.666199024	0.920257527	0.254058503	0.37367549	0.571357103
)30458C11F	63	1.048905239	1.09515112	0.046245881	0.3737208	0.571366843
)31439G07F	14	1.380980983	1.52146096	0.140479977	0.37377783	0.571394509
Bpnt1	23	0.996694506	0.665063774	-0.331630732	0.3740115	0.571394557
Zfp719	2	3.50877193	1.694915254	-1.813856676	0.37391927	0.571394557
Polrmt	26	1.055938676	1.202986905	0.147048229	0.37397495	0.571394557
Adam9	38	1.183424684	1.214449092	0.031024408	0.37393842	0.571394557
Appbp2	87	0.875929151	0.903882264	0.027953113	0.37382441	0.571394557
Atf7	16	1.261164385	1.340960283	0.079795898	0.37388674	0.571394557
Becn2	4	79.56042679	78.82198081	-0.738445978	0.37427693	0.571740544
Txndc5	95	1.530118663	1.531915083	0.00179642	0.37444496	0.571937678
Slc25a26	25	1.309104546	1.274495373	-0.034609173	0.37488956	0.57241842
Espl1	20	1.677641766	1.666165448	-0.011476318	0.37481883	0.57241842
Dpp8	45	1.040511361	0.848252478	-0.192258883	0.3748506	0.57241842
Capza2	78	1.538761342	1.587102067	0.048340725	0.37491573	0.57241842
Nudt1	5	0.414764079	0.806019839	0.39125576	0.37504112	0.572490735
Slc39a7	23	1.754423697	1.469186903	-0.285236794	0.37501263	0.572490735
Ly6e	16	1.499302879	1.572257697	0.072954818	0.37511428	0.572542854
Smyd3	8	1.848735719	2.062345086	0.213609367	0.37538537	0.572784127

Acy1	21	1.483874546	1.831064819	0.347190273	0.37534387	0.572784127
Gm1976	4	2.093596059	2.476621859	0.383025799	0.37538946	0.572784127
Eif4a1	8	2.943817904	2.034197372	-0.909620533	0.37561013	0.573061238
Hnrnpk	136	1.441763507	1.421168181	-0.020595326	0.3757056	0.573147303
Lrrc75a	112	1.535146163	1.421281687	-0.113864476	0.37592913	0.573400028
Tsnax	83	1.75869282	1.423053653	-0.335639167	0.37594941	0.573400028
Scnn1g	18	4.827209214	6.325390888	1.498181674	0.37608416	0.573545935
Poc5	18	1.011475748	1.299761675	0.288285927	0.37621003	0.573678262
Ppp2r3a	52	1.233455339	1.025837439	-0.2076179	0.37654554	0.573803568
Grwd1	16	1.846640044	1.666893199	-0.179746845	0.37660048	0.573803568
Wwox	24	0.934742962	1.053684997	0.118942035	0.37642595	0.573803568
Luc7l2	15	2.133307669	1.574907977	-0.558399692	0.37647888	0.573803568
Eif2b1	20	0.99745431	0.642691204	-0.354763106	0.37650903	0.573803568
930007I19R	58	1.020431304	1.054435181	0.034003877	0.37660503	0.573803568
Sgtb	19	3.029343015	3.059108924	0.029765909	0.37638353	0.573803568
Camsap2	71	1.294168754	1.109700495	-0.184468259	0.37639645	0.573803568
Rrp9	18	0.907728254	0.64889608	-0.258832173	0.37664893	0.573810878
Pcnxl3	61	1.468735048	1.102635441	-0.366099607	0.37670651	0.573839028
Rdh14	40	1.062070608	0.986823236	-0.075247372	0.37686128	0.573938623
Dis3l	45	1.221006622	0.995530219	-0.225476403	0.37688923	0.573938623
Ndufa10	35	1.713247958	1.430333202	-0.282914756	0.3768343	0.573938623
Tmem11	40	0.968684602	0.803602065	-0.165082537	0.37704283	0.574112953
Bloc1s3	3	1.908157406	3.026530478	1.118373071	0.37709737	0.574136413
Rap1a	89	1.128152648	1.05546618	-0.072686468	0.37741056	0.574534316
Tial1	65	1.510098503	1.476201612	-0.033896891	0.37743702	0.574534316
Pcdhb1	7	56.03467608	53.61615274	-2.41852334	0.37782281	0.574953674
Dbr1	46	1.300048515	1.305243725	0.00519521	0.37783006	0.574953674
Dapk3	24	1.080514453	1.141044218	0.060529765	0.37776524	0.574953674
Lca5	37	1.103511645	1.067981429	-0.035530216	0.37792847	0.575043802
'00008O03F	3	22.59350483	22.61334965	0.019844811	0.3781624	0.575340082
Supt6	14	1.424315488	1.297470851	-0.126844637	0.37846724	0.575627964
Meaf6	83	1.524834205	1.264286192	-0.260548012	0.37841647	0.575627964
Snx22	9	21.94574236	22.29110259	0.345360225	0.37846931	0.575627964
Coq6	3	2.499463058	3.405000196	0.905537138	0.37879996	0.576070923

Dock7	93	1.037571072	1.111277596	0.073706524	0.37883906	0.576070923
Sppl3	133	0.984798862	1.017714564	0.032915702	0.37888092	0.576074868
Dus4l	18	1.227441795	1.029138405	-0.198303389	0.37895266	0.576124255
Sh3gl1	32	1.141853306	0.972612667	-0.16924064	0.37911884	0.5762575
Ssbp2	133	1.736682626	1.696339947	-0.040342678	0.37911862	0.5762575
Abi2	55	1.160863304	1.229562574	0.068699271	0.37971134	0.577098308
Fam35a	71	0.970864697	0.812556216	-0.158308481	0.37977673	0.577137911
Pnrc2	21	0.683186436	0.37878407	-0.304402366	0.37984039	0.577174882
Mettl2	42	1.663803822	1.427564748	-0.236239074	0.38035253	0.57786276
Gm13830	36	1.586024884	1.872095805	0.286070921	0.38041826	0.57786276
Rps5	34	1.373929652	1.185978081	-0.187951571	0.3804506	0.57786276
Tmem201	57	0.938527125	0.916554312	-0.021972813	0.38042791	0.57786276
Top1mt	6	3.040687806	3.200962116	0.16027431	0.38074491	0.578249928
Ndnl2	52	5.450644586	5.184972818	-0.265671768	0.38082713	0.578314938
920006O11R	10	1.095737762	0.688912784	-0.406824978	0.38096795	0.578468921
Nub1	21	1.393309218	1.397191749	0.003882531	0.38103047	0.578503989
Wdr43	61	2.869492218	2.95036599	0.080873772	0.38133597	0.578907932
Mbd3	59	1.108042919	1.070330239	-0.037712679	0.38137876	0.578913001
030030I06R	35	18.09993769	18.12176508	0.021827389	0.38155374	0.578982159
Usp21	30	1.809885879	1.963691209	0.15380533	0.38156988	0.578982159
Magoh	13	21.97601454	22.352231	0.376216467	0.38158215	0.578982159
Taf3	38	2.057362763	1.641223759	-0.416139004	0.38152789	0.578982159
Efr3a	76	0.791795979	0.898498646	0.106702667	0.38167104	0.579057155
Sct	14	9.66668845	9.747285548	0.080597098	0.38175504	0.579124724
Ufc1	8	1.791889217	1.944600563	0.152711346	0.38182847	0.579176243
U2af1l4	8	1.050964596	1.237884655	0.186920059	0.38197391	0.579336962
Zfp446	24	1.20559448	0.780375706	-0.425218773	0.38223954	0.579620032
Eif2s3x	12	1.783341242	1.543166563	-0.240174679	0.38222088	0.579620032
Arid1b	127	1.103062382	0.879594159	-0.223468223	0.38234397	0.579718474
Gcsh	39	12.16531948	11.46268803	-0.702631455	0.382513	0.579783104
Epdr1	6	2.19047619	1.880848494	-0.309627696	0.38253768	0.579783104
Egr1	39	1.925109441	1.820062727	-0.105046714	0.38242645	0.579783104
Strada	29	1.36184039	1.239710077	-0.122130313	0.38254464	0.579783104
Vegfb	78	1.456349117	1.548499208	0.092150092	0.38272609	0.579998201

Nudcd3	20	1.386579472	1.187961968	-0.198617504	0.38305035	0.58042966
Fancl	22	1.12237781	1.268600508	0.146222699	0.38333307	0.580798091
Spg11	29	2.043028616	1.51842113	-0.524607486	0.38358311	0.581116921
Ccnl1	117	1.24233338	1.252734202	0.010400822	0.38369368	0.581224425
Mir5106	1	94.59459459	96.7032967	2.108702109	0.38389449	0.581468595
Lyrm9	38	1.346609868	1.142005045	-0.204604823	0.38417836	0.5818385
Abtb1	70	1.349478643	1.230932656	-0.118545987	0.38453401	0.582317033
Zcchc8	56	1.180568624	1.003053714	-0.17751491	0.38462785	0.582399037
Cdk19	12	2.293102965	2.021774497	-0.271328468	0.384767	0.582489531
430018J23R	2	2.257243259	4.383561644	2.126318385	0.38472734	0.582489531
Stxbp4	22	1.403556883	1.281854642	-0.121702242	0.38540128	0.583389552
Iqce	70	1.16422385	1.245136445	0.080912595	0.3855338	0.58352996
Tada2a	59	1.0574265	1.191465214	0.134038714	0.38558239	0.583543319
Lman1	49	1.402594491	1.194684244	-0.207910247	0.38572472	0.583698358
Txndc12	8	1.732295106	1.43121521	-0.301079896	0.38576439	0.583698358
Lsm2	13	0.7951647	0.538288404	-0.256876296	0.38589275	0.583832377
Rps25	12	1.018009768	0.986489045	-0.031520723	0.38645847	0.584628001
Zbtb41	67	0.916890405	1.207455958	0.290565553	0.38654052	0.584691851
Zbtb21	34	0.800411278	0.750205162	-0.050206116	0.38660059	0.584722432
Slc19a2	69	1.520380739	1.653792269	0.13341153	0.38706462	0.585363937
Pygo2	60	1.327469037	1.320373703	-0.007095334	0.38720084	0.585509595
Ndufaf1	6	3.835252846	4.862060302	1.026807455	0.38752911	0.585945613
Klhl22	43	1.114626502	1.030718858	-0.083907644	0.38759336	0.585982385
Rufy1	67	0.928247932	0.925884327	-0.002363605	0.38773421	0.586134932
Tmem194	38	0.796668677	0.797594057	0.00092538	0.3878337	0.58622494
Mtag2	51	2.91684912	2.951388835	0.034539716	0.38798995	0.586400708
Sarm1	31	10.5698513	10.06781072	-0.502040577	0.38812688	0.586497032
Kctd21	21	1.418553232	1.706105834	0.287552602	0.38813362	0.586497032
Dedd	49	1.389927513	1.555848168	0.165920656	0.38838022	0.586628027
Syngr4	10	1.596121101	0.784611063	-0.811510039	0.38836236	0.586628027
Rab5c	15	1.092129087	0.958802687	-0.1333264	0.38835247	0.586628027
Myo9b	39	1.023022881	0.818125788	-0.204897093	0.38833628	0.586628027
Hoxb6	7	8.961941518	9.208177267	0.246235749	0.38846105	0.586689727
Dido1	60	1.884020244	2.161624746	0.277604503	0.3886178	0.586866072

Pag1	34	2.586116741	2.829821583	0.243704842	0.38868427	0.586906045
Ipo13	67	1.508111502	1.525498276	0.017386774	0.38886564	0.587036903
Csnk2a1	13	1.42131631	0.991243688	-0.430072622	0.38892844	0.587036903
Tcp1	23	1.313009333	0.968888428	-0.344120905	0.38901096	0.587036903
Sema6d	99	15.63600247	16.25830201	0.622299548	0.38897739	0.587036903
Smad2	184	1.076688324	1.048982805	-0.027705519	0.38894105	0.587036903
Txnrd2	34	1.038479511	1.155606963	0.117127451	0.38892172	0.587036903
Fev	16	12.63506197	13.12526989	0.490207919	0.38910437	0.587117491
310408A11R	42	1.287258913	1.678262574	0.391003661	0.38920469	0.587208479
Pqlc2	28	1.875354767	1.901581014	0.026226247	0.38958672	0.587631751
Ube2m	72	1.201312807	1.008875136	-0.192437671	0.38968547	0.587631751
Tm9sf3	100	1.367921372	1.277920717	-0.090000655	0.38968162	0.587631751
Myeov2	6	0.28038751	0.816200267	0.535812757	0.38961009	0.587631751
Oas1c	7	2.201032213	1.742398856	-0.458633357	0.38959139	0.587631751
Dph1	12	2.528471151	1.676050761	-0.852420391	0.39002159	0.588017756
Sec24a	39	5.7703066	4.948249563	-0.822057037	0.38998753	0.588017756
Npl	4	11.31835771	13.38170427	2.063346559	0.39038164	0.58850012
Plag1	23	1.176070293	1.205043884	0.028973591	0.39050243	0.588621736
Mrps7	45	0.815668943	1.028233092	0.212564149	0.39074057	0.588719904
Sec61a2	5	0.333354026	0.733570131	0.400216105	0.39076815	0.588719904
Vamp3	65	1.018670327	0.88780207	-0.130868257	0.3907376	0.588719904
Nudc	8	0.213675214	1	0.786324786	0.39070694	0.588719904
Tpk1	36	1.343011531	1.115747396	-0.227264135	0.39061389	0.588719904
Atp1a3	33	11.57591533	12.59285693	1.016941594	0.39101253	0.589027594
Itgb7	1	7.352941176	9.826589595	2.473648419	0.39143415	0.589602202
Stub1	58	1.578014412	1.392958156	-0.185056256	0.39150396	0.589646832
Mrpl34	21	1.822405156	1.473399688	-0.349005468	0.39163205	0.589779213
Hipk3	102	1.419011842	1.376466529	-0.042545313	0.39203691	0.590328338
430033K04R	6	2.951883089	2.768929194	-0.182953894	0.39211722	0.590388688
933404O12R	27	1.449109561	1.286058742	-0.163050819	0.39225022	0.590528338
Zik1	8	1.62477876	0.86447024	-0.76030852	0.39259934	0.590811479
Dctpp1	17	0.850112187	1.36713645	0.517024263	0.39256127	0.590811479
Styxl1	37	1.895648248	2.132979476	0.237331228	0.39248201	0.590811479
Edem3	65	1.318125358	1.417029783	0.098904425	0.3925321	0.590811479

Twf2	45	1.236574358	1.193109257	-0.043465101	0.39270301	0.590906893
Pde4b	3	1.703163017	3.205128205	1.501965188	0.39282632	0.591031825
Degs1	52	1.449463568	1.085463577	-0.363999991	0.39287122	0.591038787
Rpl34	20	0.955312418	0.797573594	-0.157738824	0.39304223	0.59105366
Map3k6	42	1.382445992	1.107043062	-0.27540293	0.3929981	0.59105366
Fam13b	112	1.098617384	1.001429023	-0.097188361	0.39303087	0.59105366
Crebl2	29	1.208874883	1.066837308	-0.142037575	0.39297408	0.59105366
Sars2	24	1.439442977	1.549241858	0.109798881	0.39311152	0.591097282
Las1l	11	23.2352765	25.03043297	1.795156472	0.39323122	0.591216689
Osbpl5	29	2.575256307	2.1467639	-0.428492408	0.39333777	0.591316298
Trmt11	35	1.5562932	1.43346411	-0.12282909	0.3935079	0.59151147
Cnot10	30	1.259854024	1.313652743	0.05379872	0.39366364	0.591684953
OC10050349	8	1.355390641	1.986274814	0.630884173	0.39375977	0.591708225
Acot7	82	1.286313204	1.24386386	-0.042449344	0.39374041	0.591708225
Ap5m1	56	1.05034921	1.047611074	-0.002738137	0.39398477	0.591985711
Agps	69	1.21773361	0.849825447	-0.367908164	0.39414025	0.592158695
Slc39a9	20	1.726566314	1.831036873	0.104470559	0.39438633	0.592467751
930024E05R	24	1.250572135	1.089425248	-0.161146887	0.39451219	0.592596154
430046D13R	11	0.69842223	0.529436322	-0.168985908	0.39469692	0.592812964
Gpank1	21	1.084461228	0.943137921	-0.141323308	0.39478906	0.592890669
900076A07R	21	0.712516506	1.219737427	0.507220921	0.39508245	0.593218436
Creld2	77	1.528797518	1.589832886	0.061035368	0.39508817	0.593218436
Aldoc	3	91.83230826	91.0285144	-0.803793854	0.39517063	0.593281542
Etfdh	15	1.138085965	1.293738127	0.155652162	0.39535138	0.593370785
Actr8	13	1.676887543	1.854471254	0.177583711	0.39530561	0.593370785
Arid2	208	1.277321312	1.212831396	-0.064489916	0.39534854	0.593370785
Zfp869	12	2.027267463	1.471085792	-0.556181671	0.39540601	0.593392088
Pigq	49	1.222838619	1.201272466	-0.021566153	0.39546291	0.593416784
Cdc42	83	1.570647012	1.303472809	-0.267174203	0.39550801	0.593423774
Mrpl27	8	2.15512801	2.314081325	0.158953315	0.39557999	0.593471092
Hp1bp3	161	1.284106499	1.166637451	-0.117469049	0.39564872	0.593513525
Ldlrad4	107	1.663111559	1.417102938	-0.246008622	0.39580897	0.593637691
Rpl38	42	1.462849026	1.537928101	0.075079075	0.39583858	0.593637691
R3hdm1	27	1.010880313	1.238541246	0.227660933	0.39585286	0.593637691

Spint2	45	1.624987056	1.312250758	-0.312736298	0.39590444	0.593654381
Msantd2	112	1.362053053	1.469925438	0.107872385	0.39599865	0.593734974
Stard13	9	3.629730485	3.129988964	-0.499741521	0.39605183	0.593754039
Jdp2	79	1.481808537	1.477749711	-0.004058826	0.3962715	0.593961995
Ap3s1	80	0.957851396	1.044026732	0.086175335	0.39623437	0.593961995
Tgoln2	8	0.501013856	1.107346134	0.606332278	0.39642027	0.594124298
Tulp3	6	1.876601486	0.822555507	-1.054045978	0.3967239	0.594457925
Lonp1	28	1.770927813	1.117071119	-0.653856694	0.39671333	0.594457925
Tbc1d8	71	1.468164058	1.321017146	-0.147146913	0.39684426	0.594577568
Rwdd4a	50	1.312510285	1.419776265	0.10726598	0.39704005	0.594810177
Coa7	15	1.73145193	1.392218353	-0.339233577	0.39733368	0.595128557
Vps37d	57	2.184674041	2.328259097	0.143585056	0.39732954	0.595128557
Supt20	16	0.891493056	0.873387372	-0.018105683	0.39746799	0.595268959
Plekhh2	61	1.626523267	1.564166161	-0.062357106	0.39751029	0.595271554
Ccndbp1	25	1.526068321	1.403110352	-0.122957969	0.39765684	0.595430253
Pitrm1	4	4.339774623	4.318186839	-0.021587784	0.3976989	0.595432476
Stpg1	16	54.36338266	51.9931435	-2.370239166	0.39785064	0.595598881
Ehbp1	19	1.592296098	1.279525488	-0.31277061	0.39796048	0.59560824
Prpf38b	59	1.23834481	1.062521603	-0.175823207	0.39794445	0.59560824
Hcar2	8	91.68122841	91.88687204	0.205643633	0.39797866	0.59560824
Rab21	82	1.534664159	1.467497551	-0.067166608	0.39806725	0.595619333
Mir7655	32	1.199731294	1.480872617	0.281141323	0.39805038	0.595619333
Nisch	12	0.520639057	0.918549064	0.397910007	0.39814271	0.595671505
Tigar	43	1.691391217	1.446220453	-0.245170763	0.39831408	0.595867138
Mcm10	23	2.108901052	1.523012824	-0.585888228	0.39842144	0.595936592
Tslp	4	11.84517497	11.49294405	-0.352230927	0.39844173	0.595936592
Kat5	30	2.079933922	1.664452306	-0.415481616	0.39849423	0.595954375
Rbm5	9	1.457487072	1.276226417	-0.181260654	0.3986727	0.596099776
Itpripl1	19	2.038475802	1.07306068	-0.965415122	0.39866493	0.596099776
.10040N11F	15	0.754464226	0.991505922	0.237041696	0.39883104	0.596275764
Gabarap	23	1.236468073	1.242674912	0.006206839	0.39894826	0.596390257
Gulo	2	4.268292683	4.117647059	-0.150645624	0.39901969	0.59643628
Anxa4	24	1.515949343	1.291336915	-0.224612428	0.3991624	0.596588827
Masp1	3	9.852052709	10.75074019	0.898687477	0.39924619	0.596653284

Cml1	8	1.740797653	2.179974706	0.439177053	0.39942878	0.596865364
Zfp598	73	1.22912652	0.899586888	-0.329539632	0.39968899	0.597193378
Mir7677	13	90.19894044	90.26613315	0.067192713	0.4002061	0.597905148
Slc19a1	11	3.103764322	2.858360879	-0.245403443	0.40032841	0.598026984
Ptk2b	16	2.548713108	2.301539557	-0.247173552	0.40050046	0.598223098
Tmem42	65	1.088113456	0.961976219	-0.126137238	0.40060711	0.598321504
Tomm7	5	0.853174603	1.267025447	0.413850844	0.40068142	0.598371584
Dpy30	2	0	0.833333333	0.833333333	0.40088091	0.598608573
Faf1	82	1.1185137	1.199699016	0.081185316	0.40103484	0.598777498
Stkld1	9	2.138078978	1.527508362	-0.610570616	0.40112648	0.598853392
Dnaja2	98	1.213993243	1.206487411	-0.007505832	0.40121417	0.598915799
Hspbap1	35	0.985432634	1.130237213	0.144804579	0.40124991	0.598915799
Lamp1	47	1.384597413	1.398177675	0.013580261	0.40133277	0.59897855
Mrps25	16	1.283987606	1.260915399	-0.023072207	0.40164419	0.599382371
Spink10	4	1.767676768	0.531914894	-1.235761874	0.40174488	0.599428459
Fbxl4	49	1.089559476	1.093313805	0.003754328	0.40177792	0.599428459
Ube2v1	86	1.224573932	1.248369823	0.02379589	0.4018235	0.599428459
Specc1l	62	1.156890158	1.159185589	0.002295431	0.40183847	0.599428459
Zfp846	17	1.382870757	1.051789599	-0.331081157	0.4024962	0.600348577
330009J07R	76	1.370314005	1.341313155	-0.02900085	0.40274621	0.600599386
Cenpa	26	1.528113195	1.349498332	-0.178614863	0.40273955	0.600599386
Amdhd2	22	0.879820646	1.053865415	0.174044769	0.40298188	0.600825628
Ppme1	31	1.42860306	1.658761044	0.230157984	0.40299384	0.600825628
Lmln	67	1.221389226	1.067315125	-0.154074101	0.40302076	0.600825628
Gm10548	14	1.403144757	1.253836264	-0.149308493	0.40311189	0.600900434
Lrrc32	1	17.17171717	21.97802198	4.806304806	0.40342247	0.601119142
Pcsk4	8	5.194339252	4.440360142	-0.75397911	0.40333754	0.601119142
700027F06F	2	47.85714286	42.89215686	-4.964985994	0.40338746	0.601119142
Ankrd10	76	1.810890625	1.57110003	-0.239790595	0.40339882	0.601119142
Sra1	19	2.112102079	1.728121017	-0.383981062	0.40377577	0.601584496
Usp25	71	0.969351606	0.971729404	0.002377798	0.40399792	0.601792119
700029J07R	11	2.150169688	1.529588712	-0.620580977	0.40412018	0.601792119
Susd6	63	1.174909751	1.112214861	-0.06269489	0.40410897	0.601792119
Usp12	124	1.108605842	1.163954822	0.055348979	0.40404591	0.601792119

Dnajc12	12	3.102617666	2.096864686	-1.005752979	0.40410209	0.601792119
Numbl	66	1.724961124	1.632226578	-0.092734546	0.40437176	0.602044565
Gm4285	60	1.054079828	1.096720168	0.04264034	0.40435389	0.602044565
Etv4	12	1.502976233	1.592955396	0.089979163	0.40453762	0.602230402
Pprc1	67	1.54700005	1.353435257	-0.193564793	0.4048094	0.602512745
Unc5b	82	1.147371297	1.043136798	-0.104234499	0.40479143	0.602512745
Prpf40a	66	1.593185967	1.489933357	-0.10325261	0.40486372	0.602532482
Fbxw8	50	1.177421226	1.176177345	-0.001243881	0.40496614	0.602623789
3-1190005i(	4	0.390625	0	-0.390625	0.40509334	0.602751946
Slamf8	2	9.674266274	11.30415162	1.629885351	0.40524476	0.602916105
Ppia	86	1.120880708	1.095741907	-0.025138801	0.40536841	0.603038936
Ccnb2	32	1.120407771	1.523982914	0.403575143	0.40541639	0.603049177
Nfrkb	55	1.284692304	1.138150587	-0.146541717	0.4056666	0.603360202
Snapc3	45	1.299039513	1.35428768	0.055248167	0.40575284	0.603400016
Dld	30	1.291265882	1.599860976	0.308595094	0.40581673	0.603400016
Fzr1	43	1.518585642	1.210517527	-0.308068116	0.40579581	0.603400016
Zfp451	71	0.986735374	1.138524131	0.151788757	0.40603912	0.603669503
Prpf6	34	1.538889039	1.241495108	-0.297393931	0.40617506	0.603688122
Smpd3	27	0.9998206	0.728904148	-0.270916451	0.40616897	0.603688122
Rpap3	25	1.633674389	1.408252639	-0.22542175	0.40614344	0.603688122
Skap2	43	1.332616858	1.519564106	0.186947248	0.40623929	0.603722435
Fdxr	3	0.82815735	0.199600798	-0.628556551	0.40633543	0.603804164
Hnrnpa2b1	81	1.28850123	1.112034589	-0.176466641	0.40646411	0.603811947
Aaas	28	1.4541176	1.446298892	-0.007818708	0.40639562	0.603811947
Fam49b	107	1.42201754	1.246536189	-0.175481351	0.40645275	0.603811947
Map3k3	95	1.262923421	1.237786255	-0.025137166	0.40656257	0.603897068
Bcl2l11	166	1.527763837	1.31416516	-0.213598676	0.40665665	0.603974831
330415F09F	35	1.236387626	1.066991318	-0.169396308	0.40669724	0.603974831
Platr8	6	87.9405041	85.87948918	-2.061014924	0.40723569	0.604713261
Mir7219	1	97.5	95.19230769	-2.307692308	0.40741961	0.604828606
Wdr27	25	1.442761889	1.446238378	0.003476489	0.40743702	0.604828606
Slc25a51	36	0.867655865	0.951152817	0.083496952	0.40743004	0.604828606
Uqcr11	10	1.468355349	1.490069801	0.021714452	0.40774414	0.604978512
Trappc3	31	3.039307572	2.977959892	-0.06134768	0.40766637	0.604978512

Dars	22	0.731085647	0.88396946	0.152883813	0.40770299	0.604978512
Rbpms	80	1.378564626	1.100819387	-0.27774524	0.40773146	0.604978512
Preb	45	2.577261201	2.697290874	0.120029674	0.40760802	0.604978512
C2cd5	47	1.366959302	1.329467728	-0.037491574	0.40793412	0.605199205
Zbtb11os1	46	1.298163649	1.401126499	0.102962849	0.40800468	0.605242693
Syvn1	70	1.158902979	1.139105641	-0.019797338	0.40819242	0.60539878
333439L19F	32	1.649053316	1.28171179	-0.367341526	0.40815391	0.60539878
Zfp862-ps	7	2.048820966	1.673332424	-0.375488542	0.40831456	0.605518732
Snx12	2	25.11995571	22.66003707	-2.45991864	0.40860241	0.605838414
Lrrc48	7	3.405808884	2.519011586	-0.886797298	0.4086127	0.605838414
Mbd1	42	1.101329487	1.391751338	0.29042185	0.40872653	0.605945966
Pnisr	7	2.253979159	1.716875273	-0.537103886	0.40879155	0.605981122
Tbc1d10a	30	1.556647356	0.933510977	-0.62313638	0.40893898	0.606138451
Sgsm2	108	1.174376381	1.21664847	0.042272089	0.40911735	0.606341581
Pom121	84	4.942210828	4.89643104	-0.045779789	0.4092073	0.606413641
Kdm5b	44	1.813690353	1.683272434	-0.130417919	0.40926499	0.606437895
Mettl4	10	1.789372762	1.282217273	-0.507155489	0.40945061	0.60659044
'00099C18F	10	1.789372762	1.282217273	-0.507155489	0.40945061	0.60659044
Zfp322a	51	1.11537464	1.077677241	-0.037697399	0.40983248	0.606972333
Glcci1	77	0.985646918	0.766746235	-0.218900683	0.40981816	0.606972333
Desi1	31	1.839587222	1.252210649	-0.587376573	0.40978318	0.606972333
Zfp57	8	1.470972722	1.705259263	0.234286541	0.40994817	0.6070824
230070E04F	6	16.49184149	18.63734679	2.1455053	0.41015586	0.607326872
Gtf3a	12	1.144271774	0.705656264	-0.438615509	0.41019604	0.607326872
Tm2d1	39	1.565424998	1.613840968	0.04841597	0.41024258	0.607334498
Setd3	106	1.072033215	0.979267559	-0.092765657	0.41055759	0.607739538
Cdr2l	62	1.905986659	1.888366932	-0.017619727	0.41069395	0.607880076
)31425E22F	12	1.183047649	1.262942948	0.079895299	0.41083748	0.608031187
Sqle	65	1.142024105	1.210647338	0.068623233	0.41095703	0.608118486
Ctdp1	87	1.110709788	1.048565291	-0.062144497	0.41097935	0.608118486
Zdhhc4	42	1.90263428	1.70304979	-0.19958449	0.41102536	0.608125236
Eya3	28	1.300679529	1.154668175	-0.146011355	0.41114639	0.608136725
Pfn3	4	43.85157676	43.41309752	-0.438479249	0.41114913	0.608136725
Mir6391	9	92.1910448	93.50382138	1.312776574	0.41115745	0.608136725

Cpsf6	57	1.033217128	0.780733175	-0.252483953	0.41151898	0.608610104
Rbks	47	1.093244295	0.979752696	-0.1134916	0.4115993	0.608667556
Smad1	73	1.234921685	1.422102401	0.187180716	0.4116721	0.608713858
Acbd3	60	1.16466633	0.804085401	-0.360580929	0.41202362	0.609033242
Kpna4	112	1.186244998	1.056221698	-0.1300233	0.41204285	0.609033242
230112J17R	4	16.69588431	15.42536898	-1.270515336	0.41197626	0.609033242
Chd4	77	1.836725889	1.439964291	-0.396761598	0.41205411	0.609033242
Pcbd1	5	0.888888889	0.753052917	-0.135835972	0.412228	0.609228891
Plekhg2	44	1.412688329	1.424838724	0.012150395	0.41242519	0.609458938
Rnf223	2	96.67967226	97.98316498	1.303492724	0.41261851	0.609639907
Rock2	115	1.056941249	1.032320807	-0.024620442	0.41263075	0.609639907
Ubtd2	95	1.840763053	1.886538819	0.045775766	0.41286957	0.60993135
Gpatch4	21	1.225437032	1.207306769	-0.018130263	0.41313732	0.610172342
Mut	9	0.961587335	0.729912316	-0.231675019	0.41315745	0.610172342
Pvt1	13	0.871799343	0.712120241	-0.159679103	0.41310317	0.610172342
Zbed4	135	1.191024811	1.280241178	0.089216367	0.41331319	0.610340923
Zfp212	32	1.138243077	0.983003287	-0.15523979	0.41340098	0.610362756
Decr1	21	1.646585357	1.326102172	-0.320483185	0.41341117	0.610362756
Mapre3	46	1.90629244	1.921416068	0.015123628	0.41382426	0.610905326
Prorsd1	21	2.975312187	2.626775801	-0.348536386	0.41386192	0.610905326
Phykpl	20	1.128211391	0.769782603	-0.358428789	0.41390375	0.610905612
Peak1	56	1.379119892	1.070404466	-0.308715426	0.41424044	0.611279581
Tnfrsf10b	16	0.814329802	0.978719652	0.16438985	0.41423596	0.611279581
Pomt1	26	1.258303636	0.920513247	-0.337790389	0.41433478	0.611357319
Brpf1	53	1.156503814	1.130164813	-0.026339	0.41456882	0.611641155
Rif1	84	1.161100187	1.188533142	0.027432955	0.41484685	0.611989812
Ints12	37	1.072210253	1.129605553	0.0573953	0.41511913	0.612329922
Smap1	125	1.027282666	1.019304126	-0.007978539	0.41532266	0.612568569
Spopl	44	1.499069855	1.430454494	-0.068615361	0.41539669	0.612616177
Hmgxb4	73	1.171249035	1.166521038	-0.004727997	0.41580841	0.613161755
)30037D09F	25	2.075910993	1.794263855	-0.281647138	0.41585578	0.613169984
Htr3a	5	4.364241803	5.197108107	0.832866304	0.41592523	0.613210771
Prmt5	10	0.706333544	0.76167599	0.055342446	0.41604792	0.613330034
Snx21	76	1.337581811	1.213669738	-0.123912073	0.41619059	0.613478719

Clmn	50	1.596028703	1.637102424	0.041073721	0.41628285	0.613553083
Neat1	20	1.450806097	1.870587727	0.41978163	0.41671335	0.614125908
Dtx3	35	2.400284431	2.36562942	-0.034655011	0.41685612	0.614263209
Ewsr1	43	0.930487178	0.966375528	0.035888351	0.41689023	0.614263209
Rhoa	30	1.779820347	1.425293275	-0.354527072	0.41700936	0.614377048
Hnrnpc	12	1.873032414	1.087457157	-0.785575257	0.41718981	0.614581194
310011J03R	16	0.988954053	1.428379046	0.439424993	0.41740564	0.614837418
Immp2l	42	1.387013386	1.140268828	-0.246744559	0.41750653	0.61486259
Sik3	98	1.316451661	1.505572059	0.189120398	0.41750007	0.61486259
Urgcp	78	1.361271588	1.405596041	0.044324453	0.41781675	0.615195964
Nfx1	52	1.055027923	1.081955771	0.026927848	0.41778085	0.615195964
Gm10421	6	60.00199243	60.6069479	0.604955474	0.41805495	0.615278417
Polr1d	30	1.520475376	1.579788094	0.059312717	0.41801201	0.615278417
Hist1h1b	30	1.515968422	1.617932245	0.101963823	0.41804792	0.615278417
Micu2	46	3.34231571	3.386760481	0.044444772	0.41806495	0.615278417
Ptp4a2	123	1.263077796	1.043074961	-0.220002835	0.4180824	0.615278417
330016D10F	74	1.161856192	1.140552481	-0.021303711	0.41834786	0.615607353
C1qbp	26	1.26313818	1.587533656	0.324395477	0.41839613	0.615616652
Slc33a1	38	1.200227785	0.983900991	-0.216326794	0.41867876	0.615970743
Fam160a2	36	0.82512552	0.763238568	-0.061886952	0.41873683	0.615994404
Dnali1	15	3.710443406	3.030754729	-0.679688677	0.41895206	0.616230754
Znf512b	77	1.427905432	1.293244813	-0.134660619	0.41898148	0.616230754
Sirt3	52	1.489641059	1.251406756	-0.238234303	0.41910474	0.616285053
Zfpl1	19	1.020413495	1.098186057	0.077772563	0.41914439	0.616285053
Prlhr	2	70.31479667	73.03161048	2.716813812	0.41912704	0.616285053
Cmtm8	54	6.08959042	7.005004765	0.915414345	0.41918958	0.616289737
Otx2	26	45.98456997	45.59013559	-0.394434382	0.4192906	0.616376497
Pank4	64	1.127445043	1.065478347	-0.061966697	0.41942697	0.616505186
Nfya	58	1.259682285	1.104135807	-0.155546477	0.41946216	0.616505186
Emc7	36	1.29321819	1.329405052	0.036186862	0.41960723	0.616656635
Lmbr1l	46	1.491442435	1.427868942	-0.063573492	0.4196979	0.616728124
Caprin1	80	1.005189674	1.163113462	0.157923788	0.41974226	0.616731538
Gm16062	3	3.597342425	2.057343623	-1.539998802	0.4198587	0.616840863
Dmtf1	71	1.290089465	1.3702039	0.080114435	0.42011486	0.61715541

Disp2	9	1.731657697	1.803792752	0.072135055	0.42044166	0.617573666
Trp53inp1	57	1.160070494	1.398591004	0.23852051	0.4205091	0.6176109
Mppe1	37	1.408115799	1.015118157	-0.392997641	0.42071224	0.617785591
Fam98c	44	1.391064658	1.371676271	-0.019388387	0.42068852	0.617785591
Nrp2	39	1.577705007	1.387887583	-0.189817425	0.42080902	0.617865875
Zfp281	88	1.054611323	0.990079438	-0.064531885	0.42104993	0.61815775
Fdps	10	2.26846825	2.146691211	-0.121777039	0.4213065	0.61847255
Slc12a4	84	1.208944854	1.187070455	-0.0218744	0.42141214	0.618499686
Tmem126b	22	1.354602168	1.622437849	0.26783568	0.4214283	0.618499686
Sgms1	98	1.297268073	1.386437046	0.089168973	0.42145143	0.618499686
Arpc1a	35	1.720999032	1.517360789	-0.203638242	0.42152348	0.618503
Arf1	65	0.974663543	0.905414359	-0.069249185	0.42153799	0.618503
Cers5	49	1.188899571	1.105712915	-0.083186656	0.42177806	0.618793371
Edem2	18	1.691985483	1.724335031	0.032349548	0.42191728	0.618935735
Tmem116	1	7.717041801	6.08974359	-1.627298211	0.42256249	0.619732195
Mir5625	18	1.957038004	2.272757256	0.315719251	0.42258691	0.619732195
Zbtb49	23	1.122938127	1.293557967	0.170619841	0.42251484	0.619732195
Adam18	2	77.52293578	78.13189057	0.608954788	0.42271223	0.619854031
Nrd1	39	1.625520274	1.381994205	-0.243526069	0.4229602	0.620155676
Ryk	87	1.141183359	1.080854976	-0.060328384	0.42304669	0.620220513
.10043O21F	56	1.552978761	1.811904015	0.258925254	0.42319995	0.620331204
Asna1	15	2.315986844	2.384412611	0.068425767	0.4232378	0.620331204
Gprc5a	19	26.23343201	25.60401943	-0.629412572	0.42324901	0.620331204
Rasgef1c	72	5.632427318	5.20976595	-0.422661367	0.42346472	0.620399496
Btbd1	80	1.122076443	1.154432801	0.032356358	0.42339419	0.620399496
Vimp	35	1.188083813	1.397436279	0.209352466	0.42345643	0.620399496
Rasa1	62	1.441910761	1.373042366	-0.068868395	0.42335573	0.620399496
Mfsd3	14	1.498264925	2.207779505	0.709514581	0.42378577	0.620457751
Mrpl53	25	1.573110691	1.440360073	-0.132750618	0.42380036	0.620457751
Mir22hg	14	1.643136112	1.394912114	-0.248223998	0.42384275	0.620457751
Pcnx	73	1.462037378	1.587269889	0.125232511	0.4237417	0.620457751
Nenf	37	1.703383898	1.096182884	-0.607201015	0.42371525	0.620457751
Mir345	10	1.490348101	0.832225593	-0.658122508	0.42376134	0.620457751
Cox7c	21	1.773975844	1.883719424	0.109743581	0.42383176	0.620457751

Fance	38	1.454267224	1.719730546	0.265463321	0.42368121	0.620457751
Rhog	75	1.494872042	1.314114853	-0.180757188	0.42400741	0.620513526
Dok1	9	3.38643052	2.659816131	-0.726614389	0.4240252	0.620513526
Loxl3	9	3.38643052	2.659816131	-0.726614389	0.4240252	0.620513526
Tsn	36	0.697138161	0.653034917	-0.044103244	0.42405	0.620513526
Zfp362	189	1.739081284	1.537903706	-0.201177578	0.42422309	0.620704916
Sema5b	62	24.80786984	26.11119953	1.303329694	0.4244457	0.620968712
Pygb	9	2.674064452	2.884380615	0.210316162	0.42465671	0.621207478
Trp53rkb	26	26.41987866	26.79597193	0.376093263	0.42469357	0.621207478
Ranbp1	55	1.265077769	0.807221514	-0.457856255	0.42506389	0.621677113
Mlxip	52	1.443225384	1.461527451	0.018302067	0.42509937	0.621677113
Furin	125	0.987171655	0.915249637	-0.071922018	0.42522714	0.621801996
Atp6v0b	43	1.4658078	1.327398254	-0.138409546	0.42553316	0.622187491
Puf60	33	0.944159251	1.087864978	0.143705727	0.4255768	0.622189301
Tead4	78	1.318285051	1.301366696	-0.016918354	0.42579653	0.622448524
Macrod1	37	0.885969967	0.625933115	-0.260036852	0.42592962	0.62252719
Tuba3b	20	96.29647338	95.28726559	-1.009207786	0.42593519	0.62252719
330408A02R	22	1.584148315	1.823782667	0.239634352	0.42609455	0.62266897
Tmed8	72	1.387459945	1.126601043	-0.260858903	0.42611706	0.62266897
Cnot8	23	0.826200224	0.704677487	-0.121522737	0.42650929	0.623180063
Adipor1	102	1.161197304	0.940536629	-0.220660674	0.42655337	0.62318241
Arhgap11a	27	2.040653239	1.483420912	-0.557232327	0.42661637	0.623212402
Ddx5	45	1.342401311	1.667747741	0.32534643	0.42679517	0.623349496
Grk4	33	1.467817045	1.154759212	-0.313057833	0.42677735	0.623349496
P2ry4	1	58.49056604	54.63414634	-3.856419696	0.42696666	0.62353789
130020L05R	43	1.599880089	1.228526614	-0.371353475	0.42702705	0.623564029
Fam21	17	1.651072206	1.967503039	0.316430833	0.4271042	0.623614625
Srrd	14	0.891535573	0.77704592	-0.114489653	0.42727924	0.623808142
Zfp280b	23	1.023598541	1.027387694	0.003789153	0.42738718	0.623903645
Nup214	45	1.431322226	1.35252915	-0.078793076	0.42771512	0.624196089
Nfyc	45	1.000090631	0.983538375	-0.016552256	0.42769969	0.624196089
Klf13	110	0.873961604	0.981414311	0.107452707	0.42763524	0.624196089
Mkln1os	6	1.091269841	1.522941532	0.431671691	0.42779099	0.624244722
Gsk3b	26	1.13077776	0.99941609	-0.13136167	0.42808045	0.624294624

Mllt3	26	1.932769816	1.625179985	-0.307589831	0.42796863	0.624294624
Psmc6	41	1.865917192	1.992568357	0.126651166	0.42791445	0.624294624
Mrps27	5	0.536936937	1.655555556	1.118618619	0.42802698	0.624294624
Impdh2	17	0.790939237	0.566824815	-0.224114422	0.42805652	0.624294624
Fkbp11	3	8.981767007	9.656943506	0.675176499	0.42793735	0.624294624
Aagab	61	1.52986393	1.274066675	-0.255797254	0.42818638	0.624387059
Pla2g15	17	1.658869188	1.872039289	0.213170101	0.42823849	0.624401001
Arl14ep	33	2.318535995	2.027152697	-0.291383298	0.42846362	0.624667186
Srd5a2	7	23.97741148	21.65400426	-2.323407213	0.42902147	0.625418345
Sh3glb2	38	1.221753678	1.145998258	-0.075755419	0.42913769	0.625460882
Inip	33	1.280759042	1.557407081	0.276648039	0.42917549	0.625460882
Nup98	34	1.420468338	1.700475662	0.280007324	0.4291843	0.625460882
'00046G09F	70	1.41760855	1.429722234	0.012113685	0.42922515	0.625460882
Gstz1	27	1.06972966	1.052467865	-0.017261795	0.42926377	0.625460882
Fsd1	2	23.41269841	26.70744139	3.294742974	0.42938305	0.62557256
Oaz1	15	1.019737299	0.980460434	-0.039276865	0.42977677	0.626021874
Trp53bp1	35	1.50508516	1.652187422	0.147102262	0.42974363	0.626021874
Serac1	8	1.166666667	0.176056338	-0.990610329	0.43005423	0.626363846
Golga5	72	1.27072394	1.366526845	0.095802904	0.43011255	0.62638662
Zfp384	91	1.721670389	1.543534004	-0.178136385	0.43056826	0.626988057
Aurkb	18	1.355205177	1.036644648	-0.31856053	0.43069599	0.627111824
Mfsd12	55	0.989198688	0.929957628	-0.05924106	0.43078827	0.627183953
Insig1	74	1.12536274	0.952382512	-0.172980228	0.43089001	0.627269839
310006K23F	11	1.96844734	2.172548354	0.204101013	0.4311591	0.627429702
L3mbtl3	66	1.428986215	1.604534585	0.17554837	0.43115468	0.627429702
Coq2	30	0.965563213	1.168529132	0.202965919	0.43105096	0.627429702
Abt1	13	1.504117694	1.067584816	-0.436532878	0.43117085	0.627429702
Ttc19	103	1.119915143	1.207093585	0.087178442	0.43137479	0.627664217
Hs1bp3	17	0.783586243	1.174219726	0.390633483	0.43147191	0.627743293
Ap3m2	42	1.28445517	1.362102062	0.077646892	0.43171625	0.627974248
Dnm1l	50	1.680163229	1.157586394	-0.522576835	0.43170519	0.627974248
Psmd10	6	18.51543583	16.84962379	-1.665812047	0.43180285	0.628037958
Donson	98	1.299349195	1.245083058	-0.054266136	0.43188072	0.628088962
Tbata	9	73.48828484	75.09278239	1.604497549	0.43203109	0.628245389

Dnajb4	29	0.51647914	0.911350523	0.394871382	0.43226295	0.628520265
Cacnb3	101	5.746501437	5.606381513	-0.140119924	0.43231612	0.628535298
Wdr11	31	1.564802683	1.481046914	-0.083755769	0.432399	0.628593507
Lmna	64	2.668358641	2.863329682	0.194971042	0.43258256	0.628798073
Ythdf1	76	0.880851849	0.867013961	-0.013837887	0.43263129	0.628806616
Prep	116	1.237730268	1.188626393	-0.049103875	0.43296613	0.629183924
Nup62-il4i1	16	2.039213599	1.663877872	-0.375335726	0.43297664	0.629183924
Pcbd2	38	1.001135633	0.795499597	-0.205636036	0.43316093	0.629327079
Tcaf2	5	18.0892372	17.61854348	-0.470693716	0.43315037	0.629327079
130304I02R	45	0.863591069	1.339518138	0.475927069	0.43326313	0.629413241
Zscan21	11	1.352544502	0.982558803	-0.3699857	0.43354075	0.629691872
Farsb	10	0.921747134	1.224265553	0.302518419	0.43351604	0.629691872
Cenpn	11	1.49222661	0.753901868	-0.738324742	0.43375229	0.629859916
Atad3aos	5	0.75071464	0.540048455	-0.210666185	0.43377767	0.629859916
Trpm4	20	1.49782306	2.109673081	0.611850021	0.43378522	0.629859916
Eaf1	40	1.594008867	1.689141691	0.095132825	0.43383029	0.629863036
Dlgap5	13	1.48200469	1.029914627	-0.452090063	0.43393369	0.629928221
Zswim3	8	1.827756488	1.248005791	-0.579750697	0.43396105	0.629928221
Casc1	2	0	0.264550265	0.264550265	0.43416066	0.63003101
Taf7l	20	94.11883818	94.03243367	-0.086404505	0.43413726	0.63003101
Dnajc28	11	1.774271259	1.548086412	-0.226184846	0.43410088	0.63003101
Rgmb	88	1.429608259	1.502876874	0.073268615	0.4343922	0.630212515
Psmf1	2	6.060606061	4.664243683	-1.396362378	0.43440531	0.630212515
Gm5475	29	2.792400147	2.615542211	-0.176857935	0.43441458	0.630212515
Mir1956	5	0.816326531	0	-0.816326531	0.4344613	0.630217978
Rbak	21	1.11777281	0.943535107	-0.174237704	0.43452541	0.630248674
Romo1	54	0.892730863	0.903765303	0.011034439	0.43467832	0.630408157
Spc24	3	1.834862385	2.234909036	0.40004665	0.43523011	0.630958965
Polr2h	30	1.789930888	1.614625829	-0.175305059	0.43517291	0.630958965
Nceh1	24	2.292588809	1.5517685	-0.740820308	0.43517113	0.630958965
Blmh	41	1.746438952	1.48023167	-0.266207283	0.43520362	0.630958965
Abcc1	57	0.978123375	0.909637723	-0.068485652	0.43553531	0.631339048
530005N14F	80	1.080522279	0.944441835	-0.136080443	0.43560297	0.631374745
Mtfmt	32	1.225685846	1.148847089	-0.076838757	0.43571702	0.631477679

Cadm3	31	45.57325357	45.37173561	-0.201517968	0.43592421	0.631715574
Tra2b	34	1.677502849	1.076027571	-0.601475279	0.43603231	0.63180982
Klf9	69	0.638395018	0.619553802	-0.018841217	0.43614922	0.631830381
Nol11	37	1.525330763	1.368263002	-0.157067761	0.43617487	0.631830381
Thumpd1	47	0.96545073	1.260420599	0.294969869	0.43621873	0.631830381
Manba	25	1.854049807	1.791641444	-0.062408362	0.436198	0.631830381
Tprgl	74	1.172505842	1.194391985	0.021886142	0.43634588	0.631952181
Tubb6	52	1.619474029	1.400534455	-0.218939574	0.43641432	0.631988923
Zfp69	53	1.197477506	1.236759154	0.039281649	0.43652445	0.632086024
AU040320	49	1.42812525	1.380936506	-0.047188744	0.43660041	0.632133631
C2cd4c	40	4.311511984	4.420897982	0.109385998	0.436819	0.63238773
Mrpl18	6	0.732385431	0.421940928	-0.310444502	0.4371386	0.632787981
Dclre1c	5	2.228229577	2.240576205	0.012346628	0.43727317	0.632857922
Slitrk1	2	4.206349206	6.298076923	2.091727717	0.43725873	0.632857922
Cep68	56	1.268992757	1.265366338	-0.003626419	0.43737188	0.632884239
Dbx1	38	45.02842195	44.15690834	-0.87151362	0.43737761	0.632884239
Adpgk	70	1.327493439	1.339443598	0.011950159	0.43750351	0.633003995
Tmem101	18	0.757082538	0.825312841	0.068230304	0.43757219	0.633040946
Slc7a6os	34	0.622343989	0.640448976	0.018104987	0.43776971	0.633158522
Sec24b	92	1.195371125	1.27108796	0.075716835	0.43775857	0.633158522
Rpl18a	29	1.032230452	0.969861009	-0.062369443	0.43778291	0.633158522
Pinx1	31	1.447410322	1.154474407	-0.292935915	0.43783528	0.63317186
Rab25	3	85.83377963	88.65447683	2.820697199	0.43802062	0.633377477
Zscan22	16	1.954055302	1.072301235	-0.881754068	0.43808627	0.633409981
Mthfsd	9	0.932530385	1.241501359	0.308970974	0.43822508	0.63348584
Pcdhga5	4	50.97527473	49.22952586	-1.745748863	0.43818386	0.63348584
Hk1	22	1.580853551	1.516668224	-0.064185327	0.43842975	0.633719283
Hck	20	11.34283759	12.16914928	0.826311688	0.43858243	0.633752688
Srd5a3	34	1.193749513	1.223102144	0.029352631	0.43852388	0.633752688
Il25	1	49.52380952	54.90196078	5.378151261	0.43855323	0.633752688
Wdr47	31	1.05885152	1.008086761	-0.050764758	0.4386318	0.63376162
Pgrmc2	70	1.24306942	1.036653115	-0.206416305	0.43878656	0.633904598
Hcfc2	54	0.977638891	0.923596138	-0.054042752	0.43881715	0.633904598
Npff	1	95.38461538	97.6744186	2.28980322	0.43893445	0.634011629

Mzt2	8	1.720872518	1.56029671	-0.160575809	0.43898451	0.634021531
Lmnb2	15	3.361396152	2.423537339	-0.937858813	0.43904816	0.634051054
Dnajc18	30	1.88324048	1.490752925	-0.392487554	0.43914289	0.63412545
Sirt6	18	2.455218832	2.932095435	0.476876603	0.43924954	0.634217037
Extl2	18	2.517426452	2.250865553	-0.266560898	0.43930743	0.634238213
Rps14	46	1.219084056	1.46935629	0.250272234	0.43989956	0.635030616
Lbh	36	2.385901207	1.75943218	-0.626469027	0.44004182	0.635173487
Sypl	66	1.362035001	1.135593498	-0.226441503	0.44080419	0.636179576
Ddx54	47	1.171811918	1.074688599	-0.097123319	0.44082554	0.636179576
Atp6ap2	6	22.39857112	23.74625799	1.347686869	0.44087044	0.63618181
Itgb3bp	11	0.495867769	0.685930231	0.190062463	0.44095599	0.636242696
Prdx1	29	1.449016185	1.247934743	-0.201081442	0.44107983	0.636340705
.30308A19F	88	1.219159765	1.125289647	-0.093870118	0.44111065	0.636340705
Atp11a	127	5.138217673	5.818691076	0.680473403	0.44115943	0.636348515
Poldip3	67	1.100450957	0.989979715	-0.110471243	0.44126752	0.636441867
Tmem216	8	2.019296448	1.585777763	-0.433518685	0.44132259	0.636458733
Fut8	106	1.326713295	1.316595228	-0.010118067	0.44140126	0.636509641
Tysnd1	52	1.333978767	1.187780083	-0.146198685	0.44145391	0.636523006
Lgals1	3	4.581309915	5.019832281	0.438522366	0.44180623	0.63696842
Tsr1	68	1.537440154	1.265188336	-0.272251818	0.44200813	0.637196903
Rpph1	19	1.606438055	1.950175406	0.343737352	0.44213833	0.637321995
Derl1	66	1.38951223	1.471777076	0.082264846	0.44258414	0.637901949
Nsd1	25	80.74388774	80.03296454	-0.710923198	0.44279629	0.638145043
Cct6b	11	0.558114874	0.636553889	0.078439015	0.44294532	0.638256129
Glg1	43	1.513356291	1.302013784	-0.211342508	0.44296037	0.638256129
Pithd1	49	5.711790815	6.188507202	0.476716387	0.4431137	0.638414379
Pbrm1	31	1.127858841	1.072102699	-0.055756142	0.4433569	0.638702054
Tbc1d24	45	2.040649954	2.02696332	-0.013686634	0.4435238	0.638879767
Nipsnap3b	15	1.464325798	0.96071191	-0.503613888	0.4435815	0.638900176
Aup1	28	2.365486432	1.790000875	-0.575485557	0.44396016	0.63933338
Zfp946	8	4.094623052	3.198895493	-0.895727559	0.44396941	0.63933338
Vps33b	23	1.272870799	1.005073515	-0.267797284	0.4441831	0.639578337
Myo1e	27	1.471608048	1.209187486	-0.262420562	0.4443639	0.639775895
Il17d	95	1.984159081	1.878752388	-0.105406693	0.44472231	0.64022909

Letmd1	58	1.420921523	1.296344541	-0.124576983	0.4447822	0.640252501
Frs3	60	1.519391874	1.416772346	-0.102619528	0.44486115	0.640303331
Greb1l	55	9.624927147	9.59235825	-0.032568897	0.44500463	0.640355357
Glud1	112	1.049134713	1.011566865	-0.037567848	0.44510466	0.640355357
Mir7088	1	98.4	96.84210526	-1.557894737	0.44511637	0.640355357
Alas1	66	1.837593862	1.458313574	-0.379280287	0.4449463	0.640355357
Cmip	186	1.358409072	1.26817548	-0.090233591	0.44515913	0.640355357
Mst1	1	98.4	96.84210526	-1.557894737	0.44511637	0.640355357
Psen1	45	1.482978432	1.170229086	-0.312749347	0.44525044	0.640423936
Bend6	85	1.405942519	1.258700429	-0.14724209	0.44546756	0.640673422
Arhgap35	7	1.920129453	2.381005468	0.460876015	0.44556048	0.640744259
Nexn	15	9.974657098	9.14845076	-0.826206338	0.44582583	0.641002632
Ppil3	8	2.304975557	2.064071639	-0.240903918	0.4458415	0.641002632
Carf	8	1.461901037	1.050751991	-0.411149046	0.44587119	0.641002632
Mark4	42	2.018424897	2.017277726	-0.00114717	0.44596179	0.641070074
Scarna13	28	2.198409791	2.692944672	0.494534881	0.44609696	0.64120156
Pacrgl	28	2.037575387	2.56508498	0.527509593	0.44620156	0.641289102
Lyrm1	20	2.212681383	2.767793441	0.555112058	0.44633428	0.641356912
Shfm1	46	1.061616917	1.060435574	-0.001181342	0.44634237	0.641356912
Trim27	56	1.39802071	1.122843527	-0.275177183	0.44637987	0.641356912
Pfdn6	8	0.791156446	0.938514097	0.147357651	0.44642558	0.641359795
Mir7662	1	94.69026549	96.55172414	1.861458651	0.4464957	0.641397746
Rabggtb	24	1.465277256	1.416570546	-0.048706711	0.44663341	0.641407183
Dcaf6	73	1.603030236	1.351046291	-0.251983945	0.44660103	0.641407183
Eif2b4	43	1.557985911	1.417057308	-0.140928603	0.44656719	0.641407183
Tmem191c	21	0.935039276	1.31088002	0.375840744	0.44684024	0.641641416
Parvg	3	59.00288225	60.26139822	1.258515963	0.44713286	0.64199879
Map3k14	74	2.174631567	1.945401456	-0.229230111	0.44734275	0.642237313
Zdhhc12	57	1.693918979	1.781949454	0.088030475	0.44772596	0.642724586
Hic2	52	1.244423284	1.365288665	0.120865381	0.4478484	0.642837466
Zfp810	33	1.32339355	1.461787448	0.138393898	0.44796394	0.642940426
Sepsecs	36	1.421362683	1.493754353	0.072391671	0.44812263	0.643105273
Zfp715	52	1.548619354	1.560597177	0.011977823	0.44849753	0.643391607
B4galt3	29	18.32163098	18.24371191	-0.077919073	0.44846672	0.643391607

Ciao1	25	1.066687159	1.33825827	0.271571111	0.44839085	0.643391607
Mrps16	32	1.120975384	1.081484454	-0.03949093	0.4484105	0.643391607
Lox	3	29.52380952	32.41584158	2.89203206	0.44880212	0.643639784
Serpinb6a	59	1.202430725	1.071597908	-0.130832817	0.44873502	0.643639784
Trpa1	8	20.62916399	19.91805291	-0.711111081	0.4487789	0.643639784
Cacybp	92	1.329540401	1.069576434	-0.259963967	0.44892393	0.643751564
Ndufa6	45	1.248047472	1.293183367	0.045135896	0.44904973	0.64380613
Edem1	84	0.922160076	0.928720138	0.006560062	0.44902337	0.64380613
Gatad1	108	1.582750535	1.498103769	-0.084646766	0.44911721	0.643839966
Srsf3	61	1.252670554	1.132790356	-0.119880197	0.44930183	0.644041722
G3bp1	72	1.103910334	0.895538573	-0.208371762	0.44942582	0.644156519
Adora2b	38	2.740317168	2.785535279	0.045218111	0.449635	0.644350332
Sf3b4	36	1.827763539	2.053113852	0.225350313	0.44964886	0.644350332
Pcdha5	3	69.31818182	73.44444444	4.126262626	0.44972411	0.644395228
Spsb2	27	1.721399171	1.810912953	0.089513782	0.45004102	0.644719175
Zfp790	7	1.447230074	1.26269528	-0.184534794	0.45002782	0.644719175
Tmem208	14	0.97064483	1.340627906	0.369983076	0.450082	0.644719175
Grtp1	13	1.045817444	1.086550417	0.040732974	0.45019629	0.64475702
Pbx3	145	1.36881213	1.274982543	-0.093829587	0.45016377	0.64475702
Ybx1	92	1.388360301	1.253322056	-0.135038245	0.45033904	0.64489851
Cdkn3	28	1.134214948	0.864855656	-0.269359292	0.45038473	0.644901007
Cab39	46	0.839573862	0.885776047	0.046202185	0.45044848	0.644929364
Prpf4b	35	0.665959177	1.400462836	0.734503659	0.45050862	0.644952531
Notch1	14	1.92078532	1.435231857	-0.485553463	0.45067298	0.645124891
Ocel1	49	1.445807576	1.328952315	-0.11685526	0.45080617	0.645252613
Camsap1	95	1.281696362	0.969279523	-0.312416839	0.45098934	0.64545183
Arl3	7	1.059900409	1.16487219	0.104971781	0.45112704	0.645585934
Tbcc	38	1.582242825	1.317323621	-0.264919205	0.45126853	0.645669572
Ung	66	1.261494641	1.298188945	0.036694304	0.45127925	0.645669572
Heatr5a	37	1.958644619	1.686893544	-0.271751075	0.45131749	0.645669572
Naaa	10	2.676397758	2.412002437	-0.264395321	0.45169926	0.646063906
Mllt1	127	3.646562897	3.690424595	0.043861698	0.4517009	0.646063906
Slc43a2	75	1.270579211	1.32394286	0.053363648	0.45172521	0.646063906
Mdfic	25	1.164553062	1.165000837	0.000447775	0.45200961	0.64640387

Baz1b	110	1.285700071	1.326309144	0.040609073	0.45205101	0.64640387
Wrb	25	1.26175714	1.064256168	-0.197500972	0.45211075	0.646426308
Chpf2	29	1.274005612	1.237851001	-0.036154611	0.45227932	0.646541323
Zfp296	31	2.954747289	2.642217177	-0.312530112	0.45226236	0.646541323
Tmem127	46	1.699458269	1.794658581	0.095200312	0.45234177	0.646554583
Fam107b	35	1.366873796	1.271770138	-0.095103658	0.45238642	0.646554583
Aacs	37	1.074111298	1.084523928	0.01041263	0.45242078	0.646554583
Bcr	115	1.448148811	1.315002632	-0.133146179	0.45254829	0.646673841
Tm2d3	59	1.331385933	1.345415837	0.014029905	0.45281796	0.64691607
Socs2	117	1.385242534	1.233820796	-0.151421737	0.45287832	0.64691607
Loxl4	18	1.076318329	1.56740636	0.49108803	0.45279901	0.64691607
Nipa1	44	1.173179247	1.24222892	0.069049673	0.45289415	0.64691607
Anxa2	27	1.656330271	1.342659341	-0.31367093	0.45301609	0.647027265
Dusp1	89	1.155944585	1.168600356	0.012655772	0.45315671	0.647165123
Setbp1	171	1.243390488	1.218158343	-0.025232145	0.45328055	0.647210019
Tubd1	19	2.728281331	2.24101552	-0.487265812	0.45332047	0.647210019
Foxq1	46	11.74953182	10.89545127	-0.854080553	0.45330879	0.647210019
Dffb	84	1.573703521	1.474634765	-0.099068756	0.45353821	0.647457891
)30473A02F	11	1.590717507	2.232832098	0.642114591	0.45360525	0.64749061
Cfl1	68	1.123309039	1.266271758	0.142962719	0.45396364	0.647862336
Ado	95	0.906829564	0.896796811	-0.010032753	0.45399812	0.647862336
Tex30	56	1.18638797	1.372817853	0.186429883	0.45392465	0.647862336
700026F02F	3	48.65460882	46.10329639	-2.551312427	0.45440569	0.648143767
Nanos2	1	29.33333333	24.17582418	-5.157509158	0.45431981	0.648143767
Ppp5c	24	1.569017398	1.566151789	-0.00286561	0.4543495	0.648143767
)30007P13F	17	8.181078642	8.046192481	-0.13488616	0.45441618	0.648143767
Ddit3	24	1.605654987	1.413357625	-0.192297362	0.45435935	0.648143767
Chic2	105	1.291485877	1.233986598	-0.057499278	0.45448069	0.648172769
Nkx2-9	14	55.00070206	56.72393257	1.723230511	0.45472422	0.64833105
Rwdd2b	21	1.214018196	0.834699724	-0.379318472	0.4547242	0.64833105
Ell	31	1.307991384	1.504508557	0.196517173	0.45471212	0.64833105
Lgmn	36	1.710304018	1.378313444	-0.331990574	0.45485739	0.648457911
Tstd3	10	2.074762577	2.675928373	0.601165795	0.45502937	0.648640079
Gpa33	2	81.11497501	82.92338862	1.808413618	0.45557562	0.648886422

Plagl2	72	1.126817503	0.924179243	-0.20263826	0.45532921	0.648886422
Pa2g4	43	1.093721686	1.178392507	0.084670821	0.45525369	0.648886422
Rrp1b	16	1.466723998	1.252403162	-0.214320836	0.45555425	0.648886422
Rnd3	3	2.630052755	2.849002849	0.218950094	0.45560016	0.648886422
Clu	18	2.527584148	2.334072379	-0.193511769	0.45554086	0.648886422
Zmynd12	43	1.718828062	3.023659497	1.304831435	0.455509	0.648886422
.30311K13F	51	1.326386999	1.273588014	-0.052798985	0.455393	0.648886422
Rnf216	83	1.165136935	1.282436902	0.117299967	0.45555899	0.648886422
Tsx	1	93.67088608	90.625	-3.045886076	0.45598395	0.649256416
Tceanc2	14	0.926565534	1.387107499	0.460541964	0.45590679	0.649256416
Otud7b	51	0.975596294	0.976858516	0.001262222	0.45599268	0.649256416
310055G02F	32	1.566697865	1.316959477	-0.249738387	0.45620997	0.649439761
Accs	20	1.875578572	2.231003419	0.355424847	0.45620404	0.649439761
)30612E09F	42	8.10065393	7.655273791	-0.445380139	0.45692374	0.650392765
Tbl2	19	2.589875052	2.495170591	-0.094704461	0.45717119	0.650681873
Zfp420	8	1.020108208	1.035919129	0.015810921	0.45741447	0.65077564
Vip	3	19.84755199	21.40877972	1.561227722	0.45737805	0.65077564
Cbwd1	7	1.713288709	1.910315289	0.19702658	0.45731404	0.65077564
Gabpb1	49	2.121023952	2.462726615	0.341702663	0.45732626	0.65077564
Ppp2r5c	109	1.364533247	1.486980724	0.122447476	0.45750067	0.650835173
Snx17	24	1.390676192	1.27506885	-0.115607342	0.45770986	0.651069647
Cdk9	48	1.348743218	1.016418063	-0.332325155	0.45791394	0.651296811
Snupn	12	0.963442867	0.500471827	-0.46297104	0.45837842	0.651894253
Pck2	3	1.642522636	1.610904585	-0.031618051	0.45868302	0.652264236
Tap1	12	0.892778291	0.6641753	-0.228602991	0.45892407	0.65254378
Eif2s1	34	1.594386162	1.139557137	-0.454829025	0.45910273	0.652671323
Tmem29	18	2.913550948	2.024282716	-0.889268233	0.45907927	0.652671323
H2-T9	7	1.590702604	2.262577402	0.671874798	0.45922305	0.652779135
Wdr89	11	1.183260698	2.10297496	0.919714263	0.45929322	0.652815644
Pam16	28	1.237393246	1.386878333	0.149485087	0.4595014	0.652998321
Magef1	32	0.884234598	0.870395189	-0.013839409	0.45951074	0.652998321
Slain2	100	0.88670949	0.895315532	0.008606041	0.45955597	0.652999351
Lama1	8	50.60098641	50.15837868	-0.442607723	0.4596559	0.653078108
Chp1	33	1.296363734	0.961015463	-0.335348271	0.45971689	0.653101524

Simc1	75	1.43897563	1.267584277	-0.171391353	0.45985235	0.653147961
Ddhd1	26	2.084732459	2.449179548	0.364447089	0.45988311	0.653147961
C1qtnf6	4	36.79986739	39.85068131	3.050813916	0.45981087	0.653147961
Olig1	9	7.997200269	9.177933178	1.180732909	0.45997678	0.653154563
Gm16897	30	1.29372117	1.206948988	-0.086772182	0.4599596	0.653154563
Hccs	34	17.2326103	17.40448391	0.171873611	0.46005873	0.653207729
Rbm15b	155	2.265513136	2.07450749	-0.191005646	0.46014949	0.653273371
Rab4a	30	1.02121293	1.198091025	0.176878095	0.4604983	0.653705333
Fam171a2	51	4.008784898	3.678645323	-0.330139575	0.46079918	0.65406918
Hdac11	6	3.11325929	1.950318393	-1.162940896	0.46100291	0.654231796
Mgat2	80	1.154772258	0.9013603	-0.253411958	0.46098777	0.654231796
Ereg	9	5.595912817	5.223195285	-0.372717532	0.46143167	0.654776943
Rp9	103	1.165729825	1.214892059	0.049162233	0.4614924	0.654799789
Lrp6	105	1.189422388	0.975718114	-0.213704274	0.46158462	0.654867326
Dnm2	29	1.600836616	1.423603891	-0.177232725	0.46207517	0.655395526
Ndufa2	8	0.86407767	1.090338706	0.226261036	0.4620551	0.655395526
Vmac	44	1.732816472	1.824154964	0.091338492	0.46209092	0.655395526
Capns1	24	1.90428111	1.96405112	0.05977001	0.46235158	0.655574709
Setd1b	82	1.490511087	1.492647631	0.002136544	0.46239595	0.655574709
Cog2	15	1.106139892	0.756934356	-0.349205536	0.46233022	0.655574709
Orc4	18	1.630543949	1.862851397	0.232307447	0.46236001	0.655574709
Man2c1	15	1.302238244	1.128852242	-0.173386002	0.46255006	0.655649328
Gm5868	18	22.37207507	21.78488359	-0.587191477	0.46258263	0.655649328
Foxj3	145	1.149623249	1.039854893	-0.109768356	0.46255613	0.655649328
Shc4	2	3.188992109	2.784446015	-0.404546093	0.46265804	0.655692883
Fam122a	49	1.611017065	1.602607451	-0.008409615	0.4627175	0.655713821
Zfp599	14	2.332812863	1.918731333	-0.41408153	0.4628645	0.655858792
Nxt1	48	1.001349237	1.109971746	0.108622509	0.46294528	0.655908361
Dsn1	37	1.366763485	1.499901813	0.133138328	0.46298888	0.655908361
Mfsd8	16	2.557783969	2.57333878	0.015554811	0.46304598	0.655925933
Map4k5	28	0.802396312	0.783280668	-0.019115644	0.46315764	0.656020779
'00011C11F	2	16.09219269	14.97763966	-1.114553034	0.46325847	0.656100259
Sc5d	48	1.108726538	1.196996787	0.088270249	0.46333003	0.656138277
Isca1	69	1.082175443	0.956604173	-0.12557127	0.46341481	0.656195018

Atp6v1c1	56	1.346624574	1.195165871	-0.151458703	0.46349116	0.656239806
Zfand1	25	1.615501643	1.678431263	0.06292962	0.46365001	0.656274738
Mir320	26	1.363410534	1.524426216	0.161015682	0.46361045	0.656274738
i10030E20F	43	1.02404708	0.953436229	-0.070610851	0.46360429	0.656274738
Cnnm3	93	1.622911265	1.623687354	0.000776089	0.46381438	0.656408226
Zfp329	28	1.648342288	1.104174971	-0.544167318	0.46383378	0.656408226
Csnk1a1	74	1.017500588	0.967663092	-0.049837497	0.46398174	0.656554303
Dst	131	1.71039989	1.565545917	-0.144853974	0.46441656	0.656979518
Ccdc53	13	1.907521686	2.032563251	0.125041565	0.4643628	0.656979518
Eef1a1	64	1.431336257	0.930455868	-0.500880389	0.46438806	0.656979518
Mcf2l	8	51.02153057	49.96750831	-1.054022257	0.46454349	0.657095733
Fam120aos	186	1.26855752	1.226105156	-0.042452364	0.46505806	0.657696794
Slc30a1	98	1.09279491	1.041286261	-0.051508649	0.46502092	0.657696794
Tubb4b	33	1.360026049	1.571007468	0.210981419	0.46523947	0.657826556
Tmem126a	32	1.402883582	1.228149487	-0.174734095	0.46521069	0.657826556
Chchd2	20	3.281351577	2.471124088	-0.810227488	0.46565307	0.658102733
Pcf11	65	1.221212588	1.009749039	-0.211463548	0.46560348	0.658102733
Mkln1	55	1.179457687	0.862547283	-0.316910404	0.46565904	0.658102733
Vezf1	87	1.469892937	1.714334438	0.244441501	0.46561195	0.658102733
Stx11	38	0.95261711	1.140214073	0.187596964	0.46564747	0.658102733
Mul1	43	1.775916845	1.598221479	-0.177695365	0.46571549	0.65811913
Snhg4	10	1.530458721	1.152290702	-0.378168018	0.46599286	0.658300375
Atxn2l	92	1.54734679	1.294367587	-0.252979204	0.46602319	0.658300375
Ccdc84	32	1.494017591	1.349630582	-0.144387009	0.46601191	0.658300375
Litaf	34	2.032014284	1.587961612	-0.444052672	0.46595715	0.658300375
Gm14325	2	8.013322884	7.061532888	-0.951789996	0.46611871	0.658308554
Neurl4	104	1.133973158	1.115430406	-0.018542751	0.46610696	0.658308554
Pgap2	35	2.03221572	2.034730247	0.002514527	0.46622426	0.658330908
Rab10	97	1.37913039	1.107508221	-0.271622169	0.46620079	0.658330908
Alg11	27	1.005882845	0.947943522	-0.057939323	0.46627012	0.658332312
Tomm34	60	1.536179944	1.300910538	-0.235269406	0.46636639	0.658404885
Ubn2	64	0.939805913	1.092171595	0.152365682	0.46652953	0.658571841
Tram2	29	1.510774485	1.364686237	-0.146088248	0.4666149	0.658628989
Il1rapl2	11	37.38082989	40.09814358	2.717313687	0.46667087	0.658644637

Mkrn2	18	0.751341355	0.964475576	0.213134222	0.46679709	0.658759424
Mir6966	4	71.31895417	72.82478682	1.505832649	0.4669465	0.658906912
Supv3l1	31	1.287697362	1.272251697	-0.015445665	0.46700645	0.658928144
Ccdc32	15	1.752788702	2.077361012	0.32457231	0.46746797	0.659515915
Pus7l	5	2.058729797	1.169092945	-0.889636852	0.46774662	0.659591953
Palm	30	1.038342581	1.09931637	0.060973789	0.46771121	0.659591953
Sdf2	24	1.68508696	1.39308972	-0.29199724	0.46764287	0.659591953
Hoxd1	51	52.99434311	52.71872981	-0.275613295	0.46774011	0.659591953
Snord35b	20	1.166127211	1.32176357	0.155636359	0.46765756	0.659591953
Ets1	33	1.320667742	1.252212567	-0.068455175	0.46788316	0.659657718
'00024G13F	1	53.39805825	48.98989899	-4.408159263	0.46786757	0.659657718
Mex3d	119	1.221732982	1.030926667	-0.190806314	0.46798577	0.659738998
Ntn4	89	1.837757436	2.135164412	0.297406976	0.46813405	0.65988463
Cep97	8	0	0.412087912	0.412087912	0.46823185	0.659959104
Xbp1	41	1.307216777	0.998521498	-0.308695279	0.46834109	0.660049666
Dcp2	53	1.665068983	1.540508051	-0.124560932	0.46841832	0.660095116
Pdlim3	9	18.92695218	19.50696141	0.580009221	0.46854127	0.660141588
Pex10	21	1.179094329	0.738867454	-0.440226875	0.46852583	0.660141588
Kdm5a	50	1.482451089	1.084745216	-0.397705873	0.46866102	0.660246903
Pcdhgc3	21	2.183999341	1.646040274	-0.537959067	0.46888025	0.660492353
Gltp	55	1.395806181	1.180811158	-0.214995023	0.46899941	0.660596787
Mir7687	68	1.752675932	1.674120143	-0.078555789	0.4691964	0.660810827
Srsf9	73	0.913731007	0.844254498	-0.069476509	0.46925972	0.660836583
Zc3h14	46	1.095872419	0.965130409	-0.13074201	0.46947135	0.660993034
Dhx57	15	1.554229481	1.772930719	0.218701238	0.46942286	0.660993034
Pex7	49	1.065758165	0.943769383	-0.121988782	0.46950596	0.660993034
Nek11	17	0.959070759	0.857881702	-0.101189057	0.46958839	0.661045671
Por	43	1.714475509	1.705453448	-0.00902206	0.46973808	0.66112954
Zfp219	211	1.578583486	1.528052498	-0.050530988	0.46970252	0.66112954
Akap1	39	1.083895792	1.132448046	0.048552254	0.46981742	0.661177796
Tctex1d2	15	1.927757556	1.483790858	-0.443966698	0.46991671	0.661254106
Bbx	34	0.878712695	1.011062655	0.13234996	0.46998451	0.661286094
Ang	2	2.201257862	2.006799717	-0.194458144	0.47018465	0.661355626
R3hcc1l	36	1.266256766	1.30054487	0.034288104	0.47034793	0.661355626

)32416H05F	54	1.976807332	2.050346174	0.073538842	0.47032086	0.661355626
Snx10	5	2.120051086	1.084618981	-1.035432105	0.47027982	0.661355626
Bcl2l1	47	1.392089818	1.638386403	0.246296584	0.47039448	0.661355626
Zfp672	45	1.512083469	1.215340363	-0.296743105	0.47038617	0.661355626
Rnase4	2	2.201257862	2.006799717	-0.194458144	0.47018465	0.661355626
Myadml2	4	9.173172794	9.843363325	0.670190531	0.47008308	0.661355626
D17Wsu92e	62	1.329701215	1.284561944	-0.045139271	0.47078508	0.661714583
Cep44	2	0.505050505	0.676703934	0.171653429	0.47075849	0.661714583
Pdcd2l	27	0.961262327	0.929442976	-0.031819351	0.47072448	0.661714583
Knstrn	27	1.267666449	0.946081971	-0.321584479	0.47092484	0.661720872
Prdx5	15	1.603972122	1.09018756	-0.513784563	0.47087692	0.661720872
Fzd5	75	1.586200233	1.353377838	-0.232822396	0.47091567	0.661720872
Tgfbr2	56	1.322962771	1.351142168	0.028179397	0.4711132	0.661912552
Larp4	61	1.498215625	1.39039005	-0.107825575	0.47121261	0.661912552
Fam63a	27	1.700582587	1.302786819	-0.397795769	0.47116034	0.661912552
Whsc1l1	94	1.406042251	1.075374042	-0.33066821	0.47124168	0.661912552
Rraga	28	0.822865597	0.670245613	-0.152619984	0.47150207	0.662214914
Wdr76	1	3.937007874	2.222222222	-1.714785652	0.47183817	0.662623537
Ammecr1l	62	1.488814277	1.531429708	0.042615431	0.47203464	0.662709169
Chd3os	14	61.78222096	61.38249644	-0.399724515	0.47198412	0.662709169
Tmem255a	5	17.85713006	21.59336057	3.736230508	0.47200753	0.662709169
Gm5544	1	53.09734513	57.69230769	4.59496256	0.47217572	0.662843825
Wdtc1	62	1.48526771	1.535021511	0.049753801	0.47244616	0.663160034
Polr2m	60	1.006258955	0.926010128	-0.080248826	0.47269735	0.663330224
Me2	103	1.354031067	1.29811732	-0.055913747	0.47274823	0.663330224
Pole3	4	1	0.396825397	-0.603174603	0.47270916	0.663330224
Atg16l2	23	1.263332585	1.179578856	-0.083753728	0.47271973	0.663330224
Kbtbd4	11	1.841109923	2.076925364	0.235815441	0.47291743	0.663440757
Gm20753	7	1.754542359	1.754834462	0.000292103	0.47288496	0.663440757
AI314180	75	0.961233353	0.895358079	-0.065875274	0.4730978	0.663566918
Smad7	104	1.51572195	1.31172714	-0.20399481	0.47309153	0.663566918
Serinc4	1	4.473684211	3.473945409	-0.999738801	0.47333109	0.663830676
Gtf2h3	24	0.884192808	0.541769815	-0.342422994	0.47339336	0.663854569
Mfsd2a	46	1.9508816	1.765455242	-0.185426358	0.47346123	0.663886296

Ibtk	69	1.401161621	1.304721572	-0.096440049	0.47381478	0.66431856
Sidt1	7	10.07219491	11.60747028	1.535275372	0.47396767	0.664405957
Med18	23	1.781749972	1.301941996	-0.479807975	0.47392656	0.664405957
Sec14l2	14	4.143948462	5.176543892	1.03259543	0.47403458	0.664436283
Gm5468	7	2.105646265	1.445632441	-0.660013824	0.47419701	0.664600476
Zfp54	5	1.227304068	1.603520619	0.376216551	0.47453951	0.665016974
Mir7045	95	1.859272686	1.595992648	-0.263280038	0.47462711	0.66507622
Dnajc16	51	1.057404703	0.851472832	-0.205931871	0.47492489	0.665429941
Tjp2	191	1.399623649	1.242318314	-0.157305335	0.47502793	0.665510771
Nek1	40	2.230058963	2.338158295	0.108099332	0.4751607	0.665591512
Ndufaf6	41	1.672389751	1.438895467	-0.233494284	0.47517628	0.665591512
Slirp	1	3.459119497	4.559270517	1.10015102	0.47526178	0.665592146
Txnip	33	1.647794539	1.521251113	-0.126543425	0.47526745	0.665592146
Lamtor5	20	1.313840863	1.346602048	0.032761185	0.47554194	0.665913004
Schip1	51	1.758270492	1.599245297	-0.159025195	0.4756592	0.666013656
Mir6941	5	85.50839866	80.74081715	-4.76758151	0.47589817	0.666284678
Eif3b	61	0.853948734	0.905714752	0.051766017	0.47595033	0.66629414
Prrc2b	39	1.068385965	1.298435282	0.230049317	0.4761317	0.666348548
Dus3l	43	1.378109775	1.486626044	0.108516269	0.47635248	0.666348548
Mier2	102	0.957106855	0.995354134	0.038247279	0.47624214	0.666348548
Uqcrc1	13	1.801512159	1.680119552	-0.121392607	0.47634238	0.666348548
Med25	43	1.176377429	0.820466568	-0.355910861	0.47624366	0.666348548
Top2a	21	1.516752808	1.442951606	-0.073801203	0.476238	0.666348548
Mcfd2	61	1.204432478	1.296076493	0.091644015	0.47634285	0.666348548
Cfap20	70	1.433219673	1.216966403	-0.216253271	0.47624141	0.666348548
Adck2	20	1.323898999	1.455546386	0.131647387	0.47640382	0.666356844
Zfp523	60	0.991050105	1.006490503	0.015440398	0.47662744	0.666606082
Mir7666	24	0.379792904	0.469295612	0.089502708	0.47688444	0.666711924
Rabl6	70	1.255309989	1.299305396	0.043995407	0.47688485	0.666711924
Champ1	39	1.381622995	1.054909078	-0.326713917	0.47686289	0.666711924
Wdr77	52	1.440506362	1.457846709	0.017340347	0.47683049	0.666711924
Sap130	36	1.637470306	1.669621345	0.032151039	0.4771291	0.666862782
Ccr4	1	91.73553719	89.87854251	-1.85699468	0.47705908	0.666862782
Mtcl1	112	1.400843422	1.280287034	-0.120556387	0.47710656	0.666862782

Atxn1	37	1.58280289	1.351164696	-0.231638194	0.47733429	0.667086039
Ccdc103	13	2.096290373	1.447606776	-0.648683597	0.47742133	0.667144134
Rnf4	53	1.261197791	1.280602761	0.01940497	0.47817724	0.668125312
Dennd4b	11	1.116628785	1.315879184	0.199250399	0.47821454	0.668125312
Uba3	32	0.987612097	1.043311939	0.055699842	0.4783892	0.6683057
Akna	11	28.85888212	29.48110364	0.622221519	0.47866867	0.668632467
Hint1	92	1.092880583	0.91312187	-0.179758713	0.4787708	0.668711476
Slc39a14	46	1.079365713	0.995590287	-0.083775427	0.47904183	0.669026342
Snord65	5	5.298749544	4.715007215	-0.583742329	0.47911876	0.669070107
Usp40	9	1.484226882	1.8853636	0.401136718	0.4794088	0.669411431
Cdadc1	18	2.559646452	2.079380111	-0.480266342	0.47955112	0.669546438
Ick	56	1.477471532	1.303920849	-0.173550683	0.47978474	0.669554094
Vps4b	38	1.454711938	1.215895843	-0.238816095	0.47973292	0.669554094
Dus1l	35	6.349385851	6.505383881	0.155998029	0.47960243	0.669554094
Vps54	114	1.291642375	1.10034597	-0.191296404	0.47974014	0.669554094
Mir3086	3	1.243781095	1.560710195	0.3169291	0.47970148	0.669554094
Cox8a	21	1.549309133	1.312426163	-0.236882971	0.47985658	0.669590669
Nanos1	73	1.340552667	1.201336294	-0.139216373	0.47995738	0.66961325
Erp29	42	1.135207592	1.259597024	0.124389432	0.47996403	0.66961325
310022A10F	18	1.788125504	1.351867583	-0.436257921	0.48012574	0.669721563
Bcs1l	24	1.645123736	1.526451112	-0.118672624	0.48013295	0.669721563
33418N02F	49	1.525919052	1.605653106	0.079734054	0.48022775	0.669790131
Sec61g	23	1.084887581	1.437521573	0.352633993	0.4803268	0.669864607
Sfxn2	21	1.24337392	1.262368283	0.018994363	0.48053124	0.670041167
Nbeal1	84	1.60422772	1.260250429	-0.343977291	0.48054472	0.670041167
Ap2b1	50	1.705470912	1.901842286	0.196371374	0.48064735	0.670081178
Ccdc18	24	0.867741	0.626244023	-0.241496977	0.48066475	0.670081178
Mboat2	81	1.492909199	1.636185889	0.14327669	0.48084915	0.670274567
Cadm1	70	1.291621468	1.34645111	0.054829642	0.48098474	0.670399889
Trappc4	30	1.359576607	1.385746391	0.026169784	0.48121232	0.670653388
Hsbp1	48	3.419607347	3.009438988	-0.41016836	0.48147177	0.67093764
Snx11	34	1.335288117	1.153246215	-0.182041903	0.48150772	0.67093764
Dmap1	8	2.15480564	2.314463416	0.159657776	0.48155604	0.670941257
Ccdc102a	61	1.633600162	1.387578107	-0.246022055	0.48192483	0.67126388

Psmb5	14	1.394574737	1.470641673	0.076066936	0.48185598	0.67126388
Zfp141	2	2.978723404	2	-0.978723404	0.48190498	0.67126388
Ctnna1	65	1.106223228	1.151799001	0.045575773	0.48199092	0.67129221
Elf2	281	1.302373173	1.246524078	-0.055849095	0.48209342	0.671371244
Lrpprc	32	1.181348938	0.904169335	-0.277179603	0.48221906	0.67148249
Wbp4	102	1.511261426	1.325945243	-0.185316184	0.48227075	0.671490744
Zbed6	19	1.864892476	1.530033485	-0.334858991	0.48241789	0.671576556
Angel1	31	1.490839582	1.481289616	-0.009549966	0.48242391	0.671576556
Esam	16	37.45040847	37.35250661	-0.097901862	0.48258743	0.671740466
Acbd6	53	1.34649715	1.370559458	0.024062308	0.48269423	0.671761676
Chd1	102	1.247400101	1.144606771	-0.102793329	0.48266756	0.671761676
Sncaip	86	41.85711338	41.68829564	-0.168817736	0.48291921	0.672002877
Arhgap18	5	1.491955551	1.932692833	0.440737282	0.48295913	0.672002877
Zfp593	40	1.535470301	1.287151434	-0.248318867	0.48310544	0.672142712
Prmt6	73	3.820843059	3.777171773	-0.043671286	0.48343826	0.672478241
Diap1	57	1.241563662	1.273870278	0.032306616	0.4834056	0.672478241
Zfp516	75	1.963925248	1.679531388	-0.28439386	0.48355787	0.672580868
Slc25a22	10	1.458888327	0.95260426	-0.506284066	0.48371871	0.672740812
Chchd5	22	1.33685271	1.667282133	0.330429422	0.48395575	0.672815419
Psmd9	18	2.027985909	2.170454399	0.142468489	0.48394523	0.672815419
Apol7d	22	1.298578286	1.179990193	-0.118588093	0.48395362	0.672815419
Fam179a	3	67.49341528	62.89469193	-4.59872335	0.48387122	0.672815419
Mfge8	22	1.882668639	1.893101828	0.010433189	0.48407289	0.672914511
Ifnar2	38	1.153758957	1.368843164	0.215084207	0.48421061	0.673042204
Sfxn1	67	1.147216087	0.923229286	-0.223986801	0.48463071	0.67356232
Birc3	16	1.707402418	1.549703657	-0.157698761	0.48470026	0.673595191
Noa1	31	0.928904765	0.867845845	-0.061058921	0.48482507	0.673652532
Tatdn2	105	0.90126438	0.929540129	0.028275748	0.48483334	0.673652532
Ccnd1	33	1.166784754	1.103050474	-0.06373428	0.48521605	0.674056644
Ppox	4	1.948339017	3.227998641	1.279659624	0.48521019	0.674056644
Tmx4	37	1.371770781	1.033717582	-0.338053199	0.48526399	0.67405943
Tnks2	54	1.26496997	0.97771771	-0.287252261	0.48538104	0.674093637
500015A07R	9	2.051655415	2.747717863	0.696062449	0.48565612	0.674093637
Gm21992	8	1.954743955	1.672282675	-0.28246128	0.48550329	0.674093637

Rbm14	8	1.954743955	1.672282675	-0.28246128	0.48550329	0.674093637
Eps15	44	1.374575146	1.454414207	0.079839061	0.48559405	0.674093637
BC031181	18	1.953384381	1.637125574	-0.316258807	0.48565153	0.674093637
Ppm1g	36	1.056871414	1.247192587	0.190321173	0.48535587	0.674093637
Lgals3	11	0.917634223	0.921597801	0.003963578	0.48556989	0.674093637
Pus7	39	1.05776902	0.897075206	-0.160693814	0.48582706	0.674267118
)30047H15F	26	0.556873415	0.805905271	0.249031855	0.48592663	0.674341531
Gfod2	34	2.737375633	3.385797699	0.648422066	0.48597799	0.674346063
Churc1	19	1.179842104	1.266380118	0.086538014	0.48606776	0.674346063
Bola2	26	1.036778956	1.040426749	0.003647793	0.48606494	0.674346063
Ctnnb1	135	1.634044289	1.500994046	-0.133050243	0.4862164	0.67443925
Nup205	81	0.951419044	0.982532355	0.031113311	0.48622685	0.67443925
Pbxip1	2	4.651442308	6.486676017	1.835233709	0.48641399	0.674635054
Wsb2	54	1.622872384	1.287832979	-0.335039405	0.48648454	0.674669141
Dalrd3	19	8.747851728	9.189923101	0.442071373	0.48656666	0.67471925
Kif2a	138	1.198980999	1.127022494	-0.071958506	0.48668246	0.674752308
Abracl	17	2.297359096	1.60806174	-0.689297356	0.48666768	0.674752308
Zfp217	113	5.69345603	5.471719419	-0.221736612	0.48695062	0.675060311
Dgcr14	34	1.282518929	1.320938793	0.038419865	0.48711989	0.675231186
Pigf	21	0.904363034	0.845026098	-0.059336936	0.48741395	0.675511177
Rac1	93	1.131559181	1.079580866	-0.051978315	0.48738529	0.675511177
Cep57l1	30	1.296801424	1.320353982	0.023552558	0.48755476	0.675581149
Tsga10	101	1.292468073	1.258483146	-0.033984928	0.48755652	0.675581149
Rplp0	21	1.079652968	1.369261904	0.289608937	0.48772852	0.675691874
Cep192	39	1.399724739	1.222922667	-0.176802073	0.487718	0.675691874
Surf1	11	1.100419857	1.046130961	-0.054288896	0.48778411	0.675705087
Idh3a	44	1.348685392	1.290720455	-0.057964937	0.48792857	0.6758414
Insig2	37	1.745206286	1.199236025	-0.545970261	0.48805903	0.675923211
Map3k10	38	1.162653711	0.972999393	-0.189654318	0.48807976	0.675923211
Limd2	105	1.912993017	1.498072572	-0.414920445	0.48821145	0.676041785
Spcs3	72	1.044190813	0.955025408	-0.089165405	0.48839829	0.676046664
Edc3	13	1.785401734	1.978806801	0.193405067	0.48832603	0.676046664
Bcl2l13	21	1.075487148	1.037048119	-0.038439029	0.48841223	0.676046664
Inpp4b	16	2.304433704	1.724044726	-0.580388978	0.48843736	0.676046664

Zdhhc2	103	1.185482017	1.086794126	-0.098687891	0.48844533	0.676046664
AA474331	20	8.210615521	7.687075037	-0.523540484	0.4885544	0.676133852
Gramd2	2	31.61846596	28.519939	-3.098526955	0.48860712	0.676143047
Rpa2	37	1.411914146	1.193659224	-0.218254922	0.48890838	0.676338343
Btf3	77	1.060773758	1.176288823	0.115515066	0.48884211	0.676338343
Sesn1	77	1.548361176	1.508019436	-0.04034174	0.4889016	0.676338343
Hiatl1	18	0.914048574	0.838917431	-0.075131143	0.48893261	0.676338343
Ints10	18	2.277662529	1.85916097	-0.418501558	0.48903097	0.676394457
Rmnd5a	65	1.28126419	1.212732849	-0.068531341	0.48906537	0.676394457
Mtx3	14	6.691266194	6.178323556	-0.512942638	0.48920815	0.676528174
Mir6909	3	98.67000093	98.62538428	-0.044616649	0.48927856	0.676561779
Uri1	87	1.032963528	1.087688925	0.054725396	0.4893994	0.676665105
Vti1b	69	1.85883681	1.804197195	-0.054639615	0.48944881	0.67666966
Rabggta	26	1.653412635	1.364817187	-0.288595448	0.48975414	0.677028006
Hectd3	56	1.342846891	1.355407207	0.012560316	0.49043242	0.677901777
Ist1	47	1.672620084	1.719578566	0.046958482	0.49080304	0.678350176
Catsperd	4	11.15796042	10.21126761	-0.946692815	0.490944	0.678481089
Ralgapa2	53	0.992374721	0.883542984	-0.108831736	0.49099246	0.678484169
Ulbp1	1	58.58585859	54.19354839	-4.392310199	0.49127939	0.678816738
Sh3yl1	4	1.315789474	0.3125	-1.003289474	0.49136355	0.678861458
Sun2	78	1.26031483	1.185446595	-0.074868235	0.49140428	0.678861458
Zfp184	20	1.02805671	1.244459049	0.216402339	0.49150943	0.678942808
Thra	75	1.592262513	1.521172366	-0.071090147	0.49156854	0.678960544
Ucp2	36	1.882507942	1.526392572	-0.356115369	0.49202438	0.679526191
Asb8	51	1.490586416	1.292090214	-0.198496202	0.4922708	0.679536751
Snx9	133	0.991033525	0.818479089	-0.172554436	0.49224406	0.679536751
Mnat1	1	3.225806452	2.051282051	-1.1745244	0.49230988	0.679536751
Eif3j1	8	1.430695966	0.679483796	-0.751212171	0.49214377	0.679536751
Snrpf	8	1.76056338	1.181469298	-0.579094082	0.49208934	0.679536751
Akirin1	74	1.682609859	1.654200482	-0.028409376	0.49228318	0.679536751
Gch1	49	1.551761985	1.301756063	-0.250005922	0.4923628	0.679545879
Uba6	61	1.270899753	1.325478813	0.05457906	0.49265566	0.679677515
Tmem135	25	1.481247855	1.207231845	-0.27401601	0.49251777	0.679677515
Ska2	3	1.72201722	1.883465996	0.161448776	0.49262257	0.679677515

Zbed5	9	1.599024931	1.530271655	-0.068753277	0.49264025	0.679677515
Snhg6	25	1.053114034	1.158708592	0.105594558	0.49272888	0.679677515
300002E11R	5	2.442656019	1.851988719	-0.5906673	0.49273609	0.679677515
Gpr107	13	1.329736078	0.816205386	-0.513530691	0.49290395	0.679845147
Mir678	1	95.74468085	97.59036145	1.845680595	0.49324364	0.680185813
Tmem167	44	1.58885492	1.486378873	-0.102476047	0.4932092	0.680185813
Tmem183a	52	1.391891536	1.297840778	-0.094050757	0.49332938	0.680240111
Tbc1d7	32	1.430460134	1.501076652	0.070616518	0.49341524	0.680294583
Tbc1d23	13	1.712507122	2.388387346	0.675880225	0.49356277	0.68030623
Pard6b	52	1.327904328	1.270338938	-0.05756539	0.49355244	0.68030623
Tor1b	41	1.325223775	0.980783696	-0.344440079	0.49350142	0.68030623
Acox1	16	0.966873584	0.736461764	-0.23041182	0.49366102	0.680313841
Otulin	36	0.99000731	1.310089865	0.320082556	0.49364612	0.680313841
Lrwd1	52	1.875925376	1.841857951	-0.034067425	0.49374799	0.6803698
Ipo11	17	1.755328386	1.426111294	-0.329217092	0.49396193	0.680600691
Clk4	46	1.32897962	0.893203426	-0.435776194	0.49416116	0.680754241
Hspa9	82	1.271267023	1.123610242	-0.147656781	0.49416616	0.680754241
B4galt1	11	2.558562421	1.586946923	-0.971615499	0.49424592	0.68080021
Ptrf	25	2.089472399	1.745592067	-0.343880331	0.49440149	0.680925705
Shank1	19	5.484839991	6.744509377	1.259669386	0.49442983	0.680925705
Narf	31	1.646084522	1.585238202	-0.060846321	0.49452616	0.680994444
Gpc2	18	7.234295491	8.343523085	1.109227594	0.49473288	0.681215191
Ccdc28b	3	13.46186906	12.70891277	-0.752956285	0.49494394	0.681441865
Tpcn1	26	2.028095052	1.994320277	-0.033774774	0.4950783	0.681540633
Fth1	9	0.14199648	0.831550044	0.689553565	0.49510857	0.681540633
Synrg	56	1.60545106	1.43854318	-0.166907881	0.49530768	0.681750762
Nos1ap	105	1.398550611	1.360283864	-0.038266747	0.49545488	0.681889408
Chadl	4	78.42114641	79.86881559	1.447669184	0.49555641	0.681965187
Ptger1	2	42.19982724	39.29227941	-2.907547827	0.49611732	0.682673061
Ptpn9	47	0.873985392	0.997618742	0.12363335	0.49624153	0.682703222
Fancm	73	1.024221126	1.045230946	0.02100982	0.49623361	0.682703222
Ggnbp2os	25	1.860611242	1.60137745	-0.259233792	0.49632534	0.682703222
Rnpepl1	140	1.01488564	0.979740527	-0.035145113	0.49630264	0.682703222
Dennd1a	26	1.030627677	0.86717105	-0.163456627	0.49674777	0.683220238

Nt5dc1	27	1.852608484	1.82108128	-0.031527204	0.49688314	0.683342374
Crat	37	1.968393874	1.840445804	-0.12794807	0.49701647	0.683461688
Amn1	23	6.260839087	6.514414562	0.253575474	0.49721011	0.683663905
Ctdnep1	16	1.457797373	1.195720246	-0.262077126	0.49777535	0.684292718
Nbr1	3	0.490196078	0	-0.490196078	0.49779743	0.684292718
Hnrnpm	17	0.424836601	0.444977503	0.020140901	0.49780733	0.684292718
Ss18l1	42	0.8658014	0.621248789	-0.244552611	0.49786469	0.684307458
Tspan4	75	1.341870923	1.516862785	0.174991862	0.49813789	0.684490622
Egln2	67	1.870553892	1.477532007	-0.393021885	0.49807564	0.684490622
Mogs	65	0.947635375	1.015032276	0.067396901	0.49810308	0.684490622
Trafd1	49	1.272922911	1.039220629	-0.233702282	0.49835232	0.684721153
Triap1	20	2.070418789	1.826693314	-0.243725475	0.49848486	0.684736648
Nat10	43	1.812999694	1.530642684	-0.28235701	0.49847002	0.684736648
Irs2	103	0.956037561	1.018528888	0.062491327	0.49855025	0.684736648
Trmt2a	22	1.245255047	1.041236101	-0.204018947	0.4985259	0.684736648
Zfp706	74	1.269836841	1.194149776	-0.075687065	0.49863414	0.684787768
Rab6b	40	6.211497552	7.645591435	1.434093883	0.4987242	0.684847361
130026L21F	13	43.34221402	39.13810641	-4.204107606	0.49902057	0.685190218
Ccno	57	2.038768874	1.754678529	-0.284090345	0.49908783	0.685218456
Hmox1	19	1.867760123	2.071616144	0.203856021	0.49919142	0.68529656
Htra2	62	1.338200445	1.26995109	-0.068249355	0.49924133	0.685300965
Vps13d	20	0.503681821	0.672054805	0.168372984	0.49952213	0.685622276
Endog	15	1.206767543	1.432203542	0.225435999	0.50022233	0.68651912
Mipep	33	1.545516572	1.554894609	0.009378037	0.50042803	0.686672992
Fmn2	11	34.85873098	35.68767308	0.828942101	0.50040625	0.686672992
Selk	36	1.628538797	1.385146333	-0.243392464	0.50054951	0.686775453
Grcc10	5	1.880005671	1.147720154	-0.732285517	0.50090186	0.68719464
Ccdc130	33	4.379387304	4.546621393	0.167234088	0.50106273	0.68728683
)31434O11F	25	1.528236447	1.627808475	0.099572029	0.50106029	0.68728683
Ccng1	20	2.163106204	2.087965573	-0.075140631	0.50132072	0.687576435
Prkacb	80	1.538953932	1.421329258	-0.117624674	0.50154712	0.687758379
Rer1	69	1.112457227	1.092451886	-0.020005342	0.50151852	0.687758379
Bbs4	11	1.122115239	1.370709691	0.248594452	0.50168218	0.687879298
Papd7	211	1.103200929	0.999900329	-0.1033006	0.50188275	0.688090018

F2rl1	43	2.369269715	2.177211478	-0.192058237	0.50206606	0.688277034
Sh3rf1	68	4.18595368	3.423185944	-0.762767736	0.50224923	0.688306366
Taf7	35	2.153264463	2.234972415	0.081707952	0.50224679	0.688306366
Hcn3	46	4.678556292	5.072504513	0.393948221	0.50221635	0.688306366
)00057B20F	3	98.84057971	98.34494076	-0.495638948	0.50231257	0.688306366
Naa25	55	1.163437851	0.998540009	-0.164897841	0.50232199	0.688306366
AA414768	6	10.76123386	9.071773637	-1.689460226	0.50253919	0.688539689
)30405A21F	35	0.996090822	0.554844739	-0.441246083	0.50279959	0.688832156
Tfrc	27	2.155853005	1.641349045	-0.51450396	0.50285006	0.688836989
Ly75	20	1.547670932	1.509348607	-0.038322325	0.50293066	0.688883092
Mdn1	39	0.879818103	0.961857875	0.082039772	0.50312723	0.68908801
Agpat6	91	1.616725072	1.53678913	-0.079935942	0.50321013	0.689137227
Srebf2	29	1.236565539	1.378461287	0.141895747	0.50338241	0.689244521
Dtnbp1	91	1.075675417	1.072192304	-0.003483113	0.50335909	0.689244521
Taf5	23	1.796997123	1.654497003	-0.142500119	0.50365453	0.689552767
Ube2g1	86	1.010008447	1.079965649	0.069957203	0.50384611	0.689686368
Trim46	7	4.215273376	3.632954063	-0.582319313	0.50381805	0.689686368
Gm16712	3	0	0.617283951	0.617283951	0.50407621	0.689872631
Gm38431	10	1.125374591	0.872355336	-0.253019255	0.50407151	0.689872631
20014N23F	27	2.162656328	1.925131257	-0.237525071	0.50424658	0.689977088
Fbxo38	23	1.148331144	0.738114587	-0.410216558	0.50421012	0.689977088
Ebna1bp2	6	2.948548153	2.629911817	-0.318636336	0.504433	0.690100019
Gm20604	30	1.399489585	1.219351042	-0.180138543	0.5045894	0.690100019
Cfap57	6	2.948548153	2.629911817	-0.318636336	0.504433	0.690100019
430015G10F	17	1.102932581	0.915823968	-0.187108613	0.50464276	0.690100019
AK010878	30	1.399489585	1.219351042	-0.180138543	0.5045894	0.690100019
Atg14	28	1.261783521	1.436187083	0.174403562	0.50451597	0.690100019
Arpp19	59	1.197199097	1.129210647	-0.06798845	0.50466562	0.690100019
Cox20	77	1.119829445	1.062446364	-0.057383081	0.5047136	0.690101314
Il34	5	32.88870008	35.37432687	2.485626785	0.5049972	0.690424763
Polr2e	40	1.525272821	1.3453605	-0.17991232	0.50505463	0.690438951
Rnf32	7	0.216450216	0.458986175	0.242535959	0.50527191	0.690562384
Slc25a29	56	1.7171917	1.28047004	-0.43672166	0.50528611	0.690562384
Srsf10	126	1.334009245	1.225495611	-0.108513634	0.5052761	0.690562384

Plekhh1	75	1.634203221	1.345339212	-0.288864009	0.50547325	0.690689493
Slc22a23	127	1.614061635	1.326870979	-0.287190656	0.50545313	0.690689493
Tmem65	131	1.409789773	1.313987016	-0.095802757	0.50553431	0.690708609
700112J16R	13	2.687980384	2.011049131	-0.676931254	0.50559458	0.690726648
Eif3j2	48	1.439224799	1.311105556	-0.128119243	0.50607065	0.691248318
Plscr4	10	0.799138621	0.902547439	0.103408818	0.50606274	0.691248318
Ipo7	45	1.286438165	1.261092605	-0.02534556	0.50621642	0.691254398
Btd	15	2.378494706	2.034664424	-0.343830283	0.50617802	0.691254398
931414P19R	20	0.951503329	0.775282546	-0.176220783	0.50613114	0.691254398
Zfp473	15	2.655466235	2.162245883	-0.493220351	0.50631448	0.691259651
Get4	56	0.683507119	0.69001922	0.006512101	0.50630974	0.691259651
Hdgf	81	1.160443118	1.262574636	0.102131518	0.50665145	0.691655354
930509E16R	11	1.006466419	1.761023948	0.75455753	0.50677628	0.691761412
Khsrp	47	1.583762435	1.584615994	0.000853559	0.50693691	0.691916302
Katnbl1	61	1.705390832	1.778956192	0.07356536	0.50705536	0.691968225
Cct7	44	1.021729712	1.221512026	0.199782314	0.50706926	0.691968225
Ptgds	2	92.10526316	92.65328269	0.548019533	0.50712902	0.691985421
Lpin2	67	1.408715134	1.206397068	-0.202318066	0.50730484	0.692160968
Nlk	75	1.639914442	1.647736229	0.007821787	0.50740859	0.692238162
Tbrg4	69	1.682929982	1.484212545	-0.198717437	0.50770454	0.692577529
Utp11l	27	1.694109843	1.318905076	-0.375204767	0.5078764	0.692656254
Ralgapb	60	1.089247339	1.092832076	0.003584736	0.50792256	0.692656254
Rps8	19	1.187945872	0.982608263	-0.205337608	0.50794351	0.692656254
Abcc4	77	0.979315529	0.994270969	0.01495544	0.50795107	0.692656254
.10038B12R	19	1.582695745	1.591042485	0.008346739	0.50824658	0.692994828
Trim75	1	96.86098655	97.76536313	0.904376581	0.5083628	0.693007324
Trim68	24	1.222299973	1.103440393	-0.11885958	0.50831633	0.693007324
Rad50	36	1.187598594	1.142706806	-0.044891788	0.50842541	0.693007324
Ccdc25	30	1.114143495	1.048662299	-0.065481197	0.50844465	0.693007324
Anks1	56	1.368189676	1.35719942	-0.010990255	0.50860844	0.69316618
Particl	12	0.725763678	0.77899694	0.053233261	0.50874848	0.693292648
Rsrc1	23	1.132132315	1.01268309	-0.119449225	0.50886957	0.693328882
Trak2	18	0.882399443	0.880434035	-0.001965408	0.50883095	0.693328882
Sap30	34	1.012373371	0.587952978	-0.424420392	0.50912139	0.693519747

Ndufaf2	33	1.768088762	1.681456263	-0.0866325	0.50909851	0.693519747
Zcchc9	15	1.344873885	1.025005043	-0.319868842	0.50915144	0.693519747
Ptgs2	1	3.301886792	2.262443439	-1.039443354	0.50942056	0.693815647
Slc12a2	158	1.261873636	1.163027755	-0.098845881	0.50946324	0.693815647
Rnf149	81	1.081292192	0.841971409	-0.239320783	0.50969905	0.694061244
Map4	76	1.274576761	1.367781725	0.093204964	0.50973818	0.694061244
Chmp7	37	1.174875287	1.273543231	0.098667944	0.50988497	0.694193272
Heatr6	41	1.088097419	0.9375849	-0.150512519	0.50992976	0.694193272
Tnk2	89	1.998286333	1.866974869	-0.131311464	0.51018141	0.694471424
Erbb2ip	216	1.245138415	1.20298094	-0.042157475	0.51062214	0.694967553
Pvrl3	114	1.092438522	1.123710665	0.031272143	0.5106406	0.694967553
)33408J17R	16	2.002110457	1.375537686	-0.626572771	0.51082536	0.695154539
Lmo4	85	1.350201443	1.469092719	0.118891276	0.51096387	0.695214076
Cic	62	1.539751877	1.528574704	-0.011177173	0.51093623	0.695214076
Zfp119a	4	1.360544218	1.645397613	0.284853395	0.51135921	0.695687476
Dstyk	60	1.416675736	1.463071581	0.046395845	0.51167223	0.696048789
Ulk2	64	0.962329804	0.849717035	-0.112612769	0.51189299	0.696284557
AA415398	45	1.315274551	1.154769478	-0.160505073	0.51194326	0.696288391
Riok3	32	1.07333225	1.565746425	0.492414175	0.51216124	0.696391223
Gadd45g	29	1.181170011	1.128869219	-0.052300792	0.51214956	0.696391223
Rasd1	44	3.730095083	3.969852826	0.239757743	0.51213842	0.696391223
Strn	67	1.42826259	1.313779125	-0.114483465	0.5122975	0.696511955
Wdr83	22	1.787847191	2.094878813	0.307031622	0.51252459	0.69670874
Med26	51	1.135720376	1.071592915	-0.064127461	0.5125372	0.69670874
Ppip5k2	24	1.777336427	1.376492451	-0.400843976	0.51289581	0.697131634
Polr2c	27	1.132530074	0.790664806	-0.341865267	0.513063	0.697294288
Btc	7	2.852484293	2.254751657	-0.597732636	0.51311126	0.697295301
Ctage5	123	1.178310437	1.163500139	-0.014810298	0.51363034	0.697914253
Cep83	76	1.050490891	1.061887756	0.011396865	0.51370941	0.697914253
Papola	44	1.649334122	1.68970098	0.040366858	0.51368083	0.697914253
Ulk4	32	1.506670884	1.376144918	-0.130525966	0.51386301	0.69803688
Cyb561d2	3	1.025641026	1.485220954	0.459579928	0.51389481	0.69803688
Wdhd1	61	1.371297651	1.122122652	-0.249174999	0.51412044	0.698205367
Pigyl	57	1.924607168	1.736762784	-0.187844384	0.51410016	0.698205367

Asap2	67	9.208449399	9.532993606	0.324544207	0.51416159	0.698205367
Pik3r1	90	4.105759115	4.006454069	-0.099305046	0.51424669	0.698256314
L2hgdh	13	1.558782114	1.659319801	0.100537687	0.51433395	0.698310179
Ccdc105	9	21.47548201	24.89907347	3.423591463	0.51442803	0.698373289
Socs6	49	1.087617397	1.050301464	-0.037315933	0.51450782	0.698416992
BC020402	39	1.075264436	1.031295403	-0.043969033	0.51467247	0.698575883
510039O18F	67	1.6064892	1.445043069	-0.161446131	0.5148157	0.698705663
Mir7653	7	1.415729406	1.44609442	0.030365013	0.51498574	0.698742556
Gm13889	34	9.672396174	11.06555435	1.39315818	0.51493889	0.698742556
Ppp4r2	57	0.94898361	1.080817717	0.131834107	0.51491844	0.698742556
Ttc4	12	0.503017123	0.579101896	0.076084773	0.51518568	0.698826691
Cdca5	33	1.098547501	1.197908801	0.099361299	0.51519062	0.698826691
AI118078	48	1.457730099	1.241556279	-0.21617382	0.5151806	0.698826691
Rprd1b	28	0.760329547	0.869653654	0.109324108	0.51531082	0.698925133
Dmwd	105	1.289933095	1.468087422	0.178154327	0.51547856	0.69904171
Atp6v0c	29	1.482601469	1.359740007	-0.122861462	0.51549205	0.69904171
Hus1	20	1.269155131	1.655716641	0.38656151	0.51561356	0.699141879
Tecpr1	37	1.383961123	1.10659032	-0.277370803	0.51602446	0.699246744
Pdzd8	95	1.205689406	1.164811388	-0.040878018	0.51588572	0.699246744
Pdcd4	49	1.711552169	1.511277766	-0.200274403	0.51599311	0.699246744
Rab38	25	2.374314706	2.281362215	-0.092952491	0.51597239	0.699246744
Slc25a4	41	1.118810534	1.151626317	0.032815783	0.51597561	0.699246744
Jrk	42	1.59302355	1.508616847	-0.084406703	0.51600752	0.699246744
Iqcg	5	3.802677942	4.552255732	0.74957779	0.5159576	0.699246744
933434E20F	10	2.251900462	2.343192726	0.091292263	0.5163649	0.699643453
230208H11F	46	1.732980263	1.539135462	-0.193844801	0.51651598	0.699783533
Luc7l	20	1.378515143	1.402078027	0.023562884	0.51659309	0.69982339
Atg10	33	1.228323789	1.628524478	0.400200689	0.5169028	0.70017831
930455B14F	1	12.37113402	10.75268817	-1.618445849	0.51713221	0.700295131
Tiparp	126	1.341228288	1.278520867	-0.062707422	0.51709353	0.700295131
Thap1	42	1.369732208	1.201974144	-0.167758064	0.51710698	0.700295131
Ap2s1	18	1.804053521	1.329469108	-0.474584412	0.5174254	0.700498236
Rpe	21	1.388416932	1.4576952	0.069278268	0.51740452	0.700498236
Mir6975	8	2.097794025	2.088403298	-0.009390726	0.51737846	0.700498236

Slc15a4	52	0.993782474	0.781865894	-0.21191658	0.51771785	0.700764853
Psmb1	28	1.200055062	0.927345964	-0.272709099	0.51770218	0.700764853
Idua	17	1.817409895	1.225725902	-0.591683993	0.51833207	0.701531531
Helb	8	2.744183271	3.102908846	0.358725575	0.51864615	0.70189188
Mapk11	52	1.871869468	1.946748695	0.074879227	0.51888836	0.70215491
Dtd1	24	1.125412493	1.288305442	0.162892949	0.5189379	0.702157186
110004E09R	68	1.131429551	1.166702472	0.035272921	0.51900158	0.702178605
Rimbp3	1	34.93975904	31.81818182	-3.121577218	0.51936903	0.702462867
Agpat2	43	7.197321872	6.245365643	-0.951956228	0.51937572	0.702462867
Kctd19	4	35.79594836	34.72178165	-1.074166709	0.51934817	0.702462867
Ogfrl1	78	1.584525075	1.721933648	0.137408572	0.51940317	0.702462867
Inca1	57	1.615048183	1.436130517	-0.178917665	0.51973262	0.702843657
Stk25	64	1.468793364	1.260522679	-0.208270686	0.5198984	0.703003054
Slc10a7	14	0.767299159	0.731651635	-0.035647525	0.52025516	0.703379785
Mir6405	1	6.787330317	5.384615385	-1.402714932	0.52029353	0.703379785
Fxr2	59	1.179167854	1.037159436	-0.142008418	0.52032081	0.703379785
Stx17	45	1.492066944	1.255655497	-0.236411447	0.52044841	0.703487473
Dap	24	1.126786214	1.189932271	0.063146057	0.5206505	0.703695807
Epn1	57	1.915071023	1.491359532	-0.423711491	0.52082176	0.703797631
Plxna1	134	1.840960083	1.627631109	-0.213328974	0.52078127	0.703797631
Dhrs3	33	1.174611771	1.0633627	-0.11124907	0.52088261	0.703815041
Csrp2bp	36	1.182903766	1.458306102	0.275402336	0.52115074	0.704112506
Slc22a15	76	1.352158053	1.208731271	-0.143426782	0.52146472	0.704419739
)17H6S56E-!	19	0.598292099	0.723106549	0.12481445	0.52147415	0.704419739
Sec61b	4	0.668918619	0.962937368	0.29401875	0.52157755	0.704494564
30043M19R	46	1.402587593	1.241118046	-0.161469548	0.52171301	0.704612674
Tmem123	10	0.447761194	1.338957387	0.891196193	0.52225893	0.705090404
Cnot7	49	2.138217225	2.042150901	-0.096066324	0.52223069	0.705090404
Sptlc2	85	1.597936205	1.283764302	-0.314171903	0.5222078	0.705090404
Thap2	32	1.511305242	1.328654425	-0.182650817	0.52217192	0.705090404
Tle2	19	2.368138076	2.803099024	0.434960948	0.52241132	0.705231249
Parp2	5	2.715529753	3.521758198	0.806228445	0.52265007	0.705488648
Timmdc1	29	1.187397774	0.863779518	-0.323618256	0.52275076	0.705559662
Fibp	11	1.264158608	1.351211453	0.087052845	0.52296181	0.705649806

Abhd16a	45	1.202971946	1.161462049	-0.041509898	0.52293859	0.705649806
Sod1	41	1.43046835	1.165676092	-0.264792258	0.52292489	0.705649806
Rasa2	98	1.058838138	0.944175774	-0.114662364	0.5231462	0.705711211
Rfx7	83	1.20215954	1.279269577	0.077110037	0.52309863	0.705711211
Pdpr	50	1.161692188	1.053955603	-0.107736585	0.5231516	0.705711211
Specc1	73	2.118133621	1.752045835	-0.366087786	0.52350927	0.706128783
Rad17	60	1.202351636	1.09878374	-0.103567895	0.52357369	0.706150762
Tfpt	5	1.191757323	1.043133486	-0.148623837	0.5237995	0.706195687
Pemt	25	1.430938126	1.433431678	0.002493551	0.5237718	0.706195687
Hnrnpab	70	0.820244667	0.953057087	0.13281242	0.52374727	0.706195687
Nckap1	116	1.566934729	1.463823488	-0.103111241	0.52375376	0.706195687
Mrpl51	52	1.517312358	1.35357303	-0.163739327	0.52425606	0.7067463
Rufy4	41	1.198915935	1.279699333	0.080783399	0.52437327	0.706839372
Map3k5	141	1.030790854	0.949516261	-0.081274594	0.5244729	0.706908727
Mxd3	55	1.217291439	1.193869318	-0.023422121	0.52458924	0.707000595
Nmi	12	1.051352003	1.237642839	0.186290836	0.52473954	0.707138211
Actr3b	25	6.86338967	6.599182449	-0.264207221	0.52494246	0.707346709
Ehbp1l1	19	1.329714505	1.349524183	0.019809678	0.52506736	0.707450044
Cops6	4	1.557320919	2.587221199	1.02990028	0.52513911	0.707481759
Slc25a54	1	96.32352941	97.65625	1.332720588	0.52527716	0.70753783
Asic4	14	10.43878577	9.508575837	-0.930209928	0.52525235	0.70753783
Mir3083	87	1.4019345	1.432684052	0.030749552	0.52547203	0.707735338
Zfp207	2	0.980392157	1.948051948	0.967659791	0.52558356	0.707820593
Pcyt1a	77	3.455912142	3.249441356	-0.206470786	0.52572736	0.70794928
Dusp3	13	1.543816301	0.995624076	-0.548192225	0.52585588	0.708057371
Gm15800	28	1.828853271	1.848553292	0.019700021	0.526047	0.70818474
Capn10	25	1.576722354	1.5813391	0.004616747	0.52601131	0.70818474
Pak6	64	14.72801589	15.5008839	0.772868006	0.52626336	0.708280934
Apoo	4	26.75364248	25.25402325	-1.499619229	0.52634275	0.708280934
Emc10	29	0.831977978	1.078817616	0.246839638	0.52635979	0.708280934
Dnaja1	72	1.679503881	1.359027613	-0.320476268	0.52632645	0.708280934
Zfp758	5	1.511299435	1.799808429	0.288508994	0.52623885	0.708280934
Gstk1	20	13.42973691	12.86322319	-0.566513714	0.52641349	0.708288245
Chd1l	15	1.004516394	1.082782823	0.078266429	0.52649307	0.708330366

Comt	17	0.926487096	1.252328795	0.325841699	0.52663037	0.708450135
Prr22	2	80.94983656	80.1429733	-0.806863258	0.526832	0.708656407
Gm4532	64	1.271695421	1.256331707	-0.015363714	0.52723255	0.709130194
Vps26b	22	1.662169858	1.318995166	-0.343174692	0.52735345	0.709167825
Leng8	29	1.178214998	1.084498767	-0.093716232	0.52745384	0.709167825
Ticrr	35	1.598800102	1.527582138	-0.071217964	0.52742884	0.709167825
Mrpl36	10	1.948276858	2.821167623	0.872890765	0.52742832	0.709167825
Ddx18	9	1.655062273	1.717227112	0.062164839	0.52775589	0.709508931
Ermap	2	62.20960405	65.1935644	2.983960343	0.52795534	0.709665764
Chchd3	21	1.098912676	0.768064656	-0.33084802	0.52796928	0.709665764
Trim65	21	5.451665216	4.602852529	-0.848812687	0.52803302	0.709686444
Tcf19	29	1.376317312	1.595340367	0.219023055	0.52843637	0.709846422
Serp1	18	1.286244315	1.068894801	-0.217349513	0.5284423	0.709846422
Mbd5	4	3.376580173	5.492125984	2.115545811	0.52820376	0.709846422
Cox5a	93	1.177995187	1.111551591	-0.066443596	0.52838314	0.709846422
'00012C14F	16	51.54682971	51.67959797	0.132768251	0.52829298	0.709846422
Cchcr1	29	1.376317312	1.595340367	0.219023055	0.52843637	0.709846422
Gpatch3	57	2.941363677	3.67534772	0.733984043	0.52859482	0.709986308
Rnf166	85	1.317572752	1.178838372	-0.13873438	0.52875875	0.71014149
)30581F22F	22	1.167560959	0.808246347	-0.359314612	0.52884054	0.710186333
Timm9	44	2.058041768	1.615934913	-0.442106855	0.52891062	0.710215452
Mir1898	2	79.14473684	78.71794872	-0.426788124	0.52911906	0.710430325
Traf4	56	1.376513979	1.278417795	-0.098096184	0.52916791	0.710430915
Fbxw4	100	1.767948569	1.51514569	-0.252802879	0.52936215	0.710626669
Mcee	34	1.750694111	1.316677338	-0.434016773	0.52941331	0.710630345
Acsf2	21	3.347665453	5.063092843	1.715427391	0.52953969	0.710734963
Med9	24	0.692816396	1.07248344	0.379667044	0.52972635	0.710920469
Tsc1	34	1.532681482	1.5387751	0.006093618	0.53017794	0.711146031
Timeless	16	11.86766586	11.95457014	0.086904283	0.53005708	0.711146031
Mrps12	6	2.121601017	2.464496577	0.34289556	0.5300175	0.711146031
Zfand5	103	1.057195397	0.936324855	-0.120870542	0.5301852	0.711146031
Lyrm4	7	0.98912787	0.630335292	-0.358792578	0.53009178	0.711146031
Zfp101	16	1.013255526	0.957694374	-0.055561152	0.5301027	0.711146031
Gmps	56	1.267025517	1.323408596	0.056383079	0.53031357	0.711222429

Ythdc1	72	1.105244807	0.969877127	-0.135367681	0.53033909	0.711222429
Cadm2	17	35.68775285	36.17242046	0.484667605	0.53054396	0.711302153
Api5	80	0.900721836	0.83486244	-0.065859396	0.53050659	0.711302153
Tceb3	69	0.913558571	0.999432874	0.085874303	0.53050797	0.711302153
Mrpl49	14	1.152891062	1.092766356	-0.060124706	0.53061219	0.711328633
Uchl3	80	1.233623113	1.277690628	0.044067515	0.5310728	0.711881091
Pth2	2	32.31474408	29.82115923	-2.493584847	0.53121284	0.712003768
Dbi	24	1.275716217	1.047020835	-0.228695382	0.53136552	0.712143357
Vps26a	30	1.777924483	1.560626897	-0.217297586	0.53169765	0.712523405
Ncbp1	33	1.15002843	0.648076619	-0.501951812	0.53175604	0.712534801
933432I09R	1	95.65217391	96.90721649	1.255042582	0.53194894	0.712534801
Ubl5	7	1.980687326	1.979162769	-0.001524557	0.53192103	0.712534801
Stau2	66	1.45128958	1.361058364	-0.090231217	0.5318943	0.712534801
Pdcd11	25	0.963096598	0.891542449	-0.071554149	0.53188766	0.712534801
Dcaf17	15	0.906038897	0.922293995	0.016255098	0.53231412	0.712893807
310410L24R	25	1.063272794	1.151407389	0.088134594	0.53230591	0.712893807
Wrnip1	64	1.054641682	1.111842861	0.057201179	0.5323982	0.712941337
Tchp	21	0.956840058	1.048484823	0.091644764	0.53262536	0.713037876
Mtif2	18	2.678170143	2.606184392	-0.071985751	0.53257913	0.713037876
Dnajb9	49	1.068252452	0.94614957	-0.122102882	0.53252092	0.713037876
Ckap2	58	1.267091657	1.106895981	-0.160195676	0.53266466	0.713037876
Tti2	5	2.935903376	2.564661426	-0.37124195	0.53286666	0.713243221
Nup93	34	0.933176072	0.851026769	-0.082149304	0.53304586	0.713418003
Git1	105	1.078551649	1.156609225	0.078057577	0.53336484	0.713714728
Cdkn1a	12	1.847592627	2.018962139	0.171369512	0.53335892	0.713714728
Nr1h2	19	1.346681955	1.514982606	0.168300651	0.53346659	0.713785794
Ppapdc1b	12	1.345883431	1.397443095	0.051559664	0.53364729	0.71396247
Naa11	32	91.83692422	91.93072348	0.093799266	0.53381405	0.714055363
Rap1b	123	1.027545212	0.797793936	-0.229751277	0.53380291	0.714055363
Gprc5d	1	98.41897233	97.65625	-0.762722332	0.53386341	0.714056303
Taf6l	8	2.530047873	2.005777532	-0.524270341	0.53393562	0.7140878
Homer1	135	1.333093253	1.195856862	-0.137236391	0.53404265	0.714165843
Qsox2	30	0.673515326	0.511590795	-0.161924531	0.53414151	0.714232955
Wasf1	73	1.058812113	1.002004787	-0.056807325	0.53432013	0.714406708

430016H08R	28	1.894433567	1.434574412	-0.459859154	0.53442562	0.714482645
Ict1	44	1.655096	1.362329152	-0.292766848	0.53460265	0.71452402
Naa20	68	1.27694711	1.119626782	-0.157320328	0.53452465	0.71452402
Gng2	5	62.11290456	60.93334548	-1.179559085	0.53458315	0.71452402
Mier1	117	0.958927782	0.985182549	0.026254766	0.5349432	0.71478387
Mina	29	1.63652943	1.671091868	0.034562438	0.53489185	0.71478387
Plxdc1	32	3.796707271	3.274530984	-0.522176287	0.53491584	0.71478387
Farp2	30	1.061052229	1.151166608	0.090114379	0.53554254	0.715454414
Cope	11	0.825705126	0.915145841	0.089440715	0.53552571	0.715454414
Mtmr10	55	1.214072691	1.322574967	0.108502276	0.53592083	0.71589461
Filip1l	57	1.443143668	1.188360501	-0.254783166	0.53607792	0.715974101
Ndst1	15	92.8496459	91.81685121	-1.032794687	0.53606163	0.715974101
930512B01R	4	1.619187264	1.169851036	-0.449336228	0.53646621	0.716427476
Ago2	114	1.085264293	1.26211711	0.176852818	0.53693855	0.716993019
Psat1	31	1.324129952	1.158945933	-0.165184019	0.53705345	0.717057878
Gapdh	15	2.071984034	2.280148574	0.20816454	0.53708485	0.717057878
Rxra	166	1.433692089	1.306422236	-0.127269853	0.53716532	0.717100058
Fam219b	35	1.222555124	1.354551697	0.131996573	0.53766347	0.717699786
Pih1d2	45	1.354898956	1.195706333	-0.159192623	0.53773693	0.717732552
Il20ra	25	13.22309284	13.31050111	0.087408267	0.5380423	0.718074827
Smarcc1	84	1.17019956	1.438554163	0.268354603	0.53826406	0.718240134
Ppm1b	48	0.868803536	0.760727475	-0.108076061	0.53821813	0.718240134
Snapc2	27	0.641423624	0.773005769	0.131582145	0.53843701	0.718405597
Ddx23	27	1.352023971	1.788531302	0.436507331	0.53864285	0.718599902
Coa5	15	1.238323328	1.231546118	-0.00677721	0.53868059	0.718599902
Mir1894	2	0.403225806	1.219512195	0.816286389	0.53877639	0.718662367
Phospho1	79	2.688732869	2.47495338	-0.213779489	0.53903464	0.718745506
Slc25a44	2	1.842105263	2.443991853	0.60188659	0.53900932	0.718745506
Snord68	65	1.549016761	1.541794106	-0.007222655	0.53893036	0.718745506
Pfdn5	11	2.79452963	2.893648917	0.099119287	0.53900541	0.718745506
Rps6ka2	55	1.511298502	1.465375798	-0.045922704	0.53940024	0.71903698
Tbpl1	119	1.442473126	1.407553238	-0.034919889	0.53939075	0.71903698
Kif21b	33	2.615901378	3.134367254	0.518465876	0.53935373	0.71903698
Rab1	45	1.401783108	1.475822085	0.074038977	0.53945414	0.71904352

Styx	25	1.0095105	0.954303649	-0.055206851	0.53953231	0.719082386
Rpl27a	4	3.17953568	2.533083211	-0.646452468	0.5398569	0.719319005
Atad3a	43	1.409290571	1.402530894	-0.006759677	0.53982563	0.719319005
Snora3	4	3.17953568	2.533083211	-0.646452468	0.5398569	0.719319005
Sox13	48	0.819663678	0.908677294	0.089013616	0.53995028	0.719378097
Shisa4	12	2.161603766	1.501938346	-0.659665421	0.54013103	0.71955358
Ralgps2	68	0.990541655	0.839443395	-0.15109826	0.54026818	0.719670959
)30467K11F	17	1.135011337	0.679562601	-0.455448736	0.54039175	0.719770221
Pbld2	25	1.371719432	1.294825498	-0.076893934	0.54051464	0.719868568
Gpr135	40	16.55775266	16.7831955	0.225442835	0.5406591	0.71993321
Zmym1	19	0.715442698	0.838153138	0.12271044	0.54071037	0.71993321
Mpnd	39	1.273899921	1.15926788	-0.114632041	0.54067465	0.71993321
Dars2	12	1.54378462	1.282053914	-0.261730706	0.54090732	0.72013011
Agbl3	51	1.022151171	0.850324268	-0.171826903	0.54105673	0.720263678
Hist1h1d	4	2.484238357	1.784989066	-0.699249291	0.54115836	0.72033361
Rwdd1	65	1.436455517	1.121920597	-0.314534921	0.54141774	0.720416064
Trp53bp2	82	1.100627937	1.123277463	0.022649526	0.54162126	0.720416064
Snora7a	32	1.104786091	1.262119093	0.157333001	0.54166215	0.720416064
Prpf3	45	1.130547961	1.097490681	-0.03305728	0.54157889	0.720416064
Rpl32	32	1.104786091	1.262119093	0.157333001	0.54166215	0.720416064
Mum1	19	1.851005588	1.14273432	-0.708271268	0.54162409	0.720416064
Cenph	20	1.297397405	1.499527584	0.202130179	0.54159839	0.720416064
Mgst3	5	1.052631579	1.271754983	0.219123404	0.54152835	0.720416064
310010H24F	89	1.170351166	1.067603374	-0.102747791	0.54134014	0.720416064
Mdh1	11	1.197063614	1.114838346	-0.082225268	0.54182458	0.720500332
Golim4	28	1.856815947	1.349165035	-0.507650912	0.54191907	0.720500332
Ccdc112	56	1.916506019	1.773064001	-0.143442018	0.54182782	0.720500332
Rtn4ip1	6	1.204285935	1.290751179	0.086465244	0.54192191	0.720500332
Mus81	7	2.066638452	1.646232524	-0.420405928	0.54218213	0.720650408
BC055324	8	1.215519643	1.450324792	0.234805149	0.54210896	0.720650408
Fam208a	84	3.353877767	3.229780085	-0.124097682	0.54213375	0.720650408
Ski	215	1.325246322	1.259222096	-0.066024226	0.5423758	0.720682949
Zbtb9	57	0.832034746	1.067965321	0.235930575	0.54231881	0.720682949
Rpp25l	47	1.412734467	1.354770195	-0.057964272	0.54235537	0.720682949

Ddi2	66	0.997941474	0.937503346	-0.060438128	0.54240306	0.720682949
Mir8102	42	1.442390891	1.468644064	0.026253173	0.5425952	0.720872971
Tcf20	12	19.93668815	21.55795475	1.621266603	0.54286411	0.721164938
Noc3l	34	1.317464724	1.432384561	0.114919837	0.54311085	0.721427416
Arhgap26	87	1.650263581	1.400096004	-0.250167577	0.54330489	0.721565016
Ak6	86	1.119699684	1.146833291	0.027133606	0.54331279	0.721565016
Hnrnpf	191	1.868843792	1.805867694	-0.062976098	0.54355094	0.721815968
Kirrel3	37	2.340965497	2.021845674	-0.319119823	0.54379264	0.722001044
Wasl	69	1.067310079	0.869576068	-0.197734011	0.54378718	0.722001044
Rnf215	20	1.666535557	1.18937337	-0.477162187	0.54383791	0.722001044
Copb2	22	1.533029093	1.647264395	0.114235302	0.54393403	0.722054071
Cc2d1b	85	1.294797444	1.37869734	0.083899896	0.54402547	0.722054071
Slc37a3	31	1.804761123	1.460889248	-0.343871876	0.54401967	0.722054071
Kpna3	87	0.928634708	0.936188215	0.007553506	0.54407781	0.722058225
Adck3	45	2.356558124	2.773662444	0.41710432	0.54449331	0.722544297
Gm7444	1	92	89.32038835	-2.67961165	0.54480774	0.722830806
Atg13	7	1.47023733	0.668589354	-0.801647977	0.54478952	0.722830806
Gatb	8	2.385159596	2.068471661	-0.316687936	0.54495993	0.722902004
Fam162a	31	0.645265785	0.456563883	-0.188701902	0.54493049	0.722902004
Afap1	35	1.624768468	1.368040261	-0.256728207	0.54520728	0.723164748
Rassf7	49	1.436725312	1.47845315	0.041727838	0.54533147	0.723198728
Dtymk	16	0.830299721	0.960462642	0.130162921	0.54530377	0.723198728
Ptma	95	1.207245656	1.207172806	-7.29E-05	0.54541852	0.723248812
Rps26	20	1.395017601	1.297275839	-0.097741761	0.54561079	0.723394379
Dcdc2a	68	40.99356588	42.9820409	1.988475014	0.54567619	0.723394379
Hmgn1	78	1.302356316	1.358715625	0.056359309	0.54563593	0.723394379
Ndc80	39	0.751349993	0.807683345	0.056333352	0.54582944	0.723532179
Ccs	10	1.144447184	0.531392763	-0.613054421	0.54599459	0.723685714
Mcmbp	105	1.077038345	0.96876709	-0.108271255	0.54609275	0.723750452
N4bp1	41	0.820047823	0.763831027	-0.056216796	0.54647133	0.724186789
Zfp316	54	1.625899326	1.638480854	0.012581528	0.54653526	0.724206099
Gcn1l1	69	1.520473911	1.341658748	-0.178815162	0.54669661	0.724223707
Arrdc2	9	3.718503719	2.60941982	-1.109083898	0.54660654	0.724223707
Rab23	76	1.155834214	1.220697406	0.064863192	0.5466844	0.724223707

Erh	40	1.378499446	1.495798864	0.117299419	0.54680641	0.724303783
Rnf168	53	1.005149854	1.036907406	0.031757552	0.54687026	0.724322978
'00123L14F	1	93.86503067	95.39473684	1.529706167	0.54696622	0.72438469
Picalm	82	1.520147201	1.139788552	-0.380358649	0.54704367	0.724390584
Atp6v1a	29	0.894510188	1.029831237	0.135321049	0.54706941	0.724390584
Zfp943	13	1.579206219	1.070526085	-0.508680134	0.54721953	0.724470868
Mcl1	101	0.953903499	0.899729658	-0.054173841	0.54732752	0.724470868
Map4k4	174	1.497812577	1.386088898	-0.111723679	0.54723604	0.724470868
Cacnb2	35	11.77932598	11.83171183	0.052385852	0.54728216	0.724470868
Gm16386	4	1.026992902	1.608233873	0.581240971	0.54737723	0.724471322
Eaf2	58	1.439551935	1.769407245	0.329855311	0.5475604	0.724648389
Spata2l	3	0.925925926	0.808080808	-0.117845118	0.54777779	0.724814388
Ksr1	35	0.91732044	0.563377654	-0.353942787	0.54778462	0.724814388
Zfp282	56	1.33872689	1.065818277	-0.272908613	0.5478982	0.724899306
Ralbp1	78	1.483639883	1.661313265	0.177673382	0.54833864	0.725351226
Wash1	22	2.857254641	2.76387946	-0.093375181	0.54832075	0.725351226
Hyou1	90	1.191071286	1.055645317	-0.135425969	0.54845885	0.72540857
Hmg20a	15	0.85311461	0.696134	-0.15698061	0.54853029	0.72540857
Me1	66	1.535009277	1.74466958	0.209660303	0.54851353	0.72540857
Rpl18	40	1.036448451	1.247878427	0.211429977	0.54865466	0.725507659
30072M11I	38	2.724111576	2.37101469	-0.353096885	0.54876618	0.725589744
Ehmt1	83	1.201043053	1.101710051	-0.099333001	0.54888732	0.725684524
Mageb3	33	1.888211684	1.722964824	-0.16524686	0.54900253	0.725771453
Elac1	84	1.270726253	1.070305222	-0.200421031	0.54911519	0.725855
Prss36	2	79.33921424	77.5363536	-1.802860643	0.54922158	0.725930232
Plp2	6	16.01863848	16.95496638	0.936327904	0.54929645	0.725963802
Jak3	5	1.582816011	1.116351329	-0.466464682	0.54939277	0.726025708
Cdpf1	21	1.733445427	1.601525189	-0.131920238	0.54968776	0.72615567
Nhp2	24	1.593788165	1.519084323	-0.074703842	0.54973854	0.72615567
Tbc1d10c	18	4.216669221	4.589549027	0.372879805	0.54956487	0.72615567
Gtf2b	42	0.929956862	0.822675271	-0.107281591	0.5496897	0.72615567
Snora16a	5	0.720779221	0.997245179	0.276465958	0.54961612	0.72615567
Cox11	37	1.262050304	1.090519483	-0.171530821	0.54991329	0.726306878
Tbc1d5	58	1.209510995	1.184754977	-0.024756018	0.54997895	0.726306878

Klf11	86	1.228254721	1.345268258	0.117013537	0.5500015	0.726306878
Efna4	26	1.354717236	1.506842146	0.15212491	0.55026285	0.726554043
Hmbs	20	1.278412495	1.038677989	-0.239734506	0.55028769	0.726554043
Tipin	33	0.763416773	1.013455716	0.250038943	0.55056415	0.726853646
Zfp369	18	0.8040249	1.034817024	0.230792123	0.55082854	0.727044775
Galnt4	58	2.036495271	1.95971565	-0.076779621	0.55084013	0.727044775
Ddx41	40	1.516602754	1.30852526	-0.208077493	0.55087719	0.727044775
Rhou	96	1.578652443	1.535135666	-0.043516777	0.5509071	0.727044775
Hhex	87	1.581877809	1.462527081	-0.119350728	0.55102098	0.727129666
Mipol1	32	1.13897455	1.069130208	-0.069844343	0.55110802	0.727144918
Gm10532	2	78.75362319	81.03881279	2.285189597	0.55113165	0.727144918
Nifk	15	0.49758238	0.555180272	0.057597892	0.55151874	0.727590217
Dhfr	73	1.399817275	1.265035037	-0.134782238	0.55173322	0.727807737
Slc23a2	80	1.216578498	1.187269656	-0.029308842	0.55232243	0.728454011
330133O14R	12	1.037269047	0.730020513	-0.307248534	0.55229435	0.728454011
Arrb1	48	1.040510301	0.925179128	-0.115331172	0.55243181	0.728512175
Mad2l1bp	14	1.788641123	1.749013248	-0.039627875	0.55246582	0.728512175
Phtf2	112	1.130766371	0.973759639	-0.157006732	0.55256114	0.728572392
Lrrc1	36	1.530707314	1.759735979	0.229028665	0.55270285	0.728624132
Mrps22	9	2.175256458	1.701339666	-0.473916792	0.55274725	0.728624132
Dennd5a	69	1.270909276	1.054240538	-0.216668739	0.55274934	0.728624132
Szrd1	7	2.303072593	1.479654149	-0.823418443	0.55282212	0.72863713
Irx4	95	35.54578904	36.62557813	1.079789098	0.55285851	0.72863713
Cacna1b	45	37.15110266	37.78559026	0.634487607	0.55295803	0.728702846
Fam120a	115	1.298212048	1.173748706	-0.124463343	0.5533659	0.729174858
Ccdc94	16	0.754157138	0.812295106	0.058137968	0.55374687	0.729611343
Pcnt	47	1.250463426	1.187487201	-0.062976225	0.55386518	0.729636205
Prc1	24	1.142507748	0.855638609	-0.286869139	0.55383676	0.729636205
Abcb6	9	0.182149362	0.633244589	0.451095226	0.55398068	0.729638942
Eri1	70	0.905935973	0.822834545	-0.083101428	0.55407202	0.729638942
Atp5k	23	3.356209024	3.656901221	0.300692197	0.55409191	0.729638942
Spty2d1	17	1.419193353	1.270093096	-0.149100257	0.55393288	0.729638942
Mrps24	73	1.159125291	1.133195619	-0.025929672	0.55411588	0.729638942
Exo5	15	1.607274522	1.603166574	-0.004107948	0.55430165	0.729818069

Spin2c	4	22.91700896	21.71338966	-1.203619305	0.55463558	0.73019222
Mapkbp1	12	1.3675073	1.252181329	-0.115325971	0.55485128	0.730267805
Gm14023	8	2.172030388	2.044300657	-0.127729731	0.55489206	0.730267805
Mxra7	54	17.30518724	17.6783113	0.373124061	0.55485033	0.730267805
Klhl26	46	1.460615865	1.518029464	0.057413598	0.55474957	0.730267805
Atad1	41	1.555290794	1.58908425	0.033793455	0.55526373	0.730691414
Methig1	3	47.4796748	48.66180049	1.18212569	0.55552388	0.730837141
Ube2cbp	54	1.181347958	1.287010789	0.105662831	0.55551756	0.730837141
Mettl7a2	3	47.4796748	48.66180049	1.18212569	0.55552388	0.730837141
Snx2	56	1.782117545	1.474799932	-0.307317612	0.55568462	0.730976257
Cd82	14	4.058638453	4.036098124	-0.022540329	0.55572926	0.730976257
Smim13	16	1.96336021	1.91142367	-0.05193654	0.55656268	0.731875683
Zfp39	27	1.727995747	1.68020744	-0.047788307	0.55650918	0.731875683
Cpsf3	7	1.528386834	1.430458005	-0.097928829	0.5565556	0.731875683
Rab7	72	9.707455073	9.270273949	-0.437181124	0.55684677	0.732062834
Prr5	113	2.21920635	2.0625719	-0.15663445	0.55677215	0.732062834
Zfp9	14	1.178234147	0.785264841	-0.392969306	0.55685466	0.732062834
Ncapg2	37	1.792570158	1.593815247	-0.198754912	0.55702968	0.732227316
Trim13	5	94.28301573	92.94771922	-1.33529651	0.55715349	0.732324462
Arl8a	88	1.110292313	1.036835528	-0.073456785	0.5573423	0.732507022
700028I16R	31	1.55658895	1.47593667	-0.08065228	0.55740256	0.732520615
Nudt18	53	1.41537198	1.489242281	0.073870302	0.55755901	0.732594994
Cbx2	80	1.187380051	1.137626359	-0.049753692	0.55755506	0.732594994
Adrm1	97	1.233490953	1.020868567	-0.212622387	0.55783266	0.73270547
Fgf23	3	28.65208041	29.17575758	0.523677164	0.55776627	0.73270547
Gli2	51	22.954823	23.24113361	0.286310604	0.5578205	0.73270547
Pdk1	33	0.971430114	0.887034428	-0.084395686	0.55784282	0.73270547
Ift52	26	1.525110465	2.161863048	0.636752582	0.55809657	0.732973159
Lrrk1	33	1.581408489	1.352414114	-0.228994375	0.55818149	0.733019074
Wrap53	3	0.980392157	0.396825397	-0.58356676	0.55824453	0.733036263
Park2	17	0.618648855	0.736432334	0.117783479	0.55842399	0.7332063
Gng11	15	12.82793359	14.78324065	1.955307062	0.55862537	0.733405087
Gcnt1	21	14.71129899	14.87821378	0.166914787	0.5588146	0.733587887
E2f8	113	1.296909243	1.239196211	-0.057713032	0.55900211	0.733702774

Hmga2	30	1.933384181	2.095580532	0.16219635	0.55900159	0.733702774
Srp68	29	1.55431694	1.7659339	0.211616959	0.55926955	0.733968448
Phf7	25	0.992049059	0.550959014	-0.441090046	0.55930456	0.733968448
Gnb1l	74	1.14843538	1.137879427	-0.010555952	0.55935744	0.733972195
)30445N18F	16	1.986141388	2.354864592	0.368723204	0.55947122	0.734055861
Pot1a	6	2.069207993	2.330986752	0.261778759	0.55965309	0.734097557
Rbbp8	48	1.018527724	0.90537185	-0.113155874	0.55961066	0.734097557
Ccdc57	11	1.78857923	1.261198481	-0.527380749	0.55957198	0.734097557
Cactin	80	0.861747975	1.003416654	0.141668679	0.55972431	0.734125361
Lnx2	11	1.443780938	1.175635958	-0.26814498	0.5598147	0.73417829
Magi1	69	1.146868828	1.233645761	0.086776933	0.55987962	0.734197819
Impad1	30	1.211853573	1.213436375	0.001582801	0.56042166	0.734646006
Elp6	25	1.352202311	1.88880167	0.536599359	0.56041733	0.734646006
Mir7228	1	98.59943978	99.07692308	0.477483301	0.56036177	0.734646006
Eif3h	35	1.305287932	1.477611163	0.172323231	0.56032549	0.734646006
Mast3	43	1.442167746	1.472046298	0.029878553	0.56072049	0.734972089
Zfp358	36	1.77971658	2.071844409	0.292127829	0.56108601	0.735385507
730017L22F	15	1.52802501	1.054965054	-0.473059956	0.56115905	0.735415554
Ddx20	89	1.234512678	1.223498765	-0.011013914	0.56137104	0.735627665
Nrf1	73	1.255098311	1.140397936	-0.114700375	0.56142657	0.735634743
.30023H24F	64	1.551718497	1.42033818	-0.131380317	0.56158445	0.73577591
Dazap1	86	0.962728577	1.040694454	0.077965877	0.56173808	0.735873405
Irx6	21	41.46773451	40.18973841	-1.277996104	0.56175916	0.735873405
Brd2	51	1.615662638	1.555034411	-0.060628227	0.56188042	0.735900858
Ccdc157	11	1.085192945	1.275771805	0.19057886	0.56184253	0.735900858
Asap1	150	1.082204405	1.133877381	0.051672976	0.56205796	0.735912353
Smarcd1	58	3.843145644	3.980274531	0.137128887	0.56196019	0.735912353
Mettl8	7	0.64516129	0.636765449	-0.008395841	0.56208979	0.735912353
Sdccag8	44	1.804022631	1.275148926	-0.528873705	0.56199913	0.735912353
Eif2s2	24	1.568895375	1.463363921	-0.105531454	0.56233969	0.735928069
Calr3	8	1.085467049	1.311815942	0.226348893	0.56238683	0.735928069
Il6	2	5.039929427	5.427823454	0.387894027	0.56241547	0.735928069
Mcm3ap	16	0.986010517	0.768549765	-0.217460753	0.56222401	0.735928069
Pigk	21	1.063908486	1.109045077	0.045136591	0.56238966	0.735928069

Pgd	34	1.229364813	1.553100624	0.323735811	0.5622588	0.735928069
Scrib	120	1.353085652	1.089382454	-0.263703198	0.56245286	0.735928069
Mir1904	68	1.284513663	1.007862756	-0.276650907	0.56253385	0.735968411
Dcp1b	8	2.47635743	1.639864965	-0.836492465	0.56265262	0.735996611
Tpm3	33	0.998003574	0.974774988	-0.023228585	0.56265572	0.735996611
Mcat	46	1.425463845	1.356138866	-0.069324978	0.56285174	0.736142975
Mir425	2	74.9092559	77.8092006	2.899944705	0.56286794	0.736142975
Pnoc	4	97.09659628	96.00362339	-1.092972893	0.56312203	0.736409654
Rab11b	36	1.408848525	1.28122238	-0.127626145	0.56325592	0.736411653
Ifih1	20	2.160031361	1.90712199	-0.252909371	0.56327412	0.736411653
Rpusd3	15	2.220177937	2.113148782	-0.107029155	0.5632379	0.736411653
Srxn1	56	1.436145997	1.378472998	-0.057672998	0.56334664	0.736440854
Oser1	98	1.061330983	1.045983497	-0.015347487	0.56356688	0.736515137
Dpm1	61	1.176489838	1.393504518	0.21701468	0.56395329	0.736515137
Fchsd1	38	2.729451064	2.680582824	-0.04886824	0.56385184	0.736515137
Tmem217	20	2.192372878	2.572005468	0.379632589	0.56384968	0.736515137
Srrm2	62	1.159431555	1.076640574	-0.082790981	0.56362217	0.736515137
Tldc1	1	98.61111111	97.26027397	-1.350837139	0.56395557	0.736515137
Pum2	114	1.303251597	1.253667753	-0.049583843	0.56357926	0.736515137
Cox6b1	14	1.418339873	1.896573467	0.478233594	0.56375033	0.736515137
Zfp395	114	1.25181522	1.24737053	-0.00444469	0.56390631	0.736515137
Dpm1-adnp	61	1.176489838	1.393504518	0.21701468	0.56395329	0.736515137
Letm1	66	1.156078458	0.919445835	-0.236632623	0.56371082	0.736515137
Mrps2	58	1.282705928	1.334863369	0.052157441	0.56420783	0.736779
Fbln2	25	2.202812752	1.945387252	-0.2574255	0.56435318	0.736780958
Ranbp10	17	0.982203969	1.107449433	0.125245463	0.56435995	0.736780958
Dpm2	21	2.29510365	1.865345307	-0.429758343	0.56430583	0.736780958
Setd6	25	1.433703349	1.176312232	-0.257391117	0.56451246	0.736783407
Spice1	41	1.418266294	1.188784968	-0.229481326	0.56445297	0.736783407
Nab1	115	1.404000313	1.343381132	-0.060619181	0.56447488	0.736783407
Prn	5	1.926780301	2.326508101	0.399727799	0.56480528	0.736907043
8-Sep	44	1.223396496	1.09809883	-0.125297666	0.5646925	0.736907043
Seh1l	111	1.101070271	1.09451692	-0.006553351	0.56480806	0.736907043
Prnp	5	1.926780301	2.326508101	0.399727799	0.56480528	0.736907043

Rmdn2	27	1.445069387	1.517086349	0.072016963	0.56497346	0.737057305
Mir7238	12	1.320260082	1.24474817	-0.075511912	0.56520323	0.737094936
Dtwd2	64	3.222839335	2.96060912	-0.262230214	0.56519473	0.737094936
[illegible]30480K23R	28	1.276090062	1.356418623	0.080328561	0.56520065	0.737094936
Ttc39a	30	8.108291917	7.978330334	-0.129961582	0.56518565	0.737094936
Lrrc49	12	1.411358687	1.130080926	-0.281277761	0.56539301	0.737211395
Cdc5l	58	1.246737572	1.25777852	0.011040947	0.56535076	0.737211395
Pick1	34	1.528524261	1.337449176	-0.191075085	0.56563932	0.737430807
Psmg1	34	1.045657749	0.946948518	-0.098709232	0.56566179	0.737430807
Alg9	47	1.129168055	1.188440281	0.059272226	0.56575501	0.73748681
Gpd1l	49	1.282074203	1.273350844	-0.008723359	0.56581331	0.737497293
Csnk1g2	30	1.247448704	1.171652494	-0.075796209	0.56591345	0.737562309
Icosl	55	1.781081372	2.110581744	0.329500372	0.56600329	0.737592686
[illegible]10009O20R	3	2.140672783	1.380670611	-0.760002171	0.56603729	0.737592686
Fam206a	3	2.112676056	2.066207801	-0.046468256	0.56615624	0.737676214
Rap2a	114	1.018756209	0.881446066	-0.137310143	0.56622855	0.737676214
Csrp1	21	1.274499059	1.06116219	-0.213336869	0.56626188	0.737676214
[illegible]10039H08R	22	1.73033753	1.638881463	-0.091456067	0.56630248	0.737676214
Itpk1	93	1.070355951	1.041810589	-0.028545362	0.56658704	0.737981384
Tmem214	37	1.892936798	1.299083503	-0.593853295	0.56721251	0.738730485
Pisd-ps2	4	0.488087708	0.966708023	0.478620315	0.56740652	0.738917578
Vta1	21	1.053774054	0.977954026	-0.075820028	0.56756168	0.739054047
Dpcd	5	0.465179175	1.046326484	0.581147309	0.56763448	0.739083267
Ybey	8	0.595238095	0.771604938	0.176366843	0.56770092	0.739104186
Lcp1	10	1.550989176	1.679795972	0.128806796	0.56834282	0.739808616
Fip1l1	40	1.290270017	1.46910851	0.178838493	0.56832626	0.739808616
Tnfrsf1a	23	2.08127217	1.967254069	-0.114018101	0.56851033	0.739961021
Ptgr1	2	3.887778888	8.333333333	4.445554446	0.56911545	0.740682943
Cpne2	79	1.234656348	1.244025211	0.009368863	0.5692205	0.740753954
Memo1	137	1.208025406	1.119264831	-0.088760575	0.56930171	0.740793948
Trpc3	86	12.95128739	13.81115533	0.859867939	0.56952836	0.741023154
Itch	54	1.328775265	1.240035757	-0.088739508	0.56998012	0.741545201
Ift46	11	1.437465764	1.412740984	-0.02472478	0.57030841	0.741872261
Depdc1b	68	1.257683662	1.179290797	-0.078392865	0.57033263	0.741872261

Pmepa1	98	1.299715353	1.299441778	-0.000273574	0.5705956	0.742148541
Larp7	6	1.170906719	0.79213816	-0.378768559	0.57100175	0.742365783
Nr4a2	163	1.309023241	1.194594231	-0.11442901	0.5708231	0.742365783
310013L24F	93	1.155907857	1.18106926	0.025161402	0.57093231	0.742365783
Zdhhc15	5	18.67109635	20.88979592	2.218699573	0.57090299	0.742365783
Gm11974	5	2.75635767	2.669515934	-0.086841736	0.57101558	0.742365783
Snx7	18	1.015894604	0.881897616	-0.133996989	0.57125425	0.742610288
Kifc3	33	6.706021749	5.526050684	-1.179971066	0.57137172	0.742697187
Endov	33	1.109343677	1.133296658	0.023952981	0.57166896	0.743017748
Gm9962	22	55.97363541	55.84881346	-0.12482195	0.57187759	0.74315726
Ing5	38	0.932013442	1.076144849	0.144131408	0.57187434	0.74315726
Lyrm5	19	1.064403318	1.131444611	0.067041293	0.57222908	0.743548177
Vasp	60	1.815719372	1.83411238	0.018393009	0.57245388	0.743774415
Tmem41b	31	1.100268136	1.201223002	0.100954866	0.57302872	0.744455368
Nt5c3b	11	1.671333313	1.225047656	-0.446285657	0.57337132	0.744702664
Abhd17a	32	1.837147262	1.892623658	0.055476396	0.57332132	0.744702664
Rbm18	6	1.126063264	0.783338906	-0.342724358	0.57334818	0.744702664
Rab14	95	1.203303452	1.517852386	0.314548934	0.57351213	0.744753712
230028O05F	19	4.86600352	4.891445248	0.025441729	0.57349354	0.744753712
Atp6v1b2	16	1.174885056	0.469104818	-0.705780238	0.57383085	0.745101669
Gm7334	45	0.823504662	0.676862887	-0.146641774	0.57399395	0.745181564
Slc18a3	50	15.89942245	16.25646312	0.357040667	0.57395306	0.745181564
Prpf39	37	0.887325291	0.744731603	-0.142593687	0.57413795	0.745302568
230217O12F	14	2.681168726	2.382759415	-0.298409311	0.57419309	0.745308215
Nebl	26	1.600273158	1.877974578	0.277701421	0.57427259	0.745345482
Gm9958	46	0.85516884	0.966724631	0.111555791	0.5743292	0.745353025
Mfap1b	2	6.701030928	4.901960784	-1.799070144	0.5744636	0.745461524
Gm19345	13	30.52034679	31.04688173	0.526534943	0.57484089	0.745885152
Zfp189	12	1.574507514	1.68830484	0.113797326	0.5752614	0.746364787
Gga3	63	1.174343632	1.174178665	-0.000164967	0.57583095	0.747037698
530043F03F	8	0.811382114	0.392675208	-0.418706905	0.57613237	0.747141547
Dnase2a	35	4.589500817	4.883634223	0.294133405	0.5762165	0.747141547
Tmem33	52	1.312606327	1.307057548	-0.005548779	0.5760233	0.747141547
110021A11F	15	1.099310425	1.025128642	-0.074181783	0.57604423	0.747141547

Pdzd3	1	32.8358209	37.5	4.664179104	0.57611158	0.747141547
Cdca8	11	1.542485324	0.907952509	-0.634532815	0.57618238	0.747141547
Ap1g2	17	17.00143319	16.4710845	-0.530348684	0.57634068	0.747173144
'00003D09F	5	1.130386862	0.715486867	-0.414899995	0.5763427	0.747173144
Ccdc38	8	1.63751371	1.349977995	-0.287535715	0.57643517	0.747227005
Slc35g1	68	1.215100452	1.123678729	-0.091421722	0.57672797	0.747540524
H2-Ke6	49	1.385918804	1.271103973	-0.114814831	0.57698695	0.747810152
Inafm2	103	1.301910999	1.308488969	0.00657797	0.57713298	0.747933349
Scand1	23	1.618540649	1.297880464	-0.320660184	0.57724659	0.748014526
Gm9855	20	1.267070218	0.848821188	-0.41824903	0.57736649	0.748103827
Nek8	24	1.273596753	1.550615554	0.277018801	0.57746994	0.748105752
Pacs2	143	1.430220311	1.330600951	-0.09961936	0.57742595	0.748105752
Shpk	23	1.282276882	1.32512921	0.042852328	0.57783073	0.748507076
Tfg	41	2.200166154	1.973411603	-0.226754551	0.57800127	0.748661901
D1Ertd622e	70	1.121306269	1.052125373	-0.069180896	0.57815535	0.74879537
Ddhd2	88	0.967123125	1.122557975	0.15543485	0.57856339	0.749257715
Tmem38b	32	2.041782228	1.862832015	-0.178950212	0.57871166	0.749383596
Idh1	23	1.538546991	1.807199336	0.268652345	0.57891576	0.749530962
Zfp143	50	1.315235472	1.313664626	-0.001570846	0.57892762	0.749530962
Dnajb12	3	1.344053151	2.298850575	0.954797424	0.57900144	0.749560394
Efcab7	18	0.492458684	0.792662312	0.300203628	0.57913259	0.749664045
310032O08F	16	1.646788388	1.166197935	-0.480590453	0.57957296	0.75016791
Rcan1	72	1.831624296	1.359853519	-0.471770777	0.57969236	0.750256275
Atp13a3	121	1.28219972	1.288304561	0.006104841	0.57976601	0.750285422
Ahdc1	16	1.02494716	1.062591087	0.037643926	0.579894	0.750384884
Rgs7bp	20	27.24860411	27.5181516	0.269547486	0.57999913	0.750454748
Mlkl	17	1.95236961	1.67385511	-0.278514501	0.58015383	0.750555078
Brd7	82	0.902824096	0.806534456	-0.09628964	0.58017897	0.750555078
Rps16	5	0.750819881	1.364145658	0.613325778	0.58038189	0.750619062
310061I04R	68	1.280210591	1.132191425	-0.148019166	0.58034758	0.750619062
AF357399	5	0.750819881	1.364145658	0.613325778	0.58038189	0.750619062
Banp	97	1.1893853	1.116028826	-0.073356474	0.58059064	0.7507567
Map3k4	39	1.236942805	1.220196045	-0.01674676	0.58054334	0.7507567
H2afv	64	1.232384023	1.098824268	-0.133559755	0.5810211	0.751247127

Rilpl2	14	1.174741809	0.929089445	-0.245652364	0.58116651	0.75136893
10-Sep	40	1.612254345	1.465263471	-0.146990874	0.58151834	0.751707535
Zfp536	49	46.11849299	46.333187	0.214694007	0.58153087	0.751707535
Naa16	57	0.908787439	0.953251108	0.04446367	0.58179173	0.751978493
045n01rik-r	1	4.377104377	3.542234332	-0.834870045	0.58232784	0.752284487
310045N01F	1	4.377104377	3.542234332	-0.834870045	0.58232784	0.752284487
Rock1	81	1.204773823	1.369051936	0.164278114	0.58238734	0.752284487
Mettl17	26	2.138314362	2.041584968	-0.096729394	0.58221637	0.752284487
Hoxa6	36	41.11652215	42.60637853	1.489856379	0.58211718	0.752284487
Wdr37	13	1.422931762	1.625364189	0.202432427	0.58224734	0.752284487
Tarbp2	23	1.330932344	1.347805802	0.016873458	0.58234634	0.752284487
Zmat3	61	1.596432488	1.554027969	-0.042404519	0.5824716	0.752288688
Yipf1	12	1.511982629	1.322610814	-0.189371815	0.58254439	0.752288688
Atf4	12	3.170702461	2.711404155	-0.459298306	0.58250243	0.752288688
Tmem165	59	1.8290947	1.714561958	-0.114532742	0.58263707	0.752342165
Drosha	42	1.449066201	1.515967337	0.066901136	0.58302844	0.752767048
Madd	71	1.173303989	1.179365979	0.00606199	0.58306871	0.752767048
Bloc1s2	21	1.453983569	1.476890496	0.022906927	0.5835964	0.753382027
Nhs	116	37.93043974	38.01811095	0.087671207	0.58385743	0.753652704
Ddit4	67	1.275349335	1.277446395	0.00209706	0.5843149	0.754176873
Prkar2a	88	0.998811173	0.840043491	-0.158767682	0.58469827	0.754567944
Lmtk2	57	1.267203771	1.520919743	0.253715972	0.58477216	0.754567944
Mdp1	12	1.404123429	1.146205813	-0.257917615	0.58473148	0.754567944
Trp53inp2	48	1.612802109	1.401513605	-0.211288505	0.58494221	0.754675263
Slc46a3	28	1.049540416	1.339762758	0.290222342	0.58495819	0.754675263
Slc35b4	17	1.180144299	1.414614018	0.234469718	0.58510881	0.75473687
Nfxl1	38	1.172097345	1.014592487	-0.157504858	0.58510215	0.75473687
Fbxo45	71	1.072438232	0.981950671	-0.09048756	0.5855321	0.755152175
Tmem106b	48	1.503755736	1.58919871	0.085442974	0.5855337	0.755152175
Ahcyl2	53	1.537803166	1.65290201	0.115098845	0.58558586	0.755153076
Eme1	8	2.392380128	2.043421576	-0.348958552	0.58577027	0.755258139
930426D05F	2	27.40406666	26.01265388	-1.391412781	0.58576688	0.755258139
Cdc14a	64	1.164912734	1.086376913	-0.078535821	0.58601135	0.755502602
310053B23F	1	52.47524752	48.90510949	-3.570138036	0.58607378	0.755516706

Eif5a	60	1.837847788	1.668156974	-0.169690813	0.58615903	0.755560229
Mapk7	63	1.285704927	1.28955932	0.003854392	0.58656558	0.756007998
Eif4a3	34	0.453252479	0.759155655	0.305903177	0.58660945	0.756007998
Sdf2l1	15	2.594005946	2.098698605	-0.495307342	0.58670964	0.756070713
Arl1	46	1.466304151	1.461348302	-0.004955849	0.58692333	0.756279681
Etohi1	12	2.089912972	2.023200994	-0.066711978	0.58701994	0.756286152
Rpl41	14	1.024179871	1.145430972	0.121251101	0.58703143	0.756286152
Sfrp4	10	13.76317648	15.36301959	1.599843112	0.58729547	0.756559895
Rps2	6	1.934744948	1.685353348	-0.2493916	0.5877531	0.757013788
Snora64	6	1.934744948	1.685353348	-0.2493916	0.5877531	0.757013788
Dusp9	18	13.33002238	14.17010485	0.840082463	0.58780258	0.757013788
Naf1	39	1.1660605	1.18880285	0.02274235	0.58802856	0.757238354
Tor2a	45	2.276790204	1.943771326	-0.333018878	0.58811573	0.757284156
Col4a6	2	49.24379915	46.81236203	-2.431437122	0.58837185	0.757547468
Chmp1a	49	1.339537865	1.382684151	0.043146286	0.58876441	0.75778695
Uqcrfs1	38	1.200900535	1.219115579	0.018215045	0.58868805	0.75778695
Parp3	11	0.96725526	0.762786887	-0.204468373	0.58874171	0.75778695
Pitpnc1	23	0.974474638	1.318805459	0.344330821	0.58863585	0.75778695
Acap3	67	0.957635258	0.947570245	-0.010065013	0.58897905	0.757982731
Asxl2	57	1.068126231	1.040390165	-0.027736066	0.58901984	0.757982731
Qtrt1	30	1.810646829	1.550384321	-0.260262508	0.58917824	0.758120084
Tcea2	31	1.121433397	1.165095274	0.043661877	0.58930944	0.758222417
Slc12a3	2	89.22773445	90.52083333	1.293098887	0.58948235	0.758378399
Bag1	64	1.151434333	1.240239607	0.088805273	0.58965048	0.758528198
Ric8b	24	1.958218862	1.057846024	-0.900372838	0.58991207	0.758798183
Rpn2	74	1.387410158	1.223634206	-0.163775952	0.59022917	0.759139525
Bmf	39	1.709102113	1.796268177	0.087166064	0.5903928	0.759283431
Knop1	68	1.338157165	1.192106451	-0.146050714	0.59048756	0.759338743
Foxm1	32	1.249110642	1.304679557	0.055568916	0.59054846	0.759350521
Vkorc1l1	41	1.148816887	1.203862859	0.055045972	0.59072853	0.759515508
Commd2	6	2.128274997	1.843965832	-0.284309164	0.59080326	0.759545036
Akip1	42	1.640409007	1.313258179	-0.327150828	0.59112093	0.75986738
Gtpbp3	16	1.614386907	1.829363342	0.214976436	0.59115756	0.75986738
Cxcl10	3	0.555555556	0.99009901	0.434543454	0.59131706	0.76000583

Tnfrsf22	18	2.340006856	2.334296915	-0.005709941	0.59139921	0.760044836
Hnrnpul1	47	1.13300704	0.944617127	-0.188389913	0.5917614	0.760443714
Sp3	205	1.151102816	1.102413732	-0.048689084	0.59184669	0.760486717
Armc10	25	1.684980954	1.409108481	-0.275872473	0.59198465	0.760597391
Tbcel	25	1.39436029	1.289551823	-0.104808466	0.59220699	0.760749836
Gmnn	48	1.092793326	1.16436891	0.071575584	0.59220289	0.760749836
Bak1	13	2.550292377	2.556283311	0.005990934	0.59233848	0.760852142
Prkag1	21	1.615770447	1.497837755	-0.117932692	0.59307461	0.761731008
Slc38a6	17	1.220370804	1.3239981	0.103627296	0.59328899	0.761939672
Sar1b	16	1.977397131	2.520825134	0.543428004	0.59351583	0.762164284
U2af1	81	1.093217535	0.997445145	-0.095772389	0.59368324	0.762312548
Pcyox1l	43	5.369323878	5.780060251	0.410736372	0.59396148	0.762603094
Emc8	65	0.890866342	1.018903516	0.128037174	0.59405628	0.762611958
Cox4i1	48	1.004312444	0.91221083	-0.092101614	0.59407232	0.762611958
Sdhaf3	22	1.142677382	0.930663088	-0.212014294	0.59418566	0.762690726
230354K17R	27	1.649235582	1.485443531	-0.16379205	0.59435277	0.762838497
Fkbp2	35	1.405373266	1.421538586	0.01616532	0.59464753	0.763016596
Pdzd11	5	17.66046938	16.3990128	-1.261456581	0.59457834	0.763016596
Tm7sf3	39	1.685580418	1.547062036	-0.138518382	0.59462079	0.763016596
Vrk3	5	3.578336557	3.352586318	-0.22575024	0.59470019	0.76301745
Parp16	34	1.743283511	1.594061513	-0.149221998	0.59484453	0.763135921
Chrac1	32	0.627606769	0.641413948	0.013807179	0.59495726	0.763213815
Rps10	76	1.366723311	1.232333813	-0.134389498	0.59518459	0.76343869
AI846148	31	2.055476127	1.978284219	-0.077191907	0.59538206	0.763599144
Lin7b	27	7.610695029	10.36170705	2.751012025	0.59556009	0.763599144
933406C10R	6	1.56838335	1.02395817	-0.54442518	0.59545098	0.763599144
Tprkb	15	1.311459101	1.242805589	-0.068653512	0.59549328	0.763599144
Eml4	81	1.240644213	1.317165986	0.076521773	0.59556987	0.763599144
Gtpbp1	49	1.088009898	1.127637936	0.039628038	0.59569966	0.763698827
Metrn	18	1.018835616	1.270903709	0.252068093	0.59577408	0.76372751
Primpol	14	2.423627767	1.725476094	-0.698151673	0.59596944	0.763911213
Commd8	15	0.904286618	1.705306733	0.801020115	0.59622684	0.764174393
Cherp	66	0.8450072	1.066566143	0.221558942	0.59634296	0.764256467
Mir5100	18	0.586122222	0.841754063	0.255631841	0.59644237	0.764317117

Suds3	127	1.26755353	1.234311186	-0.033242344	0.59662531	0.76448205
Pms1	4	0	0.161290323	0.161290323	0.59667527	0.76448205
Zbtb17	73	1.313089627	1.15836036	-0.154729267	0.59700186	0.764714387
Gm7008	14	1.137724883	1.237734325	0.100009442	0.59695581	0.764714387
Tmem14c	39	1.301843506	1.359831176	0.05798767	0.59701295	0.764714387
Sec11c	13	1.042922519	1.340598472	0.297675953	0.59718192	0.764864061
Tnfaip3	42	1.305501247	1.094140288	-0.211360959	0.59730473	0.764954584
Frrs1	14	1.884726509	1.767216516	-0.117509994	0.59772864	0.765430677
Wdr92	3	0.806451613	0.303030303	-0.50342131	0.59784826	0.765517049
Atpif1	8	1.763410662	1.458246315	-0.305164347	0.59790243	0.765519611
Zfp12	13	0.518436336	1.021511478	0.503075142	0.59797876	0.765550541
Coil	36	1.574765553	1.646818388	0.072052835	0.59810243	0.765584298
Wapal	101	1.363982061	1.301068292	-0.062913769	0.59810947	0.765584298
Smndc1	98	1.522186029	1.505387697	-0.016798332	0.59827144	0.76565804
11-Mar	114	58.20255154	58.18295933	-0.019592204	0.59825296	0.76565804
Ctxn1	44	4.047197303	3.905733088	-0.141464215	0.59836429	0.765710089
Plekhm2	90	1.646344426	1.258980476	-0.38736395	0.5987712	0.766163984
310036O22R	44	1.593616357	1.365982786	-0.22763357	0.59913133	0.766557951
Lztfl1	26	1.074782025	1.319535277	0.244753251	0.59938688	0.766684364
Fgd6	55	1.236106419	1.056692075	-0.179414344	0.59937746	0.766684364
700062C07R	24	5.430762724	5.560956531	0.130193807	0.59934641	0.766684364
Mtf1	40	1.746617896	1.56755747	-0.179060426	0.59949062	0.766750224
Gpn3	55	1.113778844	1.093202036	-0.020576808	0.59966722	0.766775602
Nampt	103	1.275255753	1.27531554	5.98E-05	0.59960486	0.766775602
Zfp408	8	3.452350609	3.039181025	-0.413169584	0.5996183	0.766775602
Mapre2	55	1.776400784	1.556532286	-0.219868498	0.59980448	0.766884287
Zfp746	123	1.181413225	1.077735534	-0.103677691	0.59987828	0.766906663
Myl9	24	21.7270066	22.8459014	1.118894804	0.59992651	0.766906663
Pfdn2	13	1.262486849	0.759745692	-0.502741156	0.60007301	0.766960313
Ncaph	31	1.196982463	1.030167072	-0.166815391	0.60005125	0.766960313
Prps1l3	10	0.107526882	0.35869177	0.251164888	0.60025764	0.767062672
Alg6	11	0.336729206	0.82092704	0.484197835	0.60020729	0.767062672
Cops2	28	1.645914179	1.767578892	0.121664713	0.60050087	0.767306671
Ccdc172	5	92.36817204	92.8168499	0.448677859	0.60058091	0.767342126

Cep290	34	1.396394365	1.836833243	0.440438878	0.6006767	0.767397695
Lrrc3b	32	56.15425618	55.81869097	-0.335565216	0.60076848	0.767448135
Efna1	26	1.651269746	1.831367769	0.180098023	0.6010244	0.767708226
Strap	9	1.063829787	0.444444444	-0.619385343	0.60133644	0.7679731
Hspe1	57	1.140903768	1.09935163	-0.041552138	0.60131932	0.7679731
Celf5	39	3.584816294	3.765605741	0.180789447	0.60152921	0.768152441
Nipa2	55	1.101614284	1.045744518	-0.055869767	0.60183477	0.768446213
Pigl	54	1.42949682	1.357886233	-0.071610587	0.601864	0.768446213
Setdb2	9	1.080246914	0.957854406	-0.122392507	0.60212805	0.768657894
Gm4013	11	1.573170119	1.927706526	0.354536407	0.60213456	0.768657894
Rbbp6	17	1.737833645	1.617743917	-0.120089728	0.60229031	0.768789839
Psmc2	20	0.767200047	0.827425108	0.060225061	0.60262511	0.769150284
Slc25a27	17	1.72310399	1.335463997	-0.387639993	0.60277646	0.769276549
Ino80c	28	1.398542518	1.370223133	-0.028319386	0.60305368	0.769429595
Tomm6	25	1.242962852	1.61403226	0.371069408	0.60300795	0.769429595
310022K09R	30	2.061754361	2.021191306	-0.040563055	0.60304503	0.769429595
Entpd7	21	1.273846269	1.374691304	0.100845035	0.60336085	0.769728533
Gnb5	61	1.363948021	1.646039283	0.282091262	0.60339289	0.769728533
Ppp4r1l-ps	7	0.707213579	0.868230048	0.16101647	0.60368047	0.769816392
Ano6	79	1.361253064	1.10156417	-0.259688894	0.60380309	0.769816392
Fam76b	33	1.365147292	1.54101993	0.175872639	0.60367037	0.769816392
Alg5	36	0.997254665	0.957252448	-0.040002217	0.60379366	0.769816392
Acbd4	9	1.789623759	1.444918083	-0.344705676	0.60374621	0.769816392
Fam45a	13	1.169218456	1.385772603	0.216554147	0.603829	0.769816392
Rbl1	61	1.260522755	1.099533294	-0.160989462	0.60368303	0.769816392
Map3k2	69	1.151221474	1.064768655	-0.086452819	0.60400342	0.769884153
Eml5	61	1.127894763	0.931160306	-0.196734457	0.60397123	0.769884153
Trappc12	19	1.346520908	1.227297815	-0.119223093	0.60408186	0.769884153
Mtmr9	51	1.530898325	1.332979162	-0.197919163	0.60409201	0.769884153
Pias3	61	1.512446587	1.580368195	0.067921608	0.60416615	0.76991177
Purb	93	1.039897381	0.961055191	-0.07884219	0.60425187	0.76995414
Ttc16	11	0.728491066	0.430202827	-0.298288239	0.60459938	0.770263184
Ptrh1	11	0.728491066	0.430202827	-0.298288239	0.60459938	0.770263184
Tdrd7	61	1.555782283	1.289593279	-0.266189003	0.604671	0.770287548

Mypop	7	1.639851708	1.061512253	-0.578339454	0.60474111	0.770309983
Hsp90b1	41	1.659993512	1.972813787	0.312820275	0.60498345	0.770503659
Gm10785	29	1.34222708	1.191925016	-0.150302064	0.60505068	0.770503659
Hyls1	46	1.380502092	1.131428129	-0.249073964	0.60502477	0.770503659
i00002K03F	36	58.46533291	58.27810695	-0.187225962	0.6056536	0.771204524
Gnpnat1	68	1.943120058	1.788713114	-0.154406944	0.60574704	0.771246484
Lrfn4	31	1.421276266	1.318479147	-0.102797118	0.60579167	0.771246484
Ccz1	34	1.294695333	1.159794123	-0.13490121	0.60587982	0.771291793
Nucks1	28	1.222724161	1.054326198	-0.168397963	0.60614293	0.771510258
Mpz	8	1.415846494	0.941504509	-0.474341985	0.60615659	0.771510258
Chfr	12	1.928212505	1.905579911	-0.022632595	0.60625517	0.771568808
Slc25a15	96	1.140427269	1.003444927	-0.136982342	0.60645346	0.771754235
Per3	49	1.575014643	1.694035597	0.119020954	0.60666033	0.771816684
Ttc9c	4	2.09715563	2.470240444	0.373084814	0.6066446	0.771816684
Tex261	44	1.449721293	1.323990247	-0.125731046	0.6065831	0.771816684
Stxbp2	15	1.242612966	1.278676964	0.036063999	0.60685334	0.771995315
Rnd1	6	1.873026085	1.468470596	-0.404555489	0.60696116	0.771998622
Imp3	24	0.584422466	1.067876782	0.483454316	0.60693939	0.771998622
Fam212a	39	1.782789462	1.955621652	0.17283219	0.60706244	0.772060518
Tet2	81	1.095695548	1.097932242	0.002236694	0.60718274	0.772079685
Cyp51	40	0.725925833	0.843750776	0.117824943	0.60716751	0.772079685
Thoc3	40	1.011539557	1.112426643	0.100887087	0.60726194	0.772113489
Rab6a	30	1.231090117	1.080100877	-0.15098924	0.60740725	0.772164425
Mfsd11	43	1.825306899	1.443012325	-0.382294574	0.60735859	0.772164425
Prrt1	2	82.23426679	79.91596639	-2.318300401	0.60753074	0.772244618
Srp19	67	1.078060019	1.207856425	0.129796407	0.60760827	0.772244618
Sdhb	34	1.521238075	1.40212732	-0.119110755	0.60762821	0.772244618
Clptm1l	39	1.186123391	1.077025336	-0.109098056	0.60792103	0.77254986
Ebag9	86	1.350948086	1.325728237	-0.025219849	0.60798555	0.772564938
Arf6	125	1.256569006	1.246141171	-0.010427834	0.60806849	0.772603428
Ppp1r12c	43	0.851867192	0.707142609	-0.144724582	0.60855827	0.773158793
Thrap3	62	1.608828156	1.618730045	0.009901888	0.60874308	0.773326629
Snrpb	41	1.349483287	1.367050074	0.017566786	0.60882652	0.773365688
Neu1	12	0.53369336	1.310325477	0.776632117	0.60889754	0.773388947

Rab40c	11	1.427941221	1.428695826	0.000754605	0.60909849	0.773571257
Tmem252	3	55.60373382	53.6365833	-1.967150512	0.60919923	0.773571257
Zeb1	31	1.201757997	1.297860289	0.096102292	0.6091969	0.773571257
Yrdc	86	1.067466083	0.987922487	-0.079543596	0.60925312	0.773572756
Slc25a11	24	1.380775578	1.629480165	0.248704586	0.60935065	0.773627862
Pdxdc1	41	1.161815038	0.946464612	-0.215350426	0.60940197	0.773627862
310015D24F	2	85.17316017	86.5728368	1.399676629	0.60960812	0.773822624
Cxcl14	14	35.07504514	36.15765975	1.082614605	0.60966775	0.773831376
Arnt	3	5.291005291	4.920101781	-0.37090351	0.60979836	0.773930216
Rfwd2	47	0.764078236	0.859681605	0.095603369	0.60999904	0.774117952
Rmrp	12	1.054740872	1.128015658	0.073274786	0.61013457	0.774222987
Jkamp	5	1.268055556	1.444772084	0.176716528	0.61021254	0.774254975
Wdfy1	59	1.011990742	1.141764949	0.129774207	0.61035598	0.774370019
Sar1a	35	2.011714273	2.073291504	0.061577231	0.61067001	0.774701452
Olfr658	2	75	73.19587629	-1.804123711	0.61095017	0.774922882
Mfap1a	2	1.968503937	1.111111111	-0.857392826	0.61092237	0.774922882
Chmp3	42	1.354126721	1.18711571	-0.16701101	0.61102124	0.774946045
B3galt6	29	0.93911863	0.629966253	-0.309152377	0.61117134	0.775069422
Mterf4	27	3.773015962	5.011452984	1.238437021	0.61125108	0.775103561
Eif4g2	61	1.36326742	1.373009334	0.009741914	0.61143103	0.775197765
Mta3	28	1.644215899	1.594111966	-0.050103932	0.61141341	0.775197765
Nxn	77	1.282553032	1.395928655	0.113375623	0.61152411	0.775248791
Gsap	6	1.17743069	1.180776342	0.003345651	0.61169399	0.775397167
Fam98a	42	1.776529278	1.50189508	-0.274634199	0.61201254	0.775500946
Drg1	43	1.33565307	1.326349933	-0.009303137	0.61209295	0.775500946
Zmym5	76	1.288992696	1.269255767	-0.019736929	0.61206949	0.775500946
St3gal1	35	1.693357631	1.525930871	-0.16742676	0.61205082	0.775500946
Fgfrl1	119	3.450701932	3.298261681	-0.152440251	0.61202142	0.775500946
Snn	55	2.178939605	2.006481365	-0.17245824	0.61192461	0.775500946
Kifc2	6	3.484831933	3.276187271	-0.208644661	0.61216386	0.775523824
Alox8	3	5.832193342	6.116207951	0.284014609	0.61246975	0.775844364
Mapkapk2	57	1.047304222	1.150137888	0.102833667	0.61275629	0.77584442
Asb1	33	1.500896938	1.536632816	0.035735878	0.61265384	0.77584442
Ankrd54	40	1.172471937	0.997137728	-0.175334209	0.61278703	0.77584442

Sav1	123	1.019963879	0.894386063	-0.125577815	0.61255382	0.77584442
Tmem186	35	2.029324836	1.600046306	-0.42927853	0.61260424	0.77584442
Reep5	85	1.201791837	1.279654252	0.077862414	0.61278482	0.77584442
Golga7	22	1.199138388	1.065032932	-0.134105456	0.6133915	0.776542742
Eno1b	16	2.438971724	2.632479668	0.193507944	0.61344649	0.776545359
Ghdc	3	3.351369077	2.3115988	-1.039770277	0.6137195	0.776556221
330035P11F	5	1.24210348	1.733281778	0.491178297	0.61371967	0.776556221
Nrarp	51	1.247428222	1.22743968	-0.019988542	0.61364484	0.776556221
Sord	4	2.75	2.5	-0.25	0.61357764	0.776556221
Thap7	53	2.116448623	2.038625553	-0.07782307	0.61351548	0.776556221
Frat2	68	0.98730029	1.210877779	0.22357749	0.61396725	0.776601618
Rrm2b	43	1.50953833	1.249911245	-0.259627085	0.61385875	0.776601618
Tsku	79	1.520788047	1.587940483	0.067152436	0.61393156	0.776601618
Tstd2	9	1.342080172	1.810068727	0.467988554	0.61386258	0.776601618
330403N18F	2	63.13401442	64.70649659	1.572482171	0.61402296	0.776605143
Camkmt	36	1.182284517	1.658785487	0.47650097	0.61419354	0.776651138
Nmt2	43	0.968105103	0.933459307	-0.034645796	0.6142181	0.776651138
Sphk2	53	1.358761895	1.12241304	-0.236348855	0.61412951	0.776651138
930555B11F	10	1.299831927	0.795080913	-0.504751014	0.61431629	0.776708356
Nudt19	39	1.107484625	0.84085897	-0.266625655	0.61454339	0.776759122
Slc25a28	60	1.560155068	1.272769696	-0.287385371	0.61456344	0.776759122
Grb14	40	4.667310059	4.44216152	-0.225148539	0.61447126	0.776759122
Bckdhb	37	1.329122609	1.42232192	0.09319931	0.61456818	0.776759122
Ubr4	35	1.227087629	1.166288214	-0.060799414	0.61476306	0.776938521
Srsf11	65	1.555611035	1.363971416	-0.191639618	0.61496248	0.77712362
Gfm1	3	2.5	2.315612316	-0.184387684	0.615162	0.777308813
Max	89	1.512525277	1.584389539	0.071864263	0.61540698	0.777542678
Clk3	36	1.291555627	0.948089384	-0.343466243	0.61545306	0.777542678
Ssx2ip	80	2.140907369	1.651285252	-0.489622117	0.61558795	0.777646138
Baiap2l1	13	1.712610992	1.768050556	0.055439563	0.61567319	0.777686869
Gpr180	77	1.196048321	1.115906926	-0.080141395	0.61577469	0.777748138
Gxylt1	53	1.217372139	1.379636532	0.162264394	0.61584379	0.777768464
Tnk1	8	41.2018318	40.97813479	-0.223697008	0.61631687	0.778231971
Tex40	29	66.75229767	66.63258047	-0.119717197	0.61629507	0.778231971

Acadl	30	1.591118687	1.481852304	-0.109266384	0.61641671	0.778291071
Tmem170	19	1.09719007	1.007700106	-0.089489964	0.61707645	0.779057021
Fbxl21	19	1.572856968	1.834962276	0.262105309	0.61719841	0.779143969
Pex11g	2	5.924006908	6.506489215	0.582482306	0.61761682	0.77947099
Brca1	9	1.666100644	1.290262874	-0.37583777	0.6175672	0.77947099
Ldlrap1	96	3.788645921	3.184898181	-0.603747741	0.6175493	0.77947099
Ccdc85b	32	1.177648056	1.258118771	0.080470716	0.61772218	0.779536912
Hmgb2	88	1.271490826	1.20338748	-0.068103346	0.61777872	0.779541232
Gabbr1	16	2.012129747	2.670782181	0.658652433	0.61833921	0.780181386
Tfeb	97	4.273686694	4.046144632	-0.227542062	0.61857915	0.780349932
Hormad2	21	80.06696379	79.28014715	-0.786816647	0.61855902	0.780349932
Atrn	53	0.985220284	0.775067253	-0.210153031	0.61873179	0.780475395
Lrrc20	10	1.409837338	1.52632495	0.116487612	0.61893969	0.780670532
Daxx	38	1.400257758	1.417080141	0.016822383	0.61907743	0.780730918
Tceb1	22	1.955438723	1.358412405	-0.597026318	0.61909397	0.780730918
Tmem234	17	0.925989086	1.11850997	0.192520884	0.61925038	0.780778182
Ankrd17	101	0.973354663	0.879495844	-0.093858819	0.61929108	0.780778182
Asb3	11	0.737610622	1.136590239	0.398979617	0.61918915	0.780778182
Tsen54	70	1.023737635	0.935279881	-0.088457754	0.6194388	0.780854863
Coq10b	82	1.338678442	1.210699696	-0.127978746	0.61945832	0.780854863
Rragc	98	1.380161542	1.30381977	-0.076341771	0.61952719	0.78087459
Tufm	17	1.435453502	1.160465214	-0.274988288	0.6196146	0.780914325
)30577N17F	110	1.329613352	1.230258213	-0.099355139	0.61966515	0.780914325
Pop7	70	1.523616408	1.283846433	-0.239769976	0.61972117	0.780917859
Htatsf1	30	18.99791903	18.86748876	-0.130430267	0.61983257	0.780991166
Mir6947	6	93.5999972	93.44969463	-0.150302569	0.61998517	0.781116368
Psmd3	52	1.218188638	1.188168568	-0.03002007	0.62016144	0.78127138
Tbce	23	1.783438047	1.839667528	0.056229481	0.62036601	0.781320976
Slc15a3	5	53.87337258	54.25798446	0.384611879	0.62037057	0.781320976
Dnajb6	18	0.887221186	0.699048166	-0.18817302	0.62029554	0.781320976
Psmd13	16	0.91191278	0.511037467	-0.400875314	0.62041379	0.781320976
Ctnnbip1	42	1.281612425	1.359636804	0.078024378	0.62064221	0.781541564
Clptm1	28	1.703455915	1.59249626	-0.110959655	0.62096239	0.781877645
Wdr83os	3	2	0.925925926	-1.074074074	0.62154735	0.782412789

Nphs1os	13	33.93557957	42.68796711	8.752387538	0.62151857	0.782412789
Atf5	36	1.486165585	1.356573159	-0.129592426	0.62146037	0.782412789
700020I14R	20	1.587891178	1.262226188	-0.325664989	0.62225474	0.783096826
Atl1	92	1.35020039	1.322269552	-0.027930838	0.62218462	0.783096826
Prrc1	63	1.163168369	1.040200254	-0.122968114	0.62240877	0.783096826
310002D19F	12	1.292229964	1.33234679	0.040116825	0.62241095	0.783096826
Ganab	28	1.381528363	1.19726792	-0.184260443	0.62239686	0.783096826
Rel	117	1.365164968	1.280765262	-0.084399706	0.62229647	0.783096826
Afg3l2	81	1.21272799	1.253022582	0.040294592	0.62256665	0.78309898
Nr4a3	86	5.767362139	5.856751042	0.089388903	0.62262613	0.78309898
D10Wsu102	56	1.75688423	1.559458244	-0.197425986	0.62262271	0.78309898
Rpl7a	10	1.375438596	1.189447591	-0.185991006	0.62261649	0.78309898
933433G15F	29	0.901018298	0.892487949	-0.008530349	0.62268937	0.783111403
Arrb2	34	1.258329485	1.521890353	0.263560868	0.62284482	0.783239777
Nfkbib	19	1.63015364	1.980603096	0.350449457	0.62290844	0.783247158
Gtf2h2	22	1.398706942	1.358603022	-0.040103919	0.62295745	0.783247158
Ppp2r2d	90	1.018401226	0.886937409	-0.131463817	0.62331204	0.783491589
310022B05F	83	1.897909946	1.361072227	-0.536837719	0.62323567	0.783491589
Akt1	108	1.069228774	0.934458885	-0.134769889	0.62328534	0.783491589
Pcmt1	17	0.861736397	1.067046697	0.2053103	0.62350838	0.783671265
Stard7	47	1.294919547	1.468744323	0.173824776	0.62360688	0.78372793
Gpr160	29	3.261190671	3.307333503	0.046142832	0.62390006	0.783962111
Clic1	3	3.947041687	3.231166248	-0.715875439	0.62389329	0.783962111
Erbb2	55	1.570800274	1.545418075	-0.025382199	0.62425907	0.784346052
Herpud1	13	0.93150649	0.877953963	-0.053552527	0.62444908	0.784517615
Mlx	12	1.805104884	1.281684946	-0.523419938	0.62450605	0.784522031
Frg1	40	1.486322428	1.336077359	-0.150245069	0.6246908	0.784534387
Prmt1	31	1.075572068	0.846621729	-0.228950338	0.62489014	0.784534387
Upf2	55	1.646545084	1.811140855	0.164595772	0.62487594	0.784534387
Ankmy2	15	2.02640977	1.534086374	-0.492323397	0.62476329	0.784534387
Qtrtd1	17	1.362715802	1.961054335	0.598338533	0.62457232	0.784534387
Pdia4	43	1.333783551	1.690678026	0.356894475	0.62485759	0.784534387
Ap5b1	92	1.114986392	1.147806128	0.032819736	0.62467705	0.784534387
Prdm4	87	1.415911867	1.296238799	-0.119673068	0.62494551	0.784536782

Pigx	14	1.854893859	2.183282187	0.328388328	0.62525426	0.784857231
Pgp	112	1.21325721	1.192676659	-0.020580551	0.6253354	0.784891933
Rfx3	55	0.963232263	0.773070488	-0.190161775	0.62557194	0.785113414
Ywhah	51	1.286090085	1.111884711	-0.174205374	0.62561886	0.785113414
Unc13d	6	3.250538472	3.458603082	0.20806461	0.62579754	0.785203343
Smyd2	92	1.132499093	1.066337022	-0.06616207	0.62578544	0.785203343
Hexdc	5	0.567375887	0.683160415	0.115784528	0.62599016	0.785377879
Taf5l	113	0.93966729	0.893353231	-0.046314059	0.62622966	0.78554038
Pxn	55	1.237028066	0.981967646	-0.25506042	0.62629108	0.78554038
Brpf3	108	1.662555943	1.491880798	-0.170675145	0.62635633	0.78554038
Slc4a1ap	35	1.514422785	1.399694689	-0.114728096	0.62633788	0.78554038
E4f1	11	1.280152472	1.574470011	0.294317539	0.62638735	0.78554038
Mphosph10	30	1.605338497	1.194924756	-0.410413741	0.62653898	0.785663388
Hnrnph3	54	1.148692024	1.194633205	0.045941181	0.62661477	0.785691286
Hdhd2	20	0.987324079	0.561367053	-0.425957027	0.62677641	0.785826818
Bcl6	65	0.871225233	1.047188486	0.175963254	0.62698482	0.785834576
Atf1	121	0.974296678	0.807798996	-0.166497682	0.62696274	0.785834576
Yif1b	27	1.350850416	1.007079536	-0.343770881	0.62690653	0.785834576
Abcf3	9	1.685460842	1.673907293	-0.011553549	0.62699681	0.785834576
Aplp2	46	1.177283348	1.067063782	-0.110219565	0.62710777	0.785839404
Top3a	9	0.684276007	1.09529083	0.411014822	0.6270903	0.785839404
Prpf38a	17	1.447773129	1.310554905	-0.137218224	0.62752933	0.786233389
Pcgf2	90	1.197688692	1.084305103	-0.113383589	0.62752398	0.786233389
Mex3c	139	1.039069243	1.05640747	0.017338227	0.62787901	0.786604339
Rpl11	33	1.00359095	0.948826114	-0.054764836	0.62798268	0.786667047
Atp13a4	2	38.13559322	35.50724638	-2.628346844	0.62809158	0.786690686
Tex9	18	2.239619391	2.214150463	-0.025468928	0.62810877	0.786690686
Hsd17b10	1	25.58139535	28.24427481	2.66287946	0.62830653	0.786725881
Rtn3	21	0.733482642	0.855505148	0.122022506	0.62840494	0.786725881
510038B21R	8	1.20748337	1.41663695	0.209153579	0.62838091	0.786725881
Ufm1	28	1.158910193	1.433924622	0.275014429	0.62825908	0.786725881
Herc4	62	1.174675658	1.147891533	-0.026784125	0.62839455	0.786725881
Coq7	26	0.840428442	1.14853532	0.308106877	0.6285221	0.786762241
Epc2	82	1.335666114	1.344325409	0.008659295	0.62854121	0.786762241

Eif3e	17	1.061873867	0.981314131	-0.080559735	0.62861092	0.786782385
Med13l	203	1.28217089	1.128874172	-0.153296719	0.6286869	0.786810372
8-Mar	51	1.304327792	1.025743689	-0.278584102	0.62874682	0.786818253
Gsto2	22	0.928790412	0.697896784	-0.230893628	0.62883485	0.786861312
Smim8	5	2.04633239	2.344171464	0.297839074	0.62901333	0.786993721
Crebbp	159	1.222325013	1.185363254	-0.036961759	0.62904793	0.786993721
Zscan12	21	2.198938276	1.611288137	-0.587650138	0.62910157	0.786993723
AA543186	28	1.330782769	1.489856091	0.159073322	0.62919107	0.787030518
Pkd2	79	1.181566709	1.445106467	0.263539758	0.62923825	0.787030518
Rnf7	18	1.624069062	1.507919195	-0.116149867	0.62929204	0.787030718
Sertad1	33	1.559477942	1.358377678	-0.201100264	0.62959606	0.787343842
Atp2a2	131	1.109815755	0.957375157	-0.152440598	0.62989475	0.787650237
Celf6	32	5.457650288	6.011033448	0.553383161	0.63007816	0.787745329
Pop4	22	2.18955613	2.221191185	0.031635055	0.63006977	0.787745329
Coq9	37	1.011698041	1.011135743	-0.000562298	0.63041829	0.788036293
Cox6a1	72	1.404960114	1.296458501	-0.108501613	0.63041593	0.788036293
Gsto1	28	1.665614079	1.50114543	-0.164468649	0.63054626	0.788129111
Vps53	6	1.181886896	0.78125	-0.400636896	0.63072034	0.788178605
630045J12F	81	1.390181624	1.193111045	-0.197070579	0.63074699	0.788178605
Zfp809	15	1.901239795	1.664334508	-0.236905287	0.63064525	0.788178605
Cstf2t	19	1.970725184	1.423761034	-0.546964151	0.63086622	0.78826046
Prpsap2	20	1.208206109	1.063918095	-0.144288014	0.6312738	0.78870258
Mea1	53	1.585710952	1.815145363	0.229434411	0.63140153	0.788743523
330031N03F	78	1.648466714	1.764875852	0.116409138	0.63141408	0.788743523
Ccdc142	56	1.430942673	1.355105652	-0.07583702	0.63179608	0.789153536
Plk3	64	1.280028385	1.280437421	0.000409036	0.63196621	0.789231668
Ndufb9	19	1.989463619	1.783330435	-0.206133184	0.63192854	0.789231668
Plekha3	99	4.008128052	3.63278168	-0.375346372	0.6321187	0.78935493
Fbxl12	39	2.02904122	1.977888105	-0.051153114	0.63225842	0.789462219
Surf6	32	1.942453188	1.905934551	-0.036518637	0.63238108	0.789548201
Tmem68	17	1.722941481	1.410232911	-0.31270857	0.63246924	0.789591084
Pdik1l	134	1.391512251	1.083052373	-0.308459877	0.63266506	0.789768364
Bach1	81	1.002709266	1.126564094	0.123854828	0.63289605	0.78992231
Med21	26	1.722012465	1.502212242	-0.219800223	0.63287306	0.78992231

Epb4.1	151	1.618253415	1.608611423	-0.009641992	0.63314732	0.790107131
Cnot6l	131	1.310830059	1.075369876	-0.235460183	0.63315182	0.790107131
Esrra	68	1.557874566	1.531650367	-0.026224199	0.63332623	0.790257578
Gm10549	8	0.754708005	0.913235117	0.158527112	0.63339127	0.790271527
Bub1	17	1.453744984	1.523864637	0.070119653	0.63349287	0.790331098
Atp5g1	6	2.279616698	1.772645329	-0.50697137	0.63364324	0.790451489
Adcy9	77	1.116372483	1.127075756	0.010703273	0.63389477	0.790698053
Usp7	176	1.254690107	1.278823512	0.024133405	0.63402054	0.790787708
Ric1	135	1.099513535	1.121277388	0.021763853	0.63411301	0.79083582
Gdap1	5	36.39303492	37.65102844	1.257993518	0.63443477	0.79116987
Gemin5	55	1.337496397	1.304790619	-0.032705779	0.6345489	0.791244953
Ubqln1	137	1.322289073	1.139187368	-0.183101705	0.63469192	0.791356036
Vaultrc5	10	1.369374253	1.601638599	0.232264346	0.63495996	0.791586171
Mtbp	29	1.751341473	1.518954938	-0.232386535	0.63501416	0.791586171
Gmfb	12	0.691551801	0.802118418	0.110566617	0.63503833	0.791586171
Slc25a1	93	1.657566256	1.561495418	-0.096070837	0.63514953	0.791657534
Mta2	70	1.099606095	1.063982476	-0.035623619	0.63532163	0.791804791
Cnot1	40	1.019580216	1.147601225	0.128021009	0.63537647	0.791805892
Zfp507	50	0.827800203	1.260781048	0.432980845	0.63551541	0.791888566
Txndc9	38	0.849219956	1.061257521	0.212037566	0.63555075	0.791888566
Wdr61	12	1.097000012	1.502535357	0.405535345	0.63564448	0.79193811
Hps3	34	1.736094593	1.428735745	-0.307358847	0.63578637	0.79198041
Nfkbiz	106	1.505124725	1.439820491	-0.065304234	0.63576873	0.79198041
Tmem41a	16	1.489677037	1.177493253	-0.312183784	0.63586144	0.79200669
Ddx17	19	1.407354904	1.634485366	0.227130462	0.63596657	0.7920704
Smug1	5	0.540540541	1.100917431	0.560376891	0.63605309	0.79211093
Atp5o	31	1.414275885	1.471418344	0.057142459	0.63615265	0.792167687
Frmd6	69	1.09850876	1.139097202	0.040588442	0.63650276	0.792536397
Ccnl2	48	1.035097544	0.859742442	-0.175355102	0.63681166	0.792541383
Shq1	9	0.354284474	0.624315736	0.270031262	0.63676791	0.792541383
Ogfod3	22	0.381937051	0.424649603	0.042712553	0.63667397	0.792541383
Smarcad1	58	0.993995576	0.703478729	-0.290516847	0.63682737	0.792541383
Med28	31	1.027777002	1.049932812	0.02215581	0.63688483	0.792541383
Pls1	18	2.613919947	2.166779811	-0.447140135	0.63672234	0.792541383

Slc39a13	40	1.681715644	1.288030778	-0.393684866	0.63685029	0.792541383
Fnbp4	46	0.77722052	0.866400617	0.089180098	0.63717144	0.792830809
Pycr2	24	1.715103902	1.908256704	0.193152802	0.63731921	0.792947444
Dennd4c	31	1.309017142	1.145132079	-0.163885063	0.63737387	0.792948213
Smg9	5	0.499004975	0.454545455	-0.044459521	0.6377275	0.793259415
Vps29	39	1.112540284	1.053647519	-0.058892765	0.63773213	0.793259415
Sympk	95	1.331091575	1.382115522	0.051023947	0.63798846	0.793443733
Cstb	19	1.526866279	1.974600668	0.447734389	0.63794967	0.793443733
Rpl6	29	0.746439955	0.833242032	0.086802077	0.63831539	0.793783055
Top1	64	1.237772609	1.216815924	-0.020956684	0.63839703	0.793817303
Dffa	17	0.675920234	0.92464029	0.248720056	0.63868649	0.794109947
Ddx28	6	0.510738272	0.790733591	0.279995319	0.63882182	0.794210915
Ppap2c	27	1.519429961	1.655080506	0.135650545	0.63900782	0.794366234
Cpsf4	20	1.735196527	1.399769784	-0.335426743	0.63905502	0.794366234
Fam8a1	48	1.285044519	1.187881354	-0.097163165	0.63918797	0.794431956
Utp18	59	1.100461642	1.001572634	-0.098889008	0.63921617	0.794431956
Plin2	4	2.672198016	2.82249742	0.150299404	0.63952973	0.794485208
ChkbCpt1b	26	1.208207894	1.133067644	-0.07514025	0.63947918	0.794485208
Galnt12	35	1.677051678	1.334472103	-0.342579575	0.63952178	0.794485208
Chkb	26	1.208207894	1.133067644	-0.07514025	0.63947918	0.794485208
Mdh2	62	1.205937654	1.407898867	0.201961213	0.63942415	0.794485208
Vps8	55	1.04842574	1.204468658	0.156042919	0.63987919	0.794784772
Slc27a6	3	42.46762558	41.83895338	-0.628672196	0.63983139	0.794784772
[illegible]19Bwg1357	32	1.870566047	1.852412918	-0.018153129	0.63993964	0.794792578
Fdx1l	38	1.40640953	1.47594052	0.06953099	0.64023495	0.795092044
Ctsd	50	1.196430337	1.149196868	-0.047233469	0.64036592	0.795187397
Igfbp4	19	1.905162925	1.656709419	-0.248453506	0.64049739	0.795283351
Cfb	4	64.99409458	62.18511878	-2.808975797	0.64073714	0.795513738
Tacc3	28	0.818762819	0.865994966	0.047232147	0.6408658	0.795538846
Unk	21	1.292517899	1.625486737	0.332968838	0.64082828	0.795538846
Creb3	11	1.742951112	1.695779803	-0.047171309	0.64095458	0.795581749
Ntan1	15	0.456619278	0.345147716	-0.111471562	0.64109669	0.795690842
Klhl21	26	1.079562428	0.917394817	-0.162167611	0.64115945	0.795701438
Oraov1	21	1.560758777	1.161385884	-0.399372894	0.64121786	0.795706626

Mkks	13	1.015816779	0.768890004	-0.246926776	0.64168412	0.796150571
Slx4ip	13	1.015816779	0.768890004	-0.246926776	0.64168412	0.796150571
Zbtb40	12	1.536879796	1.315992761	-0.220887036	0.64179359	0.796151757
Zfp11	24	1.917322277	1.831538686	-0.085783592	0.64177878	0.796151757
Pcbp1	178	1.164301984	1.212651999	0.048350015	0.64193418	0.796175276
Rexo4	29	1.357722625	1.314582598	-0.043140026	0.64208384	0.796175276
Afmid	4	1.633986928	1.609170306	-0.024816622	0.64205111	0.796175276
Rfx2	73	0.953807199	1.036119584	0.082312384	0.64207563	0.796175276
Parg	27	1.1217648	0.88258939	-0.23917541	0.64206785	0.796175276
Triobp	73	1.500014729	1.541162008	0.041147279	0.64222749	0.796286119
Adam23	58	17.58851797	20.36813936	2.779621397	0.64231674	0.796329495
Actg1	62	1.132939939	1.171153537	0.038213597	0.64298274	0.796875469
Ezr	84	1.355007544	1.16950935	-0.185498194	0.64284061	0.796875469
Wdr91	41	2.120239164	2.287098137	0.166858973	0.64299625	0.796875469
Cpsf3l	39	0.971792949	0.904479057	-0.067313892	0.64293051	0.796875469
Surf4	16	1.336597997	1.403241737	0.06664374	0.64302865	0.796875469
Ppapdc3	5	72.56152329	68.43154814	-4.129975146	0.64341355	0.797264959
Lsm11	38	1.45667126	1.288493799	-0.168177461	0.64345161	0.797264959
Pdia6	50	5.207845094	5.227019699	0.019174605	0.64379615	0.797624514
Cdc42ep2	7	2.515645693	2.53510339	0.019457697	0.64397848	0.797783051
Atox1	40	4.987378935	5.139903502	0.152524567	0.64412701	0.797874203
Tmem138	20	2.548873477	2.065721944	-0.483151533	0.64426955	0.797874203
Krtap17-1	6	58.40641709	57.13554949	-1.270867598	0.64424191	0.797874203
Smek1	74	0.915682433	1.08572941	0.170046978	0.64416857	0.797874203
Cenpt	12	0.699672668	0.399553343	-0.300119325	0.64437414	0.797884429
Mdk	23	43.46192165	43.31634953	-0.145572115	0.64438656	0.797884429
Pfdn1	14	1.320221255	1.457250611	0.137029356	0.6445498	0.797951891
F11r	18	1.322843852	1.024838135	-0.298005717	0.64450969	0.797951891
Rasal2	30	1.075125573	1.285410336	0.210284763	0.64477044	0.798090384
Strn3	73	1.754406582	1.320123592	-0.43428299	0.64473955	0.798090384
Gcdh	23	1.854574876	1.933885094	0.079310218	0.64525259	0.798602117
Aqp11	32	1.39087554	1.311650962	-0.079224579	0.64529272	0.798602117
Gdap2	35	1.745095303	1.561749444	-0.183345859	0.64565954	0.798988708
410002F23R	48	0.917032966	0.9713426	0.054309634	0.64577765	0.799067483

Pcdhgb1	3	58.51975981	57.15913681	-1.360623003	0.64597765	0.799247553
Zfp830	26	1.400967206	1.619280038	0.218312832	0.64620523	0.799369504
Lgr4	102	1.552742403	1.281823672	-0.270918731	0.64623964	0.799369504
Wibg	30	1.116745478	1.481485495	0.364740017	0.64615178	0.799369504
Nf2	57	1.603107035	1.430830962	-0.172276073	0.64638952	0.799420123
Egln3	37	1.943536601	1.966132467	0.022595866	0.64635667	0.799420123
Pla2g12b	2	90.94977022	91.52034772	0.570577503	0.64656612	0.799571145
Cd151	27	2.166408449	1.74877567	-0.417632779	0.64702549	0.800037465
Ano8	20	1.761230309	1.595744935	-0.165485374	0.64705224	0.800037465
.30024G19F	6	7.86384199	8.383990891	0.520148902	0.64730515	0.80028274
BC089491	36	20.84758076	22.30043352	1.452852757	0.64741368	0.800349477
Ablim2	48	1.748769237	2.32117474	0.572405503	0.64762188	0.800361337
Ephb1	34	33.61293799	33.35705636	-0.255881627	0.64768182	0.800361337
Senp6	38	1.542291646	1.738593405	0.196301759	0.64769598	0.800361337
Tpt1	96	1.374163579	1.455501704	0.081338125	0.64749595	0.800361337
Uspl1	5	1.697971273	1.560113713	-0.13785756	0.64760064	0.800361337
Ufd1l	8	2.474395501	2.237546939	-0.236848562	0.64776564	0.8003695
Itpr3	33	1.291751149	1.241103434	-0.050647715	0.64781988	0.8003695
Nipal2	38	7.856630463	8.438381231	0.581750769	0.64786622	0.8003695
Map1s	53	1.120521315	1.280992636	0.16047132	0.64794177	0.800395444
Jmjd6	43	1.442222642	1.306415774	-0.135806868	0.64800149	0.800401838
Aatk	30	31.1683545	31.00665764	-0.161696859	0.64814315	0.800509436
Fam43b	6	15.08470696	15.02312761	-0.061579348	0.64862004	0.801031009
Pygm	10	79.85207863	78.52413998	-1.32793865	0.6490094	0.801444416
Cad	45	3.752875659	3.740977937	-0.011897722	0.64917764	0.801517262
Mef2d	61	1.122368773	1.127799194	0.005430421	0.6491306	0.801517262
Tbc1d14	43	1.345707819	1.723503748	0.377795929	0.64929366	0.801580027
Crebzf	92	0.981568058	1.017426685	0.035858626	0.64933773	0.801580027
Stxbp1	32	0.847165066	0.793735459	-0.053429607	0.64955299	0.801719087
Pet100	5	1.196039604	2.032008632	0.835969028	0.64955964	0.801719087
Tmc7	16	1.335444711	1.063133816	-0.272310895	0.64964823	0.80172827
Zfp46	23	1.839018649	1.534752078	-0.304266571	0.64967636	0.80172827
Ccdc159	14	1.118752127	1.294460494	0.175708367	0.64990994	0.801881653
Csgalnact2	10	1.020666425	1.418996011	0.398329586	0.64990217	0.801881653

Ube2s	17	0.998038332	0.778925847	-0.219112486	0.6500495	0.801986409
Ikbkg	5	11.76152981	11.12991736	-0.631612453	0.65011867	0.802004314
Gars	36	0.966274543	0.861080982	-0.105193562	0.65041078	0.802297224
Rsl24d1	41	1.481247122	1.369340712	-0.11190641	0.65050614	0.802347404
Wls	3	2.557251908	2.639178392	0.081926483	0.65066992	0.802481963
C4b	2	47.57225434	46.1195542	-1.452700131	0.65077903	0.802549083
Amfr	90	1.131131753	1.1519268	0.020795047	0.65091888	0.802654091
Gm15787	17	1.317296807	1.496050614	0.178753807	0.65158478	0.803407702
Stx4a	39	1.394938684	1.218173172	-0.176765513	0.65176627	0.803496449
Ubiad1	13	1.784193327	1.302792325	-0.481401002	0.65173654	0.803496449
Cmtr2	20	1.906221959	1.59552201	-0.31069995	0.65191546	0.803612858
Nme3	6	1.9457112	0.873728326	-1.071982874	0.65219067	0.803884574
Foxk1	69	1.062050773	0.951315632	-0.110735141	0.65302757	0.804411026
Slc7a15	4	95.17343268	94.5433624	-0.630070279	0.65280159	0.804411026
Nxpe3	74	1.231996955	1.246823169	0.014826214	0.6532756	0.804411026
Als2	51	1.309991082	1.323603567	0.013612485	0.6531639	0.804411026
921524J17R	21	1.146113615	0.846778641	-0.299334974	0.6532549	0.804411026
310021J22R	8	1.287673297	1.26546892	-0.022204377	0.65321499	0.804411026
Nt5dc3	50	1.553167413	1.169194967	-0.383972446	0.65267871	0.804411026
Gm10778	3	1.786067134	1.877934272	0.091867138	0.6529902	0.804411026
Syngr1	39	6.288139373	5.199880375	-1.088258997	0.65316813	0.804411026
Serf1	64	1.355152795	1.113789175	-0.24136362	0.65286025	0.804411026
Helq	58	1.672342446	1.618167115	-0.054175331	0.6529388	0.804411026
Ssr2	8	1.756705589	1.576412154	-0.180293436	0.65321417	0.804411026
Tmco3	27	1.450331505	1.248575525	-0.20175598	0.65341292	0.804512597
Mtmr2	43	0.931407586	0.833332983	-0.098074603	0.65348042	0.804528206
Adss	55	1.138803479	1.111464632	-0.027338847	0.65368742	0.804715538
Lmod1	17	70.90720114	68.52852076	-2.378680377	0.65378945	0.804759202
Acvr2a	62	0.920499106	0.649697642	-0.270801464	0.65383258	0.804759202
Fkbpl	2	3.389830508	2.127659574	-1.262170934	0.65390269	0.804778004
Samd1	161	1.336537282	1.347010369	0.010473087	0.65411527	0.804972115
Itsn2	116	1.283716574	1.139078042	-0.144638532	0.65432965	0.805100893
Phlpp2	68	1.409530152	1.229498832	-0.18003132	0.65431346	0.805100893
Necap1	23	1.201652782	1.384324193	0.182671411	0.65444821	0.805111757

Bms1	39	1.087218017	1.007132551	-0.080085466	0.65441279	0.805111757
Nuak2	11	0.528007346	0.679740578	0.151733232	0.6548115	0.805221146
Eif4b	21	1.777605259	1.568239874	-0.209365385	0.65478291	0.805221146
Cpox	25	1.140544775	1.597783187	0.457238412	0.65469226	0.805221146
Ube4b	20	1.848637085	1.693955933	-0.154681153	0.65472537	0.805221146
Mapk1ip1l	70	1.252450897	1.397734376	0.145283479	0.65469248	0.805221146
Srrm1	71	1.256350458	1.125363524	-0.130986933	0.65513643	0.80548571
Bahcc1	22	1.214943603	1.305324876	0.090381274	0.65513224	0.80548571
Vamp2	61	0.969218686	0.975239107	0.006020421	0.6552241	0.805526007
Ankle1	27	1.494740599	1.303751998	-0.190988601	0.65555387	0.80579641
Fnbp1l	100	1.34867767	1.190381683	-0.158295987	0.65551047	0.80579641
Nsun2	34	1.407836069	0.909957869	-0.4978782	0.65567305	0.80587539
Zfp553	41	0.93173146	0.895530365	-0.036201095	0.65577287	0.805921639
Zdhhc20	96	1.183588809	1.196858304	0.013269495	0.65582052	0.805921639
Slc30a4	89	0.895568519	0.909797676	0.014229157	0.65605258	0.805970052
Trp53i13	57	1.974219535	2.046769885	0.07255035	0.65600125	0.805970052
Mov10	48	1.777216854	1.645476774	-0.13174008	0.65613454	0.805970052
Gltscr1l	3	93.61128311	93.67737526	0.066092147	0.655984	0.805970052
Kif9	10	1.912003961	1.861079146	-0.050924815	0.65610259	0.805970052
BC005561	42	1.020316765	0.750850011	-0.269466754	0.65624982	0.806044179
Fkbp4	62	1.187242178	1.067226509	-0.120015669	0.65669069	0.806450678
'00086O06F	5	1.28440367	0.828402367	-0.456001303	0.65665298	0.806450678
Crabp2	8	10.3125192	10.54598954	0.233470337	0.65708869	0.806736906
Actr2	86	1.533292211	1.41739864	-0.115893571	0.65698081	0.806736906
Wnt5a	52	24.77672751	26.42266402	1.645936506	0.65706179	0.806736906
Psmc3	7	1.10211384	1.189546202	0.087432362	0.6572965	0.806924525
Slc27a4	73	1.339190089	1.210577029	-0.12861306	0.65736937	0.806946472
Cryz	21	0.86661299	1.285914081	0.419301092	0.65748332	0.807018836
Apopt1	34	0.974606678	1.268124132	0.293517455	0.65759161	0.807084253
Tsen34	26	2.06853935	1.42015981	-0.64837954	0.65781337	0.807153896
'00021K19F	11	2.073224694	1.84203172	-0.231192974	0.65777978	0.807153896
Hnrnph2	9	15.29387062	14.8742333	-0.419637322	0.65772706	0.807153896
Atp5g2	24	1.020693542	0.752864514	-0.267829027	0.65799121	0.807304597
Atg4c	7	1.385927505	1.643934169	0.258006664	0.65850877	0.80761389

Rccd1	13	0.647319	0.679060985	0.031741985	0.65869989	0.80761389
Phf21a	137	1.509731784	1.376745584	-0.1329862	0.65859718	0.80761389
Etohd2	17	1.58626347	1.396236107	-0.190027363	0.65829864	0.80761389
Cyth2	45	1.190242098	1.115393121	-0.074848976	0.65873863	0.80761389
Btf3l4	44	1.46422611	1.307490066	-0.156736044	0.65850092	0.80761389
Dcakd	29	0.797824023	0.811174013	0.01334999	0.65869847	0.80761389
Tmem203	21	1.004681949	0.996945675	-0.007736274	0.65837063	0.80761389
532415L05R	34	1.31754559	1.511557074	0.194011484	0.65862654	0.80761389
Ccdc138	27	2.049906882	1.77063611	-0.279270772	0.65905873	0.807938829
Vkorc1	8	16.10749164	15.96445659	-0.143035048	0.65932916	0.808067829
Anp32a	24	1.189372083	1.400163556	0.210791473	0.65928889	0.808067829
Sec22c	20	1.479568295	1.619602054	0.140033759	0.65921927	0.808067829
Cr1l	19	1.474939647	1.619949605	0.145009958	0.65946004	0.808160732
Prickle4	17	2.519416249	2.718883084	0.199466835	0.65973059	0.808394578
Zfp777	307	1.110647004	1.098173266	-0.012473739	0.65979113	0.808394578
Ttll1	23	3.118013967	3.052726216	-0.06528775	0.65981613	0.808394578
Gipc1	42	0.77668724	0.765211703	-0.011475537	0.66016143	0.808750113
Lrp2bp	1	44	48	4	0.6602591	0.808802234
Sgsh	19	1.063180497	1.236492578	0.173312082	0.66032195	0.80881171
Bdnf	108	16.34271363	18.57021434	2.227500702	0.66041967	0.808863893
930549G23R	16	1.530199181	1.233557885	-0.296641295	0.66050187	0.808894955
Vps72	10	2.298228977	2.037688279	-0.260540698	0.66055528	0.808894955
Stx12	36	1.582854907	1.306959929	-0.275894978	0.66063481	0.808924832
Cflar	26	0.92553172	0.734521592	-0.191010129	0.66070027	0.808937485
P2rx4	9	0.664437586	0.433922167	-0.230515418	0.6608396	0.809004686
Mapkap1	22	0.795539678	0.890855996	0.095316319	0.66086542	0.809004686
930059L03R	13	1.658741216	1.821918981	0.163177765	0.6609236	0.809008417
Gm6225	10	1.217428442	1.045585776	-0.171842666	0.66098877	0.809020699
510039K10R	60	1.0867726	1.221051578	0.134278978	0.66125612	0.809145458
Cep19	20	1.210350505	1.627471151	0.417120646	0.66116401	0.809145458
Cep164	13	0.862477473	1.206549911	0.344072438	0.66124132	0.809145458
Kif5a	13	5.253558179	6.189516618	0.93595844	0.66136944	0.809216637
930506C21R	16	0.936006671	1.122555508	0.186548837	0.66142704	0.809219643
Tmem18	19	1.382900994	1.397128262	0.014227268	0.66149019	0.80922943

)31440P22F	58	1.693469605	1.437488523	-0.255981082	0.66160873	0.809306973
Psmc4	11	1.405221724	1.137149777	-0.268071948	0.66182701	0.809506511
Pgls	46	1.307745893	1.287491138	-0.020254755	0.6619284	0.809563038
Ncapd2	57	1.581923148	1.51637486	-0.065548287	0.6622652	0.809907456
Chst12	5	1.016144349	1.608391608	0.592247259	0.66240279	0.809969195
Camta1	199	1.419366432	1.346075296	-0.073291136	0.66242608	0.809969195
Ngly1	29	1.318863377	1.189504301	-0.129359075	0.66275504	0.810101409
Nemf	14	1.523879735	1.942172006	0.418292271	0.66273206	0.810101409
Bivm	4	3.121091176	3.671790673	0.550699497	0.66261746	0.810101409
Vipr2	23	3.362760086	3.699935046	0.33717496	0.66268818	0.810101409
Pramef12	4	90.14736524	89.86143559	-0.285929651	0.66291352	0.810160151
Rbm4	20	1.8892772	1.855037628	-0.034239572	0.66286481	0.810160151
Cbx6	63	1.100629017	1.110611385	0.009982368	0.66370256	0.810989377
Npcd	63	1.100629017	1.110611385	0.009982368	0.66370256	0.810989377
Mir132	23	1.550705228	1.400273682	-0.150431546	0.66377188	0.811006547
Mns1	8	1.797093029	1.449856176	-0.347236853	0.66402397	0.811247007
Dnal4	33	1.140156677	1.14788172	0.007725043	0.66409871	0.811270773
Kif22	41	1.5561768	1.442134563	-0.114042237	0.6647452	0.811435873
Rbm34	10	2.595300887	1.173195615	-1.422105272	0.66465017	0.811435873
Epg5	24	1.526188846	1.392352568	-0.133836278	0.66478114	0.811435873
Arxes2	5	33.25212208	32.74750338	-0.504618698	0.66480909	0.811435873
Crlf3	46	1.437973229	1.484356493	0.046383264	0.66466245	0.811435873
Dclre1b	24	1.340524086	1.126886008	-0.213638078	0.66478261	0.811435873
Dnajc4	8	2.204370011	2.047214256	-0.157155755	0.66436287	0.811435873
Gca	26	3.540499614	3.850357304	0.30985769	0.66484214	0.811435873
Wipi1	30	0.809549963	0.795140944	-0.014409019	0.6647184	0.811435873
Fam188a	25	1.772888934	1.90623538	0.133346445	0.66433685	0.811435873
Gga1	44	1.101425718	1.07409371	-0.027332008	0.66469787	0.811435873
Nid2	24	48.76422246	48.40432135	-0.359901109	0.6652737	0.811593966
Xndc1	19	1.64550745	1.332120373	-0.313387078	0.66531787	0.811593966
Mef2a	114	1.616555088	1.552713972	-0.063841116	0.66551728	0.811593966
Usp6nl	142	1.30877697	1.124461061	-0.184315909	0.66540477	0.811593966
Sac3d1	38	0.817533291	0.649726519	-0.167806772	0.66553909	0.811593966
Grpel1	20	1.062250052	1.076359131	0.01410908	0.66510905	0.811593966

Krt39	1	86.11111111	88.46153846	2.35042735	0.66558006	0.811593966
Lss	18	1.230942797	0.948045238	-0.282897559	0.66518056	0.811593966
Cpd	59	0.876364291	0.952232986	0.075868694	0.66553111	0.811593966
Zfp202	51	1.455663982	1.374215871	-0.081448111	0.66535228	0.811593966
Xntrpc	19	1.64550745	1.332120373	-0.313387078	0.66531787	0.811593966
Metrnl	48	1.749689174	1.639461601	-0.110227573	0.66593811	0.811902947
Spin1	140	1.317441739	1.220879491	-0.096562248	0.66605477	0.811902947
Trmt61b	63	1.602043754	1.481088024	-0.12095573	0.66602442	0.811902947
Spns2	95	2.009952436	1.73330016	-0.276652277	0.66599661	0.811902947
Rnf8-cmtr1	20	1.10919375	0.9332024	-0.17599135	0.66619597	0.811940171
Rnf8	20	1.10919375	0.9332024	-0.17599135	0.66619597	0.811940171
Cyp2u1	23	3.716953445	3.939227738	0.222274293	0.66630174	0.812001638
Sf3a2	66	1.192508282	0.967261849	-0.225246433	0.66639694	0.812050221
Ercc5	34	1.801345374	2.011128986	0.209783612	0.66658986	0.812104605
Raf1	80	0.951189518	1.02721262	0.076023102	0.6666076	0.812104605
Ppp1r9b	51	1.311364673	1.1431514	-0.168213273	0.66653876	0.812104605
Rps3	20	1.1732373	1.424370809	0.251133509	0.66677587	0.812154966
Cox7a2l	15	1.573454539	1.194566684	-0.378887855	0.66674575	0.812154966
Hal	5	53.42277433	56.14387856	2.721104232	0.66681498	0.812154966
Rps27a	4	3.172381384	3.845085113	0.672703729	0.66698061	0.812289273
Lnp	33	1.215437211	1.138449368	-0.076987843	0.66718068	0.812465502
Tep1	16	1.352217695	1.046520072	-0.305697623	0.66752703	0.812819816
Tmem128	20	0.780261173	0.70367199	-0.076589183	0.66765197	0.812904492
Ptgr2	16	1.617229205	1.267175387	-0.350053818	0.66798749	0.813245536
Dpep3	5	97.41094596	96.89642572	-0.514520241	0.66809977	0.813314749
Jak1	68	1.390031834	1.440498792	0.050466959	0.66837303	0.813579916
Qpctl	7	1.171858016	1.316569039	0.144711023	0.66885808	0.814102809
Noxo1	53	1.630255779	1.617956929	-0.012298849	0.66891683	0.814106786
630001P10R	6	10.45045045	9.829059829	-0.621390621	0.66899272	0.814123385
Slc29a3	17	1.957788408	1.956236851	-0.001551558	0.66904143	0.814123385
Abi1	29	1.011448789	1.156223633	0.144774844	0.66918124	0.814225998
Zfp597	32	2.45363347	2.320527119	-0.133106351	0.66945005	0.814485538
Jag1	108	1.203360648	1.106407265	-0.096953383	0.66963985	0.814648907
Polr3g	50	1.568628407	1.476477651	-0.092150756	0.66973521	0.814697377

Rad23a	34	1.371252877	1.365362051	-0.005890826	0.66990317	0.814834139
Abca5	10	1.02325211	0.814794216	-0.208457894	0.66998655	0.81486802
Myo9a	53	1.469309215	1.398382454	-0.070926761	0.67022091	0.814882889
Dancr	54	1.6815931	1.877910318	0.196317218	0.67017232	0.814882889
Pgm2l1	41	1.391034522	1.212227046	-0.178807476	0.67013799	0.814882889
Pkmyt1	86	1.406871911	1.342664939	-0.064206972	0.67007388	0.814882889
Fbxl14	145	1.041933805	1.042599622	0.000665817	0.67053395	0.815195956
Ptms	101	1.382118618	1.305819996	-0.076298622	0.67077727	0.815221605
Lyst	70	1.627476309	1.430092349	-0.19738396	0.67074451	0.815221605
Rad9a	25	1.142561126	1.225865575	0.08330445	0.67067992	0.815221605
Epc1	73	1.17925739	0.939003952	-0.240253438	0.67075757	0.815221605
Trim8	104	1.170612492	1.259837159	0.089224668	0.67083832	0.815228277
Nup35	37	1.947767015	1.743599867	-0.204167148	0.67104078	0.815406787
Txn2	21	2.229391964	2.317758636	0.088366672	0.6715165	0.815917296
Trib2	42	3.281029803	3.2241981	-0.056831702	0.6716302	0.815987873
Ajuba	51	1.538631303	1.558538034	0.019906731	0.67179383	0.816060551
Tmem160	45	1.211251916	1.041706546	-0.16954537	0.67180124	0.816060551
Cenpv	79	1.252024635	1.272418335	0.0203937	0.67214768	0.816413793
Mettl15	1	5.882352941	4.954954955	-0.927397986	0.67239282	0.816643949
Ap2a2	60	1.274598533	1.106297622	-0.168300911	0.67271802	0.816971305
Enah	182	3.424693096	3.754875582	0.330182486	0.67296615	0.817205002
Rchy1	40	1.118117788	1.079799681	-0.038318107	0.6733004	0.817407969
Ercc6	28	1.507541413	1.460201649	-0.047339764	0.67329448	0.817407969
Trappc9	36	1.64719624	1.466230665	-0.180965575	0.67325021	0.817407969
Cend1	16	51.60344442	53.09304273	1.489598303	0.67347698	0.817554697
Wnk1	77	1.802360958	1.888871756	0.086510797	0.67362099	0.817603983
Fam210a	33	0.906079255	0.697195102	-0.208884153	0.67362901	0.817603983
Lsm5	13	1.139845647	1.092978098	-0.04686755	0.67383115	0.817715289
700101I11R	3	3.212498388	4.75943839	1.546940001	0.67383217	0.817715289
Arglu1	29	1.209889673	0.925779468	-0.284110205	0.67406086	0.817925163
Car4	8	26.40010255	26.92048651	0.520383954	0.6741319	0.817943728
Pknox1	121	1.098660575	0.964224589	-0.134435986	0.67430285	0.818083509
Rps19bp1	14	1.586875411	1.094692943	-0.492182469	0.67439241	0.818118474
Iqgap2	81	1.300530807	1.093923365	-0.206607443	0.67444318	0.818118474

Ppm1d	55	1.031374677	1.012161791	-0.019212886	0.67472207	0.818389123
Mrpl17	30	1.331104614	1.138683312	-0.192421302	0.67507975	0.818687621
Fut11	54	1.037316447	1.063505005	0.026188558	0.67503108	0.818687621
Otx2os1	7	36.03470991	36.68479018	0.650080264	0.67525807	0.818716928
Fam32a	42	1.589188657	1.517427391	-0.071761266	0.67536377	0.818716928
Med10	33	1.815423784	1.810855753	-0.004568031	0.67546253	0.818716928
Atad2b	22	1.111047695	1.120181193	0.009133498	0.67533419	0.818716928
Atp6v1h	28	1.238700713	0.993089505	-0.245611208	0.67542926	0.818716928
Rfx1	54	1.174686121	1.078752629	-0.095933492	0.67549447	0.818716928
Alad	44	1.23157913	1.17018973	-0.061389399	0.67521374	0.818716928
Sf1	49	1.276798757	1.015216988	-0.261581769	0.67557533	0.81874731
Zfand2a	25	1.684180266	1.754611573	0.070431308	0.6756333	0.818749944
Drg2	13	1.452202269	1.386604412	-0.065597857	0.67578642	0.818867873
Tmco4	3	1.222332034	1.804417122	0.582085088	0.67587333	0.818905562
Eif4a2	55	1.226422283	0.942748089	-0.283674194	0.67608492	0.819028968
Ndufs8	12	0.965281959	0.631123084	-0.334158875	0.67608681	0.819028968
Ell2	39	1.195209429	1.085483068	-0.109726361	0.67643169	0.819311482
Mxd4	32	0.92718896	0.931609142	0.004420182	0.67638039	0.819311482
Crcp	32	1.372149875	1.532633683	0.160483808	0.67689281	0.819802331
Cenpo	25	1.679058492	1.229292945	-0.449765548	0.67727627	0.820131374
Prpf18	14	1.460020218	1.492108963	0.032088745	0.67727497	0.820131374
Serhl	4	2.977190876	2.996422182	0.019231306	0.67772516	0.820404138
Pla2g6	10	2.741142845	2.233285811	-0.507857035	0.67758497	0.820404138
Mbtps1	97	1.128907604	0.881299869	-0.247607735	0.67769142	0.820404138
Myg1	13	22.31641384	22.06862059	-0.247793242	0.6776184	0.820404138
Cmc2	11	0.540350923	0.916923576	0.376572653	0.6780885	0.820776261
E2f5	40	1.17926124	1.075454068	-0.103807172	0.67826362	0.820920522
Snrk	114	1.128625858	1.020492733	-0.108133126	0.67838438	0.820998964
Cpeb4	64	1.262503457	1.26501839	0.002514934	0.67845048	0.821011242
Gtf2a1	142	1.297766552	1.106302224	-0.191464328	0.67854138	0.821053535
Rgl2	61	1.607410922	1.531983068	-0.075427854	0.67869964	0.821177317
Nat6	2	80.41554155	80.50348849	0.087946939	0.67888674	0.821335972
00003M07I	29	22.31755642	21.73131992	-0.586236499	0.67899981	0.821337332
Carkd	37	1.26400445	1.235418376	-0.028586074	0.67899194	0.821337332

Glb1	8	3.240328405	3.572767231	0.332438826	0.67926714	0.821592976
Cdc25b	19	2.073766306	1.411418513	-0.662347794	0.67937068	0.82165049
Exo1	32	1.577427902	1.85163014	0.274202238	0.67962762	0.821893499
Arl15	57	2.954279711	2.691969823	-0.262309888	0.67994467	0.822141402
Mknk2	74	0.939138508	0.952882575	0.013744067	0.67990249	0.822141402
Osbpl3	32	1.323896957	1.188350634	-0.135546323	0.68056641	0.822825367
Smek2	60	1.231753469	1.092242374	-0.139511096	0.68069412	0.822911976
Anp32e	12	2.409430439	1.995021047	-0.414409392	0.68084113	0.823021895
Ap5z1	45	1.355980799	1.280293471	-0.075687329	0.68101378	0.823094984
Olfml2b	43	3.111955356	3.619679607	0.507724251	0.6809935	0.823094984
Polr3f	23	1.660905925	1.323751228	-0.337154697	0.68115493	0.823129987
Pikfyve	21	1.607560622	1.480290354	-0.127270267	0.68113887	0.823129987
AW549877	11	0.838943982	0.712783979	-0.126160003	0.68134259	0.823153395
Wbscr17	42	56.70006428	55.56987915	-1.130185128	0.68124153	0.823153395
Mrpl52	30	1.90921222	1.653650893	-0.255561327	0.68133324	0.823153395
Thoc7	77	0.923252839	0.901687585	-0.021565254	0.68154481	0.823329916
Evx1	8	36.73863169	36.44060131	-0.298030383	0.68169002	0.823369766
10070M22I	41	1.073610288	1.154589331	0.080979043	0.68168524	0.823369766
Kif3c	52	1.762744062	1.649618246	-0.113125816	0.68184913	0.823494164
Bptf	127	1.218152997	1.309515034	0.091362037	0.68212027	0.823665359
Scamp4	37	0.933586836	1.077665519	0.144078683	0.68207647	0.823665359
Chrna5	2	25.62789323	27.35294118	1.725047943	0.68215927	0.823665359
Timm44	9	0.90796679	0.723273455	-0.184693336	0.68238994	0.823876089
Rfesd	8	1.327262083	1.194415457	-0.132846626	0.6825441	0.823994411
Zic1	15	42.27927332	43.45597293	1.176699609	0.68285794	0.824305485
Rspry1	46	1.394671753	1.312988956	-0.081682796	0.6830704	0.82449412
Mir3091	16	1.03265261	0.870442165	-0.162210445	0.68333525	0.82474597
Zc3h8	26	2.321477935	2.048679934	-0.272798001	0.68363401	0.825038696
6-Mar	111	1.037026236	0.9556879	-0.081338336	0.68377734	0.825131125
930426L09R	52	0.899649254	0.90488948	0.005240226	0.68382306	0.825131125
Minpp1	69	1.146060486	1.013716111	-0.132344376	0.68392604	0.825187532
Ppp1r15b	73	0.926218912	0.781092689	-0.145126224	0.68442989	0.825727552
Tbc1d8b	2	22.42194766	21.26618903	-1.155758622	0.68454853	0.82573491
Sh3bp1	27	1.965114504	2.008705839	0.043591335	0.68453326	0.82573491

Zfp142	25	1.148545887	0.85082375	-0.297722137	0.6846898	0.825769554
Stat6	7	2.018226759	1.142238344	-0.875988415	0.68467872	0.825769554
Rexo1	34	1.451726603	1.259047099	-0.192679504	0.68488806	0.82594078
Sepw1	12	0.673064369	0.780292679	0.107228311	0.68497295	0.825975267
Eif4e2	54	1.414196784	1.14033151	-0.273865274	0.68509274	0.826051832
Gm11762	26	1.663802573	1.420828565	-0.242974008	0.68532406	0.826194975
Zfyve16	78	1.237770936	1.068381116	-0.169389821	0.68530762	0.826194975
Sec16a	63	1.467602281	1.292815027	-0.174787254	0.68564961	0.826519541
Slc30a7	39	1.739573824	1.728372615	-0.011201209	0.68591747	0.826774513
Kat2b	50	0.939773479	0.908241418	-0.031532061	0.68600195	0.826808434
Mir7071	50	0.901816166	1.038878596	0.13706243	0.68618257	0.826958196
Abce1	29	0.994924279	1.051940562	0.057016283	0.68635699	0.827032557
Cisd3	35	1.075691039	1.382291804	0.306600765	0.68632939	0.827032557
Ankrd28	137	1.184287457	1.080572719	-0.103714737	0.68662887	0.827292228
Xrcc1	48	1.226138729	1.028519158	-0.19761957	0.68686233	0.82747009
Zfp931	7	3.880589498	3.699171529	-0.181417969	0.68688927	0.82747009
Gpr137	65	1.387442736	1.323775873	-0.063666863	0.68705869	0.827606242
Abca16	6	84.68704966	85.01165875	0.324609087	0.68727537	0.827799287
Aunip	26	1.347723729	1.236713463	-0.111010266	0.68737315	0.827849119
Mgat1	42	2.0019412	1.997292614	-0.004648586	0.68751572	0.827952871
Ireb2	58	1.841840547	1.447189219	-0.394651328	0.68797765	0.828387557
Serf2	73	1.240492476	1.035451903	-0.205040574	0.68798958	0.828387557
Bcap29	57	1.057715045	1.167967605	0.11025256	0.68827815	0.82846309
Zfp330	23	1.484907166	1.087392971	-0.397514195	0.68822355	0.82846309
Nf1	80	1.740207329	1.559261395	-0.180945934	0.68817546	0.82846309
Saraf	10	1.730247719	1.823675205	0.093427486	0.68826267	0.82846309
Tbck	22	0.811439122	0.763864647	-0.047574475	0.68840401	0.828546624
Kdm1b	67	1.705429226	1.496814918	-0.208614307	0.68855388	0.828659037
Cox10	16	1.482806296	1.74136872	0.258562424	0.688662	0.828721188
Bhlhe41	61	2.139367665	2.015709396	-0.123658269	0.68872314	0.828726803
Cenpb	48	1.205891734	1.319285682	0.113393948	0.68888957	0.828859101
Gnptg	12	1.377060098	1.619859497	0.242799399	0.6889896	0.828911483
Bpgm	14	1.946927942	1.721024141	-0.225903801	0.68909183	0.82891895
430405H02F	39	1.15909712	1.007407113	-0.151690006	0.68910878	0.82891895

Ankrd34b	23	12.03481308	11.28486503	-0.749948052	0.68939052	0.829163699
Zc3h18	112	1.284842385	1.236223078	-0.048619306	0.68942526	0.829163699
Sh3d19	4	98.43638982	98.09355255	-0.34283726	0.68950028	0.829185965
Ggact	68	1.307134614	1.40219127	0.095056656	0.68956582	0.829196822
Gins2	9	2.327208255	1.994919849	-0.332288406	0.6896925	0.829213257
Exoc6	70	1.291608628	1.345017375	0.053408746	0.6896695	0.829213257
Tmem64	91	1.167969114	1.012721463	-0.155247651	0.69011099	0.829648426
Pea15a	21	1.491355699	1.608618522	0.117262822	0.69034944	0.829867099
Tmf1	86	1.338831007	1.224715079	-0.114115928	0.69048773	0.829965352
Txnl1	34	1.353101829	1.366390802	0.013288973	0.69085883	0.83034339
Strn4	17	1.911066914	1.645544099	-0.265522816	0.6911951	0.830651013
Pdcl	4	0.235849057	0.555555556	0.319706499	0.69122799	0.830651013
Mmp17	50	2.311856757	2.053587753	-0.258269005	0.69130202	0.830671951
Tmem230	21	1.087711508	1.063516528	-0.02419498	0.69143507	0.830763791
Cpt1a	12	0.688749798	1.136177956	0.447428158	0.69154405	0.830797586
Pole	26	1.719338337	1.447582754	-0.271755583	0.69157643	0.830797586
Cry1	143	1.093023966	0.99500102	-0.098022946	0.69175777	0.830947412
Wdr41	18	1.238734152	1.08312356	-0.155610592	0.69196624	0.831129791
Fadd	48	1.314274758	1.582027036	0.267752278	0.69208992	0.83114228
Ercc3	13	1.349672064	1.249657149	-0.100014915	0.69206692	0.83114228
Flywch2	10	3.382312925	1.695156695	-1.68715623	0.69222944	0.831173786
Phf1	28	1.292347315	1.328114746	0.035767432	0.69218544	0.831173786
Ankrd44	94	2.063709881	2.273227357	0.209517476	0.69257612	0.831427733
)30453N24F	19	1.832343507	1.659059164	-0.173284343	0.69259909	0.831427733
Zc3h7b	22	1.358153936	1.126828139	-0.231325798	0.69261092	0.831427733
Wipf1	82	8.203252711	9.087116863	0.883864152	0.69299341	0.831598052
Fbxl8	24	1.411766452	1.122487189	-0.289279263	0.69310032	0.831598052
Iars	4	22.56681178	24.31286222	1.746050448	0.69292354	0.831598052
Zfp799	5	1.584891252	1.768925558	0.184034306	0.69331952	0.831598052
Map4k3	104	2.035742721	1.637285095	-0.398457627	0.69312957	0.831598052
Eif1	10	0.261693963	0.458715596	0.197021633	0.69296694	0.831598052
Tubgcp6	31	1.087206531	1.085564154	-0.001642377	0.69327142	0.831598052
Mios	89	1.108153839	1.015354652	-0.092799187	0.69284136	0.831598052
Apaf1	77	1.261784728	1.245056377	-0.016728351	0.69326217	0.831598052

Setd5	61	1.450222527	1.214121369	-0.236101158	0.69318243	0.831598052
Klhdc10	51	1.458644283	1.433990664	-0.024653619	0.69347631	0.831718134
Mir212	54	1.820738973	2.17979784	0.359058868	0.69359968	0.831798113
310037I17R	42	1.094053299	1.074741868	-0.019311432	0.6937366	0.831894328
Stx5a	4	1.252819791	1.108538059	-0.144281732	0.69406733	0.832222912
Spg7	86	1.281159748	1.265396375	-0.015763373	0.6943217	0.832459891
Abhd6	4	2.001188171	1.369215635	-0.631972536	0.69440054	0.832486401
Pik3r2	55	1.533220488	1.334520987	-0.198699501	0.69451576	0.832556516
Apbb3	6	0.27100271	0.600600601	0.329597891	0.6947211	0.832734634
Rhobtb1	58	1.190985426	1.249377323	0.058391896	0.69482619	0.83279258
Mrpl4	36	1.322299289	1.179861937	-0.142437353	0.69519261	0.833163696
Lsm12	38	0.79202923	1.188200005	0.396170774	0.69544369	0.83339655
Zfp428	38	1.430288485	1.066141253	-0.364147232	0.69586454	0.833832778
Ccdc125	22	1.525817969	1.018438578	-0.507379391	0.69595299	0.833870673
Nicn1	7	3.237377814	2.458761282	-0.778616531	0.6962606	0.834073358
Cuta	81	1.488505069	1.326993056	-0.161512014	0.69624277	0.834073358
Tmem237	60	1.576688019	1.572127684	-0.004560335	0.69629267	0.834073358
Zfp959	6	1.841620626	0.995653518	-0.845967108	0.69639807	0.834131517
Dusp11	4	3.106261655	2.999465258	-0.106796397	0.69686045	0.83454006
Il17rd	82	1.724680129	1.630769126	-0.093911004	0.69690977	0.83454006
Trappc11	8	1.072937088	1.541749064	0.468811976	0.69684203	0.83454006
Nbn	64	1.399321682	1.257546594	-0.141775088	0.69705959	0.834606448
Hjurp	5	2.110315524	1.605930571	-0.504384953	0.69707896	0.834606448
Enoph1	81	0.818714908	0.788008676	-0.030706231	0.69716142	0.834637084
Prr23a2	13	84.71101803	84.21920272	-0.491815308	0.69730553	0.834741509
Xpo7	111	1.064140385	0.945927396	-0.118212988	0.69744016	0.834766467
Mir290a	3	85.43891958	84.70004669	-0.738872897	0.69743124	0.834766467
Aktip	49	3.455888451	2.967853179	-0.488035272	0.69782728	0.835161698
Isg20l2	32	0.964341432	1.13729138	0.172949947	0.69830254	0.835662332
Arfgap1	70	1.673414154	1.607891024	-0.06552313	0.69852825	0.835805544
Nup133	42	1.254090746	1.00311366	-0.250977086	0.69854009	0.835805544
Tmem141	10	1.882022511	2.247280811	0.3652583	0.69859309	0.835805544
Cep63	54	1.248406037	1.100371326	-0.148034711	0.69881323	0.836000758
Sap30l	80	0.995088649	0.974870628	-0.02021802	0.69913245	0.836295199

Ankrd45	12	55.64376405	54.65241532	-0.991348725	0.69923273	0.836295199
Srfbp1	48	1.182614615	0.920476856	-0.262137758	0.69923692	0.836295199
Kif15	8	2.029429879	1.572220413	-0.457209467	0.69928732	0.836295199
Tm9sf2	58	1.380947697	1.168869328	-0.212078369	0.6993498	0.836301765
Pola2	11	0.445842673	0.293880024	-0.151962649	0.69964692	0.83658889
Vdac3	60	1.171532118	1.190265653	0.018733535	0.69978645	0.836687554
Gna12	80	1.034013204	0.941834788	-0.092178416	0.69990134	0.836756741
Srp54a	5	0.779220779	0.842105263	0.062884484	0.70006418	0.83688324
Gm4925	6	1.572327044	0.501289515	-1.071037529	0.70021719	0.836997973
Orc1	30	0.911100101	1.02244715	0.11134705	0.70045407	0.837110494
Ankib1	35	0.815513126	0.734817339	-0.080695787	0.70048162	0.837110494
Hsdl2	37	1.38102995	1.179765471	-0.201264479	0.70048247	0.837110494
Dbndd1	43	2.844105223	3.33478494	0.490679717	0.70055699	0.837125695
Narfl	28	1.229130075	1.214204835	-0.01492524	0.70060929	0.837125695
Ttc33	19	1.427700415	1.548451122	0.120750707	0.70108064	0.837552492
Pacrg	6	0.151515152	0.253518411	0.10200326	0.70105247	0.837552492
Trip12	47	1.533353048	1.842919359	0.309566311	0.7011532	0.83757099
Thoc1	41	1.227119224	1.064432149	-0.162687074	0.70134676	0.837597652
Spata5	52	1.161528778	1.043677838	-0.117850941	0.70129762	0.837597652
Mark3	52	1.142613504	0.991053015	-0.151560489	0.70133776	0.837597652
530052E02R	69	2.884149779	2.378724361	-0.505425419	0.70140749	0.83760201
Alkbh2	19	1.040395358	0.893633117	-0.14676224	0.70172096	0.837635519
Megf11	52	10.68769337	10.63134884	-0.056344523	0.7016179	0.837635519
Dusp6	21	2.173119768	1.843706899	-0.329412869	0.70153627	0.837635519
Malsu1	35	0.863947927	0.919021222	0.055073294	0.7016789	0.837635519
Rpia	49	2.577997533	2.819809261	0.241811728	0.70171311	0.837635519
Cdk5	17	1.318806104	1.369446063	0.05063996	0.70182761	0.83769468
Hist2h2be	8	1.442241382	1.761732245	0.319490863	0.70190751	0.837721904
Zfp414	8	2.672329499	1.756266813	-0.916062686	0.70206409	0.837840636
Arhgef37	21	2.419317677	1.983646661	-0.435671016	0.70217981	0.837910596
Sufu	38	1.046143288	1.003856869	-0.042286419	0.70232019	0.837934067
Supt3	46	1.318599841	0.980065335	-0.338534506	0.70237079	0.837934067
Abcc10	7	1.389940194	2.607585002	1.217644809	0.70230738	0.837934067
Lrp11	50	1.315814622	1.372164746	0.056350124	0.70279613	0.83830519

AI987944	2	2.58096334	3.086419753	0.505456413	0.70274413	0.83830519
Gar1	28	1.425639281	1.358010171	-0.06762911	0.70285476	0.838306983
Ccdc43	34	1.649189413	1.64378881	-0.005400603	0.70303806	0.838457455
Ufl1	36	1.547472472	1.373945259	-0.173527213	0.70333166	0.838517957
Tmem45b	13	20.50405917	21.0375737	0.533514531	0.7033165	0.838517957
Nde1	35	1.199420191	1.140154548	-0.059265643	0.70323343	0.838517957
Blcap	28	1.417164998	1.579144726	0.161979728	0.70343165	0.838517957
Rbm39	25	1.232214376	1.02946063	-0.202753747	0.70340646	0.838517957
Dnmt3a	220	6.692787486	7.491736345	0.798948859	0.70340077	0.838517957
Cep104	54	1.377615431	1.44935416	0.071738729	0.70371048	0.838721055
Cdc42ep4	28	1.382981013	1.207088067	-0.175892946	0.70371634	0.838721055
Tmem100	5	12.15545107	10.24543626	-1.910014812	0.70382692	0.838784721
Itm2b	65	1.441448786	1.185377445	-0.256071341	0.70398704	0.83890741
Esco2	21	1.328587982	1.137862636	-0.190725346	0.70419191	0.839083396
Gm15772	32	1.113001143	1.179148884	0.066147742	0.70436809	0.839225177
Cdk5r2	71	11.01623421	12.19092537	1.174691158	0.70448868	0.839300711
Ppp3cb	23	1.98491296	1.386767888	-0.598145072	0.70470181	0.839486469
Dnajc1	43	1.244219139	1.190023815	-0.054195324	0.70479725	0.839532011
Tfb1m	11	0.786784995	0.645940962	-0.140844033	0.70488515	0.839568558
Mpp2	13	3.993033819	3.398828212	-0.594205607	0.7053059	0.840001529
Idnk	16	3.66267667	2.970031488	-0.692645182	0.70559348	0.840275824
Hn1	30	1.276297667	1.295734155	0.019436488	0.70575567	0.840400776
Mir7049	5	97.2184769	97.15996246	-0.058514442	0.70608146	0.840720492
Pcmtd2	4	0.824175824	0.726982787	-0.097193037	0.70625114	0.840807594
Tnfsf8	1	62.79069767	60.89108911	-1.899608566	0.70626921	0.840807594
Ranbp6	11	1.391959865	1.664750989	0.272791124	0.70644096	0.840943835
Men1	14	2.48951994	2.636453496	0.146933556	0.70674008	0.841095213
AI429214	6	2.925998487	2.751055595	-0.174942892	0.70673182	0.841095213
Ppm1l	113	1.556497171	1.410488591	-0.14600858	0.70670639	0.841095213
Mocs1	10	1.170017245	1.418543154	0.248525909	0.70685936	0.841100728
Rpl35a	6	1.217228464	1.105100003	-0.112128461	0.70682749	0.841100728
Sri	10	2.245570622	1.923604045	-0.321966577	0.70694072	0.841129341
Srp14	43	1.198485266	1.137915991	-0.060569275	0.70714748	0.841307131
Shoc2	72	1.808727975	1.613577222	-0.195150752	0.70721082	0.84131427

Skp1a	65	1.202695682	0.980408582	-0.2222871	0.70731868	0.841374376
;00012H06F	27	1.47215189	1.420971466	-0.051180424	0.70744495	0.841456364
Prkcg	2	12.20043573	10.77586207	-1.424573661	0.7077364	0.841734794
Cep95	26	1.405736149	1.561937331	0.156201182	0.70784863	0.841800052
Taf4b	87	3.104151322	2.813557675	-0.290593648	0.70810739	0.842039529
Mapk14	122	1.293724814	1.157542776	-0.136182038	0.70827422	0.842115525
Fank1	24	1.39882473	1.298337727	-0.100487002	0.70828607	0.842115525
Golga4	38	1.622078797	1.398479468	-0.223599329	0.70867066	0.842504523
Ccm2	57	1.749367821	1.593947655	-0.155420167	0.7088351	0.842631748
Asb17	1	97.01492537	96.36363636	-0.651289009	0.70907668	0.842680116
ɔ31417E11F	4	92.64705882	91.7677144	-0.879344422	0.7091055	0.842680116
Lamtor4	18	1.840030836	1.495708236	-0.3443226	0.70910455	0.842680116
Ndufc1	43	1.563077064	1.487194753	-0.075882311	0.70898732	0.842680116
Slc37a4	10	3.452469657	2.185142705	-1.267326953	0.70955053	0.843140697
Gigyf1	2	96.52406417	95.87589633	-0.648167845	0.70976532	0.843327636
Pkig	46	1.285734366	0.94843984	-0.337294527	0.70990615	0.843358395
Rps12	25	1.339108956	1.069712606	-0.26939635	0.70987953	0.843358395
Rora	172	7.471078758	7.703446387	0.232367629	0.71033107	0.843672914
Rsph3a	2	2.85398611	4.057591623	1.203605513	0.71025344	0.843672914
Chpf	69	1.308902061	1.184925012	-0.123977049	0.71034339	0.843672914
Rnmtl1	30	0.920772334	0.717637926	-0.203134408	0.7107667	0.844107367
;10507B11F	77	1.114204687	1.026913343	-0.087291344	0.71083358	0.844118472
Stx7	8	1.819125765	1.393954422	-0.425171343	0.71091693	0.844149135
Tmed4	15	1.031206389	1.001929732	-0.029276657	0.71098751	0.844164636
Ran	131	1.064778006	1.006188262	-0.058589744	0.71141634	0.844317768
Fbxl6	35	1.581929252	1.061043977	-0.520885276	0.71124492	0.844317768
Mir8116	6	2.653730542	2.395370455	-0.258360087	0.71128703	0.844317768
Pomgnt1	45	1.084893164	1.178464362	0.093571198	0.71146172	0.844317768
Unc50	17	0.704991985	0.743527385	0.0385354	0.71146084	0.844317768
Psip1	48	1.347571811	1.323832926	-0.023738885	0.71130633	0.844317768
Numa1	24	0.734310628	0.626857493	-0.107453134	0.71170799	0.844496582
Slc4a7	91	1.007449209	0.904494385	-0.102954824	0.71172749	0.844496582
Phf20l1	80	1.147064368	1.162020316	0.014955948	0.71217163	0.844955246
M6pr	14	1.398893764	1.739335473	0.340441708	0.71223311	0.844959872

Prpf4	21	1.602975991	1.23657687	-0.366399122	0.71247092	0.84507314
Wdr13	5	22.9115821	23.71099037	0.799408269	0.71250136	0.84507314
Afg3l1	46	0.787700742	0.854773343	0.067072601	0.71242876	0.84507314
Gkn1	3	96.76712723	96.3521726	-0.41495463	0.71280618	0.845074033
Mki67	41	1.38902894	1.153073705	-0.235955235	0.7130478	0.845074033
Xpc	28	1.073444296	0.917035882	-0.156408415	0.71296531	0.845074033
Usp43	87	1.758007458	1.56702036	-0.190987098	0.71347487	0.845074033
)30507D05F	24	1.291888586	1.264472024	-0.027416562	0.71348114	0.845074033
Ccdc6	110	1.355470875	1.321160125	-0.03431075	0.71270368	0.845074033
Pdpk1	47	1.601525392	1.42059413	-0.180931263	0.71323851	0.845074033
Rcan3	68	1.205105294	1.029232222	-0.175873072	0.71267001	0.845074033
St13	43	1.366759047	1.381371919	0.014612872	0.7126006	0.845074033
190007I07R	3	2.498161694	1.995652899	-0.502508794	0.7128537	0.845074033
Engase	22	2.366299187	2.307717521	-0.058581666	0.71344724	0.845074033
Hpse	13	3.874990661	4.712262606	0.837271944	0.71339691	0.845074033
Platr26	1	13.63636364	15.44715447	1.810790835	0.71325841	0.845074033
Senp8	25	1.568753265	1.434984789	-0.133768476	0.71287139	0.845074033
Bard1	39	1.725914686	1.74222569	0.016311005	0.71303309	0.845074033
Itgb1	79	0.917561875	0.952016839	0.034454964	0.71320203	0.845074033
Rabgap1l	56	5.033881214	5.089484265	0.055603051	0.71312503	0.845074033
G3bp2	52	0.758103498	0.944466726	0.186363228	0.71382958	0.845418507
Trim41	24	1.651771237	1.825920971	0.174149734	0.71393936	0.845480285
Entpd5	32	2.824884154	3.23902164	0.414137486	0.71419852	0.845718935
Gen1	22	1.492989876	1.402351809	-0.090638067	0.71438482	0.845871288
Pcnxl4	28	1.648143367	1.304608831	-0.343534536	0.71453445	0.845960684
Dnlz	30	1.807770282	1.472196857	-0.335573425	0.71457562	0.845960684
.30046K22F	30	1.055408826	1.00096251	-0.054446315	0.71497818	0.846368976
430035B10F	34	0.904965973	0.90872804	0.003762067	0.71509046	0.846433602
Slc39a10	126	1.299872619	1.133428076	-0.166444543	0.7153405	0.846611474
Pex12	42	1.50286125	0.966913419	-0.535947831	0.71541381	0.846611474
Fbxl12os	21	1.541465835	1.262875037	-0.278590799	0.71536342	0.846611474
N4bp2l2	27	1.230135383	1.113307037	-0.116828346	0.71551737	0.846665746
210408I21R	19	0.915771583	0.979700092	0.063928509	0.71563319	0.846734513
Rab2a	49	1.249743627	1.373599776	0.12385615	0.71657002	0.847774609

Oxsm	22	1.451307967	1.428640256	-0.022667712	0.71663223	0.847779859
C2cd2l	55	1.637696646	1.561596258	-0.076100387	0.71677233	0.8478089
Omp	8	95.8344925	96.49995467	0.665462172	0.71673071	0.8478089
Ect2	3	1.970719322	2.53317938	0.562460057	0.71693481	0.847932728
Zfp131	58	1.57115956	1.593407708	0.022248148	0.71703422	0.847945008
230114K14R	5	0.942612943	0.625	-0.317612943	0.71706076	0.847945008
430042O09R	34	1.052086531	1.102951566	0.050865035	0.71718321	0.848009029
Dhx16	46	1.310774025	1.306308343	-0.004465682	0.71723048	0.848009029
Fgf21	1	93.75	95.45454545	1.704545455	0.71754611	0.848313858
Mydgf	20	0.530436992	0.884143199	0.353706208	0.71774721	0.848483251
Ankrd13a	70	1.284582335	1.041298529	-0.243283806	0.71814178	0.848742055
Yap1	43	1.802963914	1.844247808	0.041283893	0.71818388	0.848742055
Zc3h10	45	1.157378992	1.185071005	0.027692013	0.71825534	0.848742055
Tmx1	10	1.527630494	1.242315039	-0.285315455	0.71824941	0.848742055
Cd47	89	1.403322777	1.382184433	-0.021138345	0.71803068	0.848742055
Cyba	19	1.192492939	1.442820199	0.25032726	0.71887222	0.849402608
Man2a1	131	1.428324307	1.326657199	-0.101667108	0.71906277	0.849559349
Kti12	41	1.473729629	1.251494639	-0.222234989	0.71917427	0.849622677
Rhpn1	16	4.109626015	3.850704449	-0.258921567	0.71940117	0.84982231
Hk2	22	2.347826091	2.508341629	0.160515538	0.71950395	0.849838718
Mcm9	25	1.181398027	1.158015341	-0.023382686	0.71953089	0.849838718
Grsf1	123	1.036695545	1.021848475	-0.01484707	0.71962612	0.849882789
Helz	62	1.507654226	1.677744545	0.170090319	0.72003341	0.850245988
Pspc1	12	1.631383347	1.538990588	-0.092392759	0.72004954	0.850245988
Cdc7	53	1.175311966	1.093523749	-0.081788217	0.72020155	0.850357066
Kctd3	38	1.251482324	1.210223455	-0.04125887	0.72041078	0.85053567
Stt3b	70	1.080753147	0.962068868	-0.118684279	0.72059537	0.850616718
Ppil1	21	1.153891344	1.737653076	0.583761732	0.72053879	0.850616718
Alg1	5	0.24691358	0.695652174	0.448738594	0.72146323	0.850693898
Pigs	30	2.113573612	1.789280486	-0.324293126	0.72127913	0.850693898
Nek7	101	1.366335457	1.330550366	-0.035785091	0.72157735	0.850693898
Akap10	47	1.950481346	2.025670533	0.075189187	0.72129317	0.850693898
Ctdsp1	74	2.440580082	2.284375802	-0.15620428	0.7217031	0.850693898
Mir1a-1	1	58.49056604	60.8	2.309433962	0.72141011	0.850693898

Hnrnpa3	31	0.575803933	0.554772039	-0.021031894	0.72145387	0.850693898
Wdr60	31	1.182525834	1.029175261	-0.153350573	0.72088816	0.850693898
Smarcb1	46	0.96022873	1.042902822	0.082674092	0.72152485	0.850693898
Moap1	29	0.839071532	1.461397247	0.622325715	0.72170426	0.850693898
310001G20F	26	1.229505898	1.177165065	-0.052340833	0.72086448	0.850693898
Zfp651	62	1.494657947	1.774781488	0.280123541	0.72135881	0.850693898
Vps18	11	0.960893561	1.036531257	0.075637696	0.72113769	0.850693898
Neurl1b	57	1.278864051	1.283764604	0.004900553	0.72098299	0.850693898
Adnp	25	1.288906907	1.540344232	0.251437325	0.72112568	0.850693898
Ngb	13	17.77516125	21.28294225	3.507781001	0.72144274	0.850693898
Poglut1	43	1.024844622	1.13283833	0.107993707	0.72165268	0.850693898
Fastkd3	29	1.142960204	1.268442482	0.125482278	0.7213634	0.850693898
Cetn3	42	1.167459733	1.197360706	0.029900973	0.72193184	0.850893806
Rreb1	148	1.479175728	1.425246427	-0.053929301	0.722211	0.851017766
Zfp804a	88	5.752363083	6.418355864	0.665992782	0.72210064	0.851017766
Diras2	14	6.332658838	7.002903491	0.670244653	0.72220297	0.851017766
Nsun6	29	1.657651464	1.567665229	-0.089986235	0.722376	0.851143846
Islr2	7	1.471819643	1.974726629	0.502906986	0.72253045	0.851257471
500014C10F	20	23.96867884	23.82862807	-0.140050775	0.72261399	0.851287545
Dnajc25	54	0.960164261	0.835299145	-0.124865117	0.72268789	0.851306261
Krt42	10	89.11685956	88.19849886	-0.918360692	0.72274899	0.851309894
Yars	23	1.250506499	1.327621511	0.077115012	0.72288747	0.851404669
Ptpn14	96	2.287518853	1.95162903	-0.335889823	0.72300865	0.851479048
Pdgfd	3	29.55742297	31.62778023	2.070357265	0.7231944	0.851629459
Tctn1	18	1.215601125	1.20267274	-0.012928385	0.72344413	0.851669838
G2e3	37	1.278607017	1.134346709	-0.144260308	0.72341136	0.851669838
Cyb5r3	32	3.469334969	3.486976676	0.017641707	0.72334931	0.851669838
410089E03F	20	1.095604811	1.189912174	0.094307363	0.72346085	0.851669838
Gins3	34	1.036737907	1.156289174	0.119551268	0.72374666	0.851732981
Zyx	93	1.629070034	1.482894265	-0.14617577	0.72374247	0.851732981
Ccne1	108	2.360924165	2.368105681	0.007181515	0.72363437	0.851732981
Izumo4	49	1.734988976	1.631894186	-0.10309479	0.72372764	0.851732981
Fxr1	140	1.264310028	1.197515076	-0.066794952	0.72389846	0.851756086
033403F01F	23	1.728986251	1.171669995	-0.557316255	0.72394043	0.851756086

Dhrs13	63	0.907433267	0.90817176	0.000738493	0.72388057	0.851756086
Setd2	104	1.386354203	1.337886653	-0.048467549	0.72405537	0.851815932
Coasy	23	1.497201212	1.250557084	-0.246644128	0.7241074	0.851815932
Efcab2	51	1.267344532	1.073860048	-0.193484483	0.72418569	0.851839746
Polg2	42	1.416988111	1.238152838	-0.178835273	0.72437665	0.85192872
Gzmn	1	83.33333333	80.43478261	-2.898550725	0.72437745	0.85192872
Paxip1	120	1.078645789	1.142308326	0.063662537	0.7244763	0.851976697
Pld2	44	1.602019016	1.382429921	-0.219589095	0.72456845	0.852016783
Mir7654	87	1.088238035	0.887040605	-0.20119743	0.7246611	0.85205745
Ssu72	31	1.18863483	0.940212909	-0.248421921	0.7247617	0.852107459
Thada	39	1.296650149	1.471905752	0.175255603	0.72510722	0.852377102
'00097O09F	6	0.649350649	0.701754386	0.052403737	0.72508669	0.852377102
Phldb2	31	1.533251184	1.857447164	0.32419598	0.72535518	0.852600282
Gnptab	90	1.209989059	1.113904555	-0.096084504	0.72605368	0.853284612
Scap	75	1.290664586	1.298039392	0.007374806	0.7260529	0.853284612
Rab35	103	1.249719416	1.198451486	-0.051267931	0.72620895	0.853398747
Gm13363	38	0.946931598	0.825748447	-0.121183151	0.72630301	0.853440929
Qrsl1	14	1.356122427	1.665529255	0.309406828	0.72646582	0.853508116
Senp7	22	1.02332244	1.113452166	0.090129725	0.72647651	0.853508116
Jpx	29	0.84154152	0.982968171	0.14142665	0.72691778	0.853958175
Rasgef1b	45	1.109403725	1.256751775	0.14734805	0.72717197	0.854188399
)30563E18F	19	2.01925668	1.981747592	-0.037509088	0.72736982	0.854296507
Mob3a	43	1.287145666	1.213259141	-0.073886524	0.72738044	0.854296507
Kansl2	78	1.082286476	1.076148143	-0.006138332	0.727615	0.854503607
Uqcrq	5	1.630604266	1.927722036	0.297117771	0.72792144	0.854795075
330418K02F	18	1.340114254	1.307149541	-0.032964713	0.72806905	0.854899998
Usp42	130	1.351341904	1.274820206	-0.076521698	0.72817582	0.854920186
Kif3a	34	1.991331108	1.766552179	-0.224778929	0.72820277	0.854920186
Pdrg1	16	1.734301084	1.301045832	-0.433255252	0.72862102	0.85503649
Slc25a16	27	1.134660206	1.221332756	0.086672549	0.72864195	0.85503649
Haus1	31	1.2236507	1.388554539	0.164903839	0.72850767	0.85503649
Slc29a1	44	2.064053745	1.904750809	-0.159302936	0.72843371	0.85503649
Cnbp	29	0.459986377	0.652707419	0.192721041	0.72865145	0.85503649
Rlim	18	18.66601221	19.17321235	0.507200145	0.72841544	0.85503649

Mir7119	5	93.80981553	92.09557393	-1.714241599	0.7288872	0.855194805
Med7	25	0.893255916	1.013095305	0.119839389	0.7289612	0.855194805
Rpl21	29	13.55195424	12.30278415	-1.249170083	0.72892118	0.855194805
310474O19F	31	1.036346129	1.155160758	0.118814629	0.72929557	0.855517308
Maff	81	1.630489388	1.670078879	0.03958949	0.7293527	0.855517308
Ttk	23	1.487467028	1.275569609	-0.211897419	0.72948898	0.855540384
Stam2	39	1.732893015	1.445396039	-0.287496976	0.72947397	0.855540384
Mnt	48	1.571404222	1.431708171	-0.139696051	0.72957932	0.855577956
Hyal1	1	8.391608392	7.258064516	-1.133543875	0.73096963	0.856301305
Gle1	15	0.49738221	0.798386427	0.301004216	0.73088463	0.856301305
Fndc3a	126	1.09829599	1.059550917	-0.038745072	0.73112983	0.856301305
Rpl14	53	1.654511503	1.718658593	0.06414709	0.73068777	0.856301305
Tmem39a	38	1.469899231	1.421368983	-0.048530249	0.73058651	0.856301305
Srsf4	108	1.289023478	1.251631988	-0.03739149	0.73106269	0.856301305
Cav2	27	2.350140136	2.315815237	-0.034324899	0.73034831	0.856301305
Mir7077	7	90.61522531	86.69782297	-3.917402341	0.73083174	0.856301305
Ppan	32	1.256821477	1.215370303	-0.041451174	0.73090691	0.856301305
Tff1	1	93.5483871	92.38095238	-1.167434716	0.73027681	0.856301305
Slc38a2	54	1.190999245	1.153391823	-0.037607421	0.73091075	0.856301305
Car14	2	4.985431235	5.214044371	0.228613135	0.73108555	0.856301305
Fam96a	2	1.004016064	0.727272727	-0.276743337	0.73071198	0.856301305
Gnb2	6	1.54806981	1.560580177	0.012510367	0.73075217	0.856301305
Atg12	15	1.587538536	1.238142227	-0.349396309	0.73111329	0.856301305
Ern1	75	1.131302873	1.190300152	0.058997279	0.73085857	0.856301305
Krt26	5	91.17691711	92.32759129	1.15067418	0.73139729	0.856546199
Isoc1	49	1.628296449	1.376297007	-0.251999441	0.73158744	0.856658517
Cdc37l1	52	1.423161345	1.308160834	-0.115000511	0.73160996	0.856658517
Oas1b	9	3.565975761	3.387300236	-0.178675525	0.73197615	0.856780255
Slc32a1	10	21.22146197	21.50669943	0.285237453	0.73202879	0.856780255
Mon1a	16	1.838287959	1.591084709	-0.24720325	0.73200613	0.856780255
Ndufv2	67	0.949624922	0.95757377	0.007948848	0.73198058	0.856780255
Apoa2	3	32.42424242	31.3363772	-1.087865227	0.73206425	0.856780255
Cep83os	35	1.12552067	1.106957975	-0.018562695	0.73183959	0.856780255
Spryd7	34	2.544575618	3.594252304	1.049676686	0.73216106	0.856800419

Gmeb2	23	1.510085618	1.658078873	0.147993256	0.73219826	0.856800419
Bloc1s1	19	1.218117174	1.235627206	0.017510032	0.7324368	0.856966268
Bri3	66	1.417552008	1.402022315	-0.015529693	0.73245679	0.856966268
Tspyl4	5	0.19047619	0.434782609	0.244306418	0.73266158	0.857000876
Snx3	66	1.038246707	0.909751128	-0.128495579	0.73258521	0.857000876
Strip2	77	4.970492688	4.786681852	-0.183810835	0.73264127	0.857000876
Pask	47	1.549770926	1.459238245	-0.090532681	0.73285819	0.857094209
15-Sep	35	1.118043619	1.090554729	-0.02748889	0.73285483	0.857094209
Kctd9	68	1.334932629	1.318022151	-0.016910478	0.73294836	0.857131352
Nmral1	6	4.14608569	3.96700383	-0.17908186	0.73317743	0.857327209
Ptgfrn	80	1.580769497	1.369153527	-0.21161597	0.73323269	0.857327209
Shkbp1	13	1.986990463	1.865659142	-0.121331321	0.73353409	0.857554952
Vdac2	55	1.724311272	1.317763624	-0.406547648	0.73354435	0.857554952
Rab1b	9	8.802160262	8.218905713	-0.583254549	0.7337295	0.857703076
)30546C10F	4	89.92537313	89.23509414	-0.690278992	0.73403559	0.857775353
Lppos	60	1.958897101	1.919004258	-0.039892843	0.73408361	0.857775353
Rmnd1	26	1.535224436	1.569832249	0.034607813	0.73398846	0.857775353
Mir8111	9	2.025145444	2.391886171	0.366740727	0.73407124	0.857775353
Obox6	1	92.08633094	90.90909091	-1.177240026	0.73397383	0.857775353
Dek	105	1.366949942	1.279665669	-0.087284273	0.73414534	0.857779177
Fitm2	4	1.058791508	1.19047619	0.131684683	0.73438327	0.857988859
Arhgef1	54	1.862570642	1.574221201	-0.288349441	0.73458588	0.858157254
Stxbp3	36	1.503258965	1.674243623	0.170984659	0.73492521	0.858181611
Hsdl1	56	1.169171634	1.293754514	0.12458288	0.73495763	0.858181611
Psd3	4	84.8407567	85.21535511	0.374598404	0.73467025	0.858181611
)30040B21F	20	1.897175489	1.320340602	-0.576834887	0.73493678	0.858181611
)30558J18R	20	1.235698082	0.999098193	-0.236599889	0.73491677	0.858181611
Tsen2	12	1.214847814	1.058841471	-0.156006343	0.734865	0.858181611
Trpt1	20	1.291354442	1.37867053	0.087316088	0.73513907	0.858325162
Morc2a	54	1.211653347	1.350455226	0.138801878	0.73600288	0.859265353
Cmpk1	98	1.205269206	1.059773635	-0.145495571	0.73611478	0.85931038
Vps13b	78	1.341717934	1.237485526	-0.104232409	0.73615857	0.85931038
Fam207a	26	2.457996241	2.214345045	-0.243651196	0.73628015	0.859383943
Rapgef6	40	1.4108996	1.411514072	0.000614472	0.73658266	0.859531926

Wdr46	48	1.516456195	1.534201067	0.017744872	0.73654888	0.859531926
Pigo	12	1.49425863	1.568597568	0.074338938	0.73656351	0.859531926
Gal3st4	8	25.9172124	26.75879254	0.841580131	0.73682979	0.859751936
Ralgps1	57	1.460991529	1.473374532	0.012383003	0.736919	0.859787663
Fam134c	49	1.494645614	1.494363674	-0.00028194	0.73700813	0.85982329
Pef1	11	1.073780021	1.366321486	0.292541465	0.73709433	0.859855492
Tigd2	51	1.476859585	1.267214044	-0.209645541	0.73732141	0.859983654
Hacd3	69	1.385063091	1.191747733	-0.193315358	0.73727447	0.859983654
Golt1b	57	1.418602865	1.166116491	-0.252486375	0.73742353	0.860023394
Dync1li2	29	1.532834813	1.511655557	-0.021179255	0.7374727	0.860023394
Arl2bp	51	1.336429955	1.315928323	-0.020501633	0.73773513	0.86026107
Sfrp5	16	22.29829881	24.21101083	1.912712028	0.73794434	0.860436647
Smc5	53	0.834114587	1.023555171	0.189440584	0.73846491	0.860770046
Amdhd1	29	2.052902182	2.438589761	0.385687579	0.73841591	0.860770046
Pdhb	25	1.396067567	1.265182533	-0.130885034	0.73842936	0.860770046
Magi3	76	1.067831745	0.984021208	-0.083810536	0.73834303	0.860770046
Blzf1	10	1.798621932	1.662332941	-0.136288991	0.7386427	0.860908889
Rbbp5	11	1.158503495	1.366014226	0.207510731	0.73873787	0.860951432
Zfp318	99	1.056303184	0.971892959	-0.084410225	0.73888835	0.86099005
Tob1	91	1.377644107	1.374191199	-0.003452908	0.7388635	0.86099005
Mir423	33	1.622584199	1.610647502	-0.011936697	0.73905645	0.861012337
Ash2l	63	1.584005639	1.479556821	-0.104448818	0.73908351	0.861012337
Kif18a	43	1.540424837	1.454373783	-0.086051055	0.73907293	0.861012337
Ubr3	93	1.195744569	1.171978073	-0.023766496	0.73924411	0.861117823
Csf3r	3	26.13399085	26.40804514	0.27405429	0.73938903	0.861117823
Cebpz	25	1.206592485	0.889969447	-0.316623038	0.73931965	0.861117823
Tubb2a	28	1.376690978	1.423916342	0.047225364	0.73940879	0.861117823
Fam216a	38	1.276861085	1.191300779	-0.085560306	0.73950629	0.861163032
Tnfrsf13c	7	5.164142263	5.529142256	0.364999994	0.7396882	0.861306511
Ssbp1	21	1.583775922	1.335936563	-0.247839359	0.74003441	0.86150456
Mthfd2	38	1.233241019	1.331494084	0.098253065	0.74003286	0.86150456
Gm10433	54	2.067265938	1.965775668	-0.101490271	0.73997044	0.86150456
Jarid2	110	1.20435271	1.231875621	0.027522911	0.74013716	0.86155582
Ikbke	1	37.95620438	40	2.04379562	0.74033094	0.861713029

Ticam1	6	2.797582089	2.173893513	-0.623688576	0.74042776	0.861757371
Mettl23	11	0.840292416	0.952830099	0.112537682	0.74077274	0.862090511
Ogg1	53	1.694272191	1.664423357	-0.029848834	0.74093284	0.862208449
Cdca2	50	0.982577209	1.241592453	0.259015244	0.74187853	0.862940904
Gtf2a2	50	0.992577457	0.962721827	-0.02985563	0.74187325	0.862940904
Ikbip	29	1.010121582	1.07755399	0.067432408	0.74179078	0.862940904
Lrtm2	20	51.39388472	50.68359186	-0.710292866	0.74197393	0.862940904
Prpf31	9	1.206262375	1.874902657	0.668640282	0.74192113	0.862940904
Fam222b	12	1.366582645	1.645331931	0.278749285	0.741962	0.862940904
Arid5b	5	1.209859792	0.900143366	-0.309716426	0.74182042	0.862940904
Sytl2	8	11.91538904	11.35005072	-0.565338317	0.74207831	0.862993907
Ces2f	1	92.98245614	91.81818182	-1.164274322	0.7421915	0.863057143
430049B03R	44	17.66593054	16.22615772	-1.439772817	0.74243409	0.863270829
Paics	14	0.56867501	0.589186477	0.020511467	0.74260771	0.863335879
Nsmce4a	70	1.321122478	1.327386654	0.006264176	0.74256703	0.863335879
Alcam	31	1.560423413	1.59002639	0.029602976	0.74274579	0.863428011
Stil	15	0.349912323	0.440130165	0.090217843	0.74287247	0.863506863
Zbtb25	103	0.890703822	0.867825186	-0.022878636	0.7430533	0.863648647
Abcd3	122	1.076912657	1.034122316	-0.042790341	0.74334386	0.863917935
Slc36a1	18	1.289483451	1.255786676	-0.033696776	0.74341642	0.863933838
Slc22a18	6	52.30532752	52.50358977	0.198262253	0.7435045	0.863967775
Fam114a1	26	1.184523717	1.236119	0.051595283	0.74369327	0.864000679
Ushbp1	5	55.48908	54.59154214	-0.897537859	0.74370696	0.864000679
Atp2b1	221	1.247643816	1.184009017	-0.063634799	0.74370946	0.864000679
Pramef8	10	1.094629009	1.459057293	0.364428284	0.7438786	0.864060365
Rc3h1	65	1.070463174	0.956092401	-0.114370773	0.74384472	0.864060365
410127L17R	27	14.16139262	13.23649141	-0.924901213	0.74401168	0.864078152
Dhx36	56	1.586111768	1.475235605	-0.110876163	0.74396077	0.864078152
Nipal1	9	2.191740157	2.180928548	-0.010811609	0.74414132	0.864124963
F2r	28	1.209907931	1.111137535	-0.098770396	0.74416977	0.864124963
Parp6	59	1.184683187	1.157214171	-0.027469016	0.74437557	0.864295548
Zfp560	20	1.171105505	1.338789485	0.16768398	0.74481637	0.864716851
Paxbp1	49	1.120125712	1.162819567	0.042693855	0.74485628	0.864716851
Kazald1	16	3.017557387	3.067114361	0.049556974	0.74525004	0.865037107

Hars	11	1.280590787	0.867144041	-0.413446746	0.74519146	0.865037107
Xrn2	63	1.167180626	1.262456512	0.095275886	0.74540873	0.865084447
Afap1l1	22	5.201220034	5.165588596	-0.035631438	0.74540138	0.865084447
Cept1	7	0.432900433	0.235478807	-0.197421626	0.74547459	0.865092463
Fam214a	70	2.563773696	2.556116241	-0.007657455	0.74558419	0.865151221
Ttc3	39	1.004807407	1.229235932	0.224428525	0.7457383	0.865193214
Ndc1	29	1.498279746	1.369657782	-0.128621964	0.74571426	0.865193214
Vps4a	76	1.562850668	1.248144882	-0.314705786	0.74596615	0.865320734
Ahr	59	0.894526736	0.904370842	0.009844107	0.74593802	0.865320734
Tbc1d10b	121	1.394790028	1.339900812	-0.054889216	0.74615788	0.86547472
Smc6	59	1.393334446	1.375071112	-0.018263334	0.74631858	0.865524287
Ezh2	61	1.235456075	1.142133405	-0.09332267	0.74630783	0.865524287
Tfip11	49	1.234753383	1.223439047	-0.011314337	0.74666335	0.865650469
Nvl	16	1.083218726	1.535181069	0.451962343	0.74651682	0.865650469
Rps27l	57	1.314800587	1.2431694	-0.071631186	0.74657332	0.865650469
Gpatch11	17	0.712673435	0.603091023	-0.109582412	0.74662348	0.865650469
Oxsr1	98	1.006969561	1.070017126	0.063047566	0.74688235	0.865699184
Alx1	19	44.52043507	44.2086274	-0.311807673	0.74680142	0.865699184
Emc2	45	1.575170673	1.415879362	-0.159291311	0.74686258	0.865699184
Zfp935	3	1.22574692	2.43902439	1.21327747	0.74729294	0.866062867
Zc3h6	65	1.29857208	1.091535554	-0.207036526	0.74731416	0.866062867
Nol7	43	3.338984566	3.247383036	-0.091601531	0.74743915	0.86613931
Clta	32	0.86992803	0.900086639	0.03015861	0.74768135	0.866283154
Rarres1	43	17.96243147	18.87029618	0.907864713	0.74764207	0.866283154
Acad10	18	1.128220798	1.313647629	0.185426831	0.74776805	0.866315206
Gsk3a	58	1.242626552	1.192561547	-0.050065005	0.74812131	0.866656039
Kctd16	3	73.74345848	72.77866213	-0.96479635	0.74825782	0.866745758
Trmt1	18	1.917183622	1.41305806	-0.504125562	0.74861298	0.866981246
Phf12	79	1.519223468	1.605310308	0.08608684	0.74853656	0.866981246
Rnpc3	45	0.901559832	0.989814133	0.088254301	0.74863837	0.866981246
Wdr45b	47	1.193815878	1.220828309	0.027012431	0.74883437	0.867074065
Myo1b	71	1.164497462	1.123042129	-0.041455332	0.74883669	0.867074065
Btbd2	3	9.372043011	9.343773304	-0.028269707	0.74899693	0.86717072
Mitf	22	1.932098633	2.147557133	0.2154585	0.74903836	0.86717072

Zfand4	74	1.940722173	1.473327993	-0.46739418	0.74928092	0.867383098
Mrps28	33	1.437459603	1.299810488	-0.137649115	0.74936747	0.867414863
Ap5s1	13	0.75926651	0.626218184	-0.133048326	0.74951212	0.86744545
Pnkp	15	2.222594365	2.165636043	-0.056958322	0.74947669	0.86744545
Smurf1	90	1.024149048	1.024239181	9.01E-05	0.74987326	0.867794967
Hint2	23	2.326248123	1.960254232	-0.365993891	0.75005476	0.867868127
Chn2	78	2.587675999	2.541242089	-0.04643391	0.75003734	0.867868127
230056P14F	6	1.782614087	1.039237881	-0.743376206	0.7502109	0.867980351
Kctd18	55	1.738489696	1.231782495	-0.506707201	0.75033608	0.868056739
Nt5e	28	2.643040751	2.85629739	0.213256638	0.7503953	0.868056812
Khdrbs1	83	0.840841715	0.997498644	0.156656929	0.75059017	0.868190475
Hadhb	2	1.951219512	1.573426573	-0.377792939	0.75062918	0.868190475
Cspp1	8	1.774361707	1.849035583	0.074673876	0.75100325	0.868349357
Ankrd9	121	0.984187893	1.130667983	0.146480089	0.75091576	0.868349357
Fam53c	4	0.871718895	1	0.128281105	0.75098357	0.868349357
Hspb11	35	1.477308	1.554433537	0.077125537	0.75098093	0.868349357
Rbm6	31	1.376481448	1.611512225	0.235030777	0.75107469	0.868363538
Zeb2os	7	10.3880119	9.920634921	-0.467376976	0.75151567	0.868804927
Rps7	41	2.383419779	2.447707303	0.064287523	0.7517524	0.868914811
500012F01F	80	1.427578824	1.371632539	-0.055946285	0.75184758	0.868914811
Cul1	161	1.182016993	1.099399817	-0.082617176	0.75182184	0.868914811
Arv1	28	1.483165099	1.328917231	-0.154247868	0.75181602	0.868914811
Fam117a	39	2.382740054	2.019131329	-0.363608725	0.75200337	0.869026416
Ppig	52	1.208527876	1.245636035	0.037108158	0.75221607	0.86913532
Mrpl46	16	1.514308921	1.564640128	0.050331207	0.75215709	0.86913532
Nubp1	22	1.143983855	1.374585058	0.230601203	0.75229316	0.869155953
Cox17	17	1.535534046	1.615426776	0.07989273	0.752702	0.869559847
Cgnl1	26	1.941593447	1.906512012	-0.035081435	0.75282778	0.869636686
Dynlrb2	3	5.97912191	7.096576482	1.117454572	0.75330391	0.870058933
Arl10	36	0.856381808	0.730550368	-0.12583144	0.75331189	0.870058933
Tesk1	13	1.613173203	1.235446864	-0.377726339	0.75379102	0.870302491
)10107G23F	28	1.423061969	1.248941569	-0.1741204	0.75377646	0.870302491
Nr2c2	120	1.171638938	1.198049276	0.026410338	0.75368598	0.870302491
Mettl21a	32	1.205075513	1.317768518	0.112693005	0.75378354	0.870302491

Dzip3	2	2.318005262	2.683217952	0.365212689	0.75381932	0.870302491
Yaf2	154	1.013614326	1.00790102	-0.005713306	0.75407321	0.870321719
Nme2	45	1.214860742	1.305314948	0.090454206	0.75397855	0.870321719
Nmnat1	35	1.416177794	1.259324751	-0.156853043	0.75402515	0.870321719
.30307A14F	47	1.400757047	1.391218657	-0.009538389	0.75390402	0.870321719
Sec31a	51	1.130922913	1.0552773	-0.075645612	0.75414909	0.870340843
Tada2b	113	1.206778811	1.144644652	-0.062134158	0.75427018	0.870412125
Ublcp1	45	1.576315568	1.3615698	-0.214745768	0.75459061	0.870713421
Eif4h	80	1.001185447	1.077431921	0.076246474	0.7549573	0.871068044
Nfatc3	71	1.187425713	1.050510786	-0.136914927	0.75502268	0.871074989
Syt4	2	22.44682036	23.1298753	0.683054938	0.75518653	0.871195524
Fbxo32	39	1.535984771	1.858580938	0.322596167	0.75542147	0.871279301
Nutf2-ps1	35	1.180131424	1.156797009	-0.023334416	0.75536165	0.871279301
Arl16	15	1.347334679	1.145225575	-0.202109105	0.75543727	0.871279301
Gnl1	53	1.30997952	1.181563019	-0.128416501	0.75561289	0.871413359
Il3ra	26	1.85232389	1.692163773	-0.160160116	0.75581915	0.87158273
Ext1	95	1.016455255	0.98641367	-0.030041585	0.75601089	0.871715134
30020D12F	6	61.80385898	62.12454789	0.320688909	0.75605278	0.871715134
Mbd6	18	1.092582864	1.275056716	0.182473851	0.7561872	0.871801618
Hist4h4	13	0.359480977	0.372036432	0.012555455	0.75637133	0.871834375
Rin3	57	1.454100601	1.380129363	-0.073971238	0.75644742	0.871834375
Cerkl	30	35.64458705	36.4880682	0.843481148	0.75644044	0.871834375
Rps3a1	29	0.800270264	0.929603645	0.12933338	0.75645327	0.871834375
Cinp	7	2.242905629	1.625545271	-0.617360359	0.75672633	0.87187517
Strip1	26	1.505022876	1.434113107	-0.070909769	0.75668115	0.87187517
Yipf4	39	1.521020972	1.087757484	-0.433263488	0.75671619	0.87187517
Denr	76	1.517689655	1.359526795	-0.158162861	0.75665577	0.87187517
Zfc3h1	60	1.331390643	1.199892835	-0.131497807	0.75680731	0.871900019
Pcdhga3	10	69.41033895	68.07462965	-1.335709299	0.75696981	0.872018758
Psmd7	49	1.190313034	1.274736638	0.084423604	0.75719471	0.872209371
Dhx9	38	0.845967642	0.713170721	-0.13279692	0.75775258	0.872646473
Ypel2	48	1.204163715	0.940269904	-0.263893812	0.75771556	0.872646473
Gm1661	7	0.71942446	0.6814562	-0.03796826	0.75765401	0.872646473
Mrfap1	27	0.462789411	0.412173886	-0.050615525	0.75795046	0.87280586

930015E06F	103	1.24973765	1.120201927	-0.129535723	0.7585788	0.873198848
Mir1936	7	77.55386264	77.82978545	0.27592281	0.75843165	0.873198848
Reps1	106	1.345321979	1.394709741	0.049387762	0.75854436	0.873198848
Slc25a46	65	0.986764581	1.002957621	0.01619304	0.75868065	0.873198848
Fez2	57	1.372497634	1.479371351	0.106873717	0.75859002	0.873198848
Tmem258	7	14.02962545	14.21078606	0.181160614	0.75870828	0.873198848
Nin	130	1.896168332	1.881383552	-0.014784781	0.75851093	0.873198848
Ppcdc	14	1.346688539	1.360954278	0.014265739	0.75903885	0.873510786
Gnl2	42	1.038816024	0.906322993	-0.132493031	0.75926654	0.873602291
Zfp1	14	0.871632908	0.775323653	-0.096309255	0.75929696	0.873602291
Casp3	24	1.974869498	1.673548667	-0.301320831	0.75923725	0.873602291
Xpo1	36	1.438193983	1.45123317	0.013039187	0.75986975	0.874192767
Fcho2	59	1.320580836	1.187028658	-0.133552179	0.75999664	0.874201671
Vezt	9	1.04912138	1.4193745	0.370253121	0.75994317	0.874201671
Nckipsd	46	1.148641611	0.904249397	-0.244392213	0.76026293	0.874439433
Mon2	70	1.331157114	1.26369177	-0.067465345	0.76042263	0.874554565
Zfp637	53	1.40716248	1.252822046	-0.154340433	0.76052474	0.874603445
Akr7a5	37	1.361279714	1.393647663	0.032367949	0.76088871	0.874751315
Aak1	51	1.394447544	1.30939695	-0.085050594	0.76071351	0.874751315
Baz2b	31	1.59902047	1.127850251	-0.471170218	0.76089112	0.874751315
Fyco1	29	1.258716346	1.14175951	-0.116956835	0.76089177	0.874751315
Ankrd52	99	0.971273704	0.867297346	-0.103976358	0.76098443	0.874789308
Cntd1	2	1.694915254	2.201257862	0.506342607	0.76109781	0.874791869
Ccdc171	63	0.812031352	0.89780777	0.085776418	0.76110589	0.874791869
'00065D16F	67	0.918482986	0.857910336	-0.06057265	0.76196849	0.875714731
Vamp4	19	1.410144294	1.61053636	0.200392066	0.7622924	0.87601838
P3h1	45	1.054654026	1.119303719	0.064649693	0.76273848	0.876137023
Cast	15	1.488845772	1.137871144	-0.350974628	0.76250939	0.876137023
Calm1	17	1.253424215	1.327025627	0.073601412	0.76266857	0.876137023
Neo1	175	1.317089747	1.217831222	-0.099258524	0.76267768	0.876137023
Herc2	35	1.514194404	1.306408994	-0.20778541	0.76271833	0.876137023
Mtx2	32	0.805031501	0.82511623	0.020084729	0.76275388	0.876137023
Nus1	61	1.354528513	1.240629556	-0.113898957	0.76296067	0.876305956
Utp15	26	1.014499115	0.910295721	-0.104203394	0.76305331	0.876343763

Trib3	8	1.489386143	1.278167641	-0.211218502	0.7631616	0.876399538
Rps13	14	1.264750455	1.168693786	-0.096056668	0.76330205	0.876492233
Ccnk	33	1.056878581	0.949805707	-0.107072875	0.76350085	0.876651912
Nomo1	42	0.949084496	0.812343508	-0.136740988	0.76368153	0.876790766
Ino80dos	16	0.810921643	0.677620772	-0.133300871	0.76399636	0.876980733
Tas1r1	11	0.981328613	0.731905628	-0.249422985	0.76398208	0.876980733
Snx13	85	1.123272338	1.149341581	0.026069243	0.76402628	0.876980733
Ezh1	39	1.475058308	1.599972371	0.124914063	0.76444334	0.877390819
Cox15	29	1.230830857	1.084696796	-0.146134062	0.76476048	0.877548891
7-Sep	68	1.409197714	1.304883171	-0.104314543	0.76471492	0.877548891
Uqcrc2	25	1.331107908	1.510972859	0.179864952	0.7647422	0.877548891
Apc2	3	66.65212333	69.42719117	2.775067839	0.76513614	0.87791131
Pitpna	56	1.425890576	1.442433704	0.016543127	0.76537148	0.878112674
Prkdc	30	1.439159714	1.467974417	0.028814702	0.76548377	0.878172838
Mpi	5	1.09409716	1.344028071	0.249930911	0.76572944	0.87818316
Rad54b	35	0.884720056	1.040544896	0.15582484	0.76579164	0.87818316
Rdh13	19	1.374348196	1.525145774	0.150797578	0.76579199	0.87818316
Mpp6	113	1.315147694	1.283480772	-0.031666922	0.7657351	0.87818316
Hsf2bp	7	97.24919198	97.7414025	0.492210516	0.76558968	0.87818316
Map2k1	43	1.182790838	1.056958272	-0.125832565	0.76610124	0.878331864
Acot4	5	8.001769495	8.262186048	0.260416553	0.76608524	0.878331864
Agl	49	1.644113116	1.646184018	0.002070902	0.76606829	0.878331864
Cnppd1	22	0.930414846	0.869178595	-0.061236251	0.76617046	0.878342603
Mad2l2	31	2.78723656	2.710944006	-0.076292555	0.76637044	0.878434611
Eef1e1	37	3.649717181	3.645356011	-0.00436117	0.76632718	0.878434611
Ppp1r12a	106	1.664197023	1.572640003	-0.091557019	0.76645196	0.878459424
Mars2	116	1.216659287	1.187519474	-0.029139813	0.76656565	0.878521113
Papolg	24	1.756471755	1.57307226	-0.183399495	0.76688896	0.878823002
Vwc2l	1	67.33333333	65.67164179	-1.661691542	0.76705342	0.878874199
Samd5	60	1.255756511	1.270901091	0.01514458	0.76700606	0.878874199
Wdr19	13	4.483575081	4.529357615	0.045782534	0.7672494	0.879030114
Bcl7b	45	1.330047432	1.421983474	0.091936042	0.76749181	0.879185537
Ggps1	28	1.936038185	2.093227898	0.157189713	0.76750489	0.879185537
Cmbl	12	27.94150718	27.7251748	-0.216332376	0.76789264	0.879372318

Zc2hc1a	42	0.996635932	0.96020326	-0.036432672	0.76785245	0.879372318
Abhd8	36	1.521129228	1.65386076	0.132731532	0.76780204	0.879372318
Ahctf1	46	1.128769161	1.034470398	-0.094298764	0.76790765	0.879372318
Gm5113	3	0.966183575	0.529100529	-0.437083046	0.76810232	0.879526603
Rad1	12	2.038979398	1.401268176	-0.637711222	0.76817983	0.879546726
Kif13b	27	1.732834825	1.804239861	0.071405036	0.76847789	0.879819351
310009A15R	8	0.608333534	0.75248087	0.144147337	0.76909757	0.880196132
Rab3gap1	3	1.981949674	3.08432702	1.102377346	0.76888159	0.880196132
Abcc5	57	1.537700901	1.548664214	0.010963312	0.76897186	0.880196132
Ficd	53	2.416715222	2.166900794	-0.249814427	0.76908676	0.880196132
Megf8	23	1.573264101	1.381273425	-0.191990676	0.76918018	0.880196132
Zcchc2	92	1.104037121	0.882032389	-0.222004732	0.76918952	0.880196132
Sos2	135	1.227011396	1.228618501	0.001607105	0.76922688	0.880196132
Casp6	25	1.182768465	1.347895884	0.165127419	0.76954273	0.880488886
Snx33	14	1.541041099	1.531306983	-0.009734117	0.76987971	0.880805775
Tnrc6a	27	1.47422927	1.122071131	-0.352158139	0.77053365	0.881485215
Rab22a	36	1.756358594	1.672374734	-0.08398386	0.77081898	0.881685103
Slc25a36	38	1.912418597	1.890833739	-0.021584858	0.77088864	0.881685103
Trio	121	1.303834828	1.096193436	-0.207641392	0.77086777	0.881685103
Tomm6os	30	1.19547166	1.433474271	0.238002611	0.77144886	0.882162985
Ccdc71l	72	1.103869587	1.081031541	-0.022838046	0.7714479	0.882162985
Ier3ip1	33	1.683017026	1.827530599	0.144513573	0.77151	0.882162985
Rpp40	6	0.827895019	0.902977052	0.075082032	0.77154694	0.882162985
Spry4	42	1.137626353	1.405485119	0.267858765	0.77160875	0.882164925
Mir665	1	89.94413408	89.09090909	-0.853224987	0.77204608	0.882531661
Rhbdf2	25	0.931056191	1.121691655	0.190635463	0.77204981	0.882531661
Maged2	1	23.26732673	22.05128205	-1.216044681	0.77231717	0.88267207
Fau	4	0.841135575	0.644494474	-0.196641101	0.77241732	0.88267207
Arl4a	109	1.260076372	1.25168629	-0.008390082	0.77241008	0.88267207
Apbb2	75	1.221624874	1.269107442	0.047482568	0.77223683	0.88267207
'30508B09R	56	0.911855567	1.03940736	0.127551794	0.7724734	0.88267207
Gpr68	18	16.51010166	16.31708206	-0.1930196	0.77260961	0.88275897
Prpsap1	57	0.969806231	0.89566399	-0.074142241	0.77278202	0.882887218
Phtf1os	36	1.324370353	1.044624317	-0.279746036	0.77290461	0.882958529

Ddx39b	58	1.36034186	1.186674839	-0.173667021	0.77323488	0.883267056
Tmem241	24	1.218572073	1.302349407	0.083777334	0.77374272	0.88376266
Wbp1	34	1.884994152	1.750745188	-0.134248964	0.77391694	0.88376266
Eng	19	54.07907598	53.99404192	-0.085034059	0.77386711	0.88376266
Sp1	73	1.28171491	1.318608697	0.036893787	0.77386061	0.88376266
Ahcy	27	0.89485177	0.897805774	0.002954004	0.7740301	0.88376266
Fancg	7	1.207967417	1.357740332	0.149772915	0.77399768	0.88376266
Sco1	28	1.089201804	1.256984193	0.167782389	0.77424744	0.883942032
H2afj	10	0.569050321	0.740402509	0.171352188	0.77438608	0.884031536
Etfa	35	1.081739076	0.900238428	-0.181500648	0.77451909	0.884114593
C1d	25	1.629093526	1.547329385	-0.081764141	0.77460982	0.884149388
Isca2	5	2.31777199	1.770383541	-0.547388449	0.77489156	0.884264615
Mbtd1	25	2.948151774	2.640220704	-0.307931069	0.7748265	0.884264615
10015M20I	12	0.400286141	0.41963089	0.019344749	0.77485432	0.884264615
Agpat4	11	1.531422484	1.879088761	0.347666277	0.77521447	0.884495535
Npc2	18	1.785475616	1.561130513	-0.224345103	0.77516266	0.884495535
Smu1	26	1.559806696	1.366221352	-0.193585344	0.77558488	0.884540662
Lin54	89	1.716430301	1.757101515	0.040671214	0.77558935	0.884540662
Ifitm5	2	92.45336348	92.27642276	-0.176940718	0.77555736	0.884540662
Mtr	31	1.357921513	1.06111853	-0.296802983	0.77546509	0.884540662
Efcab14	26	0.700692806	0.742095223	0.041402417	0.7755664	0.884540662
Pkp2	55	1.70493444	1.593439483	-0.111494956	0.77561569	0.884540662
Ipmk	84	1.43719809	1.455598605	0.018400514	0.77594391	0.884826334
Larp1b	132	0.92000308	0.887544369	-0.032458711	0.77600401	0.884826334
Wnt7b	184	16.59780731	17.68675756	1.088950253	0.77604708	0.884826334
333420G17F	16	1.280787564	1.490006492	0.209218928	0.7761288	0.884850745
Cct6a	35	1.022837728	1.307936787	0.285099059	0.77622679	0.884893715
Tmco1	58	1.227282959	1.085340385	-0.141942574	0.77661682	0.884925856
Lamtor3	9	1.440625991	1.739826208	0.299200217	0.77642506	0.884925856
Aph1a	14	1.280205683	1.004750826	-0.275454857	0.77653303	0.884925856
Lyrm7	6	1.97275668	3.364636212	1.391879531	0.77640609	0.884925856
Pcgf3	28	1.176056677	1.125593498	-0.050463179	0.77644728	0.884925856
Gria3	9	57.45190136	57.07874996	-0.373151401	0.77657949	0.884925856
Tbc1d20	41	1.189317023	1.322406674	0.133089652	0.77690296	0.885180631

Insl6	16	1.455893513	1.366384087	-0.089509426	0.77696106	0.885180631
Sergef	36	1.582135883	1.515896761	-0.066239122	0.77715803	0.885209713
Ttll12	16	1.434918716	1.533629171	0.098710455	0.7771561	0.885209713
Ccl27a	8	1.533697242	1.932949339	0.399252097	0.77716756	0.885209713
Hira	38	1.441908398	1.487447862	0.045539464	0.77743887	0.885392974
Gabarapl2	47	1.505988305	1.343199784	-0.162788521	0.77744913	0.885392974
Hsf5	72	97.4317073	97.40325331	-0.028453984	0.77760025	0.885496361
BC025920	1	2.542372881	3.191489362	0.64911648	0.77775595	0.885604938
Ankrd16	10	2.546912028	2.981810003	0.434897975	0.77782415	0.885613872
Nckap1l	4	59.20304472	59.7985438	0.59549908	0.77800091	0.885746395
Dbp	24	2.047976338	2.141856426	0.093880088	0.77806612	0.885751921
430091E24R	47	1.64817737	1.299278516	-0.348898854	0.7781633	0.885793827
Snap47	4	0.257731959	0.595238095	0.337506136	0.77825105	0.885825003
Snx4	49	1.37842441	1.41036169	0.03193728	0.77856056	0.886108557
330432E09R	6	39.3099772	39.16251011	-0.147467095	0.77884013	0.88626048
Ctps	56	1.271893645	1.379529401	0.107635755	0.77883789	0.88626048
Spidr	31	1.560553627	1.534875878	-0.025677749	0.77893563	0.88626048
Srd5a1	3	0.548696845	0.529776696	-0.018920149	0.77892523	0.88626048
Supt16	68	1.18667818	1.113592175	-0.073086005	0.7790408	0.886311416
Mtss1	84	1.512037682	1.28538809	-0.226649592	0.77915854	0.886376655
Echdc1	10	1.458482548	1.061593121	-0.396889427	0.77931126	0.88648166
Zfp109	8	1.492912884	1.470866181	-0.022046703	0.77966395	0.886676648
Zc3h3	27	7.511706336	6.599983785	-0.91172255	0.77957998	0.886676648
Blm	34	1.23148185	1.216095218	-0.015386633	0.77964429	0.886676648
Mvp	6	94.88883271	95.35870032	0.469867615	0.77975741	0.886714215
Slc25a24	15	2.007515932	1.76605672	-0.241459212	0.77993058	0.886773692
110036E04R	17	3.714299924	3.478111759	-0.236188166	0.77990237	0.886773692
Nrip1	114	1.255347966	1.189775842	-0.065572124	0.78000598	0.886790716
Glo1	7	1.932701484	1.8235589	-0.109142585	0.78034929	0.887043572
Zc3h15	63	1.680800886	1.389476844	-0.291324041	0.78033142	0.887043572
Mansc1	21	15.50325879	16.3899892	0.88673041	0.78042762	0.887063891
Kif20b	30	1.239923657	0.983951949	-0.255971709	0.78049981	0.887077237
Urm1	10	1.647082288	1.231228335	-0.415853953	0.78087077	0.88743012
Dhx30	65	1.440867794	1.314980389	-0.125887405	0.78093729	0.887436983

Ntrk1	25	11.27597538	12.12570065	0.849725271	0.78116578	0.8876279
Runx2	79	1.193567045	1.051053854	-0.142513191	0.78127938	0.887688245
Gdi2	61	1.226973214	1.267314511	0.040341297	0.78147831	0.887835743
Traip	4	1.275111218	2.25	0.974888782	0.78189323	0.887835743
Grina	20	1.422233723	1.258791574	-0.163442149	0.78169541	0.887835743
Fasn	75	1.378327081	1.287955716	-0.090371365	0.78182473	0.887835743
Pnpla7	33	0.931131968	1.202978663	0.271846695	0.78165718	0.887835743
Tnfaip2	24	5.339340625	5.05777181	-0.281568815	0.78174992	0.887835743
Scfd1	10	1.474019858	2.156526013	0.682506155	0.78183486	0.887835743
Ttc7	35	1.195012169	1.117972788	-0.07703938	0.78187234	0.887835743
Mterf3	86	1.385163571	1.183546043	-0.201617529	0.78198408	0.887870191
Mrps5	36	1.832773965	1.596408566	-0.236365399	0.78216126	0.888002656
Frrs1l	41	16.43903745	17.12602207	0.686984617	0.7822437	0.888027545
Cltb	57	1.83072381	1.556668912	-0.274054898	0.78266868	0.888323977
Slc6a17	61	47.85726845	48.01392236	0.15665391	0.78272826	0.888323977
Taok1	46	1.123739541	1.068134947	-0.055604593	0.78279317	0.888323977
930502E09F	26	1.193036536	0.930719814	-0.262316722	0.78280751	0.888323977
Cct4	39	1.283158842	1.249738482	-0.03342036	0.78279114	0.888323977
Eci2	26	2.01530956	1.806031259	-0.209278301	0.78312012	0.888610007
Thap11	72	1.325506627	1.203345144	-0.122161483	0.78322779	0.888651135
Rabep1	101	1.559254613	1.490256058	-0.068998555	0.78327748	0.888651135
10006O24F	79	1.335288515	1.252436799	-0.082851716	0.78370955	0.889003856
Ube3c	93	0.836558068	0.74228172	-0.094276348	0.78364932	0.889003856
Pawr	64	1.71976546	1.412696571	-0.30706889	0.78378491	0.889020625
Skp2	12	1.879635908	1.934663971	0.055028062	0.78392076	0.889105982
Rplp2	45	0.978393011	1.005596174	0.027203163	0.78432524	0.889423062
Arf4	64	0.946180371	0.82188472	-0.12429565	0.78444277	0.889423062
Prpf8	4	0.45045045	0.235849057	-0.214601394	0.78441894	0.889423062
Pctp	15	1.756048284	1.25120616	-0.504842124	0.78433155	0.889423062
Kdelr1	15	1.247090743	1.768960308	0.521869564	0.78470011	0.889621535
Eno1	26	1.274697199	1.466188751	0.191491552	0.78475096	0.889621535
Lama3	28	6.185015788	6.571798899	0.386783111	0.78481686	0.889621535
Synj1	61	1.065152867	0.855684769	-0.209468098	0.78488767	0.889621535
Dgcr8	64	1.070500238	0.996814519	-0.073685719	0.78492095	0.889621535

BC017643	4	1.42260524	0.597585685	-0.825019555	0.78505264	0.889701355
Cdkn2a	49	2.889033217	2.985409483	0.096376266	0.7851726	0.889701355
Acad11	13	2.022911198	1.745989201	-0.276921997	0.78517327	0.889701355
Hook3	38	0.607888522	0.85421296	0.246324438	0.78542261	0.889915172
Tmem55a	17	1.238087881	1.277350355	0.039262474	0.78549637	0.889930032
Ddit4l	41	7.574632489	7.924678607	0.350046118	0.78564376	0.889959588
Lrch1	36	1.55052494	1.453753401	-0.096771539	0.78562606	0.889959588
Yae1d1	17	0.907012518	1.13074784	0.223735322	0.7857653	0.890028558
Atp11b	88	1.220180429	1.101332289	-0.11884814	0.78662254	0.890881752
Nup62cl	38	58.39022121	57.96863557	-0.421585636	0.78681182	0.890881752
Sass6	36	1.547008093	1.142519424	-0.404488669	0.7868221	0.890881752
930503L19R	7	1.063727622	1.135926733	0.072199111	0.78676326	0.890881752
Phf20	42	1.160644871	1.143013398	-0.017631474	0.78670212	0.890881752
Pou6f1	35	1.640508593	1.656763602	0.016255009	0.78696331	0.890952807
Hipk2	159	1.750455556	1.738912526	-0.01154303	0.78700629	0.890952807
Nrtn	60	1.821687884	1.61451136	-0.207176524	0.78769344	0.89138687
Shox2	97	19.83346757	20.39186012	0.558392546	0.78761622	0.89138687
Fjx1	78	1.968798317	1.715522806	-0.25327551	0.78764386	0.89138687
Net1	57	1.37198325	1.525680732	0.153697482	0.78747383	0.89138687
Usp8	45	0.934206146	1.019795823	0.085589677	0.78765158	0.89138687
Bcas3	10	2.694104806	1.968830465	-0.725274341	0.78815024	0.891766255
Efnb2	106	1.191890023	1.151856646	-0.040033376	0.78809181	0.891766255
Dennd3	25	1.700877291	1.678334355	-0.022542935	0.78832393	0.891894018
Ccdc83	1	5.555555556	5	-0.555555556	0.78867403	0.892221324
Mfrp	10	68.19988916	68.34911171	0.149222558	0.78880717	0.892303147
930563E22R	16	1.126362167	1.127853155	0.001490987	0.7888848	0.892322178
Pola1	27	16.55360766	16.54509465	-0.008513015	0.78896637	0.892345655
330018D20R	39	1.122656338	1.298476924	0.175820586	0.7891337	0.892466119
Slc18a1	13	0.938398163	0.529876395	-0.408521768	0.78944413	0.892550692
Ptger4	46	2.83280631	3.469551637	0.636745327	0.78944345	0.892550692
Lrrc56	43	1.429747394	1.285418546	-0.144328847	0.78945178	0.892550692
Ptprf	59	1.605644807	1.543284335	-0.062360472	0.78941024	0.892550692
Ndufs4	12	1.448136813	1.177961836	-0.270174977	0.78967818	0.892669101
Smim3	23	0.87073102	0.596216946	-0.274514075	0.7896689	0.892669101

Anln	41	0.976655302	1.059767629	0.083112327	0.78985334	0.89279833
Fam69b	34	7.680424598	8.223322267	0.542897669	0.7899518	0.89284085
Arhgdia	112	1.236942643	1.336404148	0.099461505	0.79044257	0.893326729
Pwwp2a	52	1.039580008	1.197186075	0.157606067	0.79099479	0.893795878
Brip1	35	1.192833426	1.14051279	-0.052320636	0.79104042	0.893795878
Cotl1	42	1.27326308	0.866951714	-0.406311366	0.79094973	0.893795878
Ecd	5	0.853658537	0.357142857	-0.496515679	0.79117117	0.89387479
Zkscan14	5	0.921052632	0.789640191	-0.131412441	0.79128353	0.893932905
Rpl37a	14	2.141025111	2.070692819	-0.070332292	0.79167015	0.894300829
330026I12R	1	47.76119403	50	2.23880597	0.79189079	0.894481215
Pstk	31	1.085825697	1.200947285	0.115121588	0.79220493	0.894767181
Tusc2	11	0.951814861	1.209182357	0.257367496	0.79227022	0.894772051
Plekha1	100	1.254635376	1.138771845	-0.115863531	0.79236009	0.894804678
Anapc13	60	1.242337724	1.204889978	-0.037447746	0.7927667	0.895194963
Toe1	9	2.734078881	2.717120449	-0.016958431	0.79290275	0.895279695
Aff1	63	1.084278745	1.115752926	0.03147418	0.79319744	0.895543523
Uhrf1	55	4.283487851	3.995786822	-0.28770103	0.7933881	0.895552074
'00063O14F	1	94.05940594	94.87179487	0.812388931	0.79338258	0.895552074
Hsd17b7	27	1.326584817	1.246235413	-0.080349404	0.7933287	0.895552074
Usp18	19	1.69959794	1.837118436	0.137520497	0.79346254	0.895567215
Pgrmc1	28	19.4372813	19.29904928	-0.138232014	0.79374364	0.895677806
Tmpo	175	1.144173593	1.066729998	-0.077443595	0.79370863	0.895677806
Mxi1	111	1.101835569	1.130760342	0.028924774	0.79363975	0.895677806
Il7	21	1.727493059	1.584601805	-0.142891254	0.79381574	0.895690283
Gsr	34	3.787428918	3.71597793	-0.071450988	0.79397187	0.895797571
Dnajc19	11	2.684140824	1.982160083	-0.701980741	0.79405911	0.895827123
Fzd4	46	1.667673891	1.62517101	-0.042502881	0.79417733	0.895891611
Bzw2	13	1.250870099	1.115461248	-0.135408852	0.79432855	0.895993327
Ola1	49	1.606640763	1.192581927	-0.414058836	0.79443317	0.896042456
Arhgef39	24	0.927598309	0.959379533	0.031781224	0.79460265	0.896164723
Gpr62	16	8.965419976	10.529712	1.564292028	0.79487127	0.896253209
Katnal1	43	1.70275137	1.234230179	-0.468521191	0.79479918	0.896253209
Elp3	7	0.214822771	0.090415913	-0.124406858	0.79492541	0.896253209
Zbtb39	52	1.173682982	1.341583466	0.167900484	0.79482815	0.896253209

Samhd1	16	1.495557927	1.425413776	-0.070144151	0.79516957	0.896338146
Ggct	30	1.742168394	1.415534994	-0.3266334	0.795184	0.896338146
Lacc1	19	0.923714012	1.273375177	0.349661165	0.79517888	0.896338146
Col4a3bp	66	1.223366502	1.373618737	0.150252236	0.7953623	0.896470267
Mroh8	68	1.069656287	1.008578197	-0.06107809	0.79551446	0.896514394
Tmem167b	23	1.139368739	1.269597294	0.130228555	0.79552364	0.896514394
Mir3077	15	0.949215253	1.154742827	0.205527574	0.79583917	0.896801106
Rab11fip4	43	10.6182228	11.26968509	0.651462291	0.79609278	0.897018006
Dck	46	1.269410574	1.319034885	0.049624311	0.79630543	0.897188717
Utp6	22	2.009933067	2.265073088	0.255140021	0.79647189	0.897307368
Acvr2b	65	1.475031937	1.518344309	0.043312372	0.79654818	0.897324431
)30465K10F	49	0.878679905	0.756802248	-0.121877657	0.79666424	0.897386283
Sec24c	18	1.711504719	1.795112466	0.083607747	0.79712672	0.897821022
Hist1h4a	39	1.917405349	1.572480078	-0.344925271	0.79717256	0.897821022
Lrch4	19	1.518719745	1.193293939	-0.325425807	0.79747633	0.897980512
Ppp1r11	7	2.891872036	2.20426979	-0.687602246	0.79749851	0.897980512
Gm20605	19	1.518719745	1.193293939	-0.325425807	0.79747633	0.897980512
Tra2a	29	1.271546198	1.174708263	-0.096837935	0.79755895	0.897980512
Bmp8a	12	5.149361262	4.831454211	-0.317907051	0.79763646	0.897998881
Pggt1b	23	1.324166422	0.954448739	-0.369717684	0.79773264	0.898038265
Zfp157	5	1.608298411	1.869168978	0.260870567	0.79780221	0.898047684
Hs6st1	140	1.343393447	1.151955823	-0.191437624	0.79807128	0.89828166
Cwc25	7	2.002778053	2.633856134	0.631078081	0.79813519	0.898284691
Snx30	33	1.726035878	1.745807072	0.019771194	0.79835634	0.898346473
Coa6	35	1.32457706	1.291909071	-0.032667988	0.7983542	0.898346473
Dnttip1	50	1.005307857	0.901276027	-0.10403183	0.79837375	0.898346473
Fam174a	29	2.239502183	1.740868563	-0.49863362	0.79849323	0.898412026
Kcns2	51	29.54551859	30.67713985	1.131621268	0.79865311	0.898475811
Duox2	1	38.88888889	40.90909091	2.02020202	0.79900548	0.898475811
Mir200c	16	84.60604613	85.38390938	0.777863243	0.79900846	0.898475811
Mthfd1l	19	1.462823145	1.223751246	-0.239071899	0.79903975	0.898475811
Tbc1d13	16	2.089375299	2.248028727	0.158653428	0.79892087	0.898475811
Pou2f1	104	1.275734574	1.080701936	-0.195032638	0.79867393	0.898475811
Tesk2	44	1.296056415	1.707686242	0.411629827	0.79878699	0.898475811

Snrnp35	26	1.698344411	1.847897376	0.149552965	0.79896463	0.898475811
Supt4a	11	1.51948094	1.535176268	0.015695328	0.79913232	0.898511049
Cd55	25	6.471231066	6.708520962	0.237289896	0.79952178	0.898811199
Trpc1	27	1.13775528	1.101380808	-0.036374472	0.79950575	0.898811199
Mtch2	52	1.04381378	1.070386347	0.026572567	0.79967884	0.898918893
Leng1	21	9.549483407	9.606654227	0.05717082	0.79985712	0.899031839
Mier3	120	1.126308985	1.105140727	-0.021168258	0.79990185	0.899031839
Cbr4	15	1.817292957	1.676109609	-0.141183348	0.80043514	0.899493428
Smox	55	1.534775385	1.502500655	-0.03227473	0.80041407	0.899493428
Brd1	137	1.135358566	0.910996342	-0.224362224	0.80075351	0.899588451
Siah2	95	1.244759889	1.329394608	0.084634719	0.80076491	0.899588451
Rfc2	19	1.658435084	1.588949743	-0.069485341	0.80066592	0.899588451
Hrk	87	8.351188846	8.371656145	0.0204673	0.80074079	0.899588451
Lmf2	21	1.345049502	1.484653605	0.139604103	0.8012632	0.900079318
Ppp1cb	37	0.949343007	0.928229248	-0.021113759	0.80152419	0.900303582
Lama5	52	1.663616578	1.634033942	-0.029582635	0.80160376	0.90031854
Alpl	55	11.01309067	13.88828895	2.87519828	0.80166022	0.90031854
Trmt61a	22	0.722613151	0.684841437	-0.037771715	0.80183137	0.900441838
Ncs1	139	2.64383475	2.446858517	-0.196976234	0.80198466	0.900446417
Ppp1r10	60	1.301230849	1.403586343	0.102355494	0.80201954	0.900446417
Inha	3	39.78442728	37.7306286	-2.053798683	0.80197847	0.900446417
Stard3	46	1.188156982	1.215989983	0.027833002	0.80227148	0.900660366
Ppp2r2a	23	2.548692149	2.089722461	-0.458969688	0.80284881	0.9007572
Fam160b2	65	1.549922983	1.46081885	-0.089104133	0.80264722	0.9007572
Pidd1	4	13.29307789	13.61002031	0.316942424	0.80274691	0.9007572
AV051173	16	0.922479365	0.878006542	-0.044472822	0.80273807	0.9007572
Snx16	49	0.994888146	1.037651301	0.042763155	0.80242838	0.9007572
Chchd6	13	0.918121367	0.731060024	-0.187061343	0.80266562	0.9007572
Apcdd1	93	2.530511452	2.354829292	-0.17568216	0.80252451	0.9007572
Pank3	57	1.476974398	1.415886505	-0.061087893	0.80281347	0.9007572
Nubp2	1	11.65644172	12.58741259	0.93097087	0.80330951	0.901205182
Hist1h4i	7	1.487720686	1.346045553	-0.141675133	0.8036812	0.901534533
Plcxd2	17	1.874495873	1.80171234	-0.072783533	0.80397171	0.901534533
OC1026344(	25	74.91325698	74.70010513	-0.213151854	0.80391467	0.901534533

Snord2	25	0.520261896	0.697955238	0.177693342	0.80376325	0.901534533
Taldo1	9	2.417439162	1.817584136	-0.599855026	0.80396098	0.901534533
Mrrf	9	1.78486824	1.745388796	-0.039479444	0.80383248	0.901534533
110001I22R	38	1.816438715	1.616842114	-0.199596601	0.80406088	0.901565629
Tex264	59	1.335877707	1.293645538	-0.042232169	0.80424224	0.901700084
Tcof1	5	1.400460537	1.680506129	0.280045592	0.80432166	0.901720231
Phldb1	5	3.831732829	3.353513774	-0.478219055	0.80454006	0.901896163
Slc7a1	51	0.789612514	0.82104928	0.031436767	0.80466916	0.901971984
Dpp9	44	1.14488167	1.111621051	-0.033260619	0.80490837	0.902102301
Cryab	12	59.78993202	58.50505188	-1.284880141	0.80487928	0.902102301
Tmem30a	52	1.280119887	1.410242639	0.130122752	0.8049825	0.902116484
.10008P14F	33	1.231684925	1.091221619	-0.140463306	0.80517139	0.902259255
Zfp708	13	2.296319842	2.401467167	0.105147326	0.80530684	0.902342132
Ctnna3	33	0.988608385	0.715251743	-0.273356642	0.80554048	0.902509524
Mettl18	8	2.131131613	1.92393637	-0.207195243	0.80557924	0.902509524
Sdc1	30	1.573801689	1.540716964	-0.033084725	0.80564589	0.902515288
Adprh	22	1.237006132	0.941559916	-0.295446216	0.80586321	0.902689832
Mthfd2l	71	1.278873115	1.102933022	-0.175940093	0.80622762	0.902969039
Agfg1	76	1.361168876	1.322618942	-0.038549934	0.80623554	0.902969039
Tctex1d4	1	69.36936937	67.91044776	-1.458921608	0.80647884	0.903034759
Als2cl	14	0.290697674	0.488367303	0.197669628	0.80641267	0.903034759
Acsl5	4	4.954189049	4.573155956	-0.381033093	0.80646104	0.903034759
Golga3	35	1.895093818	1.728190332	-0.166903486	0.806821	0.903348953
Dcun1d3	20	1.675374957	1.873410978	0.198036021	0.80706349	0.903447532
Swt1	44	1.652665222	1.595369425	-0.057295797	0.80700968	0.903447532
Dusp14	83	1.069776029	0.951489548	-0.118286481	0.80709375	0.903447532
Aspscr1	67	1.589232523	1.328328686	-0.260903837	0.80719696	0.903494138
Bcdin3d	28	1.265681864	1.272268857	0.006586994	0.80730895	0.903550568
Nfia	23	13.97684682	12.4005281	-1.576318725	0.80758739	0.903793269
Gba2	26	2.332891675	2.225383334	-0.107508341	0.80797619	0.904159429
Mgat4b	76	1.586753145	1.32715529	-0.259597855	0.80818701	0.904326383
Uvrag	59	1.364821929	1.213373398	-0.15144853	0.80830277	0.904386955
Vcp	28	1.101121693	1.072592552	-0.028529141	0.80858707	0.904636069
Lrrc63	7	1.573347288	1.376190476	-0.197156811	0.80876654	0.904739746

Sdha	22	1.097190532	1.164290329	0.067099797	0.80880305	0.904739746
AV039307	21	2.488098617	2.747176005	0.259077388	0.80902203	0.904806136
310035C23R	26	2.430471339	1.645685936	-0.784785404	0.80898327	0.904806136
Tapt1	167	1.271483908	1.338875173	0.067391265	0.80904738	0.904806136
Cog3	69	1.013385532	0.98864779	-0.024737741	0.8091641	0.904829095
Id1	1	6.598984772	7.168458781	0.56947401	0.80919124	0.904829095
Peli1	57	1.004832143	0.997526054	-0.007306089	0.8093412	0.904927824
Ciz1	24	0.864030044	0.884159293	0.020129249	0.8095489	0.905091085
Tubg1	17	0.708363962	0.68221555	-0.026148412	0.80969397	0.905153948
Slc9a5	10	1.244636171	1.171782623	-0.072853549	0.80979018	0.905153948
Yme1l1	34	1.142069921	1.294241448	0.152171527	0.80975687	0.905153948
Zfp638	41	1.276871743	1.179762212	-0.09710953	0.80985277	0.905154967
Cdk6	46	0.986087139	0.920958564	-0.065128575	0.80992905	0.90517128
Kdm2b	102	1.371177359	1.193330053	-0.177847306	0.81000305	0.905185044
Gskip	41	1.537145182	1.584009548	0.046864366	0.8107277	0.905856867
Tmem220	4	1.075268817	1.298701299	0.223432481	0.81066599	0.905856867
Snord15a	23	1.336411881	1.405807392	0.069395511	0.81088928	0.905956554
Gls	68	0.977766871	0.792554484	-0.185212388	0.81094039	0.905956554
Glrx3	17	1.348250563	1.044887175	-0.303363388	0.81129329	0.906074872
Oaz1-ps	30	0.817535662	0.868984644	0.051448982	0.81114802	0.906074872
Epb4.1l2	52	1.593405501	1.328445287	-0.264960214	0.81122174	0.906074872
Atg4b	44	0.686843831	0.670957682	-0.015886149	0.81123532	0.906074872
Stx6	59	1.103859688	1.308842803	0.204983115	0.81155766	0.906301152
Gps2	42	2.464510168	2.553233834	0.088723666	0.81162499	0.90630737
Hdac10	21	1.519762104	1.49490608	-0.024856024	0.81189415	0.906401
Snord110	23	0.876950782	0.937194687	0.060243905	0.8118802	0.906401
Aco1	27	1.549371317	1.276425344	-0.272945973	0.81189236	0.906401
Diap3	57	1.180465749	1.256032286	0.075566536	0.81205506	0.906511671
Fbxo34	65	1.31864996	1.146078123	-0.172571836	0.81217681	0.906538136
Slc12a7	42	1.171945266	1.099134187	-0.072811079	0.8122851	0.906538136
Cldn12	53	1.636811893	1.508690111	-0.128121783	0.81232588	0.906538136
Slc4a2	45	6.820541383	5.633689113	-1.18685227	0.81223079	0.906538136
Ift122	15	1.410258697	1.218726813	-0.191531884	0.81250291	0.906611549
932412D23R	82	1.49281598	1.364917019	-0.127898962	0.81251523	0.906611549

Pold2	17	0.863277632	1.095022092	0.23174446	0.81259727	0.906634151
Sh3glb1	62	1.099598978	1.276511796	0.176912818	0.812706	0.906686529
Prr14l	18	0.777007245	0.760889449	-0.016117796	0.81298412	0.906927848
Atxn10	16	1.481189095	1.363679234	-0.117509861	0.81312548	0.907016592
Tmem170b	139	0.95188628	1.006039294	0.054153014	0.81322065	0.907053802
Batf3	44	4.874838207	5.350322065	0.475483858	0.81328714	0.90705902
190005I06R	51	1.682211103	1.509223832	-0.17298727	0.81336946	0.907081884
Dph5	5	0.744680851	0.651652591	-0.09302826	0.81363066	0.907098227
Tmem87a	6	0.877192982	0.56709582	-0.310097162	0.81389681	0.907098227
'00017B05F	74	1.183791129	1.216221887	0.032430758	0.81400228	0.907098227
Ubqln4	41	1.669662953	1.562674124	-0.10698883	0.8137282	0.907098227
Zcchc6	50	1.517795013	1.229947816	-0.287847197	0.8139717	0.907098227
Cnn3	90	1.418962589	1.360270694	-0.058691896	0.81373566	0.907098227
Gng10	69	1.030325255	0.763163628	-0.267161627	0.81382074	0.907098227
Ganc	6	0.877192982	0.56709582	-0.310097162	0.81389681	0.907098227
Srebf1	31	1.311466462	1.259998733	-0.051467729	0.81386123	0.907098227
Efhd2	39	1.441589925	1.404645939	-0.036943986	0.8138693	0.907098227
Rem1	42	18.5396091	19.6804618	1.140852696	0.81408505	0.907108182
Mkl1	37	1.209684416	1.271659308	0.061974892	0.81413485	0.907108182
Lactb	55	1.300200332	1.247959061	-0.052241271	0.81427272	0.907192918
Acad12	16	0.856109535	0.54668572	-0.309423815	0.81444202	0.907312648
Zfp260	4	2.156421197	2.333342756	0.176921559	0.81459287	0.90741181
Gtf3c1	35	1.070233067	1.060978816	-0.009254251	0.81487944	0.907618728
Bri3bp	78	1.037320406	0.920733379	-0.116587026	0.81490233	0.907618728
Mir8101	75	1.246371628	1.151278884	-0.095092744	0.81552696	0.90769437
Atp7b	31	1.28153066	1.574402135	0.292871475	0.81507939	0.90769437
Ivd	10	0.977491472	0.952759688	-0.024731783	0.81533996	0.90769437
Prrg4	27	1.077977531	1.045942673	-0.032034858	0.81551205	0.90769437
Slc5a6	20	2.268921364	1.841057028	-0.427864336	0.81545738	0.90769437
'00038G22F	13	0.753839578	0.62139527	-0.132444308	0.8152602	0.90769437
Josd2	25	21.03333451	20.9645707	-0.068763817	0.81529325	0.90769437
Rmdn1	7	0.71942446	0.822416867	0.102992406	0.81540064	0.90769437
Fbxo28	59	1.659856642	1.391071417	-0.268785225	0.81538081	0.90769437
Sh2b3	92	1.420233134	1.25528536	-0.164947774	0.81580395	0.907933806

Faap100	70	1.035285137	0.954238306	-0.081046831	0.81614031	0.908170387
Esco1	60	0.853988875	0.659483402	-0.194505473	0.81613034	0.908170387
Mrpl43	69	0.981168067	0.930085149	-0.051082918	0.81623338	0.908205084
Plekhf1	46	1.058128288	1.095643651	0.037515363	0.81635212	0.908268327
)30427A07F	36	1.291095811	1.559031331	0.26793552	0.8164859	0.908348296
Mrps21	4	94.17377927	93.62206057	-0.551718697	0.81668675	0.908501885
Sh3tc2	5	76.33520356	73.3270362	-3.008167359	0.81674778	0.908501885
Ube4a	25	1.893608205	1.816172174	-0.07743603	0.81694858	0.908506672
Krt27	8	92.08095576	91.38217426	-0.698781498	0.81693816	0.908506672
Coq5	42	1.213018024	1.25797583	0.044957807	0.81699973	0.908506672
Abcb4	51	5.66459686	5.704868111	0.04027125	0.81688506	0.908506672
Vrk2	13	2.199042651	2.045030312	-0.15401234	0.8171567	0.908543523
Arl2	53	1.352721442	1.299431173	-0.053290269	0.8171044	0.908543523
Ripk2	90	1.700353985	1.62309415	-0.077259836	0.81738678	0.908730478
Maea	77	1.88664155	1.807608485	-0.079033065	0.81755885	0.908852916
Fech	41	1.244222681	1.097939138	-0.146283542	0.81766593	0.908903103
Ywhae	43	1.441391422	1.325099449	-0.116291973	0.81783845	0.909026017
Lnpep	122	1.48249865	1.322019207	-0.160479443	0.81794334	0.909073743
Mphosph6	28	3.017034511	2.775504817	-0.241529694	0.81820679	0.909228807
Gm13986	1	13.86138614	14.92537313	1.063986996	0.81818099	0.909228807
Lncbate1	1	80.55555556	79.33884298	-1.21671258	0.81846525	0.909336489
Kif18b	38	1.380248387	1.375424471	-0.004823916	0.8186755	0.909336489
Armc8	26	1.374330366	1.05758254	-0.316747827	0.81849255	0.909336489
Ddx3x	16	2.012708453	1.899812881	-0.112895573	0.81865462	0.909336489
Plscr1	12	1.698064839	1.351277669	-0.346787169	0.81863458	0.909336489
Smad6	47	1.249657347	1.684032612	0.434375265	0.81838621	0.909336489
Gucd1	30	1.630812939	1.56089833	-0.06991461	0.81886994	0.909414776
Ddx10	41	1.784643469	1.599084432	-0.185559037	0.8188653	0.909414776
Ascc2	16	1.462475843	1.796700643	0.3342248	0.81915925	0.909667237
Agpat3	102	1.106902833	1.17207293	0.065170097	0.81938251	0.909707264
Stag3	15	4.667867187	4.518894369	-0.148972818	0.81962926	0.909707264
Ppp2r5d	46	1.6555436	1.545950279	-0.10959332	0.8193037	0.909707264
Cep128	20	1.151639924	1.104783594	-0.046856331	0.81938855	0.909707264
Dedd2	2	2.69655704	3.416149068	0.719592028	0.81956989	0.909707264

Smc3	72	1.120732856	1.005292919	-0.115439936	0.81961339	0.909707264
Cox14	25	1.432581894	1.062060819	-0.370521075	0.8196094	0.909707264
Fam20b	76	1.268016595	1.135351641	-0.132664954	0.82033752	0.910149162
Cnih1	55	0.933586941	0.991414695	0.057827753	0.82027655	0.910149162
Chst14	20	1.49542996	1.477440871	-0.017989088	0.82015694	0.910149162
Zmat4	9	18.61296514	18.33506193	-0.277903211	0.82018936	0.910149162
Prr12	15	3.327672768	3.141507268	-0.1861655	0.82026681	0.910149162
Ptpn13	102	1.508885641	1.441705827	-0.067179814	0.82060683	0.910379115
Cep295	33	1.03771157	0.884702288	-0.153009283	0.82074025	0.9104583
Fam229b	5	0.727272727	0.40405071	-0.323222017	0.82089997	0.910566651
Cbx1	12	1.107636984	1.235385156	0.127748172	0.82109815	0.910648797
Alyref	84	1.037892609	1.070263414	0.032370805	0.82108142	0.910648797
Melk	23	2.120144279	1.884397159	-0.23574712	0.8211634	0.910652333
Tmem2	53	1.779651584	1.664915028	-0.114736556	0.82152698	0.910819077
Lmbrd2	25	1.443552606	1.396894259	-0.046658347	0.82143067	0.910819077
Atxn7l1	22	0.355424994	0.724553413	0.369128419	0.82156204	0.910819077
Fam129a	20	10.61554748	10.71999701	0.10444953	0.82153606	0.910819077
Dstn	27	1.405138482	1.51731389	0.112175408	0.82171488	0.910850899
Exoc2	38	1.316953102	1.109951516	-0.207001586	0.82165641	0.910850899
Zranb3	7	1.023442858	1.322259136	0.298816278	0.82198803	0.911066525
Sgsm3	37	6.217566787	6.051482129	-0.166084658	0.82214646	0.911066525
Akap12	38	23.59417282	25.10240452	1.508231698	0.82215776	0.911066525
Fpgs	52	7.365966746	7.091344085	-0.274622661	0.82205186	0.911066525
930403L14R	58	1.445705097	1.338151671	-0.107553425	0.82245566	0.911327823
Spdye4b	7	1.096182729	1.288673662	0.192490933	0.82272714	0.911333843
Adck5	38	1.3964868	1.352284358	-0.044202442	0.82289583	0.911333843
Cdc25c	10	2.204732632	2.790653983	0.585921351	0.82285159	0.911333843
Zbtb43	41	0.98259183	1.027888253	0.045296423	0.82259824	0.911333843
Eif2ak3	38	1.310866527	1.160272628	-0.150593899	0.8228782	0.911333843
Rpa1	32	0.980808839	0.975599363	-0.005209476	0.82282876	0.911333843
Zfyve27	55	1.490793667	1.065808623	-0.424985043	0.82257734	0.911333843
732491K20R	9	0.408691056	0.614570615	0.205879559	0.82343009	0.911375258
Nol4	41	13.27871728	13.21518457	-0.063532707	0.82342109	0.911375258
Ccdc91	60	1.468801598	1.505237842	0.036436244	0.8231108	0.911375258

Rnf170	78	1.172814978	1.152709138	-0.02010584	0.82308236	0.911375258
Dusp4	68	1.428302828	1.345825613	-0.082477215	0.8232854	0.911375258
Eef2	47	1.029570736	1.152479244	0.122908508	0.82323853	0.911375258
Gnpat	16	0.623944666	0.697986314	0.074041649	0.82336455	0.911375258
Atg2a	20	1.183725927	1.071320822	-0.112405105	0.82315249	0.911375258
Nagk	32	1.898085729	1.951648624	0.053562895	0.82365369	0.911553978
Ttll4	22	1.527555119	1.41596415	-0.11159097	0.82416333	0.911661372
Tdrd3	148	1.05730342	0.985205778	-0.072097642	0.82404129	0.911661372
Taco1	30	1.330505972	1.337372738	0.006866766	0.82426867	0.911661372
Prkrir	86	1.306802734	1.138525226	-0.168277508	0.82412528	0.911661372
Klhl8	43	1.076082573	0.944561679	-0.131520894	0.82391739	0.911661372
Arl6	26	1.092556273	1.068477146	-0.024079126	0.8239805	0.911661372
Utp14a	5	11.21524309	10.86056717	-0.354675918	0.82393668	0.911661372
Pigp	43	1.192419848	1.421816258	0.229396409	0.82430725	0.911661372
Rufy2	19	2.860772206	4.379201311	1.518429105	0.82430987	0.911661372
Gas8	15	4.72587689	4.553981002	-0.171895888	0.82442063	0.91171515
Zfp277	15	0.954548623	0.954160837	-0.000387787	0.82465796	0.911908881
Bok	59	1.343170412	1.387435384	0.044264973	0.82483969	0.912041112
Esrp1	19	9.000799318	9.534767533	0.533968215	0.82510736	0.912268344
Ppp1r35	42	1.398640486	1.322676963	-0.075963523	0.82517203	0.912271103
Dnaic1	10	0.97430681	0.996581291	0.022274482	0.82529749	0.912272339
Rfc3	42	1.907858461	1.86796383	-0.039894631	0.82529316	0.912272339
Ostm1	41	1.008339324	0.908364155	-0.099975169	0.82565097	0.912459637
H3f3a	137	1.162996546	1.062219854	-0.100776693	0.82565347	0.912459637
Nrxn1	7	31.5165521	32.23824826	0.721696161	0.8255434	0.912459637
Slc25a25	129	5.986485358	6.185408229	0.198922871	0.82616065	0.912524183
Ssb	25	1.128646757	1.314434161	0.185787404	0.82591583	0.912524183
Trmt44	17	0.668416603	0.478598267	-0.189818336	0.82620937	0.912524183
Kbtbd12	13	35.46989978	35.71982924	0.249929459	0.82611652	0.912524183
110001J03R	12	1.420583838	1.212287842	-0.208295995	0.82599745	0.912524183
Gopc	38	1.514237055	1.434875586	-0.079361469	0.82586488	0.912524183
Foxk2	156	0.9450257	0.872521335	-0.072504365	0.82599908	0.912524183
Pisd	33	1.17799614	1.423729879	0.245733738	0.82611564	0.912524183
Arhgap1	17	1.909445286	1.920251039	0.010805754	0.82671675	0.913005637

Nek4	23	0.876692307	0.66535101	-0.211341297	0.82676972	0.913005637
Mapk3	57	1.80332269	1.726242816	-0.077079874	0.82691798	0.913100641
Fahd1	17	1.467732264	1.731740927	0.264008664	0.82725213	0.91332826
Gjb3	15	2.828715943	2.220795253	-0.60792069	0.82730952	0.91332826
Nars	21	1.536926743	1.439870853	-0.09705589	0.82731084	0.91332826
Vwa3a	3	45.97552875	46.12772241	0.152193659	0.82767677	0.913562924
Tbc1d32	9	1.190262441	1.432987125	0.242724684	0.82771017	0.913562924
Pelo	33	1.257445174	1.130805318	-0.126639856	0.82767629	0.913562924
10052M02I	2	1.95796101	2.630265893	0.672304883	0.82794559	0.913743098
Srsf6	72	0.907718842	0.834600996	-0.073117846	0.82806022	0.913743098
Rrp8	34	1.753549027	1.75840824	0.004859213	0.82802624	0.913743098
Mon1b	41	2.17016046	1.927395433	-0.242765027	0.82836551	0.914011242
Nsmaf	87	1.400903941	1.27003399	-0.130869951	0.82854423	0.914041613
Zfp689	28	1.084731694	1.108405149	0.023673454	0.828536	0.914041613
Cst3	10	0.751960136	0.46087239	-0.291087746	0.82857991	0.914041613
Rhbdd2	14	0.43304606	0.539923316	0.106877256	0.82867294	0.914075529
Cntrl	45	1.652834916	1.598264065	-0.054570851	0.82892214	0.914144254
Tmem263	62	1.078663915	1.179201019	0.100537105	0.82887442	0.914144254
Mir135b	4	96.35037576	96.83787913	0.487503372	0.8288197	0.914144254
Syt10	18	59.53643664	59.20144525	-0.334991385	0.8292146	0.914398066
Anapc10	12	0.775070731	0.981337905	0.206267175	0.82928153	0.914403153
Lppr2	23	1.930886361	1.832249053	-0.098637308	0.82940579	0.914459529
Bola1	29	1.090079605	1.393607719	0.303528113	0.82951961	0.914459529
Fbxo9	23	1.304169111	1.297674231	-0.006494881	0.82945841	0.914459529
Zdhhc24	20	2.52012397	2.736003762	0.215879792	0.82961189	0.914474313
Bag4	55	0.925218197	1.071775814	0.146557617	0.82965766	0.914474313
Lrrc27	7	0.965709904	0.723892935	-0.241816968	0.82991174	0.914616968
Wwtr1	42	2.388004901	2.292285006	-0.095719895	0.82986745	0.914616968
Crot	38	1.994434659	1.600019311	-0.394415347	0.83005272	0.914645021
Clcn2	63	1.560569255	1.588365885	0.02779663	0.83006812	0.914645021
Rilpl1	28	9.141272921	9.092267988	-0.049004932	0.83012419	0.914645021
Tmem125	6	5.435305435	3.830440976	-1.60486446	0.83022857	0.914682508
Zfp213	17	1.475559818	1.410285531	-0.065274287	0.83028288	0.914682508
Snap29	50	1.637466425	1.526644845	-0.110821581	0.83056133	0.914808844

Mrpl14	21	0.583819791	0.804698584	0.220878792	0.83051084	0.914808844
Aldh6a1	19	1.680068955	1.760728743	0.080659788	0.83058459	0.914808844
Snord55	39	1.300032009	0.977491666	-0.322540344	0.83079342	0.914825384
Upk3a	6	16.94771453	16.04863071	-0.899083822	0.83080796	0.914825384
Ddx52	20	1.00177959	0.863396987	-0.138382603	0.83084898	0.914825384
)10001N08F	20	1.07546255	0.851319564	-0.224142986	0.83068037	0.914825384
Terf2ip	41	1.123127774	0.929903978	-0.193223796	0.8310533	0.914844417
Spdya	78	1.486920357	1.386375649	-0.100544708	0.83104767	0.914844417
Lactb2	29	1.142531082	0.927946881	-0.2145842	0.83093011	0.914844417
Capza1	19	1.268858636	1.245431936	-0.0234267	0.83138812	0.91510327
Snhg3	14	0.532463456	0.772450533	0.239987077	0.83141317	0.91510327
Aqr	11	0.539516309	0.737934551	0.198418242	0.83163724	0.915212609
Ripply2	60	20.44313718	21.80687811	1.363740932	0.83161964	0.915212609
Rassf2	27	5.893184561	5.81860794	-0.07457662	0.83227715	0.915436241
Snhg7	19	0.945651085	1.017210287	0.071559202	0.83209985	0.915436241
Phf21b	98	1.40319568	1.537317708	0.134122028	0.83221341	0.915436241
Kifc1	15	1.565545269	1.467110976	-0.098434293	0.83190706	0.915436241
Snora17	19	0.945651085	1.017210287	0.071559202	0.83209985	0.915436241
Ndufaf4	28	1.981246991	1.552759531	-0.428487459	0.83213197	0.915436241
'00022H01F	28	0.856121503	0.802185339	-0.053936164	0.83227145	0.915436241
Cyp27b1	4	31.98329954	32.24629729	0.262997752	0.83265833	0.915686828
Ergic1	33	1.583271746	1.420533151	-0.162738595	0.83266234	0.915686828
Gpatch1	15	1.886198517	1.389518629	-0.496679888	0.83269218	0.915686828
Ptcd1	8	0.875991272	0.863970395	-0.012020877	0.83283048	0.915770285
Nploc4	60	1.41696079	1.306307249	-0.110653541	0.83302436	0.915777599
Eif3m	31	1.557029467	1.215437431	-0.341592036	0.83300296	0.915777599
Tgfb1	35	1.799731076	1.64829541	-0.151435666	0.83289989	0.915777599
Polk	10	2.19592692	2.617506491	0.421579571	0.83316663	0.91579679
Chmp5	21	1.325631805	1.505432486	0.179800681	0.833115	0.91579679
Ttc28	36	1.167888299	1.064310154	-0.103578145	0.83324458	0.915813868
;10012K16F	12	2.012760673	1.4386505	-0.574110173	0.83338008	0.915894192
Gbas	68	1.483321241	1.424107014	-0.059214228	0.83349312	0.91594983
Midn	108	1.272427021	1.236624859	-0.035802162	0.83369285	0.916000573
Hoxb9	42	20.40739597	20.13219403	-0.275201939	0.83363896	0.916000573

Dhx32	57	1.149666311	1.18634601	0.036679699	0.83422595	0.916000573
Zc3h4	72	1.599504643	1.52564223	-0.073862413	0.83422581	0.916000573
Cul2	45	1.690980417	1.686308378	-0.004672039	0.83381637	0.916000573
030056L22F	30	0.621522678	0.647666671	0.026143993	0.83398179	0.916000573
Myo19	10	1.784073543	1.860888591	0.076815048	0.83385471	0.916000573
Lrrc8b	57	1.321619275	1.092202477	-0.229416798	0.83409221	0.916000573
Idh3g	15	18.17667142	19.02588841	0.849216988	0.83417354	0.916000573
Ice2	15	1.309951001	1.263326474	-0.046624526	0.83396127	0.916000573
Gad1os	24	39.93226146	39.82793344	-0.104328022	0.83402513	0.916000573
310004N23F	2	2.51572327	2.283105023	-0.232618248	0.83428853	0.916000739
Ndufb3	3	3.510887772	3.032456764	-0.478431008	0.8344593	0.91605115
Ccdc82	7	0.336134454	0.549450549	0.213316096	0.83441657	0.91605115
Unc13a	47	0.901778797	0.922984265	0.021205468	0.83461366	0.916152069
Zfp169	24	9.93729224	9.920199239	-0.017093001	0.8347419	0.916155765
Phf13	164	1.46882323	1.324478029	-0.144345201	0.83471183	0.916155765
Psma1	15	1.125718782	1.236128151	0.110409369	0.83494736	0.916251092
310001K24F	12	0.684223547	0.937846069	0.253622522	0.83495363	0.916251092
932438A13F	41	1.184482235	1.337192456	0.15271022	0.83503357	0.916270289
Apoa1bp	42	1.172284932	1.103166091	-0.069118841	0.83535441	0.916321265
Nktr	67	1.091673855	1.049697607	-0.041976248	0.83549048	0.916321265
Cand1	81	1.196720457	1.128212538	-0.068507919	0.83625293	0.916321265
Smad5	130	3.897907956	3.106851036	-0.79105692	0.8367583	0.916321265
Dap3	105	1.312277263	1.181882645	-0.130394618	0.83572823	0.916321265
Atxn7l3	60	1.766544699	1.992012789	0.22546809	0.83527945	0.916321265
Dctn5	8	1.66759054	0.887853741	-0.779736799	0.83552743	0.916321265
Iscu	47	0.790196258	0.800542687	0.010346428	0.83673964	0.916321265
Ttc32	12	9.001080561	7.788106735	-1.212973825	0.83656631	0.916321265
930528A17F	20	1.141014709	1.309714091	0.168699381	0.83548667	0.916321265
P2rx7	5	85.70042285	88.22585996	2.525437116	0.8367899	0.916321265
Akap9	59	1.117765012	1.124116712	0.006351701	0.83575324	0.916321265
Zfp60	16	2.056962094	2.234224971	0.177262877	0.83626578	0.916321265
Tsg101	25	1.553772934	1.340199018	-0.213573916	0.83672349	0.916321265
Cklf	23	1.633644862	1.25335994	-0.380284923	0.83664943	0.916321265
Zfp7	17	5.790739428	4.62783603	-1.162903399	0.83647456	0.916321265

Dmrt2	16	42.77931707	43.18193162	0.40261455	0.8360768	0.916321265
Msmo1	45	1.044869119	1.075346681	0.030477562	0.83682849	0.916321265
Mir8098	25	1.59353993	1.296533948	-0.297005982	0.83589342	0.916321265
Tom1	27	1.406367183	1.174371593	-0.23199559	0.83629635	0.916321265
Chrm2	5	54.6031746	54.35376416	-0.249410446	0.83600259	0.916321265
Cwc15	26	1.393680354	1.433497476	0.039817121	0.83588207	0.916321265
Caprin2	28	1.688307552	1.342000933	-0.346306619	0.83560557	0.916321265
Det1	27	0.816177485	0.80963488	-0.006542605	0.83605738	0.916321265
Dph2	3	3.381503812	3.098245116	-0.283258696	0.83616538	0.916321265
Psma7	42	0.993252695	1.010000805	0.016748111	0.83545152	0.916321265
Kif16b	58	1.742099031	1.501164495	-0.240934536	0.83557087	0.916321265
Zw10	24	1.509761901	1.65142501	0.141663109	0.83643965	0.916321265
Slc14a1	2	54.83146067	53.84615385	-0.985306828	0.83724901	0.916508677
Zfp572	14	45.68334689	46.22069377	0.537346877	0.83711161	0.916508677
Fndc3b	130	1.234178061	1.162602298	-0.071575764	0.83724948	0.916508677
Mir8105	68	0.87202323	0.9657646	0.09374137	0.83713377	0.916508677
Rfx5	7	1.055970174	0.664391019	-0.391579154	0.83759539	0.91679696
Cox18	41	1.367979616	1.587143772	0.219164157	0.83770026	0.91679696
Rhob	80	1.041402848	1.053292757	0.011889909	0.83769218	0.91679696
Pan3	146	1.380323214	1.362801765	-0.017521448	0.83784536	0.916887373
Smarcc2	79	1.066123013	1.02986095	-0.036262063	0.83868876	0.917213344
Vps41	31	1.52159705	1.539108465	0.017511416	0.83845391	0.917213344
Rars	25	1.974363061	1.692015356	-0.282347705	0.83844602	0.917213344
Dusp2	78	2.778976074	2.769876134	-0.00909994	0.83874374	0.917213344
Basp1	28	2.18800919	1.854889473	-0.333119717	0.83845898	0.917213344
Zfp27	2	47.83183366	44.57013575	-3.261697917	0.83869346	0.917213344
Kremen1	105	1.370087664	1.218224074	-0.15186359	0.8388933	0.917213344
Tars	17	2.389179602	2.135128703	-0.254050899	0.83861545	0.917213344
Tmcc1	46	1.333677681	1.281637786	-0.052039895	0.83880761	0.917213344
Arhgef12	130	1.164496986	0.958863076	-0.205633911	0.83823001	0.917213344
Acap2	29	0.811411171	0.991200466	0.179789295	0.83852113	0.917213344
E2f2	23	0.986448959	0.997076783	0.010627824	0.83884137	0.917213344
Dnajc13	52	1.030627217	0.969947911	-0.060679306	0.83914316	0.917320904
Thtpa	6	2.084020048	2.116629701	0.032609653	0.83906113	0.917320904

Trim32	3	5.158730159	5.612244898	0.453514739	0.83919159	0.917320904
Iffo1	30	2.452069201	2.130395634	-0.321673567	0.83924173	0.917320904
Chd9	15	1.30737082	0.917865379	-0.389505442	0.83992296	0.917997135
Nudcd2	15	0.967475241	0.68849877	-0.278976471	0.84006131	0.91806262
Stk10	49	1.824256346	2.071929216	0.24767287	0.8402957	0.91806262
Rassf3	37	1.610308745	1.667954551	0.057645806	0.84022694	0.91806262
Ablim3	43	2.892500589	2.907148157	0.014647568	0.8402736	0.91806262
Naga	15	1.699549373	1.621839651	-0.077709722	0.84029157	0.91806262
Lynx1	6	1.586719732	2.265795207	0.679075475	0.84036867	0.918073992
Hyi	16	0.746804017	0.588767985	-0.158036033	0.84056025	0.918101179
Oxr1	142	1.339901983	1.364922327	0.025020344	0.84064382	0.918101179
Fhl4	8	2.881978806	3.130757985	0.248779179	0.8405837	0.918101179
Comp	8	9.824229708	10.04175674	0.217527028	0.8406345	0.918101179
)00009J06R	20	1.003734469	1.005269072	0.001534603	0.84078574	0.918187834
Stat3	58	1.322974292	1.349527544	0.026553252	0.84100849	0.918294409
Ank	119	1.14321421	0.96535199	-0.17786222	0.84100715	0.918294409
Npat	36	1.090102784	0.997980348	-0.092122436	0.84138911	0.918641648
Arfgef3	28	5.212718621	6.117881144	0.905162522	0.84160981	0.918814247
Fam124a	71	5.720161976	6.662917784	0.942755808	0.84173322	0.918844325
Pcbp4	42	1.527763453	1.386607265	-0.141156188	0.84176259	0.918844325
Fam175a	43	1.104833152	1.19445119	0.089618038	0.84191159	0.918870262
Notch4	4	15.5995935	15.86001248	0.260418987	0.84189027	0.918870262
Ptbp3	95	1.087328385	1.085271208	-0.002057178	0.84199867	0.918896955
Kars	5	3.052358243	2.685496112	-0.366862131	0.84211391	0.918954379
Urb1	14	2.284441782	2.130883716	-0.153558066	0.84244113	0.919243096
Klf10	50	1.499166985	1.325615839	-0.173551146	0.84286335	0.919635419
Inpp4a	29	1.324979652	1.658692941	0.333713289	0.84298855	0.919703639
Dcps	6	1.02124183	0.996902826	-0.024339004	0.84331438	0.919990721
AI597479	32	1.475310534	1.297090512	-0.178220023	0.84346351	0.920078605
Jmjd4	5	0.526315789	0.779220779	0.25290499	0.84352034	0.920078605
Scyl3	63	1.558263676	1.688398104	0.130134428	0.84364035	0.920141105
Sgpl1	71	1.342327031	1.128150895	-0.214176137	0.84374373	0.920185468
Ccnc	32	1.634057653	1.584541813	-0.04951584	0.84401563	0.920413593
Rangrf	28	9.284488696	7.828938156	-1.45555054	0.84413533	0.920447018

Rdh10	182	1.212919945	1.229616603	0.016696658	0.84417173	0.920447018
Mlycd	87	1.096279436	1.071139826	-0.02513961	0.84451735	0.920687038
Nars2	17	1.309613917	0.800845577	-0.50876834	0.84451599	0.920687038
Rplp1	16	0.61007628	0.667230398	0.057154118	0.8448323	0.92096198
Reep3	116	1.349519736	1.13510808	-0.214411656	0.84505012	0.921091265
Ppid	56	1.40204834	1.414576961	0.012528622	0.84507644	0.921091265
Klhl11	49	1.243190644	1.013084391	-0.230106253	0.84544939	0.921331286
Slc20a1	88	1.334510597	1.292487947	-0.04202265	0.84550259	0.921331286
Surf2	16	1.342028114	1.474328703	0.132300589	0.84540703	0.921331286
Pik3r3	32	2.282272083	1.979132732	-0.303139351	0.8455478	0.921331286
Pi4ka	32	0.914844668	0.764958641	-0.149886027	0.84571049	0.921440139
Fam173b	10	0.871271586	0.690639269	-0.180632316	0.84600987	0.921629459
Psmd6	58	1.216471745	1.187720037	-0.028751708	0.84595351	0.921629459
Rad18	52	7.962033521	8.091930608	0.129897086	0.8465913	0.921790258
Pdcd7	67	5.14886213	4.98477881	-0.16408332	0.84660693	0.921790258
Pomk	5	3.074251166	3.160140611	0.085889445	0.84650238	0.921790258
Cfap61	16	0.944472791	0.879144758	-0.065328033	0.84642164	0.921790258
Tab1	49	1.494056285	1.337033496	-0.15702279	0.84644173	0.921790258
Rfc1	35	1.181132385	1.092932514	-0.088199871	0.84647637	0.921790258
Foxl2	88	13.49983323	14.24298801	0.743154776	0.84672284	0.921790258
Tmppe	74	1.305176928	1.115182074	-0.189994854	0.84666821	0.921790258
Samd14	54	1.914777374	2.054172852	0.139395478	0.84652056	0.921790258
Cmtm6	37	0.91200054	0.983261154	0.071260614	0.84682539	0.921833517
Rusc1	53	11.06627522	11.30359529	0.237320073	0.84695778	0.92187439
Heca	86	1.106655003	1.033495778	-0.073159225	0.84698859	0.92187439
Pcdhgb7	7	68.09710609	68.14491394	0.047807854	0.84724852	0.922088908
Thap6	20	0.842189964	0.867768759	0.025578795	0.84740343	0.922189103
)30462N17F	97	1.040531863	0.928771913	-0.11175995	0.84789575	0.922656456
Rab8a	49	1.69085707	1.711328972	0.020471902	0.84815393	0.922868959
Vdac1	36	1.063320573	1.051317341	-0.012003232	0.84837165	0.922911285
Jmjd8	7	15.86615736	15.46433837	-0.401818995	0.84850576	0.922911285
Pnn	57	1.543806034	1.489709771	-0.054096263	0.8485073	0.922911285
Golga2	13	2.093026296	1.649631147	-0.443395149	0.84841472	0.922911285
Stt3a	14	0.288879059	0.327205118	0.038326059	0.84847243	0.922911285

Eml6	34	1.62000139	1.684492917	0.064491527	0.84857137	0.922912556
Rnf169	58	1.241769935	1.051870424	-0.189899511	0.84869358	0.922977068
Mir3960	48	1.420304052	1.489994368	0.069690316	0.84876315	0.922984327
Sbno2	57	0.956553984	1.121275841	0.164721857	0.84889087	0.922986413
Fkbp15	20	1.210948216	1.211060069	0.000111853	0.84888739	0.922986413
Hmg20b	82	1.126867852	1.066806781	-0.060061071	0.84897279	0.923007088
Twsg1	41	1.223101895	1.202364587	-0.020737308	0.84920194	0.923187825
Snord89	1	99.32885906	99.12280702	-0.206052043	0.84948609	0.92323395
Sh3pxd2b	47	0.945863597	0.987848517	0.041984921	0.84970365	0.92323395
Rab11fip2	84	1.737976546	1.582253219	-0.155723327	0.8496501	0.92323395
Sf3b6	29	2.691281604	3.021921281	0.330639677	0.8497477	0.92323395
AY074887	37	1.329384183	1.272896514	-0.056487669	0.84942914	0.92323395
Zfp41	79	1.825265822	1.747417881	-0.077847942	0.84963479	0.92323395
Spsb3	26	28.64355607	27.93140873	-0.71214734	0.84932047	0.92323395
21506M07I	3	80.68965517	79.61234177	-1.0773134	0.84952694	0.92323395
Ypel5	58	1.483355504	1.251564259	-0.231791245	0.84999118	0.923293406
Gpsm1	34	1.821009389	1.659851541	-0.161157849	0.84998394	0.923293406
Crk	79	1.428852211	1.2435379	-0.185314311	0.84998873	0.923293406
Siah1a	131	1.00236283	1.135820382	0.133457552	0.85023599	0.923490962
Fam117b	41	1.478043992	1.292255833	-0.185788159	0.85046123	0.923608424
Kmt2d	148	1.57599297	1.529309906	-0.046683064	0.85047002	0.923608424
Ern2	3	46.13128867	46.59266112	0.461372443	0.85055397	0.923631246
Shank2	56	54.56902201	54.39774538	-0.17127663	0.85062197	0.923636731
Zfp454	4	33.66567879	31.84053623	-1.825142556	0.85075588	0.923713787
Zfp90	36	1.457284879	1.539275	0.081990121	0.85092065	0.923813036
Tm2d2	27	1.508467505	1.424262154	-0.084205351	0.8509732	0.923813036
Slx1b	37	1.47839199	1.343345719	-0.135046272	0.85130536	0.92410526
Pet117	2	0.961538462	1.25	0.288461538	0.85140998	0.924150457
Tbp	45	0.999480702	1.166471448	0.166990746	0.851557	0.92424167
Uimc1	38	1.184018425	1.126169277	-0.057849148	0.85177573	0.924410697
Pip5k1b	119	1.070499636	1.062954715	-0.007544921	0.85197336	0.924496934
Mbd4	17	1.409271692	1.226824087	-0.182447605	0.85198119	0.924496934
00026H17I	19	1.706747144	2.107890427	0.401143283	0.85212531	0.924584942
Zfp668	29	1.031316083	1.619040944	0.587724862	0.85287876	0.925128833

Gm6297	73	1.616473371	1.43315618	-0.183317191	0.85278176	0.925128833
Adk	40	1.253713719	0.938707987	-0.315005731	0.8528595	0.925128833
Tbrg1	26	1.103560128	1.390368252	0.286808124	0.85279972	0.925128833
Cfap36	32	1.556075079	1.598499352	0.042424273	0.85300542	0.925197834
Ankrd26	14	1.612748982	1.70791031	0.095161328	0.85326887	0.925415186
Taf4a	53	0.994406225	1.1853683	0.190962075	0.85348877	0.925585266
Rhod	14	2.445595747	1.86511667	-0.580479077	0.85373238	0.925676391
Tmem120a	23	1.57867197	1.304162498	-0.274509471	0.85376205	0.925676391
Tmem80	27	0.921653364	0.823397924	-0.09825544	0.85372344	0.925676391
Hdac1	47	1.339653371	1.291170322	-0.048483049	0.85436409	0.926260701
Rb1	152	1.246942823	1.441599096	0.194656273	0.85443162	0.92626548
Trim39	97	1.540219298	1.443941966	-0.096277332	0.85457642	0.926285596
Rpp38	8	2.415980066	1.969501102	-0.446478963	0.85453425	0.926285596
Zdhhc17	115	1.221146817	1.098107789	-0.123039028	0.85464839	0.92629518
Smpd4	9	1.558277558	1.326737413	-0.231540145	0.85476784	0.926356228
Anapc16	9	0.707451115	1.038945655	0.331494541	0.85508946	0.926636339
Tube1	26	0.981319401	0.971402585	-0.009916816	0.85565575	0.926989616
Ssr4	11	17.58196383	18.66530438	1.083340553	0.85572211	0.926989616
Plk2	85	1.846099541	1.741279184	-0.104820357	0.85557086	0.926989616
Ckap4	132	1.093768131	1.087180969	-0.006587162	0.85558402	0.926989616
Usp49	57	1.272514357	1.236430176	-0.036084181	0.85573132	0.926989616
Tmem158	70	1.241984493	1.121794411	-0.120190082	0.85590245	0.927106554
'00019A02F	2	96.22082625	96.43077397	0.209947721	0.85623807	0.927401638
Cysrt1	7	38.37182663	38.44392632	0.072099688	0.85634249	0.927446279
Hacd2	36	1.171194866	1.375949594	0.204754728	0.85645114	0.9274955
Rpl27	45	1.592305166	1.535593014	-0.056712152	0.85669163	0.927565274
Mrps18a	39	1.533043837	1.631593748	0.098549912	0.8567052	0.927565274
Sec13	10	1.168632886	1.68494638	0.516313494	0.8566969	0.927565274
Ldha	25	1.076944714	0.964908967	-0.112035746	0.85696732	0.927712171
Rmdn3	50	1.605596244	1.614654402	0.009058158	0.85695973	0.927712171
Ascc1	13	0.777661059	0.985356184	0.207695126	0.85714989	0.927841356
Zgpat	41	1.293273512	1.604454029	0.311180517	0.85736091	0.927884929
Usp31	82	1.187042339	1.119978957	-0.067063383	0.85733799	0.927884929
Ercc4	36	0.991287162	1.152532579	0.161245417	0.85737984	0.927884929

Atr	44	0.736624212	0.788176256	0.051552044	0.8578975	0.928376687
Ccnt2	38	1.17738707	1.198628657	0.021241587	0.85814212	0.928504463
Vps52	43	1.400000103	1.371799165	-0.028200938	0.85813146	0.928504463
130036L24R	38	0.990230183	0.994739237	0.004509054	0.85823536	0.928536875
Rpl9	17	1.265358952	1.154688172	-0.11067078	0.85834352	0.928540729
Polh	10	1.658105219	1.78625118	0.128145961	0.85836548	0.928540729
Dynll2	74	1.009674984	0.918318426	-0.091356558	0.858459	0.928573449
Vamp1	15	0.771484718	0.669114589	-0.102370129	0.85863949	0.928636596
Atp5b	36	1.474108019	1.495166311	0.021058292	0.85864395	0.928636596
Oscp1	42	5.640146811	4.548826832	-1.091319979	0.85885192	0.928784725
Zbtb7a	83	1.505471426	1.305119954	-0.200351472	0.8589075	0.928784725
Tnfaip8	6	1.088206438	1.215460427	0.127253989	0.85902727	0.92880351
Lars	10	2.467985533	2.100483855	-0.367501678	0.85905147	0.92880351
Snx24	97	1.240002925	1.147892965	-0.09210996	0.85937136	0.929080918
Chuk	48	1.232678903	1.282885849	0.050206946	0.85952543	0.929179033
Usp36	30	0.385620746	0.443847246	0.0582265	0.85964015	0.929182452
Safb2	77	0.909288632	0.820142507	-0.089146125	0.85965524	0.929182452
Plxnd1	76	1.631553047	1.831983922	0.200430876	0.85976301	0.929230489
Stk38l	49	1.242562098	1.218655708	-0.023906389	0.86003534	0.929456369
Abra	5	74.77996547	71.79039025	-2.989575215	0.8602592	0.929629829
Fkrp	78	1.829552457	1.607261147	-0.22229131	0.86038053	0.929692478
Pon2	35	1.277337616	1.295157912	0.017820296	0.8611522	0.93028351
Tmem240	61	1.371728798	1.472805588	0.10107679	0.86118108	0.93028351
Alpk3	24	50.79416924	50.2700873	-0.524081936	0.86100115	0.93028351
Rpl4	10	0.817401258	1.101904032	0.284502774	0.86111309	0.93028351
'00034P13R	36	1.352349818	0.981959319	-0.370390499	0.86130556	0.930349487
Cwf19l1	11	0.998997881	1.160959935	0.161962054	0.86141289	0.93039693
Cacfd1	35	1.161725144	1.399855474	0.23813033	0.86154673	0.930417902
00066M21I	58	1.324939838	1.178793889	-0.14614595	0.86156394	0.930417902
Actr1b	29	1.216918797	1.333578434	0.116659638	0.86162252	0.930417902
Rabif	42	1.43609096	1.244569984	-0.191520976	0.86175886	0.930496658
Spata1	78	1.269753359	1.067225835	-0.202527523	0.86196055	0.930645948
Six5	39	1.190635098	1.253377877	0.062742778	0.86231241	0.930888861
Chchd7	50	1.117258802	1.139540733	0.022281931	0.86229658	0.930888861

Ankrd12	112	1.2274419	1.27528999	0.04784809	0.8624825	0.930935508
Carm1	147	1.091809332	1.085476105	-0.006333227	0.86242389	0.930935508
Clip2	56	1.308161837	1.605747008	0.297585171	0.86266746	0.93104653
Klhl2	115	0.982123346	0.97745731	-0.004666035	0.86271226	0.93104653
Gm11627	2	18.91524461	19.56601355	0.650768943	0.86315131	0.931445239
Nudt9	48	1.651619206	1.977937142	0.326317935	0.86320865	0.931445239
Kpnb1	121	1.23962821	1.28292064	0.04329243	0.86328106	0.931454875
Bnip2	62	1.616070015	1.555528145	-0.060541871	0.86357037	0.931698521
Spred1	114	0.877117444	0.864105419	-0.013012025	0.86371503	0.931786083
Tfam	46	1.386619241	1.100774194	-0.285845048	0.86383981	0.931852192
Ccdc88c	83	1.144676978	1.156038222	0.011361243	0.86396132	0.931914762
Uckl1	31	1.275428945	1.21172504	-0.063703905	0.86453081	0.932254934
Ndufaf5	32	1.490328312	1.628692026	0.138363714	0.86448435	0.932254934
Casp2	9	1.125033502	0.954074621	-0.170958881	0.86448062	0.932254934
Yipf5	39	1.716395826	2.027625157	0.311229332	0.86445721	0.932254934
Cops4	19	1.466285792	1.042696515	-0.423589277	0.86460406	0.932265413
Pter	1	4.166666667	4.451038576	0.284371909	0.86477544	0.932381685
Gm13375	125	0.990583629	1.06345695	0.072873322	0.86485197	0.932395695
Mgat3	132	1.883545616	1.729691106	-0.15385451	0.86506728	0.932422306
Polr2j	22	0.912833632	1.079212812	0.16637918	0.86504881	0.932422306
Rrbp1	108	0.857666557	0.875688299	0.018021742	0.86498352	0.932422306
Tm9sf1	75	1.072594639	1.090007936	0.017413297	0.86518282	0.932478346
Lvrn	17	51.54075084	51.96946939	0.428718549	0.86529316	0.932528772
Rnf121	15	1.659505146	1.413392386	-0.246112759	0.8653948	0.932569819
Ngdn	20	2.18024784	2.124575786	-0.055672054	0.86591288	0.933059599
Ddb1	7	1.372410059	2.202635979	0.83022592	0.86607009	0.933112636
Fam60a	15	0.659356288	0.602895381	-0.056460907	0.86608928	0.933112636
Nkiras2	23	1.413888616	1.494302045	0.080413429	0.86625063	0.933128697
BC027231	58	1.730839066	1.726583366	-0.004255699	0.86635855	0.933128697
Lrp8	88	1.135604011	1.124637822	-0.010966189	0.86632509	0.933128697
Ipo4	13	0.706502406	1.064234848	0.357732442	0.86635442	0.933128697
Rpn1	42	1.222730518	1.076106622	-0.146623896	0.86670403	0.933239967
Arhgap9	4	49.15702599	49.41336869	0.256342696	0.86665953	0.933239967
D10Jhu81e	20	1.669821727	1.512848593	-0.156973135	0.86671625	0.933239967

Tmem87b	30	2.058241664	1.744424556	-0.313817108	0.86666177	0.933239967
Ppa2	78	3.195829058	2.951371473	-0.244457585	0.86710015	0.933267374
Imp4	8	2.658680119	1.966239668	-0.692440452	0.86711625	0.933267374
Atpaf1	45	1.490726187	1.404364541	-0.086361646	0.86696623	0.933267374
Inpp5f	34	1.686517715	1.6599564	-0.026561315	0.86711803	0.933267374
Wbscr16	54	1.300666502	1.207099601	-0.093566901	0.86701327	0.933267374
Btbd6	44	1.017178287	0.956311056	-0.060867232	0.86712331	0.933267374
Rgs4	2	8.941176471	8.580123547	-0.361052924	0.86750871	0.933271464
Mllt6	87	1.548856905	1.184117973	-0.364738932	0.86733738	0.933271464
Lrit3	1	98.97959184	99.19354839	0.21395655	0.86740163	0.933271464
Cep162	10	1.702637424	1.891630115	0.188992691	0.86742516	0.933271464
Rsbn1l	69	0.893561957	1.033736212	0.140174255	0.86750762	0.933271464
Fra10ac1	28	1.125129855	0.942360846	-0.182769008	0.86730423	0.933271464
Amd2	2	1.094890511	1.307189542	0.212299032	0.86762032	0.933323116
BC068281	2	1.5625	1.265822785	-0.296677215	0.86777725	0.933423502
Riok1	27	1.690278325	1.864793159	0.174514834	0.86805692	0.933655886
Dlg1	98	4.428742971	4.082645375	-0.346097596	0.86824886	0.93365703
933424G05F	4	92.33174059	91.68643264	-0.645307949	0.86822883	0.93365703
Ddx27	13	2.094683818	1.641181203	-0.453502615	0.86815833	0.93365703
Tango6	32	1.27604957	1.468114586	0.192065016	0.86850492	0.933863938
Nod1	35	1.343850568	1.322502517	-0.021348052	0.86860773	0.933906057
Mir124a-1	6	36.05818024	36.0628747	0.004694464	0.86871888	0.933916589
Fbxl17	129	1.273482997	1.310078874	0.036595877	0.86874482	0.933916589
Eif3g	48	1.384108844	1.570820203	0.186711359	0.86910968	0.934240376
R3hdm4	32	2.012509481	2.11196226	0.099452778	0.86927139	0.934345762
Stim1	37	1.568075874	1.301766046	-0.266309828	0.8693492	0.934360961
Mir137	3	16.26904687	16.81455191	0.545505039	0.86965094	0.934616812
Mgrn1	66	1.421702261	1.432615626	0.010913365	0.8698252	0.934735624
Lrrc51	50	1.466530954	1.371581959	-0.094948996	0.87045954	0.935348803
Zdhhc21	79	1.210078216	1.083499844	-0.126578372	0.87096767	0.935757768
Trim37	84	1.382743027	1.359398954	-0.023344073	0.87094414	0.935757768
Kcna4	14	39.10615717	40.5388528	1.432695633	0.87109912	0.935830478
Trmt5	24	2.036800314	2.03665223	-0.000148084	0.87118711	0.935856491
730420D15F	30	4.871819191	5.206626086	0.334806896	0.87165939	0.93629528

i10020H08F	6	1.115249328	0.89676206	-0.218487268	0.87177057	0.93634617
)30452B06F	35	4.423477087	5.83944518	1.415968093	0.87213108	0.93666482
Atg9a	26	1.051851613	1.140373443	0.08852183	0.87313214	0.937259771
Aldh9a1	42	1.489702778	1.479928068	-0.009774711	0.87310315	0.937259771
Elk4	67	1.056868092	0.862266286	-0.194601806	0.87282084	0.937259771
Kdelc2	42	5.575134264	5.654700828	0.079566564	0.87305194	0.937259771
Parp1	16	1.297228398	1.339835308	0.04260691	0.87275987	0.937259771
Cnpy3	29	1.377998527	1.210514155	-0.167484371	0.87304571	0.937259771
Pmf1	10	1.301760497	1.146649724	-0.155110773	0.87294725	0.937259771
Cyth3	59	1.408224084	1.528220772	0.119996687	0.87335857	0.937434246
.10002H16F	37	1.239330865	1.516675311	0.277344446	0.87356428	0.937586467
Eny2	12	1.03501055	0.830400251	-0.204610299	0.87382235	0.937794868
Pf4	9	58.09083867	57.89038371	-0.200454952	0.87405648	0.937955097
Tigd5	69	1.514069793	1.366492235	-0.147577558	0.87409949	0.937955097
Ube2v2	40	1.078139979	1.187402751	0.109262772	0.87441097	0.938152126
Cep57	29	1.353826412	1.290483764	-0.063342648	0.87435053	0.938152126
Gdpd1	3	2.346597065	1.687382298	-0.659214767	0.87482504	0.93852776
Echdc3	18	8.031628241	8.488846971	0.45721873	0.87501965	0.938530688
Clk1	22	0.900213075	0.926294117	0.026081042	0.87489607	0.938530688
Slc26a11	18	2.191038077	1.750309119	-0.440728957	0.87495619	0.938530688
Mapk8ip3	26	1.346523516	1.166056249	-0.180467267	0.87520513	0.938661022
Hat1	20	1.290251284	1.116265486	-0.173985798	0.87541713	0.938819777
Zc3h7a	42	0.595868663	0.729169292	0.133300628	0.87576	0.939118838
Nmt1	10	1.644693768	0.984606211	-0.660087557	0.87606705	0.939221809
Myo15	1	91.42857143	90.76923077	-0.659340659	0.87607976	0.939221809
Ftl1	19	2.292331627	1.598831771	-0.693499855	0.87624005	0.939221809
Ucp1	5	14.31758953	13.19540871	-1.122180812	0.87613691	0.939221809
Mapk8	98	1.083037718	0.93179111	-0.151246607	0.8762096	0.939221809
Mir5627	18	0.65668294	0.862239656	0.205556716	0.87619149	0.939221809
Bphl	32	1.075224441	1.138488037	0.063263596	0.87668014	0.939428703
Dlec1	20	1.981846734	2.738508976	0.756662242	0.87664968	0.939428703
Atic	42	1.510701503	1.603968941	0.093267438	0.87668916	0.939428703
Cap1	29	2.069523542	1.765668179	-0.303855363	0.8765563	0.939428703
Arpc4	43	1.437360094	1.313328467	-0.124031627	0.87680595	0.939444966

Dis3	81	1.439202531	1.32092584	-0.11827669	0.87683237	0.939444966
Smurf2	123	1.151588884	1.155942874	0.00435399	0.8772371	0.939741368
Ifnar1	66	1.500300476	1.524101122	0.023800646	0.87718759	0.939741368
Hars2	20	1.645159285	1.159179424	-0.485979861	0.87732357	0.939765394
Uba52	40	1.027257165	1.249741563	0.222484398	0.87742643	0.939806971
Cox5b	45	1.292291129	1.299319903	0.007028774	0.87751915	0.939837682
Msl2	104	0.986257699	0.936857924	-0.049399774	0.87762657	0.939884125
Zfp346	46	1.424748259	1.398271194	-0.026477064	0.87778858	0.939989026
Usp24	146	1.250909906	1.237231681	-0.013678225	0.87787081	0.940008479
Sec61a1	67	1.321297131	1.432056911	0.11075978	0.87847096	0.94037663
Dr1	54	0.947185366	0.974018037	0.026832671	0.87837402	0.94037663
Wasf2	43	1.809700702	1.619337519	-0.190363184	0.8783383	0.94037663
Zfp628	36	1.442696595	1.563701882	0.121005287	0.87841627	0.94037663
Gmeb1	56	1.995594623	1.718230065	-0.277364558	0.87878524	0.940644442
Ep400	37	0.843893584	0.959526462	0.115632878	0.8791279	0.940736736
Cd27	3	73.66826443	73.67618765	0.007923219	0.87909193	0.940736736
Fgfr1op	19	1.361875604	1.822552588	0.460676985	0.87906298	0.940736736
Gfi1	5	10.88080952	10.7177505	-0.16305902	0.87900488	0.940736736
Tspan17	46	1.565314825	1.479072552	-0.086242273	0.87935682	0.940844478
Ldlr	19	1.887998776	1.313812376	-0.574186399	0.87932274	0.940844478
Dnajb7	3	90.07633588	93.51419176	3.437855884	0.87945282	0.940878589
Csnk2a2	104	4.368999506	4.262002772	-0.106996735	0.88003177	0.941429332
Ndrg3	54	1.051153129	1.021387909	-0.02976522	0.88031672	0.941665521
Vps13c	5	1.234280391	1.150262152	-0.08401824	0.88041693	0.941704058
Timm10b	2	1.830692244	3.296703297	1.466011053	0.88049273	0.941716498
Azin1	61	1.383746628	1.445435651	0.061689023	0.88060362	0.941766452
Cdc42se2	51	1.159394517	0.961594197	-0.19780032	0.88083219	0.941942251
Pitpnm1	63	2.491124938	2.698775275	0.207650337	0.8812287	0.942128555
Casc5	16	1.030341734	1.067571654	0.037229919	0.88126322	0.942128555
Bckdk	27	1.684501039	1.356492272	-0.328008766	0.88115615	0.942128555
Irf2	21	2.086525465	2.249274807	0.162749342	0.88123837	0.942128555
Nedd1	45	1.420104814	1.157689041	-0.262415773	0.88163677	0.942459235
Bicd1	6	2.035288592	2.936461673	0.901173081	0.88198937	0.942698812
Nadk	25	0.91755137	0.902172929	-0.015378441	0.88197052	0.942698812

Etl4	27	5.073922986	4.91030138	-0.163621606	0.88212086	0.942770679
Lima1	25	24.27838616	23.58429449	-0.694091671	0.88245588	0.942805993
Usp3	75	1.665292247	1.56942922	-0.095863027	0.88229331	0.942805993
Sec23a	34	1.711269037	1.636809329	-0.074459708	0.88247515	0.942805993
E2f4	57	1.570468315	1.549673031	-0.020795285	0.88235653	0.942805993
Snhg8	31	0.78695155	0.872245818	0.085294267	0.88226511	0.942805993
Mrpl41	48	1.067285191	1.22487099	0.1575858	0.88287789	0.943030288
Faim	66	1.164850345	1.411354889	0.246504545	0.8828355	0.943030288
Rsl1d1	4	1.202163895	0.862068966	-0.340094929	0.88277742	0.943030288
Ankrd13b	96	1.162328924	1.167791298	0.005462374	0.88304575	0.943140942
Fam178a	39	1.030897455	1.110172576	0.079275121	0.88317496	0.943149495
Kmt2b	116	1.515342331	1.358574191	-0.15676814	0.88318231	0.943149495
Mrs2	82	1.188290483	1.007359446	-0.180931037	0.88364343	0.943573252
Sys1	65	1.448851766	1.436959773	-0.011891993	0.88378892	0.943591289
Ubp1	94	1.240527111	1.240828403	0.000301292	0.88376093	0.943591289
Plekha5	101	1.440879723	1.290975644	-0.149904079	0.88387128	0.943610556
Ndn	8	59.18526898	59.36992359	0.184654609	0.88476411	0.943739808
Nosip	8	1.601214415	1.426033096	-0.175181319	0.88471989	0.943739808
Mcur1	47	1.02983387	0.772426772	-0.257407099	0.88424256	0.943739808
Prrg2	8	1.601214415	1.426033096	-0.175181319	0.88471989	0.943739808
Adal	63	1.006281823	1.019618606	0.013336783	0.8844546	0.943739808
Hmgxb3	68	1.011505771	0.985094426	-0.026411345	0.88415021	0.943739808
Arhgap21	66	0.887386225	0.950153875	0.06276765	0.8846929	0.943739808
Unc119b	73	1.077092294	1.095329921	0.018237628	0.88426978	0.943739808
Ildr1	24	19.39981516	19.66372034	0.263905179	0.88455379	0.943739808
Dscr3	48	1.14493924	1.164954085	0.020014845	0.88458269	0.943739808
Pth1r	100	10.63228937	11.60799634	0.975706966	0.88448822	0.943739808
110002L01F	25	0.928080278	0.847295005	-0.080785273	0.88413	0.943739808
Mvk	19	1.062915284	1.047281154	-0.01563413	0.88484629	0.943758868
Itpripl2	38	1.817588893	1.558339276	-0.259249617	0.88528238	0.94415536
Tmem192	66	1.335592346	1.060508294	-0.275084052	0.88543089	0.944224444
Actr5	42	1.017382081	0.987406762	-0.029975319	0.88561701	0.944224444
Hnrnpll	47	0.948253813	0.950469134	0.002215322	0.88564267	0.944224444
Serinc1	47	1.181508469	1.119971211	-0.061537258	0.88566889	0.944224444

Bzrap1	5	10.10549944	10.14657972	0.041080285	0.88552921	0.944224444
Herc1	41	1.208980056	1.139537681	-0.069442375	0.88596303	0.944469411
Nfs1	8	1.165428365	1.049279039	-0.116149326	0.88607084	0.944515729
Wdr7	9	1.647202407	1.396647181	-0.250555226	0.88649222	0.944553213
Ppp1r37	58	0.978670629	0.849446302	-0.129224328	0.88643937	0.944553213
Ube2j1	80	1.109689739	1.09118168	-0.018508059	0.88627565	0.944553213
St3gal3	73	1.567191914	1.373156875	-0.194035039	0.88628407	0.944553213
Zpr1	47	1.272444058	1.366965597	0.094521539	0.88633559	0.944553213
Slc25a12	53	1.278872694	1.329857015	0.05098432	0.886438	0.944553213
Mir7665	1	97.97979798	98.24561404	0.265816055	0.8867904	0.944733721
Rxrb	83	1.303473217	1.404700746	0.101227529	0.88674607	0.944733721
Cbx3	77	1.229152592	1.154513083	-0.074639509	0.88693714	0.944821461
Ago3	47	1.404716746	1.273150962	-0.131565783	0.8872821	0.945120316
Snhg5	31	0.834992625	0.787757632	-0.047234992	0.88756998	0.945289731
Ipo5	112	1.330830997	1.285993573	-0.044837424	0.88753385	0.945289731
Osgep	17	0.873395392	0.928839869	0.055444477	0.88774282	0.94540519
Efemp2	53	8.926768404	8.661960061	-0.264808342	0.88796239	0.945435382
Mrps18b	58	1.33219654	1.409933727	0.077737187	0.88796446	0.945435382
Hps4	34	1.264453013	1.404672914	0.140219902	0.88785706	0.945435382
Mir7074	2	67.27564103	66.53858785	-0.737053177	0.88815977	0.945533745
Zfp956	4	1.71901528	1.480812013	-0.238203267	0.88818571	0.945533745
Rell1	63	1.33484816	1.325026572	-0.009821588	0.88839666	0.945637775
Atp1a1	73	2.107721861	1.642081491	-0.46564037	0.88841232	0.945637775
Sepn1	62	0.933315994	0.868298886	-0.065017108	0.8885814	0.945680548
Orai1	71	1.338282346	1.21928821	-0.118994136	0.88855279	0.945680548
.30060C02F	30	0.836273856	0.661441487	-0.174832369	0.88894266	0.945860676
Gstm5	26	2.958902014	2.895846046	-0.063055967	0.88894402	0.945860676
Stau1	71	1.297196433	1.250787029	-0.046409404	0.88892868	0.945860676
Ptp4a1	73	1.248313666	1.264953444	0.016639779	0.88907376	0.945861547
Efr3b	57	3.516215877	3.950451393	0.434235516	0.88901221	0.945861547
Lman2	7	1.371457258	2.665622389	1.294165131	0.88947138	0.946099728
'00034H15F	34	1.040047349	1.333172566	0.293125217	0.88948008	0.946099728
Adi1	18	1.177291493	1.160199509	-0.017091984	0.88949106	0.946099728
Ndufa9	22	1.161989675	1.628550633	0.466560958	0.88957018	0.946115298

Alox12	9	10.66076097	11.30184659	0.641085613	0.88964029	0.946121297
Gtf3c5	35	1.442310381	1.041024294	-0.401286087	0.8897892	0.946211078
Hibch	31	2.944788079	1.800721303	-1.144066776	0.89002615	0.946325902
Lins	20	1.764419673	1.702354882	-0.062064791	0.88998208	0.946325902
Rnf187	77	1.143659631	0.846893427	-0.296766204	0.89025925	0.946505164
Traf3ip1	71	1.190827461	1.032951563	-0.157875899	0.89038806	0.946573523
Ankra2	54	1.11355363	1.022700223	-0.090853407	0.89045926	0.946580639
Bicd2	19	1.094400488	0.705168799	-0.389231688	0.89080884	0.946843848
Upf3a	69	1.590441436	1.568911418	-0.021530018	0.89111703	0.946843848
Recql	45	1.399449302	1.162544492	-0.23690481	0.89111829	0.946843848
Rfxap	74	1.136876754	0.964191107	-0.172685647	0.8910791	0.946843848
Aco2	29	1.243500294	1.233197262	-0.010303032	0.89093203	0.946843848
Lrp10	57	1.358506596	1.200100931	-0.158405666	0.89117288	0.946843848
Glrx2	79	1.00408672	0.815718899	-0.188367821	0.89122306	0.946843848
Tgds	19	1.3937543	1.289517771	-0.104236529	0.89099131	0.946843848
Slc1a5	1	4	4.347826087	0.347826087	0.89150134	0.946876822
Gm4890	16	1.101660345	0.843576326	-0.258084019	0.89142496	0.946876822
Aar2	16	1.232660574	1.336504117	0.103843544	0.89151221	0.946876822
Inf2	49	1.587372427	1.744084711	0.156712284	0.89148021	0.946876822
Prkcsh	5	2.111290918	2.87608971	0.764798792	0.89164086	0.946944919
Yy1	164	1.180349224	1.018093593	-0.162255631	0.89171393	0.946953988
Sfpq	24	1.611835943	1.495219265	-0.116616679	0.89203916	0.947230812
Lmo7	51	3.623396207	3.436620175	-0.186776032	0.892204	0.947337304
.10056K07F	30	2.164181775	2.000044496	-0.164137279	0.89244718	0.947458392
Iqgap1	62	1.357170245	1.253641823	-0.103528422	0.89239761	0.947458392
Klhdc8a	78	4.229544643	4.412002268	0.182457625	0.89287352	0.947842436
Cacng3	17	33.09327228	35.75190658	2.658634299	0.89314275	0.947853962
430416N02F	46	1.252683066	1.127459019	-0.125224047	0.89312	0.947853962
Tmem104	27	0.84367643	0.78450963	-0.0591668	0.89308317	0.947853962
Slc35b2	28	1.500192792	1.556227951	0.056035159	0.8930475	0.947853962
Cutc	34	0.867787286	0.884154228	0.016366942	0.89341175	0.948070869
Zbtb45	16	1.209415639	1.04574729	-0.163668349	0.89366975	0.948261684
Ccdc115	15	2.17449379	1.51292601	-0.66156778	0.8937208	0.948261684
Tug1	88	1.260748347	1.337840661	0.077092314	0.89394972	0.948284444

Rbm45	25	1.022920043	0.952365645	-0.070554398	0.89385169	0.948284444
Samd4b	98	1.235703216	1.342182224	0.106479008	0.89400075	0.948284444
Thoc5	29	1.413221395	1.447198179	0.033976784	0.89392784	0.948284444
Aste1	25	1.114222899	0.914788425	-0.199434474	0.89425752	0.948446183
700018L02R	28	0.613481602	0.750535306	0.137053704	0.8942825	0.948446183
Hsf2	52	0.803197969	0.717747056	-0.085450913	0.89482981	0.948958056
Prkab1	37	1.721779551	2.542480399	0.820700848	0.89510973	0.949141221
Hace1	38	0.996875064	0.989130555	-0.007744508	0.89513189	0.949141221
Rheb	88	1.227906175	1.279124061	0.051217887	0.89536261	0.949317263
Slc41a3	14	72.31393005	70.5974515	-1.716478545	0.89548849	0.949382136
Polr2l	10	0.518809657	0.651567944	0.132758287	0.89565388	0.94948888
Tbcb	19	1.090225132	1.107895923	0.017670791	0.89573271	0.949503853
Dym	57	1.537094831	1.461125152	-0.075969679	0.89583515	0.949543845
Rptor	14	4.430743076	3.894673112	-0.536069965	0.89642381	0.950099169
Pfdn4	26	16.21404789	16.27759427	0.06354638	0.89668113	0.950303253
700120C14R	12	0.913621672	0.809143012	-0.10447866	0.89709718	0.950675527
Rnf19a	84	1.235055496	1.18682268	-0.048232817	0.89748031	0.950760397
Gramd4	82	1.052412397	0.941299793	-0.111112604	0.89749732	0.950760397
Mir33	4	74.80053191	77.66650579	2.865973877	0.89745989	0.950760397
Pigg	22	1.657825396	1.295614422	-0.362210974	0.89750123	0.950760397
Psma5	24	0.808325281	0.700593174	-0.107732108	0.89737284	0.950760397
Tfap4	32	0.996346357	0.771348705	-0.224997652	0.8976783	0.95076591
Smkr-ps	29	1.528977897	1.467526952	-0.061450945	0.89767165	0.95076591
Mcm8	24	1.052162007	1.062658954	0.010496947	0.89770853	0.95076591
Polr1b	14	1.808416936	2.739380802	0.930963866	0.8977656	0.95076591
Acbd5	36	1.440478439	1.290394525	-0.150083914	0.89870875	0.951554228
Zfp438	30	0.801637157	1.146585189	0.344948032	0.89881856	0.951554228
Rfc5	34	1.331630897	1.377772457	0.04614156	0.89889307	0.951554228
Dppa3	3	82.49342238	79.88749969	-2.605922699	0.89892521	0.951554228
Ephb3	63	12.82549839	12.75528346	-0.07021493	0.8989639	0.951554228
Hist3h2a	11	1.189460586	0.837005889	-0.352454696	0.89886065	0.951554228
Kmt2e	9	0.703181664	0.770336884	0.067155221	0.8988344	0.951554228
Atg3	37	1.931223674	2.066994113	0.135770439	0.89916538	0.951643327
Lars2	3	4.065248635	4.304669842	0.239421208	0.89924263	0.951643327

Plaa	51	1.455074048	1.323005848	-0.1320682	0.8992355	0.951643327
Ttc21b	27	1.647891361	1.610960291	-0.03693107	0.89939627	0.951668656
Cfap58	14	2.768384192	3.260941414	0.492557222	0.89934573	0.951668656
Sh2b2	20	1.813145129	1.586501003	-0.226644126	0.89952915	0.951740625
Gxylt2	35	17.52872602	18.44879502	0.920068996	0.89991285	0.95180842
Lsm1	69	0.893285816	0.929189587	0.035903771	0.89980726	0.95180842
Dph3	33	1.605146843	1.673061745	0.067914903	0.89975083	0.95180842
Dynll1	57	1.171209551	1.116554525	-0.054655027	0.89991754	0.95180842
Tox4	17	1.491275697	1.451320152	-0.039955545	0.89973496	0.95180842
Pdxp	78	1.316816777	1.361690721	0.044873944	0.90052585	0.952100941
Glyr1	42	1.163559505	0.886857858	-0.276701647	0.90057385	0.952100941
Ifrd1	32	1.0913356	1.118373801	0.027038201	0.90048463	0.952100941
Upp1	11	1.704444746	2.348901099	0.644456353	0.90039237	0.952100941
Atxn7l3b	77	1.410392234	1.279524516	-0.130867718	0.90058342	0.952100941
Tradd	36	1.031014443	0.898195074	-0.132819368	0.90043499	0.952100941
Opa3	27	1.014684793	1.000971822	-0.013712971	0.90068589	0.952140679
Evi5l	28	1.708744874	1.484190402	-0.224554472	0.90091581	0.952315131
Gpatch8	69	1.53815925	1.55003019	0.01187094	0.90112593	0.952468621
Fancc	19	1.324870495	1.604904495	0.280034	0.901323	0.952608303
Tet3	45	2.742659087	2.52399203	-0.218667058	0.90163615	0.95287064
Rps11	42	0.953945207	1.019551635	0.065606428	0.90177949	0.952891241
Setd1a	106	1.168875783	0.965614063	-0.20326172	0.90178552	0.952891241
Cul3	125	1.095794222	1.079303872	-0.016490349	0.90185251	0.952893412
Slc17a5	35	1.010057484	0.940441326	-0.069616158	0.90194694	0.952924578
10032A03F	5	1.648756219	1.891457708	0.242701489	0.90230836	0.953237793
Dlg2	7	42.86296429	42.83091277	-0.032051525	0.902382	0.953246954
Tab2	137	1.437826408	1.455559036	0.017732628	0.90274143	0.953337233
Trappc2l	14	1.102812191	0.973876272	-0.128935918	0.90266959	0.953337233
Uros	36	1.535132716	1.524567442	-0.010565274	0.9027923	0.953337233
Rps6ka1	84	13.54052313	12.78271251	-0.757810616	0.9025731	0.953337233
Yod1	32	1.624418234	1.986951002	0.362532768	0.90272042	0.953337233
Mettl5	10	2.060990287	1.256377035	-0.804613252	0.90288649	0.953368089
210404O09F	9	1.901965139	1.900071168	-0.001893971	0.90299242	0.953411336
Ccdc86	18	2.106894011	1.645787338	-0.461106672	0.90327342	0.953639408

Htatip2	30	2.471270584	2.725769115	0.254498531	0.90375382	0.953754489
Mical3	6	33.94973903	35.24745757	1.297718534	0.90381359	0.953754489
Tulp4	95	1.324188212	1.249816824	-0.074371388	0.90381271	0.953754489
N6amt1	7	2.826436305	3.092529406	0.266093101	0.90379873	0.953754489
Pfn1	82	1.224573098	1.171387877	-0.053185221	0.90383739	0.953754489
Hilpda	16	1.557171221	1.195471256	-0.361699965	0.90369433	0.953754489
Scamp2	14	0.918303825	0.847389839	-0.070913986	0.90356694	0.953754489
Sema3a	1	4.430379747	4.694835681	0.264455934	0.9039848	0.953829986
Chchd1	27	1.066244305	1.049076689	-0.017167616	0.90403894	0.953829986
Ktn1	51	0.743664455	0.617964456	-0.125699999	0.90416004	0.953889169
)10300C02F	82	2.028831741	1.884495665	-0.144336076	0.90449425	0.953940891
Slc29a2	12	1.55579108	0.941374411	-0.614416669	0.90447449	0.953940891
Gtpbp8	30	3.568876897	3.590037408	0.021160512	0.90443856	0.953940891
Clcn3	53	0.865112343	0.776922918	-0.088189425	0.90459912	0.953940891
Chn1os3	2	97.20938375	97.30037752	0.090993766	0.90459772	0.953940891
Gm9776	18	0.880888791	0.750546703	-0.130342088	0.90459313	0.953940891
Vopp1	48	1.495259955	1.498028502	0.002768546	0.90475804	0.953976078
Asxl1	91	1.248102361	1.251435243	0.003332882	0.90488154	0.953976078
Ttc1	3	2.159823381	2.682070781	0.5222474	0.90482118	0.953976078
Mir7059	5	58.03036581	59.72555263	1.695186819	0.90489253	0.953976078
Rpsa	57	2.61751625	2.505000165	-0.112516085	0.9051029	0.954129308
Mmadhc	6	1.153039832	0.796788881	-0.356250951	0.90525866	0.954156415
Ankrd40	52	1.889971811	1.596146724	-0.293825087	0.90524598	0.954156415
Obfc1	28	1.44251251	1.359704888	-0.082807622	0.90556824	0.954414163
Slc52a2	23	2.357066625	2.016903929	-0.340162696	0.90564882	0.954430531
Hspa5	36	1.351720825	1.537946371	0.186225546	0.90578634	0.954472034
E2f7	119	1.015787375	0.959680375	-0.056106999	0.90581829	0.954472034
Senp3	15	0.851601049	0.628140517	-0.223460533	0.90598574	0.954579936
Kdm4b	47	1.346064364	1.322739162	-0.023325202	0.9063452	0.954890112
Kif1b	115	1.203130183	1.206441278	0.003311095	0.90655638	0.954930089
Prosc	38	1.241041844	1.316662132	0.075620288	0.90657837	0.954930089
Sh2d5	10	1.226403866	1.350365014	0.123961148	0.90650042	0.954930089
Olfml2a	9	8.99471511	10.54198955	1.547274438	0.90677571	0.955069397
Atoh8	73	1.520999703	1.433094275	-0.087905428	0.90694806	0.955089101

Ss18	128	1.360348411	1.211429774	-0.148918637	0.90690843	0.955089101
Lipa	8	2.868736059	2.382588255	-0.486147805	0.90698968	0.955089101
230103J11R	13	1.572058023	1.228004143	-0.34405388	0.90717998	0.955220941
Zfp14	5	5.256770614	5.095772773	-0.160997841	0.90742732	0.955412818
Mdc1	38	1.49010819	1.477572011	-0.012536179	0.90773508	0.955668288
Naa38	30	1.584137615	1.516020833	-0.068116781	0.90788466	0.95575719
Erp44	19	0.472138815	0.425806546	-0.046332269	0.90807597	0.955868107
Rhoq	93	1.173200004	1.246513796	0.073313792	0.9081203	0.955868107
Mcu	41	1.373650178	1.243910161	-0.129740017	0.90836273	0.956054701
Eftud1	22	2.356452096	1.785249608	-0.571202488	0.9086675	0.95630689
Sox8	7	1.563207435	2.98272317	1.419515735	0.90873434	0.956308646
Slc25a42	38	1.325891631	1.083105286	-0.242786345	0.90890844	0.956423262
Lect1	1	86.02941176	86.53846154	0.509049774	0.90958744	0.95702136
Tmem59	37	1.452732513	1.323583929	-0.129148584	0.90960726	0.95702136
Chrna9	1	88.60759494	88.28125	-0.326344937	0.9098426	0.957200333
'00001G17F	3	1.471584102	1.512893465	0.041309364	0.91018531	0.957218151
Agfg2	42	1.296928279	1.30385265	0.00692437	0.91018569	0.957218151
Zzef1	29	0.789980028	0.763128518	-0.026851509	0.91011272	0.957218151
Rrp36	20	1.58919376	1.467032942	-0.122160818	0.91003173	0.957218151
Ckmt1	9	7.197722394	7.736465728	0.538743334	0.91017198	0.957218151
Pik3c2a	42	1.37522581	1.360247849	-0.014977961	0.91031916	0.957289902
Sema3f	19	0.95502895	1.050625609	0.095596659	0.91091127	0.957508869
Agpat5	75	1.269424691	1.307587427	0.038162737	0.91069004	0.957508869
Syf2	52	1.901608812	1.567565453	-0.334043359	0.91099505	0.957508869
Atg16l1	64	1.057626517	0.969865363	-0.087761153	0.9110494	0.957508869
Hspb3	7	85.72841361	84.99601801	-0.732395599	0.91082412	0.957508869
Stard10	108	2.236501239	1.858366572	-0.378134668	0.91097608	0.957508869
Acot13	46	1.198622953	1.055686243	-0.142936709	0.91089338	0.957508869
Cog7	43	1.375124505	1.425453667	0.050329163	0.91081063	0.957508869
Dis3l2	38	1.524343398	1.929130469	0.404787071	0.91136845	0.957775597
Bend7	98	1.286784847	1.295720511	0.008935664	0.91145327	0.957796142
Ddx1	16	1.147075735	1.524621734	0.377546	0.91156097	0.95783225
Mpv17	2	0.458715596	0.367647059	-0.091068538	0.91161818	0.95783225
Rdx	57	1.099311768	1.209252664	0.109940896	0.91177756	0.957931115

Gm608	56	2.269180378	2.276356614	0.007176236	0.91205017	0.958080334
)33427G17F	8	2.453262811	2.58497807	0.131715259	0.91198634	0.958080334
Sccpdh	103	1.262362804	1.30116726	0.038804455	0.91213913	0.958105194
Mybl1	73	1.216723098	1.103292848	-0.11343025	0.91227275	0.958176964
Gmppb	16	1.546005377	1.135731893	-0.410273484	0.91248255	0.958191567
Umps	19	2.061357035	1.909544348	-0.151812687	0.91241861	0.958191567
Keap1	68	1.098870385	1.118398095	0.019527709	0.91247239	0.958191567
Pgap1	37	1.076702704	1.080232699	0.003529995	0.91269618	0.958347317
Hes1	4	2.192982456	2.915140415	0.722157959	0.91299008	0.958587326
Nit2	1	4.643962848	4.469273743	-0.174689105	0.91306404	0.95859639
Mmaa	68	1.495773607	1.402048025	-0.093725582	0.91318225	0.95862621
Frs2	117	1.309928381	1.009621306	-0.300307075	0.9132231	0.95862621
Ccdc58	46	0.824540744	0.863627371	0.039086627	0.91332635	0.958666016
Usp46	96	1.257245518	1.224961839	-0.032283679	0.91369145	0.958912056
Gm23363	6	48.91468646	48.4535165	-0.461169954	0.91368075	0.958912056
Kri1	44	1.464569343	1.441097939	-0.023471404	0.91390405	0.959066582
Homez	48	4.301671093	3.93587943	-0.365791663	0.91399248	0.959090792
Sdad1	32	1.818894964	2.054623754	0.23572879	0.91416299	0.959201119
Ccl22	2	78.44827586	75.72685953	-2.721416328	0.91429544	0.95927151
Ehd1	83	1.205218474	1.174509874	-0.0307086	0.91456609	0.959349692
Sbno1	15	2.14358228	2.097015255	-0.046567025	0.91453665	0.959349692
Hadha	18	1.50783419	1.487408095	-0.020426095	0.91455472	0.959349692
Snhg17	12	1.596437245	2.032720917	0.436283672	0.91463394	0.959352282
Tcn2	10	3.146352643	3.36966238	0.223309736	0.91472164	0.959375694
Naa40	62	0.868325701	0.789079416	-0.079246286	0.91485357	0.95944549
B3galnt2	53	1.201600643	0.945753948	-0.255846695	0.91522681	0.959768323
Cnst	70	1.206196482	1.197877278	-0.008319204	0.91529397	0.959770168
Bbc3	41	1.328957046	1.643180563	0.314223517	0.91562204	0.960045575
Pabpc1	72	1.232682916	1.066728656	-0.16595426	0.91571409	0.960073488
Psmb2	13	2.622638932	2.435857089	-0.186781843	0.91583441	0.960131033
Klhl28	18	1.964428417	2.017494964	0.053066548	0.91615068	0.960393995
)10026O09F	17	1.080953214	0.938130613	-0.142822601	0.91624882	0.960428258
Srp72	45	1.103338651	1.12989953	0.026560879	0.91696893	0.961114433
Usp20	15	0.60318621	0.912749494	0.309563284	0.91710412	0.961187472

Etf1	52	1.440348228	1.305142684	-0.135205543	0.91724556	0.961267055
Agrn	136	1.694431436	1.690388907	-0.00404253	0.91738265	0.961342073
Ctnnbl1	17	1.876234239	1.753383158	-0.122851081	0.91749831	0.961394616
Arl6ip6	72	1.231690376	1.16050536	-0.071185016	0.91759928	0.961431759
Qk	118	1.097862591	1.011791687	-0.086070904	0.917725	0.961494831
Mapkapk5	76	1.010544303	1.058819673	0.04827537	0.91801673	0.96173181
Sik1	101	1.111077228	1.115707642	0.004630414	0.91808491	0.96173458
Tlk1	181	1.236434975	1.140386294	-0.096048682	0.91822794	0.961776483
Uqcc3	17	1.341558266	1.057206577	-0.284351689	0.91827844	0.961776483
Slc26a9	7	69.43418331	70.26672085	0.832537546	0.91832154	0.961776483
Tmem179b	9	0.628727037	0.52013791	-0.108589127	0.91851924	0.961914881
Arfgef1	122	1.127464096	1.069727341	-0.057736755	0.91873246	0.961932227
Ccnj	110	1.979373971	1.928330806	-0.051043166	0.91867308	0.961932227
Pds5b	69	1.222581976	1.081373658	-0.141208318	0.91872043	0.961932227
Tbl1x	59	19.1775335	18.93700481	-0.240528693	0.9189579	0.962099611
Abhd3	39	4.428955408	5.083881454	0.654926047	0.91910082	0.962151434
Inpp5e	32	0.993034913	1.181076384	0.188041471	0.91913853	0.962151434
Sft2d2	40	1.319005385	1.29856854	-0.020436845	0.9194581	0.962417307
Pkn3	20	2.650572435	2.539439947	-0.111132488	0.91958838	0.962485009
Fam89b	43	13.56680809	13.25289052	-0.313917573	0.91965596	0.962487094
Ube2f	92	1.118803859	1.148606913	0.029803054	0.91983063	0.962543677
Celf1	73	1.470502333	1.211031713	-0.25947062	0.91984121	0.962543677
Pafah1b2	81	1.17467101	1.127714682	-0.046956328	0.92019723	0.962847562
Pcgf6	36	1.042554893	1.03559656	-0.006958333	0.92028906	0.962874988
H2afy	78	1.649518197	1.539688204	-0.109829994	0.92072703	0.963058557
Fastk	66	5.392571426	5.327986909	-0.064584517	0.92071143	0.963058557
Vapa	147	1.238968366	1.114759788	-0.124208578	0.92070244	0.963058557
Slc25a10	35	1.201948505	1.394622894	0.192674389	0.92068031	0.963058557
Txlng	10	19.66977319	19.8125716	0.142798405	0.92131731	0.96351281
Hnrnpul2	16	1.176612528	0.791173636	-0.385438891	0.9213514	0.96351281
Ephb4	6	1.035860275	1.476793249	0.440932974	0.9213583	0.96351281
Higd1a	55	1.409752023	1.419188325	0.009436302	0.92154518	0.963639562
Cenpq	10	1.696958497	2.175428089	0.478469592	0.92168312	0.963679327
Calu	86	1.678091503	1.432190646	-0.245900857	0.92171455	0.963679327

Larp1	113	1.086501134	1.036200373	-0.05030076	0.92184677	0.963748892
Krit1	10	1.460807724	1.732456914	0.27164919	0.92201792	0.96385916
U2af2	55	1.09131778	1.018154994	-0.073162786	0.92213397	0.963911803
Anks3	10	0.562861153	0.531562116	-0.031299037	0.92254883	0.964002106
Taf1c	16	1.518518066	1.514324951	-0.004193115	0.92238488	0.964002106
Mrpl32	5	0.881542087	1.111943375	0.230401288	0.92248017	0.964002106
Gpcpd1	68	1.057403865	1.10470499	0.047301125	0.92253066	0.964002106
Adamts20	93	67.06004126	67.30957206	0.249530803	0.92250371	0.964002106
Fam104a	65	0.67502131	0.800036381	0.125015072	0.92282055	0.964038969
Grhl1	138	1.676007189	1.53836714	-0.137640049	0.92270313	0.964038969
110037F02R	36	1.257741512	1.290569369	0.032827856	0.92273661	0.964038969
Fam120b	3	5.79096887	4.869688985	-0.921279885	0.9229126	0.964038969
Fsd1l	54	3.104249981	3.435607671	0.33135769	0.92287446	0.964038969
Gm6981	145	1.05114997	1.023008305	-0.028141665	0.92310252	0.964168727
Rcc2	35	1.218642918	1.190535655	-0.028107263	0.9232412	0.964244941
Arhgef2	86	12.24721255	11.85850996	-0.388702595	0.92350573	0.964379637
Fbxo47	19	1.605249705	1.693309933	0.088060229	0.92355896	0.964379637
Trappc6b	38	1.469406771	1.411687653	-0.057719117	0.92363305	0.964379637
Olfr212	1	99.39759036	99.31034483	-0.087245534	0.92359027	0.964379637
Ilf3	46	1.244459234	1.379509161	0.135049928	0.9237447	0.964427587
Wdr73	7	2.729213593	2.968813727	0.239600134	0.92384849	0.964467328
Letm2	24	1.446496667	1.333345866	-0.113150801	0.92392705	0.964480725
Dpp10	25	39.04964075	39.56139853	0.511757782	0.92403245	0.96452213
Rn4.5s	9	74.11876512	72.80744843	-1.311316691	0.92433255	0.964766753
Pvr	37	1.2588988	1.298129425	0.039230626	0.92442642	0.964796106
Mpp7	24	1.373606013	1.236029398	-0.137576615	0.9246283	0.964936138
Pnpla8	50	0.964599038	0.892236417	-0.072362621	0.92475786	0.964936138
Tmem242	5	1.584549878	1.602683031	0.018133153	0.92471132	0.964936138
Zfp386	30	1.070774688	0.866379096	-0.204395593	0.92490527	0.965021323
Nudt6	60	1.233222445	1.100749378	-0.132473067	0.9252268	0.965261935
Dlgap2	36	47.39437552	46.98311283	-0.411262696	0.92533322	0.965261935
Rnf41	18	1.364140928	1.248914416	-0.115226512	0.92527851	0.965261935
Flii	37	1.168683441	1.206349019	0.037665577	0.92542812	0.96529231
Dgat1	74	1.228165557	1.182045842	-0.046119715	0.92568714	0.96542524

Pmpcb	18	0.821125983	0.721253385	-0.099872598	0.92566493	0.96542524
Phlda1	92	1.111571247	1.028052788	-0.083518458	0.92603478	0.965628012
Pttg1ip	9	1.795272536	1.280440803	-0.514831733	0.92607898	0.965628012
Med9os	10	0.90069086	2.254653263	1.353962403	0.92597102	0.965628012
Tmem205	15	1.45965496	1.406575826	-0.053079134	0.9262465	0.965665441
Ddx31	32	1.247685832	1.24800624	0.000320408	0.92622263	0.965665441
Abcb8	10	0.910316756	0.740980097	-0.16933666	0.92631832	0.965671716
Suv39h2	55	0.860137351	0.918343622	0.058206271	0.92675293	0.966056156
Amd1	19	1.422497286	1.394670164	-0.027827122	0.92710774	0.966297194
Lig4	16	1.6961741	1.312352784	-0.383821316	0.92711586	0.966297194
Grk6	97	1.489503817	1.443905957	-0.04559786	0.92724576	0.966363939
Rarg	55	2.611583219	2.499734925	-0.111848295	0.92758719	0.966582471
Ndfip2	86	1.196303623	1.035659239	-0.160644384	0.92757694	0.966582471
Iqca	11	11.34711942	13.99981823	2.652698811	0.92769005	0.966621013
Atp8b3	7	4.383547491	4.214657908	-0.168889583	0.92808724	0.966768834
Gm12522	12	1.980749738	1.489405136	-0.491344603	0.92813558	0.966768834
Wee1	112	1.252252929	1.012542605	-0.239710324	0.928125	0.966768834
Itga4	16	25.86057969	26.21371368	0.35313399	0.92815617	0.966768834
Fam149b	9	1.086212657	1.183738658	0.097526001	0.92816133	0.966768834
Maf1	23	0.605691599	0.833659003	0.227967404	0.92824896	0.966791486
Iws1	28	1.074584978	0.95124948	-0.123335499	0.9286692	0.966833799
Smchd1	114	1.188950236	1.155100074	-0.033850162	0.92868491	0.966833799
Zc3h12c	85	1.532856143	1.471257298	-0.061598845	0.92843869	0.966833799
Slc16a6	99	1.402062055	1.277405875	-0.124656179	0.92855225	0.966833799
Amacr	24	1.358505101	1.423374309	0.064869207	0.92837714	0.966833799
Tbc1d19	25	1.447685361	1.350650696	-0.097034665	0.92863256	0.966833799
Mettl3	5	1.590070251	2.314967105	0.724896854	0.92877058	0.966854388
Aim1	3	1.777078857	2.086552559	0.309473702	0.92920316	0.967236087
Trub2	11	1.775656983	1.775374444	-0.000282539	0.92937161	0.967342819
Stat5b	100	1.319786496	1.289932884	-0.029853612	0.92953451	0.96737513
Ism2	38	1.833833302	1.985120379	0.151287077	0.9295013	0.96737513
Asap3	19	1.568845714	1.540327103	-0.028518611	0.92963545	0.967380524
Zfp821	88	1.207152501	1.184369371	-0.022783129	0.92967154	0.967380524
Hmgcl	52	2.78102024	2.673232713	-0.107787528	0.92985874	0.967506711

Dtwd1	26	1.632606438	1.245922011	-0.386684427	0.93003534	0.967621855
Ndufs3	1	4.776119403	4.918032787	0.141913384	0.93036512	0.967786781
Ilk	14	2.612545181	2.314148346	-0.298396835	0.93039172	0.967786781
Trim50	4	65.75741331	65.05607079	-0.701342523	0.93026979	0.967786781
Zswim6	171	1.375638415	1.185933542	-0.189704873	0.93050022	0.967831032
Prkd3	76	1.556770074	1.386170698	-0.170599376	0.93060818	0.967874723
Dvl3	70	1.447477875	1.224906395	-0.222571479	0.93076554	0.967969771
Polr3c	10	2.08109834	2.213871466	0.132773126	0.93084502	0.967983832
'00094K13F	12	1.574641954	1.806240218	0.231598263	0.93096137	0.968036221
Cox16	6	3.140575701	2.646846473	-0.493729228	0.93138786	0.96839649
Smim7	24	1.396301591	1.129390342	-0.266911249	0.93143983	0.96839649
Eef1g	9	1.019023157	1.06688796	0.047864803	0.93210101	0.968471585
Smarca4	18	1.075714182	1.085193535	0.009479354	0.93159456	0.968471585
Cfdp1	45	1.369659063	1.104870904	-0.264788159	0.93193921	0.968471585
Pwp1	21	1.732917806	1.335840204	-0.397077602	0.93210605	0.968471585
Elovl1	25	2.696776501	2.338968649	-0.357807851	0.93210241	0.968471585
Ehmt2	114	1.889807433	1.738752739	-0.151054693	0.93201795	0.968471585
Ttc25	19	0.864696685	1.146694503	0.281997818	0.93174226	0.968471585
Capn2	77	1.253160785	1.073935237	-0.179225548	0.93198071	0.968471585
Ngfr	30	12.10736218	11.60914677	-0.498215407	0.9318807	0.968471585
Clpx	40	1.431732192	1.44782568	0.016093488	0.93221715	0.968478123
Fbxo11	133	1.241120228	1.224778654	-0.016341574	0.93224434	0.968478123
Ccdc124	15	1.62548622	1.5461919	-0.07929432	0.93232487	0.968493218
Scn1b	79	1.347516927	1.410103734	0.062586807	0.93266225	0.968657503
Oard1	15	1.106273012	0.741575825	-0.364697187	0.93258747	0.968657503
Gpr19	25	13.40753867	12.61126124	-0.796277432	0.93268106	0.968657503
Susd3	1	15.70247934	16.10169492	0.399215576	0.93275476	0.968665484
Scg3	1	50	49.58333333	-0.416666667	0.93286058	0.968706824
Gpatch2l	21	1.287574535	1.19018808	-0.097386455	0.93336597	0.969094473
Htr1a	6	25.43181818	26.43528914	1.003470955	0.93330192	0.969094473
'00012D14F	14	1.68319592	1.802359981	0.119164061	0.9337644	0.969323062
Dgcr2	23	1.270890055	0.957633653	-0.313256403	0.9337843	0.969323062
Cenpc1	15	0.911968551	0.864775139	-0.047193412	0.93365392	0.969323062
Tmem9b	62	1.384467099	1.582834884	0.198367785	0.93386727	0.969340623

Tmem168	32	1.658087637	1.582719269	-0.075368368	0.93441237	0.969583963
Olfr314	1	97.2972973	97.1659919	-0.131305394	0.93443208	0.969583963
Cyhr1	56	1.433317443	1.591416784	0.15809934	0.93431617	0.969583963
Mir6916	9	93.1775465	94.04713868	0.869592184	0.93439585	0.969583963
Lsm8	51	1.116252448	1.12257333	0.006320882	0.93435965	0.969583963
Isg20	1	62.83185841	63.31360947	0.48175106	0.93452461	0.969611402
Invs	30	1.708140807	1.247813889	-0.460326919	0.93489773	0.969929955
Morf4l1	14	1.273763567	1.097256208	-0.176507359	0.93567583	0.970410895
Armc6	30	1.437969765	1.250683893	-0.187285872	0.93578535	0.970410895
Hdac3	13	1.139955673	1.283623466	0.143667793	0.93582422	0.970410895
Rrp1	22	0.996675881	1.137792483	0.141116602	0.93573236	0.970410895
Zfp458	3	1.461219939	1.412429379	-0.048790561	0.93580923	0.970410895
Gm12669	47	1.201656779	1.112907577	-0.088749203	0.93565114	0.970410895
Spsb1	33	1.654407857	1.324509848	-0.32989801	0.93566887	0.970410895
Cahm	74	1.002921283	0.933606091	-0.069315192	0.93595366	0.970476546
Taf9	96	1.140844767	1.108228824	-0.032615943	0.93668268	0.970986424
Hey1	116	1.605370819	1.499612108	-0.105758711	0.93670705	0.970986424
Gmcl1	89	1.195112745	1.237908424	0.042795679	0.9366578	0.970986424
Bmpr1a	154	1.380200711	1.30178768	-0.078413031	0.93671009	0.970986424
Rnf38	108	1.115178983	1.148267624	0.03308864	0.93680446	0.971015655
Fbxl15	11	3.516264644	3.22140489	-0.294859755	0.93692226	0.971069161
Bcl7a	101	1.52316688	1.380197673	-0.142969207	0.93701519	0.971093708
Pi4k2a	58	1.079086585	0.875688347	-0.203398237	0.93715362	0.971093708
Zbtb33	58	18.4790323	18.89576956	0.416737264	0.93747232	0.971093708
Fam195a	25	1.359100587	1.494535513	0.135434927	0.93735707	0.971093708
Abl2	56	1.545997437	1.430312522	-0.115684915	0.93746198	0.971093708
Cage1	14	1.651188212	1.614594147	-0.036594066	0.93747536	0.971093708
Fastkd2	2	4.255319149	4.444444444	0.189125296	0.93717479	0.971093708
Pik3cb	132	0.991746776	0.944460119	-0.047286657	0.93732349	0.971093708
Asf1b	52	1.542211745	1.499950111	-0.042261634	0.93777248	0.971127271
Tekt1	7	35.3820598	37.41223462	2.030174814	0.93765395	0.971127271
Acp6	18	1.413105294	1.300598068	-0.112507226	0.93771321	0.971127271
Impa2	8	1.356950211	1.263872452	-0.093077759	0.93774043	0.971127271
Dhx34	6	0.650406504	0.528058993	-0.122347511	0.93787892	0.971160447

230037L18F	25	1.923343144	2.055855135	0.13251199	0.93806925	0.971160447
Eefsec	40	1.460196773	1.221287504	-0.238909269	0.93803216	0.971160447
Llgl1	62	1.159777456	1.086023094	-0.073754363	0.93803601	0.971160447
Slc30a9	83	1.376241935	1.371941294	-0.004300641	0.93827802	0.97123954
Pcdhgc4	12	11.16906953	11.45378635	0.284716822	0.93821881	0.97123954
Rnf214	52	1.463880121	1.415357152	-0.048522969	0.93854029	0.971305465
Egfl6	2	22.87878788	22.25306838	-0.625719498	0.93850039	0.971305465
Cxxc4	13	1.033430161	1.231215132	0.197784972	0.93841436	0.971305465
Dpy19l4	55	1.490676544	1.405598794	-0.08507775	0.93910876	0.971825243
Apbb1	12	1.050213925	0.892875281	-0.157338644	0.93919574	0.97184672
Poln	13	1.882310319	1.817816225	-0.064494094	0.93926484	0.971849682
Alkbh5	99	1.074461103	1.097554647	0.023093543	0.93938163	0.971901999
H3f3b	56	1.25391264	1.238037193	-0.015875448	0.93955476	0.971977028
Pif1	7	2.411679705	1.547797161	-0.863882544	0.93958663	0.971977028
Pip4k2c	35	1.425094866	1.401344764	-0.023750102	0.93973384	0.972060785
Tspan3	53	1.645016943	1.313163461	-0.331853482	0.93993196	0.972197192
Appbp2os	24	0.795371153	0.913104571	0.117733418	0.94015044	0.97235463
AI413582	42	1.719232102	1.315233929	-0.403998173	0.94038032	0.972523845
Uhrf1bp1l	47	1.772542342	1.776727244	0.004184901	0.94063668	0.972670922
Haus8	23	1.285577646	1.229918936	-0.05565871	0.94065511	0.972670922
Ccnf	53	1.275629435	1.346581456	0.070952021	0.94095529	0.972775671
510005L07F	13	24.3997978	21.40444019	-2.995357611	0.94094451	0.972775671
Map1a	23	7.521057411	7.392526037	-0.128531374	0.94093099	0.972775671
Lsm6	50	1.363215627	1.372675762	0.009460135	0.94106724	0.972822868
Pex2	20	1.131401592	0.993361771	-0.138039821	0.94132901	0.973024932
Cbl	64	1.220430061	1.137000379	-0.083429683	0.94139712	0.973026788
Cops8	24	0.763974347	0.648085627	-0.115888719	0.94152463	0.973090046
Sidt2	38	1.316485394	1.287374978	-0.029110416	0.94174381	0.973248024
Snhg20	11	0.736362367	1.034062711	0.297700344	0.94214556	0.973594645
Ddr1	81	12.87015415	11.96774247	-0.902411684	0.94228179	0.973598295
Tspyl1	27	1.103597934	0.940898309	-0.162699625	0.94226591	0.973598295
Got2	9	1.061129593	1.335625979	0.274496385	0.94238495	0.973636325
Lyar	40	1.834880714	1.832225003	-0.002655711	0.94251273	0.973699792
Npc1	49	1.21566632	1.160518242	-0.055148078	0.94276449	0.973822763

Chmp4b	42	1.217702219	1.127761704	-0.089940515	0.94272689	0.973822763
Smpd1	8	2.165069406	1.842169568	-0.322899838	0.94320622	0.974073344
Sesn2	77	1.335797358	1.535086983	0.199289626	0.94319556	0.974073344
Slbp	16	0.81703657	0.990655323	0.173618752	0.94316586	0.974073344
Kdm4c	53	1.177151124	1.032198557	-0.144952567	0.94360342	0.974377419
Atxn7	70	1.532746079	1.397163029	-0.135583051	0.94363347	0.974377419
Zc3h11a	26	1.429999299	1.409408795	-0.020590503	0.94415624	0.974437236
Xrcc2	19	1.661086825	1.306836602	-0.354250223	0.94411697	0.974437236
Anapc15	10	1.576893181	1.718869947	0.141976766	0.94379972	0.974437236
Usp15	55	1.884885242	1.777229255	-0.107655987	0.94399435	0.974437236
E2f1	78	1.188175873	1.107886108	-0.080289764	0.94402976	0.974437236
Tubb2b	7	1.26984127	1.07022607	-0.1996152	0.94385132	0.974437236
Sumo1	37	1.205559933	1.157534817	-0.048025116	0.94395519	0.974437236
Tmod3	39	1.29495958	1.234391673	-0.060567907	0.94459018	0.974747978
Rps21	16	1.193792699	1.081795892	-0.111996808	0.94457993	0.974747978
Pou4f1	114	10.50709334	11.1444934	0.637400059	0.94503547	0.975089789
Cenpp	18	1.233401049	0.983476491	-0.249924558	0.94512076	0.975089789
Usp30	65	1.36604485	1.347628035	-0.018416815	0.9451047	0.975089789
Dtx4	39	1.458789636	1.142069688	-0.316719948	0.94537401	0.975282495
Pde7a	168	1.131965339	1.032347587	-0.099617753	0.94549337	0.97533706
Ctdsp2	41	5.936508946	5.668248743	-0.268260203	0.94561994	0.975399055
Prkca	100	1.200876235	1.228567579	0.027691344	0.94569481	0.975407717
Spry1	49	0.936415905	0.858868671	-0.077547234	0.94586054	0.975510089
Ccdc51	10	1.718001068	1.50034057	-0.217660498	0.94599475	0.975579943
Ubr7	47	1.26051717	1.354652806	0.094135637	0.94635463	0.975882486
Zfp688	19	0.977052723	1.475516423	0.4984637	0.94643951	0.975901441
Spdl1	35	1.72920816	1.571016375	-0.158191785	0.94656337	0.975960573
Trip10	8	2.321782635	1.975560398	-0.346222237	0.94680107	0.976137067
Trp53	1	4.705882353	4.494382022	-0.21150033	0.94691696	0.976187959
Napepld	14	2.702483351	2.001932248	-0.700551103	0.94698759	0.976192199
Ndufs5	27	1.385239802	1.557459351	0.172219549	0.94760668	0.976727593
230046K03R	68	0.764418014	0.619822446	-0.144595568	0.94764009	0.976727593
Gm12191	2	0.434782609	0.612244898	0.177462289	0.9477709	0.976793809
Ypel1	43	1.219717121	1.588151728	0.368434607	0.94801222	0.976962184

Kdelr3	10	1.025523831	1.540767586	0.515243755	0.94806743	0.976962184
510012G03F	67	1.353659065	1.155682326	-0.197976739	0.94881631	0.977253496
Mob1a	50	1.263353784	1.18136582	-0.081987964	0.94881287	0.977253496
Ncoa5	48	1.049404899	1.131199897	0.081794998	0.94864239	0.977253496
Sp5	28	10.87721919	11.14006053	0.262841339	0.94879719	0.977253496
Gpr12	45	50.05775407	50.00270445	-0.05504962	0.94878475	0.977253496
Scd2	7	2.351351949	2.401522333	0.050170383	0.94849838	0.977253496
010315B03F	9	1.506189588	1.880827974	0.374638387	0.94881473	0.977253496
Pnp	16	1.753721774	1.239098241	-0.514623533	0.94904446	0.977351283
Cyb5r1	27	1.351074609	1.423434481	0.072359872	0.94897903	0.977351283
Bccip	9	1.454243226	1.152893198	-0.301350028	0.94915144	0.977392857
Wars	14	1.693564972	1.434311846	-0.259253126	0.94946574	0.977594093
Timm21	35	1.358112473	1.416464186	0.058351712	0.9494801	0.977594093
10306M01F	17	1.712515522	1.629309999	-0.083205523	0.94994662	0.977848383
Dvl1	100	2.284713854	2.295899833	0.011185979	0.94988755	0.977848383
Wdr35	17	1.430221551	1.768141736	0.337920185	0.94999363	0.977848383
Vsx2	37	25.80325637	27.8950879	2.091831528	0.94981573	0.977848383
Peli3	27	2.39981104	1.880654082	-0.519156958	0.95014354	0.977905665
Sec62	35	1.044814521	1.204765957	0.159951436	0.95018257	0.977905665
Klhdc2	77	1.230554552	1.124235833	-0.106318719	0.95040435	0.977928159
Pmm2	22	1.608323374	1.576850519	-0.031472855	0.95039524	0.977928159
Tmem134	21	1.472408289	1.419803391	-0.052604898	0.95035299	0.977928159
Ccdc106	13	36.6584943	38.56566841	1.907174104	0.95073439	0.978199156
Hist1h3e	6	0.880990355	1.575075075	0.69408472	0.95102972	0.978312687
Slc35a4	23	1.868418229	1.59734809	-0.271070139	0.95103094	0.978312687
Itfg1	50	1.095079448	1.214448183	0.119368735	0.95104474	0.978312687
Snrpd3	8	2.146742198	2.289339186	0.142596988	0.95190684	0.97909613
.10057O12F	13	2.325040952	2.210866366	-0.114174586	0.95193979	0.97909613
Setx	36	1.374703418	1.246036782	-0.128666636	0.95205939	0.979150507
Pacsin2	43	1.064733268	0.853490826	-0.211242442	0.95220948	0.979234624
Lrrc42	51	1.708901725	1.700250856	-0.008650869	0.95234137	0.979234624
Nsfl1c	3	1.459854015	0.979245499	-0.480608515	0.95231237	0.979234624
Pdp1	110	1.387689608	1.210662975	-0.177026633	0.95242393	0.979250889
Rab20	46	2.597024363	3.147310421	0.550286058	0.95260304	0.979366426

Mgat5	17	1.234616908	1.461448752	0.226831843	0.95292156	0.979419416
Klhl42	19	0.808082634	1.041465764	0.23338313	0.95286938	0.979419416
Maml1	53	1.282638161	1.038627798	-0.244010363	0.95276863	0.979419416
C1rb	3	8.591065292	8.712121212	0.12105592	0.95290482	0.979419416
Sec23b	11	0.824362351	0.948642989	0.124280638	0.95317201	0.979608214
Tmem189	94	1.282819	1.309317365	0.026498365	0.95338482	0.979689693
Spryd3	67	1.766544332	1.75902812	-0.007516213	0.95337087	0.979689693
Atp5c1	14	1.855308428	2.031409565	0.176101137	0.95362118	0.979863954
Itga3	52	4.721182341	4.767786881	0.04660454	0.95406081	0.979972587
Akap8l	8	1.329677498	1.437635371	0.107957873	0.95388106	0.979972587
Spc25	13	0.809412259	0.866012292	0.056600033	0.95400323	0.979972587
Cuedc2	14	1.48177561	1.391438725	-0.090336885	0.95386694	0.979972587
Txndc17	32	1.322821492	1.456068423	0.133246931	0.95398497	0.979972587
Ergic3	17	1.298755425	1.050850007	-0.247905418	0.95466362	0.980523128
Cpeb3	41	1.210001523	0.907642729	-0.302358794	0.95492987	0.980659309
Lonrf3	23	62.28127821	62.31687921	0.035600993	0.9548647	0.980659309
Slk	70	0.846080728	0.906546539	0.060465812	0.95501315	0.980676205
Klhl41	5	96.65485672	96.32894508	-0.325911636	0.95515432	0.980752538
Ska3	9	0.168350168	0.254066921	0.085716752	0.95537187	0.980907277
Kdm1a	27	1.495757367	1.108319328	-0.387438039	0.95549324	0.980957801
Slc39a1	20	1.364950133	1.766388627	0.401438493	0.95555478	0.980957801
Fam161b	5	2.942976804	2.926403316	-0.016573488	0.95606453	0.981412447
Lrrc47	96	0.841228089	0.909251178	0.06802309	0.95625497	0.98153927
Cyld	33	1.010919839	0.945354871	-0.065564967	0.95640667	0.981626321
Osbpl9	19	2.064632101	1.830350422	-0.234281679	0.95652438	0.981678471
Slc4a11	25	1.492006052	1.080006394	-0.411999658	0.95664927	0.981737983
Ppard	28	1.221069087	0.812184195	-0.408884892	0.95698039	0.981940439
Pura	62	1.173911816	1.103519584	-0.070392233	0.95692024	0.981940439
Tmem115	24	1.16674611	0.936574529	-0.230171581	0.95708459	0.981978694
Psma6	34	1.659046752	1.266839996	-0.392206756	0.95717374	0.982001497
Cyp4f41-ps	6	2.712395132	2.778430713	0.066035581	0.95730597	0.982068495
Cep250	38	1.212847601	1.192591028	-0.020256573	0.95758903	0.982290206
Fbxo10	80	1.293379831	1.227963899	-0.065415932	0.95771891	0.982354767
Zfp110	22	1.311792944	1.302650861	-0.009142083	0.95789717	0.982468941

Brms1l	77	1.473532206	1.321702114	-0.151830092	0.95822403	0.982693283
Rnf114	34	3.890297086	3.738238808	-0.152058278	0.95838024	0.982693283
Rnf219	40	1.024083999	0.881720116	-0.142363882	0.9583617	0.982693283
Zbtb26	10	2.613584717	3.110502881	0.496918163	0.95838378	0.982693283
Atm	26	1.247061619	1.159342896	-0.087718724	0.95849489	0.982738541
Ccdc122	22	1.33639079	1.265554356	-0.070836434	0.95881431	0.982997354
Aimp2	20	1.193945683	0.958169786	-0.235775898	0.95900347	0.983122601
Plekhf2	116	1.316248647	1.117475882	-0.198772765	0.95923081	0.983286967
Med4	29	1.906407633	1.878653482	-0.027754151	0.95932384	0.983313636
Igbp1	1	20	19.73684211	-0.263157895	0.95977385	0.983576606
Flnb	27	1.516329307	1.371125236	-0.145204072	0.95978148	0.983576606
Slc35b3	60	1.397429871	1.168197753	-0.229232118	0.95968702	0.983576606
Mat2b	34	1.357655948	1.612415045	0.254759097	0.96011053	0.98384511
Gpc6	2	3.296703297	3.415401703	0.118698406	0.96046625	0.98400347
Gm10638	109	0.904897822	0.984476073	0.079578251	0.96034372	0.98400347
Nck2	91	1.213988788	1.153729915	-0.060258873	0.9604518	0.98400347
Ubac1	49	1.94207663	2.071579749	0.129503118	0.96067026	0.984017754
Zfp292	88	1.113223278	1.069558188	-0.04366509	0.96068136	0.984017754
Ybx3	107	1.035720575	0.946476265	-0.089244311	0.96056057	0.984017754
Atad5	29	1.782098209	1.729212081	-0.052886128	0.96085056	0.984122365
Plxna2	46	1.343066277	1.439408603	0.096342326	0.96092291	0.984127774
Ubap2	20	1.889673696	2.19230175	0.302628054	0.96101543	0.984153845
Panx1	54	1.421836307	1.298563022	-0.123273285	0.96122892	0.984303785
Slc31a1	19	2.197509366	2.025959257	-0.171550109	0.96196501	0.984888859
Nfkbie	36	1.710305479	1.689162693	-0.021142786	0.96200164	0.984888859
Mir7024	4	92.9104099	93.56113215	0.650722244	0.96191801	0.984888859
Hells	65	1.216747069	1.017637733	-0.199109336	0.96255286	0.985384447
Lsm14b	81	1.231314487	1.165174847	-0.066139641	0.96270091	0.985400568
Sdcbp	19	1.686613485	1.651085751	-0.035527734	0.96270291	0.985400568
Dgat2	37	1.791498701	1.547768238	-0.243730463	0.96293794	0.985572392
Oxct1	32	1.221409096	1.118556974	-0.102852123	0.96320006	0.98577191
200014J11R	58	1.298088008	1.079153449	-0.218934559	0.96337691	0.985790318
Lin9	109	1.075000082	1.272061119	0.197061037	0.96341958	0.985790318
Ubxn11	11	0.307319304	0.371057514	0.06373821	0.963315	0.985790318

Senp1	14	1.544443188	1.211204079	-0.33323911	0.96360852	0.985846153
Gltscr2	19	1.911179999	1.539856131	-0.371323867	0.96355015	0.985846153
Med15	16	1.889092774	1.825045539	-0.064047234	0.96403822	0.986217012
Snai1	40	2.157242003	2.002291194	-0.154950809	0.96410865	0.986220307
Kbtbd2	96	1.004614488	0.990415752	-0.014198736	0.96417857	0.986223086
Ogdh	63	1.511922175	1.276968583	-0.234953592	0.96445616	0.98636951
Prune2	11	1.259446727	1.34808627	0.088639543	0.96439511	0.98636951
Elavl1	56	1.052093108	0.951669405	-0.100423703	0.96455194	0.986398718
Mir574	19	1.262070891	1.37263904	0.110568149	0.96476012	0.986474112
Dhrs7	18	1.479117533	1.254708496	-0.224409037	0.96475406	0.986474112
Dicer1	108	1.017830303	1.003047383	-0.01478292	0.96488496	0.986511498
Vps11	23	1.237386175	1.254110357	0.016724182	0.96493114	0.986511498
Sltm	113	1.24932669	1.103525633	-0.145801056	0.96513644	0.98665265
Dnajb2	35	1.946859155	1.655405882	-0.291453273	0.96536423	0.986816767
Ap3b2	43	14.8316029	15.23549903	0.403896129	0.96554409	0.986931879
Tmem219	25	1.257922852	1.22371367	-0.034209181	0.96567623	0.986998188
Pcmtd1	54	1.161905046	1.091217393	-0.070687653	0.96591174	0.987170142
3-Mar	27	1.33552031	1.334785275	-0.000735034	0.96617025	0.987365573
Heatr9	1	76.47058824	76.74418605	0.273597811	0.96629362	0.987422884
Fam160b1	67	1.536786965	1.333376898	-0.203410067	0.96650341	0.987499726
Acad8	8	1.620939409	1.234192358	-0.386747051	0.96648725	0.987499726
Arpc2	53	1.078007914	0.971955338	-0.106052575	0.9668712	0.987600445
Stk3	73	1.149750294	1.186566787	0.036816493	0.96671102	0.987600445
Atp6v0d1	50	1.282434783	1.239149604	-0.043285179	0.96682103	0.987600445
Nupl1	68	0.971022145	0.990308081	0.019285936	0.96684534	0.987600445
Tns3	52	1.085706288	1.049704868	-0.03600142	0.96701087	0.987667269
Lysmd3	54	0.986235592	0.796297954	-0.189937637	0.96707124	0.987667269
Nrm	24	1.065664395	1.119267675	0.05360328	0.9671471	0.987676008
Fryl	68	1.304128815	1.385597909	0.081469094	0.96725017	0.987712527
Smpd2	14	1.353261101	1.244430121	-0.10883098	0.96751614	0.987915378
Chsy1	162	1.248616235	1.077523028	-0.171093207	0.96758627	0.987918244
Plau	2	0.649350649	0.613496933	-0.035853717	0.96776214	0.987918481
Eif3a	67	0.901744822	0.889803886	-0.011940936	0.96778848	0.987918481
Pogk	63	1.929548518	1.546931572	-0.382616946	0.96772622	0.987918481

Dnajc5g	8	86.70153244	88.28812237	1.586589929	0.96810357	0.988171385
Gm6793	47	1.1589191	1.067071722	-0.091847378	0.96854535	0.988484802
)30467D21F	16	88.34733155	88.17050094	-0.17683061	0.96854088	0.988484802
Nelfcd	9	1.620180734	1.327284053	-0.29289668	0.96865331	0.988526235
Gm5	4	92.79308622	93.13426195	0.34117573	0.96880272	0.988609956
Elmo2	82	6.22689195	6.11655758	-0.110334371	0.96909396	0.988809949
Rpl36al	53	1.265732904	1.387889526	0.122156622	0.96940302	0.988809949
Dbf4	64	1.229870521	1.089211173	-0.140659348	0.96937877	0.988809949
Gm17296	108	1.088017942	0.921895991	-0.166121951	0.96927878	0.988809949
Inpp1	39	1.545881424	1.484610041	-0.061271383	0.96925131	0.988809949
B3gnt2	69	1.041931469	1.113891715	0.071960246	0.96940263	0.988809949
Heatr3	4	0.649350649	0.602409639	-0.046941011	0.96969451	0.988943891
Txlna	8	1.300020968	1.020302759	-0.279718208	0.9704752	0.988943891
Rras2	55	1.112063661	1.098326005	-0.013737657	0.97026092	0.988943891
Ifngr1	8	1.70454884	1.698674423	-0.005874418	0.9704294	0.988943891
Vrk1	22	2.191942903	2.360909905	0.168967002	0.96975971	0.988943891
Tnfrsf21	48	1.253212549	1.340563566	0.087351018	0.97053198	0.988943891
Reep4	24	0.841766332	0.735857916	-0.105908416	0.97027397	0.988943891
Tgfa	40	1.325089221	1.398161366	0.073072145	0.97044388	0.988943891
Prcp	34	1.687827624	1.943390723	0.255563098	0.97081384	0.988943891
Trappc6a	17	1.391367981	1.557945763	0.166577782	0.96992518	0.988943891
Tnfrsf14	1	9.677419355	9.523809524	-0.153609831	0.97078763	0.988943891
Arhgap19	28	1.426028338	1.321208038	-0.1048203	0.9705125	0.988943891
Fam227b	20	1.613069374	1.404649372	-0.208420001	0.97012773	0.988943891
.10045C21F	20	1.462899631	1.273706038	-0.189193594	0.97036557	0.988943891
Col18a1	17	38.78959854	38.39330329	-0.396295255	0.96980629	0.988943891
Cyb5d1	4	0.610204082	0.646464646	0.036260565	0.97016249	0.988943891
Msgn1	4	93.32686811	92.95594132	-0.370926787	0.97081483	0.988943891
Dclre1a	1	6.542056075	6.474820144	-0.067235931	0.97071415	0.988943891
Npm3	72	1.412127878	1.213908908	-0.19821897	0.97063506	0.988943891
Ino80d	125	1.425188039	1.29288015	-0.13230789	0.9708911	0.988952936
Nudt14	48	1.790406103	1.669389266	-0.121016838	0.97146282	0.989466611
BC030867	22	1.613373465	1.636121493	0.022748028	0.97186745	0.989810032
Tnfaip1	15	1.31392457	1.238225561	-0.075699009	0.97194165	0.989816906

Pdia3	67	1.229025713	1.161700347	-0.067325367	0.97233357	0.990147313
30401M01I	44	1.254201171	1.248219626	-0.005981545	0.97284026	0.990388375
Ccdc126	11	1.534485763	1.306264395	-0.228221369	0.9727527	0.990388375
Rb1cc1	78	1.081831167	1.146622509	0.064791342	0.97267145	0.990388375
Slc13a5	13	13.63291505	14.54972477	0.916809726	0.97282421	0.990388375
Uba2	53	3.027800169	2.639530326	-0.388269843	0.97299126	0.990473375
Ing2	118	1.158789617	1.261907309	0.103117692	0.97306749	0.990482267
Wdpcp	7	1.18861024	0.932714649	-0.255895592	0.97329843	0.990648625
Micall1	111	1.198351608	1.290575692	0.092224085	0.97338704	0.990670099
Ccp110	20	2.315895222	2.365322665	0.049427443	0.97347511	0.99069102
Aire	6	6.310679612	7.495837007	1.185157395	0.9737412	0.990893095
Ppp6c	51	1.032048374	0.966340246	-0.065708128	0.97386145	0.990946741
Cep76	114	1.180023316	1.216972321	0.036949005	0.97429952	0.991082059
Nop56	57	1.209518879	1.057581368	-0.151937511	0.97421845	0.991082059
Endod1	62	1.799697081	1.445310617	-0.354386464	0.97439968	0.991082059
Nat9	17	1.120554405	0.9860298	-0.134524605	0.97414037	0.991082059
Fam210b	43	1.692696936	1.708386534	0.015689598	0.9743507	0.991082059
Pomt2	23	1.471826765	1.552682169	0.080855404	0.97431326	0.991082059
U2surp	34	0.980873074	0.986692476	0.005819402	0.9745414	0.991157507
Far1	77	0.984555222	1.007067931	0.022512708	0.97470365	0.991253817
Tmx2	10	2.424327104	2.195872684	-0.228454421	0.97498936	0.991256688
Grid1	129	34.99057225	36.31940919	1.328836941	0.97511178	0.991256688
Eif2a	62	1.505321665	1.399212765	-0.106108899	0.97483446	0.991256688
Med19	10	2.424327104	2.195872684	-0.228454421	0.97498936	0.991256688
Copa	2	1.470588235	1.530612245	0.06002401	0.97486657	0.991256688
Ccdc77	52	1.156123241	1.301097934	0.144974692	0.97505407	0.991256688
Alox12e	2	20.02723312	18.51342261	-1.513810502	0.97524511	0.991323547
Hmga1	85	1.132148185	1.158260017	0.026111832	0.97543762	0.991450557
Zfp760	20	1.48100625	1.178288491	-0.302717759	0.97574873	0.991487378
P4hb	94	1.065633344	0.942725843	-0.122907501	0.97555265	0.991487378
30003M21I	6	3.774250441	3.380245272	-0.394005169	0.97564892	0.991487378
Tmem143	8	0.35294313	0.517800865	0.164857735	0.97601439	0.991487378
Depdc5	23	1.744193325	1.630967744	-0.11322558	0.97588978	0.991487378
Ywhag	27	1.209472694	0.925243607	-0.284229086	0.97583986	0.991487378

Mesdc1	82	1.2594501	1.133873836	-0.125576264	0.97595819	0.991487378
Hs2st1	61	0.96040392	0.848792339	-0.111611581	0.97601109	0.991487378
Pyurf	24	1.986819516	1.833708815	-0.1531107	0.97641851	0.991571185
Mir7069	2	88.65384615	84.75732601	-3.896520147	0.9764409	0.991571185
Fxyd5	6	0.974702381	0.782828283	-0.191874098	0.97644768	0.991571185
Gab2	111	1.332679156	1.440450153	0.107770997	0.97643084	0.991571185
Lgals8	14	1.185044946	1.322055138	0.137010192	0.97623822	0.991571185
Pofut2	42	1.651895291	1.212538561	-0.43935673	0.97650233	0.991571185
Kctd6	96	1.014127817	0.871321659	-0.142806159	0.97680554	0.99181044
Dolpp1	48	1.171775136	1.099305423	-0.072469713	0.97695794	0.991896549
Mrpl38	74	1.477869051	1.291171055	-0.186697996	0.97726327	0.991951161
Dcaf4	12	2.025910581	1.146803387	-0.879107195	0.97719604	0.991951161
Plagl1	10	54.70360589	55.02393048	0.320324589	0.97734973	0.991951161
Shmt1	10	1.030893989	0.931375026	-0.099518962	0.97711224	0.991951161
Mfsd2b	4	25.41472416	23.93937339	-1.475350777	0.97730152	0.991951161
Tbc1d12	60	1.264170355	1.230920709	-0.033249646	0.97778075	0.99208204
Ddx59	29	1.042281038	1.038878638	-0.0034024	0.97768848	0.99208204
Ttl	54	1.146533995	1.155989092	0.009455097	0.97761877	0.99208204
Mtmr12	68	1.002309551	1.0048372	0.002527649	0.9776512	0.99208204
Rab17	2	18.15199563	18.30985915	0.157863529	0.97781672	0.99208204
Sertad2	136	1.501609642	1.387100108	-0.114509533	0.97803187	0.99216313
Pigw	97	1.167484651	1.206714172	0.039229521	0.9780285	0.99216313
Slc30a5	27	0.844939469	0.70081988	-0.14411959	0.97838267	0.992450387
Papd5	91	0.944123119	0.882822136	-0.061300983	0.97861627	0.992618729
Ube2e3	129	1.180127172	1.262609693	0.082482521	0.97890509	0.992843051
Vps37a	60	1.567947401	1.572582064	0.004634663	0.9789967	0.992849266
Dusp10	15	1.785438806	1.302068429	-0.483370377	0.97904654	0.992849266
Uqcrh	3	1.228070175	1.255230126	0.02715995	0.97961411	0.993282008
Psmd11	72	1.535277178	1.414215663	-0.121061515	0.97974402	0.993282008
Mepce	8	0.148809524	0.135869565	-0.012939959	0.97960725	0.993282008
10320M18I	28	1.098309663	1.127830614	0.029520952	0.97967811	0.993282008
Tmem50b	58	1.407981004	1.402136778	-0.005844226	0.97990937	0.993304241
Myt1	1	7.692307692	7.575757576	-0.116550117	0.97996114	0.993304241
Zmym4	96	1.551948935	1.463177575	-0.088771361	0.97996903	0.993304241

Jade1	76	17.17985467	16.88180989	-0.298044773	0.98019836	0.99333085
.10059G10F	14	2.134470322	1.592427208	-0.542043113	0.98015967	0.99333085
Tdrd9	6	92.1568752	91.55553963	-0.60133557	0.98010094	0.99333085
Tmtc2	34	1.198709243	1.208684382	0.009975139	0.98054665	0.993420567
Cish	19	2.027029632	2.09156516	0.064535528	0.98052299	0.993420567
Naa35	93	1.199584127	1.260799196	0.06121507	0.98055769	0.993420567
Zfp174	20	1.986170128	1.831144974	-0.155025155	0.98051098	0.993420567
Tlcd1	16	1.629161926	1.424705528	-0.204456398	0.98109058	0.993891829
Tnks	50	1.269821628	1.09229685	-0.177524778	0.98124304	0.993931249
Mcm5	29	1.440246388	1.106525481	-0.333720907	0.98126496	0.993931249
Cox7a2	52	1.143814747	1.064517799	-0.079296948	0.98141752	0.994017163
Mrps31	17	2.57334775	1.960004877	-0.613342873	0.98202692	0.9944425
Ppil2	10	0.57679016	0.540540541	-0.036249619	0.98206663	0.9944425
Rev1	149	1.202778842	1.111752952	-0.09102589	0.98210854	0.9944425
Psmd8	29	1.020280698	1.125242485	0.104961787	0.98195223	0.9944425
Gjd3	27	27.00010686	27.5365279	0.536421037	0.98246381	0.994596342
Snapin	37	1.023883222	1.117362628	0.093479406	0.98244307	0.994596342
Spcs1	49	1.623566148	1.003515788	-0.620050359	0.98244155	0.994596342
Tnfrsf23	4	1.298701299	0.560334003	-0.738367296	0.98298379	0.994854728
Zbtb14	151	1.108265143	1.053184616	-0.055080527	0.98282007	0.994854728
Wdr5	33	1.002457685	0.860759193	-0.141698492	0.98287647	0.994854728
Uxs1	42	1.360364916	1.553407841	0.193042925	0.98299023	0.994854728
Zdhhc23	64	2.467151849	2.237030828	-0.23012102	0.98336967	0.995130839
Zdhhc13	50	1.391991388	1.221719462	-0.170271926	0.98339868	0.995130839
Tmem51	24	2.215057217	2.247626926	0.032569709	0.98361786	0.995283993
Rps6kc1	31	1.285204609	1.196065918	-0.089138692	0.98378443	0.9953839
Spast	60	1.296000695	1.155468255	-0.14053244	0.9838852	0.995417226
D16Ertd472(	65	0.880793484	0.861563212	-0.019230272	0.98402119	0.995486168
Tanc1	67	1.673805483	1.619838468	-0.053967014	0.98422756	0.995557674
Arpc1b	37	1.654839307	1.531674129	-0.123165178	0.98418626	0.995557674
Ice1	51	1.264001079	1.202115312	-0.061885768	0.98433242	0.995595113
Ctdspl2	24	0.987262994	1.012847125	0.025584132	0.98440842	0.995603364
Brk1	41	1.137704627	1.184963718	0.047259091	0.98480244	0.995933223
Nsun4	11	1.384205862	0.83832176	-0.545884102	0.98487727	0.995940259

H2-D1	12	1.56574889	1.69688546	0.13113657	0.98508682	0.996083515
Mrpl13	10	0.929396151	0.79154653	-0.137849621	0.98543621	0.996230861
Tpi1	10	2.445513913	2.124158723	-0.321355191	0.98538049	0.996230861
Lamtor2	20	1.336477468	1.332486913	-0.003990555	0.9853432	0.996230861
Mlf2	68	1.475812506	1.546616044	0.070803538	0.98597915	0.996253485
Rpusd4	12	1.432727928	1.00987561	-0.422852318	0.98583454	0.996253485
Ranbp3	37	1.888695953	1.999429552	0.110733599	0.9858942	0.996253485
Ak2	18	1.715226443	1.334664924	-0.380561519	0.98601893	0.996253485
Anapc4	23	1.376309981	1.47338628	0.097076299	0.98584328	0.996253485
Rps23	30	1.095609363	1.199497186	0.103887822	0.98573215	0.996253485
Med11	18	0.750535621	0.793527419	0.042991798	0.98606962	0.996253485
Nol9	66	1.118027228	0.996442331	-0.121584897	0.98567661	0.996253485
Ddrgk1	7	2.244478693	1.575451342	-0.669027351	0.98592454	0.996253485
Hnrnpdl	149	0.977671969	0.939773899	-0.03789807	0.98614286	0.99625889
Nr1d1	8	1.899007559	2.183560708	0.284553149	0.98637708	0.996313465
Rfk	59	1.021141554	0.992666242	-0.028475312	0.98640057	0.996313465
Fubp1	20	0.504419815	0.623174448	0.118754633	0.98630218	0.996313465
Tshz1	10	1.906676326	1.509953479	-0.396722847	0.98656749	0.996413472
Pdcd6	5	0.208333333	0.224719101	0.016385768	0.98681476	0.996594613
Cdh24	58	1.567495763	1.651945932	0.084450169	0.98717883	0.996893689
Itsn1	87	1.052861075	0.948663263	-0.104197812	0.9875096	0.99695328
Ankfy1	31	1.544034858	1.849299558	0.3052647	0.98745356	0.99695328
Csde1	44	1.520269128	1.652389911	0.132120782	0.98743461	0.99695328
Rest	83	3.90181959	3.806793365	-0.095026225	0.98749896	0.99695328
Trerf1	18	10.33928967	10.55044631	0.211156641	0.98760882	0.996984854
Tk2	21	1.787965981	1.564869563	-0.223096419	0.98974325	0.99716295
Sil1	25	1.458882947	1.389690365	-0.069192582	0.989119	0.99716295
Ttc39aos1	13	1.786191478	2.041046004	0.254854526	0.98858566	0.99716295
Mthfd1	50	2.50808387	2.602044997	0.093961127	0.98790145	0.99716295
Bend3	95	0.919697736	0.942826094	0.023128359	0.98898917	0.99716295
Zcchc7	36	1.267450323	1.375919545	0.108469223	0.98946174	0.99716295
Cdca4	73	1.330546844	1.029916577	-0.300630267	0.98842882	0.99716295
Hnf4a	6	92.30537729	92.94815891	0.642781621	0.98914577	0.99716295
Ube2q1	58	1.475703552	1.502627113	0.026923561	0.98932183	0.99716295

Gm11346	2	88.63636364	88.54540359	-0.090960049	0.98945483	0.99716295
Ccdc101	24	1.377693163	1.326383508	-0.051309655	0.98813589	0.99716295
Noc2l	77	1.081843831	1.028670194	-0.053173638	0.98929107	0.99716295
Nkain2	15	2.529375962	2.337264052	-0.192111911	0.98954796	0.99716295
Optn	23	1.114394277	1.384789117	0.27039484	0.98852577	0.99716295
Sapcd2	6	13.78590892	15.34404811	1.558139188	0.98901351	0.99716295
Ccdc97	7	2.388898109	1.784868906	-0.604029203	0.98809903	0.99716295
30013C23F	30	2.212250989	2.192210789	-0.020040199	0.98930172	0.99716295
Rnf31	56	1.538080281	1.782428222	0.244347941	0.98920849	0.99716295
Exosc10	37	1.091740174	1.098694034	0.00695386	0.98915501	0.99716295
Morn1	29	1.084458763	1.110955566	0.026496803	0.98895266	0.99716295
Smg8	9	1.206570975	0.930839629	-0.275731346	0.98882373	0.99716295
Pex16	17	2.153470424	1.974443562	-0.179026862	0.98966983	0.99716295
Dera	16	1.081151766	0.943569008	-0.137582758	0.98962343	0.99716295
Hist1h1c	11	0.885054682	1.016762077	0.131707395	0.98979108	0.99716295
Gm5105	6	96.75981444	97.03598719	0.276172748	0.98853196	0.99716295
Adprm	12	1.333952942	1.54360267	0.209649728	0.98806034	0.99716295
Patz1	69	1.078107712	1.129692462	0.051584751	0.9891548	0.99716295
00015O10F	1	96.03174603	96	-0.031746032	0.98974148	0.99716295
Pdzd7	7	61.62217845	61.59386197	-0.028316486	0.98831863	0.99716295
Prune	11	1.261663015	1.644909031	0.383246016	0.98982387	0.99716295
Commd10	25	1.127386312	1.076043119	-0.051343193	0.98994126	0.997212742
Mre11a	16	1.229204406	1.229903216	0.000698809	0.99002192	0.997225542
Ing3	36	1.189650978	1.301571498	0.11192052	0.99010801	0.997243801
Rbm25	20	1.027111727	0.801611107	-0.22550062	0.9901929	0.997260847
Eftud2	36	1.315419319	1.058650373	-0.256768946	0.99043421	0.997354771
Fam98b	12	0.367835144	0.407524892	0.039689748	0.99049006	0.997354771
10107E04F	35	1.398853412	1.603266492	0.204413079	0.99046251	0.997354771
Ilf2	14	1.57826027	1.834704584	0.256444314	0.99074665	0.997544693
Acvr1b	110	1.294812067	1.245772455	-0.049039612	0.9912687	0.997828053
33405D12F	30	1.205022573	1.468010662	0.262988089	0.9912258	0.997828053
Cggbp1	88	1.363230809	1.398338826	0.035108017	0.99130008	0.997828053
Tada1	41	1.239850395	1.195146396	-0.044703999	0.9912786	0.997828053
Malat1	16	0.875184549	0.919938478	0.04475393	0.99223404	0.997975325

Zyg11b	37	1.743853848	1.884182625	0.140328776	0.99174459	0.997975325
Riok2	16	1.548814322	1.223704424	-0.325109898	0.99199559	0.997975325
Klhl4	3	43.04809323	43.59466489	0.546571666	0.99176692	0.997975325
Aven	107	1.522277683	1.420247274	-0.102030409	0.99223287	0.997975325
Mcm6	39	1.209048251	1.304879455	0.095831204	0.99182567	0.997975325
Rngtt	54	2.085434729	1.884533702	-0.200901027	0.9922625	0.997975325
Ppp1r3e	35	1.247766552	1.065953118	-0.181813434	0.99207082	0.997975325
Bag2	55	1.286719334	1.163324982	-0.123394353	0.99174059	0.997975325
Gga2	14	1.628312256	1.419787728	-0.208524528	0.99189559	0.997975325
Pipox	2	8.600697472	8.65248227	0.051784798	0.99205658	0.997975325
Arid3a	87	1.800685396	1.598427153	-0.202258243	0.99204885	0.997975325
Agap3	119	1.408469068	1.124353489	-0.284115579	0.99240891	0.998054169
Ywhaq	56	1.119863514	1.11920255	-0.000660963	0.99248899	0.998066299
Cuedc1	99	19.29084877	19.08558986	-0.205258908	0.99266008	0.998135542
Vwa8	52	1.765879152	1.661089409	-0.104789742	0.99269389	0.998135542
Hdac6	12	20.55590766	21.64699178	1.091084118	0.99299066	0.998365536
Nt5m	81	1.286744756	1.209775023	-0.076969733	0.99311596	0.998423098
Selo	73	1.115183893	0.961572293	-0.153611599	0.99343555	0.998675976
Zfp692	18	1.104904724	0.994889298	-0.110015425	0.99370795	0.998695128
Fdx1	49	0.838939138	1.124997157	0.286058019	0.9936244	0.998695128
Hinfp	25	1.392430558	1.452456195	0.060025637	0.99363035	0.998695128
Hmga2-ps1	8	1.124935319	1.420369008	0.295433689	0.99372683	0.998695128
Tmem238	91	1.176571415	1.064754166	-0.111817249	0.99385416	0.998707191
Thap3	35	1.656902405	1.278970042	-0.377932363	0.99397695	0.998707191
Fads1	61	1.142541559	1.083672148	-0.058869411	0.99401108	0.998707191
Mecr	47	1.78619515	1.716831495	-0.069363655	0.99395049	0.998707191
210406O10F	22	1.260239915	1.262601221	0.002361306	0.99473989	0.999029027
H2-M3	13	3.073495932	3.369231532	0.2957356	0.99468649	0.999029027
Tmem55b	68	1.148450629	1.096768623	-0.051682006	0.99462102	0.999029027
Nupl2	23	1.209897622	1.129884029	-0.080013593	0.99442731	0.999029027
Dnajb14	85	1.13648463	1.078819419	-0.05766521	0.99447412	0.999029027
Rara	130	2.377144262	2.304159653	-0.072984609	0.99472878	0.999029027
Thumpd2	43	1.591100773	1.466168767	-0.124932007	0.99503616	0.999094598
532428C04F	46	1.529008965	1.530399044	0.001390079	0.99507752	0.999094598

Cenpu	38	1.8653075	1.672771749	-0.19253575	0.99493005	0.999094598
Vps25	17	1.183002407	1.476480175	0.293477768	0.99503983	0.999094598
Kcnv1	1	74.46808511	74.43609023	-0.031994881	0.99515758	0.999106615
Chst8	44	14.55302469	15.49061446	0.937589771	0.99549609	0.999241373
Tcf3	60	0.93708361	0.871053565	-0.066030045	0.99545659	0.999241373
Slc39a6	55	0.826510074	0.85116894	0.024658867	0.99539109	0.999241373
Scyl1	64	1.289078065	1.33089604	0.041817975	0.99577887	0.999251804
Spryd4	10	1.078633779	1.346768771	0.268134992	0.9957467	0.999251804
Nipsnap3a	6	49.14734544	48.45370263	-0.693642811	0.99569327	0.999251804
Ctns	9	1.612649434	1.320012187	-0.292637247	0.99563679	0.999251804
Wdr3	29	1.102620337	1.033483803	-0.069136534	0.99619013	0.999383116
Rbm19	30	1.868383957	1.575652837	-0.29273112	0.99609458	0.999383116
Mrps10	15	1.078442003	0.955706487	-0.122735516	0.99601035	0.999383116
Nap1l4	73	1.024974746	0.963812252	-0.061162494	0.99625025	0.999383116
Stat5a	25	3.771423938	3.523306172	-0.248117766	0.99624172	0.999383116
Prepl	25	1.267082622	1.724875081	0.457792459	0.9966205	0.999481221
Lcmt2	22	0.627446011	0.781223556	0.153777545	0.99661626	0.999481221
Zfp407	92	1.630398937	1.525530033	-0.104868904	0.9965755	0.999481221
Mcm3	21	1.41209576	1.197363298	-0.214732462	0.99643106	0.999481221
Mex3b	97	1.36867078	1.307389436	-0.061281344	0.99674511	0.999490877
Sptlc1	57	3.448399076	2.877156777	-0.571242299	0.99677888	0.999490877
Nsmce2	9	0.947316954	0.836466489	-0.110850465	0.99683447	0.999490877
Elk3	25	1.116130174	1.050220389	-0.065909785	0.99693558	0.999523962
Ap4b1	19	1.014480834	1.066922854	0.052442019	0.99713475	0.999655343
Rnf126	127	1.243461687	1.114806648	-0.128655039	0.99727851	0.999665866
Ssr3	13	0.682251975	0.500501772	-0.181750203	0.99728149	0.999665866
Vwa9	30	4.882518713	5.04479076	0.162272047	0.99738693	0.999703267
Clasrp	49	1.493700325	1.172628469	-0.321071856	0.99796594	0.99974687
Ror1	39	1.290852725	1.026664782	-0.264187943	0.99795495	0.99974687
Trim7	13	12.47869261	12.83316999	0.354477378	0.99809129	0.99974687
Dram2	51	1.304514468	1.085384934	-0.219129535	0.9980425	0.99974687
Pigc	28	1.587018902	1.698145527	0.111126625	0.99817987	0.99974687
310442N19F	2	71.21710526	70.69642686	-0.520678402	0.99776892	0.99974687
Plekhb2	24	1.181887687	1.311761634	0.129873947	0.9977595	0.99974687

Mir5133	32	1.767029175	1.588494827	-0.178534347	0.9981042	0.99974687
Pja2	24	1.197043183	0.953103203	-0.243939979	0.99817193	0.99974687
Kcna7	28	7.254607865	8.215342154	0.960734288	0.99756899	0.99974687
Slc35f5	53	1.737396164	1.645673286	-0.091722878	0.99760965	0.99974687
Ints6	77	1.20783524	1.022859296	-0.184975944	0.99825684	0.999755727
Szt2	6	1.120408926	1.032908203	-0.087500722	0.9983267	0.999757456
Cpt2	25	1.210412896	1.49729454	0.286881644	0.99851424	0.999877022
C1rl	1	100	100	0	1	1
Lrrc29	2	0	0	0	1	1
Srcap	4	0	0	0	1	1
Gm12238	1	100	100	0	1	1
Spa17	2	0	0	0	1	1
Zfp764	10	1.659327395	1.238120316	-0.421207079	0.99905386	1
Atp5a1	47	1.152277261	1.340889363	0.188612102	0.99881905	1
Zfp663	1	100	100	0	1	1
Mphosph8	21	0.84178989	1.069819084	0.228029194	0.99979389	1
Kirrel	16	1.312351491	0.878079173	-0.434272317	0.99900251	1
Neu3	3	0	0	0	1	1
Hist2h3c2	2	0	0	0	1	1
Mir125b-1	1	0	0	0	1	1
Trit1	4	0	0	0	1	1
Pogz	37	1.021458177	0.81208787	-0.209370306	0.99947011	1
Aftph	63	0.954580169	0.972531806	0.017951636	0.99891783	1
[illegible]10008N11[illegible]	1	100	100	0	1	1
Capg	4	0	0	0	1	1
Cxxc1	65	1.161647237	1.043552621	-0.118094617	0.99947288	1
Mapre1	36	0.904330375	0.769735051	-0.134595323	0.99919333	1
