# Supplementary material for: Bone morphogenetic protein 7 promotes resistance to immunotherapy
Source: Nat Commun. 2020 Sep 24;11:4840. doi: 10.1038/s41467-020-18617-z (PMC7519103; doi:10.1038/s41467-020-18617-z)
Supplement: Supplementary file 6 — Reporting Summary [file 41467_2020_18617_MOESM6_ESM.pdf]

nature research

Corresponding author(s): Maria Angelica Cortez

Last updated by author(s): 08/05/2020

Reporting Summary

Nature Research wishes to improve the reproducibility of the work that we publish. This form provides structure for consistency and transparency in reporting. For further information on Nature Research policies, see Authors & Referees and the Editorial Policy Checklist .

Statistics

For all statistical analyses, confirm that the following items are present in the figure legend, table legend, main text, or Methods section.

n/a	Confirmed	
☐	☒	The exact sample size (*n*) for each experimental group/condition, given as a discrete number and unit of measurement
☐	☒	A statement on whether measurements were taken from distinct samples or whether the same sample was measured repeatedly
☐	☒	The statistical test(s) used AND whether they are one- or two-sided<br>*Only common tests should be described solely by name; describe more complex techniques in the Methods section.*
☐	☒	A description of all covariates tested
☐	☒	A description of any assumptions or corrections, such as tests of normality and adjustment for multiple comparisons
☐	☒	A full description of the statistical parameters including central tendency (e.g. means) or other basic estimates (e.g. regression coefficient) AND variation (e.g. standard deviation) or associated estimates of uncertainty (e.g. confidence intervals)
☐	☒	For null hypothesis testing, the test statistic (e.g. *F*, *t*, *r*) with confidence intervals, effect sizes, degrees of freedom and *P* value noted<br>*Give P values as exact values whenever suitable.*
☒	☐	For Bayesian analysis, information on the choice of priors and Markov chain Monte Carlo settings
☐	☒	For hierarchical and complex designs, identification of the appropriate level for tests and full reporting of outcomes
☐	☒	Estimates of effect sizes (e.g. Cohen's *d*, Pearson's *r*), indicating how they were calculated

Our web collection on statistics for biologists contains articles on many of the points above.

Software and code

Policy information about availability of computer code

Data collection	Nanostring data were collected with the NanoStringNorm package. Immunofluorescence slides were examined with a fluorescence microscope (Leica, Buffalo Grove, IL), and images were captured by a charge-coupled device camera and imported into the Advanced Spot Image analysis software package.IHC slides were examined with a Leica DMI6000B microscope (Leica, Buffalo Grove, IL), and images were captured by a charge-coupled device camera and imported into the Advanced Spot Image analysis software package
Data analysis	Prism 8.0 software (GraphPad) and excell were used for statistical analyses. R (version 3.5.1) software was used for Nanostring and RPPA analysis. Differential expression analysis for Nanostring was done using the moderated t statistic from the LIMMA package. Trim Galore! (version 0.4.1), Bismark (version v0.16.1) and Bowtie (version 1.1.2) were used for RRBS mapping. bismark_methylation_extractor script from Bismark and an in-house Perl script were used for RRBS methylation calling. Differential methylation on CpG sites was statistically assessed by R/Bioconductor package methylKit (version 0.9.5). Heatmap for RRBS study was plotted by heatmap.2 function in R 9version 3.5.1). The significance of differential methylation on gene level was calculated using Stouffer's zscore method by combining all the qualified CpG sites inside each gene's promoter region (defined as -1000bp to +500 of TSS), and was corrected to FDR by Benjamini & Hochberg (BH) methodCorrelation analysis for mRNA data for TCGA Lung Adenocarcinoma cohort was performed in R (version 3.5.1; http:///www.r-project.org/). IHC images were quantified using Fiji software (http://fiji.sc). FlowJo (version 10) was used for Flow cytometer analysis.

For manuscripts utilizing custom algorithms or software that are central to the research but not yet described in published literature, software must be made available to editors/reviewers. We strongly encourage code deposition in a community repository (e.g. GitHub). See the Nature Research guidelines for submitting code & software for further information.

Data

Policy information about availability of data

All manuscripts must include a data availability statement. This statement should provide the following information, where applicable:

- Accession codes, unique identifiers, or web links for publicly available datasets
- A list of figures that have associated raw data
- A description of any restrictions on data availability

All data generated or analyzed during this study are included in this published article (and its supplementary information files). Unique material requests should be directed to the corresponding author. Requests are reviewed by MD Anderson Cancer Center to verify whether the request is subject to any intellectual property or confidentiality obligations. Any material that can be shared will be released via a Material Transfer Agreement. GSE50081 raw data can be accessed at The Gene Expression Omnibus (GEO) database (https://www.ncbi.nlm.nih.gov/geo/). Clinical information for patients with lung adenocarcinoma was retrieved from the article “An Integrated TCGA Pan-Cancer Clinical Data Resource to Drive High-Quality Survival Outcome Analytics”, Cell. Volume 173 (https://www.sciencedirect.com/science/article/pii/S0092867418302290?via%3Dihub), but smoking status. The information regarding smoking status of these was retrieved from cBioPortal for Cancer Genomics (http://www.cbioportal.org/)(Ref: Cerami E et al, The cBio Cancer Genomics Portal: An Open Platform for Exploring Multidimensional Cancer Genomics Data, Cancer Discov. 2012 May;2(5):401-4. doi: 10.1158/2159-8290.CD-12-0095). Gene expression for BMP7 was downloaded as fragments per kilobase millions (FPKM) quantification mRNA-seq data from the Genomic Data Commons Data Portal (https://portal.gdc.cancer.gov/). The Reduced representation bisulfite sequencing (RRBS) data have been deposited in the GEO database at https://www.ncbi.nlm.nih.gov/geo/ under the GEO accession number GSE154993.

Field-specific reporting

Please select the one below that is the best fit for your research. If you are not sure, read the appropriate sections before making your selection.

☒ Life sciences ☐ Behavioural & social sciences ☐ Ecological, evolutionary & environmental sciences

For a reference copy of the document with all sections, see nature.com/documents/nr-reporting-summary-flat.pdf

Life sciences study design

All studies must disclose on these points even when the disclosure is negative.

Sample size

No sample size calculation was performed. Number of experimental animals per group was chosen based on previous experiments using similar experimental approaches.

Data exclusions

No data were excluded from the analysis.

Replication

Data was collected from two independent experiments as stated in Figure legends and all attempts were successful.

Randomization

Mice were randomized in four groups before we start in vivo experiments. Patients were selected based on exclusion/inclusion criteria. Inclusion Criteria: Patients must have histological confirmation of metastatic cancer with at least one metastatic or primary lesion in the liver, lung, or adrenal gland. Patients who have completed previous systemic therapies 5 drug half-lives or 4-weeks prior to enrollment on study, whichever is shorter. Note: patients with anaplastic thyroid will be waived from this inclusion criteria given the rapid trajectory of their disease. All patients must have at least one metastatic or primary lesion within the lung or liver located in an anatomical location amenable to SBRT treatment with 50 Gy in 4 fractions, or if not, with either a lung, liver, or adrenal lesion treatable to 60 Gy in 10 fractions.

Repeat radiation in fields previously radiated will be allowed at the discretion of the treating physician. Age >/= 18 years ECOG performance status </=2 (Karnofsky >60%). Patients must have normal organ and marrow function as defined below: * Total bilirubin </= 2.0 mg/dL. (Does NOT apply to patients with Gilbert's Syndrome) * Aminotransferase (AST) Serum Glutamic Oxaloacetic Transaminase (SGOT)/ Alanine Aminotransferase (ALT) Serum Glutamic-Pyruvic Transaminase (SGPT) <2.5 X institutional upper limit of normal (patients with liver involvement will be allowed </= 5.0 X institutional upper normal limit) *WBC >/= 2500/uL, ANC >/= 1000/uL *Platelets >/= 75K *Hemoglobin >/= 9g/dL *Creatinine </= 2.0 x ULN Patients must be willing and able to review, understand, and provide written consent before starting therapy. Patients with brain metastasis will be included as long as they are free of neurologic symptoms related to metastatic brain lesions and who do not require or receive systemic corticosteroid therapy in the 14 days prior to beginning ipilimumab therapy. Patients that have previously progressed on immunotherapy such as ipilimumab will be eligible.

Exclusion Criteria: Serious autoimmune disease at the discretion of the treating attending: Patients with a history of active serious inflammatory bowel disease (including Crohn's disease and ulcerative colitis) and autoimmune disorders such as rheumatoid arthritis, systemic progressive sclerosis [scleroderma], Systemic Lupus Erythematosus or autoimmune vasculitis [e.g., Wegener's Granulomatosis] are excluded from this study. Active diverticulitis, intra-abdominal abscess, Gastrointestinal (GI) obstruction, abdominal carcinomatosis or other known risk factors for bowel perforation.Any underlying medical or psychiatric condition, which in the opinion of the Investigator, will make the administration of study drug hazardous or obscure the interpretation of Adverse Events: (AE's) e.g. a condition associated with frequent diarrhea or chronic skin conditions, recent surgery or colonic biopsy from which the patient has not recovered, or partial endocrine organ deficiencies. Uncontrolled intercurrent illness including, but not limited to, ongoing or active infection, history of congestive heart failure, unstable angina pectoris, cardiac arrhythmia, or psychiatric illness/social situations that would limit compliance with study requirements. Known active HIV, Hepatitis B, or Hepatitis C that has not been documented to be cured. Any non-oncology vaccine therapy used for prevention of infectious diseases (for up to one month prior to or after any dose of ipilimumab). Concomitant therapy with any of the following: IL-2, interferon or other non-study immunotherapy regimens; cytotoxic chemotherapy; immunosuppressive agents; other investigational therapies; or chronic use of systemic corticosteroids while receiving ipilimumab (as long as steroid replacement is significantly greater than what is required for physiologic replacement, i.e. in hypothyroidism). Pregnant women are excluded from this study. Women of child-bearing potential and men must agree to use contraception prior to study entry and for the duration of study participation. Acceptable

	forms of birth control include: Birth control pills plus a barrier method, such as a condom or diaphragm, Intrauterine devices (IUD) plus a barrier method, Implantable or injectable birth control (such as NorplantR or epo-ProveraR) started at least 3 months before joining the study, plus a barrier method, or Double-barrier method, such as a condom when used in combination with a diaphragm. Should a woman become pregnant or suspect she is pregnant while participating in this study, she should inform her treating physician.History of or current immunodeficiency disease or prior treatment compromising immune function at the discretion of the treating physician. Prior allogeneic stem cell transplantation.
Blinding	Investigators were blinded during data collection.

Reporting for specific materials, systems and methods

We require information from authors about some types of materials, experimental systems and methods used in many studies. Here, indicate whether each material, system or method listed is relevant to your study. If you are not sure if a list item applies to your research, read the appropriate section before selecting a response.

Materials & experimental systems

n/a	Involved in the study
☐	☒ Antibodies
☐	☒ Eukaryotic cell lines
☒	☐ Palaeontology
☐	☒ Animals and other organisms
☐	☒ Human research participants
☐	☒ Clinical data

Methods

n/a	Involved in the study
☒	☐ ChIP-seq
☐	☒ Flow cytometry
☒	☐ MRI-based neuroimaging

Antibodies

Antibodies used	BMP7 (Santa Cruz Biotechnology, Catalog#sc-53917), MAPK14 (Thermo Fisher Scientific, Catalog #PA5-17713), SMAD1(Thermo Fisher Scientific, Catalog #38-5400), anti-Phospho-SMAD1/SMAD5 (Ser463, Ser465) (Thermo Scientific–Life Technologies, Catalog #MA5-15124), anti-mannose receptor (CD206) (Abcam, Catalog #ab64693), or anti-CD4 (Bioss, Catalog #bs-0647R).BMP7 (Abcam, Catalog #ab56023), Phospho-Smad1 (Ser463/465)/ Smad5 (Ser463/465)/ Smad9 (Ser465/467) (Cell Signaling Technologies, Catalog#13820), p38 MAPK (Cell Signaling Technologies, Catalog #8690), Vinculin (Cell Signaling Technologies, Catalog #13901), β-Actin (Cell Signaling Technologies, Catalog # 3700). p38α MAPK (L53F8) (Cell Signaling, Catalog #9228) and Phospho-Smad1 (Ser463/465), Smad5 (Ser463/465), and Smad9 (Ser465/467) (D5B10) (Cell Signaling, Catalog #13820).anti-rabbit IgG (H+L), F(ab')2 Fragment (Alexa Fluor 488 Conjugate) (Cell Signaling, Catalog#4412), or Anti-mouse IgG (H +L), F(ab')2 Fragment (Alexa Fluor 488 Conjugate) (Cell Signaling, Catalog #4408). anti-CD16/CD32, fluorochrome-conjugated anti-CD3 (Cat #100353), anti-CD4 (Cat #100406), anti-CD8 (Cat #100734), anti-CD45 (Cat #103126), anti-CD11b (Cat #101226), anti-CD11c (Cat #117310), anti-F4/80 (Cat #123108), and anti-CD206 (Cat# 141716). InVivoMAb anti-mouse CTLA-4 (CD152), Clone 9D9, Catalog # BE0164. InVivoMAb anti-mouse PD-1 (CD279). Clone RMP1-14. Catalog # BE0146. LEAF purified anti-mouse CD28 antibody (Biolegend, Catalog# 102115, Clone 37.51). LEAF purified anti-mouse CD3ε antibody (Biolegend, Catalog# 100301, Clone 145-2C11).
Validation	BMP7 (Santa Cruz Biotechnology, Catalog#sc-53917, (4E7)). It is validated for Enzyme Linked Immunosorbent Assay, Western Blotting. Recommended for detection of BMP-7 of mouse, rat and human origin by WB, IP and ELISAPMID: # 25605802 Gustafson, B. et al. 2015. Diabetes. 64: 1670-81. PMID: # 22916288 Morone S. et al. 2012. PLoS One. 7(8): e43649. PMID: # 21317922 Fiaschetti, G. et al. 2011. Oncogene. 30: 2823-2835. PMID: # 32273773 Clin Cosmet Investig Dent. 12: 79-85.<br>MAPK14 (Thermo Fisher Scientific, Catalog #PA5-17713). This Antibody was verified by Knockdown to ensure that the antibody binds to the antigen stated. Recommended for detection of MAPK14 in Human, Mouse, Non-human primate, Rat. No references so far.<br>SMAD1 (Thermo Fisher Scientific, Catalog #38-5400), validation not available. Verified by IHC, western blotting. Chip assay and IF by 18 publications. Recommended for detection of SMAD1 of Human, Mouse, Non-human primate. References for IHC is PMID: 24705250. Recurrent somatic mutations in ACVR1 in pediatric midline high-grade astrocytoma. Nature Genetics. 2014 May;46 (5):462-6. doi: 10.1038/ng.2950. Epub 2014 Apr 6.<br>anti-Phospho-SMAD1/SMAD5 (Ser463, Ser465) (Thermo Scientific–Life Technologies, Catalog #MA5-15124). Validated by IF, western blotting, and flow cytometer. Recommended for detection of anti-Phospho-SMAD1/SMAD5 (Ser463, Ser465) of Human, Mouse, Rat. No references available.<br>Anti-mannose receptor (CD206) (Abcam, Catalog #ab64693). Validated by IHC and IF. Reacts with: Mouse, Rat, Human. Suitable for: IHC-P, WB, Flow Cyt, ICC/IF. Ab64693 has been referenced in 238 publications. Some publications for reference as follows. Zhang Q et al. Apoptotic SKOV3 cells stimulate M0 macrophages to differentiate into M2 macrophages and promote the proliferation and migration of ovarian cancer cells by activating the ERK signaling pathway. Int J Mol Med 45:10-22 (2020).PubMed: 31746376Zhao SJ et al. Macrophage MSR1 promotes BMSC osteogenic differentiation and M2-like polarization by activating PI3K/AKT/GSK3ß/ß-catenin pathway. Theranostics 10:17-35 (2020).PubMed: 31903103Orgaz JL et al. Myosin II Reactivation and Cytoskeletal Remodeling as a Hallmark and a Vulnerability in Melanoma Therapy Resistance. Cancer Cell 37:85-103.e9 (2020).PubMed: 31935375Zhou J et al. LncGBP9/miR-34a axis drives macrophages toward a phenotype conducive for spinal cord injury repair via STAT1/STAT6 and SOCS3. J Neuroinflammation 17:134 (2020).PubMed: 32345320Yang HC et al. C-C chemokine receptor type 2-overexpressing exosomes alleviated experimental post-stroke cognitive impairment by enhancing microglia/macrophage M2 polarization. World J Stem Cells 12:152-167 (2020).<br>anti-CD4 (Bioss, Catalog #bs-0647R). CD4 Polyclonal Antibody is validated for WB, IHC-P, FCM and IF(IHC-P) in human, mouse and rat samples. The serum is batch tested, and then after purification we validate again in the applications already mentioned.

Citations: Smit, Francis Edwin, et al. "Does prolonged post-mortem cold ischemic harvesting time influence cryopreserved pulmonary homograft tissue integrity?." Cell and Tissue Banking: 1-14. Jaime-Ramirez, Alena C., et al. "Reolysin and Histone Deacetylase Inhibition in the Treatment of Head and Neck Squamous Cell Carcinoma." Molecular Therapy-Oncolytics 5 (2017): 87-96. IHC-PMOUSE. Jaime-Ramirez et al. Reolysin and Histone Deacetylase Inhibition in the Treatment of Head and Neck Squamous Cell Carcinoma. (2017) Mol.Ther.Oncolytics. 5:87-96. IHC-PMOUSE. Xiong Y et al. Functions of T-cell subsets and their related cytokines in the pathological processes of autoimmune encephalomyelitic mice.(2018) Int J Clin Exp Pathol;11 (10):4817-4826.FCM-IHC-MOUSE. Hu X et al. Atmospheric H2S triggers immune damage by activating the TLR-7/MyD88/NF-κB pathway and NLRP3 inflammasome in broiler thymus. Chemosphere. 2019 Jul 22;237:124427. WB-IF-CHICKEN. Sun J1 et al. Clinical effects of lentinan combined with budesonide inhalation in treating acute exacerbation of chronic obstructive pulmonary disease under mechanical ventilation. Exp Ther Med. 2019 Mar;17(3):1503-1508. FCM-HUMAN

BMP7 (Abcam, Catalog#ab56023). Validated by WB, IHC-P, ICC/IF. Reacts with: Mouse, Rat, Human. The ab56023 has been referenced in 28 publications. Some publications for reference as follows. Sun R et al. Expression of BMP7 in cervical cancer and inhibition of epithelial-mesenchymal transition by BMP7 knockdown in HeLa cells. Int J Mol Med 45:1417-1424 (2020).PubMed: 32323730Cheng J et al. Catalpol Promotes the Proliferation and Differentiation of Osteoblasts Induced by High Glucose by Inhibiting KDM7A. Diabetes Metab Syndr Obes 13:705-712 (2020).PubMed: 32214833Zhang X et al. Branched Chain Amino Acids Protects Rat Mesangial Cells from High Glucose by Modulating TGF-ß1 and BMP-7. Diabetes Metab Syndr Obes 12:2433-2440 (2019).PubMed: 31819569Li et al. Muscle injury promotes heterotopic ossification by stimulating local bone morphogenetic protein-7 production. J Orthop Translat 18:142-153 (2019).PubMed: 31508317Chiang ER et al. Use of Allogeneic Hypoxic Mesenchymal Stem Cells For Treating Disc Degeneration in Rabbits. J Orthop Res 37:1440-1450 (2019).PubMed: 31062869Miao N et al. Loss of Fam20c causes defects in the acinar and duct structure of salivary glands in mice. Int J Mol Med 43:2103-2117 (2019).PubMed: 30864688Meng Q et al. Myofibroblast-Specific TGFß Receptor II Signaling in the Fibrotic Response to Cardiac Myosin Binding Protein C-Induced Cardiomyopathy. Circ Res 123:1285-1297 (2018).PubMed: 30566042Kim Y et al. Evaluation of Mesenchymal Stem Cell Sheets Overexpressing BMP-7 in Canine Critical-Sized Bone Defects. Int J Mol Sci 19:N/A (2018).PubMed: 30018197Yao H et al. BMP7 antagonizes proliferative vitreoretinopathy through retinal pigment epithelial fibrosis in vivo and in vitro. FASEB J N/A:fj201800858RR (2018).PubMed: 30383450

Phospho-Smad1 (Ser463/465)/ Smad5 (Ser463/465)/ Smad9 (Ser465/467) (Cell Signaling Technologies, Catalog#13820). Species Reactivity: Human, Mouse, Rat. Applications for Immunoprecipitation (IP) Western Blotting (WB). 138 citations have been found for this product. Some publications as follows. In Regenerative Therapy on 1 June 2020 by Cai, C., Wang, J., et al. Integration of Nodal and BMP Signaling by Mutual Signaling Effector Antagonism. In Cell Reports on 7 April 2020 by Soh, G. H., Pomreinke, A. P., et al. BMP Signaling Gradient Scaling in the Zebrafish Pectoral Fin. In Cell Reports on 24 March 2020 by Mateus, R., Holtzer, L., et al. BMP-SMAD1/5 Signaling Regulates Retinal Vascular Development. In Biomolecules on 23 March 2020 by Benn, A., Alonso, F., et al. Mutant ACVR1 Arrests Glial Cell Differentiation to Drive Tumorigenesis in Pediatric Gliomas. In Cancer Cell on 16 March 2020 by Fortin, J., Tian, R., et al. Dullard-mediated Smad1/5/8 inhibition controls mouse cardiac neural crest cells condensation and outflow tract septation. In eLife on 27 February 2020 by Darrigrand, J. F., Valente, M., et al. BMP4 induces asymmetric cell division in human glioma stem-like cells.In Oncology Letters on 1 February 2020 by Koguchi, M., Nakahara, Y., et al. BMP4 gene therapy enhances insulin sensitivity but not adipose tissue browning in obese mice. In Molecular Metabolism on 1 February 2020 by Hoffmann, J. M., Grünberg, J. R., et al. BMP9 mediates the anticancer activity of evodiamine through HIF-1α/p53 in human colon cancer cells. In Oncology Reports on 1 February 2020 by Li, F. S., Huang, J., et al.

p38 MAPK (Cell Signaling Technologies, Catalog #8690). Validated by IHC and western blotting. Species Reactivity: Human, Mouse, Rat, Hamster, Monkey, Bovine, Pig. Product Citations: 708. Some publications as follows. Curcumin inhibited the growth and invasion of human monocytic leukaemia SHI-1 cells in vivo by altering MAPK and MMP signalling. In Pharmaceutical Biology on 1 December 2020 by Zhu, G., Shen, Q., et al. Protective Effects of Punicalagin on Osteoporosis by Inhibiting Osteoclastogenesis and Inflammation via the NF-κB and MAPK Pathways. In Frontiers in Pharmacology on 2 June 2020 by Wang, W., Bai, J., et al. Upregulated NTF4 in colorectal cancer promotes tumor development via regulating autophagy. In International Journal of Oncology on 1 June 2020 by Yang, Z., Chen, Y., et al. Cilostazol alleviate nicotine induced cardiomyocytes hypertrophy through modulation of autophagy by CTSB/ROS/p38MAPK/JNK feedback loop. In International Journal of Biological Sciences on 14 May 2020 by Wang, S. Y., Ni, X., et al. Identification and functional activity of matrix-remodeling associated 5 (MXRA5) in benign hyperplastic prostate. In Aging (Albany NY) on 11 May 2020 by Xiao, H., Jiang, Y., et al. QKI deficiency leads to osteoporosis by promoting RANKL-induced osteoclastogenesis and disrupting bone metabolism. In Cell Death & Disease on 7 May 2020 by Du, T., Yan, Z., et al. Bicyclol Attenuates Acute Liver Injury by Activating Autophagy, Anti-Oxidative and Anti-Inflammatory Capabilities in Mice. In Frontiers in Pharmacology on 5 May 2020 by Zhao, T. M., Wang, Y., et al. XPD inhibits cell growth and invasion and enhances chemosensitivity in esophageal squamous cell carcinoma by regulating the PI3K/AKT signaling pathway. In International Journal of Molecular Medicine on 4 May 2020 by Jian, J., Li, S., et al. MD2 activation by direct AGE interaction drives inflammatory diabetic cardiomyopathy. In Nature Communications on 1 May 2020 by Wang, Y., Luo, W., et al. Neuroprotective effects of FK866 against traumatic brain injury: Involvement of p38/ERK pathway. In Annals of Clinical and Translational Neurology on 1 May 2020 by Tan, Z., Chen, L., et al.

Vinculin (Cell Signaling Technologies, Catalog #13901). Validated by IHC and western blotting. Species Reactivity: Chlorocebus sabaeus (Green monkey) Homo sapiens (Human)Mus musculus (House mouse)Rattus norvegicus (Rat). 76 citations have been found for this product. Some of the publications as follows. Temozolomide antagonizes oncolytic immunovirotherapy in glioblastoma. In Journal for Immunotherapy of Cancer on 1 May 2020 by Saha, D., Rabkin, S. D., et al. Kinase inhibition profiles as a tool to identify kinases for specific phosphorylation sites. In Nature Communications on 3 April 2020 by Watson, N. A., Cartwright, T. N., et al. Oncogenic KrasG12D causes myeloproliferation via NLRP3 inflammasome activation. In Nature Communications on 3 April 2020 by Hamarsheh, S., Osswald, L., et al. Restoration of MARCKS enhances chemosensitivity in cancer. In Journal of Cancer Research and Clinical Oncology on 1 April 2020 by Wenzel, T., Büch, T., et al. Pharmacological ascorbate induces 'BRCAness' and enhances the effects of Poly(ADP-Ribose) polymerase inhibitors against BRCA1/2 wild-type ovarian cancer. In Oncology Letters on 1 April 2020 by Ma, Y., Chen, P., et al. Adverse maternal environment and western diet impairs cognitive function and alters hippocampal glucocorticoid receptor promoter methylation in male mice. In Physiological Reports on 1 April 2020 by Ke, X., Fu, Q., et al. TNF-Receptor-1 inhibition reduces liver steatosis, hepatocellular injury and fibrosis in NAFLD mice. In Cell Death & Disease on 31 March 2020 by Wandrer, F., Liebig, S., et al. Flow-induced Shear Stress Confers Resistance to Carboplatin in an Adherent Three-Dimensional Model for Ovarian Cancer: A Role for EGFR-Targeted Photoimmunotherapy Informed by Physical Stress. In Journal of Clinical Medicine on 28 March 2020 by Nath, S., Pigula, M., et al. D609 protects retinal pigmented epithelium as a potential therapy for age-related macular degeneration. In Signal Transduction and Targeted Therapy on 21 March 2020 by Wang, B., Wang, L., et al. Long noncoding RNA AGPG regulates PFKFB3-mediated tumor glycolytic reprogramming. In Nature Communications on 20 March 2020 by Liu, J., Liu, Z. X., et al..

Beta-Actin (Cell Signaling Technologies, Catalog # 3700). Orthogonal validation for IHC and Western blotting. Reactivity: Chlorocebus sabaeus (Green monkey) Homo sapiens (Human)Mus musculus (House mouse)Rattus norvegicus (Rat). 1,449 citations have been found for this product. Some publications as follows. Discovery of novel ATAD2 bromodomain inhibitors that trigger apoptosis and autophagy in breast cells by structure-based virtual screening. In Journal of Enzyme Inhibition and Medicinal Chemistry on 1 December 2020 by Yao, D., Zhang, J., et al. Investigation of the role and mechanism of ARHGAP5-mediated colorectal cancer metastasis. In Theranostics on 3 June 2020 by Tian, T., Chen, Z. H., et al. Adipocytes promote tumor progression and induce PD-L1 expression via TNF-α/IL-6 signaling. In Cancer Cell International on 2 June 2020 by Li, Z., Zhang, C., et al. ROS-Mediated Apoptotic Cell Death of Human Colon Cancer LoVo Cells by Milk δ-Valerobetaine. In Scientific Reports on 2 June 2020 by D'Onofrio, N., Cacciola, N. A., et al. Total saponin of Dioscorea collettii attenuates MSU crystal‑induced inflammation via inhibiting the activation of the NALP3 inflammasome and caspase‑1 in THP‑1 macrophages. In Molecular Medicine Reports on 1 June 2020 by Wang, L., Zhu, L., et al. TCRP1 induces tamoxifen resistance by promoting the activation of SGK1 in MCF‑7 cells. In Oncology Reports on 1 June 2020 by Zhao, S., Li, X., et al. MicroRNA-125b as a tumor suppressor by targeting MMP11 in breast cancer. In Thoracic Cancer on 1 June 2020 by Wang, Y., Wei, Y., et al. BRD4 Inhibition Protects Against Acute Pancreatitis Through Restoring Impaired Autophagic Flux. In Frontiers in Pharmacology on 28 May 2020 by Shen, S., Li, B., et al. Chronic Restraint Stress Induces Gastric Mucosal Inflammation with Enhanced Oxidative Stress in a Murine Model. In Psychology Research and Behavior Management on 23 May 2020 by Yisireyili, M., Alimujiang, A., et al. TNFAIP8 controls murine intestinal stem cell homeostasis and regeneration by regulating microbiome-induced Akt signaling. In Nature Communications on 22 May 2020 by Goldsmith, J. R., Spitofsky, N., et al.

p38alpha MAPK (L53F8) (Cell Signaling, Catalog #9228). Orthogonal validation. Applications for western blotting, IHC, FACs Reactivity: Chlorocebus sabaeus (Green monkey)Homo sapiens (Human)Mus musculus (House mouse)Rattus norvegicus (Rat)Sus scrofa domesticus (Pig). 42 citations have been found for this product. Some publications as follows. Evidence for functional selectivity in TUDC- and norUDCA-induced signal transduction via α5β1 integrin towards choleresis. In Scientific Reports on 2 April 2020 by Bonus, M., Sommerfeld, A., et al. Activation of the pattern recognition receptor NOD1 augments colon cancer metastasis. In Protein Cell on 1 March 2020 by Jiang, H. Y., Najmeh, S., et al. Mechanically activated Piezo1 channels of cardiac fibroblasts stimulate p38 mitogen-activated protein kinase activity and interleukin-6 secretion. In The Journal of Biological Chemistry on 15 November 2019 by Blythe, N. M., Muraki, K., et al. Ubiquitination of RIPK1 suppresses programmed cell death by regulating RIPK1 kinase activation during embryogenesis. In Nature Communications on 13 September 2019 by Zhang, X., Zhang, H., et al. Targeting F-Box Protein Fbxo3 Attenuates Lung Injury Induced by Ischemia-Reperfusion in Rats. In Frontiers in Pharmacology on 11 June 2019 by Hung, K. Y., Liao, W. I., et al. NADPH oxidase 2 inhibitors CPP11G and CPP11H attenuate endothelial cell inflammation & vessel dysfunction and restore mouse hind-limb flow. In Redox Biology on 1 April 2019 by Li, Y., Cifuentes-Pagano, E., et al. NAD+ metabolism governs the proinflammatory senescence-associated secretome. In Nature Cell Biology on 1 March 2019 by Nacarelli, T., Lau, L., et al. Radix Sophorae Flavescentis inhibits proliferation and induces apoptosis of AGS human gastric cancer cells. In Molecular Medicine Reports on 1 March 2019 by Kim, J. S., Shin, S. J., et al. Cardiac fibroblast-specific p38α MAP kinase promotes cardiac hypertrophy via a putative paracrine interleukin-6 signaling mechanism. In The FASEB Journal on 1 September 2018 by Bageghni, S. A., Hemmings, K. E., et al. Chaihu-Shugan-San exerts an antidepressive effect by downregulating miR-124 and releasing inhibition of the MAPK14 and Gria3 signaling pathways. In Neural Regeneration Research on 1 May 2018 by Liu, Q., Sun, N. N., et al.

Phospho-Smad1 (Ser463/465), Smad5 (Ser463/465), and Smad9 (Ser465/467) (D5B10) (Cell Signaling, Catalog #13820). Validation by biological strategies for western blotting, IHC, IF and ICC. Reactivity Homo sapiens (Human)Mus musculus (House mouse)Rattus norvegicus (Rat). 138 citations have been found for this product. Some publications as follow.

Msx2 plays an important role in BMP6-induced osteogenic differentiation of two mesenchymal cell lines: C3H10T1/2 and C2C12.

In Regenerative Therapy on 1 June 2020 by Cai, C., Wang, J., et al. Integration of Nodal and BMP Signaling by Mutual Signaling Effector Antagonism. In Cell Reports on 7 April 2020 by Soh, G. H., Pomreinke, A. P., et al. BMP Signaling Gradient Scaling in the Zebrafish Pectoral Fin. In Cell Reports on 24 March 2020 by Mateus, R., Holtzer, L., et al. BMP-SMAD1/5 Signaling Regulates Retinal Vascular Development. In Biomolecules on 23 March 2020 by Benn, A., Alonso, F., et al. Mutant ACVR1 Arrests Glial Cell Differentiation to Drive Tumorigenesis in Pediatric Gliomas. In Cancer Cell on 16 March 2020 by Fortin, J., Tian, R., et al. Dullard-mediated Smad1/5/8 inhibition controls mouse cardiac neural crest cells condensation and outflow tract septation. In eLife on 27 February 2020 by Darrigrand, J. F., Valente, M., et al. BMP4 induces asymmetric cell division in human glioma stem-like cells. In Oncology Letters on 1 February 2020 by Koguchi, M., Nakahara, Y., et al. BMP4 gene therapy enhances insulin sensitivity but not adipose tissue browning in obese mice. In Molecular Metabolism on 1 February 2020 by Hoffmann, J. M., Grünberg, J. R., et al. BMP9 mediates the anticancer activity of evodiamine through HIF‑1α/p53 in human colon cancer cells. In Oncology Reports on 1 February 2020 by Li, F. S., Huang, J., et al. Mice Lacking the Matrilin Family of Extracellular Matrix Proteins Develop Mild Skeletal Abnormalities and Are Susceptible to Age-Associated Osteoarthritis. In International Journal of Molecular Sciences on 19 January 2020 by Li, P., Fleischhauer, L., et al.

anti-rabbit IgG (H+L), F (ab')2 Fragment (Alexa Fluor 488 Conjugate) (Cell Signaling, Catalog#4412). Validation not available. Species Cross-Reactivity Key: H-Human M-Mouse R-Rat Hm-Hamster Mk-Monkey Mi-Mink C-Chicken Dm-D. melanogaster X-Xenopus Z-Zebrafish B-Bovine Dg-Dog Pg-Pig Sc-S. cerevisiae Ce-C. elegans Hr-Horse All-All Species Expected. Application Key: W-Western IP-Immunoprecipitation IHC-Immunohistochemistry ChIP-Chromatin Immunoprecipitation IF-Immunofluorescence F-Flow Cytometry E-P-ELISA-Peptide. 309 citations have been found for this product. Some publications as follows. Long noncoding RNA ZFAS1 promoting small nucleolar RNA-mediated 2'-O-methylation via NOP58 recruitment in colorectal cancer. In Molecular Cancer on 22 May 2020 by Wu, H., Qin, W., et al. TNFAIP8 controls murine intestinal stem cell homeostasis and regeneration by regulating microbiome-induced Akt signaling. In Nature Communications on 22 May 2020 by Goldsmith, J. R., Spitofsky, N., et al. Plasticity of nuclear and cytoplasmic stress responses of RNA-binding proteins. In Nucleic Acids Research on 21 May 2020 by Backlund, M., Stein, F., et al. Sonic Hedgehog Signaling Agonist (SAG) Triggers BDNF Secretion and Promotes the Maturation of GABAergic Networks in the Postnatal Rat Hippocampus. In Frontiers in Cellular Neuroscience on 20 May 2020 by Delmotte, Q., Diabira, D., et al. Identification and functional activity of matrix-remodeling associated 5 (MXRA5) in benign hyperplastic prostate. In Aging (Albany NY) on 11 May 2020 by Xiao, H., Jiang, Y., et al. Parameter tuning differentiates granule cell subtypes enriching transmission properties at the cerebellum input stage. In Communications Biology on 8 May 2020 by Masoli, S., Tognolina, M., et al. Discrete functional and mechanistic roles of chromodomain Y-like 2 (CDYL2) transcript variants in breast cancer growth and metastasis. In Theranostics on 7 May 2020 by Yang, L. F., Yang, F., et al. Exosomes Derived from Bone Marrow Stromal Cells (BMSCs) Enhance Tendon-Bone Healing by Regulating Macrophage Polarization. In Medical Science Monitor: International Medical Journal of Experimental and Clinical Research on 5 May 2020 by Shi, Y., Kang, X., et al. AREG mediates the epithelial‑mesenchymal transition in pancreatic cancer cells via the EGFR/ERK/NF‑κB signalling pathway. In Oncology Reports on 1 May 2020 by Wang, L., Wang, L., et al. Dissolved oxygen from microalgae-gel patch promotes chronic wound healing in

diabetes. In Science Advances on 1 May 2020 by Chen, H., Cheng, Y., et al.

anti-mouse IgG (H+L), F(ab')2 Fragment (Alexa Fluor 488 Conjugate) (Cell Signaling, Catalog #4408). Validation not available. Species Cross-Reactivity Key: H-Human M-Mouse R-Rat Hm-Hamster Mk-Monkey Mi-Mink C-Chicken Dm-D. melanogaster X-Xenopus Z-Zebrafish B-Bovine Dg-Dog Pg-Pig Sc-S. cerevisiae Ce-C. elegans Hr-Horse All-All Species Expected. Application Key: W-Western IP-Immunoprecipitation IHC-Immunohistochemistry ChIP-Chromatin Immunoprecipitation IF-Immunofluorescence F-Flow Cytometry E-P-ELISA-Peptide. 176 citations have been found for this product. Some publications as follows. Airway Epithelial Cell Immunity Is Delayed During Rhinovirus Infection in Asthma and COPD. In Frontiers in Immunology on 6 June 2020 by Veerati, P. C., Troy, N. M., et al. Comparative exome sequencing reveals novel candidate genes for retinitis pigmentosa. In EBioMedicine on 23 May 2020 by Yi, Z., Ouyang, J., et al. Pharmacological inhibition of PRMT7 links arginine monomethylation to the cellular stress response. In Nature Communications on 14 May 2020 by Szewczyk, M. M., Ishikawa, Y., et al. Discrete functional and mechanistic roles of chromodomain Y-like 2 (CDYL2) transcript variants in breast cancer growth and metastasis. In Theranostics on 7 May 2020 by Yang, L. F., Yang, F., et al. Isoliquiritigenin Attenuates UUO-Induced Renal Inflammation and Fibrosis by Inhibiting Mincle/Syk/NF-Kappa B Signaling Pathway. In Drug Design, Development and Therapy on 29 April 2020 by Liao, Y., Tan, R. Z., et al. D609 protects retinal pigmented epithelium as a potential therapy for age-related macular degeneration. In Signal Transduction and Targeted Therapy on 21 March 2020 by Wang, B., Wang, L., et al. β‑asarone modulates Beclin‑1, LC3 and p62 expression to attenuate Aβ40 and Aβ42 levels in APP/PS1 transgenic mice with Alzheimer's disease. In Molecular Medicine Reports on 13 March 2020 by Deng, M., Huang, L., et al. Luteolin Attenuates Doxorubicin-Induced Cardiotoxicity Through Promoting Mitochondrial Autophagy. In Frontiers in Physiology on 3 March 2020 by Xu, H., Yu, W., et al. A novel STAT3 inhibitor attenuates angiotensin II-induced abdominal aortic aneurysm progression in mice through modulating vascular inflammation and autophagy. In Cell Death & Disease on 18 February 2020 by Wu, Q. Y., Cheng, Z., et al. Ultrasmall CuS@BSA nanoparticles with mild photothermal conversion synergistically induce MSCs-differentiated fibroblast and improve skin regeneration. In Theranostics on 12 February 2020 by Xiao, Y., Peng, J., et al.

anti-CD16/CD32, (Cat #101301, clone 93), Validated by immunofluorescent staining with flow cytometric analysis. Application References Oliver AM, et al. 1999. Hybridoma 18:113. (Block)Brummel R and Lenert P. 2005. J. Immunol. 174:2429.Terrazas LI, et al. 2005. Int. J. Parasitol. 35:1349. (Block) Clements JL, et al. 2006. J. Immunol. 177:905.Mohamed W, et al. 2010. Infect Immun. 78:3306. Ouchi T, et al. 2011. J. Exp Med. 208:2607. Kmieciak M, et al. 2011. J. Vis. Exp. 47:2381. Yamazaki S, et al. 2012. PLoS One. 7:e51665. Li J, et al. 2012. Arthritis Rheum. 64:1098. Azuma M, et al. 2012. Oncoimmunology. 1:581. Koon HW, et al. 2013. J. Vis. Exp. 68:4208.

fluorochrome-conjugated anti-CD3 (Cat #100353, clone 145-2C11), Validated by immunofluorescent staining with flow cytometric analysis. Application References Leo O, et al. 1987. P. Natl. Acad. Sci. USA 84:1374. (IP, Activ, Block)Kruisbeek AM, et al. 1991. In Current Protocols in Immunology. 3.12.1. (Activ) Duke RC, et al. 1995. Current Protocols in Immunology. 3.17.1. Salvadori S, et al. 1994. J. Immunol. 153:5176. (WB) Payer E, et al. 1991. J. Immunol. 146:2536. (IF) Jacobs H, et al. 1994. Eur. J. Immunol. 24:934. (CMCD) Vossen ACTM, et al. 1995. Eur. J. Immunol. 25:1492. (Activ)Henrickson M, et al. 1995. Transplantation 60:828. (Deplete) Kinnaert P, et al. 1996. Transpl. Int. 9:386. (Deplete)Han WR, et al. 1999. Transpl. Immunol. 7:207. (Deplete) Miescher GC, et al. 1989. Immunol. Lett. 23:113. (Block) Terrazas LI, et al. 2005. Intl. J. Parasitology. 35:1349. (Activ)

anti-CD4 (Biolegend, Cat #100406, clone GK1.5), Validated by immunofluorescent staining with flow cytometric analysis. Application References Dialynas DP, et al. 1983. J. Immunol. 131:2445. (Block, IP) Dialynas DP, et al. 1983. Immunol. Rev. 74:29. (IP, Deplete)Wu L, et al. 1991. J. Exp. Med. 174:1617. (Costim) Godfrey DI, et al. 1994. J. Immunol. 152:4783. (Block)Gavett SH, et al. 1994. Am. J. Respir. Cell. Mol. Biol. 10:587. (Deplete) Schuyler M, et al. 1994. Am. J. Respir. Crit. Care Med. 149:1286. (Deplete) Ghobrial RR, et al. 1989. Clin. Immunol. Immunopathol. 52:486. (Deplete) Israelski DM, et al. 1989. J. Immunol. 142:954. (Deplete) Zheng B, et al. 1996. J. Exp. Med. 184:1083. (IHC)Frei K, et al. 1997. J. Exp. Med. 185:2177. (IHC) Felix NJ, et al. 2007. Nat. Immunol. 8:388. (Block)

anti-CD8 (Biolegend, Cat #100734, clone 53-6.7), Validated by immunofluorescent staining with flow cytometric analysis. Application References Ledbetter JA, et al. 1979. Immunol. Rev. 47:63. (IHC, IP) Hathcock KS. 1991. Current Protocols in Immunology. 3.4.1. (Deplete) Takahashi K, et al. 1992. P. Natl. Acad. Sci. USA 89:5557. (Block, IP)Ledbetter JA, et al. 1981. J. Exp. Med. 153:1503. (Block) Hata H, et al. 2004. J. Clin. Invest. 114:582. (IHC) Fan WY, et al. 2001. Exp. Biol. Med. 226:1045. (IHC) Shih FF, et al. 2006. J. Immunol. 176:3438. (FC) Kamimura D, et al. 2006. J. Immunol. 177:306.Bouwer HGA, et al. 2006. P. Natl. Acad. Sci. USA 103:5102. (FC, Deplete)Kao C, et al. 2005. Int. Immunol. 17:1607. Ko SY, et al. 2005. J. Immunol. 175:3309. (FC) Rasmussen JW, et al. 2006. Infect. Immun. 74:6590

anti-CD45 (Biolegend, Cat #103126, clone 30-F11), Validated by immunofluorescent staining with flow cytometric analysis. Application References Podd BS, et al. 2006. J. Immunol. 176:6532. (FC, CMCD) Haynes NM, et al. 2007. J. Immunol. 179:5099. (FC) Ledbetter JA, et al. 1979. Immunol. Rev. 47:63. (IP) Simon DI, et al. 2000. J. Clin. Invest. 105:293. (IHC) Seaman WE. 1983. J. Immunol. 130:1713. (CMCD) Cornet A, et al. 2001. P. Natl. Acad. Sci. USA 98:13306. (IHC) Tsuboi S and Fukuda M. 1998. J. Biol. Chem. 273:30680. (WB) Liu F, et al. 2012. Blood. 119:3295. Pelletier AN, et al. 2012. J. Immunol. 188:5561.

anti-CD11b (Biolegend, Cat #101226, clone M1/70), Validated by immunofluorescent staining with flow cytometric analysis. Application References Springer T, et al. 1978. Eur. J. Immunol. 8:539. (IP) Ault K and Springer T. 1981. J. Immunol. 126:359. (Deplete) Springer TA, et al. 1982. Immunol. Rev. 68:171. (Block) Ho MK and Springer TA. 1983. J. Biol. Chem. 258:2766. (IP) Flotte TJ, et al. 1983. Am. J. Pathol. 111:112. (IHC) Noel GJ, et al. 1990. J. Clin. Invest. 85:208. (IF) Allen LA and Aderem A. 1996. J. Exp. Med. 184:627 (IF) D'Amico A and Wu L. 2003. J. Exp. Med. 198:293. (Deplete) Brickson SJ, et al. 2003. Appl Physiol. 95:969. (Block) Clatworthy MR and Smith KG. 2004. J. Exp. Med. 199:717. (IF) Hata H, et al. 2004. J. Clin. Invest. 114:582. (IHC) Zhang Y, et al. 2002. J. Immunol. 168:3088. (IHC).

anti-CD11c (Biolegend, Cat #117310, clone N418), Validated by immunofluorescent staining with flow cytometric analysis. Application References Granucci F, et al. 1997. J. Immunol. 159:1794. Stokes RW, et al. 1998. J. Immunol. 160:5514. Metlay JP, et al. 1990. J. Exp. Med. 171:1753. (IHC, IP) Ma XT, et al. 2006. Cancer Research 66:1169. Chin RK, et al. 2006. J. Immunol. 177:290. (IF) Cervantes-Barragan L, et al. 2007. Blood 109:1131. (FC) Turnquist HR, et al. 2007. J. Immunol. 178:7018. (FC) Benson MJ, et al. 2007. J. Exp. Med. doi:10.1084/jem.20070719. (FC) You Y, et al. 2009. J. Immunol. 182:7343. (IF) Roland CL, et al. 2009. Mol. Cancer Res. 8:1761. (IHC, FC) Wikstrom M, et al.2006. J. Immunol. 177:913. Pericolini E, et al. 2008. J. Leukocyte Biol. 83:1286

anti-F4/80 (Biolegend, Cat #123108), Validated by immunofluorescent staining with flow cytometric analysis. Application References Schaller E, et al. 2002. Mol. Cell. Biol. 22:8035. (IHC) Stevceva L, et al. 2001. BMC Clin Pathol. 1:3. (IHC) Kobayashi M, et al.2008. J. Leukoc. Biol. 83:1354. Poeckel D, et al. 2009. J. Biol Chem. 284:21077. Glass AM, et al. 2013. J. Immunol. 190:4830. Koehm S, et al. 2007. J. Allergy Clin. Immunol. 120:570. (IHC) Rankin AL, et al. 2010. J. Immunol. 184:1526. (IHC) Sasi SP, et al. 2014. J Biol Chem. 289:14178. Thakus VS, et al. 2014. Toxicol Lett. 230:322. Watson NB, et al. 2015. J Immunol. 194:2796.

Hirakawa H, et al. 2015. PLoS One. 10:119360

anti-CD206 (Biolegend,Cat# 141716, CloneC068C2), Validated by immunofluorescent staining with flow cytometric analysis. Application References Keller J, et al. 2012. Biochem Biophys Res Commun. 417:217. Ito H, et al. 2012. J Am Soc Nephrol. 23:1797. Yang X, et al. 2015. PNAS. 112:2900. Peng K, et al. 2017. PLoS One. 10.1371/journal.pone.0183271. Morfoisse F, et al. 2018. Arterioscler Thromb Vasc Biol. 38:1346. Shigeta A et al. 2019. Developmental cell. 48(5):617-630. An YA et al. 2017. eLife. 6 pii: e24071. Ano Y, et al. 2017. J Biol Chem. 292:3720. Calvente CJ, et al. 2019. J Clin Invest. 130:4091. Wang C, et al. 2020. Mucosal Immunol. 13:22. Yang C, et al. 2019. J Transl Med. 1.004166667.

LEAF purified anti-mouse CD3ε antibody (Biolegend, Catalog# 100301, Clone 145-2C11). Validated by immunofluorescent staining with flow cytometric analysis. Application References Leo O, et al. 1987. P. Natl. Acad. Sci. USA 84:1374. (IP, Activ, Block) Kruisbeek AM, et al. 1991. In Current Protocols in Immunology. 3.12.1. (Activ) Duke RC, et al. 1995. Current Protocols in Immunology. 3.17.1. Salvadori S, et al. 1994. J. Immunol. 153:5176. (WB) Payer E, et al. 1991. J. Immunol. 146:2536. (IF) Jacobs H, et al. 1994. Eur. J. Immunol. 24:934. (CMCD) Vossen ACTM, et al. 1995. Eur. J. Immunol. 25:1492. (Activ) Henrickson M, et al. 1995. Transplantation 60:828. (Deplete) Kinnaert P, et al. 1996. Transpl. Int. 9:386. (Deplete) Han WR, et al. 1999. Transpl. Immunol. 7:207. (Deplete) Miescher GC, et al. 1989. Immunol. Lett. 23:113. (Block) Terrazas LI, et al. 2005. Intl. J. Parasitology. 35:1349. (Activ) Perchonock C, et al. 2006. Mol Cell Biol. 26:6005. Perchonock C, et al. 2007. J Immunol. 179:1768. Guo Y, et al. 2008. Blood. 112:480. Namavari A, et al. 2012. Invest Ophthalmol Vis Sci . 53:4575. Sandy A, et al. 2013. J Immunol. 190:5818. Wang G, et al. 2015. PLoS One. 10:121968. McCully M, et al. 2015. J Immunol. 195: 96 - 104. Bremser A, et al. 2015. PLoS One. 10: 0137393. Kim K, et al. 2016. PLoS One. 11: 0148576. Kojima T, et al. 2016. Sci Rep. 6:36457. Rissiek B, et al. 2017. Sci Rep. 10.1038/s41598-017-16613-w. Lin J, et al. 2017. Nat Commun. . 10.1038/s41467-017-01477-5.

LEAF purified anti-mouse CD28 antibody (Biolegend, Catalog# 102115, Clone 37.51), Validated by immunofluorescent staining with flow cytometric analysis. Application References (PubMed link indicates BioLegend citation) 1. Gross JA, et al. 1992. J. Immunol. 149:380. (IP, Costim) 2. Cibotti R, et al. 1997. Immunity 6:245. (Costim) 3. Masten BJ, et al. 1997. Am. J. Respir. Cell Mol. Biol. 16:335. (Block) 4. Nishio M, et al. 1996. J. Immunol. 157:4347. (Block) 5. Zhang N and He Y-W, 2005. J. Exp. Med. 202:395. (Costim) 6. Terrazas LI, et al. 2005. Intl. J. Parasitology. 35:1349. (Costim) 7. Perchonock CE, et al. 2006. Mol Cell Biol. 26 (16):6005. (Costim) 8. Wang W, et al. 2007. J. Immunol. 178:4885. (Costim) 9. Pua HH, et al. 2007. J. Exp. Med. 204:25. (Costim) 10. Perchonock CE, et al. 2007. J. Immunol. 179:1768. 11. Barbi J, et al. 2007. Blood 110:2215. 12. Milpied P, et al. 2011. Blood 118:2993. PubMed

InVivoMAb anti-mouse PD-1 (CD279). Clone RMP1-14. Catalog # BE0146. Validated by western blotting. Recommended for in vivo blocking of PD-1/PD-L signaling. Citations: Grasselly, C., et al. (2018). “The Antitumor Activity of Combinations of Cytotoxic Chemotherapy and Immune Checkpoint Inhibitors Is Model-Dependent.” Front Immunol 9: 2100. Triplett, T. A., et al. (2018). “Reversal of indoleamine 2,3-dioxygenase-mediated cancer immune suppression by systemic kynurenine depletion with a therapeutic enzyme.” Nat Biotechnol 36(8): 758-764. Moynihan, K. D., et al. (2016). “Eradication of large established tumors in mice by combination immunotherapy that engages innate and adaptive immune responses.” Nat Med. doi: 10.1038/nm.4200

InVivoMAb anti-mouse CTLA-4 (CD152), Clone 9D9, Catalog # BE0164. Validated by western blotting. Applications for in vivo CTLA-4 neutralization and Western blot. Citations: Dai, M., et al. (2015). “Curing mice with large tumors by locally delivering combinations of immunomodulatory antibodies.” Clin Cancer Res 21(5): 1127-1138. Zippelius, A., et al. (2015). “Induced PD-L1 expression mediates acquired resistance to agonistic anti-CD40 treatment.” Cancer Immunol Res 3(3): 236-244. Condamine, T., et al. (2014). “ER stress regulates myeloid-derived suppressor cell fate through TRAIL-R-mediated apoptosis.” J Clin Invest 124(6): 2626-2639.

Eukaryotic cell lines

Policy information about cell lines

Cell line source(s)	The 344SQ parental cell line (344SQP) was a generous gift from Dr. Jonathan Kurie (MD Anderson). 344SQP cells were established by Dr. Kurie laboratory at MD Anderson. 344SQ resistant cells (344SQR) were established from 344SQP by Dr. James Welsh laboratory (PMID: 27821490). 4T1, RAW 264.7 and EL4 cell lines were obtained from the American Type Culture Collection (ATCC; Manassas, VA, USA).
Authentication	Cell lines were authenticated using DDC Medical Services via STR analysis.
Mycoplasma contamination	Cells tested negative for mycoplasm during experimental studies.
Commonly misidentified lines (See ICLAC register)	No misidentified cell lines were used in the study.

Animals and other organisms

Policy information about studies involving animals; ARRIVE guidelines recommended for reporting animal research

Laboratory animals	Syngeneic 129Sv/Ev and BALB/c mice were used in the study. For both strains, we used 12-16 weeks old female mice. All mouse studies were approved by the Institutional Animal Care and Use Committee (IACUC) of The University of Texas MD Anderson Cancer Center before their initiation; animal care was provided according to IACUC standards, and all mice had been bred and were maintained in our own specific pathogen-free mouse colony at the Experimental Radiation Oncology (ERO) facility.
Wild animals	The study did not involve wild animals.
Field-collected samples	The study did not involve samples collected from the field.
Ethics oversight	All mouse studies were approved by the Institutional Animal Care and Use Committee (IACUC) of The University of Texas MD Anderson Cancer Center before their initiation; animal care was provided according to IACUC standards, and all mice had been

bred and were maintained in our own specific pathogen-free mouse colony.

Note that full information on the approval of the study protocol must also be provided in the manuscript.

Human research participants

Policy information about studies involving human research participants

Population characteristics	*Describe the covariate-relevant population characteristics of the human research participants (e.g. age, gender, genotypic information, past and current diagnosis and treatment categories). If you filled out the behavioural & social sciences study design questions and have nothing to add here, write "See above."*
Recruitment	*Describe how participants were recruited. Outline any potential self-selection bias or other biases that may be present and how these are likely to impact results.*
Ethics oversight	*Identify the organization(s) that approved the study protocol.*

Note that full information on the approval of the study protocol must also be provided in the manuscript.

Clinical data

Policy information about clinical studies

All manuscripts should comply with the ICMJEguidelines for publication of clinical research and a completedCONSORT checklist must be included with all submissions.

Clinical trial registration	NCT02444741; NCT02239900; NCT02402920. Full trial protocol can be accessed at https://clinicaltrials.gov/
Study protocol	2013-0882; 2014-1020; 2014-1003. The full trial protocol can be accessed at Clinicaltrials.gov
Data collection	Baseline patient characteristics, biological biomarkers, adverse events, treatment responses and survival from clinical trial were collected at MD Anderson Cancer Center. The samples from the 2013-0882 trial were collected from August, 2014-March, 2019. 2014-1020 samples were collected from September, 2015-March, 2019. 2014-1003 samples were collected from July, 2015-March, 2019.
Outcomes	The primary objective was to determine the safety of Pembrolizumab or Ipilimumab plus SBRT for Patients With Advanced Solid Tumors; rate of out-of-field objective responses (either CR or PR) of the non-irradiated disease sites, regardless of their location, thoracic or otherwise. The secondary objective was to determine the addition of radiotherapy to Pembrolizumab or Ipilimumab can improve the PFS rate compared to Pembrolizumab or Ipilimumab alone.

Flow Cytometry

Plots

Confirm that:

- [x] The axis labels state the marker and fluorochrome used (e.g. CD4-FITC).
- [x] The axis scales are clearly visible. Include numbers along axes only for bottom left plot of group (a 'group' is an analysis of identical markers).
- [x] All plots are contour plots with outliers or pseudocolor plots.
- [x] A numerical value for number of cells or percentage (with statistics) is provided.

Methodology

Sample preparation	Freshly isolated primary tumor tissues (from 2 or 3 mice/group) were washed with ice-cold PBS and digested with 250 μg/mL of Liberase TR (Roche) and 20 μg/mL DNase I (Roche) and incubated for 45 minutes at 37°C with shaking. Fetal bovine serum was added, and samples were filtered followed by Histopaque-1077 (Sigma-Aldrich) gradient isolation of TILs.
Instrument	Samples were analyzed with an LSR II flow cytometer.
Software	FlowJo software, version 10
Cell population abundance	for sorting CD4 T-cells we used magnetic bead separation methodology then validated the purity by flow cytometry (96% pure).
Gating strategy	For lymphoid population we gated on lymphocytes first then on CD45+ population then on CD4 and CD8 T-cells. For myeloid population we gated on CD45+ then on Gr1+ CD11b+. M2 macrophages were gated on CD206+ population.

- [x] Tick this box to confirm that a figure exemplifying the gating strategy is provided in the Supplementary Information.
